# Impact of Substitution Pattern and Chain Length on the Thermotropic Properties of Alkoxy-Substituted Triphenyl-Tristriazolotriazines †

**DOI:** 10.3390/molecules25235761

**Published:** 2020-12-07

**Authors:** Thorsten Rieth, Natalie Tober, Daniel Limbach, Tobias Haspel, Marcel Sperner, Niklas Schupp, Philipp Wicker, Stefan Glang, Matthias Lehmann, Heiner Detert

**Affiliations:** 1 Department for Chemistry, Johannes Gutenberg-University, 55099 Mainz, Germany; thorstenrieth@gmx.de (T.R.); ntober@students.uni-mainz.de (N.T.); Limbach@ghc.de (D.L.); t.haspel@gmx.de (T.H.); marcel.sperner@outlook.de (M.S.); nschupp@students.uni-mainz.de (N.S.); pwicker@students.uni-mainz.de (P.W.); stglang@gmx.de (S.G.); 2 Institute for Organic Chemistry, Julius-Maximilians-University, 97074 Würzburg, Germany

**Keywords:** star-shaped compounds, discotic liquid crystals, X-ray diffraction, differential scanning calorimetry, polarizing optical microscopy, swallow-tail, heterocycles, structure–property relation

## Abstract

Tristriazolotriazines (TTTs) with a threefold alkoxyphenyl substitution were prepared and studied by DSC, polarized optical microscopy (POM) and X-ray scattering. Six pentyloxy chains are sufficient to induce liquid-crystalline behavior in these star-shaped compounds. Thermotropic properties of TTTs with varying substitution patterns and a periphery of linear chains of different lengths, branching in the chain and swallow-tails, are compared. Generally, these disks display broad and stable thermotropic mesophases, with the tangential TTT being superior to the radial isomer. The structure–property relationships of the number of alkyl chains, their position, length and structure were studied.

## 1. Introduction

**Figure 1:**
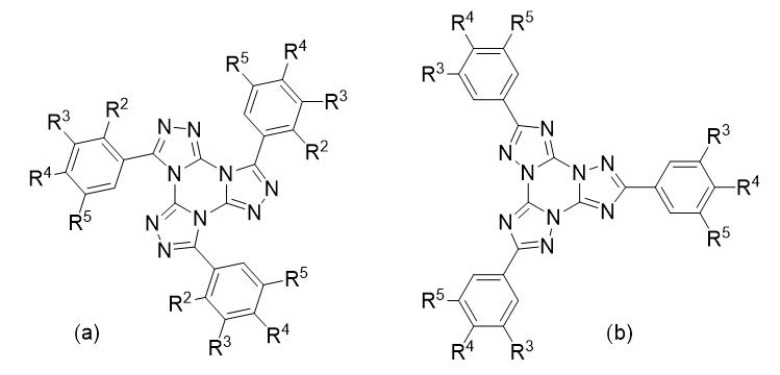
Tangential tristriazolotriazines (*t*-TTTs) (**a**) and propeller tristriazolotriazines (*r*-TTTs) (**b**) and the substitution pattern.

Tristriazolotriazines (TTTs), with a threefold alkoxyphenyl substitution [1,2,3,4] (Figure 1), not only belong to the youngest classes of discotic liquid crystals (DLCs) [5,6,7,8,9,10,11,12,13,14,15,16] but also to the small subgroup of DLCs with a heterocyclic [17,18,19,20,21,22,23,24,25,26] and electron-deficient core [27,28,29,30,31,32,33,34,35,36]. Two C_3_-symmetrical isomeric forms of these star-shaped molecules are known: the paddle-wheel with phenyl substituents in tangential positions (*t*-TTT) **1**, and, if the aromatic rings are attached to the radial positions, the propeller (*r*-TTT) **2** (Figure 1). This ostensible small change from tangential to radial substitution severely alters the mesomorphic properties. The *t*-TTT is a reliable core for discotic liquid crystals with broad mesophases [1,2,3,4,37,38,39,40], and phenyl substituents on this isomer are twisted out of the mean plane by 21–80° [41]. Isomerization to the *r*-TTT provokes a flattening of the aromatic part and facilitates crystallization. Reduced or even complete loss of mesomorphism is the typical consequence for 3,4- and 3,5-dialkoxy derivatives [42]. On the contrary, derivatives with a 3,4,5-trialkoxy periphery have lower melting points and broader phases. Although calamitic LCs have contributed to a broad range of technical innovations, discotic LCs have only found selected applications, such as in optical compensating films or sensors [9]. Understanding the structure and molecular dynamics is not a trivial task. The comprehension of structure–property relationships is the key knowledge to tailor mesomorphic, optical and electrical properties for the application of DLCs as anisotropic organic semiconductors in organic field-effect transistors, light-emitting diodes or photovoltaic devices [13]. Columnar phases of DLCs comprise stacks of disk-like molecules arranged on a regular 2D-lattice. Redox potentials and charge carrier mobilities are central properties for electronic applications; the latter is highly dependent on intermolecular order, allowing orbital overlap, e.g., in staggered cofacial conformations [14] or helical arrangements [15]. According to a theoretical study on columnar mesophases of TTTs with an electron-rich periphery, a high number of π-stacking contacts [16] render an ambipolar charge transport in TTTs on two main charge transport channels. Cyclic voltammetry of alkoxyphenyl TTTs showed only nonreversible oxidation [2], whereas the TTT core was shown to have electron-transporting characteristics [43]. The hole-transporting HOMO orbitals are located in the peripheral region; the LUMO orbitals, located in the central TTT, take the electron transport. From impedance spectroscopy, it is concluded that the conductivity in the columnar phase of dialkoxy-TTTs is mostly ionic (low-frequency regime), probably due to ionic impurities [2].

The established option for the mesophase engineering of DLCs is the variation of the periphery. The number, length, position, composition and structure of flexible side chains form a highly advantageous toolbox for custom-made mesomorphism and have been extensively studied, e.g., on triphenylenes [44,45], star systems [46,47,48,49] and macrocyclic discs [50,51,52,53,54,55]. Though some general trends have been found and successfully used for the prediction of liquid crystal (LC) behavior, they are valid only for a certain core [7]. Furthermore, the scientist is often rewarded with nonlinearities and surprising exceptions. The objective of this report is to elucidate the correlation between the mesomorphism of TTTs and the composition of their periphery. This includes the number of side chains, the variation of their position, their length and branching.

## 2. Results

### 2.1. Synthesis

**Scheme 1:**
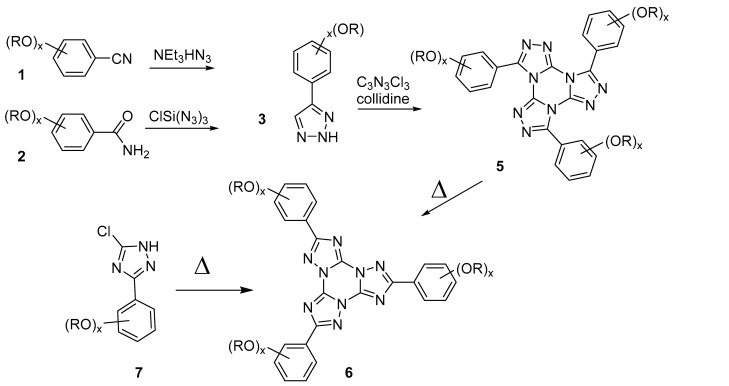
Synthesis of *t*-TTTs and thermal rearrangement to *r*-TTTs.

The only effective synthesis of *t*-TTTs is the threefold acylation/ring transformation of tetrazoles with cyanuric chloride (Scheme 1), according to Huisgen [56]. A few compounds require minor modifications, but the main problem is the chromatographic separation of diaryl triazoles **3** formed as by-products. Alkoxyaryl-tetrazoles **6** are accessible via the addition of azide to alkoxybenzonitriles **4** or, if the respective hydroxybenzonitrile is not available, via the dehydration/azidination of alkoxybenzamides **5** with triazidochlorosilane [57]. Despite earlier accounts [1,2], *t*-TTTs **1** are sensitive to high temperatures. A thermal isomerization converts **1** to its isomer **2** with different connectivity of the triazole rings, shifting the tangentially oriented aryl rings to radial positions. The cyclocondensation of chlorotriazoles **7** is the direct route to the isomeric *r*-TTTs **2** [58,59]. Besides limited accessibility of the particular chlorotriazole, poor yields in the cyclocondensation step reduce the synthetic value of this method. Therefore, nearly all *r*-TTTs known today were obtained by thermal isomerization of the tangential isomer [42].

### 2.2. Thermal Properties of 1-n-alkyloxy-t-TTTs

Some homologous series of *t*-TTTs with one to three linear alkyloxy were studied. Table 1 collects the TTTs together with their thermal characteristics. The order follows the substitution pattern (see Figure 1) and increasing chain length. Some compounds are reported in the literature, and the preparation and analytical data of new TTTs are provided in the Supporting Information (SI). Thermal properties were studied by differential scanning calorimetry (DSC) and polarized optical microscopy (POM), and structure determination of the mesophases was performed on representative compounds via X-ray diffraction (XRD). Details on equipment are given in the SI.

Triphenyl-*t*-TTT ***t*-1** is a sparingly soluble and high-melting compound (360 °C) [56]. Its *r*-isomer ***r***-**1** melts even higher: 390–400 °C [58]. The attachment of *p*-alkoxy chains significantly reduces the melting point (***t*-2**: O-C_3_H_7_: >275 °C to 76 °C for O-C_10_H_21_ (***t*-5**), but 78 °C for O-C_13_H_27_, ***t*-7**). Although these compounds are not mesomorphous, they can form stable glasses with T_g_ in the range of 35–60 °C [1,2,3,4]. A comparison of the position of the alkyl chain, e.g., for decyloxy-*t*-TTTs, reveals a strong impact: whereas 4-substituted TTT ***t*-5** melts at 76 °C, its 3-isomer ***t*-8** at 134 °C and the 2-isomer ***t*-9** is a viscous oil SI. The steric effect of the ortho-alkoxy group, pointing into the center of the disks, is probably the reason for the lack of mesomorphism of TTTs with a 2,3-, 2,4- and 2,5-didecyloxy substitution (***t*-10**–***t*-12**) SI. This appears to be valid even for TTTs with an extended π-system, e.g., ***t*-45** (Table 2).

**Figure 2:**
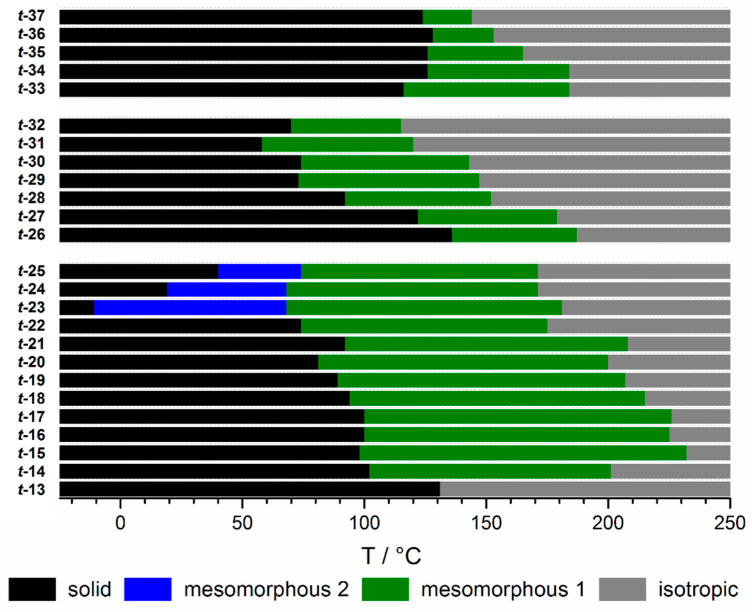
Phase behavior of 3,4-di- (***t*-13**–***t*-25**), 3,5-di- (***t*-26**–***t*-32**) and 3,4,5-trialkoxyphenyl (***t*-33**–***t*-37**)-substituted *t*-TTTs depending on chain length.

The thermal behavior of 3,4-dialkoxyphenyl-substituted *t*-TTTs is completely different. This pattern is almost a warranty for mesomorphism (Table 1, Figure 2). The width of the mesophase is typically higher than ΔT = 100 K at a maximum of 192 K (***t*-23**). Although the 3,4-dibutoxy derivative melts at 132 °C without any sign of mesomorphism, the higher homologs from 3,4-dipentyloxy (***t*-14**: Cr 102 M 201 I), at least up to 3,4-dioctadecyloxy ***t*-25**, are discotic liquid crystals ([38,42], SI). With increasing chain length, the clearing point successively drops from 232 °C (dihexyloxy, ***t*-16**) to 171 °C (dioctadecyloxy, ***t*-28**), and the melting points from 102 °C (dipentyloxy, ***t*-14**) to 74 °C (ditridecyloxy, ***t*-22**). Further chain elongation gives a surprising result. With the formation of an additional mesophase M_2_ (POM and DSC) below the typical LC range, e.g., with two tetradecyloxy chains (***t*-23**), the melting transition occurs at −11 °C with an enthalpy of 46.3 kJ/mol and an M_2_–M_1_ transition at 68 °C (9.4 kJ/mol). This phase is stable up to 181 °C (5.4 kJ/mol). Elongation of the side chains by two or four methylene groups (***t*-24**, ***t*-25**) gives a similar phase behavior. Whereas the melting points increase significantly (T_m_ = 19 °C; 40 °C), the M_2_-M_1_ transition (T_t_ = 68 °C; 74 °C) and the clearing point are less affected (T_c_ = 181 °C; 171 °C). The phenomenon of the emergence of a second mesophase with increasing chain length has been observed earlier, e.g., with triphenylene hexaesters and hexabenzoates [44,45]. Both the TTT and triphenylene series have a columnar hexagonal structure of the upper LC phase in common. However, although the transitions from the first to second mesophase in the triphenylene series occur within the typical LC range, the additional mesomorphic phase of *t*-TTTs appears below this temperature regime.

Miscibility of homologous *t*-TTTs allows the tuning of the phase transitions. Even a mixture of 25 mol % of nonmesomorphous butoxy-TTT ***t*-13** and 75 mol % decyloxy-substituted DLC ***t*-19** displays a LC phase Cr 75 M 176, only ΔT = 13 K lower and ΔT = 31 K narrower compared to pure ***t*-19**.

**Figure 3:**
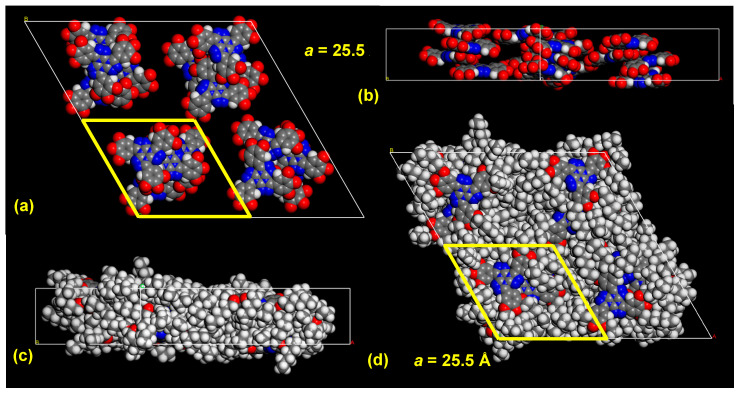
Model of a supercell (2*a* × 2*a* × 4*c*) of ***t*-33**. (**a**,**b**) Top and side view, where hexyl chains are omitted; (**c**,**d**) side and top view, including hexyl chains.

The series with the isomeric 3,5-dialkoxy pattern (***t*-26**-***t*-32**) [60] shows a more pronounced descent of phase transitions with increasing chain length ([60] SI). With decyl chains ***t*-29**, the melting point reaches a plateau at about 72 °C. The width of the typically columnar hexagonal LC phase is about half as broad as found for the 3,4-isomers (Table 1, Figure 2). It increases from hexyl ***t*-26** (ΔT = 50 K) to decyl ***t*-29** (ΔT = 74 K) and decreases to ΔT = 45 K for hexadecyl ***t*-32**. An additional bromine atom between dodecyloxy chains ***t*-31** reduces the melting point and slightly destabilizes the mesophase (ΔT = 62 K vs. ΔT = 68 K, ***t*-30**) SI. Adding a third alkoxy chain in the four-position of ***t*-30** enhances the melting point and leads to the also mesomorphous tris(3,4,5-trialkoxyphenyl) *t*-TTTs, generally with a columnar hexagonal-disordered structure ([37,42], SI). Tris-(3,4,5-trihexyloxyphenyl) substitution (***t*-33**) gives rise to a broad mesophase (Cr 116 Col_hd_ 184 I) [37,42]. According to XRD, the hexagonal columnar phase has cell parameters of a = 25.5 Å and *d* = 3.4 Å at 20 °C and in the mesophase at 145 °C. These values are *a* = 24.2 Å and d = 3.9 Å at 20 °C. An ensemble of 16 molecules in a 2*a* × 2*a* × 4*c* hexagonal supercell of ***t*-33** consisting of 16 unit cells was modeled with the program Accelrys Materials Studio 2017 R2 using the Compass2 force field [61]. In order to highlight the stabilization relative to 16 single mesogens, the energy differences were calculated for this model. As a result of the packing the valence energy increased by 500 kcal/mol, but was opposed to an energy gain of −1615 kcal/mol for the van der Waals and −448 kcal/mol for the electrostatic energy. The stabilization by nonbonding intermolecular interactions was achieved by an offset of the cores from the center of the column. Figure 3 indicates that there is a tendency to locate the peripheral trialkoxy phenyl ring above the triazine center.

Chain elongation raises the melting point to a plateau at ca. 126 °C, but the width of the mesophase successively shrinks to ΔT = 20 K for hexadecyl chains ***t*-37** due to decreasing clearing points. A comparison of the number of methylene groups in the periphery of TTTs reveals that 3,5-and 3,4,5-substituted TTTs with a similar molecular weight show a similar phase width, e.g., 3,5-dihexadecyl ***t*-32**: ΔT = 39 K and 3,4,5-tri-decyl ***t*-35**: ΔT = 45 K and 3,5-didodecyl ***t*-30**: ΔT = 69 K and 3,4,5-tri-octyl ***t*-34**: ΔT = 58 K. Cammidge reported that the exchange of a single hexyloxy chain on triphenylene DLC HAT6 versus bromine dilated the width of the LC phase by almost a factor of three [62]. A comparison of ***t*-36** (Cr 128 Col 153 I) and the didodecyloxy-bromo compound ***t*-31** (Cr 58 M 120 I) gives a proportionate augmentation. Although the 3,5-pattern guarantees lower melting and clearing points, the “less symmetrical” 3,4-series gives the broadest mesophases. Hexagonal structures and π-π-distances of about 3.48–3.8 Å are common features of the mesophases of the 3,4-, 3,5- and 3,4,5-substituted TTTs.

### 2.3. Thermal Properties of t-TTTs with Extended π-Systems and a 1-n-alkyloxy Periphery

TTTs with extended π-systems have been prepared as new materials for organic electronics, fluorophores and nonlinear optical materials. Like in the phenyl series, linear alkoxysubstitution can induce mesomorphism of TTTs with additional phenyl-, styryl- or phenylethynyl groups.

The distension of the rigid part by the elongation of the phenyl arms to naphthyl, biphenylyl, tolane or stilbene improves the π-π interaction and heightens the melting points, e.g., ***t*-5**–***t*-38** (4-decyloxyphenyl–6-decyloxynaphthyl, ΔT = +45 K) and ***t*-3**–***t*-49** (4-hexyloxyphenyl–4’-hexyloxytolanyl, ΔT = +115 K) (SI). However, vicinal dialkoxy substitution in the lateral position is beneficial for the mesomorphism of 3,4-dialkoxyphenyl-*t*-TTTs ([38], SI); even after extension of the rigid part, naphthyl replacing phenyl, these disks form broad LC phases. From 5,6-di(octyloxy)naphth-2-yl ***t*-39** to the dodecyl homolog ***t*-41**, the melting point drops from 112 °C to 59 °C, and the LC phase widens from 85 °C to 130 °C. ***t*-41** forms a hexagonal mesophase with *a* = 30.8 Å and a π-π-distance of 3.6 Å [38]. A further augmentation, naphth-2-yl to biphenyl-4-yl, destabilizes the LC phase, mainly due to enhanced melting points [38]. Although the 3’,4’-dioctyl and -decyl derivatives ***t*-42**, ***t*-43** are not mesomorphic, dodecyl chains ***t*-44** are sufficient for a broad LC phase from T = 89 °C to 198 °C. Further extension of the rigid part, stilbene (***t*-47**, ***t*-48**), tolane (***t*-50, *t*-51**) and phenylethynyltolane (***t*-52**) replacing phenyl can give DLCs ([4,37] SI). Whereas 3,5-dihexyloxy is insufficient for mesomorphism of stilbene derivative ***t*-47**, the 3,4,5-trihexyloxy periphery ***t*-48** results in a narrow mesophase at high temperatures (Cr 175 M 200 I). This pattern with slightly longer octyl chains allows mesomorphism even of the extended phenylethynyltolane TTT ***t*-52** (Cr 159 M 204 I). POM suggests a discotic nematic structure for the 45 K broad LC phase. The shorter homologs with tolane units and three octyl or dodecyl chains, ***t*-50 *t*-51**, have nearly identical melting points (82 °C, 80 °C), about ΔT ≈ 45 K lower than their phenyl homologs ***t*-34**, ***t*-36**. Unfortunately, the tolane derivatives are not mesomorphous.

### 2.4. Thermal Properties of t-TTTs with Branched Side Chains, Swallow-Tails or Chains of Different Lengths

Chain branching is another useful concept to modify the thermal properties of calamitic and discotic liquid crystals. This approach is particularly useful to reduce melting and clearing points, notably for large-core mesogens [63,64,65,66,67,68,69,70,71]. Two different strategies are applied: short-chain branching [35,36,72,73,74,75,76,77,78,79] like 3,7-dimethyloctyl or 2-ethylhexyl and Weissflogs swallow-tails [80,81,82,83,84,85].

**Figure 4:**
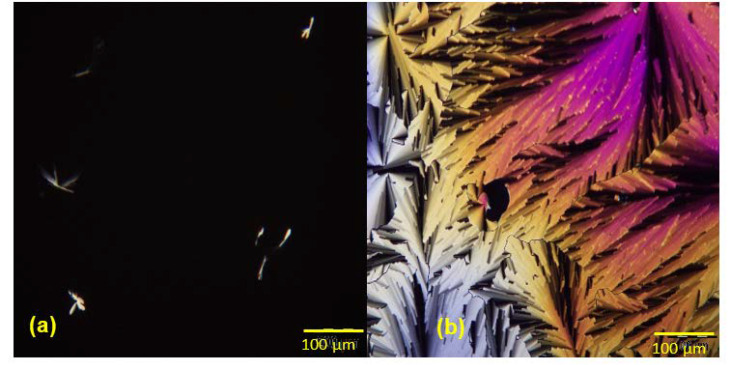
(**a**): Homeotropic growth of ***t*-60** at 135 °C (cooling rate: 1 °C/min); (**b**): fan textures at 137 °C (cooling rate: 1 °C/min).

Short-chain branching has a strong impact on thermal properties, even on nonmesomorphous 4-alkoxyphenyl-TTTs (Table 3). Although *p*-decyloxy TTT ***t*-5** melts at 76 °C, the diastereotopic mixture of the *p*-(3,7-dimethyloctyloxy) derivative ***t*-53** is a viscous oil. (SI) On the other hand, ***t*-54**, with a voluminous but rigid neomenthyloxy periphery, is crystalline up to 185 °C; if the voids in the crystal are filled with suitable solvent molecules, the crystal phase is stable up to 211 °C [41]. Short-chain branching has a similar effect in the 3,4-dialkoxy series: change from n-octyloxy (***t*-17**, T_c_ = 223 °C) to 3-octyloxy (***t*-55**, T_c_ = 158 °C) or 2-ethylhexyloxy (***t*-56**, T_c_ = 157 °C) provokes depression of the clearing point of nearly ΔT = 70 K and even more of melting points. Concerning longer chains, the impact of branching is leveled. Relative to the linear 3,4-didecyloxy TTT (***t*-29**,T_c_ = 207 °C), clearing of 4-ethyloctyl-, as well as the 3,7-dimethyloctyl analog ***t*-57**, ***t*-58** is reduced to T_c_ = 182 °C (SI) for ***t*-59** with enantiopure (*R*)-citronellyl chains T_c_ = 167 °C [1]. Laschat reported a beneficial effect on the phase width of short-chain branching in the δ-position on triphenylene systems [76]. Unfortunately, this cannot be generalized since the 3,4,5-trialkoxy derivatives ***t*-34**, ***t*-35** are mesomorphous, the branched isomers ***t*-61**, ***t*-62** are not (SI). The LC phase of ***t*-60** with the nonchiral and more rigid 3,3-dimethyloctyl chains is stable only until 154 °C and crystallization occurs on the timescale of months (first heating: T_m_ = 56 °C; T_g_ = 8 °C). Depending on the cooling rate, POM textures either show a homeotropic growth or fan structure (Figure 4).

**Figure 5:**
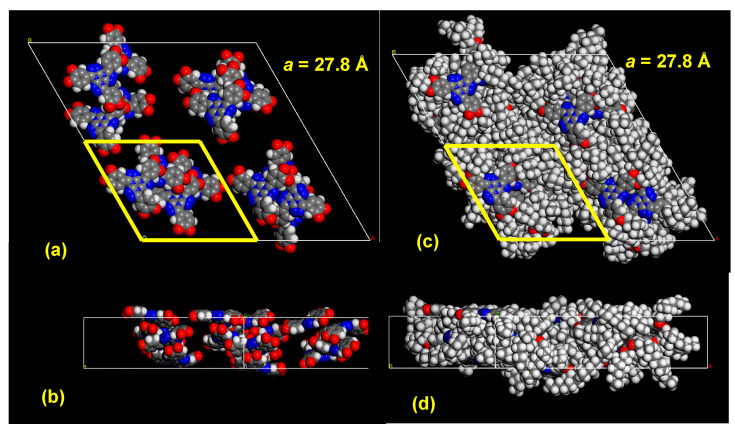
Modeled liquid crystal (LC) phase of ***t-60***: (**a**,**b**): top and side view on columns in the c-direction, where alkyl chains are omitted; (**c**,**d**) top and side view.

The XRD (SI) results reveal a hexagonal columnar mesophase of ***t*-60**. The structure is retained in the solid phase, but the lattice constants increase (130 °C: *a*_hex_ = 27.0 Å; 25 °C: *a*_hex_ = 27.8 Å). It should be noted that this lattice constant is larger than of the compound with the linear chain (3,4-O-decyloxy ***t*-19**: *a*_hex_ = 26.2 Å (20 °C) 26.8 Å (145 °C)), which can be attributed to the Thorpe–Ingold effect, preventing the alkyl chains from interdigitation. The experimental (1.02 g/mL) and X-ray density (0.99 g/mL) are nearly identical, and the π-π-distance in ***t*-60** is assumed to correspond to *d* = 3.4 Å (25 °C). This accounts for a single molecule per unit cell. The correlation length at 25 °C is 15–18 molecules. A simulation using Materials Studio [61] gives stacks of molecules twisted about 60°. These columns are arranged in a hexagonal superstructure with a lattice constant a = 27.8 Å. (Figure 5). The self-assembly of 16 molecules in a supercell analogous to ***t*-33** was geometry optimized (Figure 5). As a result of the packing, the valence energy increases by 370 kcal/mol while the intermolecular energy gain amounts to −1607 kcal/mol for the van der Waals and −392 kcal/mol for the electrostatic interaction. This stabilization of the derivatives with the branched chains is also achieved by an offset of the cores from the center of the column and a tendency to locate the peripheral dialkoxy phenyl ring above the triazine centers.

If the rim of the TTT carries three branched 4-ethyloctyloxy chains per phenyl unit (***t*-62**) [42], the melting point of T_m_ = 124 °C matches that of the analog with linear decyl chains (***t*-35**: Cr 126 Col_hd_ 165 I), but mesomorphism is erased. Diasteromerism appears to be of minor importance for LC properties. The phase width of the compound with chiral citronellyloxy chains [2] (***t*-59**: Cr 41 Col_h_ 167 I) is comparable to the TTT prepared from a racemic mixture of 3,7-dimethyloctyl chains (***t*-58**: Cr 51 M 182 I) (SI), but with the difference that the latter refrains from crystallization within the timescale of a DSC experiment. It appears that branching, e.g., the exchange of decyl to 3,7-dimethyloctyl has a comparable but more pronounced impact on TTTs as on triphenylenes [44,86].

**Figure 6:**
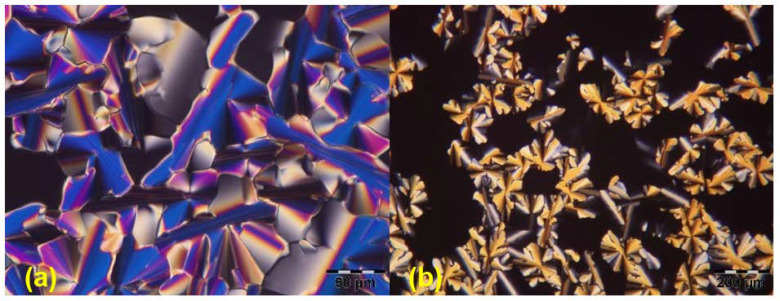
POM of ***t*-66**: (**a**): at 84 °C; (**b**): fan and homeotropic orientation at 65 °C.

Swallow-tail side chains are readily accessible via twofold malonester alkylation followed by decarboxylation and reduction. They offer a successful strategy to greatly enhance the solubility of large-core DLCs and generate mesomorphism in these materials [63,64,65,66,67,68,69,70,71]; their effect on thermal properties is expected to correlate with that of twofold alkylation with linear chains of similar length. Several *p*-alkoxyphenyl-*t*-TTTs (***t*-64**–***t*-71**) with swallow-tails are investigated. The typical “2,2-dialkylethyl” with alkyl equals hexyl to dodecyl are complimented by swallow-tails with additional branching in the alkyl unit and by tails with the systematic variation of the branching position (SI). *para*-Substitution with 2,2-dihexylethyl (2-hexyloct-1-yl) results in a mesomorphous material ***t*-64** with T_c_ = 65 °C. DSC reveals only a glass transition at 14 °C. The higher homolog ***t*-65** (2,2-dioctylethyl) displays a room-temperature LC phase with T_m_ = −15 °C (ΔH = 2.1 kJ/mol) and T_c_ = 101 °C (ΔH = 2.1 kJ/mol). The low-melting enthalpy of a pristine sample indicates incomplete crystallization; in subsequent experiments, no crystallization or melting was observed. The width of the LC phase (116 K) is nearly as large as 3,4-dinonyloxy- and 3,4-didecyloxy-TTTs ***t*-18**, ***t*-19** with a similar number of atoms in the chains (ΔT ca. 120 K), but the transitions appear at significantly lower temperatures (ΔT ca. −100 K). Further extension of the chains (***t*-66**: *p*-2,2-didecylethyl, ***t*-67**: *p*-2,2-didodecylethyl) also gives room-temperature DLCs with lower T_c_ (85 °C, 82 °C) and even lower T_m_ (−70 °C, −54 °C) (SI). The structure of these phases is columnar hexagonal (XRD), and POM textures strongly depend on the cooling rate and film thickness (Figure 6).

**Figure 7:**
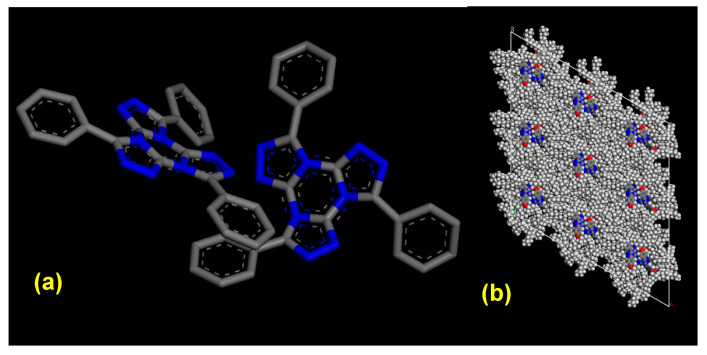
(**a**) Simulated structure of ***t*-66**: aromatic part of dimer; (**b**) view on 3 × 3 × 6 unit cells.

The mesophase of ***t*-66** has a hexagonal columnar structure with *a*_hex_ = 30.78 Å. Slightly decreasing with increasing temperature, the π-π distance is 3.58 Å. This and the aberration of X-ray density (0.79 g/mL) from the measured density (0.97 g/mL) enforced a new model for the structure. For comparison, the lattice constants of the similar 3,4-didecyloxy ***t*-19** (*a*_hex_ = 26.2 Å) and didodecyloxy ***t*-21** (*a*_hex_ = 30.4 Å) are significantly lower. The X-ray diffractogram of ***t*-66** is also in accordance with a hexagonal lattice with *a* = 30.78 Å and *c* = 5.85 Å. These parameters result in a density of 0.97 Å. A simulation [24] leads to two contorted molecules per unit cell, allowing a π-π distance of 3.58 Å and a_hex_ = 30.8. Å. This model and the X-ray reflections give a correlation length of <20 Å (Figure 7).

The series of related *p*-swallowtail *t*-TTTs with 1-hexylheptyloxy (***t*-68**: Cr 8 I), 2-hexyloctyloxy (***t*-64**: T_g_ 14 M 65 I) and 3-hexylnonyloxy (***t*-61**: Cr −71 I) shows the strong impact of the branching position on the thermal properties. Whereas the LC behavior of these low-melting compounds is severely restricted, 3,4-disubstituted *t*-TTTs ***t*-16** and ***t*-17** of similar mass are LCs with broad mesophases above 100 °C. Additional branching in the swallow-tail destabilizes the LC phase; the *p*-(diisoamyl)ethyloxy ***t*-70** TTT is nonmesomorphous (SI). Although the diastereomeric *p*-2,2-di(2-ethylhexyl)ethyloxy derivative ***t*-71** is a LC with Cr 95 M 140 I, its isomer ***t*-65** without additional branching, 2-octyldecyloxy, is characterized by Cr −15 M 101 I (SI). Likewise, ***t*-72** with two branched chains (3,4-di(2-hexyloctyl)) melts at 86 °C without any sign of mesomorphism, whereas the isomer ***t*-23** with two linear tetradecyl chains is a LC with a phase width of 192 K. Excessive branching reduces the conformational freedom in the aliphatic chains.

Discotic liquid crystals with alkoxy chains of different lengths appear only scarcely [87,88,89,90,91,92,93]. All compounds above are TTTs with three to nine peripheral chains of identical length in each case, linear or branched. Three series of TTTs with different chains on the phenyl substituent were prepared: compounds with a 3,4-3,5- and 3,4,5-di/tri-alkoxy pattern (SI). For example, 3,5-dialkoxy-substituted DLCs with either 3-butoxy-5-hexadecyloxy (***t*-73**: 47(T_g_) M 91(2.1) I) periphery or 3-hexyloxy-5-tetradecyloxy substitution (***t*-74**: Cr 59 Col_(hex?)_ 123 (4.5) I) are isomers of ***t*-29** (3,5-didecyloxy, Cr 73 M 147 I). The reduced symmetry has a strong impact on the thermal properties; the more unbalanced the periphery, the lower appear the transitions (T_m_, T_c_). Since the melting point is not as decreased as the clearing point, the width of the mesophase shrinks with the imbalanced chain length.

Accordingly, a 3-hexadexyloxy-4-methoxy periphery (***t*-75**: Cr 98 I) is not sufficient for mesomorphism, but 3-tetradecyloxy-4-hexyloxy gives a broad mesophase (***t*-76**: Cr103 M 183 I). It is interesting that the melting point corresponds to the m.p. of the dihexyloxy derivative (***t*-15**: T_m_ = 98 °C), whereas the clearing point is close to the ditetradecyloxy homolog (***t*-23**: T_c_ = 181 °C). A comparison of the LC phases of these “unsymmetrical” TTTs (***t***-**73**, ***t*-74**, ***t*-76**) with the thermal characteristics of the 3,4-didecyloxy isomer (***t*-19**: Cr 89 Col_hd_ 207 I) reveals that reduction of parity reduces liquid crystallinity. This is further substantiated by the following example: when changing the notoriously mesomorphic TTTs with a 3,4-disubstituted periphery from 3,4-didecyloxy ***t*-19** to 3-decyloxy-4-decyloxycarbonyl ***t*-77**, the LC character vanishes (SI). Thermal transitions of ***t*-78** with a 3,4-dihexyloxy-5-dodecyloxy periphery (Cr 127 M 158 I) appear nearly at the same temperatures as of ***t*-36** with three dodecyloxy chains (Cr 128 Col_hd_ 153 I); here, like with ***t*-76**, the longest chain accounts for the clearing point. With the combination of lower symmetry and branching, ***t*-79** (3,4-dihexyloxy-5-(4-ethyl)octyloxy) is rewarded by a largely reduced melting point, whereas the width of the LC phase is maintained (Cr 80 Col_h_ 136 I, ΔT = 56 K; ***t*-34**: ΔT = 58 K).

**Scheme 2:**
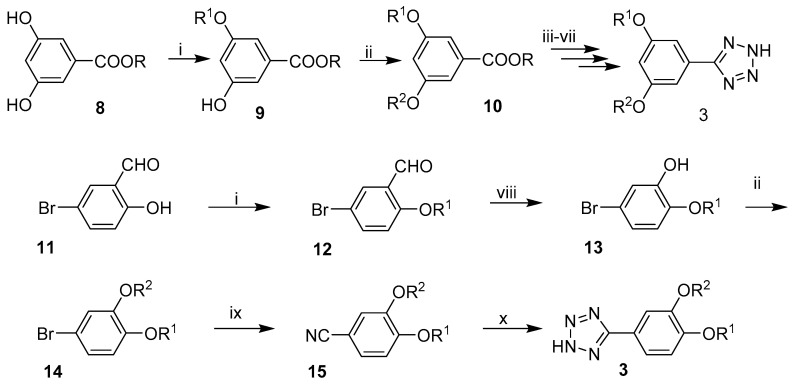
Synthesis of TTTs with chains of different lengths. i: R^1^Br, K_2_CO_3_; ii: R^2^Br, K_2_CO_3_; iii: NaOH, iv: HCl; v: SOCl_2_; vi: NH_3_ aq; vii: ClSi(N_3_)_3_; viii: mCPBA; ix: CuCN; x: Et_3_NHN_3._

The synthesis of “nonsymmetrical” 3,5-dialkoxy-TTTs is straight forward (Scheme 2): alkylation of methyl 3,5-dihydroxybenzoate **8** with a bromoalkane, the separation of di- and monoalkylated ester **9**, followed by the reaction of the latter with a second bromoalkane and conversion of the ester **10** via amide to the tetrazole **3** (SI). Similarly, partial alkylation of ethyl gallate with 1-bromohexane gives ethyl 3,4-dihexyloxy-5-hydroxybenzoate. Alkylation with a suitable reagent followed by the transformation of the ester to tetrazole and reaction with cyanuric chloride yields TTTs with a “nonsymmetrical” 3,4,5-trialkoxy periphery. The 3-alkoxy-4-alkoxy’ disubstitution requires more steps. The alkylation of 5-bromosalicylic aldehyde **11** with the first bromoalkane, Dakin reaction to yield bromoalkoxyphenol **13** and alkylation with the second bromoalkane, followed by the Rosenmund–von Braun reaction with CuCN to yield nitrile **15** and the addition of azide lead to tetrazole **3** SI.

### 2.5. Thermal Properties of Alkoxyphenyl-Substituted TTTs with Radial Structure

Prolonged heating at temperatures above 150 °C provokes rearrangement of the tristriazolotriazine. In three steps, the annulation geometry changes from the original [3,4]-annulation of triazoles to the central triazine ring to [1,5]-annulation. This brings “tangentially” oriented alkoxyaryl substituents into “radial” positions. The flattened structure of *r*-TTTs facilitates crystallization and concomitantly enhances the melting point [38,58]. The ‘radial’ isomers of t-TTTs and their thermotropic characteristics are collected in Table 4.

In addition, 3,4- and even 3,4,5-dialkoxy substitution can give mesogens [42]. Unlike the *t*-TTT series, 3,4-didecyloxy (***r*-19**: Cr 136 I) and didodecyloxy (***r*-21**: Cr_1_ 90 Cr_2_ 131 I)-substituted *r*-TTT are crystalline. Mesomorphism starts with 13 carbons per chain (***r*-22**: Cr 94 (16.8) M 119 (9.9) I) and those with 14 (***r*-23**: Cr 128 Col 136 I) or 16 carbons per chain (***r*-24**: Cr 36 M_1_ 97 M_2_ 125 Col_h_ 132 I) are also DLCs. Similar to the 3,4--didodecyloxy-TTT ***r*-21**, its 3,5-isomer ***r*-30** is not mesomorphous, but nothing more than hexyl is required for mesomorphism in the 3,4,5-trisubstituted series, (***r*-33**: Cr 13 Col_h_ 180), and the higher octyl (***r*-34**: Cr 35 Col 205 I) and decyl (***r*-35**: Cr 121Col_h_ 181 I) members display very broad mesophases without recrystallization in DSC experiments. The hexagonal mesophases (XRD) are built from columns with a helical arrangement of the disks. A pitch of 51 Å, corresponding to 14 molecules, was found for ***r*-34**. The propensity of 3,4,5-trialkoxyphenyl-substituted cores to form broad mesophases is well-known [9] but distinctively different from the tangential isomers. Branching in the periphery (***r*-62**: 3× 4-ethyloctyloxy) gives a DLC with a lower melting point and much broader mesophase (Cr 75 Col_h_ 207 I) than that found for the isomer ***r*-35** with linear decyl chains (Cr 126 Col_h_ 165 I). Though swallow-tails in the *para*-position are a successful way to *t*-TTTs with broad mesophases at low temperature, this concept fails for the *r*-isomers. 2-Decyldodec-1-yl results in crystalline ***r*-66** with T_m_ = 6 °C. The low enthalpy (ΔH = 0.7 kJ/mol) is attributed to incomplete crystallization. The swallow-tail drastically reduces the melting point, and the related ***r*-19** with two vicinal decyloxy chains melt about 130 °C higher. Swallow-tail 2-octyldecyloxy in the *m*-position on *t*-TTT ***t*-63** effectively inhibited crystallization (T_m_ = −64 °C), but the *r*-isomer ***r*-63** melts only at 75 °C without any sign of mesomorphism. Additional branching (2-(2-ethylhexyl)-4-ethyloctyloxy) stiffens the flexible parts, thus increasing the melting point of ***r*-71** (Cr 136 I), identical to the didecyloxy analog ***r*-19**. Reducing the symmetry, 3-butoxy-5-hexadecyloxy ***t*-73**, does not provoke mesomorphism (Cr 99 I), but the slightly more balanced 3-hexyloxy-5-tetradecyloxy isomer ***t*-74** is liquid crystalline even at room temperature (Cr 13 M 70 I).

**Figure 8:**
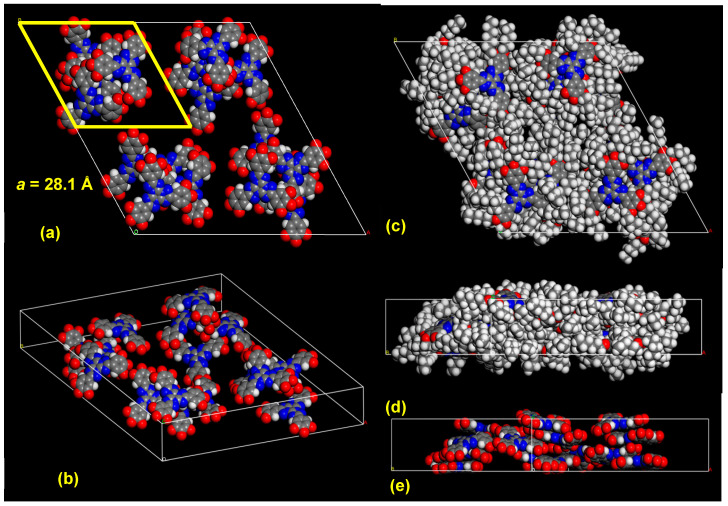
Model of a supercell (2*a* × 2*a* × 4*c*) of ***r*-33**. (**a**,**b**) Top view, where hexyl chains are omitted; (**c**,**d**) top and side view, including hexyl chains; (**e**) side view, where hexyl chains are omitted.

The structure of TTT ***r*-33** with a 3,4,5-trihexyloxyphenyl substitution was studied via X-ray diffraction and in silico [61]. ***r*-33** is a DLC with a broad mesophase (ΔT = 167 K) and hexagonal columnar structure. The experimental cell parameters are *a*_hex_ = 28.1 Å and *c* = 3.49 Å, and the density is 0.92 g/cm^3^. The model of an ensemble of 16 TTTs ***r*-33** in a supercell is depicted in Figure 8 [61] As a result, the self-assembly is stabilized by large van der Waals (−1467 kcal/mol) and electrostatic energy (−547 kcal/mol), whereas the packing results in a slight increase in valence energy (197 kcal/mol) owing to the geometric adaption of the molecular scaffold. Again, the mesogen cores are offset from the center of the columns and trialkoxy phenyl rings are preferentially located over the triazine cores.

A comparison of the 3,4,5-trihexyloxyphenyl-substituted DLCs ***r*-33** and ***t*-33** reveals a cell parameter *a* for the *r*-isomer being 2.5 Å higher than for ***t*-33**. This can be attributed not only to the more compact packing of ***t*-33** versus ***r*-33** but also to the radial topology, which generates space between the arms of the star-shaped mesogen, which is not as easily filled compared with the ***t*-33** derivative. As a result, the disorder in ***r*-33** is higher, which accounts for the much lower melting point of ***r*-33** (13 °C) compared to ***t*-33** (116 °C).

## 3. Conclusions

An extensive study of mesomorphous TTTs with di- or trialkoxyphenyl substitution reveals that the 3,4-dialkoxy pattern is nearly a guarantee for broad mesophases, whereas no 2-alkoxy chain is tolerated. 3,5-Di- and 3,4,5-trialkoxy derivatives with comparable numbers of methylene groups show similar phase widths, and the 3,5-pattern gives lower melting points. The main effect of increasing chain lengths is the reduction of clearing points. A similar effect provokes short-chain branching on the 3,4-pattern, but this is disastrous on the 3,4,5-derivatives. One swallow-tail chain per phenyl ring of *t*-TTTs largely reduces the melting point. If this group is in the *para*-position, broad mesophases are obtained. On the other hand, no *r*-TTT with a swallow-tail chain is a DLC. TTTs with chains of identical lengths are LCs with broader mesophases at higher temperatures compared to their isomers with chains of different lengths. Moderate irregularity such as short-chain branching and varying chain lengths is an effective tool to tune thermal properties. The longest chains appear to control the clearing point. This study shows that the thermal properties of TTTs are highly dependent on the substitution pattern, with individual consequences on *t*- and *r*-TTTs; however, within some homologous series, property changes seem to be predictable.

## Figures and Tables

**Table 1:** Mono-, di- and tri-1-*n*-alkoxy *t*-TTTs and their thermal characteristics.

Entry	Substitution Pattern	Transition/°C (Enthalpy/kJ/mol)	Ref
***t*-1**	R^i^ = H	Mp. 360 °C	[56]
***t*-2**	R^4^ = OC_3_H_7_	Mp. > 275	[42]
***t*-3**	R^4^ = OC_6_H_13_	Cr 129.9 I; T_g_ = 55.4	[37]
***t*-4**	R^4^ = OC_8_H1_7_	Cr 93.8 I; T_g_ = 46.7	SI
***t*-5**	R^4^ = OC_10_H_21_	Cr 76 I	SI
***t*-6**	R^4^ = OC_12_H_25_	Cr 86.8 I; T_g_ = 36.7	SI
***t*-7**	R^4^ = OC_13_H_27_	Cr 78 I	SI
***t*-8**	R^3^ = OC_10_H_21_	Cr 134 I	SI
***t*-9**	R^2^ = OC_10_H_21_	T_g_ = −64 I	SI
***t*-10**	R^2^ = R^3^ = OC_10_H_21_	Cr 69 I	SI
***t*-11**	R^2^ = R^4^ = OC_10_H_21_	Cr 50 I	SI
***t*-12**	R^2^ = R^5^ = OC_10_H_21_	Cr 89 I	SI
***t*-13**	R^3^ = R^4^ = OC_4_H_9_	Cr 131 I	SI
***t*-14**	R^3^ = R^4^ = OC_5_H_11_	Cr 102 M 201 I	SI
***t*-15**	R^3^ = R^4^ = OC_6_H_13_	Cr 98 (21.0) M 232 (3.6) I	SI
***t*-16**	R^3^ = R^4^ = OC_7_H_15_	Cr 100 (22.7) M225 (4.7) I	SI
***t*-17**	R^3^ = R^4^ = OC_8_H_17_	Cr 100 (19.9) M 226 (4.5) I	[38]
***t*-18**	R^3^ = R^4^ = OC_9_H_19_	Cr 94 (19.8) M 215 (5.7) I	SI
***t*-19**	R^3^ = R^4^ = OC_10_H_21_	Cr 89 (14.3) Col_hd_ 207 (6.7) I	[42]
***t*-20**	R^3^ = R^4^ = OC_11_H_23_	Cr 81 (5.7) M 200 (5.2) I	SI
***t*-21**	R^3^ = R^4^ = OC_12_H_25_	Cr 92.2 (19.3) Col_h_ 207.6 (6.8) I	[42]
***t*-22**	R^3^ = R^4^ = OC_13_H_27_	Cr 74 (12.4) M 175 (6.1) I	SI
***t*-23**	R^3^ = R^4^ = OC_14_H_29_	Cr-11(46.3) M_2_ 68 (9.4) M_1_ 181 (5.4) I	[42]
***t*-24**	R^3^ = R^4^ = OC_16_H_33_	Cr 19 (58.7) M_2_ 68 (9.4) M_1_ 171 (5.7) I	[42]
***t*-25**	R^3^ = R^4^ = OC_18_H_37_	Cr 40 (96.9) M_2_ 74 (9.1) M_1_ 164 (7.5) I	SI
***t*-26**	R^3^ = R^5^ = OC_6_H_13_	Cr 136 M 187 I	[60]
***t*-27**	R^3^ = R^5^ = OC_7_H_15_	Cr 122 Col_h_ 179 I	[60]
***t*-28**	R^3^ = R^5^ = OC_8_H_17_	Cr 92 M 152 I	[60]
***t*-29**	R^3^ = R^5^ = OC_10_H_21_	Cr 73 M 147 I	[60]
***t*-30**	R^3^ = R^5^ = OC_12_H_25_	Cr 74 (6.8) Col_h_ 143 (5.8) I	[60]
***t*-31**	R^3^ = R^5^ = OC_12_H_25_, R^4^ = Br	Cr 58 (9.5) M 120 I	SI
***t*-32**	R^3^ = R^5^ = OC_16_H_33_	Cr 70 (7.4) M 115 (6.0) I	SI
***t*-33**	R^3^ = R^4^ = R^5^ = OC_6_H_13_	Cr 116 (19.9) Col_hd_ 184 (4.6) I	[37]
***t*-34**	R^3^ = R^4^ = R^5^ = OC_8_H_17_	Cr 126 (22.8) Col_hd_ 184 (5.6) I	[42]
***t*-35**	R^3^ = R^4^ = R^5^ = OC_10_H_21_	Cr 126 (18.6) Col_hd_ 165 (3.7) I	[42]
***t*-36**	R^3^ = R^4^ = R^5^ = OC_12_H_25_	Cr 128 (22.7) Col_hd_ 153 (6.9) I	SI
***t*-37**	R^3^ = R^4^ = R^5^ = OC_16_H_33_	Cr 124 (24.6) M 144 (9.4) I	SI

M, M_1_ and M_2_ refer to liquid-crystalline mesophases of unknown structure, Tg: glass transition temperature.

**Table 2:** *t*-TTTs with extended p-systems and data from thermal analysis.

Entry	Substitution Pattern	Transition/°C (Enthalpy/kJ/mol)	Ref
***t*-38**	6-OC_10_H_21_-Nap [a]	Cr 121 I	SI
***t*** **-39**	5,6-OC_8_H_17_-Nap [a]	Cr 112 (1.3) Col_h_ 197(2.6) I	[38]
***t*** **-40**	5,6-OC_10_H_21_-Nap [a]	Cr 76 (7.6) M 210(2.3) I	SI
***t*** **-41**	5,6-OC_12_H_25_-Nap [a]	Cr 59 (1.5) Col_h_ 189(2.9) I	SI
***t*** **-42**	3´,4´-OC_8_H_17_-Bip [a]	Cr 148 I	SI
***t*** **-43**	3´,4´-OC_10_H_21_-Bip [a]	Cr 172 I	[38]
***t*** **-44**	3´,4´-OC_12_H_25_-Bip [a]	Cr 89 (31.1) M 199 (2.6) I	SI
***t*** **-45**	2’,5’-(OC_12_H_25_)_2_-Bip [a]	Cr 87 I	SI
***t*** **-46**	4’-(H_13_C_6_O)_2_-St [a]	mp > 240	[37]
***t*** **-47**	3’,5’-(H_13_C_6_O)_2_-St [a]	m.p. 124, Tg 13	[37]
***t*** **-48**	3’,4’,5’-(H_13_C_6_O)_2_-St [a]	Cr 175 M 200 I; Tg 178	[37]
***t*** **-49**	4’-Hexo-PEP [a]	Cr 245 I	[4]
***t*** **-50**	3,4,5-(H_17_C_8_O)_3_-PEP [a]	Cr 82 I	[4]
***t*** **-51**	3,4,5-(H_25_C_12_O)_3_-PEP [a]	Cr 80 I	SI
***t*** **-52**	3,4,5-(H_17_C_8_O)_3_-PEPEP [a]	Cr 159 M 204 I	[4]

[a] phenyl replaced by other aromatic unit; Nap = naphth-2-yl; Bip = biphenyl-4-yl; St = *E*-stilben-4-yl; PEPEP = 4-(4-phenylethynyl)-phenylethynyl)phenyl; Tg: glass transition temperature.

**Table 3:** *t*-TTTs with branched chains, swallow-tails and mixed-chain lengths, and data from thermal analysis.

Entry	Substitution Pattern	Transition/°C (Enthalpy/kJ/mol)	Ref
***t*-53**	R^4^ = O(CH_2_)_2_CH(CH_3_)(CH_2_)_3_CH(CH_3_)_2_	I	SI
***t*-54**	R^4^ = neomenthyloxy	Cr 185 I	[41]
***t*-55**	R^3^ = R^4^ = O-CH(C_2_H_5_)C_5_H_11_	Col_h_ 158 I	[1]
***t*-56**	R^3^ = R^4^ = O-CH_2_CH(C_2_H_5_)C_4_H_9_	Cr −9 M 157 I	SI
***t*-57**	R^3^ = R^4^ = O-(CH_2_)_3_CH(C_2_H_5_)C_4_H_9_	Cr72 (15.8) M 182 (5.7)	SI
***t*-58**	R^3^ = R^4^ = O(CH_2_)_2_CH(CH_3_) (CH_2_)_3_CH(CH_3_)_2_	Cr (51) M 182 (5.8) I	SI
***t*-59**	R^3^ = R^4^ = (*R*)-O(CH_2_)_2_CH(CH_3_)- (CH_2_)_2_CH=C(CH_3_)_2_	Cr 41 Col_h_ 167 I	[1]
***t*-60**	R^3^ = R^4^ = O(CH_2_)_2_C(CH_3_)_2_ C_5_H_11_	Cr 56 (17.4) Col_h_ 154 (4.6) I (T_g_ 8)	SI
***t*-61**	R^3^ = R^4^ = R^5^ = O-CH_2_-CH(C_2_H_5_)C_4_H_9_	Cr 160 I [a]	SI
***t*-62**	R^3^ = R^4^ = R^5^ = O-(CH_2_)_3_CH(C_2_H_5_)C_4_H_9_	Cr 124 I	[42]
***t*-63**	R^3^ = OCH_2_CH(C_8_H_17_)_2_	−64 (T_g_) I	SI
***t*-64**	R^4^ = OCH_2_CH(C_6_H_13_)_2_	14 (T_g_) Col_h_ 65 I	SI
***t*-65**	R^4^ = OCH_2_CH(C_8_H_17_)_2_	Cr −15 (2.1) M 101 (2.1) I	SI
***t*-66**	R^4^ = OCH_2_CH(C_10_H_21_)_2_	Cr −70 Tg 9 Tg Col_h_ 85 (2,0) I	SI
***t*-67**	R^4^ = OCH_2_CH(C_12_H_25_)_2_	Cr −54 (19.5) Col_h_ 82 (2.4) I	SI
***t*-68**	R^4^ = OCH(C_6_H_13_)	Cr 8 (1.6) I	SI
***t*-69**	R^4^ = O(CH_2_)_2_CH(C_6_H_13_)	Cr −71 I	SI
***t*-70**	R^4^ = OCH_2_CH((CH_2_)_2_CH(CH_3_)_2_)_2_	T_g_ = 42 T_m_ = 47	SI
***t*-71**	R^4^ = O-CH_2_CH(CH_2_CH(C_2_H_5_)C_4_H_9_)_2_	Cr 95 (7.1) M 140 I [b] POM: 80–120	SI
***t*-72**	R^3^ = R^4^ = OCH_2_CH(C_6_H_13_)_2_	Cr 86 I	SI
***t*-73**	R^3^ = OC_4_H_9_, R^5^ = OC_16_H_33_	47 (T_g_) M 91 (2.1) I	SI
***t*-74**	R^3^ = OC_6_H_13_, R^5^ = OC_14_H_29_	Cr 59 M 132 (4.5) I	SI
***t*-75**	R^3^ = OC_16_H_33_, R^4^ = OCH_3_	Cr 98 I	SI
***t*-76**	R^3^ = OC_14_H_29_, R^4^ = OC_6_H_13_	Cr 103 (23.0) M 183 (4.0) I	SI
***t*-77**	R^3^ = COOC_10_H_21_, R^4^ = OC_10_H_21_	Cr_1_ 61 (6.0) Cr_2_ 89 (5.3) I	SI
***t*-78**	R^3^ = R^4^ = OC_6_H_13_, R^5^ = OC_12_H_25_	Cr 127 (21.4) M 158 (4.9) I	SI
***t*-79**	R^3^ = R^4^ = OC_6_H_13_, R^5^ = O-(CH_2_)_3_CH(C_2_H_5_)C_4_H_9_	Cr 80 (23.8) Col_h_ 136 (1.5) I	SI

[a] determined by POM; Tg: glass transition temperature; [b] values from 1, heating scan.

**Table 4:** Substitution pattern of *r*-TTTs and thermal properties.

Entry	Substitution Pattern	Transition/°C (Enthalpy/kJ/mol)	Ref
***r*-1**	R^i^ = H	Cr > 390	[58]
***r*-2**	R^4^ = OC_3_H_7_	Cr > 350	[42]
***r*-3**	R^4^ = OC_6_H_13_	Cr 178 I	SI
***r*-5**	R^4^ = OC_10_H_21_	Cr 142 I	SI
***r*-15**	R^3^ = R^4^ = OC_6_H_13_	Cr 146 I	SI
***r*-19**	R^3^ = R^4^ = OC_10_H_21_	Cr 136 I	[42]
***r*-21**	R^3^ = R^4^ = OC_12_H_25_	Cr_1_ 90 Cr_2_ 131(25.2) I	[42]
***r*-22**	R^3^ = R^4^ = OC_13_H_27_	Cr 94 (16.8) M 119 (9.9) I	SI
***r*-23**	R^3^ = R^4^ = OC_14_H_29_	Cr 128 (25.4) Col 136 (1.6) I	[42]
***r*-24**	R^3^ = R^4^ = OC_16_H_33_	Cr 36 M_1_ 97 M_2_ 125 (23.4) Col_h_ 132 (1.4) I	[42]
***r*-30**	R^3^ = R^5^ = OC_12_H_25_	Cr 127 I	SI
***r*-33**	R^3^ = R^4^ = R^5^ = OC_6_H_13_	Cr 13 (8.9) Col_h_180 (3.0) I	SI
***r*-34**	R^3^ = R^4^ = R^5^ = OC_8_H_17_	Cr 35 (16.9) [a] Col 205 (2.7) I	SI
***r*-35**	R^3^ = R^4^ = R^5^ = OC_10_H_21_	Cr 121 Col_h_ 181 (3.0) I [a]	SI
***r-61***	R^3^ = R^4^ = R^5^ = O-CH_2_CH(C_2_H_5_)C_4_H_9_	Cr 241 I [b]	SI
***r*-62**	R^3^ = R^4^ = R^5^ = O-(CH_2_)_3_CH(C_2_H_5_)C_4_H_9_	Cr 75 (2.8) Col 207(4.8) I	[42]
***r*-63**	R^3^ = OCH_2_CH(C_8_H_17_)_2_	Cr 75 I	SI
***r*-66**	R^4^ = OCH_2_CH(C_10_H_21_)_2_	Cr 6 (0.7) I	SI
***r*-71**	R^4^ = O-CH_2_CH(CH_2_CH(C_2_H_5_)C_4_H_9_)_2_	Cr 136 I	SI
***r*-73**	R^3^ = OC_4_H_9_, R^5^ = OC_16_H_33_	Cr 99 (6.5) I	SI
***r*-74**	R^3^ = OC_6_H_13_, R^5^ = OC_14_H_29_	Cr 13 (4.4) M 70 (3.4) I	SI
***r*-80**	R^3^ = Cl, R^4^ = OC_6_H_13_	Cr 178 I	SI

[a] values from 1, heating scan; [b] determined by POM; Tg: glass transition temperature.

## Supplementary Materials

The following are available online, Experimental procedures, spectroscopical and analytical data, NMR spectra, DSC and POM.
