# Supplementary material for: Impact of Substitution Pattern and Chain Length on the Thermotropic Properties of Alkoxy-Substituted Triphenyl-Tristriazolotriazines
Source: Molecules. 2020 Dec 7;25(23):5761. doi: 10.3390/molecules25235761 (PMC7729490; doi:10.3390/molecules25235761)
Supplement: Supplementary file 1 [file molecules-25-05761-s001.pdf]

# Supplementary material to:

# Impact of substitution pattern and chain length on the thermotropic properties of alkoxy-substituted triphenyl-tristriazolotriazines

Thorsten Rieth, [a] Natalie Tober, [a] Daniel Limbach, [a] Tobias Haspel, [a] Marcel Sperner, [a] Niklas Schupp, [a] Philipp Wicker, [a] Stefan Glang, [a] Matthias Lehmann,[b*] and Heiner Detert[a*]

Department for Chemistry, Johannes Gutenberg-University Mainz, Duesbergweg, 10 – 14, D-55099 Mainz, Germany, Fax: +49-(0)6131-39-25338, E-mail: detert@uni-mainz.de

Julius-Maximilians-Universität, Institut für Organische Chemie, Am Hubland, 97074 Würzburg

7 General Information:
8 General procedures
8 GP1 Alkylation of cyanophenols and protocatechunitrile
8 GP2 Alkylation of benzoic esters
8 GP3 Hydrolysis of benzoic esters
8 GP4 Chlorination of benzoic acids and ammonolysis
8 GP5 Addition of azide to nitriles
8 GP6 Reaction of benzamides with triazidochlorosilane
9 GP7 Formation of tristriazolotriazines
9 1*H*-5-(4-Propoxyphenyl)tetrazole
9 3,7,11-Tris(4-propyloxyphenyl)tris([1,2,4]triazolo)[4,3-a:4',3'-c:4",3"-e][1,3,5]triazine ***t*-2**
11 2,6,10-Tris(4-propyloxyphenyl)tris([1,2,4]triazolo)[1,5-a:1',5'-c:1",5"-e][1,3,5]triazine ***r*-2**
11 5-(4-Hexyloxyphenyl)-*2H*-tetrazole
11 3,7,11-Tris(4-hexyloxyphenyl)tris([1,2,4]triazolo)[4,3-a:4',3'-c:4",3"-e][1,3,5]triazine ***t*-3**
11 2,6,10-Tris(4-hexyloxyphenyl)tris([1,2,4]triazolo)[1,5-a:1',5'-c:1",5"-e][1,3,5]triazine ***r*-3**
12 2,6,10-Tris(4-decyloxyphenyl)tris([1,2,4]triazolo)[1,5-a:1',5'-c:1",5"-e][1,3,5]triazine *r*-5
13 4-(Tridecyloxy)benzonitrile
13 5-(4-(Tridecyloxy)phenyl)-2*H*-tetrazole
13 3,7,11-Tris(4-tridecyloxyphenyl)tris([1,2,4]triazolo)[4,3-a:4',3'-c:4",3"-e][1,3,5]triazine ***t*-7**
14 Methyl 3-decyloxybenzoate
14 3-Decyloxybenzoic acid
14 3-Decyloxybenzamide
15 5-(3-Decyloxyphenyl)tetrazole
15 3 ,7,11-Tris(3-(decyloxy)phenyl)tris[1,2,4]triazolo)[4,3-a:4',3'-c:4",3"-e]-[1,3,5]triazine ***t*-8**
15 3,7,11-Tris(2-(decyloxy)phenyl)tris([1,2,4]triazolo)[4,3-a:4',3'-c:4",3"-e]-[1,3,5]triazine ***t*-9**
16 2,3-Di(decyloxy)benzoic acid

16 2,3-Di(decyloxy)benzamide
16 5-(2,3-Di(decyloxy)phenyl)-2*H*-tetrazole
17 3,7,11-Tris(2,3-di(decyloxy)phenyl)tris([1,2,4]triazolo)[4,3-a:4',3'-c:4'',3''-e][1,3,5]triazine ***t*-10**
17 5-(2,4-Di(decyloxy)phenyl)-2*H*-tetrazole
17 3,7,11-Tris(2,4-di(decyloxy)phenyl)tris[1,2,4]triazolo[4,3-a:4´,3´-c:4″,3″-e][1,3,5]triazine ***t*-11**
17 5-(2,5-Di(decyloxy)phenyl)-2*H*-tetrazole
12 3,7,11-Tris(2,5-di(decyloxy)phenyl)tris[1,2,4]triazolo[4,3-a:4´,3´-c:4″,3″-e][1,3,5]triazine ***t*-12**
18 3,4-Dibutyloxybenzonitrile
18 5-(3,4-Dibutoxyphenyl)tetrazole
19 3,7,11-Tris{3,4-butoxyphenyl}tris([1,2,4]triazolo)[4,3-a:4',3'-c:4'',3''-e][1,3,5]triazine ***t*-13**
20 5-(3,4-Dipentoxyphenyl)tetrazole
20 3,7,11-Tris{3,4-pentoxyphenyl}tris([1,2,4]triazolo)[4,3-a:4',3'-c:4'',3''-e][1,3,5]triazine ***t*-14**
21 2,6,10-Tris(3,4-di(hexyloxy)phenyl)tris([1,2,4]triazolo)[1,5-a:1',5'-c:1'',5''-e]-[1,3,5]triazine ***t*-15**
22 3,4Di(heptyloxy)benzonitrile
23 5-(3,4-Di(heptyloxy)phenyl)-2*H*-tetrazole
23 3,7,11-Tris(3,4-di(heptyloxy)phenyl)tris([1,2,4]triazolo)[4,3-a:4',3'-c:4'',3''e]-[1,3,5]triazine ***t*-16**
24 3,4-Di(octyloxy)benzonitrile
25 5-(3,4-Di(octyloxy)phenyl)-2*H*-tetrazole
25 3,7,11-Tris(3,4-di(octyloxy)phenyl)tris([1,2,4]triazolo)[4,3-a:4',3'-c:4'',3''-e]-[1,3,5]triazine ***t*-17**
26 3,4-Di(nonyloxy)benzonitrile
27 3,7,11-Tris(3,4-di(nonyloxy)phenyl)tris([1,2,4]triazolo)[4,3-a:4',3'-c:4'',3''-e]-[1,3,5]triazine ***t*-18**
29 3,4-Di(decyloxy)benzonitrile
29 5-(3,4-Di(decyloxy)phenyl)-2*H*-tetrazole
29 3,7,11-Tris(3,4-di(decyloxy)phenyl)tris([1,2,4]triazolo)[4,3-a:4',3'-c:4'',3''-e]-[1,3,5]triazine ***t*-19**
32 2,6,10-Tris(3,4-di(decyloxy)phenyl)tris([1,2,4]triazolo)[1,5-a:1',5'-c:1'',5''-e]-[1,3,5]triazine ***r*-19**
33 3,4-Di(undecyloxy)benzonitrile

32 5-(3,4-Di(undecyloxy)phenyl)-2*H*-tetrazole
32 3,7,11-Tris(3,4-di(undecyloxy)phenyl)tris([1,2,4]triazolo)[4,3-a:4',3'-c:4'',3''-e]-[1,3,5]triazine **_t_-20**
34 2,6,10-Tris(3,4-di(dodecyloxy)phenyl)tris([1,2,4]triazolo)[1,5-a:1',5'-c:1'',5''-e]-[1,3,5]triazine **_t_-21**
35 2,6,10-Tris(3,4-di(dodecyloxy)phenyl)tris([1,2,4]triazolo)[1,5-a:1',5'-c:1'',5''-e]-[1,3,5]triazine **_r_-21**
36 3,4-Di(tridecyloxy)benzonitrile
5-(3,4-Di(tridecyloxy)phenyl)-2*H*-tetrazole
36 3,7,11-Tris(3,4-di(tridecyloxy)phenyl)tris([1,2,4]triazolo)[4,3-a:4',3'-c:4'',3''-e]-[1,3,5]triazine **_t_-22**
38 2,6,10-Tris(3,4-di(tridecyloxy)phenyl)tris([1,2,4]triazolo)[1,5-a:1',5'-c:1'',5''-e]-[1,3,5]triazine **_r_-22**
39 3,4-Di(tetradecyloxy)benzonitrile
5-(3,4-Di(tetradecyloxy)phenyl)-2*H*-tetrazole
40 3,7,11-Tris(3,4-di(tetradecyloxy)phenyl)tris([1,2,4]triazolo)[4,3-a:4',3'-c:4'',3''-e]-[1,3,5]triazine **_t_-23**
41 2,6,10-Tris(3,4-di(tetradecyloxy)phenyl)tris([1,2,4]triazolo)[1,5-a:1',5'-c:1'',5''-e]-[1,3,5]triazine **_r_-23**
44 3,4-Di(hexadecyloxy)benzonitrile
44 5-(3,4-Di(hexadecyloxy)phenyl)-2*H*-tetrazole
44 3,7,11-Tris(3,4-di(hexadecyloxy)phenyl)tris([1,2,4]triazolo)[4,3-a:4',3'-c:4'',3''-e]-[1,3,5]triazine **_t_-24**
46 2,6,10-Tris(3,4-di(hexadecyloxy)phenyl)tris([1,2,4]triazolo)[1,5-a:1',5'-c:1'',5''-e]-[1,3,5]triazine **_r_-24**
48 3,4-Di(octadecyloxy)benzonitrile
48 5-(3,4-Di(octadecyloxy)phenyl)-2*H*-tetrazole
49 3,7,11-Tris(3,4-di(octadecyloxy)phenyl)tris([1,2,4]triazolo)[4,3-a:4',3'-c:4'',3''-e]-[1,3,5]triazine **_t_-25**
50 3,5-Dihexyloxybenzamide
51 5-(3,5-Dihexyloxyphenyl)tetrazole
51 3,7,11-Tris(3,5-dihexyloxyphenyl)tris([1,2,4]triazolo)[4,3-a:4',3'-c:4'',3''-e][1,3,5]triazine **_t_-26**
51 Tri(3,5-didecyloxyphenyl)2-tris[1,2,4]triazolo[4,3-a:4´,3´-c:4″,3″-e]-[1,3,5]triazine **_t_-29**
51 5-(3,5-Didodecyloxyphenyl)tetrazole
51 2,6,10-Tris(3,5-di(dodecyloxy)phenyl)tris([1,2,4]triazolo)[1,5-a:1',5'-c:1'',5''-e]-[1,3,5]triazine **_r_-30**
53 Methyl 4-bromo-3,5-didodecyloxybenzoate
53 4-Bromo-3,5-didodecyloxybenzoic acid
54 4-Bromo-3,5-didodecyloxybenzamide
54 5-(4-Bromo-3,5-didodecyloxyphenyl)-2*H*-tetrazole
55 3,7,11-Tris(4-bromo-3,5-bis(dodecyloxy)phenyl)tris([1,2,4]triazolo)[4,3-a:4',3'-c:4'',3''-e]-[1,3,5]triazine **_t_-31**
57 3,5-Di(hexadecyloxy)benzoic acid
58 3,5-Di(hexadecyloxy)benzoic acid amide
58 5-(3,5-Di(hexadecyloxy)phenyl)-2*H*-tetrazole
59 3,7,11-Tris(3,5-di(hexadecyloxy)phenyl)tris([1,2,4]triazolo)[4,3-a:4',3'-c:4'',3''-e][1,3,5] triazine **_t_-32**
61 3,4,5-Trihexyloxy-benzamide
61 5-(3,4,5-Trihexyloxyphenyl)-*2H*-tetrazole
61 3,7,11-Tris(3,4,5-trihexyloxyphenyl)tris([1,2,4]triazolo)[4,3-a:4',3'-c:4'',3''-e]-[1,3,5]triazine **_t_-33**
61 2,6,10-Tris-(3,4,5-trihexyloxyphenyl)tris([1,2,4]triazolo)[1,5-a:1',5'-c:1'',5''-e]-[1,3,5]triazine **_r_-33**

62 3,7,11-Tris(3,4,5-tri(octyloxy)phenyl)tris([1,2,4]triazolo)[4,3-a:4',3'-c:4'',3''-e] [1,3,5]triazine ***t*-34**

62 2,6,10-Tris(3,4,5-tri(octyloxy)phenyl)tris([1,2,4]triazolo)[1,5-a:1',5'-c:1'',5''-e][1,3,5]triazine ***r*-34**

64 3,4,5-Tridecyloxy-benzoic amide

64 5-(3,4,5-Tridecyloxy)phenyl-*2H*-tetrazole

64 Tri(3,4,5-tridecyloxy)phenyl-tris[1,2,4]triazolo[4,3-a:4´,3´-c:4″,3″-e]-[1,3,5]triazine ***t*-35**

65 2,6,10-Tris(3,4,5-tri(decyloxy)phenyl)tris([1,2,4]triazolo)[1,5-a:1',5'-c:1'',5''-e]-[1,3,5]triazine ***r*-35**

66 3,4,5-Tridodecyloxy-benzoic amide

66 5-(3,4,5-Tridodecyloxy)phenyl-*2H*-tetrazole

66 Tri(3,4,5-tri(dodecyloxy)phenyl)-tris[1,2,4]triazolo[4,3-a:4´,3´-c:4″,3″-e]-[1,3,5]triazine ***t*-36**

67 5-(3,4,5-Tri(hexadecyloxy)phenyl)-2*H*-tetrazole

68 3,7,11-Tris(3,4,5-tri(hexadecyloxy)phenyl)tris([1,2,4]triazolo)[4,3-a:4',3'-c:4'',3''-e]-[1,3,5]triazine ***t*-37**

68 6-Decyloxynaphthonitrile-2

69 3,7,11-Tris(6-(decyloxy)naphth-2-yl)tris([1,2,4]triazolo)[4,3-a:4',3'-c:4'',3''-e] [1,3,5]triazine ***t*-38**

69 3,7,11-Tris(5,6-di(octyloxy)naphth-2-yl)tris([1,2,4]triazolo)[4,3-a:4',3'-c:4'',3''-e] [1,3,5]triazine ***t*-39**

70 6-Bromo-1,2-di(decyloxy)naphthalene

70 5,6-Di(decyloxy)-2-naphthonitrile

71 5-(5,6-Di(decyloxy)naphth-2-yl)-2*H*-tetrazole

71 3,7,11-Tris(5,6-di(decyloxy)naphth-2-yl)tris([1,2,4]triazolo)[4,3-a:4',3'-c:4'',3''-e][1,3,5]triazine ***t*-40**

72 6-Bromo-1,2-di(dodecyloxy)naphthalene

72 5,6-Di(dodecyloxy)-2-naphthonitrile

72 5-(5,6-Di(dodecyloxy)naphth-2-yl)-2*H*-tetrazole

72 3,7,11-Tris(5,6-di(dodecyloxy)naphth-2-yl)tris([1,2,4]triazolo)[4,3-a:4',3'-c:4'',3''-e]-[1,3,5]triazine ***t*-41**

74 3,7,11-Tris(3',4'-di(octyloxy)-[1,1'-biphenyl]-4-yl)tris([1,2,4]triazolo)[4,3-a:4',3'-c:4'',3''-e]-[1,3,5]triazine ***t*-42**

5-(3',4'-Di(decyloxy)-[1,1'-biphenyl]-4-yl)-2*H*-tetrazole

75 3,7,11-Tris(3',4'-di(decyloxy)-[1,1'-biphenyl]-4-yl)tris([1,2,4]triazolo)[4,3-a:4',3'-c:4'',3''-e]-[1,3,5]triazine ***t*-43**

75 5-(3',4'-Di(dodecyloxy)-[1,1'-biphenyl]-4-yl)-2*H*-tetrazole

76 3,7,11-Tris(3',4'-di(dodecyloxy)-[1,1'-biphenyl]-4-yl)tris([1,2,4]triazolo)[4,3-a:4',3'-c:4'',3''-e]-[1,3,5]triazine ***t*-44**

77 4-(2,5-Di(decyloxy)phenyl)acetophenone

77 4-(2,5-Di(decyloxy)phenyl)benzoic acid

77 4-(2,5-Di(decyloxy)phenyl)benzamide

77 5-(2',5'-Di(decyloxy)-[1,1'-biphenyl]-4-yl)-*2H*-tetrazole

78 3,7,11-Tris(2',5'-di(decyloxy)-[1,1'-biphenyl]-4-yl)tris([1,2,4]triazolo)[4,3-a:4',3'-c:4'',3''e]-[1,3,5]triazine ***t*-45**

78 3,7,11-Tris(4´-hexyloxystilben-4-yl)tris([1,2,4]triazolo)[4,3-a:4',3'-c:4'',3''-e]-[1,3,5]triazine ***t*-46**

78 3,7,11-Tris(3´,5´-dihexyloxystilben-4-yl)tris([1,2,4]triazolo)[4,3-a:4',3'-c:4'',3''-e]-[1,3,5]triazine ***t*-47**

78 3,7,11-Tris(3´,4´,5´-trihexyloxystilben-4-yl)tris([1,2,4]triazolo)[4,3-a:4',3'-c:4'',3''-e]-[1,3,5]triazine ***t*-48**

78 4-((3, 7-Dimethyloctyl)oxy)benzonitrile

79 5-(4-((3, 7-Dimethyloctyl)oxy)phenyl)-2*H*-tetrazole

79 3,7,11-Tris(4-((3,7-dimethyloctyl)oxy)phenyl)tris([1,2,4]triazolo)[4,3-a:4',3'-c:4'',3''-e]-[1,3,5]triazine ***t*-53**

80 3,7,11-Tris(4-(neomenthyloxy)phenyl)tris([1,2,4]triazolo)[4,3-a:4',3'c:4'',3''-e]-[1,3,5]triazine ***t*-54**
80 3,4-Bis(2-ethylhexyloxy)benzonitrile
80 5-(3,4-Bis(2-ethylhexyloxyphenyl)tetrazole
81 3,7,11-Tris{3,4-bis(2-ethylhexoxy)phenyl}tris([1,2,4]triazolo)[4,3-a:4',3'-c:4'',3''-e]-[1,3,5]triazine ***t*-56**
83 3,4-Di((4-ethyloctyl)oxy)benzonitrile
84 5-(3,4-Di((4-ethyloctyl)oxy)phenyl)-2*H*-tetrazole
84 3,7,11-Tris(3,4-di((4-ethyloctyl)oxy)phenyl)tris([1,2,4]triazolo)[4,3-a:4',3'-c:4'',3''-e]-[1,3,5]triazine ***t*-51**
86 3,4-Di((3,7-dimethyloctyl)oxy)benzonitrile
86 5-(3,4-Di((3,7-dimethyloctyl)oxy)phenyl)-2*H*-tetrazole
87 3,7,11-Tris(3,4-di((3,7-dimethyloctyl)oxy)phenyl)tris([1,2,4]triazolo)[4,3-a:4',3'-c:4'',3''-e]-[1,3,5]triazine ***t*-58**
88 1-Bromo-3,3-dimethyloctane
89 3,4-Di(3,3-dimethyloctyloxy)benzonitrile
89 1*H*-5-(3,4-Di(3,3-dimethyloctyloxy)phenyl)tetrazole
90 3,7,11-Tris(3,4-di(3,3-dimethyloctyloxy)phenyl)tris[1,2,4]-triazolo[4,3-a:4´,3´-c:4´´,3´´-e][1,3,5]triazine ***t*-60**
90 3,4,5-Tri(2-ethylhexyloxy)benzoic amide
91 5-(3,4,5-Tri((2-ethylhexyl)oxy)phenyl)-2*H*-tetrazole
91 3,7,11-Tris(3,4,5-tri((2-ethylhexyl)oxy)phenyl)tris([1,2,4]triazolo)[4,3-a:4',3'-c:4'',3''-e]-[1,3,5]triazine ***t*-61**
91 2,6,10-Tris(3,4,5-tri((2-ethylhexyl)oxy)phenyl)tris([1,2,4]triazolo)[1,5-a:1',5'-c:1'',5''-e]-[1,3,5]triazine ***r*-61**
92 Ethyl 3,4,5-tri((4-ethyloctyl)oxy)benzoate
93 3,4,5-Tri((4-ethyloctyl)oxy)benzoic acid[9]
93 3,4,5-Tri((4-ethyloctyl)oxy)benzoic amide[9]
93 5-(3,4,5-Tri((4-ethyloctyl)oxy)phenyl)-2*H*-tetrazole[9]
93 3,7,11-Tris(3,4,5-tri((4-ethyloctyl)oxy)phenyl)tris([1,2,4]triazolo)[4,3-a:4',3'-c:4'',3''-e]-[1,3,5]triazine ***t*-62**
94 2,6,10-Tris(3,4,5-tri((4-ethyloctyl)oxy)phenyl)tris([1,2,4]triazolo)[1,5-a:1',5'-c:1'',5''-e]-[1,3,5]triazine ***r*-62**
96 Ethyl 3-(2-octyldecyloxy)benzoate
96 3-(2-Octyldecyloxy)benzoic acid
97 3-(Octyldecyloxy)benzoic acid amide
97 5-(3-(2-Octyldecyloxy)phenyl)-1*H*-tetrazole
97 3,7,11-Tris(3-(2-octyldecyloxy)phenyl)tris[1,2,4]triazolo[4,3-a:4´,3´-**c:4´´,3´´**e]-[1,3,5]-triazine ***t*-63**
98 2,6,10-Tris(3-((2-octyldecyl)oxy)phenyl)tris([1,2,4]triazolo)[1,5-a:1',5'-c:1'',5''-e][1,3,5]triazine ***r*-63**
99 4-((2-Hexyloctyl)oxy)benzonitrile
99 1*H*-5-(4-((2-Hexyloctyl)oxy)phenyl)tetrazole
99 3,7,11-Tris(4-((2-hexyloctyl)oxy)phenyl)tris([1,2,4]triazolo)[4,3-a:4',3'-c:4'',3''-e]-[1,3,5]triazine ***t*-64**
99 p-(2-Octyldecyloxy)benzonitrile
100 5-(4-(2-Octyldecyloxy)phenyl)-1H-tetrazole
100 3,7,11-Tris(4-(2-octyldecyloxy)phenyl)tris[1,2,4]triazolo[4,3-a:4´,3´-c:4´´,3´´e]-[1,3,5]-triazine ***t*-65**
100 2-Decyldodecan-1-ol
101 1-Bromo-2-decyldodecane
101 4-((2-Decyldodecyl)oxy)benzonitrile
101 1*H*-5-(4-((2-Decyldodecyl)oxy)phenyl)tetrazole

101 3,7,11-Tris(4-((2-decyldodecyl)oxy)phenyl)tris([1,2,4]triazolo)[4,3-a:4',3'-c:4'',3''-e][1,3,5]triazine ***t*-66**

102 2,6,10-Tris-(4-(2-decyldodecyloxy)phenyl)tris([1,2,4]triazolo)[1,5-a:1',5'-c:1'',5''-e]-[1,3,5]triazine ***r*-66**

103 2-Dodecyltetradecan-1-ol

103 1-Bromo-2-dodecyltetradecane

103 4-((2-Dodecyltetradecyl)oxy)benzonitrile

103 1*H*-5-(4-(2-Dodecyltetradecyloxy)phenyl)tetrazole

104 3,7,11-Tris(4-((2-dodecyltetradecyl)oxy)phenyl)tris([1,2,4]triazolo)[4,3-a:4',3'-c:4'',3''-e]-[1,3,5]triazine ***t*-67**

104 *p*-(1-Hexylheptyloxy)benzonitrile

104 1*H*-5-(4-(1-Hexylheptyloxy)phenyl)tetrazole

105 3,7,11-Tris(4-(1-hexylheptyloxy)phenyl)tris[1,2,4]-triazolo[4,3-a:4´,3´-c:4´´,3´´-e]-[1,3,5]-triazine ***t*-68**

106 2,6,10-Tris(4-(tridecan-7-yloxy)phenyl)tris([1,2,4]triazolo)[1,5-a:1',5'-c:1'',5''-e]-[1,3,5]triazine ***r*-68**

106 3-Hexylnonylbromide

106 4-(3-Hexylnonyloxy)benzonitrile

106 5-(4-(3-Hexylnonyloxy)phenyl)-1H-tetrazole

107 3,7,11-Tris(4-(3-hexylnonyloxy)phenyl)tris[1,2,4]-triazolo[4,3-a:4´,3´- c:4´´,3´´-e]-[1,3,5]triazine ***t*-69**

107 1-Bromo-2-(3-methylbutyl)-5-methylhexane

*107* *p*-(2-(3-Methylbutyl)-5-methylhexyloxy)benzonitrile

107 1*H*-5-((2-(3-Methylbutyl)-5-methylhexyloxy)phenyl)tetrazole

107 3,7,11-Tris((2-(3-methylbutyl)-5-methylhexyloxy)phenyl)tris[1,2,4]-triazolo[4,3-a:4´,3´-c:4´´,3´´-e][1,3,5]triazine ***t*-70**

108 Dimethyl 2,2-bis(2-ethylhexyl)malonate

108 Methyl 4-ethyl-2-(2-ethylhexyl)octanoate

109 4-Ethyl-2-(2-ethylhexyl)octan-1-ol

109 7-(Bromomethyl)-5,9-diethyltridecane

109 4-(4-Ethyl-2-(2-ethylhexyl)octyloxy)benzonitrile

109 1*H*-5-(4-(4-Ethyl-2-(2-ethylhexyl)octyloxy))tetrazole

110 3,7,11-Tris(4-((4-ethyl-2-(2-ethylhexyl)octyl)oxy)phenyl)tris([1,2,4]triazolo)[4,3-a:4',3'-c:4'',3''-e][1,3,5]triazine ***t*-71**

112 2,6,10-Tris(4-((4-ethyl-2-(2-ethylhexyl)octyl)oxy)phenyl)tris([1,2,4]triazolo)[1,5-a:1',5'-c:1'',5''-e][1,3,5]triazine ***r*-71**

113 3,4-Bis((2-hexyloctyl)oxy)benzonitrile

114 1*H*-5-(3,4-Bis((2-hexyloctyl)oxy)phenyl)tetrazole

114 3,7,11-Tris(3,4-bis((2-hexyloctyl)oxy)phenyl)tris([1,2,4]triazolo)[4,3-a:4',3'-c:4'',3''-e 1,3,5]triazine ***t*-72**

114 Methyl 3,5-di(hexadecyloxy)benzoate and methyl 3-(hexadecyloxy)-5-hydroxy benzoate

115 Methyl 3-(butyloxy)-5-hexadecyloxy benzoate

116 3-Butoxy-5-(hexadecyloxy)benzoic acid

116 3-Butoxy-5-(hexadecyloxy)benzoic amide

118 5-(3-Butoxy-5-(hexadecyloxy)phenyl)-2*H*-tetrazole

119 3,7,11-Tris(3-butoxy-5-(hexadecyloxy)phenyl)tris([1,2,4]triazolo)[4,3-a:4',3'-c:4'',3''-e][1,3,5]triazine ***t*-73**

120 2,6,10-Tris(3-butoxy-5-(hexadecyloxy)phenyl)tris([1,2,4]triazolo)[1,5-a:1',5'-c:1'',5''-e]-[1,3,5]triazine ***r*-73**

121 Ethyl 3-hydroxy-5-hexyloxybenzoate

122 Ethyl 3-tetradecyloxy-5-hexyloxybenzoate

122 3-Tetradecyloxy-5-hexyloxybenzoic acid

122 3-Tetradecyloxy-5-hexyloxybenzamide

123 5-(3-Tetradecyloxy-5-hexyloxyphenyl)-*2H*-tetrazole

124 3,7,11-Tris(3-(hexyloxy)-5-(tetradecyloxy)phenyl)tris([1,2,4]triazolo)[4,3-a:4',3'-c:4'',3''-e][1,3,5]triazine ***t*-66**
126 2,6,10-Tris-(3-hexyloxy-5-tetradecyloxyphenyl)tris([1,2,4]triazolo)[1,5-a:1',5'-c:1'',5''-e]-[1,3,5]triazine ***r*-66**
127 5-Bromo-2-hexyloxy-benzaldehyde
127 5-Bromo-2-hexyloxyphenol
127 5-Bromo-2-hexyloxy-1-tetradecyloxybenzene
128 4-Hexyloxy-3-tetradecyloxybenzonitrile
128 5-(4-Hexyloxy-3-tetradecyloxyphenyl)-*2H*-tetrazole
129 3,7,11-Tris(3-(hexadecyloxy)-4-methoxyphenyl)tris([1,2,4]triazolo)[4,3-a:4',3'-c:4'',3''-e]-[1,3,5]triazine ***t*-75**
129 3,7,11-Tris(4-(hexyloxy)-3-(tetradecyloxy)phenyl)tris([1,2,4]triazolo)[4,3-a:4',3'-c:4'',3''-e][1,3,5]triazine ***t*-76**
131 4-Cyanosalicylic acid
131 Decyl 4-cyano-2-(decyloxy)benzoate
132 Decyl 2-(decyloxy)-4-(1*H*-tetrazol-5-yl)benzoate
132 Tris(decyl) 4,4',4''-(tris([1,2,4]triazolo)[4,3-a:4',3'-c:4'',3''-e][1,3,5]triazine-3,7,11-triyl)-tris(2-(decyloxy)benzoate) ***t*-77**
133 3-(Dodecyloxy)-4,5-bis(hexyloxy)benzoic acid
134 3-(Dodecyloxy)-4,5-bis(hexyloxy)benzamide
134 3-(Dodecyloxy)-4,5-bis(hexyloxy) phenyl)tetrazole
134 3,7,11-Tris(3-(dodecyloxy)-4,5-bis(hexyloxy)phenyl)tris([1,2,4]triazolo)[4,3-a:4',3'-c:4'',3''-e][1,3,5]triazine **t-78**
136 Ethyl 3-(4-ethyloctyloxy)-4,5-di(hexyloxy)benzoate
136 3-(4-Ethyloctyloxy)-4,5-bis(hexyloxy)benzoic acid
136 3-(4-Ethyloctyl)-4,5-bis(hexyloxy)benzamide
137 1*H*-5-(3-(4-Ethyloctyl)-4,5-bis(hexyloxy)phenyl)tetrazole3,7,11-Tris(3-((4-ethyloctyl)-oxy)-4,5-bis(hexyloxy)phenyl)tris([1,2,4]triazolo)[4,3-a:4',3'-c:4'',3''-e][1,3,5]triazine ***t*-79**
140 XRD pattern of TTTs ***t*-10, *t*-60, *t*-64*, t*-66, *t*-67, *t*-79, *r*-33**
139 5-Chloro-3-(3-chloro-4-(hexyloxy)phenyl)-*1H*-1,2,4-triazole ***r*-80**
144 References

**General Information:**

$^1H$ and $^{13}C$ NMR spectra: Bruker AC 300 (300 MHz), Bruker AV 400 (400 MHz), and Bruker ARX 400 (400 MHz), solvents were $CDCl_3$, $C_6D_6$, DMSO-$d_6$. Chemical shifts are expressed as δ values in ppm, coupling constants are given in Hz. Assignments of $^1H$ and $^{13}C$ signals on the basis of HSQC and HMBC experiments. Abbreviations used for assignment of spectra: ph: phenyl, tri: triazole. Melting points: Büchi HWS SG 200, Stuart Scientific SMP3. DSC: Perkin Elmer, DSC 7, IR: JASCO 4100 FT-IR (ATR), FD-MS: Mat 95 (Finnigan); HR-ESI: Q-TOF-ULTIMA 3, Lock Spray device (Waters-Micromass), NaICsI as reference. UV-vis: Perkin-Elmer Lambda 16. Fluorescence: Perkin-Elmer LS 50B. Polarized microscopy: Olympus BX51, ColorView Olympus camera, heattable Linkam LTS 350 for temperature regulation. WAXS measurements were performed using a custom setup consisting of the Siemens Kristalloflex X-ray source (copper anode X-ray tube, operated at 35 kV/20 mA), Osmic confocal MaxFlux optics, Bruker detector Highstar, The samples were prepared by filament extrusion using a custom-built mini-extruder and were positioned perpendicular to the incident X-ray beam and vertical to the 2D detector.

**GP1 Alkylation of cyanophenols and protocatechunitrile**

A mixture of the hydroxybenzonitrile (22 mmol), alkyl bromide (1.1 eq per hydroxy group) and $K_2CO_3$ (1.5 eq per hydroxy group) in 100 ml degassed DMF was stirred at 80°C. The reaction progress was monitored by thin layer chromatography. After the reaction was complete, the mixture was acidified with 2N HCl (100 ml) and extracted with petroleum ether (3 x 50 ml). The combined organic phases were washed with brine and dried over $MgSO_4$. After removing the solvent under reduced pressure, the crude product was crystallized from petroleum ether (40-70°C).

**GP2: Alkylation of benzoic esters**

The respective hydroxybenzoate with 1-3 hydroxy groups was suspended in a solvent (DMF, acetonitrile or dioxane) and an excess of potassium carbonate (1.3 eq per hydroxy group) , alkyl bromide or alkyl iodide and in some cases one drop of aliquat 336 were added to the suspension. The resulting mixture was stirred under reflux and the reaction monitored by TLC. After completion, the reaction was quenched with hydrochloric acid (2N), the phases were separated and the aqueous phase extracted three times with toluene or chloroform. The combined organic phases were dried with $MgSO_4$ and the solvent was removed on the rotary evaporator. The crude product was either recrystallized or chromatographed through a column.

**GP3 Hydrolysis of benzoic ester**

The ethyl benzoate was dissolved in an alcohol and an excess of a sodium hydroxide solution (2N) was added. The resulting mixture was stirred under reflux overnight. After completion, the reaction was quenched with hydrochloric acid (2N) and the suspension was extracted three times with toluene or chloroform. The combined organic phases were dried with $MgSO_4$ and the solvent was removed on the rotary evaporator. The crude product was either recrystallized or chromatographed through a column.

**GP4: General procedure for the synthesis of benzamides from benzoic acids via chlorination and ammonolysis**

10 mmol) alkoxybenzoic acid and 4 mL $SOCl_2$ in 60 mL dry toluene with 1 drop DMF were refluxed for 12 h. The solvent was evaporated, the residue dissolved in 20 mL dioxane and this solution was added dropwise into 300ml heavily stirred, ice-cold, concentrated ammonia solution. Precipitated amide and chloroform extracts were dried, concentrated, and the residue recrystallized from toluene. Yield generally quantitative.

**GP5 Addition of azide to nitriles**

A mixture of the alkoxybenzonitrile (8.3 mmol), sodium azide (4 eq), and triethyl ammonium chloride (4 eq) in toluene (75 ml) was stirred under reflux for 20h. An additional amount of sodium azide (1 eq) and triethyl ammonium chloride (1 eq) was added and stirring under reflux was continued for 24h. The reaction progress was monitored by thin layer chromatography. After the reaction was complete 2N HCl (100 ml) was added. The toluene phase was separated and the aqueous layer was extracted with ethyl acetate (2 x 10 ml). The combined organic phases were dried over $MgSO_4$ and concentrated *in vacuo*. The resulting residue was crystallized from ethanol.

**GP6: General procedure for the synthesis of 5-(alkoxyphenyl)tetrazoles from alkoxybenzamides**

Sodium azide (0.066 mol) was suspended in anhydrous acetonitrile and $SiCl_4$ (0.022 mol) was added. The mixture was stirred for 45 min and the corresponding benzoic amide (10 mmol) was added. After stirring for 16 h, a second portion of $NaN_3$ and $SiCl_4$ was added and the mixture heated to 50°C until the starting material has been consumed (TLC). The cooled mixture was diluted with $CHCl_3$, extracted with brine (3 x 50 mL), and dried ($Na_2SO_4$). Alumina (basic, 15 g) was added to the filtered solution and the solvent was evaporated. The residue was placed on a silica gel column. Elution of by-products with toluene/ethyl acetate

(1/1) first, of the tetrazoles by adding glacial acetic acid (0.5%) to the eluent. **Attention**: Triazidochlorosilane, a probably highly dangerous compounds, is an intermediate in this procedure. Though several dozen reactions have been performed without any problems, all possible protective measures should be applied! **A very high risk of violent explosions**!

**GP7 Formation of tristriazolotriazines**
Under dry conditions the alkoxyphenyl-2*H*-tetrazole (2.5 mmol) and cyanuric chloride (0.75 mmol) were suspended in 10 ml of dry xylene, and 0.35 ml dry collidine (2.48 mmol) was added. The reaction mixture was gradually heated over 2 hours to 80°C. The reaction progress was monitored by thin layer chromatography. After the reaction was complete, 2N HCl (20 ml) was added. The xylene phase was separated and the aqueous layer was extracted with ethyl acetate (2 x 10 ml). The combined organic phases were dried over $MgSO_4$ and concentrated *in vacuo*. The crude product was purified by column chromatography (basic $Al_2O_3$, toluene/ethyl acetate 20:1).

**1*H*-5-(4-Propoxyphenyl)tetrazole**
This compound was prepared according to general procedure DL-Tet1 from 4.21 g (26.1 mmol, 1 eq) 4-propoxybenzonitrile **157**, 3.40 g (52.2 mmol, 2.0 eq) sodium azide and 7.57 g (55.0 mmol, 2.1 eq) triethylammonium chloride in 50 mL toluene. Precipitated solid was filtered off and the aqueous phase was extracted with dichloromethane. The solvent was removed by rotary evaporation and the combined solids were recrystallized from ethanol. The crystallization afforded 5.00 g (24.5 mmol, 94 %) of the product as colorless solid.
m.p.: 237-238 °C (dec.). $^{1}$H-NMR (400 MHz, $CDCl_3$) δ = 7.91 – 7.87 (m (AA'XX'), 2H), 6.91 – 6.86 (m (AA'XX'), 2H), 3.87 (t, $^{3}J$ = 6.6 Hz, 2H, O*CH₂*), 1.76 – 1.68 (m, 2H, $OCH_2CH_2$), 0.94 (t, $^{3}J$ = 7.4 Hz, 3H, $CH_3$). IR (neat): ṽ [1/cm] = 2942 w, 2544 w, 1855 w, 1612 s, 1498 s, 1469 m, 1395 m, 1294 m, 1254 s, 1178 s, 1047 m, 1013 s, 989 s, 832 ss, 766 m, 750 ss, 699 m, 654 s, 623 s.

**3,7,11-Tris(4-propyloxyphenyl)tris([1,2,4]triazolo)[4,3-a:4',3'-c:4'',3''-e][1,3,5]triazine *t*-2**
(ref 1) 500 mg (2.5 mmol) 5-(4-propoxyphenyl)-*2H*-tetrazole, 0.5 mL sym-collidine together with 125 mg (0.7 mmol) cyanuric chloride were stirred in 25 mL dry xylenes. The temperature was gradually raised to 80 °C. After 6 h, aqueous work-up and chromatography ($SiO_2$, $Al_2O_3$-head, toluene/ethyl acetate = 25/1) yielded 290 mg (0.5 mmol, 71 %) of a colorless solid.
**$^{1}$H-NMR** (400 MHz, DMSO-$d_6$): $\delta$ [ppm] = 7.94 (d, $^{3}J$ = 8.9 Hz, 2H, 2-H, 6-H, ph), 7.18 (d, $^{3}J$ = 8.9 Hz, 2H, 3-H, 5-H, ph), 4.07 (t, $^{3}J$ = 6.5 Hz, 2H, $OCH_2$), 1.91 – 1.67 (m, 2H, $CH_2$), 1.02 (t, $^{3}J$ = 7.4 Hz, 3H, $CH_3$). **$^{13}$C-NMR** (100 MHz, $CDCl_3$): $\delta$ [ppm] = 161.04 (C-4, ph), 149.08 (C-5, trl), 141.76 (C-3, trl), 131.53 (C-2, C-6, ph), 116.61 (C-1, ph), 114.36 (C-3, C-5, ph), 69.32 ($OCH_2$), 22.01 ($CH_2$), 10.44 ($CH_3$). **IR** (ATR): $\tilde{\upsilon}$ [$cm^{-1}$] = 2920 w, 2871 w, 1607 s, 1597 m, 1470 m, 1421 m, 1388 w, 1295 w, 1254 s, 1170 m, 1106 w, 1014 m, 838 m, 751 m, 714 w. **HR-ESI**: calcd. for $C_{33}H_{33}N_9O_3 + H^+$: 604.2785; found: 604.2775.

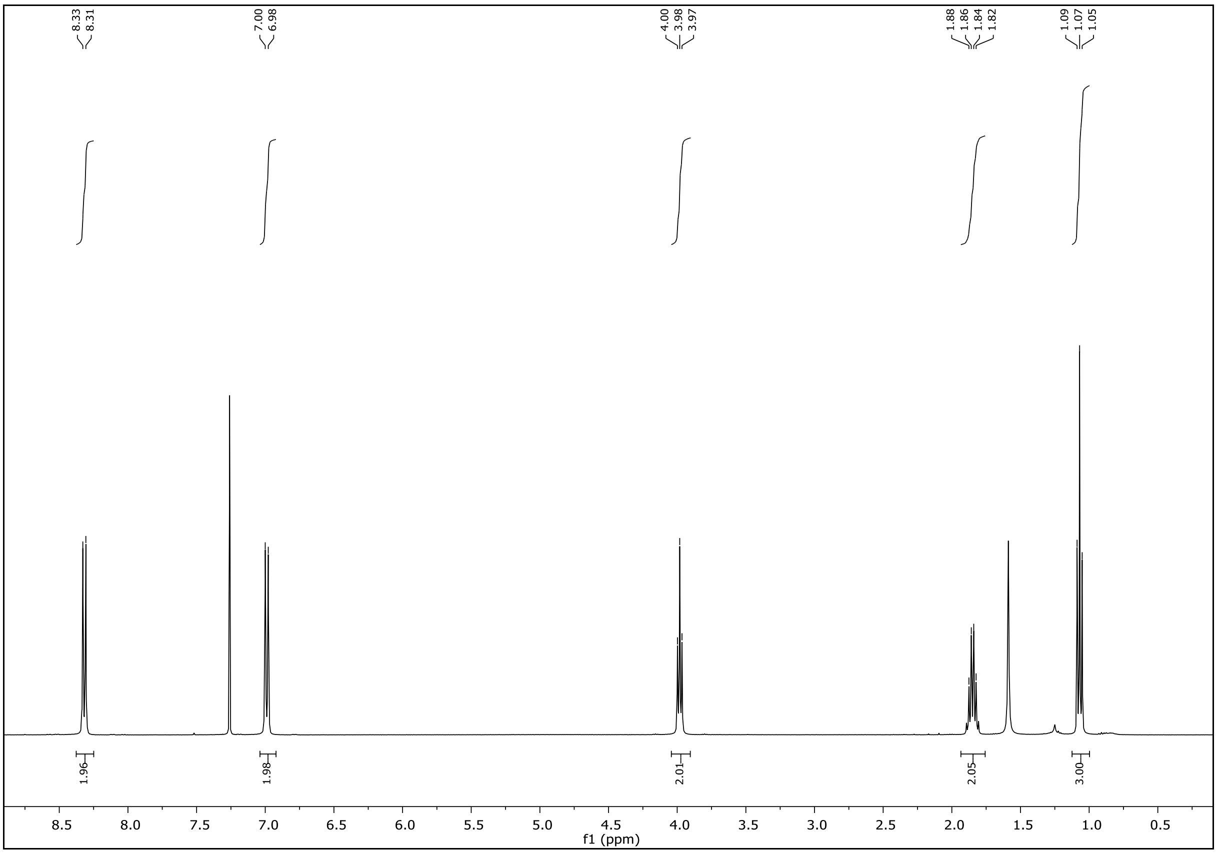
8.33
8.31
7.00
6.98
4.00
3.98
3.97
1.88
1.86
1.84
1.82
1.09
1.07
1.05
1.96
1.98
2.01
2.05
3.00
8.5 8.0 7.5 7.0 6.5 6.0 5.5 5.0 4.5 4.0 3.5 3.0 2.5 2.0 1.5 1.0 0.5
f1 (ppm)

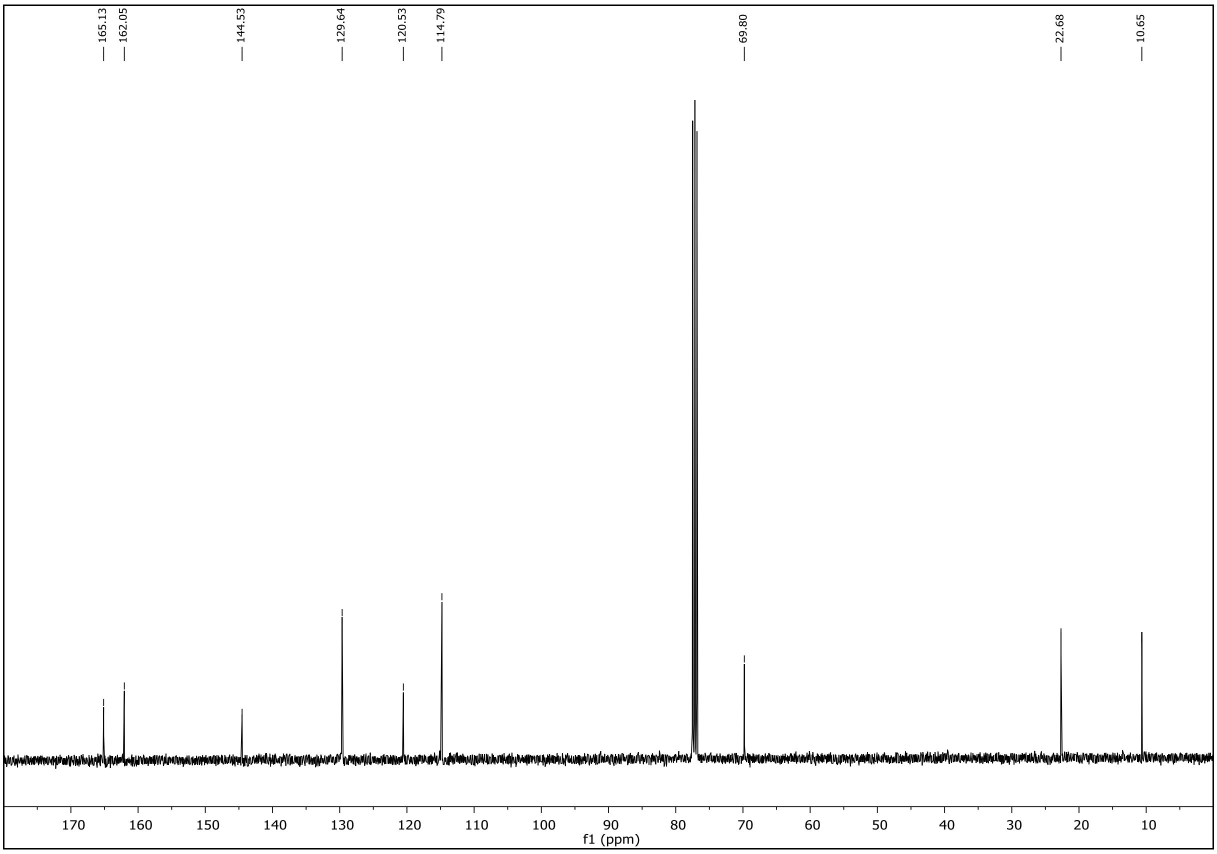
165.13
162.05
144.53
129.64
120.53
114.79
69.80
22.68
10.65
170 160 150 140 130 120 110 100 90 80 70 60 50 40 30 20 10
f1 (ppm)

**2,6,10-Tris(4-propyloxyphenyl)tris([1,2,4]triazolo)[1,5-a:1',5'-c:1'',5''-e][1,3,5]triazine *r*-2**
(ref 1) 200 mg 3,7,11-Tris(4-propyloxyphenyl)tris([1,2,4]triazolo)[4,3-a:4',3'-c:4'',3''-e][1,3,5]triazine in 4 mL octadecane was stirred for 44 h under $N^2$ at 275 °C. Addition of petroleum ether to the cooled mixture, filtration and washing with cold petroleum ether followed by recrystallization from ethanol yielded 129 mg (65 %) of a colorless solid with m.p. > 350 °C.
**$^1$H-NMR** (400 MHz, $CDCl_3$): $\delta$ [ppm] = 8.32 (d, $^3J$ = 8.8 Hz, 2H, 2-H, 6-H, ph), 6.99 ( d, $^3J$ = 8.8 Hz, 2H, 3-H, 5-H, ph), 3.98 (t, $^3J$ = 6.6 Hz, 2H, $OCH_2$), 1.88 – 1.82 (m, 2H, $CH_2$), 1.07 (t, 3H, $CH_3$). **$^{13}$C-NMR** (100 MHz, $CDCl_3$): $\delta$ [ppm] = 165.13 (C-5, trl), 162.05 (C-4, ph), 144.53 (C-3, trl), 129.64 (C-2, C-6, ph), 120.53 (C-1, ph), 114.79 (C-3, C-5, ph), 69.80 ($OCH_2$), 22.68 ($CH_2$), 10.65 ($CH_3$). **IR** (ATR): $\tilde{\nu}$ [cm$^{-1}$] = 2925 w, 1630 s, 1602 vs, 1468 s, 1425 s, 1326 m, 1252 s, 1167 s, 1118 m, 1061 m, 976 m, 834 s, 749 s, 710 m. **HR-ESI:** calcd. for $C_{33}H_{33}N_9O_3$ + $H^+$: 604.2785; found: 604.2805.

**5-(4-Hexyloxyphenyl)-*2H*-tetrazole**
4.07 g (20 mmol) (4-hexyloxy)benzonitrile, 2.72 g (40 mmol) $NaN_3$ and 5.51 g (40 mmol) triethylamine hydrochloride according to general procedure GP5 gave after 6 days 3.36 g (68 %) tetrazole as a colorless solid with m.p.= 163 °C. **$^1$H-NMR** (300 MHz, DMSO-*d*$_6$): $\delta$ [ppm] = 0.87-0.89 (t, 3H, Alkoxy-CH3); 1.30-1.44 (m, 6H, Alkoxy-CH2); 1.67-1.76 (m, 2H, Alkoxy-$CH_2$); 4.05 (t, $^3$J=6.2 Hz, 2H, Ar-O-$CH_2$); 7.14 (d, $^3$J=8.3 Hz, 2H, C(3)-H, C(3´)-H); 7.95 (d, $^3$J=8.3 Hz, 2H, C(4)-H, C(4´)-H). **$^{13}$C-NMR** (75 MHz, DMSO-*d*$_6$): $\delta$ [ppm] = 13.5 (Alkoxy-$CH_3$); 21.5, 24.6, 27.9, 30.4 (Alkoxy-$CH_2$); 67.1 (Ar-O-$CH_2$); 114.6 (C(4), C(4´)), 116.4 (C(2)); 127.9 (C(3), C(3´)); 154.7 (C(5)); 160.0 (C(1)). **IR** (KBr): $\tilde{\nu}$ [cm$^{-1}$] = 3077; 2924; 2853; 1611; 1582; 1499; 1256; 840. **FD-MS:** m/z (%)= 246.2 (100) $[M]^+$, 493.6 (5) $[2M]^+$.

**3,7,11-Tris(4-hexyloxyphenyl)tris([1,2,4]triazolo)[4,3-a:4',3'-c:4'',3''-e][1,3,5]**triazine ***t*-3**
[ref 2] 493.6 mg (2.0 mmol, 3 eq.) 5-(4-Hexyloxyphenyl)-2*H*-tetrazole and 123 mg (0.7 mmol, 1 eq.) cyanuric chloride in 20 mL dry xylenes, 0.25 mL sym-collidine gave, according to the general procedure GP7, 311 mg (0. mmol, 64%) of a colorless solid with m.p. 120 - 122 °C. ($R_f$=0.25 toluene/ethyl acetate). **$^1$H-NMR** (400 MHz, $CDCl_3$): $\delta$ [ppm] = 8.11 (d, $^3J$ = 8.9 Hz, 2H, 2-H, 6-H, ph), 7.07 (d, $^3J$ = 8.9 Hz, 2H, 3-H, 5-H, ph), 4.06 (t, $^3J$ = 6.5 Hz, 2H, $OCH_2$), 1.87 – 1.80 (m, 2H, $CH_2$), 1.51 – 1.34 (m, 6H, $CH_2$), 0.92 (t, $^3J$ = 7.4 Hz, 3H, $CH_3$).
**$^{13}$C-NMR** (75 MHz, $CDCl_3$): $\delta$= 14.1 (Alkoxy-CH3); 22.6, 25.7, 29.1, 31.5 (Alkoxy-CH2); 114.4 (C(4), C(4´)); 115.7 (C(2)); 131.8 (C(3), C(3´)); 140.4 (C(1a)); 150.8 (C(1b)); 162.0 (C(10)).
**IR** (ATR): $\tilde{\nu}$ [cm$^{-1}$] = 2952; 2930; 2857; 1616; 1592; 1484; 1427; 1382; 1293; 1251; 1178; 1120; 1005; 935; 832; 728; 695. **FD-MS:** m/z (%)= 365.0 (10), $[M]^{2+\cdot}$; 729.6 (100) 730.6 (48), 731.7 (11) $[M]^{+\cdot}$.**HR ESI-MS:** m/z calcd. ($C_{42}H_{51}N_9O_3Na$): 752.4013; found: 752.4041.

**2,6,10-Tris(4-hexyloxyphenyl)tris([1,2,4]triazolo)[1,5-a:1',5'-c:1'',5''-e][1,3,5]triazine *r*-3**
100 mg 3,7,11-Tris(4-hexyloxyphenyl)tris([1,2,4]triazolo)[4,3-a:4',3'-c:4'',3''-e][1,3,5]triazine) in 1 mL octadecane was heated for 27 h under nitrogen to 270 °C. Addition of petroleum ether to the cooled mixture precipitated the product. Filtration and wahing of the solid with petroleum ether and chromatography on silica gel gave 62 mg (62 %) of the TTT as colorless solid with m.p. 178 – 180 °C.
**$^1$H-NMR** (400 MHz, $CDCl_3$): $\delta$ [ppm] = 8.34 (d, $^3J$ = 8.8 Hz, 2H, 2-H, 6-H, ph), 7.00 ( d, $^3J$ = 8.8 Hz, 2H, 3-H, 5-H, ph), 4.03 (t, $^3J$ = 6.6 Hz, 2H, $OCH_2$), 1.86 – 1.79 (m, 2H, $CH_2$), 1.51 – 1.34 (m, 6H, $CH_2$), 0.93 (t, 3H, $CH_3$). **$^{13}$C-NMR** (100 MHz, $CDCl_3$): $\delta$ [ppm] = 165.21 (C-5, trl), 162.09 (C-4, ph), 144.58 (C-3, trl), 129.67 (C-2, C-6, ph), 120.54 (C-1, ph), 114.83 (C-3, C-5, ph), 69.36 ($OCH_2$), 31.73, 29.31, 25.85, 22.77 ($CH_2$), 14.21 ($CH_3$). **IR** (ATR): $\tilde{\nu}$ [cm$^{-1}$] = 2923 w, 2853 w, 1625 s, 1607 vs, 1464 s, 1425 m, 1328 w, 1301 m, 1246 s, 1168 m, 1121 w, 1059 w, 953 w, 837 s, 754 s, 714 m. **HR-ESI:** calcd ($C_{42}H_{51}N_9O_3$ + $H^+$): 730,4193; found: 730,4195.

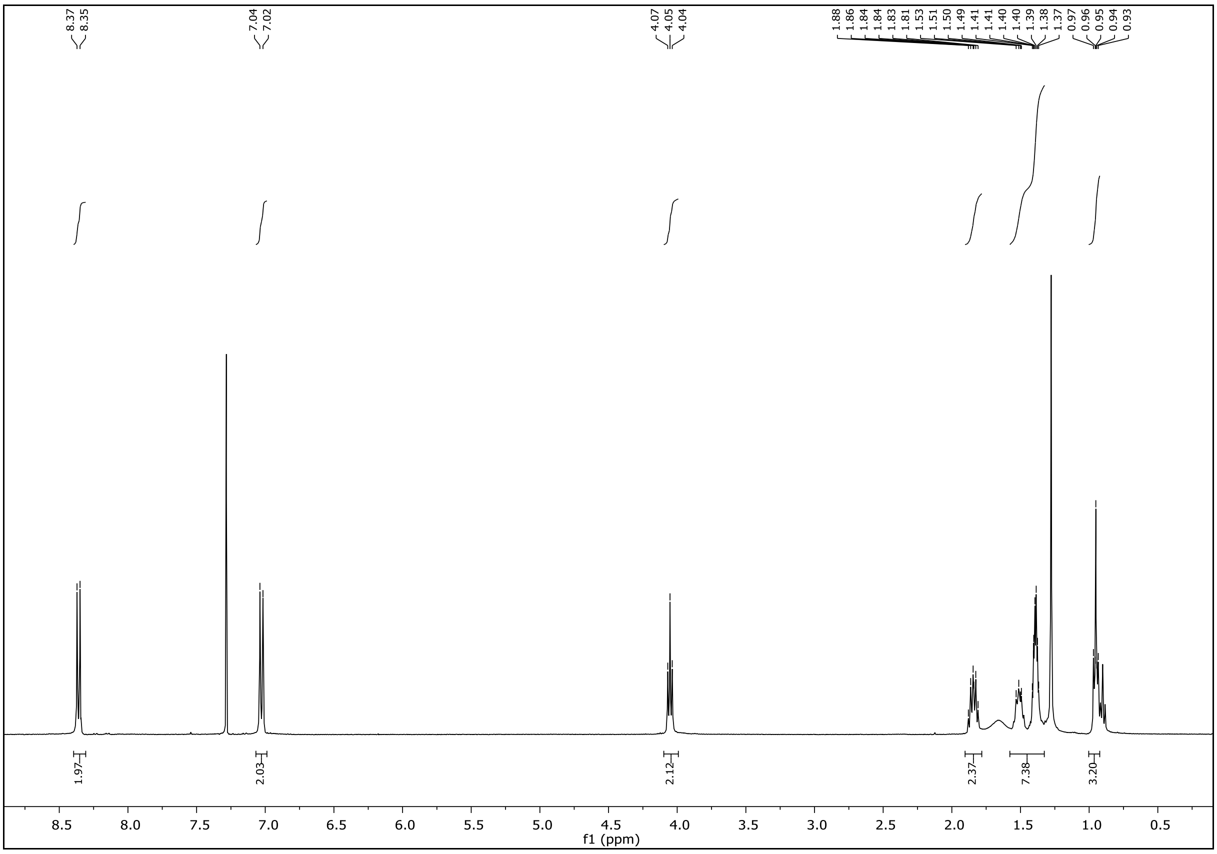


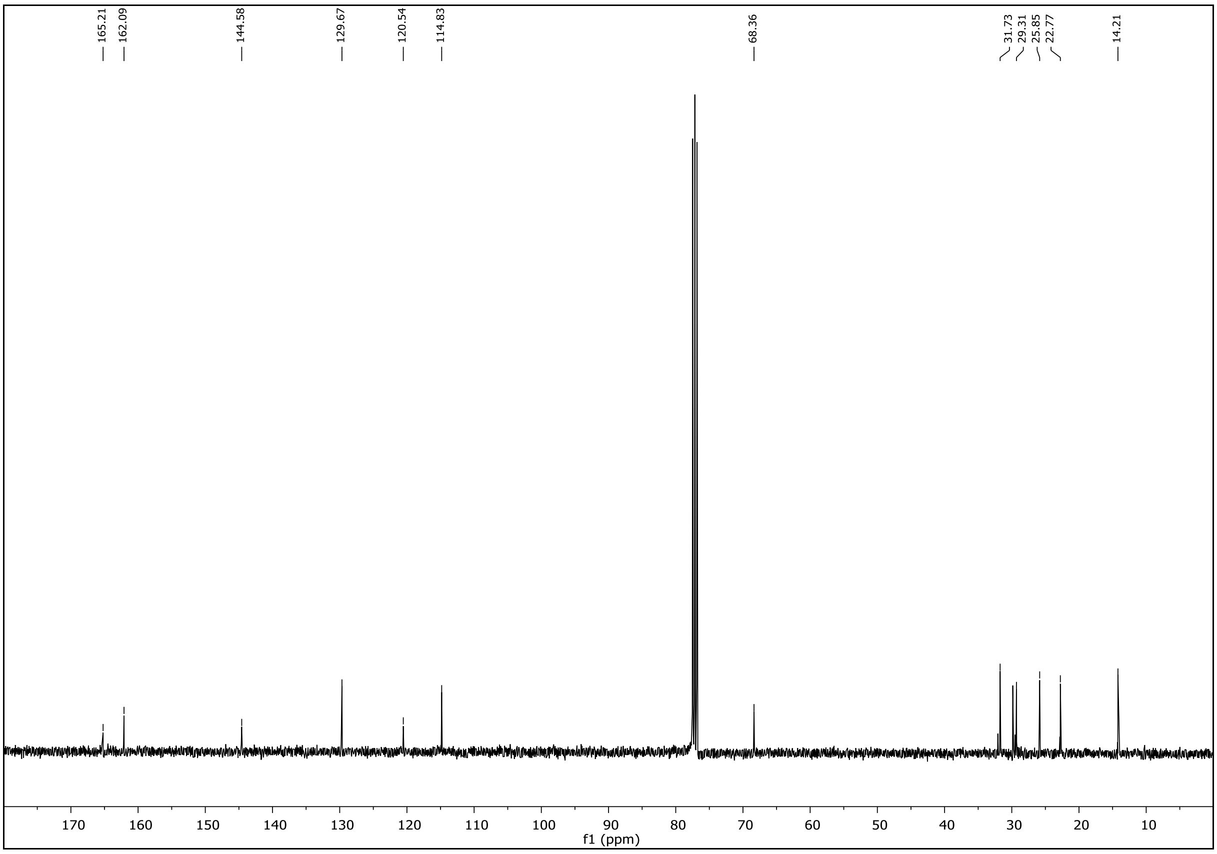


**2,6,10-Tris(4-decyloxyphenyl)tris([1,2,4]triazolo)[1,5-a:1',5'-c:1'',5''-e][1,3,5]triazine *r*-5**
$^1$H-NMR (300 MHz, ca 50 mg in $CDCl_3$): δ= 8.20 ( d, 6 H), 6.65 (d, 6 H), 3.86 (t, 6 H, $NCH_2$), 1.74 (m, 6 H); 1.23 - 1.48 (m, 42 H, $CH_2$), 0.91 (t, 9 H, $CH_3$), $^1$H-NMR (300 MHz, ca 5 mg in

$CDCl_3$): δ= 8.29 ( d, 6 H), 6.98 (d, 6 H), 4.00 (t, 6 H, $NCH_2$), 1.79 (qui, 6 H); 1.49 (qui, 6 H), 1.23 - 1.33 (m, 36 H, $CH_2$), 0.91 (t, 9 H, $CH_3$), $^{13}$C-NMR (75 MHz, $CDCl_3$): δ= 164.97, 161.90, 144.36, 129.51, 120.35, 114.60, 68.17, 31.91, 29.56, 29.44, 29.39, 29.30, 29.26. 29.17, 26.00, 22.17, 14.08; MS (FD): 1355.6 (8%, $M_3^{2+}$), 898 (100%, $M^+$), 448.9 (17 %, $M^{2+}$)

**4-(Tridecyloxy)benzonitrile**

According to the gernal procedure GP1, the compound was obtained in 80 % yield, m.p. 50 – 51 °C (Toluene). **IR:** (ATR) ṽ = 2945, 2915, 2870, 2847, 2219, 1603, 1574, 1506, 1469, 1396, 1300, 1170, 1110, 1030, 1012, 831, 810, 716 cm$^{-1}$; **$^1$H-NMR:** (300 MHz, $CDCl_3$) *δ* = 7.61 – 7.53 (m, 2H), 6.96 – 6.89 (m, 2H), 3.99 (t, $^3J$ = 6.5 Hz, 2H), 1.85 – 1.73 (m, 2H), 1.53 – 1.17 (m, 21H), 0.92 – 0.84 (m, 3H) ppm; **$^{13}$C-NMR:** (75 MHz, $CDCl_3$) *δ* = 162.61 (C-4), 134.10, 119.50, 115.31, 103.76, 68.56 ($OCH_2$), 32.07, 29.82, 29.79, 29.72, 29.68, 29.50, 29.46, 29.12, 26.07, 22.84 ($CH_2$), 14.27 ($CH_3$) ppm; **FD-MS:**m/z (%): 301.7 (100), 302.7 (15), 303.8 (1) $[M]^{\bullet+}$.

**5-(4-(Tridecyloxy)phenyl)-2*H*-tetrazole**

According to the gernal procedure GP5, the compound was obtained in 88 % yield, m.p. =142 °C (Ethanol). **IR:** (ATR) ṽ = 2911, 2847, 1615, 1582, 1503, 1259, 1040, 988, 836 cm$^{-1}$; **$^1$H-NMR:**(300 MHz, $CDCl_3$ + 0.1 mL DMSO-*d*$_6$) *δ* = 7.63 (d, $^3J$ = 8.9 Hz, 2H, H-2, H-6, ph), 6.64 (d, $^3J$ = 8.9 Hz, 2H, H-3, H-5, ph), 3.65 (t, $^3J$ = 6.5 Hz, 2H, O$CH_2$), 1.43 (p, $^3J$ = 6.5 Hz, 2H, $CH_2$), 1.17 – 0.84 (m, 21H, $CH_2$), 0.56 – 0.45 (m, 3H, $CH_3$) ppm.

**3,7,11-Tris(4-(tridecyloxy)phenyl)tris([1,2,4]triazolo)[4,3-a:4',3'-c:4'',3''-e][1,3,5]triazine *t*-7**

According to the gernal procedure GP5, the compound was obtained in 90 % yield after chromatography on $SiO_2$ + 5 cm $Al_2O_3$; toluene:ethyl acetate = 30:1. M. p. : 78 (POM). **IR:** (ATR) ṽ = 2921, 2851, 1610, 1592, 1485, 1469, 1427, 1294, 1253, 1179, 1007, 950, 832, 773, 727 cm$^{-1}$; **$^1$H-NMR:** (300 MHz, $CDCl_3$) *δ* = 8.14 – 8.06 (m, 2H, H-2, H-6, ph), 7.11 – 7.03 (m, 2H, H-3, H-5, ph), 4.06 (t, $^3J$ = 6.5 Hz, 2H, O$CH_2$), 1.83 (p, $^3J$ = 6.6 Hz, 2H, $CH_2$), 1.54 – 1.10 (m, 23H, $CH_2$), 0.92 – 0.83 (m, 3H, $CH_3$) ppm; **$^{13}$C-NMR:** (75 MHz, $CDCl_3$) *δ* = 162.17 (C-4, ph), 151.02 (C-5, trl), 140.61 (C-3, trl), 131.97 (C-2, C-6,ph), 115.88 (C-1, ph), 114.63 (C-3, C-5, ph), 68.38 ($OCH_2$), 32.07, 29.84, 29.82, 29.80, 29.77, 29.74, 29.54, 29.51, 29.29, 26.18, 22.84 ($CH_2$), 14.28 ($CH_3$) ppm; **FD-MS:** m/z (%): 1024.5 (100), 1025.6 (68) 6 (68) $[M]^{\bullet+}$.

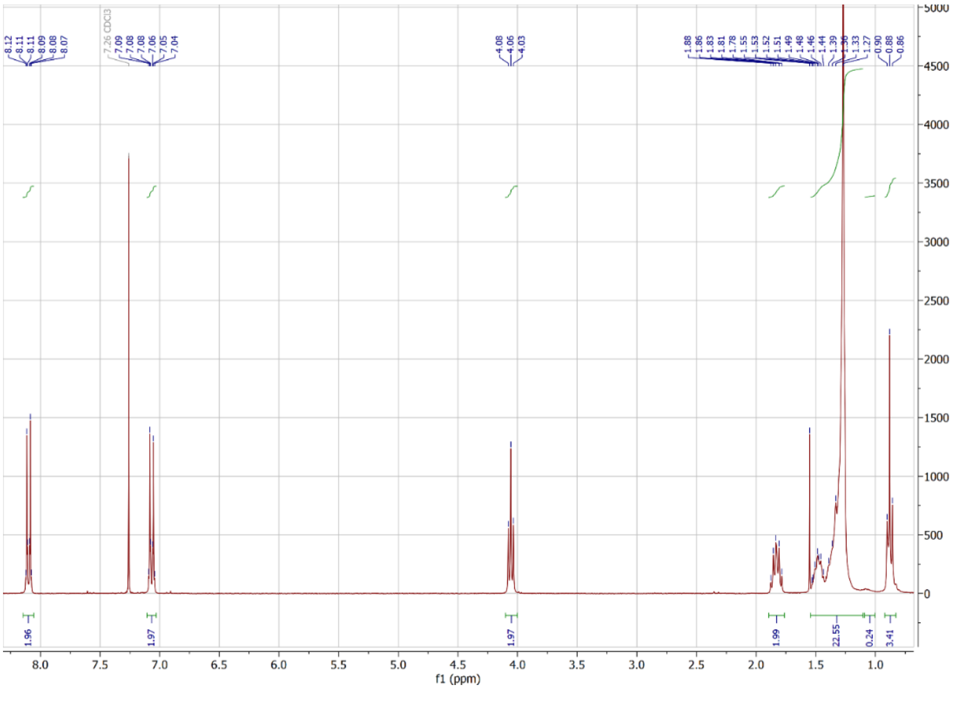

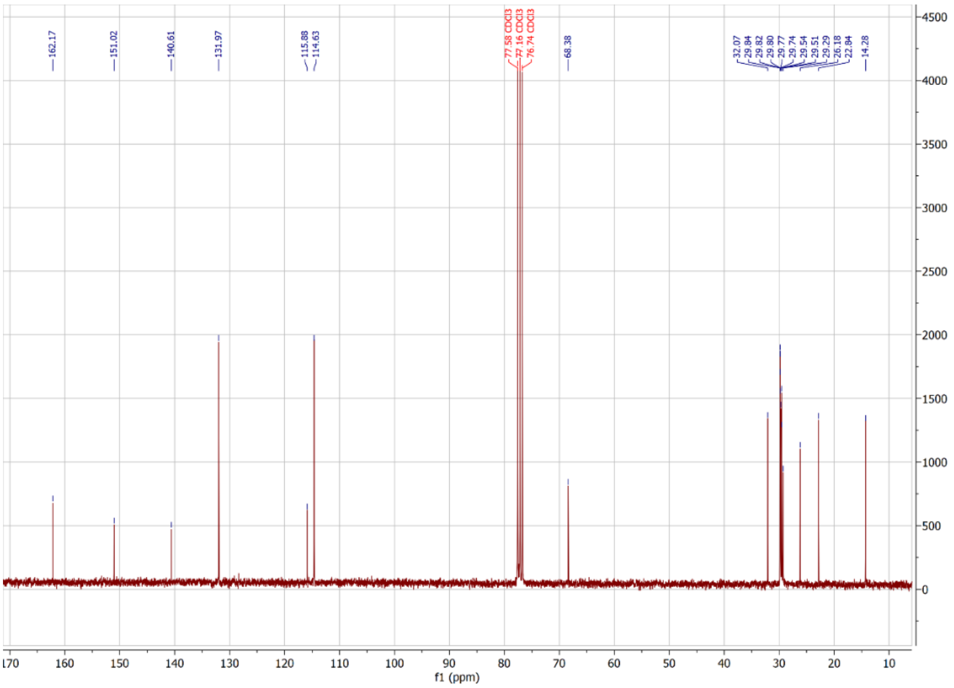


$^1$H- and $^{13}$C-NMR spectra ***t*-7**

**Methyl 3-decyloxybenzoate**

According to the general procedure, 20.0 g (131 mmol) methyl 3-hydroxybenzoate and 43.4 mL (210 mmol) 1-bromodecane and 54.5 g (394 mmol) potassium carbonate in acetonitrile gave 28.3 g (97 mmol, 74%) colorless crystals with m.p. = 35 °C.
**$^1$H-NMR** (300MHz, $CDCl_3$): δ (ppm) = 0.88 (t, 3H, $^3J$=6.8Hz, $CH_3$); 1.22-1.39 (m, 12H, $CH_2$); 1.46 (qui, 2H, $^3J$=7.0Hz, $CH_2$); 1.79 (qui, 2H, $^3J$=6.9, $CH_2$); 3.91 (s, 3H, OMe); 3.99 (t, 2H, $^3J$=6.4Hz, Ar-O-$CH_2$); 7.09 (dd, 1H, $^3J$=8.8Hz, $^4J$=2.2Hz, C(7)-H); 7.32 (t, 1H, $^3J$=7.9Hz, C(6)H); 7.54 (s, 1H, C(3)-H); 7.61 (d, 1H, C(5)-H). **$^{13}$C-NMR** (75MHz, $CDCl_3$): δ(ppm) = 14.1($CH_3$); 22.7, 26.0, 29.2, 29.3, 29.4, 29.5, 31.9, 34.1 ($CH_2$); 52.1 (OMe); 68.2 (Ar-O-$CH_2$); 114.6 C(3)); 119.9 (C(5)); 121.7 (C(7)); 129.3 (C(6)); 131.3 (C(2)); 159.1 (C(4)); 167.0 (C(1)). **IR** (ATR): v[$cm^{-1}$] = 2922, 2851, 1719, 1587, 1470, 1435, 1278, 1225, 1077, 1029, 795, 758. **ESI-MS**: m/z(%) = 315.2(100), 316.2(5) [M+Na]+.

**3-Decyloxybenzoic acid**

According to the general procedure, 14.0 g (48 mmol) methyl 3-decyloxybenzoate and 9.4 g (167 mmol) KOH in ethanol gave 13.6 g (48 mmol, 100%) colorless needles with m.p.: 82°C
**$^1$H-NMR** (300MHz, $CDCl_3$): δ (ppm) = 0.79 (t, 3H, $^3J$=6.6Hz, $CH_3$); 1.17-1.41 (m, 12H, $CH_2$); 1.47(qui, 2H, $^3J$ = 7.3Hz, $CH_2$); 1.81 (qui, 2H, $^3J$ = 6.9, $CH_2$); 4.01 (t, 2H, $^3J$=6.4Hz, Ar-O-$CH_2$); 7.15 (dd, 1H, $^3J$ = 8.5Hz, $^4J$ = 2.6Hz, C(7)-H); 7.37 (t, 1H, $^3J$ = 7.7Hz, C(6)-H); 7.62(s, 1H, C(3)-H); 7.71 (d, 1H, $^3J$ = 7.7Hz, C(5)-H). **$^{13}$C-NMR** (75MHz, $CDCl_3$): δ(ppm)=14.1 ($CH_3$); 22.7, 26.0, 29.2, 29.3, 29.4, 29.5, 31.9 ($CH_2$); 68.3 (Ar-O-$CH_2$); 115.0 (C(3)); 120.9 (C(5)); 122.4 (C(7)); 129.5 (C(6)); 130.5 (C(2)); 159.2 (C(4)); 172.1 (C(1)). **IR** (ATR): v [$cm^{-1}$]=2919, 2849, 1677, 1588, 1455, 1311, 1246, 1120, 1033, 937, 776, 755, 677. **FD-MS**: m/z(%) = 277.9(100), 278.9(20), 279.9(1) $[M]^+$.

**3-Decyloxybenzamide**

According to the general procedure, (12.6 g , 45 mmol) 3-decyloxybenzoic acid, excess thionyl chloride in toluene and excess ammonia (25% in water) gave an off-white solid (44 mmol, 12.1 g) with m.p. = 107-108°C
**$^1$H-NMR** (300MHz, $CDCl_3$): δ (ppm) = 0.77(t, 3H, $^3J$=6.6Hz, $CH_3$); 1.05-1.27 (m, 12H, $CH_2$); 1.34 (qi, 2H, $^3J$ = 7.1Hz, $CH_2$); 1.67 (qi, 2H, $^3J$ = 6.9, $CH_2$); 3.88(t, 2H, $^3J$ = 6.4Hz, Ar-O-$CH_2$); 6.03 (s, 1H, R-$NH_2$); 6.81 (s, 1H, R-$NH_2$); 6.92 (dd, 1H, $^3J$ = 8.1Hz, $^4J$ = 2.6Hz, C(7)-H); 7.21 (t, 1H, $^3J$ = 7.7Hz, C(6)-H); 7.28-7.34 (m, 2H, C(5)-H, C(3)-H). **$^{13}$C-NMR** (75MHz,

$CDCl_3$): δ (ppm) = 14.0 ($CH_3$); 22.5, 25.9, 29.1, 29.2, 29.2, 29.4, 31.8 ($CH_2$); 68.1 (Ar-$OCH_2$); 113.2 (C(3)); 118.2 (C(5)); 119.3 (C(7)); 129.3 (C(6)); 135.0 (C(2)); 159.1 (C(4)); 169.2 (C(1)). **IR** (ATR): ν [$cm^{-1}$] = 3363, 3176, 2919, 2854, 1656, 1623, 1583, 1449, 1390, 1249, 1131, 1032, 798, 752, 676. **FD-MS**: m/z(%)=276.9(100), 277.9(35), 278.9(2) $[M]^+$; 554.0(2) $[2M]^+$.

**5-(3-Decyloxyphenyl)tetrazole**
According to the general procedure, 3-decyloxybenzamide (4.0 g, 14.4 mmol), $NaN_3$ (7.0 g, 107.7 mmol), and $SiCl_4$ (4.1 mL, 35.9 mmol) in acetonitrile gave an off-white solid (3.9 g, 12.8 mmol, 89%) with m.p. = 131 °C.
**$^1$H-NMR** (300MHz, $CDCl_3$): δ (ppm) = 0.84 (t, 3H, $^3J$ = 6.6Hz, $CH_3$); 1.13-1.34 (m, 12H, $CH_2$); 1.35-1.48 (m, 2H, $CH_2$); 1.77 (qui, 2H, $^3J$ = 7.2, $CH_2$); 3.99 (t, 2H, $^3J$ = 6.6Hz, Ar-O-$CH_2$); 7.00 (dd, 1H, $^3J$ = 8.4Hz, $^4J$ = 2.4Hz, C(7)-H); 7.36 (t, 1H, $^3J$ = 7.6Hz, C(6)-H); 7.63 (dd, 1H, $^3J$ = 7.6Hz, $^4J$ = 2.4Hz, C(5)-H); 7.64u(t, 1H, $^4J$ = 2.4Hz, C(3)-H). **$^{13}$C-NMR** (100MHz, $CDCl_3$): δ (ppm) = 14.0 ($CH_3$); 22.5, 25.9, 29.1, 29.2, 29.3, 29.4, 31.8 ($CH_2$); 68.1 ($OCH_2$); 112.6 (C(3)); 117.8 (C(5)); 119.2 (C(7)); 125.4 (C(2)); 130.1 (C(6)); 156.1 (C(1)); 159.5 (C(4)). **IR** (ATR): ν [$cm^{-1}$] = 2921, 2852, 1604, 1553, 1490, 1455, 1254, 1150, 1059, 1038, 1016, 994, 868, 796, 743, 683. **FD-MS**: m/z(%) = 302.0(72), 303.0(38), 304.0(4) $[M]^+$; 605.1(100), 606.0(44), 607.1(11), 608.1(1) $[2M]^+$; 907.1(55), 908.1(27), 909.2(7) $[3M]^+$.

**3 ,7,11-Tris(3-(decyloxy)phenyl)tris[1,2,4]triazolo[4,3-a:4',3'-c:4'',3''-e]-[1,3,5]triazine *t*-8**
According to the general procedure, 5-(3-decyloxyphenyl)tetrazole (541 mg, 1.8 mmol), (100 mg, 0.5 mmol) cyanuric chloride in xylenes with 1 mL collidine gave, after 3 h reflux, aqueous work-up and chromatography on silica gel with toluene/ethyl acetate 20/1 ($R_f$ =0.4) an off-white solid (400 mg, 83 %) with m.p. = 132-136°C.
**$^1$H-NMR** (400MHz, $CDCl_3$): δ (ppm) = 0.82-0.91 (m, 9H, $CH_3$); 1.21-1.40 (m, 36H, $CH_2$); 1.40-1.51 (m, 6H, $CH_2$); 1.82 (qui, 6H, $^3J$ = 7.1Hz, $^3J$ = 6.9Hz, $CH_2$); 4.05 (t, 6H, $^3J$ = 6.4Hz, Ar-O-$CH_2$); 7.16 (d, 3H, $^3J$ = 8.2Hz, C(7)-H); 7.48 (t, 3H, $^3J$ = 8.0Hz, C(6)-H); 7.65 (s, 3H, C(3)-H); 7.70 (d, 3H, $^3J$ = 7.6Hz, C(5)-H). **$^{13}$C-NMR** (75MHz, $CDCl_3$): δ (ppm) = 14.1 ($CH_3$); 22.7, 26.0, 29.2, 29.3, 29.4, 29.6, 31.9 ($CH_2$); 68.3 (Ar-$OCH_2$); 115.6 (C(3)); 118.8 (C(5)); 122.3 (C(7)); 124.8 (C(2)); 129.6 (C(6)); 140.5 (C(1a)); 150.8 (C(1b)); 159.0 (C(4)). **IR** (ATR): ν [$cm^{-1}$] = 2921, 2852, 1579, 1467, 1444, 1307, 1235, 1164, 793, 688. **FD-MS**: m/z(%) = 449.2(1) $[M]^{2+}$; 897.3(100), 898.4(59), 899.3(16), 900.4(2) $[M]^+$. **HR ESI-MS**: m/z calcd.: 898.6071 $[M+H]^+$; found: 898.6082.

**3,7,11-Tris(2-(decyloxy)phenyl)tris([1,2,4]triazolo)[4,3-a:4',3'-c:4'',3''-e][1,3,5]triazine *t*-9**
A suspension of 5-(2-(decyloxy)phenyl)-2*H*-tetrazole (700 mg, 2.32 mmol), cyanuric chloride (123 mg, 0.70 mmol, 1.0 eq.) and 2,4,6-collidine (1 mL) in dried xylene (10 mL) was stirred over night at room temperature and under nitrogen as the inert gas. Afterwards, the reaction mixture was slowly heated to 70 °C (heating rate: ca. 10 °C/h) and the resulting mixture was stirred at 70 °C for 4 d. Then 2N HCl (10 mL) was added and the mixture was extracted with ethyl acetate (4 x 30 mL). The combined organic layers were washed with water (2 x 40 mL) and with brine (60 mL) and dried over magnesium sulfate. Removal of the organic solvent in vacuo gave the crude product mixture (1.080 g). The crude product was adsorbed on a small amount of silica gel (ca. 10 g) and then subjected to column chromatography on basic alumina (20 g, fill level: l = 10 cm, d = 2 cm, eluent: toluene/ethyl acetate, 9:1) to yield 350 mg (0.39 mmol, 58%) of a highly viscous, yellowish oil.
**$^1$HNMR** (300 MHz, $CDCl_3$, 298 K): δ (ppm) = 7.69 – 7.46 (m, 6H, 4’-H, 6’-H), 7.21 – 6.99 (m, 6H, 3’-H, 5’-H), 4.00 (t, $^3J_{HH}$ = 7.0 Hz, 18H, 1’’-H), 1.46 – 0.98 (m, 52H, 2’’-H – 9’’-H), 0.84 (t, $^3J_{HH}$ = 6.9 Hz, 9H, 10’’-H). **$^{13}$C-NMR** (75MHz, $CDCl_3$): δ(ppm) = 158.3 (C-2’), 147.9 (C-4), 140.2 (C-1),133.6 (C-6’), 131.7 (C-4’), 120.7 (C-5’), 114.1 (C-1’), 112.2 (C-3’), 68.3 (C-1’’), 32.0, 29.7, 29.7, 29.4, 29.4, 29.0, 25.8, 22.8 (*C*H2, sup.), 14.2 (C-10’’). **FD-MS:***m/z* (%) = 449.7 (0.6)$[M^{2+}]$, 897.7 (100.0)$[M^+]$, 898.7 (60.8), 899.8 (18.7), 900.6 (3.5). **IR:** ν[$cm^{-1}$] = 3074 w, 2924 s, 2853 s, 1596ss, 1534 w, 1466 s, 1423 w, 1394 w, 1325 m, 1278 s, 1250s, 1163 m, 1126 m, 1088 w, 1042 m, 1014 m, 952 m, 824 w, 751 s, 729 w, 689 m, 668 w. **EA:**$C_{54}H_{75}N_9O_3$ (898.23) calcd.(%) C 72.21, N 14.03, found (%) C 72.30, N 13.78.

**2,3-Di(decyloxy)benzoic acid**

Under nitrogen, *n*-butyllithium (14.30 mL, 2.50 mol/L in hexane) was added to 7.15 g (18.00 mmol) 1,2-di(decyloxy)benzene in 60 mL dry THF. After 12 h. at ambient temperature, $CO_2$ was bubbled into the solution. Acidulated with 2 N HCL and Extraction with ether followed by recrystallization form toluene yielded 3.30 g (7.6 mmol, 43 %) of a colorless solid with m.p. = 54 – 56 °C (toluene). **$^1$H-NMR** (400 MHz, $CDCl_3$): $\delta$ [ppm] = 11.69 (s, 1H, COOH), 7.72 (dd, $^3J$ = 7.6, $^4J$ = 2.0 Hz, 1H, 6-H, ph), 7.18 – 7.11 (m, 2H, 4-H, 5-H, ph), 4.27 (t, $^3J$ = 6.9 Hz, 2H, $OCH_2$), 4.02 (t, $^3J$ = 6.4 Hz, 2H, $OCH_2$), 1.93 – 1.77 (m, 4H, $CH_2$), 1.53 – 1.14 (m, 28H, $CH_2$), 0.88 (t, $^3J$ = 6.8 Hz, 6H, $CH_3$). **$^{13}$C-NMR** (100 MHz, $CDCl_3$): $\delta$ [ppm] = 165.62 (C-7, COOH), 151.59 (C-3, ph), 147.46 (C-2, ph), 124.77 (CH-ph), 123.72 (CH-ph), 122.33 (C-1, ph), 118.24 (CH-ph), 75.68, 69.23 ($OCH_2$), 31.97, 30.02, 29.69, 29.65, 29.61, 29.45, 29.41, 29.39, 29.35, 26.27, 25.76, 22.76 ($CH_2$), 14.18 ($CH_3$). **IR** (ATR): $\tilde{\upsilon}$ [cm$^{-1}$] = 2952 m, 2915 ss, 2871 m, 2848 s, 1689 s, 1577 m, 1474 s, 1460 s, 1429 m, 1382 w, 1302 s, 1263 m, 1223 m, 1161 w, 1077 m, 1031 m, 970 m, 932 m, 795 w, 747 s, 716 m, 659 m. **FD-MS:** m/z (%) = 434.8 $[M]^+$; 869.6 $[M_2]^+$. **HR-ESI**: calcd. for $C_{27}H_{46}N_4O_2 + H^+$: 435.3474; found: 435.3474.

**2,3-Di(decyloxy)benzamide**

2.00 g (4,6 mmol) 2,3-di(decyloxy)benzoic acid and 1.70 mL (23,0 mmol) $SOCl_2$ in 30 mL dry toluene with 1 drop DMF were reflued for 12 h. The solvent was evaporated, the residue dissolved in 10 mL dioxane and this solution was added dropwise into 200ml heavily stirred, ice-cold, concentrated ammonia solution. Precipitated amide and chloroform extracts were dried, concentrated, and the residue recrystallized from toluene. Yield quantitative, m.p. 61 – 63 °C (toluene). **$^1$H-NMR** (400 MHz, $CDCl_3$): $\delta$ [ppm] = 7.98 (s, 1H, $NH_2$), 7.68 (dd, $^3J$ = 8.0, $^4J$ = 1.7 Hz, 1H, 6-H, ph), 7.11 (t, $^3J$ = 8.0 Hz, 1H, 5-H, ph), 7.04 (dd, $^3J$ = 8.0, $^4J$ = 1.7 Hz, 1H, 4-H, ph), 5.96 (s, 1H, $NH_2$), 4.07 (t, $^3J$ = 6.9 Hz, 2H, $OCH_2$), 3.99 (t, $^3J$ = 6.4 Hz, 2H, $OCH_2$), 1.88 – 1.76 (m, 4H, $CH_2$), 1.54 – 1.18 (m, 28H, $CH_2$), 0.88 (t, $^3J$ = 6.8 Hz, 6H, $CH_3$). **$^{13}$C-NMR** (100 MHz, $CDCl_3$): $\delta$ [ppm] = 168.15 (C-7, $CONH_2$), 152.28 (C-2, ph), 147.63 (C-3, ph), 124.25 (CH-ph), 122.79 (CH-ph), 117.23 (CH-ph), 74.77, 69.11 ($OCH_2$), 32.04, 30.42, 29.77, 29.72, 29.60, 29.54, 29.47, 26.34, 26.09, 22.82 ($CH_2$), 14.25 ($CH_3$). **IR** (ATR): $\tilde{\upsilon}$ [cm$^{-1}$] = 3419 m, 3155 bw, 2917 s, 2850 m, 1772 w, 1730 w, 1702 w, 1679 ss, 1574 m, 1471 m, 1379 m, 1312 w, 1270 s, 1181 m, 1069 m, 1013 m, 973 w, 925 w, 792 w, 750 m, 716 m. **FD-MS:** m/z (%) = 433.8 $[M]^+$.

**5-(2,3-Di(decyloxy)phenyl)-2*H*-tetrazole**

According to GP6, 0.90 g (13.8 mmol) $NaN_3$ was added to 0.78 g (4.6 mmol) $SiCl_4$ in 25 mL dry acetonitrile and the mixture was stirred for 1 h at ambient temperature. 1.00 g (2.3 mmol) 4-(2,3-di(decyloxy)benzamid was added, after 12 h stirring at ambient temperature, further 0.45 g (6.9 mmol) $NaN_3$ and 0.39 g (2.3 mmol) $SiCl_4$ were added, the mixture was heated for 1 h to 50 °C. Addition of water, extraction with ether (3 x 50 mL), evaporation and recrystallization from toluene yielded 0.79 g (1.7 mmol, 70 %) of a colorless solid with m.p. = 84 – 86 °C (ethanol). **$^1$H-NMR** (400 MHz, $CDCl_3$): $\delta$ [ppm] = 13.00 (s, 1H, NH), 7.91 (d, $^3J$ = 8.0 Hz, 1H, 6-H, ph), 7.20 (t, $^3J$ = 8.0 Hz, 1H, 5-H, ph), 7.08 (d, $^3J$ = 8.0 Hz, 1H, 4-H, ph), 4.19 (t, $^3J$ = 7.0 Hz, 2H, $OCH_2$), 4.05 (t, $^3J$ = 6.3 Hz, 2H, $OCH_2$), 1.91 – 1.76 (m, 4H, $CH_2$), 1.56 – 1.16 (m, 28H, $CH_2$), 0.88 (t, $^3J$ = 6.2 Hz, 6H, $CH_3$). **$^{13}$C-NMR** (100 MHz, $CDCl_3$): $\delta$ [ppm] = 152.45, 152.08 (C-5, tet; C-2, ph), 146.11 (C-3, ph), 125.11 (CH-ph), 120.91 (CH-ph), 116.80 (C-1, ph), 116.56 (CH-ph), 74.49, 69.14 ($OCH_2$), 32.04, 32.02, 30.55, 29.76, 29.72, 29.67, 29.52, 29.48, 29.44, 26.36, 26.03, 22.82 ($CH_2$), 14.25 ($CH_3$). **IR** (ATR): $\tilde{\upsilon}$ [cm$^{-1}$] = 3730 w, 2952 m, 2919 vs, 2853 s, 1569 w, 1506 m, 1467 s, 1373 w, 1270 m, 1255 m, 1224 m, 1101 m, 1049 w, 1016 w, 795 w, 753 w, 737 w, 722 w, 668 m. **HR-ESI:** calcd. for $C_{27}H_{46}N_4O_2 + Na^+$: 481.3518; found: 481.3520.

**3,7,11-Tris(2,3-di(decyloxy)phenyl)tris([1,2,4]triazolo)[4,3-a:4',3'-c:4'',3''-e][1,3,5]triazine *t*-10**

According to the general procedure GP6, 0.30 g (0.7 mmol) 5-(2,3-di(decyloxyphenyl)-2*H*-tetrazole, 37 mg (0.2 mmol) cyanuric chloride, and 0.2 mL *sym*-collidine in 15 mL xylenes were stirred for 30 min and successively heated to 80 . When the reaction was finished (TLC), acidic work-up, extraction with ethyl acetate, evaporation of the solvents and chromatography of the residue through a silica column and alumina with a gradient toluene to toluene/ethyl acetate 40/1 yielded 109 mg (40 %) of a colorless solid with m.p. = 68 – 70 °C . $^{1}$**H-NMR** (400 MHz, $CDCl_3$): $\delta$ [ppm] = 7.16 – 7.13 (m, 9H, 4-H, 5-H, 6-H, ph), 4.07 – 4.01 (m, 12H, $OCH_2$), 1.88 – 1.79 (m, 6H, $CH_2$), 1.53 – 1.44 (m, 6H, $CH_2$), 1.40 – 1.02 (m, 84H, $CH_2$), 0.86 (m, 18H, $CH_3$). $^{13}$**C-NMR** (100 MHz, $CDCl_3$): $\delta$ [ppm] = 152.14, 148.45, 147.41 (C-5, trl; CO-ph), 139.99 (C-3, trl), 123.73 (CH-ph), 122.93 (CH-ph), 119.46, 117.48 (C-1, CH-ph), 73.93, 69.21 ($OCH_2$), 32.06, 30.16, 29.79, 29.75, 29.59, 29.52, 29.45, 26.38, 25.89, 22.83 ($CH_2$), 14.26 ($CH_3$). **IR** (ATR): $\tilde{\nu}$ [$cm^{-1}$] = 2923 vs, 2853 s, 1598 s, 1531 w, 1466 s, 1379 w, 1324 w, 1269 s, 1231 w, 1146 w, 1054 w, 990 w, 786 w, 722 w, 668 w. **FD-MS:** m/z (%) = 1367.1 $[M]^+$.

**5-(2,4-Di(decyloxy)phenyl)-2*H*-tetrazole**

According to the general procedure, 2.00 g (4.80 mmol) 2,4-didecyloxybenzonitrile, 1.11 g (17.00 mmol) $NaN_3$, 2.32 g (17 mmol) $NEt_3HCl$ in 50 mL toluene were refluxed until the nitrile had been consumed (TLC). Usual work-up and recrystallization from ethanol yielded 1.43 g (3.10 mmol, 65 %) of a colorless solid with m.p. = 105 - 107 °C (ethanol). $^{1}$**H-NMR** (400 MHz, $CDCl_3$): $\delta$ [ppm] = 12.71 (s, 1H, NH), 8.34 (d, $^3J$ = 8.7 Hz, 1H, 6-H, ph), 6.68 (dd, $^3J$ = 8.7, $^4J$ = 2.1 Hz, 1H, 5-H, ph), 6.58 (d, $^4J$ = 2.1 Hz, 1H, 3-H, ph), 4.19 (t, $^3J$ = 6.8 Hz, 2H, $OCH_2$), 4.02 (t, $^3J$ = 6.5 Hz, 2H, $OCH_2$), 2.02 – 1.91 (m, 2H, $CH_2$), 1.85 – 1.75 (m, 2H, $CH_2$), 1.56 – 1.17 (m, 28H, $CH_2$), 0.88 (t, $^3J$ = 6.8 Hz, 6H, $CH_3$). $^{13}$**C-NMR** (100 MHz, $CDCl_3$): $\delta$ [ppm] = 163.50, 157.61, 152.33 (C-5, tet; C-2, C-4, ph), 131.28 (CH-ph), 106.98 (CH-ph), 104.47 (C-1, ph), 100.01 (CH-ph), 69.46, 68.66 ($OCH_2$), 32.03, 31.99, 29.69, 29.62, 29.49, 29.43, 29.33, 29.26, 26.28, 26.12, 22.81 ($CH_2$), 14.24 ($CH_3$). **IR** (ATR): $\tilde{\nu}$ [$cm^{-1}$] = 3733 m, 2956 m, 2921 vs, 1614 vs, 1579 m, 1539 w, 1488 s, 1397 w, 1300 s, 1255 m, 1188 s, 1133 s, 1044 s, 837 m, 750 m, 671 m. **HR-ESI:** calcd. for $C_{27}H_{46}N_4O_2 + Na^+$: 481.3518; found: 481.3505.

**3,7,11-Tris(2,4-di(decyloxy)phenyl)tris[1,2,4]triazolo[4,3-a:4′,3′-c:4″,3″-e][1,3,5]triazine *t*-11**

According to the General Procedure GP7, 0.25 g (0.55 mmol) 5-(2,4-di(decyloxyphenyl)-2*H*-tetrazole and 0.2 mL *sym*-collidine in 25 mL xylenes were stirred for 30 min. Addition of 1 mL of a 0.1 M solution of cyanuric chloride in toluene to the stirred mixture. After 1 h, 0.1 mL of this solution was added and repeated every 2 h until the reaction was complete (TLC). Addition of petroleum ether and filtration through a pad of silica, evaporation of the solvents and chromatography of the residue through a silica column with petroleum ether/ethyl acetate 20/1 yielded 184 mg (72 %) of a colorless solid with m.p. = 49 – 51 °C (POM). $^{1}$**H-NMR** (400 MHz, $CDCl_3$): $\delta$ [ppm] = 7.53 (d, $^3J$ = 9.0 Hz, 3H, 6-H, ph), 6.60 (m, 6H, 3-H, 5-H, ph), 4.02 (t, $^3J$ = 6.5 Hz, 6H, $OCH_2$), 3.95 (t, $^3J$ = 7.0 Hz, 6H, $OCH_2$), 1.86 – 1.78 (m, 6H, $CH_2$), 1.53 – 1.44 (m, 6H, $CH_2$), 1.39 – 1.11 (m, 84H, $CH_2$), 0.90 – 0.83 (m, 18H, $CH_3$). $^{13}$**C-NMR** (100 MHz, $CDCl_3$): $\delta$ [ppm] = 163.80 (C-2, ph), 159.59 (C-4, ph), 147.91 (C-5, trl), 140.12 (C 3, trl), 132.42 (CH-ph), 106.56 (CH-ph), 105.74 (C-1, ph), 99.88 (CH-ph), 68.39, 68.28 ($OCH_2$), 32.04, 32.00, 29.72, 29.70, 29.63, 29.53, 29.47, 29.38, 29.35, 28.90, 26.19, 25.71, 22.82, 22.80 ($CH_2$), 14.26, 14.23 ($CH_3$). **IR** (ATR): $\tilde{\nu}$ [$cm^{-1}$] = 2921 m, 2852 m, 1614 m, 1595 s, 1542 w, 1646 m, 1389 w, 1303 m, 1181 s, 1137 m, 1089 w, 1021 w, 834 w, 722 w. **HR-ESI:** calcd. for $C_{84}H_{135}N_9O_6 + H^+$: 1367.0614; found: 1367.0614.

**5-(2,5-Di(decyloxy)phenyl)-2*H*-tetrazole**

According to the general procedure GP5, 1.43 g (3.4 mmol) 2,5-didecyloxy)benzonitrile, 0.78 g (12.00 mmol) $NaN_3$, 1.66 g (12 mmol) $NEt_3HCl$ in 30 mL toluene were refluxed until the nitrile had been consumed (TLC). Aqueous work-up and recrystallization from ethanol yielded 0.61 g (1.72 mmol, 54 %) of a colorless solid with m.p. = 115 - 116 °C (ethanol).

**$^{1}$H-NMR** (400 MHz, $CDCl_3$): *δ* [ppm] = 12.98 (s, 1H, NH), 7.93 (d,$^{4}J$ = 3.0 Hz, 1H, 6-H, ph), 7.05 (dd, $^{3}J$ = 9.1 Hz, $^{4}J$ = 3.0 Hz, 1H, 4-H, ph), 7.00 (d, $^{3}J$ = 9.1 Hz, 1H, 3-H, ph), 4.18 (t, $^{3}J$ = 6.8 Hz, 2H, $OCH_2$), 3.99 (t, $^{3}J$ = 6.6 Hz, 2H, $OCH_2$), 1.99 – 1.89 (m, 2H, $CH_2$), 1.83 – 1.74 (m, 2H, $CH_2$), 1.53 – 1.19 (m, 28H, $CH_2$), 0.87 (t, $^{3}J$ = 6.6 Hz, 6H, $CH_3$). **$^{13}$C-NMR** (100 MHz, $CDCl_3$): *δ* [ppm] = 153.83, 152.40, 150.34 (C-5, tet; C-2, C-5, ph), 120.81, 113.78, 113.72 (CH-ph), 111.83 (C-1, ph), 69.79, 69.02 ($OCH_2$), 32.02, 31.98, 29.69, 29.61, 29.51, 29.45, 29.39, 26.27, 26.12, 22.80 ($CH_2$), 14.24 ($CH_3$). **IR** (ATR): $\tilde{\nu}$ [cm$^{-1}$] = 3733 w, 2952 w, 2919 s, 2847 m, 1503 s, 1462 s, 1397 w, 1291 w, 1234 vs, 1119 w, 1067 w, 1022 w, 989 w, 946 w, 880 w, 809 w, 750 w, 722 w, 671 w. **HR-ESI:** calcd. for $C_{27}H_{46}N_4O_2$ + $Na^+$: 481.3518; found: 481.3535.

**3,7,11-Tris(2,5-di(decyloxy)phenyl)tris[1,2,4]triazolo[4,3-a:4´,3´-c:4″,3″-e][1,3,5]triazine *t*-12**

According to the procedure described GP7, 0.25 g (0.55 mmol) 5-(2,5-di(decyloxyphenyl)-2H-tetrazole and 0.2 mL sym-collidine in 25 mL xylenes were stirred for 30 min. Addition of 1 mL of a 0.1 M solution of cyanuric chloride in toluene to the stirred mixture. After 1 h, 0.1 mL of this solution was added and repeated every 2 h until the reaction was complete (TLC). Addition of petroleum ether and filtration through a pad of silica, evaporation of the solvents and chromatography of the residue through a silica column with petroleum ether/ethyl acetate 20/1 yielded 108 mg (51 %) of a colorless solid with m.p. = 88 – 90 °C (POM).
**$^{1}$H-NMR** (400 MHz, $CDCl_3$): *δ* [ppm] = 7.18 (d, $^{4}J$ = 3.0 Hz, 3H, 6-H, ph), 7.12 (dd, $^{3}J$ = 9.1, $^{4}J$ = 3.0 Hz, 3H, 4-H, ph), 6.99 (d, $^{3}J$ = 9.1 Hz, 3H, 3-H, ph), 3.96 – 3.96 (m, 12H, $OCH_2$), 1.82 – 1.72 (m, 6H, $CH_2$), 1.47 – 1.40 (m, 6H, $CH_2$), 1.38 – 1.03 (m, 84H, $CH_2$), 0.91 – 0.80 (m, 18H, $CH_3$). **$^{13}$C-NMR** (100 MHz, $CDCl_3$): *δ* [ppm] = 152.95, 152.38, 147.80 (C-5, trl; C-2, C-5, ph), 140.15 (C-3, trl), 119.82 (CH-ph), 117.12 (CH-ph), 114.19 (C-1), 113.10 (CH-ph), 68.89, 68.59 ($OCH_2$), 32.03, 31.99, 29.70, 29.54, 29.46, 29.36, 29.10, 26.19, 25.76, 22.81, 22.79 ($CH_2$), 14.23 ($CH_3$). **IR** (ATR): $\tilde{\nu}$ [cm$^{-1}$] = 2919 s, 2851 m, 1600 s, 1488 s, 1468 s, 1385 w, 1275 m, 1220 vs, 1146 w, 1026 s, 879 w, 793 m, 721 m. **FD-MS:** m/z (%) = 1367,7 [M]$^+$; 2733,5 [$M_2$]$^+$. **HR-ESI:** calcd, for $C_{84}H_{135}N_9O_6$ + $H^+$: 1367,0614; found: 1367,0645

**3,4-Dibutoxybenzonitrile**

1.5 g (11.1 mmol) Protocatechunitrile gave, according to GP1 1.97 g (6.78 mmol, 84 %) of a coorless solid with m.p. : 61°C (petroleum ether). **$^{1}$H-NMR** (300 MHz, $CDCl_3$): *δ* [ppm] = 7.23 (dd, $^{3}J$ = 8.3, $^{4}J$ = 1.9 Hz, 1H, 6-H (Ph)), 7.07 (d, $^{4}J$ = 1.9 Hz, 1H, 2-H (Ph)), 6.87 (d, $^{3}J$ = 8.4 Hz, 1H, 5-H (Ph)), 4.18 – 3.8 (2 überl. t, $^{3}J$ = 6.6 Hz, 4H, $OCH_2$), 1.94 – 1.71 (m, 4H, 2-H (Alkyl)), 1.67 – 1.36 (m, 4H, 3-H (Alkyl)), 0.98 (t, $^{3}J$ = 7.4 Hz, 6H, $CH_3$). **$^{13}$C-NMR** (75 MHz, $CDCl_3$): *δ* [ppm] = 153.18 (C-4 (Ph)), 149.15 (C-3 (Ph)), 126.42 (CN), 119.57 (C-6 (Ph)), 116.11 (C-1 (Ph)), 112.80 (C-5 (Ph)), 103.59(C-2 (Ph)), 69.25, 68.91 ($OCH_2$), 31.16, 31.08, 19.27 (Alkyl), 13.94, 13.92 ($CH_3$ **IR** (ATR): $\tilde{\nu}$ [cm$^{-1}$] = 2955 m, 2932 m, 2872 m, 2219 m, 1596 m, 1514 s, 1420 m, 1395 m, 1269 s, 1242 s, 1136 ss, 988 m, 859 m, 809 m. **ESI-MS:** *m/z* (%) = 246.9 (1), 247.2 (6), 247.7 (3), 248.2 (77), 248.5 (1) [M+H]$^+$.

**5-(3,4-Dibutoxyphenyl)tetrazole**

3,4-Dibutoxybenzonitrile (2.00 g, 8.01 mmol), $NaN_3$ (28.3 mmol, 1.84 g), and $NEt_3HCl$ (28.3 mmol, 3.90 g) in 50 mL toluene were stirred for 5 d under reflux. The cooled mixture was diluted with water (100 mL), acidulated to pH 2, the separated organic phase was washed with water, concentrated, and the residue was recrystallized from ethanol first followed by from ethyl acetate to yield 1.97 g (84 %) of a colorless solid with m.p. = 185 °C.
$^{1}$H-NMR (300 MHz, $CDCl_3$): δ / ppm = 7.55 (d, 4J = 2.1 Hz, 1H, 2-H), 7.51 (dd, $^{3}$J = 8.3, $^{4}$J = 2.1 Hz, 1H, 6-H), 6.84 (d, $^{3}$J = 8.4 Hz, 1H, 5-H), 3.95 ($2^x$ t, $^{3}$J = 6.7 Hz, 4H, OCH2), 1.71 (m, 4H, 2-H, 6-H (ph')), 1.53 – 1.28 (m, 4H, 3'-H, 7'-H), 0.99 – 0.76 (m, 6H, CH3). $^{13}$C-NMR (75 MHz, $CDCl_3$): δ / ppm = 155.24 (C-5 (tet)), 151.02 (C-4 (ph)), 148.93 (C-3 (ph)), 119.93 (C-6 (ph)), 116.21 (C-1 (ph)), 112.78 (C-5 (ph)), 111.80 (C-2 (ph)), 68.58, 68.38 ($OCH_2$), 40.08, 39.80, 39.52, 39.24, 38.96, 30.75, 30.68, 18.77, 18.74, (CH2), 13.45 ($CH_3$). IR (ATR): $\tilde{\nu}$ / cm$^{-1}$ = 2925 m, 2855 m, 1605 m, 1508 s, 1459 m, 1266 s, 1233 s, 1136 m, 1042 m, 873 m, 815

m. HRMS-ES(+): $[M+H]^+$ calcd.: 313.1640, found: 313.1642.

**3,7,11-Tris{3,4-butoxyphenyl}tris([1,2,4]triazolo)[4,3-a:4',3'-c:4'',3''-e][1,3,5]triazine *t*-13**
(1.20 mmol, 350 mg) 5-(3,4-Dibutoxyphenyl)tetrazole and (146 mg, 1.20 mmol, 160 µL) 2,4,6-collidine in 30 mL dry toluene were stirred for 1 h and cyanuric chloride (298 µmol, 55 mg)in toluene (c = 10 mg $mL^{-1}$) added and stirring was continued over night. After 24 h and 48 h, additional portions of $C_3N_3Cl_3$ (each: 10 mg) in toluene were added. Stirring for 16 h, followed by aqueous work-up and chromatography on silica gel with a head of basic alumina and toluene ethyl acetate (25/1) as eluent yielded 200 mg (232 µmol, 58 %) of a colorless solid with m.p. = 131 °C. **$^1$H-NMR** (300 MHz, $CDCl_3$): δ / ppm = 7.81 (dd, 3J = 8.4, 2.0 Hz, 1H, 6-H (ph)), 7.70 (d, *J* = 2.1 Hz, 1H, 2-H (ph)), 7.01 (d, $^3J$ = 8.5 Hz, 1H, 5-H (ph)), 4.10 (t, $^3J$ = 6.5 Hz, 4H, $OCH_2$), 1.96 – 1.73 (m, 4H, C-2, C-6 ($CH_2$)), 1.63 – 1.39 (m, 4H, C-3, C-7 ($CH_2$)), 1.11 – 0.84 (m, 6H, $CH_3$). **$^{13}$C-NMR** (75 MHz, $CDCl_3$): δ / ppm = 152.36 (C-5 (Triaz)), 151.14 (C-4 (ph)), 148.82 (C-3 (ph)), 140.61 (C-3 (Triaz)), 123.94 (C-6 (ph)), 115.93 (C-1 (ph)), 115.16 (C-5 (ph)), 112.57 (C-2 (ph)), 69.27 ($OCH_2$), 68.89 ($OCH_2$), 31.34, 31.24, 24.51, 19.36 ($CH_2$), 14.03, 14.00 ($CH_3$). **IR** (ATR): $\tilde{\nu}$ / cm-1 = 2956 s, 2871 m, 1598 s, 1491 s, 1261 ss, 1226 s, 1143 m, 1010 w, 802 w. **FD-MS**: m/z (%) = 430.7 (8), 431,3 (4) $[M]^{2+}$, 859.5 (1), 861.5 (100), 862.6 (58), 863.6 (15), 864.6 (4)$[M]^+$. **EA**: $C_{48}H_{63}N_9O_6$ (862.07) calcd.: 66.88% C, 7.37% H, 14.62% N, found: 66.80% C, 7.22% H, 14.63% N.

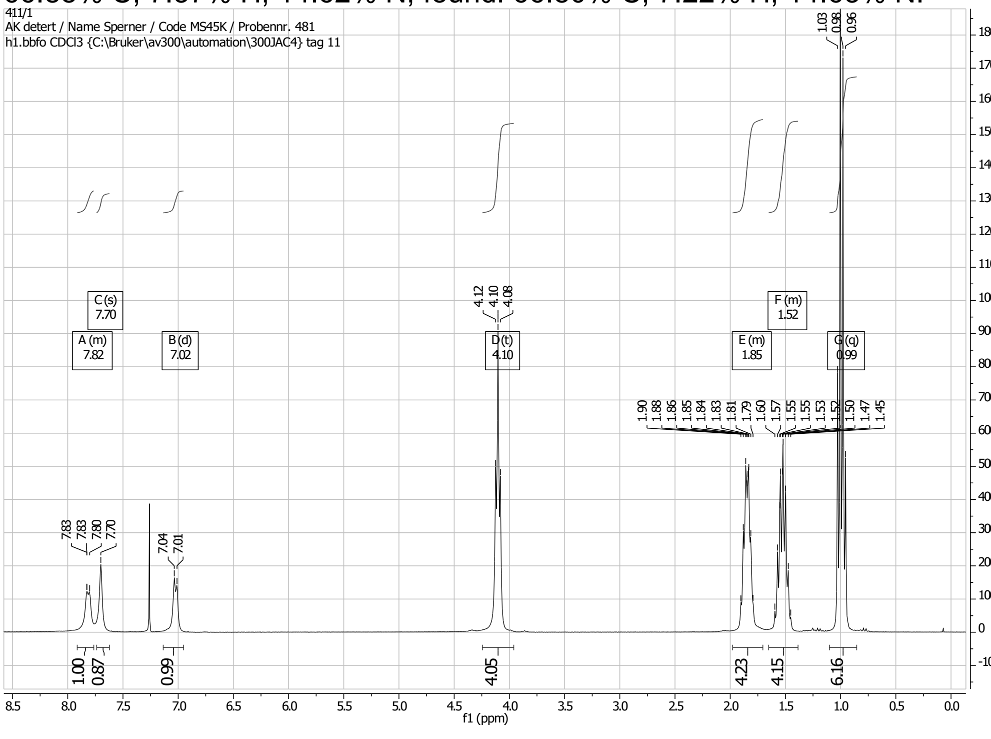


$^1$H-NMR of ***t*-13**

**3,4-Dipentoxybenzonitrile**
According to GP1 1.0 g (7.4 mmol) 4-Cyanocatechol in 60 mL *N,N*-dimethylformamide, 4.5 g (33 mmol) $K_2CO_3$ and 2.02 mL (2.46 g, 16.3 mmol.) 1-bromopentane gave 2.04 g (6.46 mmol, 87 %) of a colorless solid with m.p. = 39 °C. **$^1$H-NMR** (300 MHz, $CDCl_3$): *δ* / ppm = 7.23 (dd, $^3J$ = 8.3, $^4J$ = 1.9 Hz, 1H, 6-H (Ph)), 7.07 (d, $^4J$ = 1.9 Hz, 1H, 2-H (Ph)), 6.86 (d, $^3J$ = 8.4 Hz, 1H, 5-H (Ph)), 4.10 – 3.91 (zwei überl. t, $^3J$ = 6.4 Hz, 4H, $OCH_2$), 1.94 – 1.76 (m, 4H, $OCH_2CH_2$), 1.57 – 1.29 (m, 8H, Alkyl), 1.05 – 0.84 (m, 6H, $CH_3$). **$^{13}$C-NMR** (75 MHz, $CDCl_3$): *δ* / ppm = 153.17 (C-4 (Ph)), 149.15 (C-3 (Ph)), 126.43 (CN), 119.58 (C-6 (Ph)), 116.07 (C-1 (Ph)), 112.78 (C-5 (Ph)), 103.60 111.76 (C-2 (Ph)), 69.53, 69.21 ($OCH_2$), 28.83, 28.76, 28.24, 22.54 (Alkyl), 14.15 ($CH_3$). **IR** (ATR): $\tilde{\nu}$ / $cm^{-1}$ = 2931 s, 2871 m, 2219 m, 1596 m, 1514 ss, 1467 m, 1269 ss, 1136 s, 1048 m, 992 s, 855 w, 808 m. **ESI-MS:** *m/z* (%) = 275.2 (10), 275.7 (4), 276.2 (100), 276.5 (3), 277.2 (29) $[M+H]^+$.

**5-(3,4-Dipentoxyphenyl)tetrazole**

3,4-Dipentoxybenzonitrile (5.27 mmol, 1.45 g), $NaN_3$ (18.4 mmol, 1.20 g), and $NEt_3HCl$ (18.4 mmol, 2.54g) in 50 mL toluene were stirred for 5 d under reflux. The cooled mixture was diluted with water (100 mL), acidulated to pH 2, the separated organic phase was washed with water, concentrated, and the residue was recrystallized from ethanol to yield 1.33 g (79 %) of a colorless solid with m.p. = 154 °C. **$^1$H-NMR** (300 MHz, $CDCl_3$, 1 drop DMSO-$d_6$): $\delta$ / ppm = 7.56 (d, $^4J$ = 2.0 Hz, 1H, 2-H (Ph)), 7.51 (dd, $^3J$ = 8.4, $^4J$ = 2.1 Hz, 1H, 6-H (Ph)), 6.85 (d, $^3J$ = 8.4 Hz, 1H, 5-H), 3.96 (zwei t, überl. $^3J$ = 6.6 Hz, 4H, $OCH_2$), 1.73 (q, $^3J$ = 6.9 Hz, 4H, 2'-H, 7'-H), 1.48 – 1.16 (m, 8H, 3'-H, 4'-H, 8'-H, 9'-H), 0.93 – 0.74 (m, 6H, $CH_3$). **$^{13}$C-NMR** (75 MHz, $CDCl_3$, 1 Tropfen DMSO-$d_6$): $\delta$ / ppm = 155.45 (C-5 (Tetrazol)), 150.96 (C-4 (Ph)), 148.91 (C-3 (Ph)), 119.88 (C-6 (Ph)), 116.38 (C-1 (Ph)), 112.75 (C-5 (Ph)), 111.76 (C-2 (Ph)), 68.86, 68.67 ($OCH_2$),39.80, 39.52, 39.24, 28.42, 28.36, 27.75, 27.72, 22.01 (Alkyl), 14.11 ($CH_3$). **IR** (ATR): $\tilde{\nu}$ / $cm^{-1}$ = 2929 m, 2854 m, 1657 m, 1509 s, 1458 m, 1267 m, 826 w. **HRMS-ES(+)**: $[M+H]^+$ calcd.: 341.1953, found: 341.1949.

**3,7,11-Tris{3,4-pentoxyphenyl}tris([1,2,4]triazolo)[4,3-a:4',3'-c:4'',3''-e][1,3,5]triazine *t*-14**
(942 µmol, 300 mg) 5-(3,4-Dipetoxyphenyl)tetrazole and (118 mg, 942 µmol, 130 µL) 2,4,6-collidine in 30 mL dry toluene were stirred for 1 h and cyanuric chloride (217 µmol, 40 mg) in toluene (c = 10 mg $mL^{-1}$) added and stirring was continued over night. After 24 h and 48 h, additional portions of $C_3N_3Cl_3$ (each: 10 mg) in toluene were added. Stirring for 16 h, followed by aqueous work-up and chromatography on silica gel with a head of basic alumina and toluene ethyl acetate (20/1) as eluent yielded 226 mg (239 µmol, 76 %) of a colorless solid with m.p. = 102 °C, clearing point 201 °C.

**$^1$H-NMR** (300 MHz, $CDCl_3$): $\delta$ / ppm = 7.82 (dd, $^3J$ = 8.4, $^4J$ = 2.1 Hz, 1H, 6-H (ph)), 7.70 (d, $^4J$ = 2.1 Hz, 1H, 2-H (ph)), 7.03 (d, $^3J$ = 8.6 Hz, 1H, 5-H (ph)), 4.10 (t, $^3J$ = 6.6 Hz, 4H, $OCH_2$), 1.97 – 1.73 (m, 4H, C-2, C-7 ($CH_2$)), 1.56 – 1.31 (m, 8H, C-3, C-4, C-8, C-9 ($CH_2$)), 0.94 (q, $^3J$ = 7.1 Hz, 6H, $CH_3$). **$^{13}$C-NMR** (75 MHz, $CDCl_3$): $\delta$ / ppm = 152.37 (C-5 (Triaz)), 151.16 (C-4 (ph)), 148.83 (C-3 (ph)), 140.62 (triazine-C3), 123.91 (C-6 (ph)), 115.95 (C-1 (ph)), 115.13 (C-5 (ph)), 112.57 (C-2 (ph)), 69.25 ($OCH_2$), 68.90 ($OCH_2$), 31.34, 31.25, 19.37 ($CH_2$), 14.03, 14.00 ($CH_3$). **IR** (ATR): $\tilde{\nu}$ / $cm^{-1}$ = 2932 s, 2865 m, 1600 s, 1491 s, 1468 s, 1264 ss, 1226 m, 1144 m, 1014 w, 881 m. **FD-MS:** *m/z* (%) = 473.0 (58), 473.9 (7) $[M]^{2+}$, 945.6 (100), 946.6 (71), 947.5 (7), 948.7 (2) $[M]^+$. **EA:** calcd. for $C_{54}H_{75}N_9O_6$ (946.23): 68.54% C, 7.99% H, 13.32% N, found: 68.29% C, 7.82% H, 13.42% N.

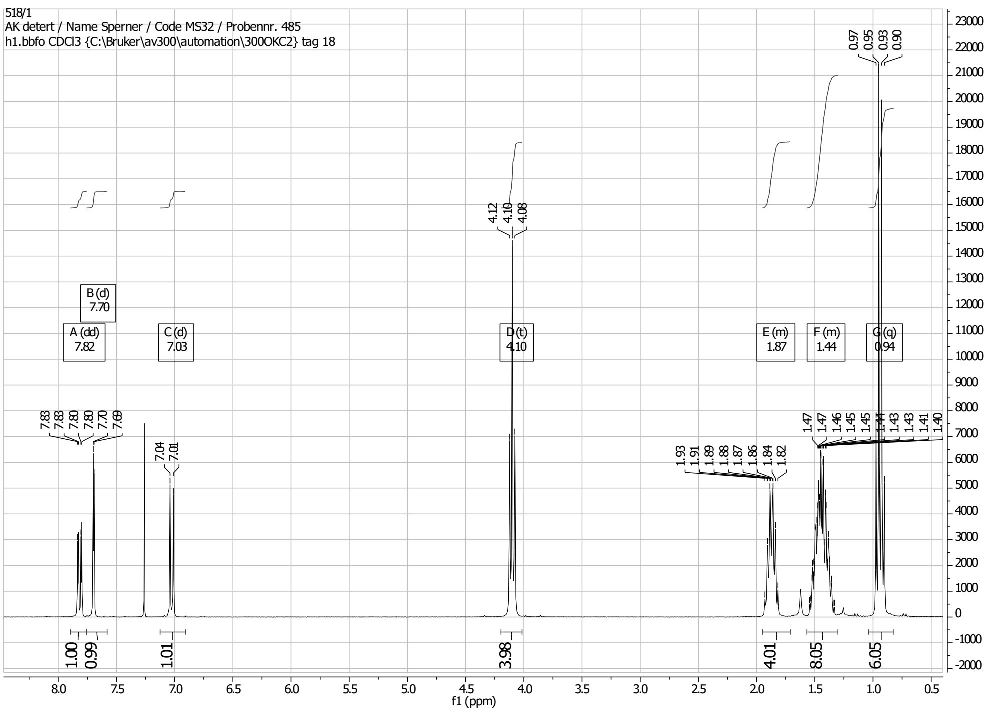

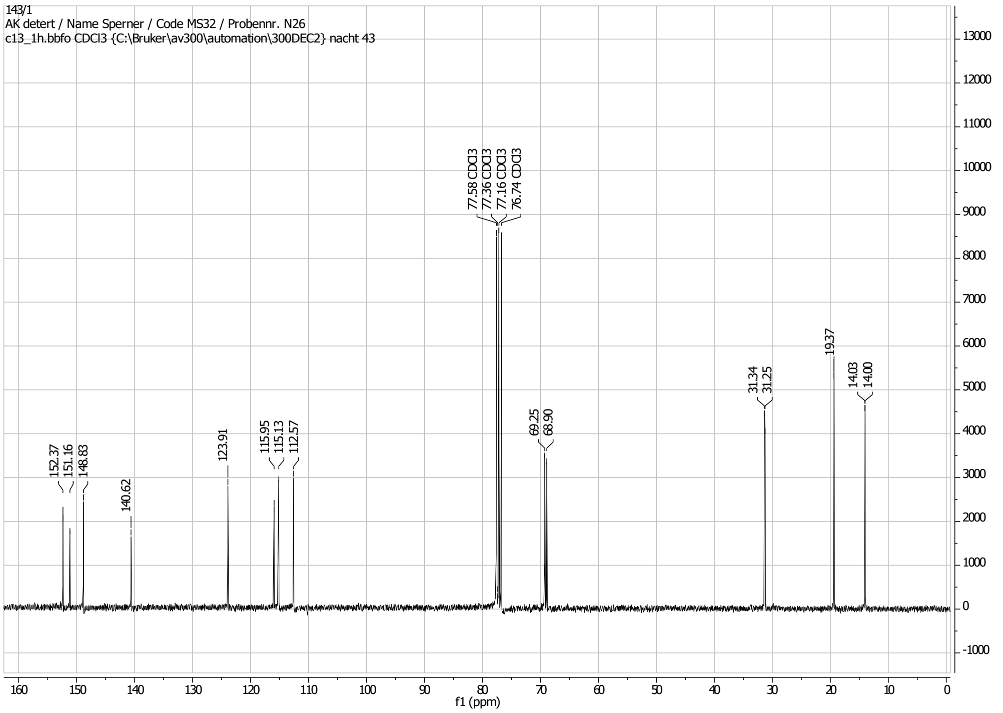


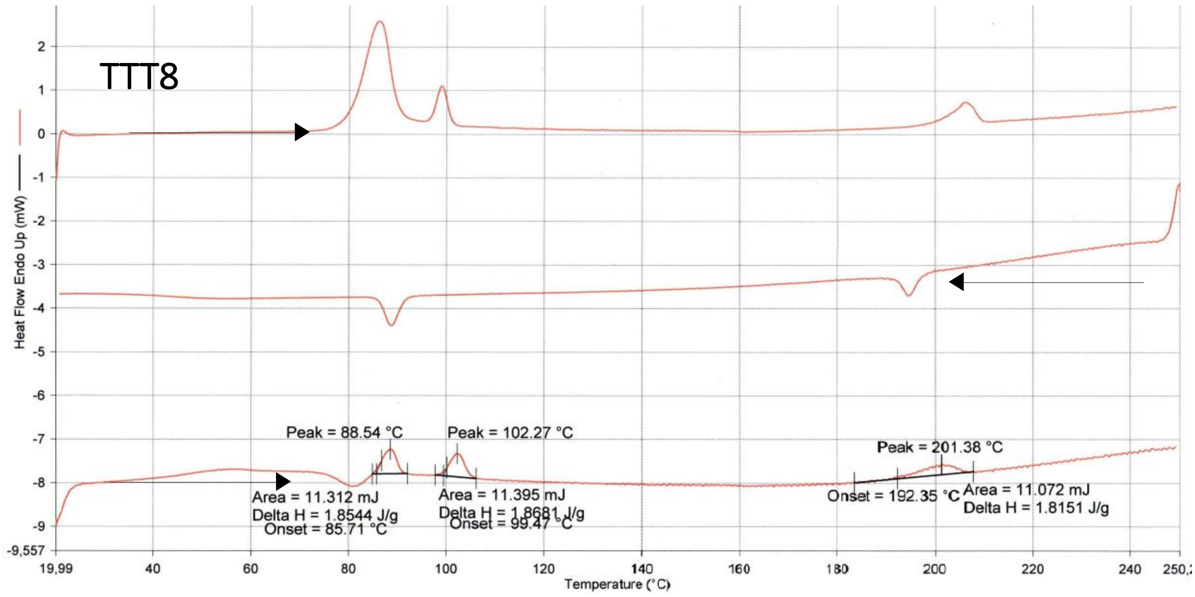


NMR spectra and DSC of ***t*-14**

**2,6,10-Tris(3,4-di(hexyloxy)phenyl)tris([1,2,4]triazolo)[1,5-a:1',5'-c:1'',5''-e][1,3,5]triazine *t*-15**

95 mg (0,09 mmol) 3,7,11-Tris(3,4-di(hexyloxy)phenyl)tris([1,2,4]triazolo)[4,3-a:4',3'-c:4'',3''-e]-[1,3,5]triazine in 2 mL octadecane were heated for 64 h at 280 °C. The product was precipitated by addition of petroleum ether and was recrystallized from this solvent to yield - 50 mg (0,05 mmol, 55 %) of a colorless solid with m.p. = 145 – 147 °C (petroleum ether). **$^1$H-NMR** (400 MHz, $CDCl_3$): $\delta$ [ppm] = 7.98 (dd, $^3J$ = 8.4, $^4J$ = 1.9 Hz, 1H, 6-H, ph), 7.86 (d, $^4J$ = 1.9 Hz, 1H, 2-H, ph), 6.93 (d, $^3J$ = 8.4 Hz, 1H, 5-H, ph), 4.11 (t, $^3J$ = 6.6 Hz, 2H, $OCH_2$), 4.04 (t, $^3J$ = 6.6 Hz, 2H, $OCH_2$), 1.91 – 1.77 (m, 4H, $CH_2$), 1.57 – 1.28 (m, 12H, $CH_2$), 0.92 (m, 6H, $CH_3$). **$^{13}$C-NMR** (100 MHz, $CDCl_3$): $\delta$ [ppm] = 165.19 (C-5, trl), 152.21 (C-4, ph), 149.28 (C-3, ph), 144.50 (C-3, trl), 121.64 (C-6, ph), 120.73 (C-1, ph), 112.76 (C-5,ph), 112.43 (C-2, ph), 69.66, 69.21 ($OCH_2$), 31.78, 31.75, 29.42, 29.29, 25.86, 25.83, 22.79, 22.77 ($CH_2$), 14.20, 14.18 ($CH_3$). **IR** (ATR): $\tilde{\upsilon}$ [cm$^{-1}$] = 2927 m, 2859 m, 1630 s, 1604 s, 1534

w, 1462 s, 1391 m, 1341 w, 1308 m, 1262 s, 1221 s, 1134 s, 1065 w, 975 w, 938 w, 863 m, 804 w, 746 s, 726 m, 659 w. **HR-ESI:** calcd. for $C_{60}H_{87}N_9O_6 + H^+$: 1030.6858; found: 1030.6895.

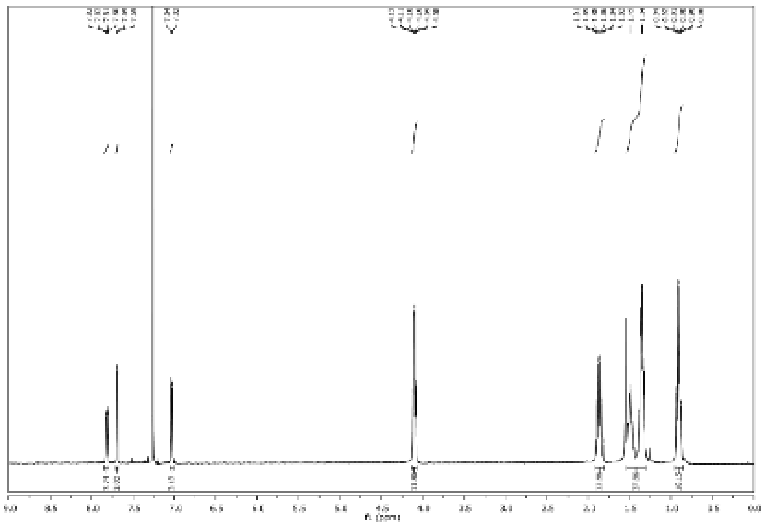

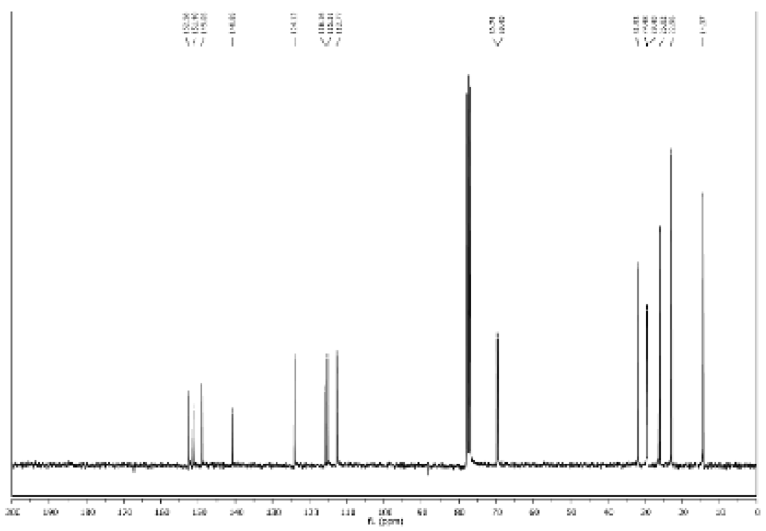

$^{1}$H-NMR and $^{13}$C-NMR of ***t*-15**

**3,4-Di(heptyloxy)benzonitrile**

2.10 g (15 mmol, 1 eq.) 3,4-Dihydroxybenzonitrile, 6.14 g (44 mmol, 3 eq.) $K_2CO_3$, and 5.83 g (33 mmol, 2.2 eq.) 1-bromoheptane were stirred in 60 mL DMF ($N_2$, 80 °C). After 18 h, additional 2.69 g (15 mmol, 1eq.) 1-bromoheptane, 3.07 g (15 mmol, 1eq.) $K_2CO_3$ were added and stirred for 60 h at 80 °C. The mixture was acidulated (2 N HCl), extracted (petroleum ether, 3 x 50 mL) and the pooled organic solutions were washed with brine , dried ($MgSO_4$) and recrystallized from petroleum ether to yield 3.70 g (10 mmol, 75 %) of a colorless solid with m.p. = 68 – 69 °C. **$^{1}$H-NMR** (400 MHz, $CDCl_3$): $\delta$ [ppm] = 7.23 (dd, $^{3}J$ = 8.3 Hz, $^{4}J$ = 1.9 Hz, 1H, 6-H, ph), 7.07 (d, $^{4}J$ = 1.9 Hz, 1H, 2-H, ph), 6.86 (d, $^{3}J$ = 8.3 Hz, 1H, 5-H, ph), 4.00 (m, 4H, $OCH_2$), 1.90 – 1.76 (m, 4H, $CH_2$), 1.50 – 1.26 (m, 16H, $CH_2$), 0.89 (t, $^{3}J$ = 6.8 Hz, 6H, $CH_3$). **$^{13}$C-NMR** (75 MHz, $CDCl_3$): $\delta$ [ppm] = 153.21, 149.18 (C-3, C-4), 126.44 (CH-ph), 119.61 (C-1), 116.12, 112.82 (CH-ph), 103.61 (C-7), 69.56, 69.23 ($OCH_2$), 31.90, 29.15, 29.08, 26.02, 22.72 ($CH_2$), 14.22 ($CH_3$). **IR** (ATR): $\tilde{\upsilon}$ [cm$^{-1}$] = 2957 s, 2930 s, 2852 s, 2220 s, 1596 s, 1517 s, 1468 s, 1421 s, 1334 m, 1276 vs, 1139 vs, 1005 s, 910 s, 855 m, 810 s, 734 s. **FD-MS:** m/z: 332,8 [$M^+$]; 664,1 [$M_2^+$].

**5-(3,4-Di(heptyloxy)phenyl)-2*H*-tetrazole**

According to the general procedure, 2.00 g (6.00 mmol) 3,4-di(heptyloxy)benzonitrile, 1.39 g (21,00 mmol) $NaN_3$, 2.91 g (21.00 mmol) triethylammonium chloride in 40 mL toluene, followed after 18 h by additional 0.40 g (6 mmol) $NaN_3$, 0.80 g (6 mmol) triethylammonium chloride. After further 24 h, 100 mL 2N HCl was added. Usual work-up yielded 1,94 g (5,20 mmol, 86 %) of a colorless solid with m.p. 186 – 187 °C (Ethanol). **$^1$H-NMR** (400 MHz, DMSO-d$_6$): $\delta$ [ppm] = 7.59 – 7.57 (m, 2H, 2-H, 6-H, ph), 7.13 (d, $^3J$ = 8.8 Hz, 1H, 5-H, ph), 4.02 (t, $^3J$ = 6.1 Hz, 4H, $OCH_2$), 1.78 – 1.69 (m, 4H, $CH_2$), 1.48 – 1.21 (m, 12H, $CH_2$), 0.86 (t, 6H, $CH_3$). **$^{13}$C-NMR** (75 MHz, DMSO-d$_6$): $\delta$ [ppm] = 154.82 (C-5, tet), 150.99, 148.77 (C-3, C-4, ph), 120.19 (C-6, ph), 116.15 (C-1, ph), 113.55, 111.78 (C-2, C-5, ph), 68.55, 68.32 ($OCH_2$), 31.32, 28.73, 28.66, 28.48, 25.51, 22.06, 22.06 ($CH_2$), 13.94 ($CH_3$). **IR** (ATR): $\tilde{\nu}$ [cm$^{-1}$] = 2918 m, 2849 m, 1602 m, 1509 vs, 1267 vs, 1132 s, 1005 s, 745 s. **HR-ESI:** calcd. for $C_{21}H_{34}N_4O_2 + H^+$: 375.2760; found: 375.2771.

**3,7,11-Tris(3,4-di(heptyloxy)phenyl)tris([1,2,4]triazolo)[4,3-a:4',3'-c:4'',3''e][1,3,5]triazine *t*-16**

According to the general procedure, 1.00 g (2.67 mmol) 5-(3,4-di(heptyloxy)phenyl)-2*H*-tetrazole, 0.15 g (0.80 mmol) cyanuric chloride in 25 mL dry xylenes and 0,40 mL *sym*-collidine were heated slowly to 60 °C and stirred for 4 h. 15 mL 2N was added, the aqueous phase extracted and all pooled organic layers washed with brine, dried, concentrated, and absorbed on silica gel (0.5g) and purified on a column of basic alumina (toluene followed by toluene/ ethyl acetate 40:1) to yield 0.67 g (0.60 mmol, 74 %) of a colorless solid with m.p. = 103 °C (DSC, 2. heating). **$^1$H-NMR** (400 MHz, $CDCl_3$): $\delta$ [ppm] = 7.81 (dd, $^3J$ = 8.5 Hz, $^4J$ = 2.1 Hz, 3H, 6-H, ph), 7.69 (d, $^4J$ = 2.1 Hz, 3H, 2-H, ph), 7.02 (d, $^3J$ = 8.5 Hz, 3H, 5-H, ph), 4.09 (t, $^3J$ = 6.4 Hz, 12H, $OCH_2$), 1.91 – 1.82 (m, 12H, $CH_2$), 1.53 – 1.26 (m, 48H, $CH_2$), 0.92 – 0.86 (m, 18H, $CH_3$). **$^{13}$C-NMR** (100 MHz, $CDCl_3$): $\delta$ [ppm] = 152.37, 151.19 (C-4, ph; C-5, trl), 148.84 (C-3, ph), 140.64 (C-3, trl), 123.91 (C-6, ph), 115.95 (C-1, ph), 115.10 (C-5, ph), 112.56 (C-2, ph), 69.53, 69.19 ($OCH_2$), 31.96, 29.33, 29.24, 29.20, 26.12, 26.10, 22.76 ($CH_2$), 14.24 ($CH_3$). **IR** (ATR): $\tilde{\nu}$ [cm$^{-1}$] = 3100 w, 2924 s, 2855 s, 1596 s, 1490 s, 1467 s, 1329 w, 1260 vs, 1227 m, 1140 m, 1008 w, 881 w, 805 w, 714 m. **FD-MS:** m/z (%): 557,3 (26) [M$^{2+}$]; 1114,1 (100) [M$^+$]. **HR-ESI:** calcd. for $C_{66}H_{99}N_9O_6 + H^+$: 1114.7797; found: 1114.7766.

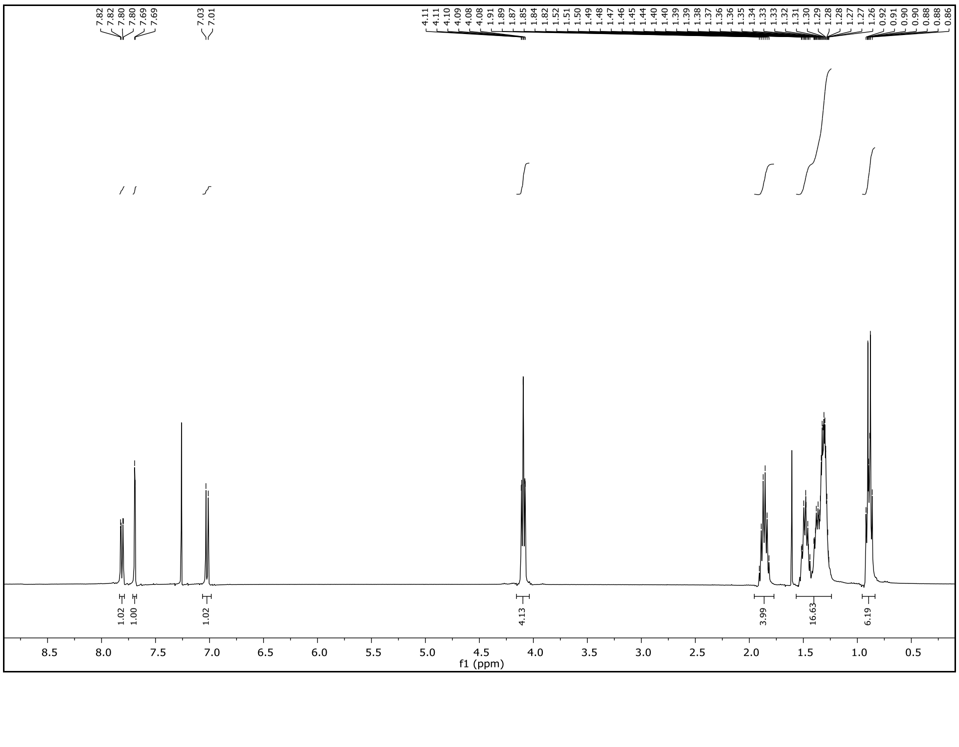

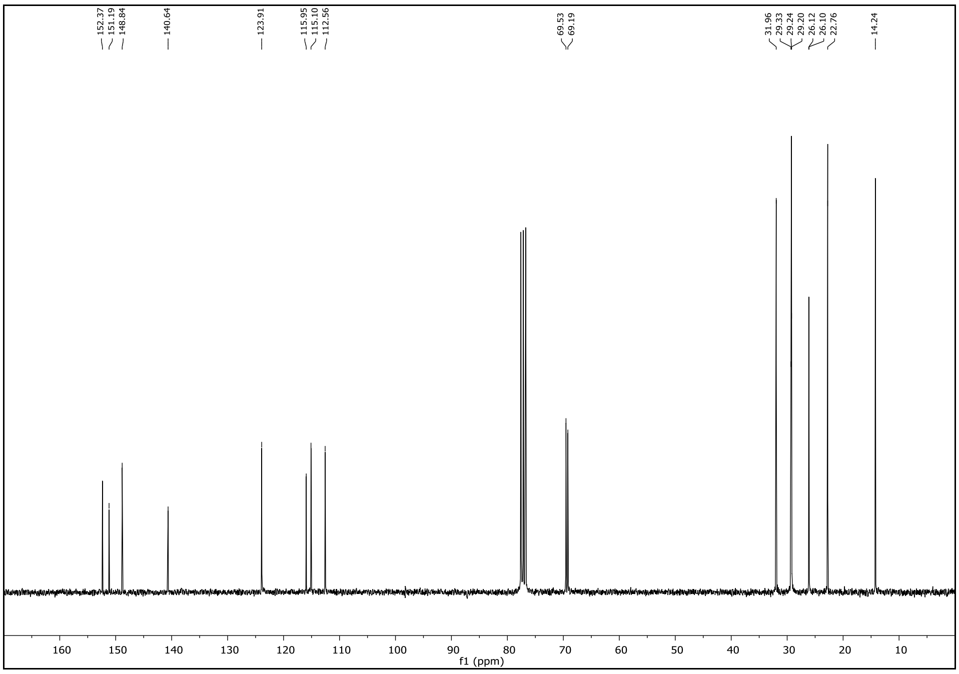


$^1$H-NMR and $^{13}$C-NMR of ***t*-16**

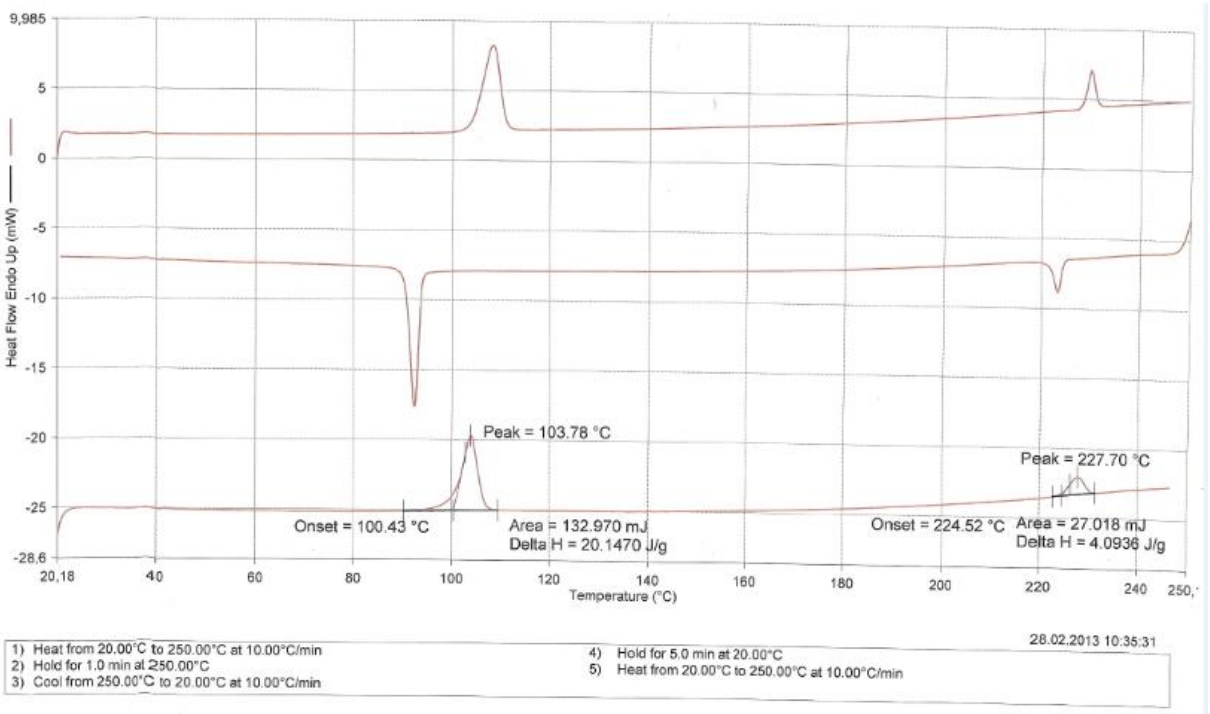


DSC of ***t*-16**

**3,4-Di(octyloxy)benzonitrile**

According to the general procedure GP1, 2.70 g (20 mmol) 3,4-dihydroxybenzonitrile gave 6,90 g (20 mol, 87 %) of a colorless solid with m.p. = 73 – 75 °C (petroleum ether). **$^1$H-NMR** (400 MHz, $CDCl_3$): $\delta$ [ppm] = 7.23 (dd, $^3J$ = 8.3 Hz, $^4J$ = 1.9 Hz, 1H, 6-H, ph), 7.07 (d, $^4J$ = 1.9 Hz, 1H, 2-H, ph), 6.86 (d, $^3J$ = 8.3 Hz, 1H, 5-H, ph), 4.06 – 3.99 (m, 4H, $OCH_2$), 1.88 – 1.77 (m, 4H, $CH_2$), 1.51 – 1.22 (m, 20H, $CH_2$), 0.91 – 0.86 (m, 6H, $CH_3$). **$^{13}$C-NMR** (100 MHz, $CDCl_3$): $\delta$ [ppm] = 153.20, 149.17 (C-3, C-4), 126.43 (CH-ph), 119.59 (C-1), 116.13, 112.82 (CH-ph), 103.61 (C-7), 69.56, 69.22 ($OCH_2$), 31.92, 29.42, 29.36, 29.14, 29.07, 26.06, 22.78 ($CH_2$), 14.22 ($CH_3$). **IR** (ATR): $\tilde{\nu}$ [cm$^{-1}$] = 2921 s, 2850 s, 2220 m, 1597 m, 1515 s, 1468 s,

1421 m, 1270 s, 1243 s, 1137 s, 907 s, 855 m, 810 m, 731 vs. **FD-MS:** m/z (%): 359,7 (100) [$M^+$]; 719,0 (2) [$M_2^+$].

**5-(3,4-Di(octyloxy)phenyl)-2*H*-tetrazole**
According to the general procedure GP5, 3.00 g (8.3 mmol) 3,4-di(octyloxy)benzonitrile, 1.88 g (29.0 mmol) $NaN_3$ and 4.02 g (29.0 mmol) triethylammonium chloride in 50 mL toluene, after 36 h additional 0.50 g (8.3 mmol) $NaN_3$ and 1.00 g (8.3 mmol) triethylammonium chloride gave 2.40 g (6.00 mmol, 71 %) of a colorless solid with m.p. =173 – 174 °C (Ethanol). **$^1$H-NMR** (400 MHz, DMSO-$d_6$/$CDCl_3$): $\delta$ [ppm] = 7.61 – 7.44 (m, 2H, 2-H, 6-H, ph), 6.92 (d, $^3J$ = 8.9 Hz, 1H, 5-H, ph), 4.00 – 3.96 (m, 4H, $OCH_2$), 1.82 – 1.65 (m, 4H, $CH_2$), 1.45 – 1.37 (m, 4H, $CH_2$), 1.31 – 1.17 (m, 16H, $CH_2$), 0.82 – 0.79 (m, 6H, $CH_3$). **$^{13}$C-NMR** (75 MHz, DMSO-$d_6$/$CDCl_3$): $\delta$ [ppm] = 150.87, 148.82 (C-3, C-4, ph), 119.76 (CH-ph), 116.30 (C-1, ph), 112.67, 111.69 (CH-ph), 68.75, 68.57 ($OCH_2$), 31.24, 28.78, 28.70, 28.61, 25.43, 22.09 ($CH_2$), 13.54 ($CH_3$). **IR (ATR):** $\tilde{\nu}$ [$cm^{-1}$] = 3733 w, 3624 w, 3071 w, 2921 s, 2849 m, 1606 m, 1509 vs, 1464 s, 1268 vs, 1235 vs, 1133 s, 1061 m, 873 s, 812 s, 745 s. **HR-ESI:** calcd. for $C_{23}H_{38}N_4O_2 + H^+$: 403.3073; found: 403.3061.

**3,7,11-Tris(3,4-di(octyloxy)phenyl)tris([1,2,4]triazolo)[4,3-a:4',3'-c:4'',3''-e][1,3,5]triazine *t*-17**
According to the general procedure, 1.00 g (2.48 mmol) 5-(3,4-di(octyloxy)phenyl)-2*H*-tetrazole and 0.14 g (0.75 mmol) cyanuric chloride in 25 mL xylenes with 0.40 mL sym-collidine were heated to 60 °C for 4 h. Dilution with water and Extraction with ethyl acetate gave the crude product that was absorbed on 0,5 g silica gel. Chromatography on basic alumina (toluene, followed by toluene/ethyl acetate 40/1) gave 0.71 g (0.6 mmol, 79 %) of a colorless solid with m.p. = 99 °C (DSC, 2. heating).
**$^1$H-NMR** (400 MHz, $CDCl_3$): $\delta$ [ppm] = 7.81 (dd, $^3J$ = 8.6 Hz $^4J$ = 2.1 Hz, 3H, 6-H, ph), 7.69 (d, $^4J$ = 2.1 Hz, 3H, 2-H, ph), 7.03 (d, $^3J$ = 8.6 Hz, 3H, 5-H, ph), 4.11 – 4.08 (m, 12H, $OCH_2$), 1.91 – 1.82 (m, 12H, $CH_2$), 1.54 – 1.24 (m, 60H, $CH_2$), 0.89 – 0.85 (m, 18H, $CH_3$). **$^{13}$C-NMR** (100 MHz, $CDCl_3$): $\delta$ [ppm] = 152.38, 151.20 (C-4, ph; C-5, trl), 148.85 (C-3, ph), 140.64 (C-3, trl), 123.92 (C-6, ph), 115.95 (C-1, ph), 115.12 (C-5, ph), 112.58 (C-2, ph), 69.55, 69.21 ($OCH_2$), 31.97, 29.53, 29.50, 29.43, 29.32, 29.23, 26.18, 26.15, 22.82 ($CH_2$), 14.25 ($CH_3$). **IR** (ATR): $\tilde{\nu}$ [$cm^{-1}$] = 3100 w, 2923 vs, 2854 s, 1597 s, 1492 s, 1468 s, 1262 vs, 1226 s, 1142 s, 1023 m, 804 m, 714 m. **HR-ESI:** calcd. for $C_{72}H_{111}N_9O_6 + H^+$: 1198.8736; found: 1198.9738.

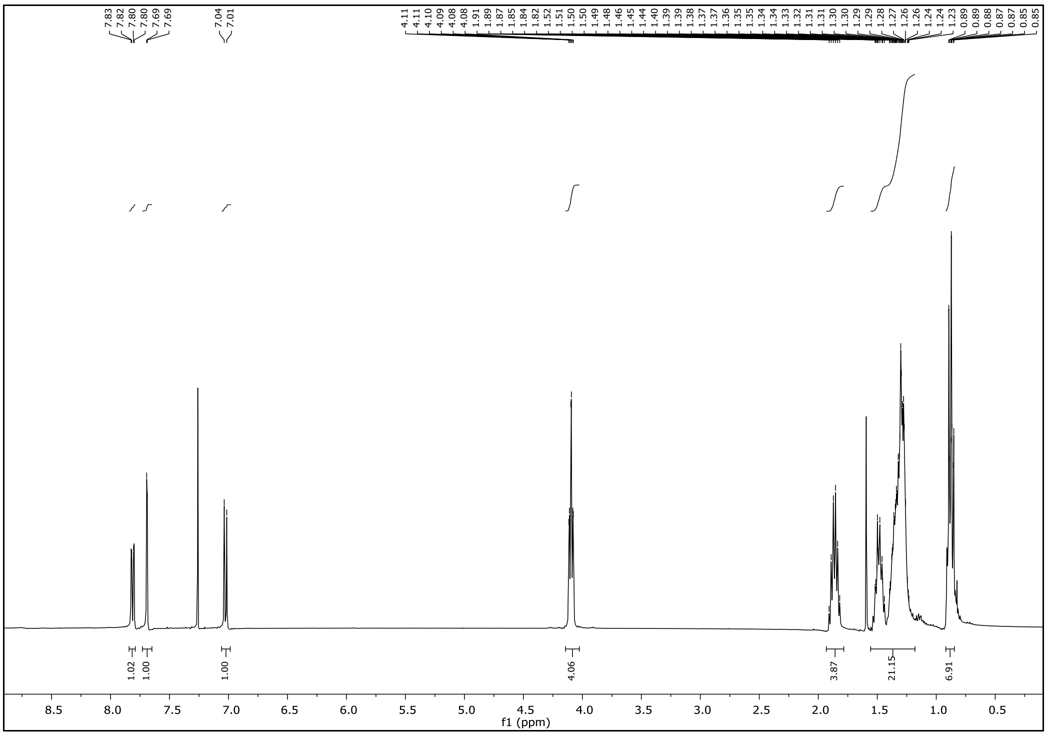

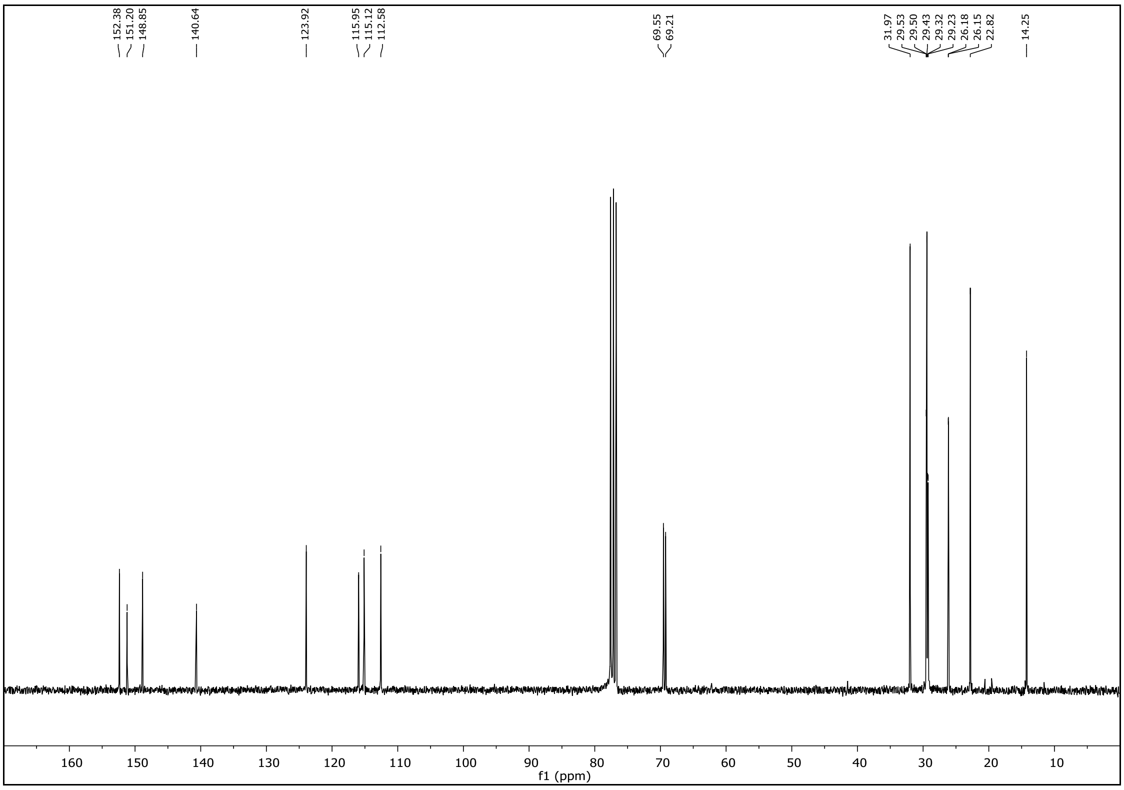


$^{1}$H-NMR and $^{13}$C-NMR of ***t*-17**

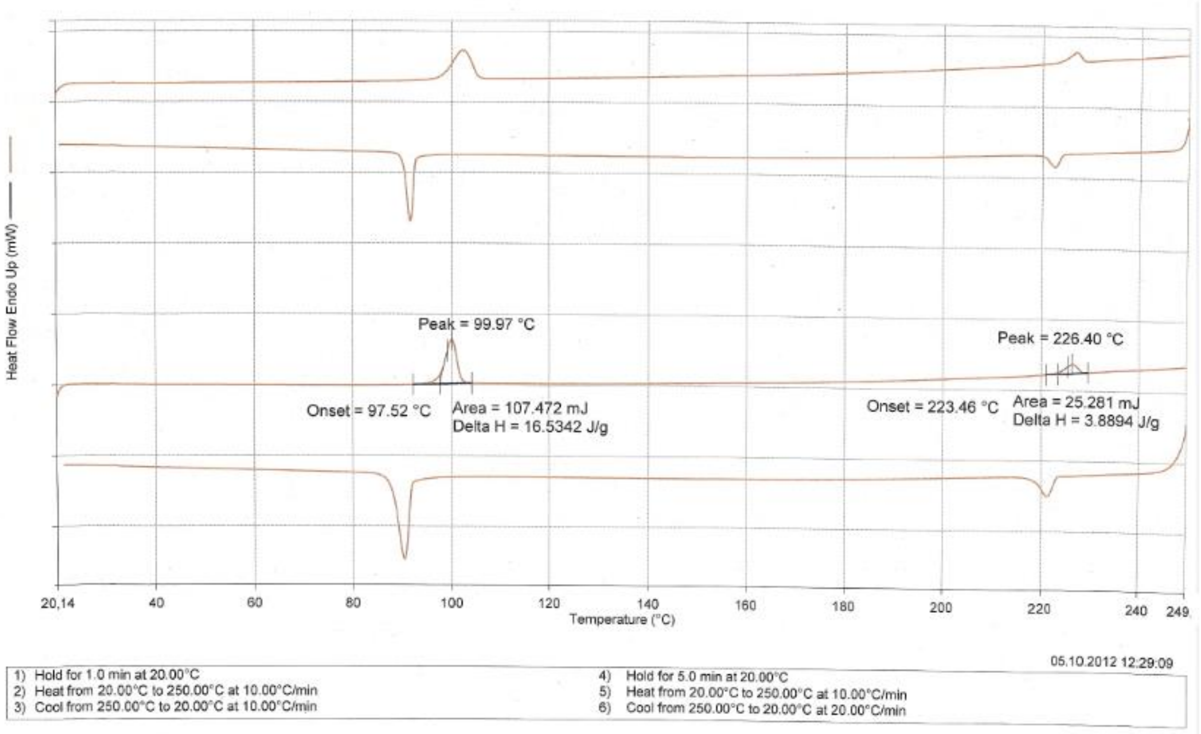


DSC of ***t*-17**

**3,4-Di(nonyloxy)benzonitrile**

According to the general procedure, 2.70 g (19 mmol) 3,4-dihydroxybenzonitrile, 7.88 g (57 mmol) potash, 8,94 g (43 mmol) 1-bromononane in 100 mL DMF under nitrogen for 36 h stirred at 80 °C, additional 4.30 g (21 mmol) 1-bromononane, 2.60 g (19 mmol) potash and 6 h stirring at 80 °C. Acidulated with 2 N HCl, extraction with light petroleum, brine and $MgSO_4$

gave, after recrystallization from petroleum ether, 5.75 g (15 mmol, 78 %) of a colorless solid with m.p. = 76 – 77 °C (petroleum ether).
**$^1$H-NMR** (400 MHz, $CDCl_3$): $\delta$ [ppm] = 7.23 (dd, $^3J$ = 8.4 Hz, $^4J$ = 1.9 Hz, 1H, 6-H, ph), 7.07 (d, $^4J$ = 1.9 Hz, 1H, 2-H, ph), 6.86 (d, $^3J$ = 8.4 Hz, 1H, 5-H, ph), 4.05 – 3.95 (m, 4H, $OCH_2$), 1.88 – 1.77 (m, 4H, $CH_2$), 1.46 – 1.27 (m, 24H, $CH_2$), 0.90 – 0.86 (m, 6H, $CH_3$). **$^{13}$C-NMR** (100 MHz, $CDCl_3$): $\delta$ [ppm] = 153.21, 149.18 (C-3, C-4), 126.44 (CH-ph), 119.61 (C-1), 116.13, 112.82 (CH-ph), 103.61 (C-7), 69.57, 69.23 ($OCH_2$), 32.02, 29.68, 29.48, 29.39, 29.15, 29.08, 26.07, 22.82 ($CH_2$), 14.25 ($CH_3$). **IR** (ATR): $\tilde{\upsilon}$ [cm$^{-1}$] = 2927 s, 2855 s, 2225 s, 1512 vs, 1468 m, 1267 s, 1136 s, 906 vs, 728 vs. **FD-MS:** m/z (%): 387.7 (100) [$M^+$].

**5-(3,4-Di(nonyloxy)phenyl)-2*H*-tetrazole**
According to the general procedure, 3.00 g (7.70 mmol) 3,4-di(nonyloxy)benzonitrile, 1.79 g (27.00 mmol) $NaN_3$, 3.73 g (27 mmol) $NEt_3HCl$ in 50 mL toluene, 36 h reflux. After 36 h, additional 0.50 g (8.3 mmol) $NaN_3$ and 1.00 g (8.3 mmol) triethylammonium chloride, 16 h reflux, gave 2.82 g (6.50 mmol, 85 %) of a colorless solid with m.p. = 159 – 160 °C (ethanol).
**$^1$H-NMR** (400 MHz, DMSO-$d_6$/$CDCl_3$): $\delta$ [ppm] = 7.55 – 7.53 (m, 2H, 2-H, 6-H, ph), 6.93 (d, $^3J$ = 8.9 Hz, 1H, 5-H, ph), 4.00 – 3.96 (m, 4H, $OCH_2$), 1.78 – 1.71 (m, 4H, $CH_2$), 1.46 – 1.38 (m, 4H, $CH_2$), 1.31 – 1.15 (m, 20H, $CH_2$), 0.80 (t, 6H, $CH_3$). **$^{13}$C-NMR** (100 MHz, DMSO-$d_6$/$CDCl_3$): $\delta$ [ppm] = 150.87, 148.79 (C-3, C-4), 119.76 (CH-ph), 116.18 (C-1), 112.69, 111.70 (CH-ph), 68.74, 68.55 ($OCH_2$), 31.30, 28.99, 28.96, 28.82, 28.80, 28.67, 28.66, 28.61, 28.54, 25.44, 25.39, 22.08 ($CH_2$), 13.54 ($CH_3$). **IR** (ATR): $\tilde{\upsilon}$ [cm$^{-1}$] = 2920 s, 2849 s, 1606 m, 1508 vs, 1464 s, 1270 vs, 1234 s, 1132 s, 1065 w, 1043 w, 813 m, 773 s, 745 s. **HR-ESI:** calcd. for $C_{25}H_{42}N_4O_2$ + $H^+$: 431.3386; found: 431.3377.

**3,7,11-Tris(3,4-di(nonyloxy)phenyl)tris([1,2,4]triazolo)[4,3-a:4',3'-c:4'',3''-e][1,3,5]triazine *t*-18**
According to the general procedure, 1.00 g (2.32 mmol) 5-(3,4-di(nonyloxy)phenyl)-2*H*-tetrazole and 0.13 g (0.70 mmol) cyanuric chloride in 25 mL xylenes with 0.30 mL *sym*-collidine were gradually heated to 60 °C for 3 h. Dilution with water, 20 mL 2N HCl and extraction with ethyl acetate (3 x 15 mL) gave the crude product that was absorbed on 0,5 g silica gel. Chromatography on basic alumina (toluene, followed by toluene/ethyl acetate 40/1) gave 0.73 g (0.57 mmol, 81 %) of a colorless solid with m.p. = 97 °C (DSC, 2. heating).
**$^1$H-NMR** (400 MHz, $CDCl_3$): $\delta$ [ppm] = 7.81 (dd, $^3J$ = 8.5 Hz $^4J$ = 2.1 Hz, 3H, 6-H), 7.69 (d, $^4J$ = 2.1 Hz, 3H, 2-H), 7.02 (d, $^3J$ = 8.5 Hz, 3H, 5-H), 4.11 – 4.08 (m, 12H, $OCH_2$), 1.91 – 1.82 (m, 12H, $CH_2$), 1.52 – 1.21 (m, 72H, $CH_2$), 0.90 – 0.85 (m, 18H, $CH_3$). **$^{13}$C-NMR** (100 MHz, $CDCl_3$): $\delta$ [ppm] = 152.38, 151.20 (C-4, ph; C-5, trl), 148.85 (C-3, ph), 140.64 (C-3, trl), 123.92 (C-6, ph), 115.95 (C-1, ph), 115.10 (C-5, ph), 112.57 (C-2, ph), 69.54, 69.20 ($OCH_2$), 32.05, 29.74, 29.59, 29.55, 29.44, 29.33, 29.24, 26.18, 26.16, 22.83 ($CH_2$), 14.26 ($CH_3$). **IR** (ATR): $\tilde{\upsilon}$ [cm$^{-1}$] = 3100 w, 2923 vs, 2854 s, 1598 s, 1493 s, 1468 s, 1263 vs, 1231 m, 1142 m, 1003 w, 883 w, 815 w, 714 m. **FD-MS:** m/z (%): 1282.7 (100) [$M^+$]. **HR-ESI:** calcd. for $C_{78}H_{123}N_9O_6$ + $H^+$: 1282.9675; found: 1282.9680.

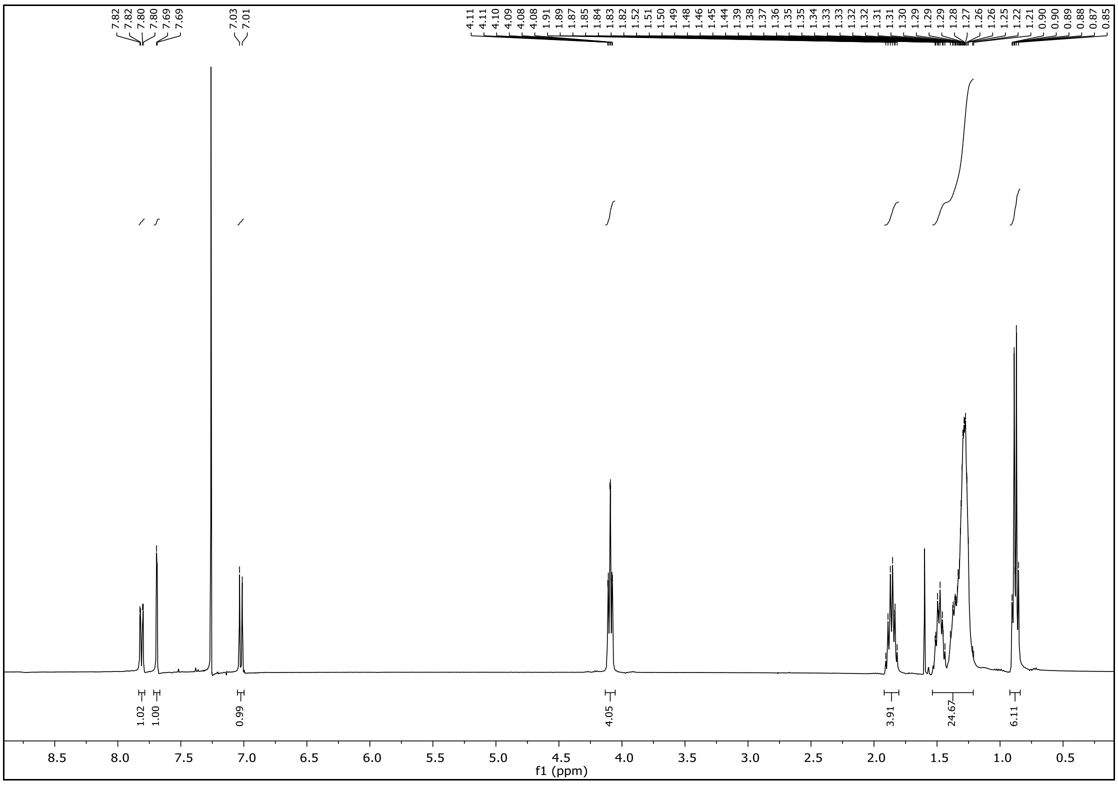


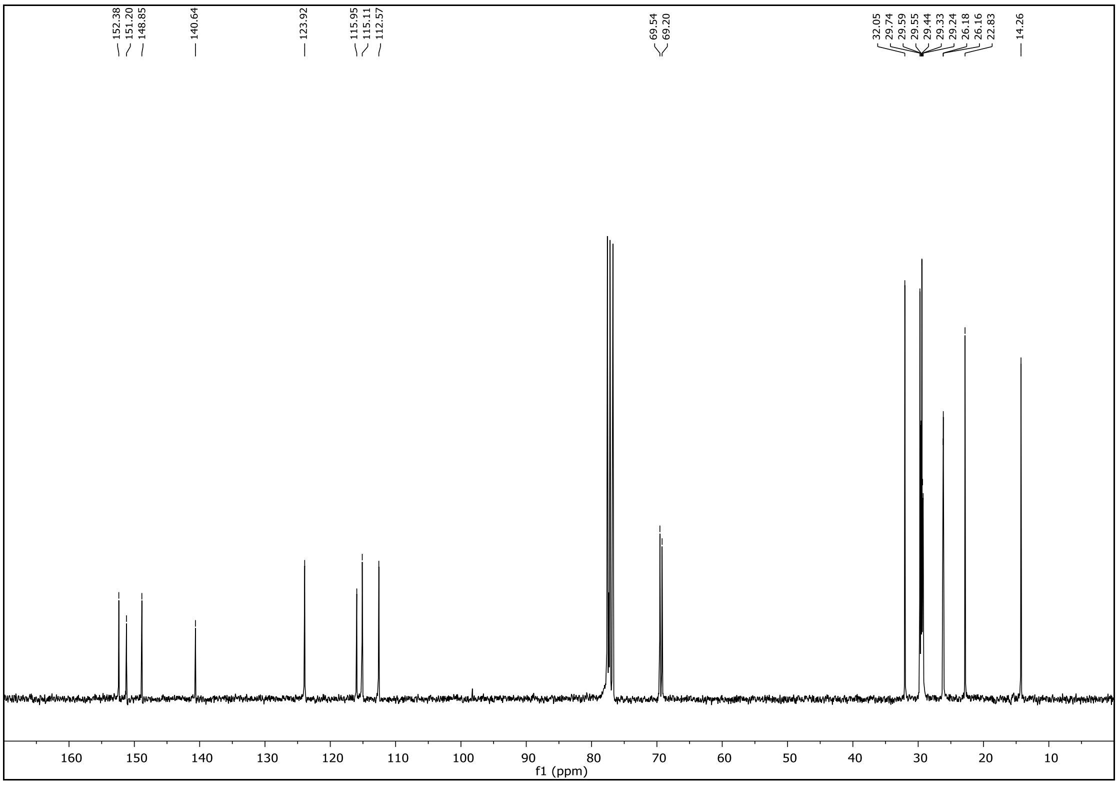


$^{1}$H-NMR and $^{13}$C-NMR of ***t*-18**

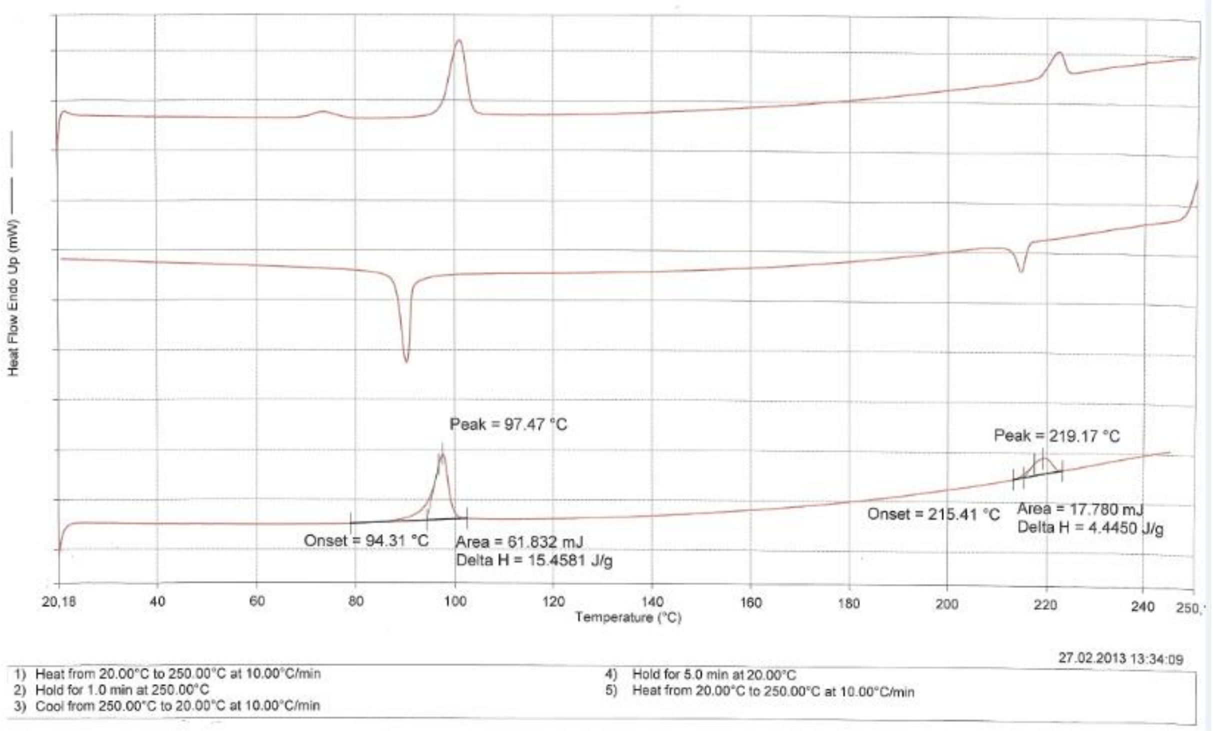


DSC of ***t*-18**

**3,4-Di(decyloxy)benzonitrile**
According to the general procedure, 2.02 g (15 mmol) 3,4-dihydroxybenzonitrile, 6.20 g (45 mmol) potash, 7,27 g (33 mmol) 1-bromodecane in 80 mL DMF under nitrogen for 48 h stirred at 80 °C, additional 2.42 g (11 mmol) 1-bromodecane, 2.07 g (15 mmol) potash and 12 h stirring at 80 °C. Acidulated with 2 N HCl, extraction with light petroleum, brine and $MgSO_4$ gave, after recrystallization from petroleum ether, 6.08 g (14.8 mmol, 98 %) of a colorless solid with m.p. = 78 – 79 °C (petroleum ether). **$^1$H-NMR** (400 MHz, $CDCl_3$): $\delta$ [ppm] = 7.23 (dd, $^3J$ = 8.4 Hz, $^4J$ = 1.9 Hz, 1H, 6-H, ph), 7.07 (d, $^4J$ = 1.9 Hz, 1H, 2-H, ph), 6.86 (d, $^3J$ = 8.4 Hz, 1H, 5-H, ph), 4.04 – 3.97 (m, 4H, $OCH_2$), 1.87 – 1.78 (m, 4H, $CH_2$), 1.50 – 1.27 (m, 28H, $CH_2$), 0.90 – 0.86 (m, 6H, $CH_3$). **$^{13}$C-NMR** (100 MHz, $CDCl_3$): $\delta$ [ppm] = 153.23, 149.20 (C-3, C-4), 126.45 (CH-ph), 119.61 (C-1), 116.18, 112.86 (CH-ph), 103.65 (C-7), 69.59, 69.26 ($OCH_2$), 32.05, 29.74, 29.70, 29.49, 29.17, 29.09, 26.08, 22.83 ($CH_2$), 14.26 ($CH_3$). **IR** (ATR): $\tilde{\nu}$ [cm$^{-1}$] = 2915 s, 2848 s, 2220 m, 1596 m, 1516 s, 1468 m, 1412 m, 1395 m, 1335 m, 1267 s, 1137 s, 1067 m, 992 m, 855 m, 721 m.

**5-(3,4-Di(decyloxy)phenyl)-2*H*-tetrazole**
According to the general procedure, 2.00 g (4.80 mmol) 3,4-di(decyloxy)benzonitrile, 1.11 g (17.00 mmol) NaN3, 2.33 g (17 mmol) $NEt_3HCl$ in 40 mL toluene, 24 h reflux. Then additional 0.32 g (4.8 mmol) NaN3 and 0.66 g (4.8 mmol) triethylammonium chloride, 12 h reflux. Yield: 1.74 g (3.80 mmol, 79 %) of a colorless solid with m.p. = 158 - 159 °C. **$^1$H-NMR** (400 MHz, DMSO-d$_6$/$CDCl_3$): $\delta$ [ppm] = 7.57 (d, $^4J$ = 2.0 Hz, 1H, 2-H, ph) 7.53 (dd, $^3J$ = 8.3 Hz, $^4J$ = 2.0 Hz 1H, 6-H, ph), 6.87 (d, $^3J$ = 8.3 Hz, 1H, 5-H, ph), 4.00 – 3.96 (m, 4H, $OCH_2$), 1.84 – 1.68 (m, 4H, $CH_2$), 1.84 – 1.68 (m, 4H, $CH_2$), 1.52 – 1.06 (m, 24H, $CH_2$), 0.95 – 0.63 (m, 6H, $CH_3$).

**3,7,11-Tris(3,4-di(decyloxy)phenyl)tris([1,2,4]triazolo)[4,3-a:4',3'-c:4'',3''-][1,3,5]triazine *t*-19**[ref 4]
According to the general procedure, 1.00 g (2.18 mmol) 5-(3,4-di(decyloxy)phenyl)-2*H*-tetrazole and 0.12 g (0.66 mmol) cyanuric chloride in 40 mL toluene with 0.50 mL *sym*-collidine were stirred for 6 h at ambient temperature. Dilution with water, 40 mL 2N HCl and extraction with ethyl acetate (3 x 30 mL) gave the crude product that was absorbed on 1.0 g

silica gel. Chromatography on a silica column with a head of basic alumina (toluene, followed by toluene/ethyl acetate 30/1; $R_f$ = 0.28 toluene/ethyl acetate 40:1) gave 0.73 g (0.57 mmol, 81 %) of a colorless solid with m.p. = 89 °C. **$^1$H-NMR** (400 MHz, $CDCl_3$): $\delta$ [ppm] = 7.81 (dd, $^3J$ = 8.5 Hz $^4J$ = 2.1 Hz, 1H, 6-H, ph), 7.69 (d, $^4J$ = 2.1 Hz, 1H, 2-H, ph), 7.03 (d, $^3J$ = 8.5 Hz, 1H, 5-H, ph), 4.11 – 4.08 (m, 4H, $OCH_2$), 1.91 – 1.82 (m, 4H, $CH_2$), 1.52 – 1.21 (m, 28H, $CH_2$), 0.90 – 0.85 (m, 6H, $CH_3$). **$^{13}$C-NMR** (75 MHz, CDCl3): □= 14.1 (CH3); 22.6, 26.0, 29.1, 29.3, 29.6, 31.9 (Alkoxy-CH2); 69.1, 69.4 (Ar-O-CH2); 112.5 (C(7)); 115.1 (C(4)); 115.8 (C(3)); 123.8 (C(2)); 140.5 C(1a)); 148.7 (C(5)); 151.1 (C(1b)); 152.3 (C(6)). **IR** (ATR): $\tilde{\upsilon}$ /cm$^{-1}$ = 2959; 2918; 2849; 1606; 1577; 1525; 1489; 1464; 144; 1395; 1339; 1261; 1225;1146; 1100; 1075; 1040; 1015; 987; 867; 808; 705. **FD-MS**: m/z (%)= 683.1 (1) $[M]^{2+\cdot}$.; 1366.2 (100), 1367.3 (92), 1368.3 (44) 1369. 3 (11) $[M]^{+\cdot}$. **EA:** $C_{84}H_{135}N_9O_6$ (1367.07) calcd.: 73.80 %C 9.95 %H 9.22 %N, found: 73.87 %C 9.88 %H 9.12%N.

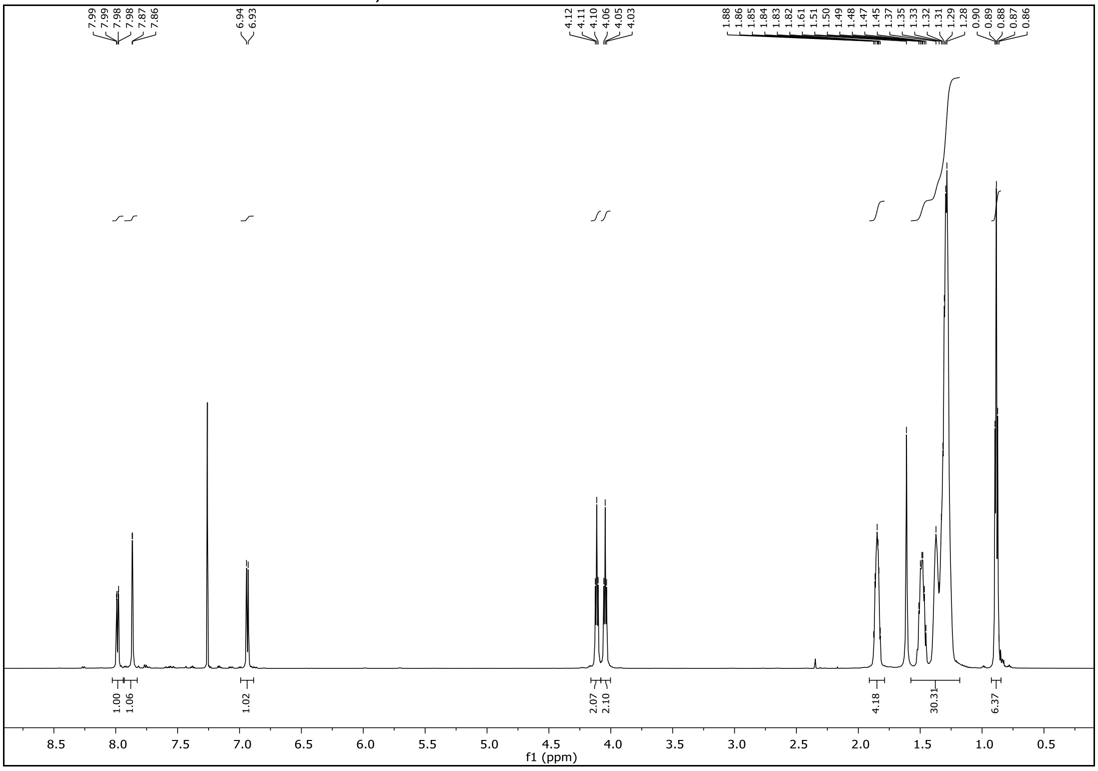

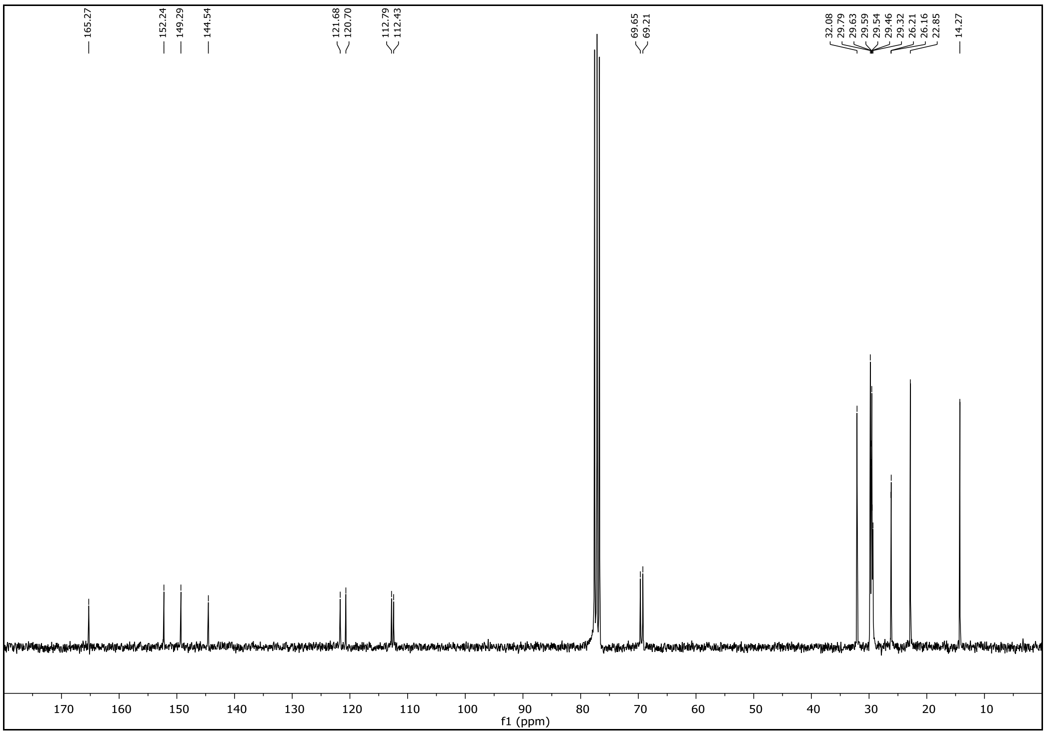


$^{1}$H-NMR and $^{13}$C-NMR of **t-19**

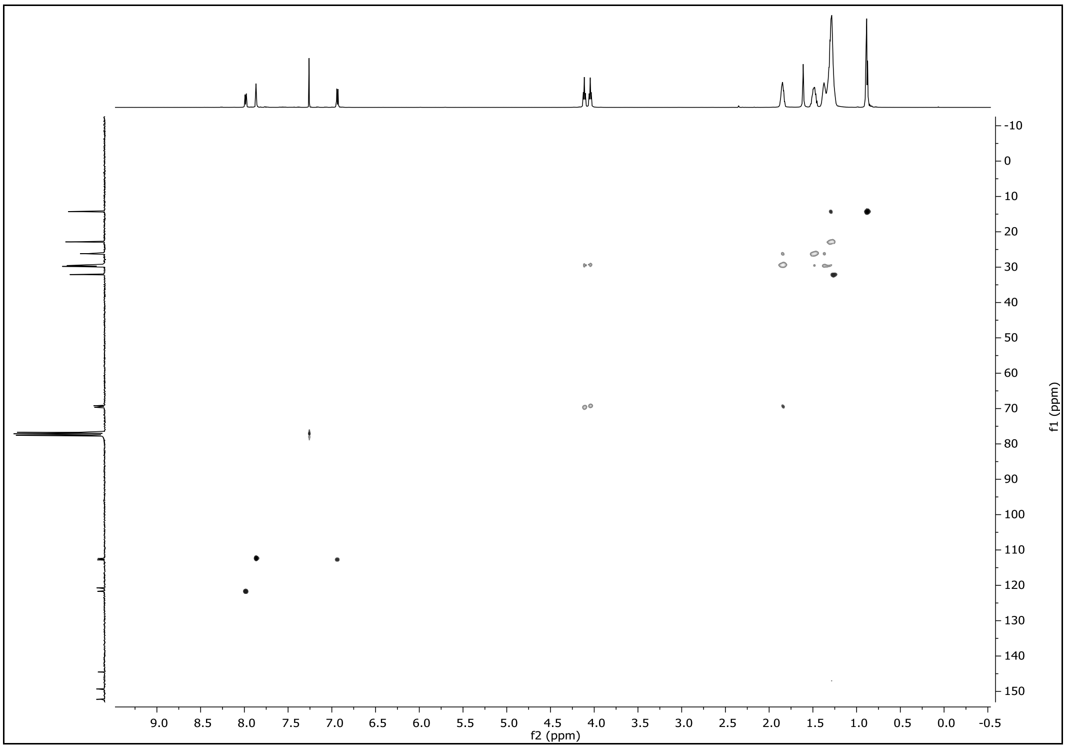


*HSQC-NMR-*

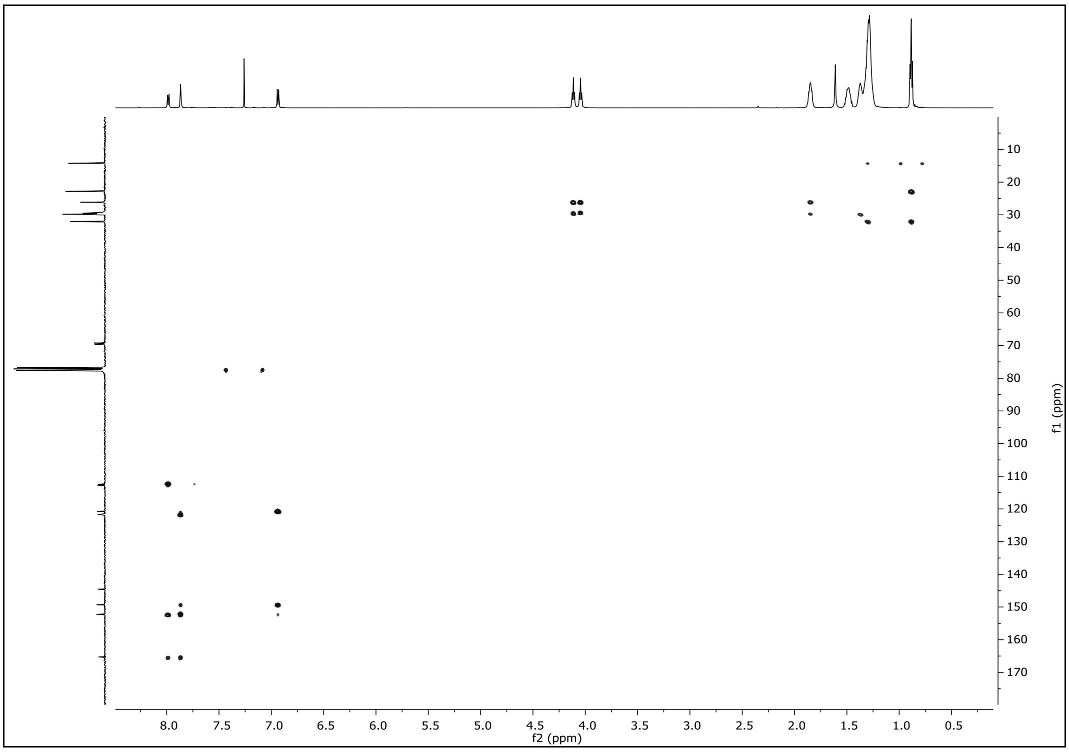


*HMBC-NMR*

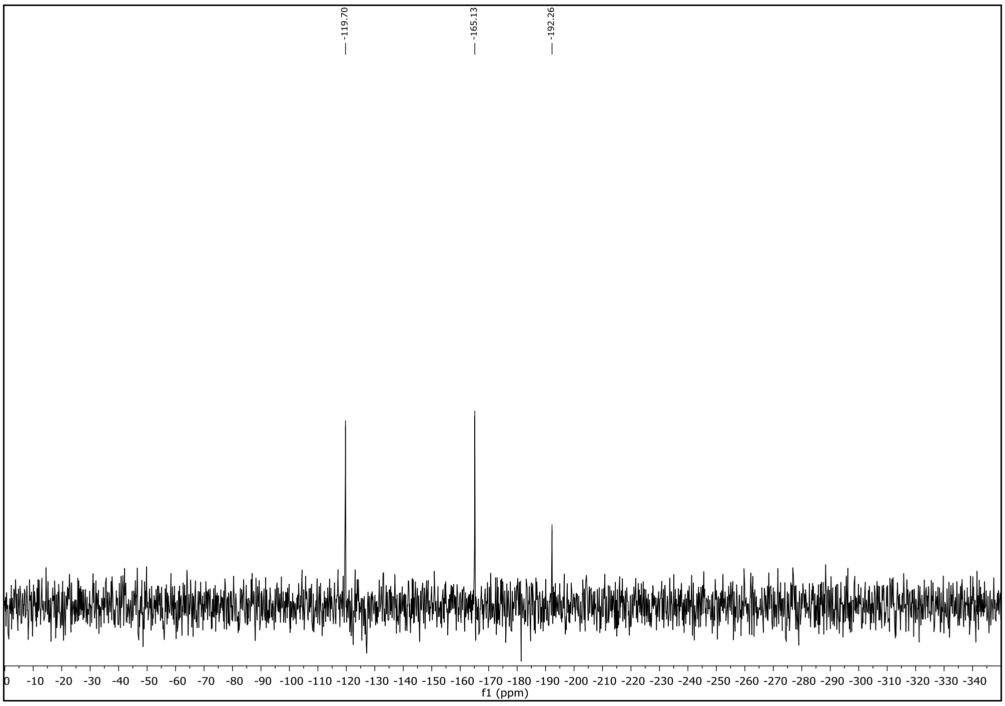


$^{15}$N-NMR of ***t*-19**

**2,6,10-Tris(3,4-di(decyloxy)phenyl)tris([1,2,4]triazolo)[1,5-a:1',5'-c:1'',5''-e][1,3,5]triazine *r*-19**

200 mg 3,7,11-Tris(3,4-di(decyloxy)phenyl)tris([1,2,4]triazolo)[4,3-a:4',3'-c:4'',3''-e][1,3,5]triazine in 2 mL 1-chloronaphthalene was stirred for 7 d unter nitrogen at 280 °C. Precipitation of the product by addition of light petroleum. Yield: 84 mg (0.6 mmol, 43 %) of a colorless solid with m.p. = 136 – 138 °C (petroleum ether). **$^{1}$H-NMR** (400 MHz, $CDCl_3$): $\delta$ [ppm] = 7.99 (dd, $^{3}J$ = 8.3, $^{4}J$ = 1.8 Hz, 1H, 6-H, ph), 7.87 (d, $^{4}J$ = 1.8 Hz, 1H, 2-H, ph), 6.94 (d, $^{3}J$ = 8.3 Hz, 1H, 5-H, ph), 4.11 (t, $^{3}J$ = 6.6 Hz, 2H, $OCH_2$), 4.05 (t, $^{3}J$ = 6.6 Hz, 2H, $OCH_2$), 1.89 – 1.80 (m, 4H, $CH_2$), 1.54 – 1.20 (m, 28H, $CH_2$), 0.92 – 0.84 (m, 6H, $CH_3$). **$^{13}$C-NMR** (100 MHz, $CDCl_3$):

$\delta$ [ppm] = 165.27 (C-5, trl), 152.24 (C-4, ph), 149.29 (C-3, ph), 144.54 (C-3, trl), 121.68 (C-6, ph), 120.70 (C-1, ph), 112.79 (C-5, ph), 112.43 (C-2, ph), 69.65, 69.21 ($OCH_2$), 32.08, 29.79, 29.63, 29.59, 29.54, 29.46, 29.32, 26.21, 26.16, 22.85 ($CH_2$), 14.2 ($CH_3$). **$^{15}$N-NMR** (40,6 MHz, $CDCl_3$, $\delta$ $CD_3NO_2$ = 0 ppm): $\delta$ [ppm] = -119.70, -165.13, -192.26. **IR (ATR):** $\tilde{\upsilon}$ [$cm^{-1}$] = 2919 s, 2849 m, 1629 s, 1604 m, 1461 s, 1390 m, 1306 w, 1262 s, 1221 s, 1138 s, 1069 w, 1021 w, 973 w, 938 w, 866 w, 810 w, 747 s, 723 m, 659 w. **HR-ESI:** calcd. for $C_{84}H_{135}N_9O_6$ + $H^+$: 1367.0614; found: 1367.0547.

**3,4-Di(undecyloxy)benzonitrile**

According to the general procedure, 2.00 g (15 mmol) 3,4-dihydroxybenzonitrile, 6.14 g (44 mmol) potash, 10.44 g (44 mmol) 1-bromoundecane in 80 mL DMF under nitrogen for 18 h stirred at 80 °C, additional 5.20 g (22 mmol) 1-bromoundecane, 3.0 g (22 mmol) potash and 24 h stirring at 80 °C. Acidulated with 2 N HCl, extraction with light petroleum (3*50 ml), brine and $MgSO_4$ gave, after recrystallization from petroleum ether, 5.16 g (12 mmol, 79 %) of a colorless solid with m.p. = 80 – 81 °C (petroleum ether).

**$^1$H-NMR** (400 MHz, $CDCl_3$): $\delta$ [ppm] = 7.23 (dd, $^3J$ = 8.4 Hz, $^4J$ = 1.9 Hz, 1H, 6-H, ph), 7.06 (d, $^4J$ = 1.9 Hz, 1H, 2-H, ph), 6.86 (d, $^3J$ = 8.4 Hz, 1H, 5-H, ph), 4.07 – 3.96 (m, 4H, $OCH_2$), 1.86 – 1.76 (m, 4H, $CH_2$), 1.51 – 1.22 (m, 32H, $CH_2$), 0.90 – 0.85 (m, 6H, $CH_3$). **$^{13}$C-NMR** (75 MHz, $CDCl_3$): $\delta$ [ppm] = 153.21, 149.18 (C-3, C-4), 126.45 (CH-ph), 119.61 (C-1), 116.14, 112.83 (CH-ph), 103.62 (C-7), 69.57, 69.24 ($OCH_2$), 32.06, 29.74, 29.50, 29.16, 29.08, 26.08, 22.84 ($CH_2$), 14.26 ($CH_3$). **IR** (ATR): $\tilde{\upsilon}$ [$cm^{-1}$] = 2919 vs, 2849 vs, 2216 w, 1596 w, 1519 s, 1467 s, 1278 s, 1138 s, 983 m, 856 w, 811w, 721 m.

**5-(3,4-Di(undecyloxy)phenyl)-2*H*-tetrazole**

According to the general procedure, 2.00 g (4.50 mmol) 3,4-di(undecyloxy)benzonitrile, 1.04 g (16.00 mmol) $NaN_3$, 2.17 g (16 mmol) $NEt_3HCl$ in 40 mL toluene, 18 h reflux. Then additional 0.30 g (4.6 mmol) $NaN_3$ and 0.60 g (4.4 mmol) triethylammonium chloride, 6 h reflux. Yield: 1.94 g (4.00 mmol, 88 %) of a colorless solid with m.p. = 157 - 158 °C.

**$^1$H-NMR** (400 MHz, DMSO-$d_6$/$CDCl_3$): $\delta$ [ppm] = 7.56 – 7.54 (m, 2H, 2-H, 6-H, ph), 6.95 (d, $^3J$ = 8.9 Hz, 1H, 5-H, ph), 4.03 – 3.97 (m, 4H, $OCH_2$), 1.79 – 1.71 (m, 4H, $CH_2$), 1.47 – 1.13 (m, 32H, $CH_2$), 0.81 (t, 6H, $CH_3$). **IR** (ATR): $\tilde{\upsilon}$ [$cm^{-1}$] = 2918 vs, 2848 vs, 1607 m, 1509 vs, 1464 s, 1268 vs, 1235 s, 1132 m, 1038 m, 873 m, 812 m, 745 s. **HR-ESI:** calcd. for $C_{29}H_{50}N_4O_2$ + $H^+$: 487.4012; found: 487.4019.

**3,7,11-Tris(3,4-di(undecyloxy)phenyl)tris([1,2,4]triazolo)[4,3-a:4',3'-c:4'',3''-e][1,3,5]triazine *t*-20**

According to the procedure above, 1.00 g (2.05 mmol) 5-(3,4-di(undecyloxy)phenyl)-2*H*-tetrazole and 0.11 g (0.60 mmol) cyanuric chloride in 40 mL xylenes with 0.30 mL *sym*-collidine were stirred for 3 h at 60 °C. Dilution with water, 40 mL 2N HCl and extraction with ethyl acetate (3 x 30 mL) gave the crude product that was absorbed on 1.0 g silica gel. Chromatography on a column of basic alumina (toluene, followed by toluene/ethyl acetate 40/1) gave 0.74 g (0.51 mmol, 68 %) of a colorless solid with m.p. = 85 (DSC, 2$^{nd}$ heating).

**$^1$H-NMR** (400 MHz, $CDCl_3$): $\delta$ [ppm] = 7.81 (dd, $^3J$ = 8.5 Hz $^4J$ = 2.1 Hz, 3H, 6-H, ph), 7.69 (d, $^4J$ = 2.1 Hz, 3H, 2-H, ph), 7.02 (d, $^3J$ = 8.5 Hz, 3H, 5-H, ph), 4.11 – 4.08 (m, 12H, $OCH_2$), 1.91 – 1.82 (m, 12H, $CH_2$), 1.52 – 1.22 (m, 96 H, $CH_2$), 0.90 – 0.85 (m, 18H, $CH_3$). **$^{13}$C-NMR** (75 MHz, $CDCl_3$): $\delta$ [ppm] = 152.37, 151.18 (C-4, ph; C-5, trl), 148.84 (C-3, ph), 140.63 (C-3, trl), 123.91 (C-6), 115.95 (C-1, ph), 115.12 (C-5, ph), 112.57 (C-2, ph), 69.55, 69.20 ($OCH_2$), 32.06, 29.79, 29.60, 29.59, 29.56, 29.51, 29.34, 29.24, 26.19, 26.16, 22.83 ($CH_2$), 14.26 ($CH_3$). **IR** (ATR): $\tilde{\upsilon}$ [$cm^{-1}$] = 2923 vs, 2853 s, 1598 m, 1492 s, 1468 s, 1264 s, 1142 m, 1003 w, 810 w, 718 w. **HR-ESI:** calcd. for $C_{90}H_{147}N_9O_6$ + $H^+$: 1451.1553; found: 1451.1530.

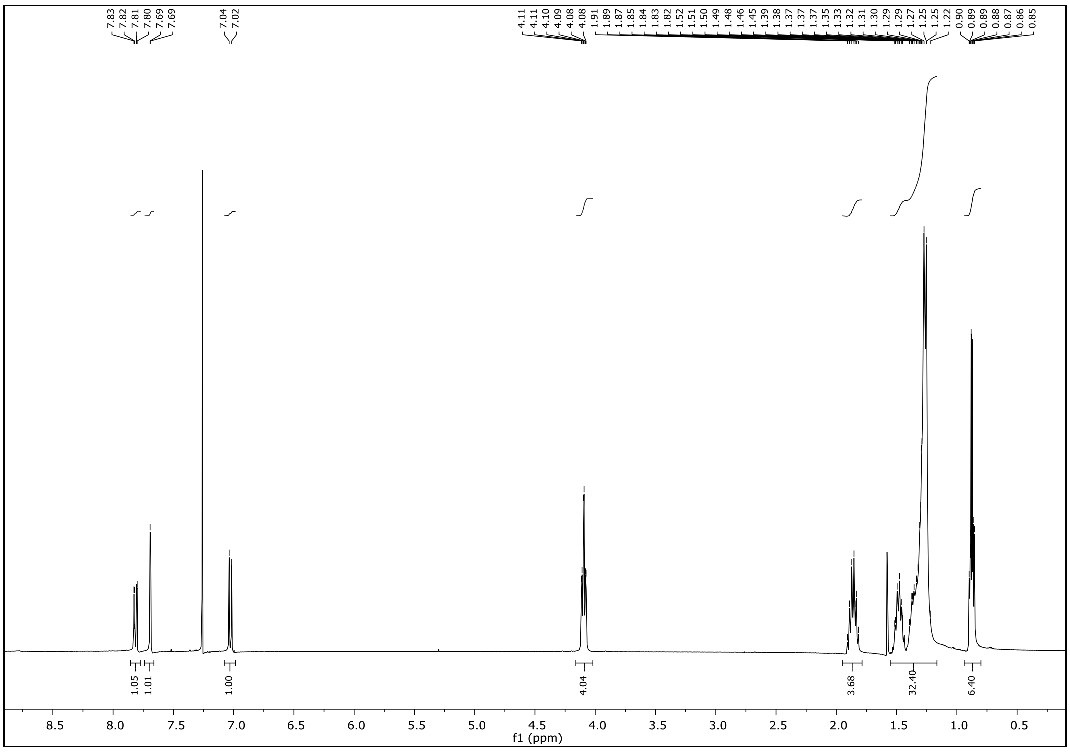


$^{1}$H-NMR and $^{13}$C-NMR of ***t*-20**

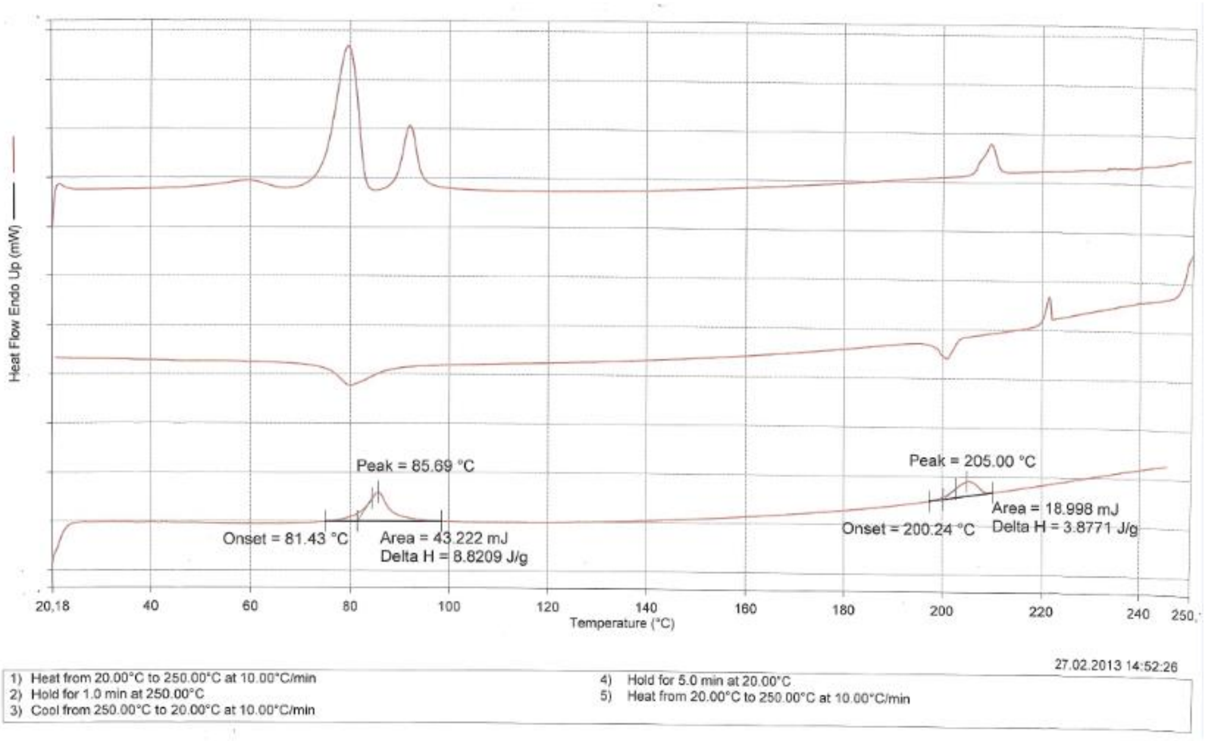


DSC of ***t*-20**

**3,7,11-Tris(3,4-di(dodecyloxy)phenyl)tris([1,2,4]triazolo)[4,3-a:4',3'-c:4'',3''-e]-[1,3,5]triazine *t*-21** [3]

According to the general procedure, 1.00 g (2.00 mmol) 5-(3,4-di(dodecyloxy)phenyl)-2*H*-tetrazole and 0.108 g (0.58 mmol) cyanuric chloride in 50 mL toluene with 0.50 mL *sym*-collidine were stirred for 14 h at ambient temperature (TLC). Dilution with water, 50 mL 2N

HCl and extraction with ethyl acetate (3 x 30 mL) gave the crude product that was absorbed on 1.0 g silica gel. Chromatography on a silica column (2*35 cm) with a head of basic alumina (5 cm; toluene, followed by toluene/ethyl acetate 30/1) gave 0.64 g (0.42 mmol, 71 %) of a colorless solid. **$^1$H-NMR** (400 MHz, $CDCl_3$): $\delta$ [ppm] = 7.82 (dd, $^3J$ = 8.4 Hz $^4J$ = 2.1 Hz, 1H, 6-H, ph), 7.70 (d, $^4J$ = 2.1 Hz, 1H, 2-H, ph), 7.03 (d, $^3J$ = 8.4 Hz, 1H, 5-H, ph), 4.12 – 4.08 (m, 4H, $OCH_2$), 1.91 – 1.82 (m, 4H, $CH_2$), 1.52 – 1.25 (m, 36 H, $CH_2$), 0.89 – 0.86 (m, 6H, $CH_3$).

**2,6,10-Tris(3,4-di(dodecyloxy)phenyl)tris([1,2,4]triazolo)[1,5-a:1',5'-c:1'',5''-e][1,3,5]triazine *r*-21**

144 mg 3,7,11-Tris(3,4-di(dodecyloxy)phenyl)tris([1,2,4]triazolo)[4,3-a:4',3'-c:4'',3''-e][1,3,5]triazine in 2 mL octadecane was stirred for 64 h unter nitrogen at 275 °C. The mixture was diluted with petroleum ether, filtered through a silica column (1*10 cm) with petroleum ether to elute octadecane followed by ethyl acetate to elute TTT. The crude product was absorbed on silica gel, chromatography with a gradient from petroleum to petroleum ether/toluene 3/1. Yield: 45 mg (38 %) of a colorless solid with m.p. = 131 °C (DSC, 2$^{nd}$ heating).
**$^1$H-NMR** (400 MHz, $CDCl_3$): $\delta$ [ppm] = 7.99 (dd, $^3J$ = 8.4, $^4J$ = 2.0 Hz, 1H, 6-H, ph), 7.87 (d, $^4J$ = 2.0 Hz, 1H, 2-H, ph), 6.95 (d, $^3J$ = 8.4 Hz, 1H, 5-H, ph), 4.11 (t, $^3J$ = 6.7 Hz, 2H, $OCH_2$), 4.05(t, $^3J$ = 6.7 Hz, 2H, $OCH_2$), 1.87 – 1.82 (m, 4H, $CH_2$), 1.52 – 1.27 (m, 36H, $CH_2$), 0.90 – 0.86 (m, 6H, $CH_3$). **$^{13}$C-NMR** (75 MHz, $CDCl_3$): $\delta$ [ppm] = 165.33 (C-5, trl), 152.25 (C-4, ph), 149.31 (C-3, ph), 144.56 (C-3, trl), 121.69 (C-6, ph), 120.69 (C-1, ph), 112.80 (C-5, ph), 112.43 (C-2, ph), 69.64, 69.22 ($OCH_2$), 32.09, 29.90, 29.85, 29.81, 29.64, 29.60, 29.55, 29.46, 29.40, 29.32, 26.22, 26.17, 22.86 ($CH_2$), 14.28 ($CH_3$). **IR** (ATR): $\tilde{\nu}$ [cm$^{-1}$] = 2918 s, 2849 s, 1630 s, 1607 m, 1539 w, 1462 s, 1390 m, 1262 s, 1220 s, 1138 s, 1070 w, 998 w, 864 w, 810 w, 746 s. **HR-ESI:** calcd. for $C_{96}H_{159}N_9O_6 + H^+$: 1535.2492; found: 1535.2469.

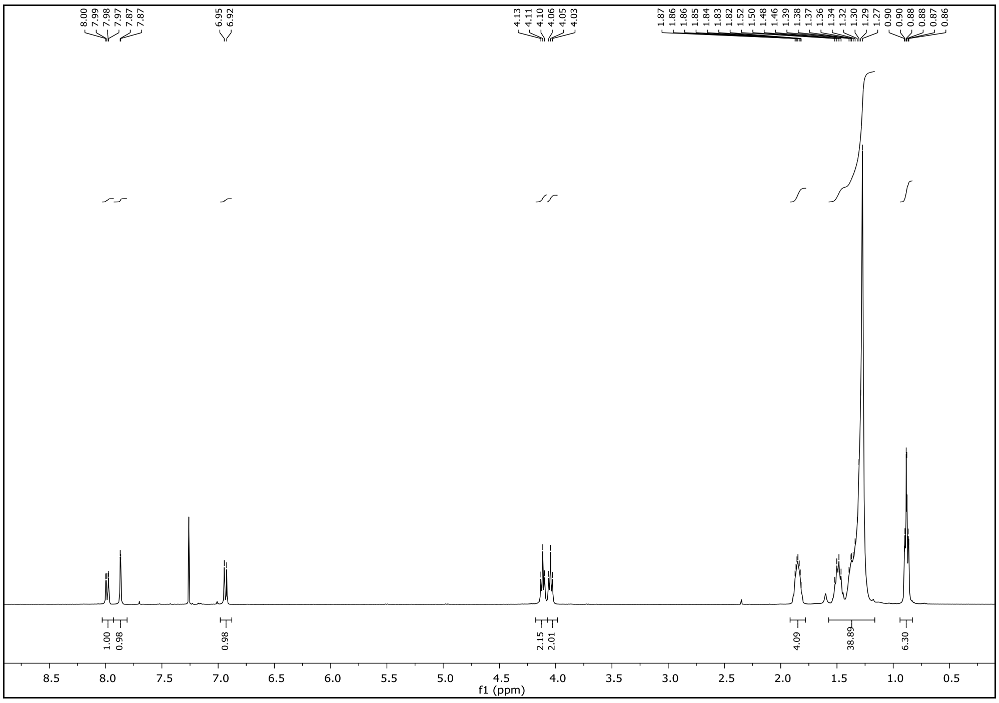

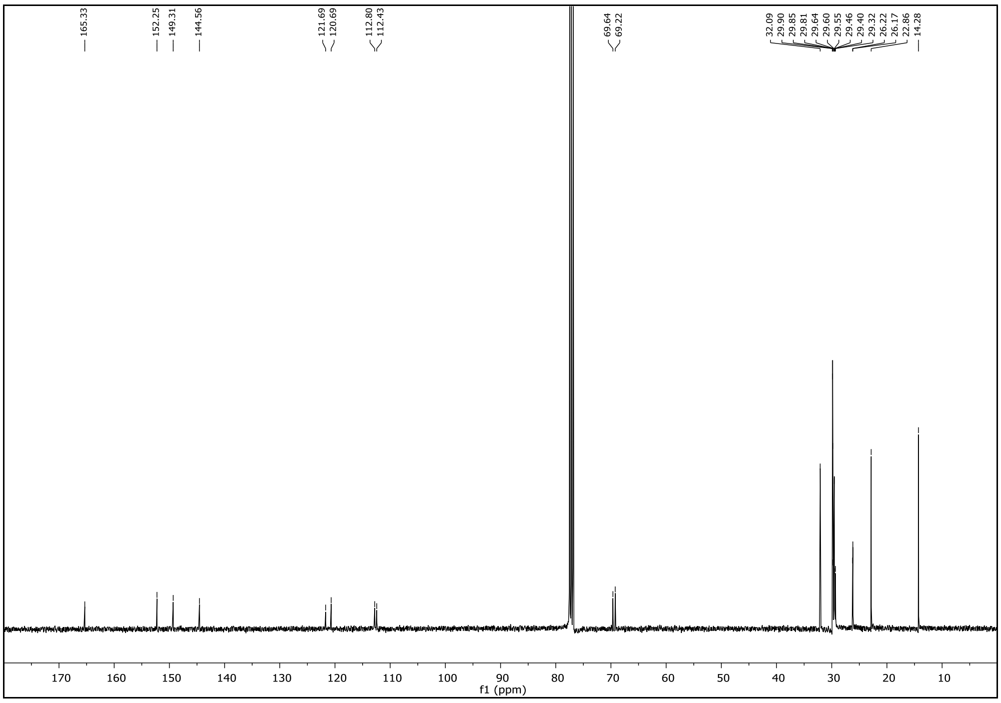


$^{1}$H-NMR and $^{13}$C-NMR of ***r*-21**

**3,4-Di(tridecyloxy)benzonitrile**

According to GP1, the nitrile was obtained in 80 % yield after recrystallisation from ethanol, m. p. 84-85 °C.

**IR:** (ATR) ṽ = 2914, 2847, 2219, 1597, 1580, 1517, 1468, 1421, 1395, 1336, 1277, 1242, 1138, 1070, 1000, 855, 809, 775, 721 cm$^{-1}$; **$^{1}$H-NMR:** (300 MHz, $CDCl_3$) $\delta$ = 7.23 (dd, $^{3}J$ = 8.3 Hz, $^{4}J$ = 1.9 Hz, 1H, 6-H), 7.07 (d, $^{4}J$ = 1.9 Hz, 1H, 2-H), 6.86 (d, $^{3}J$ = 8.4 Hz, 1H, 5-H), 4.07 – 3.92 (m, 4H, O$CH_2$), 1.87 – 1.74 (m, 4H, $CH_2$), 1.53 – 1.18 (m, 44H, $CH_2$), 0.93 – 0.82 (m, 6H, $CH_3$) ppm; **$^{13}$C-NMR:**(75 MHz, $CDCl_3$) $\delta$ = 153.20, 149.17 (C-3, C-4), 126.44 (CH-ph), 119.61 (C-1), 116.12 (CH-ph), 112.81 (CH-ph), 103.62 (C-7, CN), 69.56, 69.23 ($OCH_2$), 32.07, 29.85, 29.83, 29.81, 29.75, 29.51, 29.48, 29.16, 29.08, 26.09, 26.07, 22.84 ($CH_2$), 14.27 ($CH_3$) ppm.

**5-(3,4-Di(tridecyloxy)phenyl-2*H*-tetrazole**

According to GP5, the nitrile was obtained in 86 % yield after recrystallisation from chloroform, m. p. 157-158 °C. **IR:** (ATR) ṽ = 2916, 2848, 1606, 1509, 1464, 1269, 1235, 1131, 1070, 808, 778, 744 cm$^{-1}$; **$^{1}$H-NMR:** (300 MHz, $CDCl_3$ + 1 Tropfen DMSO-*d6*) $\delta$ = 7.60 (d, $^{4}J$ = 1.7 Hz, 1H, 2-H, ph), 7.58 – 7.51 (m, 1H, 6-H, ph), 6.90 (d, $^{3}J$=8.4 Hz, 1H, 5-H, ph), 4.06 – 3.94 (m, 4H, O$CH_2$), 1.85 – 1.71 (m, 4H, $CH_2$), 1.50 – 1.10 (m, 43H, $CH_2$), 0.86 – 0.76 (m, 6H, $CH_3$) ppm.

**3,7,11-Tris(3,4-di(tridecyloxy)phenyl)tris([1,2,4]triazolo)[4,3-a:4',3'-c:4",3"-e] [1,3,5]tri-azine *t*-22**

According to GP5, the nitrile was obtained in 86 % yield after chromatography, $SiO_2$ + 5 cm $Al_2O_3$; toluene:ethyl acetate = 30:1). M. p. 124 °C (DSC). **IR:** (ATR) ṽ = 2918, 2849, 1609, 1576, 1465, 1260, 1223, 1146, 808, 772, 720 cm$^{-1}$; **$^{1}$H-NMR:** (300 MHz, $CDCl_3$) $\delta$ =7.82 (dd, $^{3}J$=8.5, $^{4}J$ = 2.1 Hz, 1H, 6-H, ph), 7.70 (d, $^{4}J$ = 2.1 Hz, 1H, 2-H, ph), 7.03 (d, $^{3}J$ = 8.6 Hz, 1H, 5-H, ph), 4.10 (t, $^{3}J$ = 6.5 Hz, 4H, O$CH_2$), 1.93 – 1.80 (m, 4H, $CH_2$), 1.56 – 1.20 (m, 48H, $CH_2$), 0.91 – 0.85 (m, 6H, $CH_3$) ppm; **$^{13}$C-NMR:** (75 MHz, $CDCl_3$) $\delta$ = 152.39, 151.21 (C-4, ph; C-5, trl), 148.86 (C-3, ph), 140.64 (C-3, trl), 123.93 (C-6, ph), 115.96 (C-1, ph), 115.12 (C-5,ph), 112.58 (C-2, ph), 69.55, 69.22 ($OCH_2$), 32.08, 29.87, 29.83, 29.80, 29.61, 29.57, 29.53, 29.35, 29.25, 26.20, 26.17, 22.85 ($CH_2$), 14.27 ($CH_3$) ppm; **FD-MS:** m/z (%): 810.4 (7), 811.1 $[M]^{\bullet\bullet 2+}$; 1618.1 (100), 1620.8 (83), 1621.5 (15), 1622.1 (31), 1623.2 (7) $[M]^{\bullet +}$.

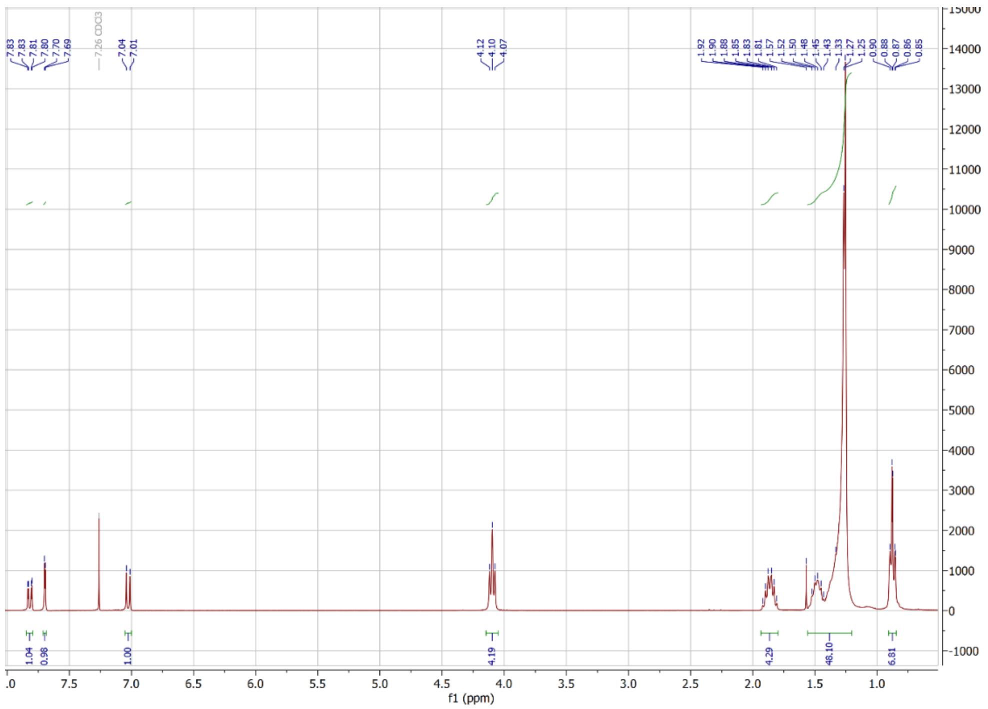


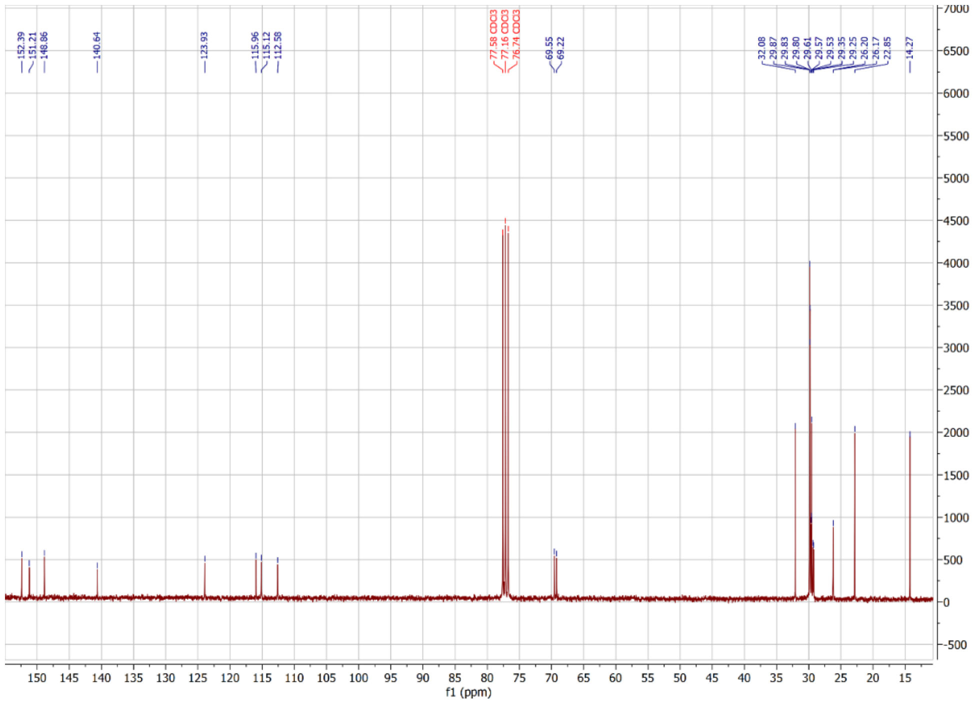


$^{1}$H- and $^{13}$C-NMR of ***t*-22**

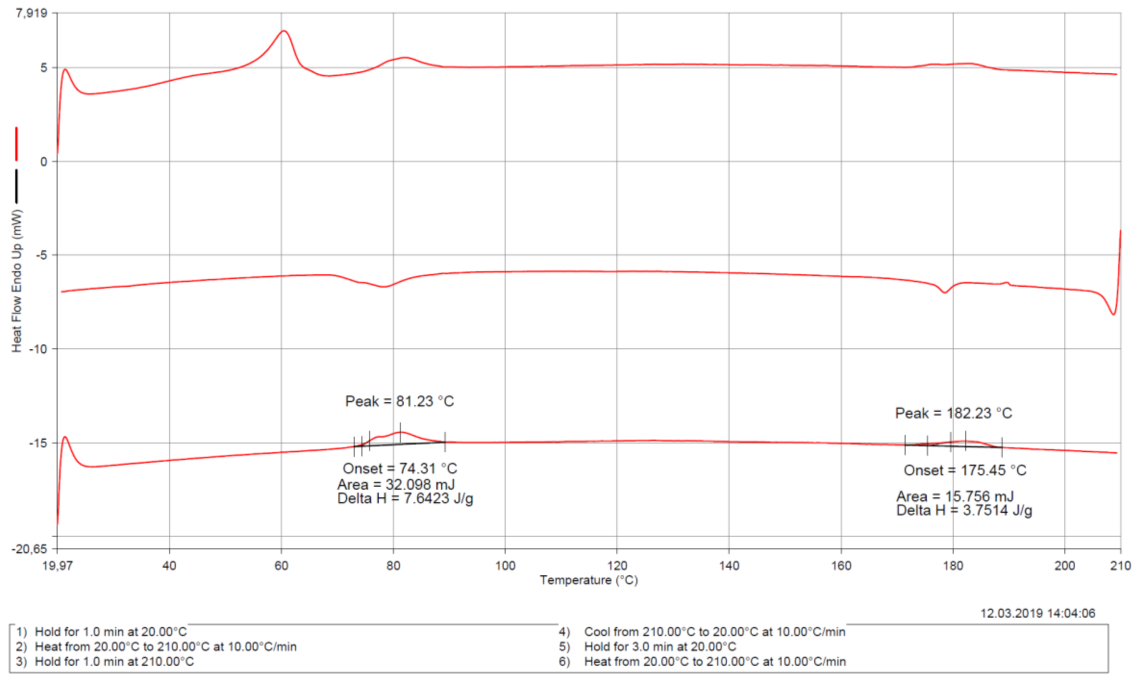


DSC of ***t*-22**

**2,6,10-Tris(3,4-di(tridecyloxy)phenyl)tris([1,2,4]triazolo)[1,5-a:1',5'-c:1'',5''-e][1,3,5]triazine *r*-22**

3,7,11-Tris(3,4-di(tridecyloxy)phenyl)tris([1,2,4]triazolo)[4,3-a:4',3'-c:4'',3''-e][1,3,5]triazine (69 mg, 0.04 mmol), 4-bromobenzoic acid (24 mg, 0.12 mmol) and octadecane (1.652 g) were heated under nitrogen for 64 h at 235 °C. the solvent was removed by chromatography ($SiO_2$+1 cm $Al_2O_3$; petroleum ether → toluene:ethyl acetate = 30:1). The crude product was purified by chromatography ($SiO_2$; petroleum ether: ethyl acetate = 5:1 → toluene: ethyl acetate = 30:1) to yield 19 mg (0.01 mmol, 28%) of a colourless solid with m.p. = 93 °C.

**IR:** (ATR) ṽ = 2918, 2849, 1631, 1605, 1535, 1461, 1391, 1308, 1262, 1220, 1139, 1070, 864, 808, 747, 722 $cm^{-1}$. **$^1$H-NMR:** (400 MHz, $CDCl_3$) $\delta$ = 8.02 (dd, $^3J$ = 8.4 Hz, $^4J$ = 2.0 Hz, 1H, 6-H, ph), 7.90 (d, $^4J$ = 2.0 Hz, 1H, 2-H, ph), 6.98 (d, $^3J$=8.6 Hz, 1H, 5-H, ph), 4.14 (t, $^3J$=6.6 Hz, 2H, $OCH_2$), 4.08 (t, $^3J$=6.6 Hz, 2H, $OCH_2$) 1.95 – 1.80 (m, 4H, $CH_2$), 1.54 – 1.17 (m, 44H, $CH_2$), 0.91 – 0.84 (m, 6H, $CH_3$) ppm. **$^{13}$C-NMR:** (75 MHz, $CDCl_3$) $\delta$ = 165.31 (C-5, trl), 152.25 (C-4, ph), 149.30 (C-3, ph), 144.55 (C-3, trl), 121.69 (C-6, ph), 120.69 (C-1, ph), 112.78 (C-5, ph), 112.43 (C-2, ph), 69.64, 69.21 ($OCH_2$), 32.09, 29.91, 29.89, 29.85, 29.82, 29.65, 29.60, 29.54, 29.47, 29.33, 26.22, 26.17, 22.85, ($CH_2$), 14.28 ($CH_3$) ppm. **APCI-MS:** $C_{102}H_{171}N_9O_6$. 1619.55 g/mol.m/z (%): 1619.34 (89), 1620.34 (100), 1621.35 (44).

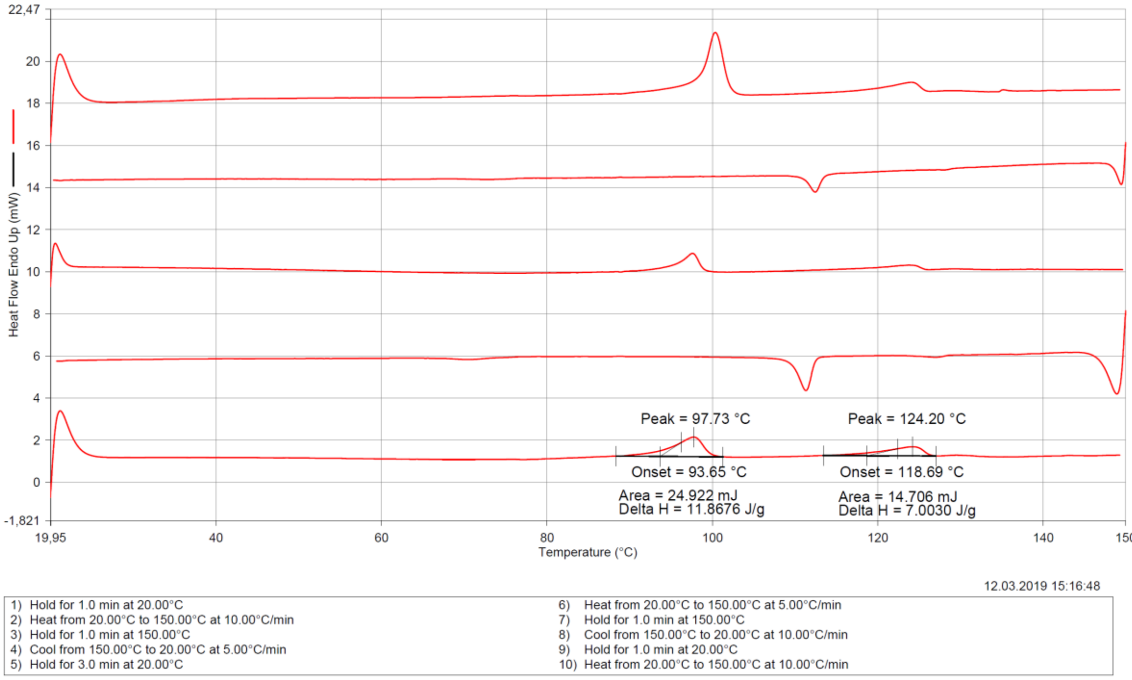


DSC of ***r*-22**

**3,4-Di(tetradecyloxy)benzonitrile**
According to the general procedure, 2.00 g (15 mmol) 3,4-dihydroxybenzonitrile, 6.14 g (44 mmol) potash, 9.03 g (33 mmol) 1-bromotetradecane in 60 mL DMF under nitrogen for 12 h stirred at 80 °C, additional 4.51 g (16 mmol) 1-bromotetradecane, 3.07 g (22 mmol) potash and 48 h stirring at 80 °C. Acidulated with 2 N HCl, extraction with light petroleum (3*80 ml), brine and $MgSO_4$ gave, after recrystallization from petroleum ether, 7.36 g (12 mmol, 93 %) of a colorless solid with m.p. = 84 – 85 °C (petroleum ether).
**$^1$H-NMR** (400 MHz, $CDCl_3$): $\delta$ [ppm] = 7.23 (dd, $^3J$ = 8.3, $^4J$ = 1.9 Hz, 1H, 6-H, ph), 7.07 (d, $^4J$ = 1.9 Hz, 1H, 2-H, ph), 6.86 (d, $^3J$ = 8.4 Hz, 1H, 5-H, ph), 4.04 – 3.97 (m, 4H, $OCH_2$), 1.89 – 1.76 (m, 4H, $CH_2$), 1.51 – 1.20 (m, 44H, $CH_2$), 0.88 (t, 6H, $CH_3$). **$^{13}$C-NMR** (75 MHz, $CDCl_3$): $\delta$ [ppm] = 153.20, 149.18 (C-3, C-4), 126.44 (CH-ph), 119.60 (C-1), 116.13, 112.83 (CH-ph), 103.62 (C-7), 69.58, 69.24 ($OCH_2$), 32.08, 29.86, 29.75, 29.52, 29.17, 29.09, 26.09, 22.85 ($CH_2$), 14.27 ($CH_3$). **IR** (ATR): $\tilde{\upsilon}$ [cm$^{-1}$] = 2916 vs, 2848 vs, 2223 w, 1519 s, 1467 s, 1420 m, 1278 s, 1241 m, 1138 s, 1004 m, 856 m, 810 m, 721 m, 669 m.

**5-(3,4-Di(tetradecyloxy)phenyl)-2*H*-tetrazole**
According to the general procedure, 3.00 g (5.70 mmol) 3,4-di(tetradecyloxy)benzonitrile, 1.31 g (19.00 mmol) $NaN_3$, 2.74 g (19 mmol) $NEt_3HCl$ in 60 mL toluene, 18 h reflux. Then additional 0.38 g (5.7 mmol) $NaN_3$ and 0.78 g (5.7 mmol) triethylammonium chloride, 24 h reflux. Yield: 2.74 g (4.80 mmol, 84 %) of a colorless solid with m.p. = 152 - 153 °C (ethanol).
**$^1$H-NMR** (400 MHz, DMSO-d$_6$/$CDCl_3$): $\delta$ [ppm] = 7.61 (d, $^4J$ = 2.0 Hz, 1H, 2-H, ph), 7.56 (dd, $^3J$ = 8.4 Hz, $^4J$ = 2.0 Hz, 1H, 6-H, ph), 6.91 (d, $^3J$ = 8.4 Hz, 1H, 5-H, ph), 4.03 – 3.97 (m, 4H, $OCH_2$), 1.83 – 1.74 (m, 4H, $CH_2$), 1.48 – 1.13 (m, 44H, $CH_2$), 0.82 (t, $^3J$ = 6.8 Hz, 6H, $CH_3$). **$^{13}$C-NMR** (100 MHz, DMSO-d$_6$/$CDCl_3$): $\delta$ [ppm] = 150.85, 148.75 (C-3, C-4), 119.77 (CH-ph), 116.13 (C-1), 112.69, 111.69 (CH-ph), 68.72, 68.51 ($OCH_2$), 31.29, 29.08, 29.03, 28.99, 28.80, 28.73, 28.60, 28.52, 25.44, 25.39, 22.06 ($CH_2$), 13.56 ($CH_3$). Due to poor solubility, some carbon signals are missing. **IR** (ATR): $\tilde{\upsilon}$ [cm$^{-1}$] = 2917 vs, 2848 s, 1606 w, 1511 s, 1465 s, 1270, s, 1234 s, 1132 m, 772 s, 721 w, 669 w. **HR-ESI:** calcd. for $C_{35}H_{62}N_4O_2$ + $H^+$: 571.4951; found: 571.4952.

**3,7,11-Tris(3,4-di(tetradecyloxy)phenyl)tris([1,2,4]triazolo)[4,3-a:4',3'-c:4'',3''- e][1,3,5] triazine *t*-23**

According to the general procedure, 1.00 g (1.75 mmol) 5-(3,4-di(tetradecyloxy)phenyl)-2*H*-tetrazole and 0.10 g (0.5 mmol) cyanuric chloride in 15 mL xylenes with 0.23 mL *sym*-collidine were stirred and heated slowly to 80 °C, his temperature was kept for 10 h. Dilution with water, 20 mL 2N HCl and extraction with ethyl acetate (3 x 20 mL) gave the crude product that was absorbed on 0.5 g silica gel. Chromatography on a alumina column (2*35 cm) with toluene, followed by toluene/ethyl acetate 40/1 gave 0.63 g (0.37 mmol, 70 %) of a colorless wax with m.p. = -10 °C (DSC, 2$^{nd}$ heating)

**$^1$H-NMR** (400 MHz, $CDCl_3$): $\delta$ [ppm] = 7.81 (dd, $^3J$ = 8.6 Hz, $^4J$ = 2.1 Hz, 3H, 6-H, ph), 7.69 (d, $^4J$ = 2.1 Hz, 3H, 2-H, ph), 7.02 (d, $^3J$ = 8.6 Hz, 3H, 5-H, ph), 4.09 (t, $^3J$ = 6.6 Hz, 12H, $OCH_2$), 1.91 – 1.82 (m, 12H, $CH_2$), 1.53 – 1.20 (m, 132H, $CH_2$), 0.89 – 0.86 (m, 18H, $CH_3$). **$^{13}$C-NMR** (75 MHz, $CDCl_3$): $\delta$ [ppm] = 152.37, 151.19 (C-4, ph; C-5, trl), 148.85 (C-3, ph), 140.64 (C-3, trl), 123.92 (C-6, ph), 115.95 (C-1, ph), 115.10 (C-5, ph), 112.56 (C-2, ph), 69.54, 69.20 ($OCH_2$), 32.07, 29.87, 29.81, 29.61, 29.57, 29.52, 29.34, 29.24, 26.19, 26.17, 22.84 ($CH_2$), 14.26 ($CH_3$). **IR** (ATR): $\tilde{\upsilon}$ [cm$^{-1}$] = 3100 w, 2918 vs, 2851 vs, 1597 s, 1491 s, 1467 s, 1260 vs, 1224 s, 1141 s, 1006 m, 907 m, 805 m, 735 s. **FD-MS:** m/z (%): 1703,8 (100) [M$^+$]. **HR-ESI:** calcd. for $C_{108}H_{183}N_9O_6 + H^+$: 1703.4370; found: 1703.4314.

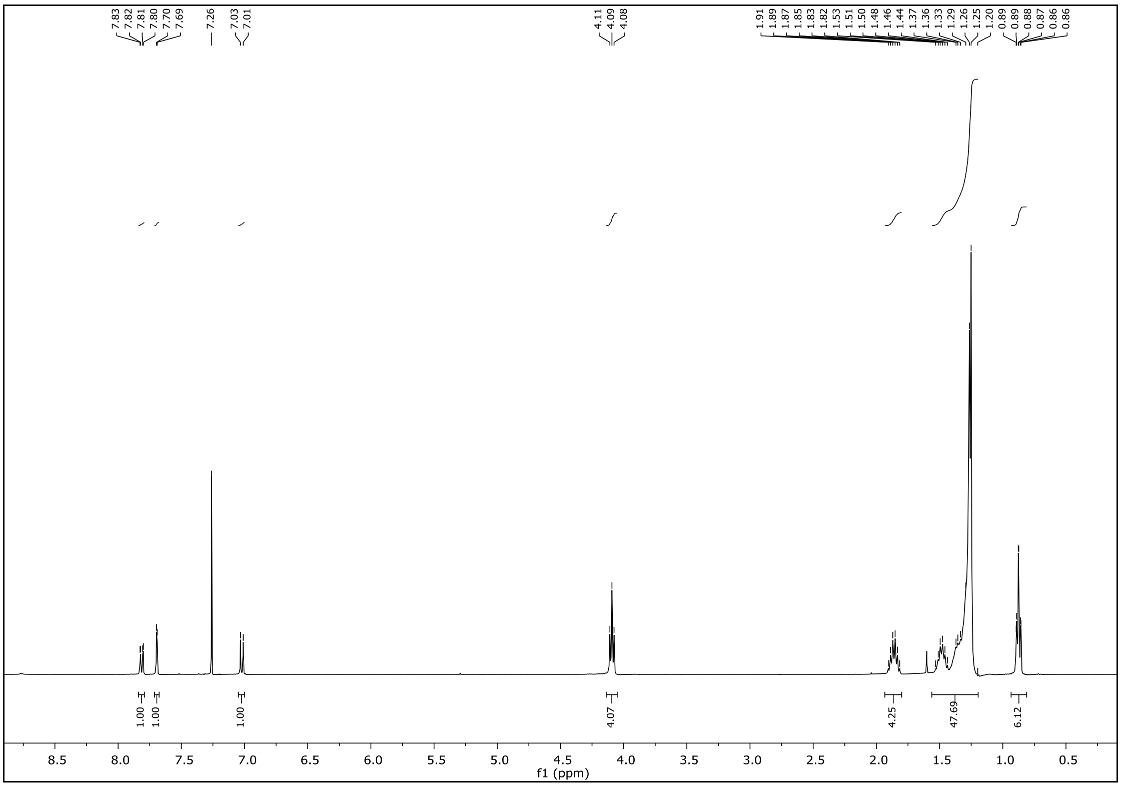

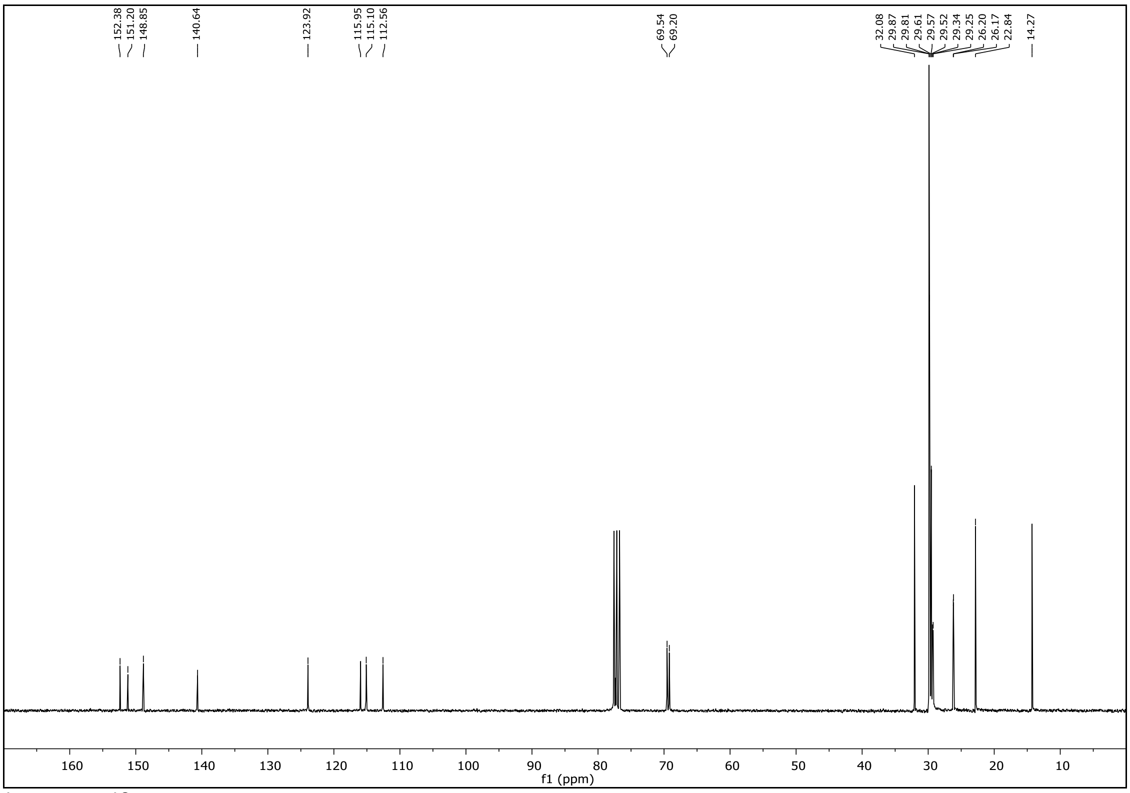


$^{1}$H- and $^{13}$C-NMR of ***t*-23**

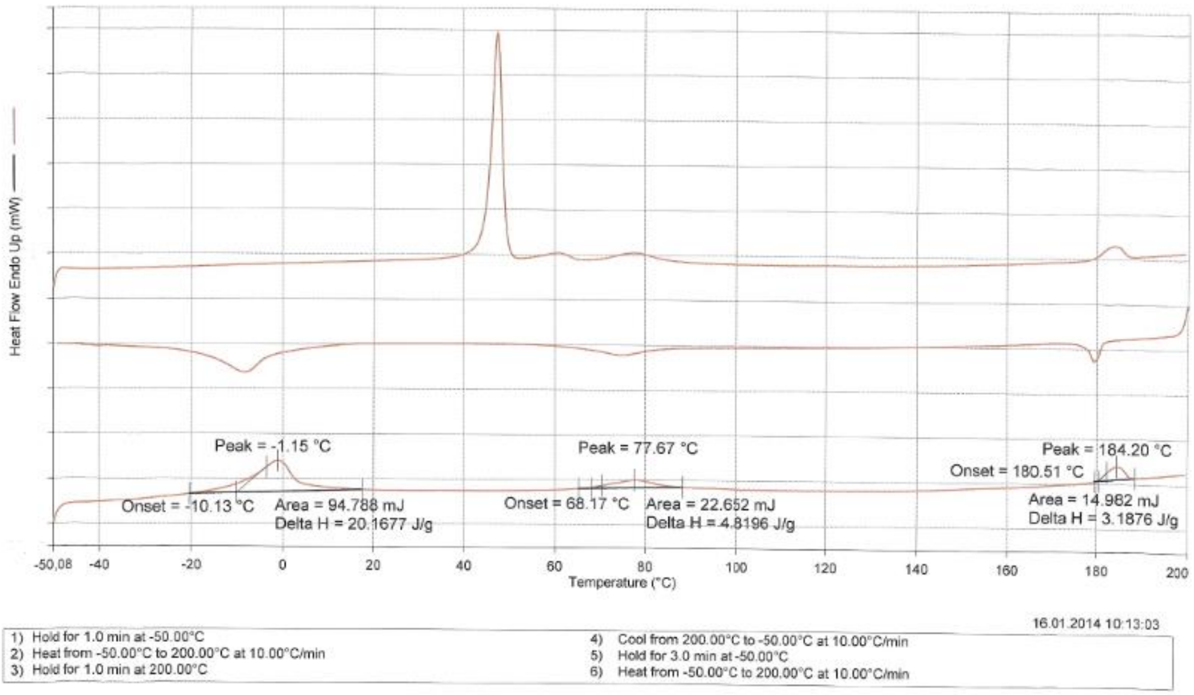


DSC of ***t*-23**

**2,6,10-Tris(3,4-di(tetradecyloxy)phenyl)tris([1,2,4]triazolo)[1,5-a:1',5'-c:1'',5''-e][1,3,5]triazine *r*-23**

200 mg 3,7,11-Tris(3,4-di(tetradecyloxy)phenyl)tris([1,2,4]triazolo)[4,3-a:4',3'-c:4'',3''-e][1,3,5]triazine in 3 mL octadecane was stirred for 90 h unter nitrogen at 275 °C. The mixture was diluted with petroleum ether, filtered through a silica column (1*10 cm) with petroleum ether to elute octadecane followed by ethyl acetate to elute TTT. The crude

product was absorbed on silica gel, chromatography with a gradient from petroleum ether to petroleum ether/toluene = 3/1 yielded 35 mg (18 %) of a colorless solid with m.p. = 128 °C (DSC, 2$^{nd}$ heating).
**$^{1}$H-NMR** (400 MHz, $CDCl_3$): *δ* [ppm] = 8.02 (dd, $^{3}J$ = 8.4, $^{4}J$ = 2.0 Hz, 1H, 6-H, ph), 7.91 (d, $^{4}J$ = 2.0 Hz, 1H, 2-H, ph), 6.99 (d, $^{3}J$ = 8.4 Hz, 1H, 5-H, ph), 4.15 (t, $^{3}J$ = 6.7 Hz, 2H, $OCH_2$), 4.08 (t, $^{3}J$ = 6.7 Hz, 2H, $OCH_2$), 1.90 – 1.84 (m, 4H, $CH_2$), 1.54 – 1.26 (m, 44H, $CH_2$), 0.90 – 0.86 (m, 6H, $CH_3$). **$^{13}$C-NMR** (75 MHz, $CDCl_3$): *δ* [ppm] = 165.45 (C-5, trl), 152.32 (C-4, ph), 149.36 (C-3, ph), 144.62 (C-3, trl), 121.76 (C-6, ph), 120.69 (C-1, ph), 112.88 (C-5, ph), 112.49 (C-2, ph), 69.65, 69.25 ($OCH_2$), 32.09, 29.88, 29.84, 29.80, 29.63, 29.59, 29.54, 29.47, 29.32, 26.22, 26.18, 22.86 ($CH_2$), 14.28 ($CH_3$). **IR** (ATR): $\tilde{\upsilon}$ [$cm^{-1}$] = 2917 s, 2848 s, 1630 s, 1605 m, 1531 w, 1461 s, 1390 m, 1311 w, 1261 s, 1220 s, 1139 s, 1071 w, 866 w, 810 w, 746 s, 721 m. **HR-ESI:** calcd. for $C_{108}H_{183}N_9O_6 + H^+$: 1703.4370; found: 1703.4374.

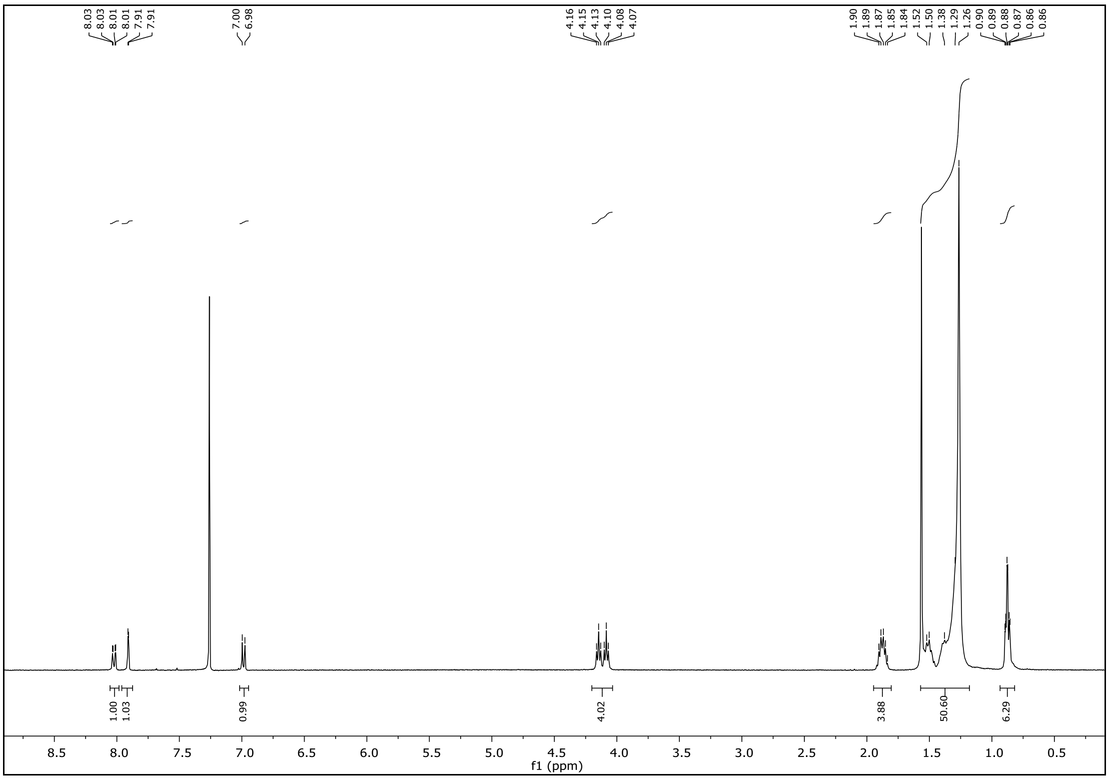

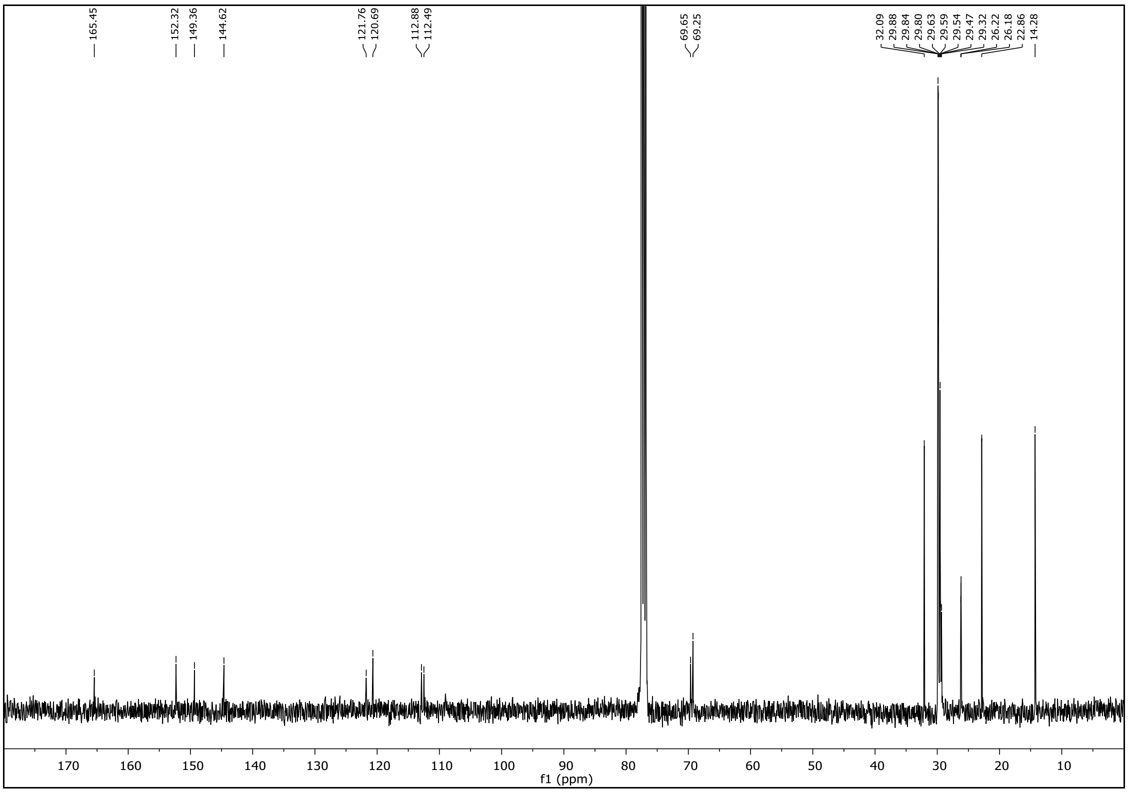


$^1$H- and $^{13}$C-NMR of ***r*-23**

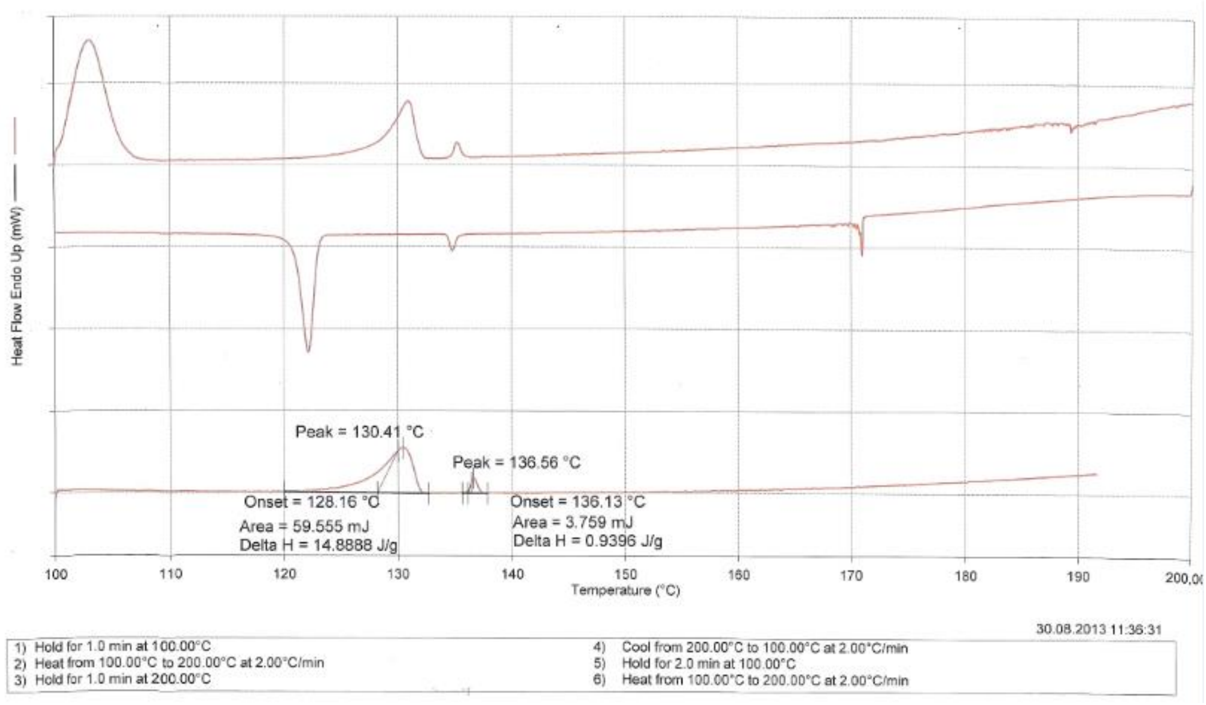


DSC of ***r*-23**

**3,4-Di(hexadecyloxy)benzonitrile**
According to the general procedure, 1.00 g (7.5 mmol) 3,4-dihydroxybenzonitrile, 3.07 g (22 mmol) potash, 6.78 g (22 mmol) 1-bromohexadecane in 50 mL DMF under nitrogen stirred at 80 °C until reaction was finished (TLC). Acidulated with 2 N HCl, extraction with light petroleum (3*30 ml), brine and $MgSO_4$ gave, after recrystallization from propanol-2 3.9 g (6.7 mmol, 90 %) of a colorless solid with m.p. = 88 – 90 °C (i-propanol).
**$^1$H-NMR** (400 MHz, $CDCl_3$): *δ* [ppm] = 7.23 (dd, $^3J$ = 8.3, $^4J$ = 1.9 Hz, 1H, 6-H, ph), 7.07 (d, $^4J$ = 1.9 Hz, 1H, 2-H, ph), 6.86 (d, $^3J$ = 8.4 Hz, 1H, 5-H, ph), 4.04 – 3.96 (m, 4H, $OCH_2$), 1.87 – 1.78 (m, 4H, $CH_2$), 1.50 – 1.19 (m, 52H, $CH_2$), 0.88 (t, 6H, $CH_3$). **$^{13}$C-NMR** (100 MHz, $CDCl_3$): *δ* [ppm] = 153.19, 149.17 (C-3, C-4), 126.44 (CH-ph), 119.61 (C-1), 116.12, 112.81 (CH-ph), 103.61 (C-7, CN), 69.57, 69.24 ($OCH_2$), 32.08, 29.87, 29.82, 29.76, 29.52, 29.49, 29.17, 29.09, 26.09, 22.85, ($CH_2$), 14.28 ($CH_3$). **IR** (ATR): $\tilde{\nu}$ [cm$^{-1}$] = 2924 vs, 2849 vs, 2217 w, 1596 w, 1522 m, 1465 m, 1414 w, 1334 w, 1282 m, 1243 m, 1134 m, 1061 m, 855 w.

**5-(3,4-Di(hexadecyloxy)phenyl)-2*H*-tetrazole**
According to the general procedure, 2.20 g (3.80 mmol) 3,4-di(hexadecyloxy)benzonitrile, 0.86 g (13.00 mmol) $NaN_3$, 1.79 g (13 mmol) $NEt_3HCl$ in 50 mL toluene, 12 h reflux. Then additional 0.25 g (3.7 mmol) $NaN_3$ and 0.51 g (3.7 mmol) triethylammonium chloride, 24 h reflux. Yield: 2.15 g (3.40 mmol, 90 %) of a colorless solid with m.p. = 148 - 150 °C (chloroform).
**$^1$H-NMR** (400 MHz, $CDCl_3$/DMSO-$d_6$): *δ* [ppm] = 7.56 (d, $^4J$ = 2.0 Hz, 1H, 2-H, ph), 7.53 (dd, $^3J$ = 8.3 Hz, $^4J$ = 2.0 Hz, 1H, 6-H, ph), 6.88 (d, $^3J$ = 8.3 Hz, 1H, 5-H, ph), 4.03 – 3.97 (m, 4H, $OCH_2$), 1.91 – 1.64 (m, 4H, $CH_2$), 1.56 – 0.99 (m, 52H, $CH_2$), 0.78 (t, $^3J$ = 6.8 Hz, 6H, $CH_3$). **$^{13}$C-NMR** (75 MHz, DMSO-$d_6$/$CDCl_3$): *δ* [ppm] = 154.96 (C-5, tet), 150.84, 148.73 (C-3, C-4), 119.76 (CH-ph), 116.10 (C-1), 112.68, 111.68 (CH-ph), 68.71, 68.49 ($OCH_2$), 31.27, 29.06, 29.00, 28.98, 28.77, 28.71, 28.58, 28.51, 25.42, 25.37, 22.04 ($CH_2$), 13.55 ($CH_3$). **IR** (ATR): $\tilde{\nu}$ [cm$^{-1}$] = 2918 s, 2849 s, 1607 w, 1510 m, 1470 m, 1271 m, 1243 m, 1128 w, 1065 w, 1043 w, 810 w, 747 w, 718 w. **HR-ESI:** calcd. for $C_{35}H_{62}N_4O_2 + H^+$: 627.5577; found: 627.5558.

**3,7,11-Tris(3,4-di(hexadecyloxy)phenyl)tris([1,2,4]triazolo)[4,3-a:4',3'-c:4'',3''-e][1,3,5] triazine *t*-24**
According to the general procedure, 0.50 g (0.80 mmol) 5-(3,4-di(hexadecyloxy)phenyl)-2*H*-tetrazole and 0.25 mL *sym*-collidine were stirred for 30 min. Addition of 45 mg (0.24 mmol) cyanuric chloride, the mixture was heated slowly to 80 °C, his temperature was kept for 10 h. Dilution with water, 50 mL 2N HCl and extraction with ethyl acetate (3 x 20 mL) gave the crude product that was absorbed on 0.5 g silica gel. Chromatography on a silica column with head of basic alumina with toluene, followed by toluene/ethyl acetate 30/1 gave 0.25 g (0.14 mmol, 56 %) of a colorless wax with m.p. = 19 °C (DSC, 2$^{nd}$ heating).
**$^1$H-NMR** (400 MHz, $CDCl_3$): *δ* [ppm] = 7.81 (dd, $^3J$ = 8.5 Hz, $^4J$ = 2.1 Hz, 3H, 6-H, ph), 7.69 (d, $^4J$ = 2.1 Hz, 3H, 2-H, ph), 7.03 (d, $^3J$ = 8.5 Hz, 3H, 5-H, ph), 4.11 – 4.08 (m, 12H, $OCH_2$), 1.91 – 1.82 (m, 12H, $CH_2$), 1.53 – 1.19 (m, 156H, $CH_2$), 0.87 (t, $^3J$ = 6.6 Hz, 18H, $CH_3$). **$^{13}$C-NMR** (75 MHz, $CDCl_3$): *δ* [ppm] = 152.40, 151.23 (C-4, ph; C-5, trl), 148.87 (C-3, ph), 140.65 (C-3, trl), 123.95 (C-6, ph), 115.95 (C-1, ph), 115.15 (C-5, ph), 112.60 (C-2, ph), 69.58, 69.23 ($OCH_2$), 32.08, 29.88, 29.82, 29.62, 29.58, 29.53, 29.35, 29.26, 26.21, 26.18, 22.85 ($CH_2$), 14.28 ($CH_3$). **IR** (ATR): $\tilde{\nu}$ [cm$^{-1}$] = 2915 vs, 2849 vs, 1610 m, 1575 m, 1529 w, 1466 m, 1260 m, 1226 m, 1445 w, 1033 w, 855 w, 803 w, 720 m, 701 w. **HR-ESI:** calcd. for $C_{120}H_{208}N_9O_6 + H^+$: 1871.6248; found: 1871,6318.

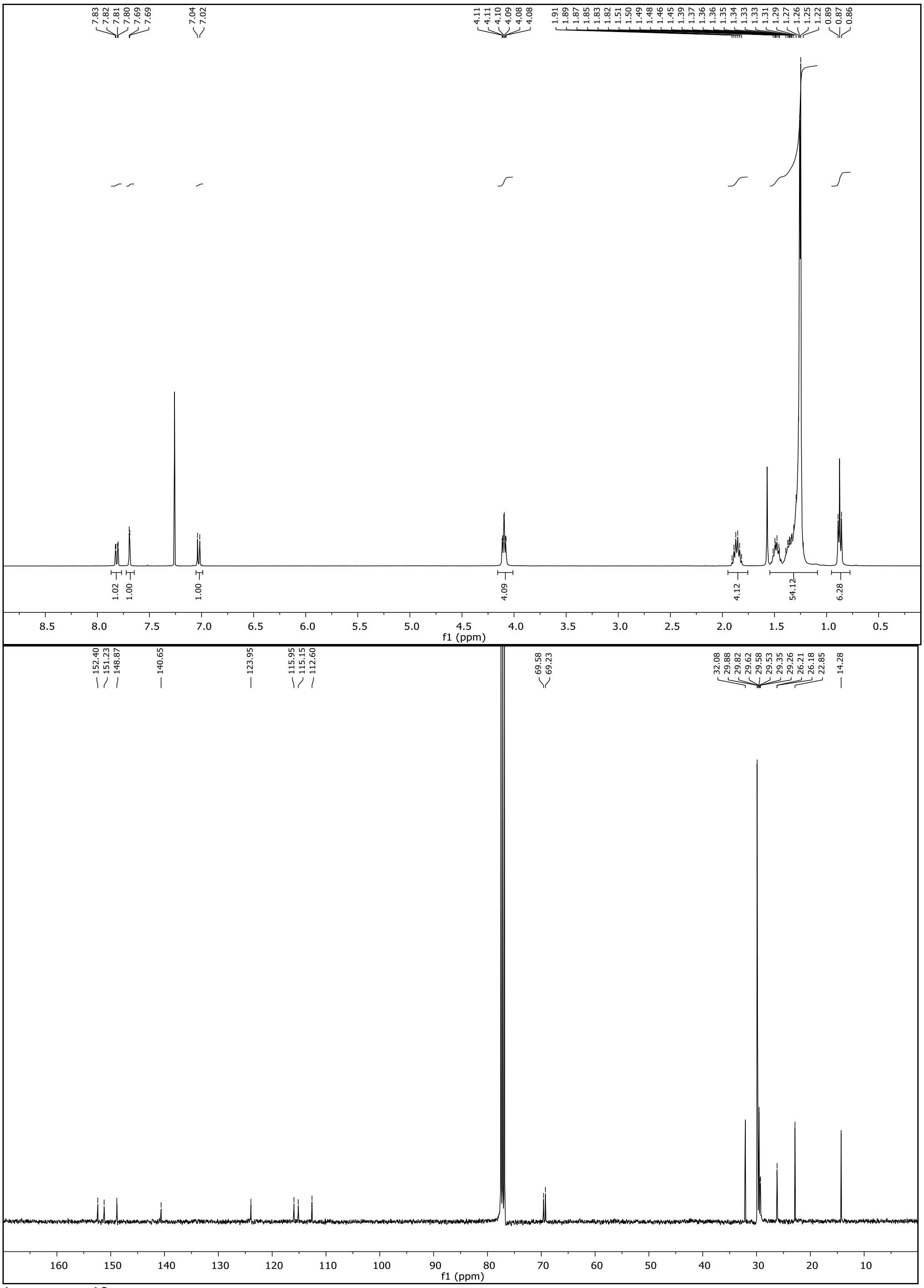

$^1H$- and $^{13}C$-NMR of ***t*-24**

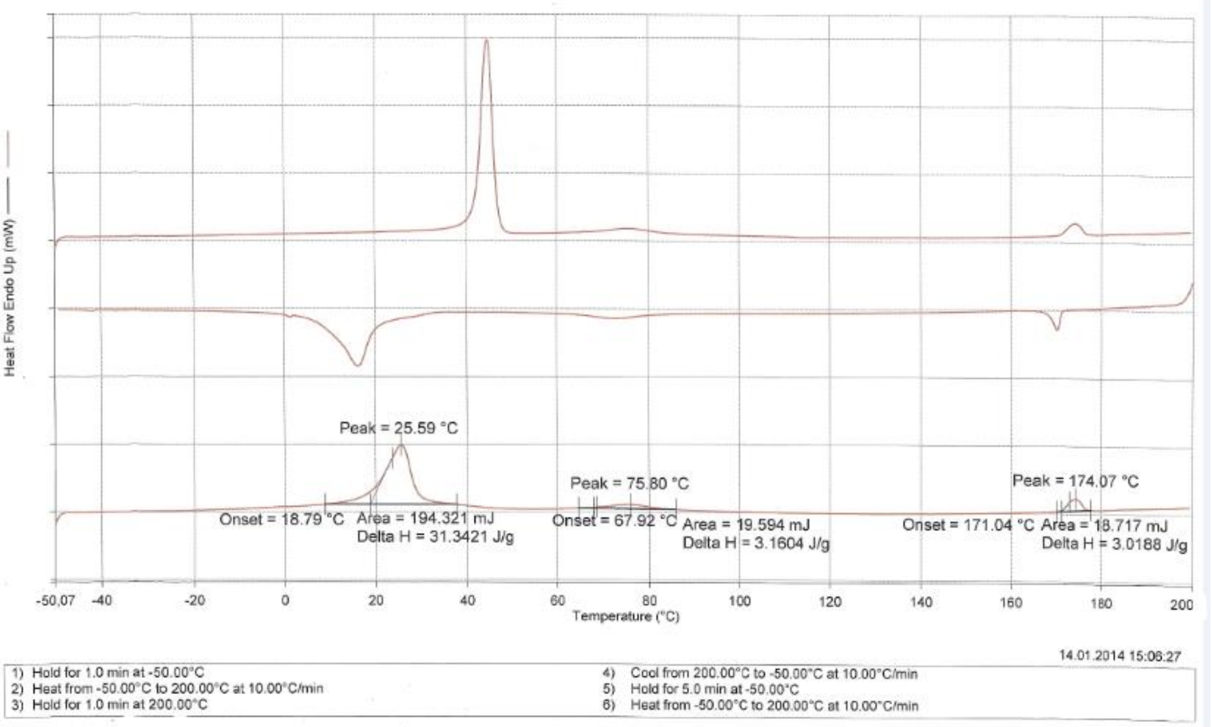


DSC of ***t*-24**

**2,6,10-Tris(3,4-di(hexadecyloxy)phenyl)tris([1,2,4]triazolo)[1,5-a:1',5'-c:1'',5''-e][1,3,5]triazine *r*-24**

150 mg 3,7,11-Tris(3,4-di(hexadecyloxy)phenyl)tris([1,2,4]triazolo)[4,3-a:4',3'-c:4'',3''-e][1,3,5] triazine in 5 mL octadecane was stirred for 75 h unter nitrogen at 275 °C. The mixture was diluted with petroleum ether, filtered through a silica column (1*10 cm) with petroleum ether to elute octadecane followed by ethyl acetate to elute TTT. The crude product was absorbed on silica gel, chromatography with a gradient from petroleum ether to petroleum ether/toluene = 3/1 yielded 32 mg (21 %) of a colorless solid with m.p. = 125 °C (DSC, 2$^{nd}$ heating).

**$^{1}$H-NMR** (600 MHz, $CDCl_3$): $\delta$ [ppm] = 8.01 (dd, $^{3}J$ = 8.3, $^{4}J$ = 2.0 Hz, 1H, 6-H, ph), 7.90 (d, $^{4}J$ = 2.0 Hz, 1H, 2-H, ph), 6.97 (d, $^{3}J$ = 8.3 Hz, 1H, 5-H, ph), 4.14 (t, $^{3}J$ = 6.6 Hz, 2H, $OCH_2$), 4.07 (t, $^{3}J$ = 6.6 Hz, 2H, $OCH_2$), 1.92 – 1.82 (m, 4H, $CH_2$), 1.55 – 1.21 (m, 52H, $CH_2$), 0.89 – 0.86 (m, 6H, $CH_3$). **$^{13}$C-NMR** (150 MHz, $CDCl_3$): $\delta$ [ppm] = 165.38 (C-5, trl), 152.25 (C-4, ph), 149.29 (C-3, ph), 144.57 (C-3, trl), 121.70 (C-6, ph), 120.64 (C-1, ph), 112.76 (C-5, ph), 112.36 (C-2, ph), 69.60, 69.20 ($OCH_2$), 32.09, 29.91, 29.89, 29.85, 29.84, 29.81, 29.64, 29.60, 29.54, 29.45, 29.31, 26.22, 26.17, 22.86 ($CH_2$), 14.29 ($CH_3$). **IR** (ATR): $\tilde{\nu}$ [cm$^{-1}$] = 2917 vs, 2848 s, 1631 s, 1605 m, 1531 w, 1462 s, 1391 m, 1263 s, 1222 s, 1137 m, 1070 w, 864 w, 810 w, 747 s, 722 m. **HR-ESI**: calcd. for $C_{120}H_{207}N_9O_6 + H^+$: 1871.6248; found: 1871.6190.

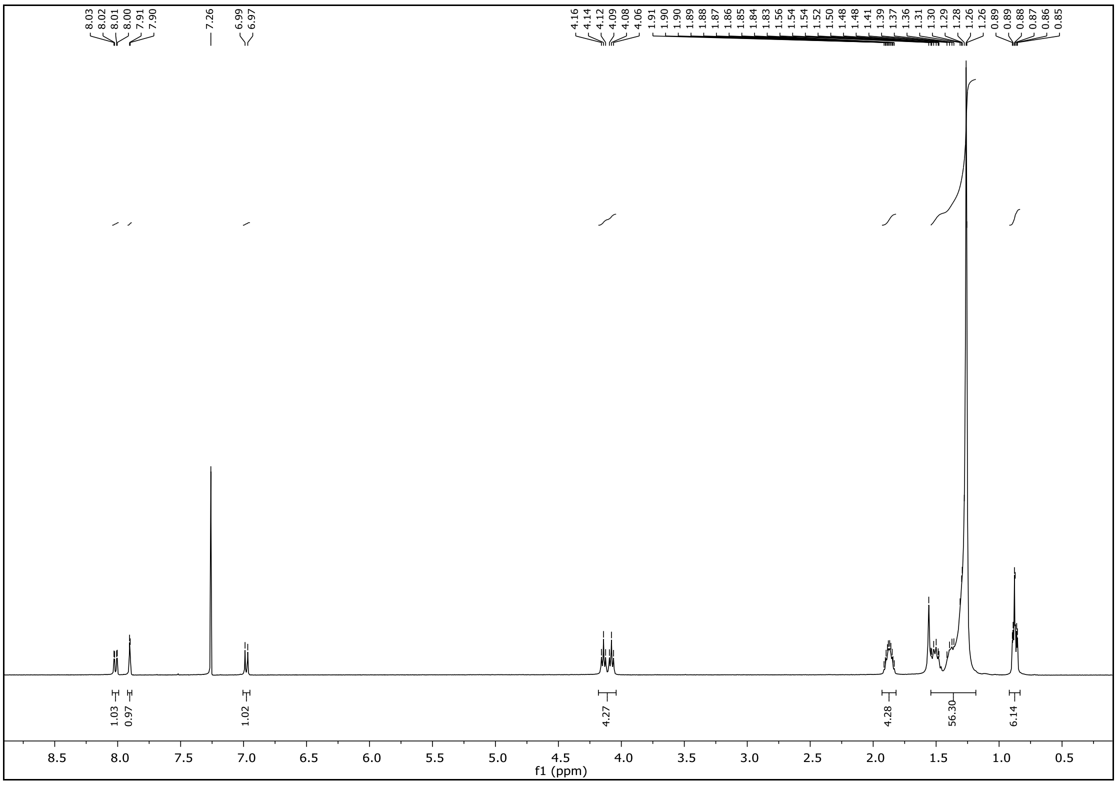


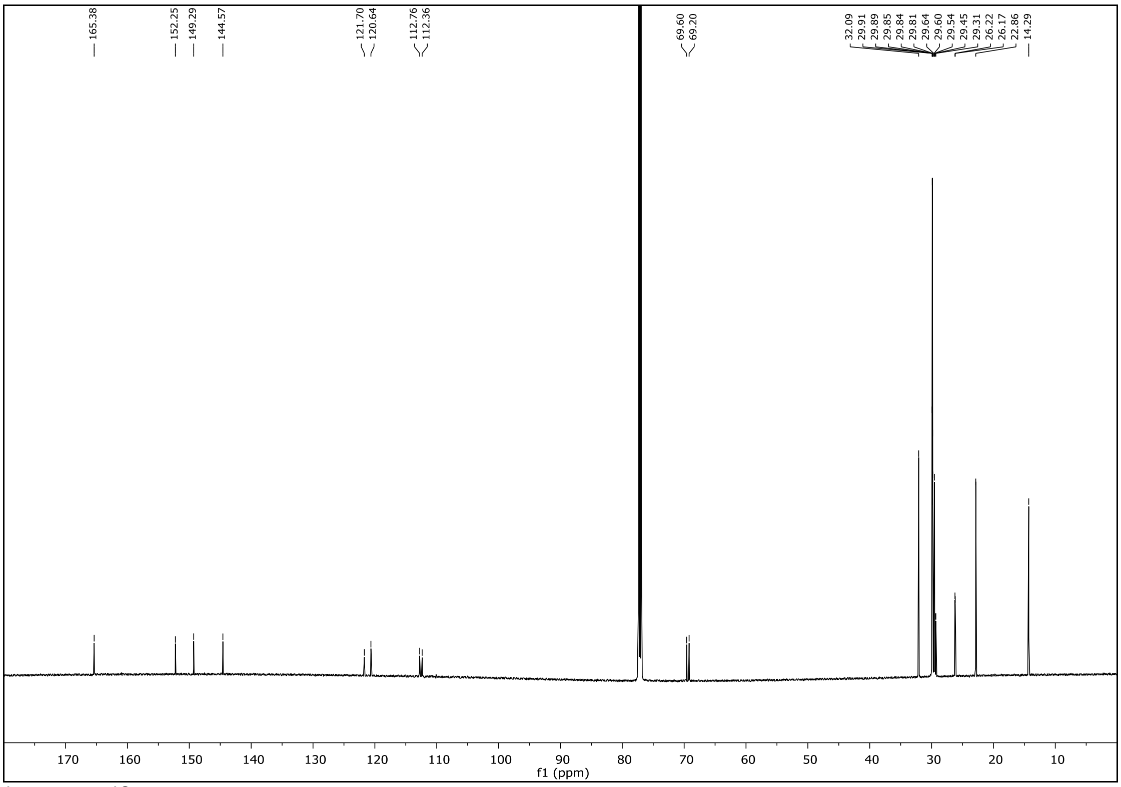


$^1$H- and $^{13}$C-NMR of ***r*-24**

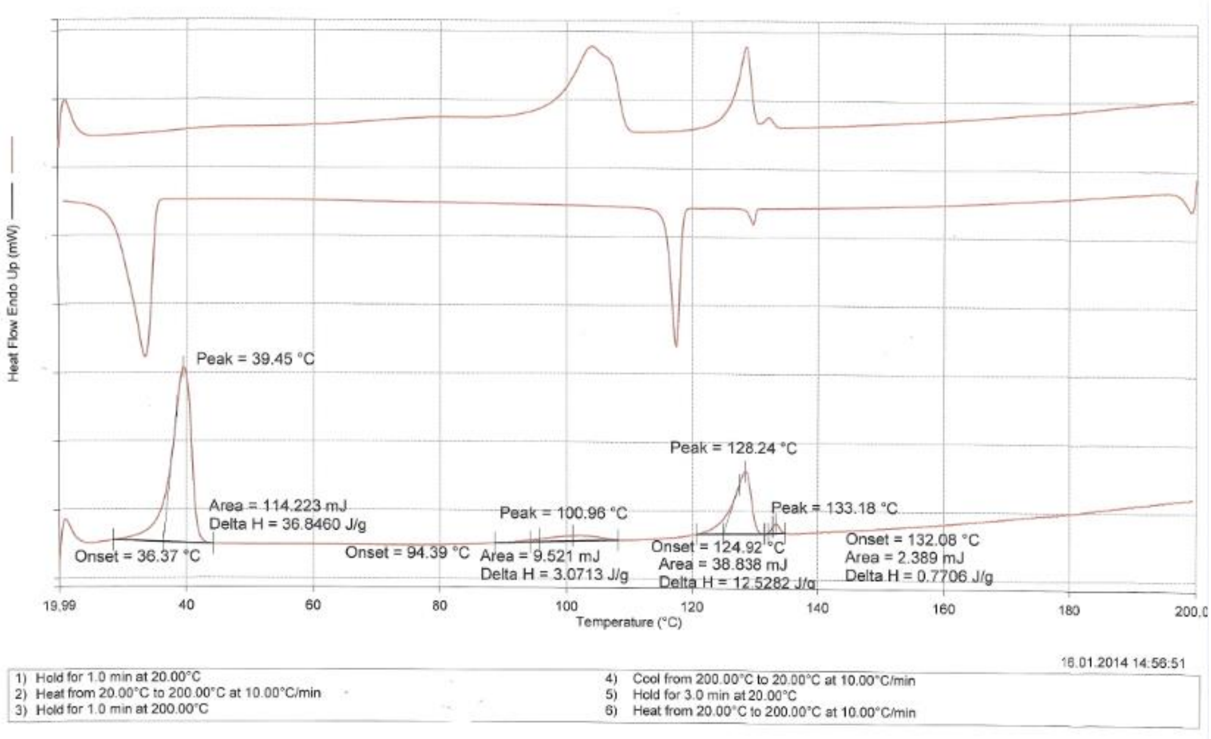


DSC of ***r*-24**

**3,4-Di(octadecyloxy)benzonitrile**
According to the general procedure, 1.00 g (7.5 mmol) 3,4-dihydroxybenzonitrile, 3.07 g (22 mmol) potash, 7.40 g (22 mmol) 1-bromooctadecane in 50 mL DMF under nitrogen stirred at 80 °C until reaction was finished (TLC). Acidulated with 2 N HCl, extraction with light petroleum (3*30 ml), brine and $MgSO_4$ gave, after recrystallization from light petroleum 4.37 g (6.8 mmol, 91 %) of a colorless solid with m.p. = 86 – 88 °C (light petroleum). **$^1$H-NMR** (400 MHz, $CDCl_3$): $\delta$ [ppm] = 7.23 (dd, $^3J$ = 8.3, $^4J$ = 1.9 Hz, 1H, 6-H, ph), 7.07 (d, $^4J$ = 1.9 Hz, 1H, 2-H, ph), 6.86 (d, $^3J$ = 8.4 Hz, 1H, 5-H, ph), 4.04 – 3.96 (m, 4H, $OCH_2$), 1.87 – 1.78 (m, 4H, $CH_2$), 1.50 – 1.19 (m, 52H, $CH_2$), 0.88 (t, 6H, $CH_3$). **$^{13}$C-NMR** (100 MHz, $CDCl_3$): $\delta$ [ppm] = 153.19, 149.17 (C-3, C-4), 126.43 (CH-ph), 119.60 (C-1), 116.11, 112.81 (CH-ph), 103.60 (C-7, CN), 69.57, 69.24 ($OCH_2$), 32.08, 29.86, 29.82, 29.76, 29.74, 29.52, 29.49, 29.16, 29.09, 26.08, 22.84, ($CH_2$), 14.27 ($CH_3$). **IR** (ATR): $\tilde{\nu}$ [cm$^{-1}$] = 2915 vs, 2848 vs, 2221 w, 1597 w, 1518 s, 1467 s, 1422 w, 1336 w, 1278s, 1244 m, 1138 s, 996 w, 810 m, 760 m, 721 m.

**5-(3,4-Di(octadecyloxy)phenyl)-2*H*-tetrazole**
According to the general procedure, 2.00 g (3.10 mmol) 3,4-di(octadecyloxy)benzonitrile, 0.72 g (11.00 mmol) $NaN_3$, 1.51 g (11 mmol) $NEt_3HCl$ in 50 mL toluene, 12 h reflux. Then additional 0.20 g (3.1 mmol) $NaN_3$ and 0.43 g (3.1 mmol) triethylammonium chloride, 24 h reflux. Yield: 1.80 g (2.60 mmol, 85 %) of a colorless solid with m.p. = 144 - 145 °C (chloroform). **$^1$H-NMR** (400 MHz, $CDCl_3$/DMSO-$d_6$): $\delta$ [ppm] = 7.61 – 7.49 (m, 2H, 2-H, 6-H, ph), 6.98 – 6.94 (m, 5-H, ph), 4.02 – 3.96 (m, 4H, $OCH_2$), 1.77 – 1.71 (m, 4H, $CH_2$), 1.48 – 1.15 (m, 60H, $CH_2$), 0.83 – 0.80 (m, 6H, $CH_3$). **$^{13}$C-NMR** (100 MHz, DMSO-$d_6$/$CDCl_3$): $\delta$ [ppm] = 150.85, 148.74 (C-3, C-4), 119.77, 112.70, 111.69 (CH-ph), 68.72, 68.50 ($OCH_2$), 31.28, 29.06, 29.01, 28.79, 28.71, 28.60, 28.52, 25.43, 25.43, 25.39, 22.05 ($CH_2$), 13.56 ($CH_3$). Signals missing due to poor solubility. **IR** (ATR): $\tilde{\nu}$ [cm$^{-1}$] = 2916 s, 2848 s, 1608 w, 1512 m, 1465 m, 1271 m, 1235 m, 1132 w, 1067 w, 812 w, 746 w, 722 w. **HR-ESI:** calcd. for $C_{35}H_{62}N_4O_2$ + $H^+$: 683.6203; found: 638.6195.

**3,7,11-Tris(3,4-di(octadecyloxy)phenyl)tris([1,2,4]triazolo)[4,3-a:4',3'-c:4'',3''-e][1,3,5]triazine *t*-25**

According to the general procedure, 0.40 g (0.60 mmol) 5-(3,4-di(octadecyloxy)phenyl)-2*H*-tetrazole and 0.1 mL *sym*-collidine were stirred for 60 min. Addition of 33 mg (0.18 mmol) cyanuric chloride, the mixture was heated slowly to 80 °C, his temperature was kept until the reaction was complete (TLC). Dilution with water, 50 mL 2N HCl and extraction with ethyl acetate (3 x 20 mL) gave the crude product that was absorbed on 0.5 g silica gel. Chromatography on a silica column with head of basic alumina with toluene, followed by toluene/ethyl acetate 40/1 gave 0.20 g (0.1 mmol, 55 %) of a colorless wax with m.p. = 40 °C (DSC, 2$^{nd}$ heating). **$^1$H-NMR** (400 MHz, $CDCl_3$): $\delta$ [ppm] = 7.82 (dd, $^3J$ = 8.4 Hz, $^4J$ = 2.1 Hz, 3H, 6-H, ph), 7.70 (d, $^4J$ = 2.1 Hz, 3H, 2-H, ph), 7.03 (d, $^3J$ = 8.4 Hz, 3H, 5-H, ph), 4.10 (t, $^3J$ = 6.5 Hz, 12H, $OCH_2$), 1.91 – 1.82 (m, 12H, $CH_2$), 1.58 – 1.14 (m, 180H, $CH_2$), 0.88 (t, $^3J$ = 6.5 Hz, 18H, $CH_3$). **$^{13}$C-NMR** (100 MHz, $CDCl_3$): $\delta$ [ppm] = 152.27, 151.08 (C-4, ph; C-5, trl), 148.73 (C-3, ph), 140.50 (C-3, trl), 123.80 (C-6, ph), 115.80 (C-1, ph), 115.00 (C-5, ph), 112.46 (C-2, ph), 69.42, 69.09 ($OCH_2$), 31.94, 29.74, 29.68, 29.47, 29.44, 29.38, 29.21, 29.12, 26.07, 26.04, 22.71 ($CH_2$), 14.13 ($CH_3$). **IR** (ATR): $\tilde{\upsilon}$ [cm$^{-1}$] = 2916 s, 2849 s, 1582 m, 1468 m, 1265 m, 1145 m, 1010 w, 909 w, 803 m, 792 m, 720 m, 681 m. **HR-ESI:** calcd. for $C_{132}H_{231}N_9O_6 + H^+$: 2039.8126; found: 2039.8199.

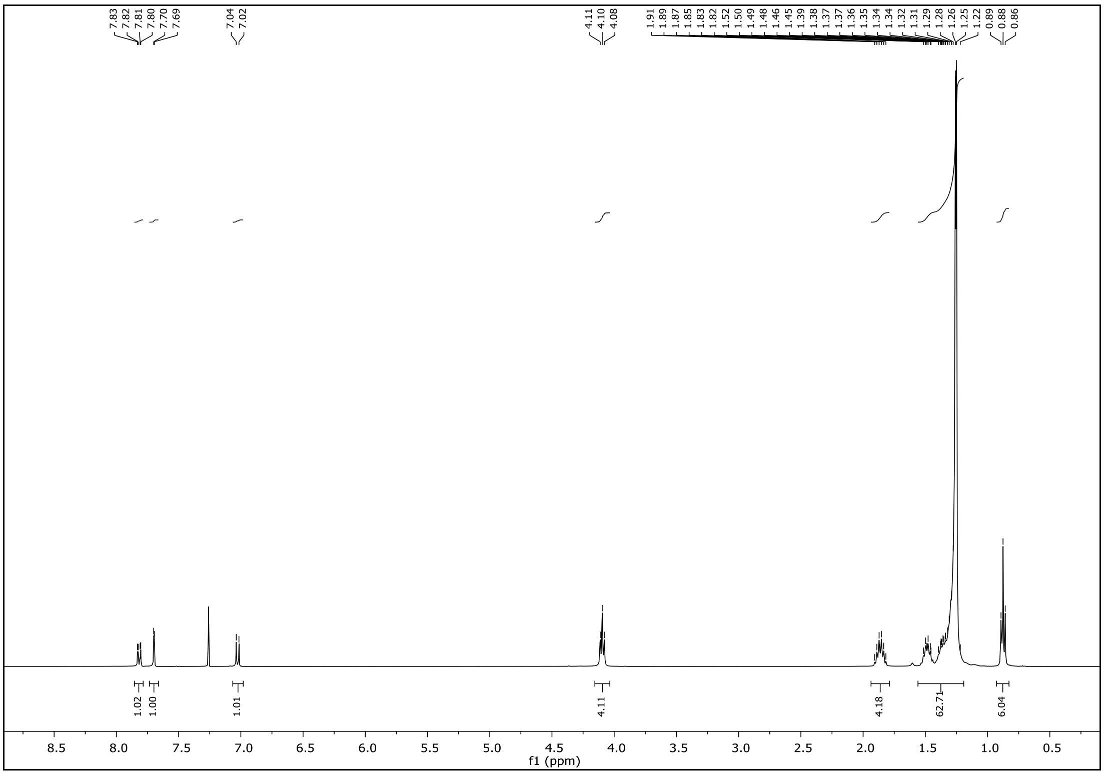

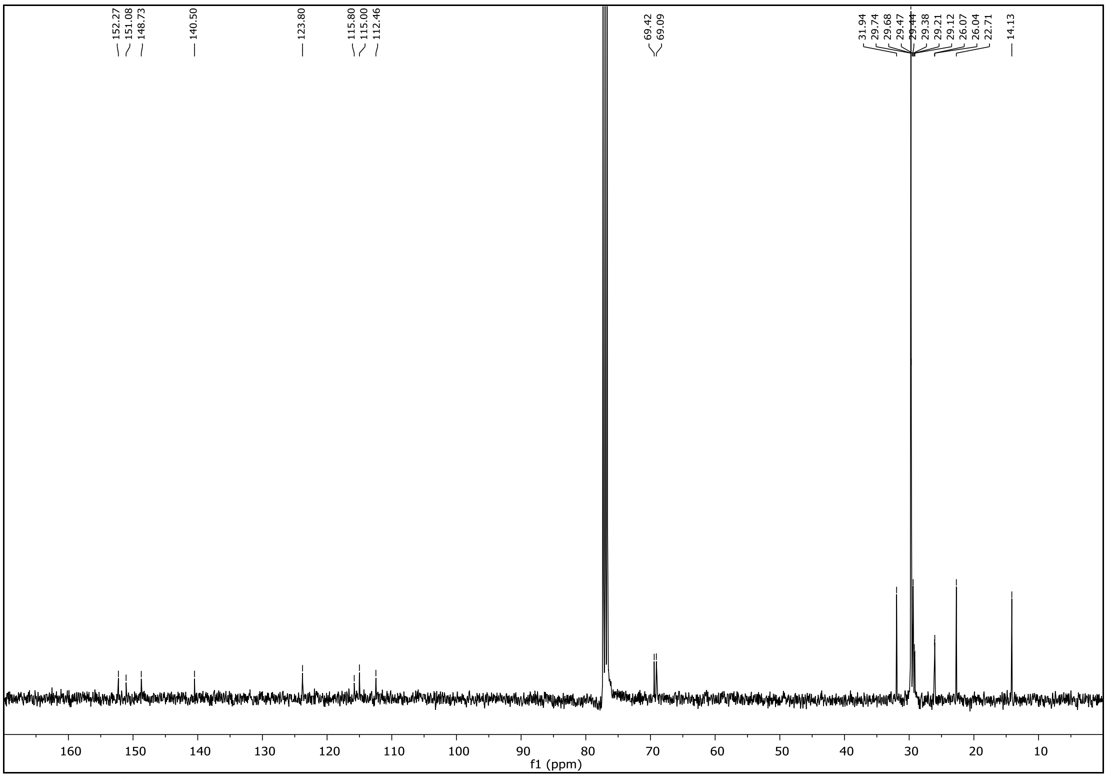


$^1$H- and $^{13}$C-NMR spectra of ***t*-25**

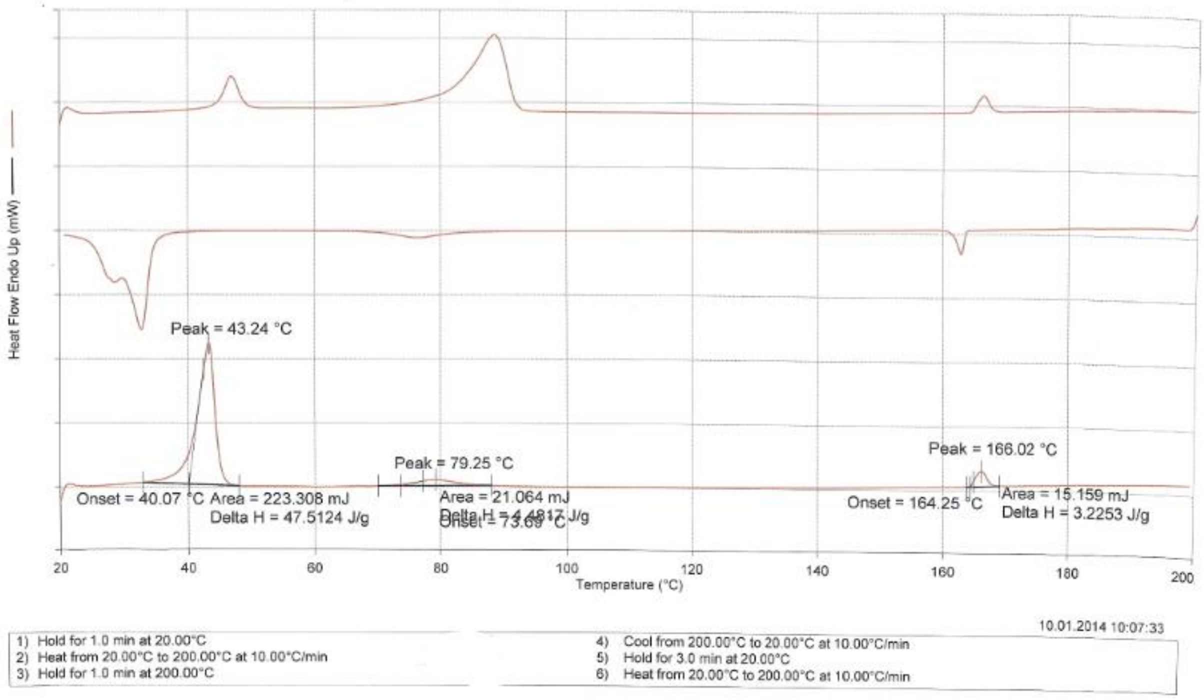


DSC of ***t*-25**

**3,5-Dihexyloxybenzamide**

According to the general procedure, 3,5-dihexyloxybenzoic acid in excess thionyl chloride and toluene gave, after refluxing, evaporation and addition of the solution of the residue in dioxane to aqueous ammonia (25 %, 0 °C) 2.8 g (100%) of a colorless solid with m.p. = 90 - 92 °C. **$^1$H-NMR** (300MHz, DMSO-$d_6$): δ (ppm) = 0.88 (t, 6H, $^3J$ = 6.8 Hz, $CH_3$); 1.26-1.34 (m,

8H, $CH_2$); 1.34-1.47 (m, 4H, $CH_2$); 1.69 (qi, 4H, $^3J$ = 7.0 Hz, $CH_2$); 3.96 (t, 4H, $^3J$ = 6.6 Hz, O-$CH_2$); 6.59 (t, 1H, $^4J$ = 2.4 Hz, C(5)-H); 7.00 (d, 2H, $^4J$ = 1.8 Hz, C(3)-H); 7.34 (s, 1H, $NH_2$); 7.91(s, 1H, $RNH_2$).

**5-(3,5-Dihexyloxyphenyl)tetrazole**
According to the general procedure, 3,5-dihexyloxybenzamide (1.5 g, 4.7 mmol), $NaN_3$ (35.5 mmol, 2.3 g), and $SiCl_4$ (1.4 mL, 11.8 mmol) in acetonitrile gave a brown solid (1.3 g. 81%) with m.p. = 80°C. **$^1$H-NMR** (300MHz, $CDCl_3$): δ(ppm) = 0.89(t, 6H, $^3J$ = 6.6 Hz, CH3); 1.27-1.37(m, 8H, $CH_2$); 1.37-1.49(m, 4H, $CH_2$); 1.75 (qi, 4H, $^3J$ = 6.5, $CH_2$); 3.96 (t, 4H, $^3J$ = 5.9Hz, O-$CH_2$); 6.60 (t, 1H, $^4J$ = 2.2 Hz, C(5)-H); 7.19 (d, 2H, $^4J$ = 2.2Hz, C(3)-H). 13C-NMR (75MHz, $CDCl_3$): δ (ppm) = 14.0 ($CH_3$); 22.6, 25.6, 29.1, 31.5 ($CH_2$); 68.5 (O-$CH_2$); 105.0 (C(5)); 105.5 (C(3)); 125.0 (C(2)); 156.9 (C(1)); 160.9 (C(4)). **IR** (ATR): ν [$cm^{-1}$] = 2953, 2931, 2854, 1601, 1562, 1454, 1166, 1052, 858, 846, 681. **FD-MS**: m/z(%)=347.3(27), 348.3(5) $[M]^{+\cdot}$; 693.5(100), 694.5(42), 695.5(9), 696.5(1) $[2M]^+$.

**3,7,11-Tris(3,5-dihexyloxyphenyl)tris([1,2,4]triazolo)[4,3-a:4',3'-c:4'',3''-e][1,3,5]triazine *t*-26**
**H-NMR** ($CDCl_3$): δ= 0.90 (t, J=6.7 Hz, 18H), 1.28-1.38 (m, 24H), 1.40-1.52 (m, 12H), 1.73-1.86 (m, 12H), 4.02 (t, J=6.5 Hz, 12H), 6.69 (s, 3H), 7.26 (s, 6H). **C-NMR** ($CDCl_3$): δ = 14.4, 22.9, 26.0, 29.5, 31.9, 68.8, 105.8, 108.8, 125.5, 140.8, 151.3, 160.5.

**Tri(3,5-didecyloxyphenyl)2-tris[1,2,4]triazolo[4,3-a:4´,3´-c:4″,3″-e]-[1,3,5]triazine *t*-29**
According to the general procedure, 0.19 g (0.66 mmol) 5-(3,5-di(decyloxyphenyl)-2*H*-tetrazole, 24 mg (0.13 mmol) cyanuric chloride, and 0.5 mL pyridine in 25 mL xylenes were stirred for 30 min and successively heated to 80 . When the reaction was finished (TLC), acidic work-up, extraction with ethyl acetate, evaporation of the solvents and chromatography of the residue through a silica column with toluene/ethyl acetate 40/1 yielded 129 mg (71 %) of a colorless solid with m.p. = 73 °C (DSC). **$^1$H-NMR** (400 MHz, in $C_6D_6$): δ = 0.92 (t(br), 18H, Alkoxy-CH3); 1.21-1.33 (m, 72H, Alkoxy-CH2); 1.36-1.45 (m, 12H, CH2); 1.68-1.76 (m, 12H, CH2); 3.97 (t, 3J=6.4 Hz, 12H, Ar-O-CH2);) 6.98 (t, $^4J$ = 2.2 Hz, 3H, C(5)-H); 7.93 (d, $^4J$=2.2 Hz, 6H, C(3)-H, C(3´)-H). **$^{13}$C-NMR** (75 MHz, in $C_6D_6$): δ = 14.1 (CH3); 22.8, 26.1, 29.4, 29.5, 29.6, 29.7, 29.8, 32.0 (CH2); 68.3 (Ar-OCH2); 105.4 (C(5)); 108.9 (C(3), C(3´)); 126.5 (C(2)); 140.3 (C(1a)); 142.1 (C(5)); 150.3; (C(1b)); 160.7 (C(4), C(4´)). **IR** (ATR): $\tilde{\nu}$ [$cm^{-1}$]= 2921; 2853; 2358; 1594; 1522; 1463; 1435; 1389; 1337; 1272; 1165; 1056; 838; 716; 703; 684; 659. **FD-MS:** m/z (%)= 683.2(17) $[M]^{2+\cdot}$; 1366.4(100), 1367.4 (98), 1368.4 (48), 1369.4 (17) $[M]^{+\cdot}$. **EA:** C84H135N9O6 (1367.07) calcd.: 73.80%C 9.95 %H 9.22%N; found: 73.83 C 9.83 %H 8.69%N.

**5-(3,5-Didodecyloxyphenyl)tetrazole**
According to the general procedure, 3,5-didodecyloxybenzoamide (4,0 g, 8.2 mmol), $NaN_3$ (61.3 mmol, 4.0 g), and $SiCl_4$ (2.3 mL, 20.4 mmol) in acetonitrile gave, after chromatography on silica gel with toluele/ethyl acetate/ triethyl amine 200/200/1, followed by methanol an off-white solid (3.3 mmol, 1.7 g , 40%)
**$^1$H-NMR** (300MHz, $CDCl_3$): δ (ppm) = 0.87 (t, 6H, $^3J$ = 6.1Hz, $CH_3$); 1.15-1.44 m, 36H, $CH_2$); 1.72 (qi, 4H, $^3J$ =7.2 Hz, $CH_2$); 3.92 (t, 4H, $^3J$ = 6.6 Hz, O-CH2); 6.56 (t, 1H, $^4J$ = 2.0 Hz, C(5)-H); 7.22 (t, 1H, $^4J$ = 2.0 Hz, C(3)-H). **$^{13}$C-NMR** (100MHz, $CDCl_3$): δ (ppm) = 14.1($CH_3$); 22.7, 26.0, 29.2, 29.3, 29.4, 29.5, 29.6, 32.0 ($CH_2$); 68.4 (O-CH2); 105.0 (C(5)); 105.5 (C(3)); 125.3 (C(2)); 157.1 (C(1)); 160.9 (C(4)). IR (ATR): ν [$cm^{-1}$] = 2923, 2853, 1599, 1559, 1464, 1389, 1287, 1164, 1056, 841, 683. FD-MS: m/z(%) = 515.4(52), 516.5(14), 517.5(1) $[M]^{+\cdot}$; 1029.9(100), 1030.9(77), 1031.9(23), 1033.0(3) $[2M]^{+\cdot}$.

**2,6,10-Tris(3,5-di(dodecyloxy)phenyl)tris([1,2,4]triazolo)[1,5-a:1',5'-c:1'',5''-e][1,3,5]triazine *r*-30**
100 mg 3,7,11-tris(3,5-di(dodecyloxy)phenyl)tris([1,2,4]triazolo)[4,3-a:4',3'-c:4'',3''-e][1,3,5] triazine in 2 mL octadecane war stirred for 72 h at 290 °C. The cooled mixture was diluted

with light petroleum, filtered through silica and after octadecane had been eluted with petroleum ether, the product was isolated with petroleum ether/toluene 5/1 to 45 mg (45 %) of a colorless solid with m.p. = 126 – 128 °C. **$^{1}$H-NMR** (400 MHz, $CDCl_3$): $\delta$ [ppm] = 7.46 (d, $^{4}J$ = 2.2 Hz, 2H, 2-H, 6-H, ph), 6.47 (t, $^{4}J$ = 2.2 Hz, 1H, ph), 3.94 (t, $^{3}J$ = 6.6 Hz, 4H, $OCH_2$), 1.71 – 1.59 (m, 4H, $CH_2$), 1.45 – 1.21 (m, 36H, $CH_2$), 0.89 (t, 6H, $CH_3$). **$^{1}$H-NMR** (400 MHz, $CDCl_3$): δ (ppm) = 0.88 (t, 18H, $^{3}J$ = 7.0Hz, $CH_3$); 1.18-1.39 (m, 96H, $CH_2$); 1.46 (qi,12H, $^{3}J$ = 7.9 Hz, $CH_2$); 1.80 (qi, 12H, $^{3}J$ = 7.1 Hz, $CH_2$); 4.01 (t, 12H, $^{3}J$ = 6.5 Hz, $OCH_2$); 6.70 (t, 3H, $^{4}J$ = 2.1Hz, C(5)-H); 7.25 (d, 6H, $^{4}J$ = 2.0Hz, C(3)-H). **$^{13}$C-NMR** (75 MHz, $CDCl_3$): $\delta$ [ppm] = 164.76 (C-5, trl), 160.63 (C-3, C-5, ph), 144.14 (C-3, trl), 129.76 (C-1, ph), 105.68 (C-2, C-6, ph), 104.96 (C-4, ph), 68.62 ($OCH_2$), 32.11, 29.91, 29.89, 29.87, 29.83, 29.68, 29.57, 29.39, 26.17, 22.87 ($CH_2$), 14.29 ($CH_3$). **$^{13}$C-NMR** (75 MHz, $CDCl_3$): δ(ppm)=14.1($CH_3$); 22.7, 26.0, 29.2, 29.3, 29.4, 29.6, 29.7, 31.9 (CH2); 68.4 (O-$CH_2$); 105.4 (C(5)); 108.5 (C(3)); 125.1 (C(2)); 140.5 (C(1a)); 150.9 (C(1b)); 160.2 (C(4)). **IR** (ATR): $\tilde{\upsilon}$ [$cm^{-1}$] = 2917 s, 2850 s, 1627 s, 1600 s, 1507 m, 1467 s, 1436 m, 1390 m, 1334 m, 1251 w, 1165 s, 1115 w, 1051 m, 832 m, 749 m, 719 m, 679 m. **FD-MS**: m/z(%) = 767.6(4), 768.4(2) $[M]^{2+}$; 1535.5(100), 1536.5(62), 1537.5(12), 1538.5(5) $[M]^{+}$. **HR-ESI:** calcd. for $C_{96}H_{159}N_9O_6 + H^{+}$: 1535.2492; found: 1535.2488.

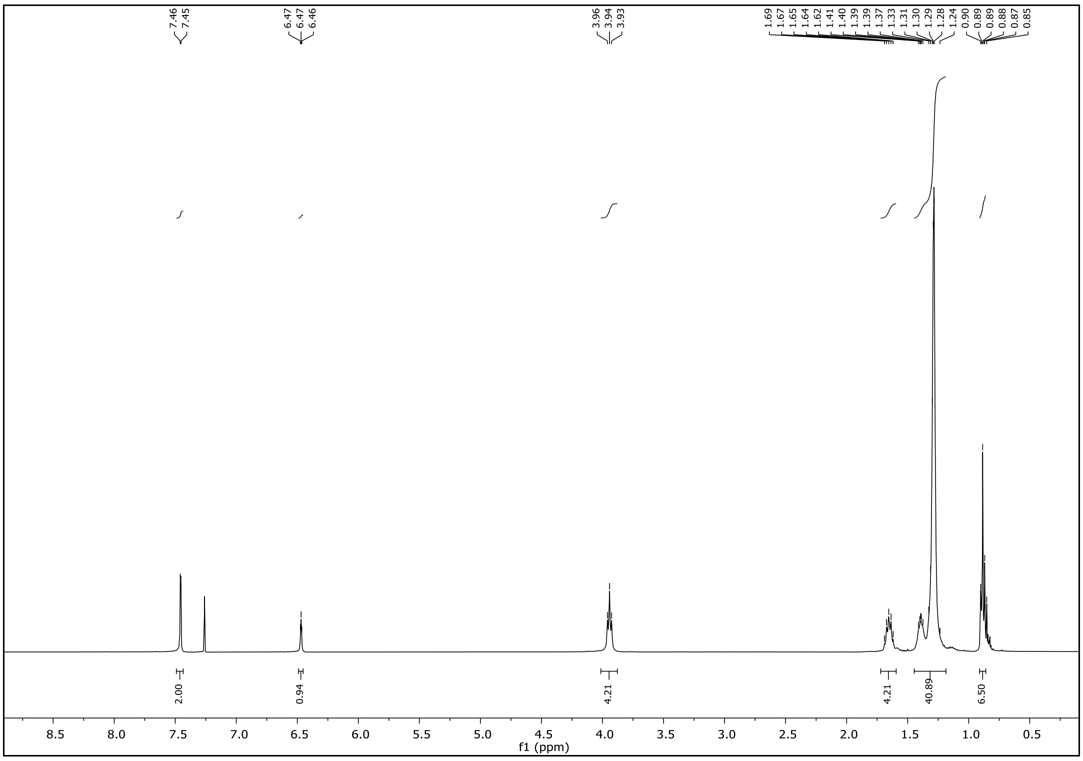

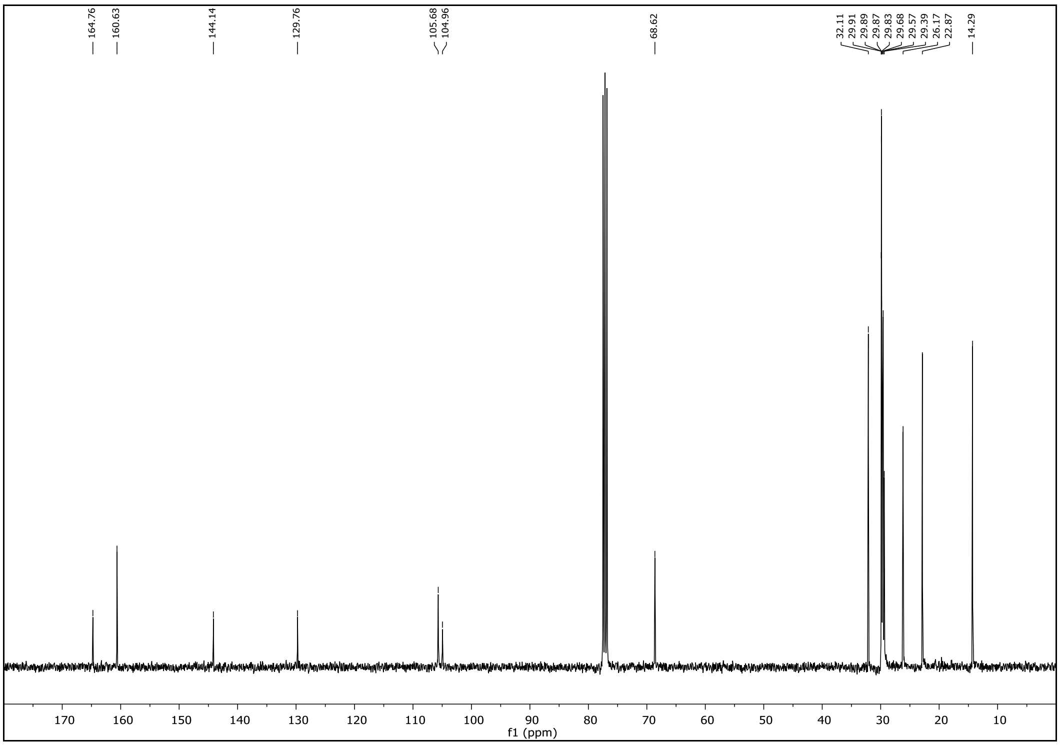


$^1$H- and $^{13}$C-NMR spectra of ***r*-30**

**Methyl 4-bromo-3,5-didodecyloxybenzoate**

4-Bromo-3,5-dihydroxybenzoic acid, prepared according to ref 7, was esterified with methanol and sulfuric acid as catalyst. The crude ester, (31.2 mmol, 7.72 g) was dissolved in DMF, potash (125 mmol, 17.3 g ) and 1-bromodecane (68.8 mmol, 17.1 g) wered added, stirring for 24 h at 80 °C. Thereafter, additional bromodecane (1.5 g) and potash (1.7 g) were added, stirring at 80 °C continued for 24 h. Aqueous work-up, acidulation with 2 N HCl and recrystallization from petroleum ether gave 11.1 g (61%) of a colorless solid with m.p. = 77 - 79 °C. **$^1$H-NMR** (300 MHz, $CDCl_3$): δ = 7.19 (s, 2H, Ar-H), 4.07 (t, $^3J$ = 6.5 Hz, 4H, $-OCH_2$), 3.91 (s, 3H, $-COOCH_3$), 1.92 – 1.76 (m, $OCH_2$-C*$H_2$* 4H), 1.55 – 1.43 (m, 4H, $-CH_2$), 1.40 – 1.23 (m, 32H, $-CH_2$), 0.91 – 0.85 (m, 6H, $-CH_2$-C*$H_3$*). **$^{13}$C NMR** (75 MHz, $CDCl_3$): δ = 166.8 (-*C*$OOCH_3$), 156.7 (3-C, 5-C, Ar-C), 130.0 (1-C, Ar-C), 107.9 (4-C, Ar-CBr), 106.5 (2-C, 6-C, Ar-CH), 69.7 ($-OCH_2$), 52.5 (COO*C*$H_3$), 32.1, 29.8, 29.7, 29.7, 29.5, 29.5, 29.2, 26.1, 22.8 ($-CH_2$), 14.3 ($-CH_2$-*C*$H_3$). **IR** (ATR): $\tilde{\nu}$ [$cm^{-1}$] = 2919 s, 2850 m, 1721 m, 1580 w, 1463 ,m 1427 w, 1382 w, 1332 w, 1239 s, 1117 s, 1003 w. **HR-ESI**: calcd. for $C_{32}H_{56}O_4Br_1$ + $H^+$: 583.3362; found: 583.3374.

**4-Bromo-3,5-didodecyloxybenzoic acid**

Methyl 4-bromo-3,5-didodecyloxybenzoate (19.0 mmol, 11.09 g) and KOH (66.5 mmol, 3.73 g) in 100 mL isopropanol with 50 mL water was refluxed for 2 h and the mainpart of the alcohol was removed in vacuo. The aqueous phase was acidulated (2 N HCl), extracted with ezther (3*40 mL) and the carboxylic acid recrystallized from ethanol to yield 9.63 g (89%) of a colorless solid with m.p. = 55 - 57 °C. **$^1$H-NMR** (300 MHz, $CDCl_3$): δ = 7.26 (s, 2H, Ar-H), 4.09 (t, $^3J$ = 6.5 Hz, 4H, $-OCH_2$), 1.92 – 1.80 (m, 4H, $OCH_2$-C*$H_2$*), 1.50 (m, 4H, $-CH_2$), 1.43 – 1.20 (m, 34H,$-CH_2$), 0.93 – 0.83 (m, 6H-$CH_2$-C*$H_3$*). **$^{13}$C NMR** (75 MHz, $CDCl_3$): δ = 171.5 (-COOH), 156.7 (3-C, 5-C, Ar-C), 128.8 (1-C, Ar-C), 108.9 (4-C, Ar-C), 106.8 (2-C, 6-C, Ar-CH), 69.6 ($-OCH_2$), 31.9, 29.7, 29.7, 29.6, 29.6, 29.4, 29.3, 29.0, 26.0, 22.7 ($-CH_2$), 14.1 (-$CH_2$-$CH_3$). **IR** (ATR): $\tilde{\nu}$ [$cm^{-1}$] = 2919 s, 2853 m, 1687 m, 1581 m, 1425 m, 1383 w, 1327 w, 1266 w, 1226 w, 1120 m, 728 m.

## 4-Bromo-3,5-didodecyloxybenzamide

4-Bromo-3,5-didodecyloxybenzoic acid (16.9 mmol, 9.63 g) in toluene was mixed with (84.5 mmol, 10.1 g, 6.1 mL) thionyl chloride versetzt and refluxed for 12 h. Toluene and residual thionyl chloride were removed in vacuo and the acid chloride was slowly added to concentrated ammonia (-18 °C, 25 %). The solid materisl was isolated, washed and dried; recrystallization from ethanol yielded 8.75 g (91% d.Th.) of a colorless solid with m.p. = 94 – 96 °C. **$^1$H-NMR** (300 MHz, $CDCl_3$): δ = 6.94 (s, 2H, Ar-H), 5.91 (d, *J* = 71.3 Hz, 1H, -$CONH_2$), 4.06 (t, *J* = 6.5 Hz, 4H, -$OCH_2$), 1.92 – 1.77 (m, 4H, -$OCH_2$-C$H_2$), 1.54 – 1.22 (m, 40H, -$CH_2$), 0.96 – 0.79 (m, 6H, -$CH_2$-C$H_3$). **$^{13}$C NMR** (75 MHz, $CDCl_3$): δ = 168.9 (-$CONH_2$), 156.5 (3-C, 5-C, Ar-C), 133.6 (1-C, Ar-C), 105.8 (4-C, Ar-CBr), 104.8 (2-C, 6-C, Ar-CH), 69.5 (-$OCH_2$), 31.8, 29.5, 29.5, 29.4, 29.2, 29.2, 29.0, 25.8, 22.6 (-$CH_2$), 14.0 ($CH_2$-$CH_3$). **IR** (ATR): $\tilde{\nu}$ [$cm^{-1}$] = 3443 m, 3293 w, 3181 w, 2916 s, 2848 m, 1653 ss, 1604 m, 1581 m, 1468 w, 1423 m, 1385 m, 1239 m, 1120 ss, 1039 w, 851 w, 777 w, 719 w, 683 w. **HR-ESI**: calcd. for $C_{31}H_{54}O_3NBr + H^+$: 568.3365; found: 568.3351.

## 5-(4-Bromo-3,5-didodecyloxyphenyl)-2*H*-tetrazole

The tetrazole was prepared from (23.2 mmol, 1.51 g) $NaN_3$, 4-bromo-3,5-didodecyloxybenzamide (3.5 mmol, 2 g), and $SiCl_4$ (7.7 mmol, 1.31 g, 0.89 mL) in 25 mL acetonitrile according to the general procedure. Due to slow conversion , after 25 h, additional $NaN_3$ (0.15 g) and $SiCl_4$ (0.1 mL) were added, stirring for 24 h continued. Aqueours work-up, extraction with dichloromethane and recrystallization from ethanol yielded a colorless solid (14 %, 290 mg) with m.p. = 87 - 88 °C.

**$^1$H-NMR** (300 MHz, $CDCl_3$): δ = 7.30 (s, 2H, Ar-H), 4.04 (t, $^3J$ = 6.4 Hz, 4H, -$OCH_2$), 1.79 (p, $^3J$ = 6.5 Hz, 4H, -$OCH_2$-C$H_2$), 1.51 – 1.38 (m, 4H, -$CH_2$), 1.36 – 1.18 (m, 33H, -$CH_2$), 0.91 – 0.82 (m, 6H, $CH_2$-C$H_3$). **$^{13}$C NMR** (75 MHz, $CDCl_3$): δ = 157.7 (3-C, 5-C, Ar-C), 123.0 (1-C, Ar-C), 106.4 (4-C, Ar-CBr), 104.4 (2-C, 6-C, Ar-CH), 70.0 (-$OCH_2$), 32.1, 29.8, 29.8, 29.8, 29.7, 29.5, 29.2, 26.1, 22.8 (-$CH_2$), 14.3 (-$CH_2$-$CH_3$). Signal of tetrazole C-5 not detected. **IR** (ATR): $\tilde{\nu}$ [$cm^{-1}$] = 2919 s, 2853 m, 1581 w, 1453 m, 1385 m, 1245 m, 1118 m, 1036 w, 844 w, 677 w. **HR-ESI:** calcd. for $C_{31}H_{53}O_2N_4Br + H^+$: 593.3430; found: 593.3434.

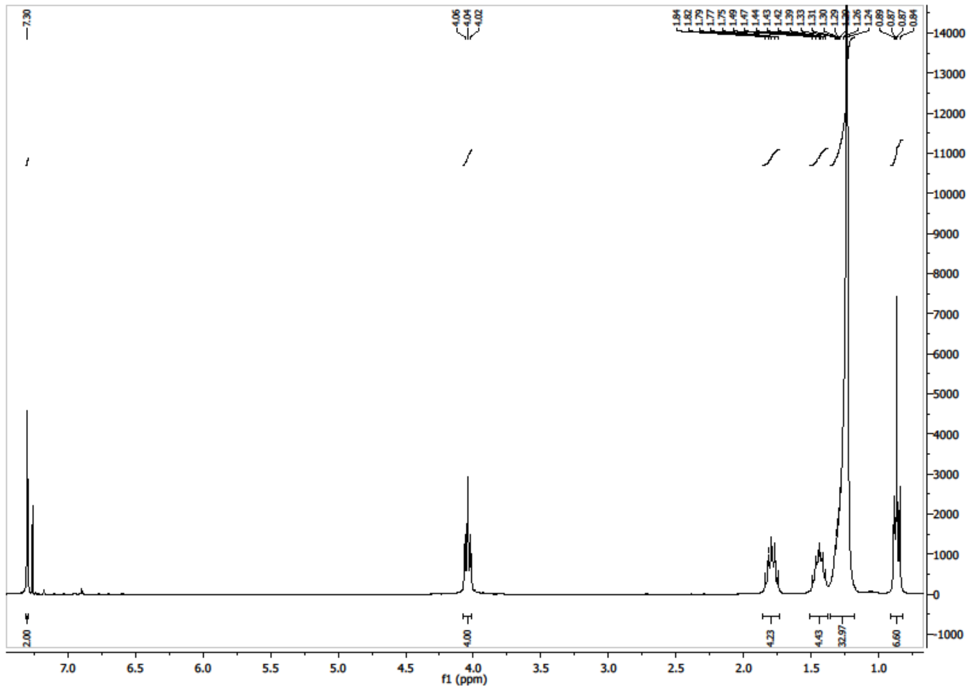

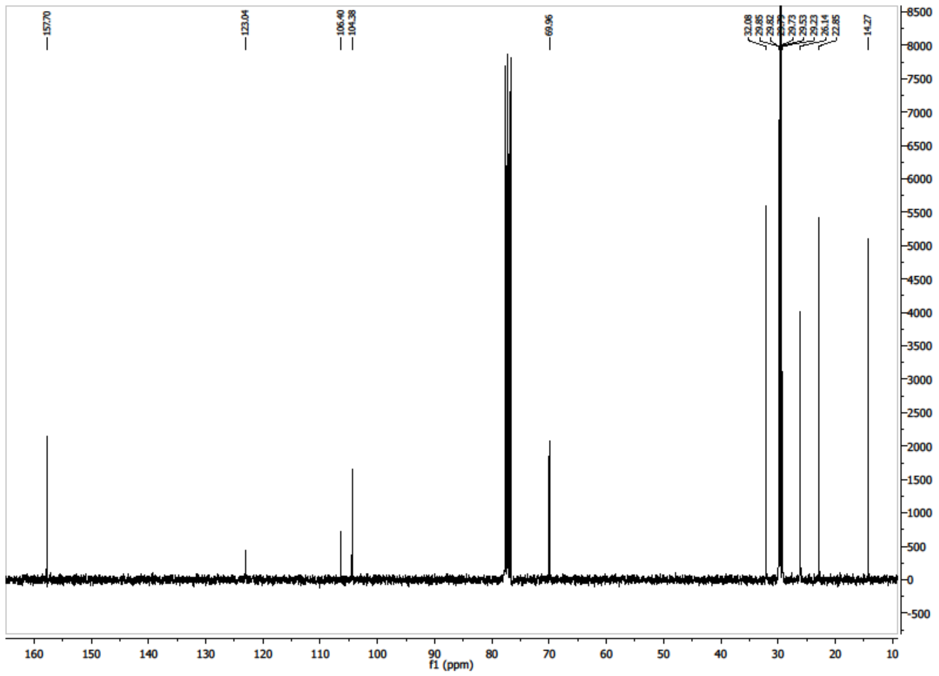


$^1$H- and $^{13}$C-NMR of 5-(4-bromo-3,5-didodecyloxyphenyl)-2*H*-tetrazole

### 3,7,11-Tris(4-bromo-3,5-bis(dodecyloxy)phenyl)tris([1,2,4]triazolo)[4,3-a:4',3'-c:4'',3''-e][1,3,5]triazine *t*-31

5-(4-Bromo-3,5-didodecyloxyphenyl)-2*H*-tetrazole (1.12 mmol, 670 mg), cyanuric chloride (0.37 mmol, 69 mg ) in 20 mL xylenes were mixed with 0.5 mL pyridine. Within 2 h, the temperature of the stirred mixture was raised to 110 °C. After 24 h, aqueous work-up yielded 900 mg of a brown solid. Chromatography on silica gel with toluene/ethyl acetate (20/1) yielded a brownish, waxy solid (240 mg, 36%) with m.p. = 120 °C (DSC). **$^1$H-NMR** (300 MHz, $CDCl_3$): δ = 7.43 (s, 6H, Ar-H), 4.13 (t, $^3J$ = 6.4 Hz, 12H, -$OCH_2$), 1.86 (p, $^3J$ = 6.5 Hz, 12H,$OCH_2$-C*$H_2$*), 1.61 – 1.44 (m, 12H, -$CH_2$), 1.26 (d, *J* = 4.3 Hz, 119H, -$CH_2$), 0.90 – 0.84 (m, 18H, $CH_2$-$CH_3$). **$^{13}$C NMR** (75 MHz, $CDCl_3$): δ = 156.9 (3-C, 5-C, Ar-C), 151.0 (5-C,TTT-C), 140.8 (3-C, TTT-C), 123.4 (1-C, Ar-C), 107.4 (2-C, 6-C, Ar-CH), 106.7 (4-C, Ar-CBr), 69.9 (-$OCH_2$), 32.1, 29.8, 29.8, 29.8, 29.7, 29.5, 29.2, 26.2, 22.8 (-$CH_2$), 14.3 (-$CH_2$-*$C$*$H_3$). **IR** (ATR): $\tilde{\nu}$ [$cm^{-1}$] = 2919 s, 2851 m, 1572 m, 1513 m, 1458 m, 1427 m, 1384 m, 1338 m, 1276 m, 1245 m, 1111 s, 1036 m, 832 m, 719 w, 702 w, 669 w, 655 w. **HR-ESI:** calcd. for $C_{96}H_{156}O_6N_9Br_3$ + $H^+$: 1768.9807; found: 1768.9799.

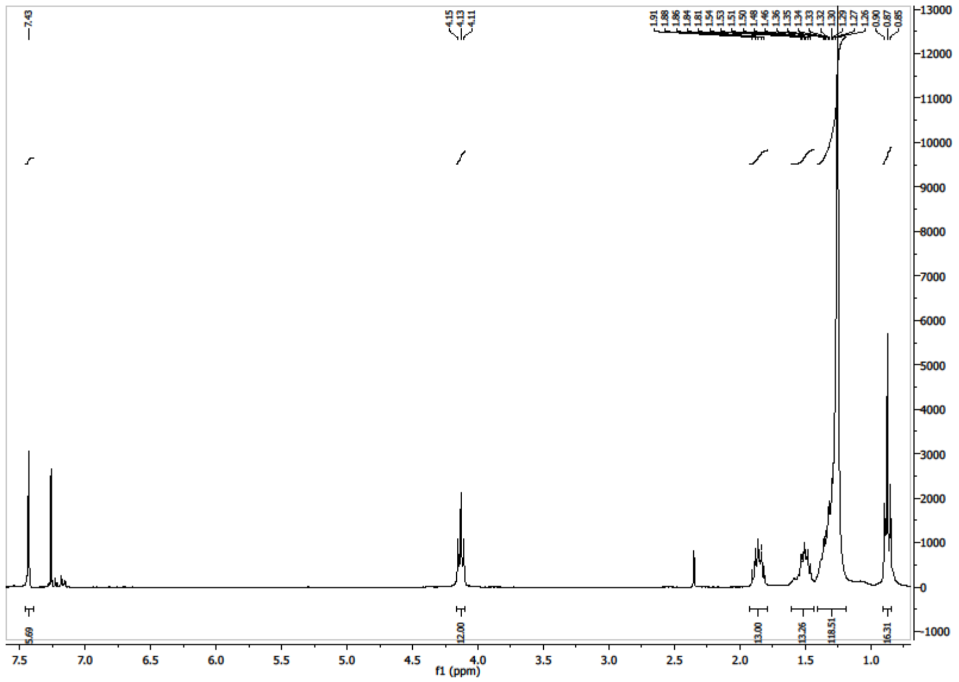

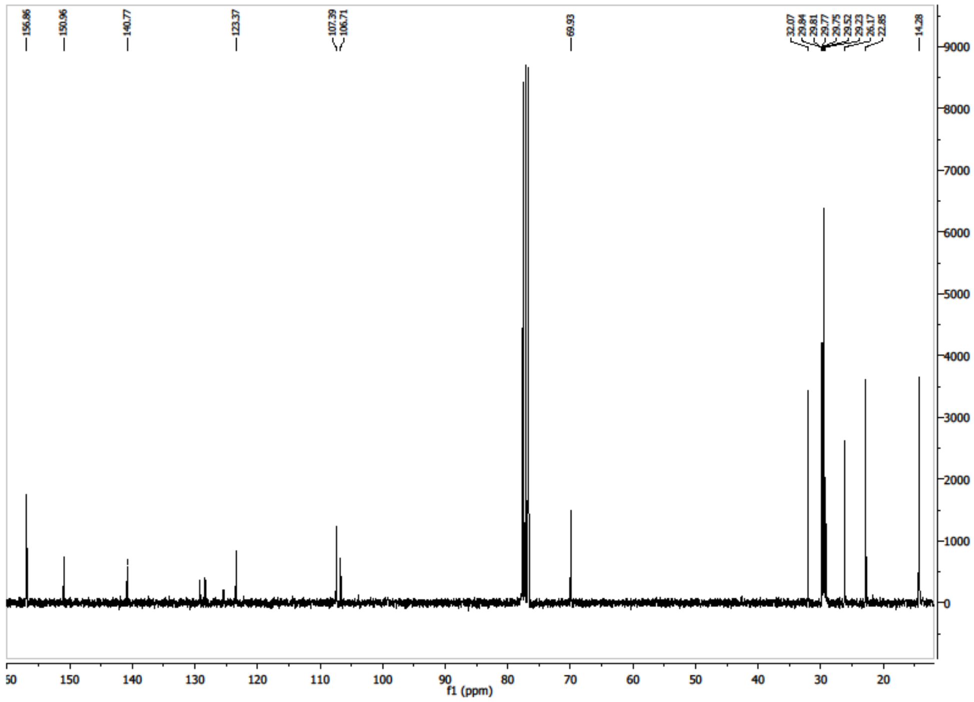

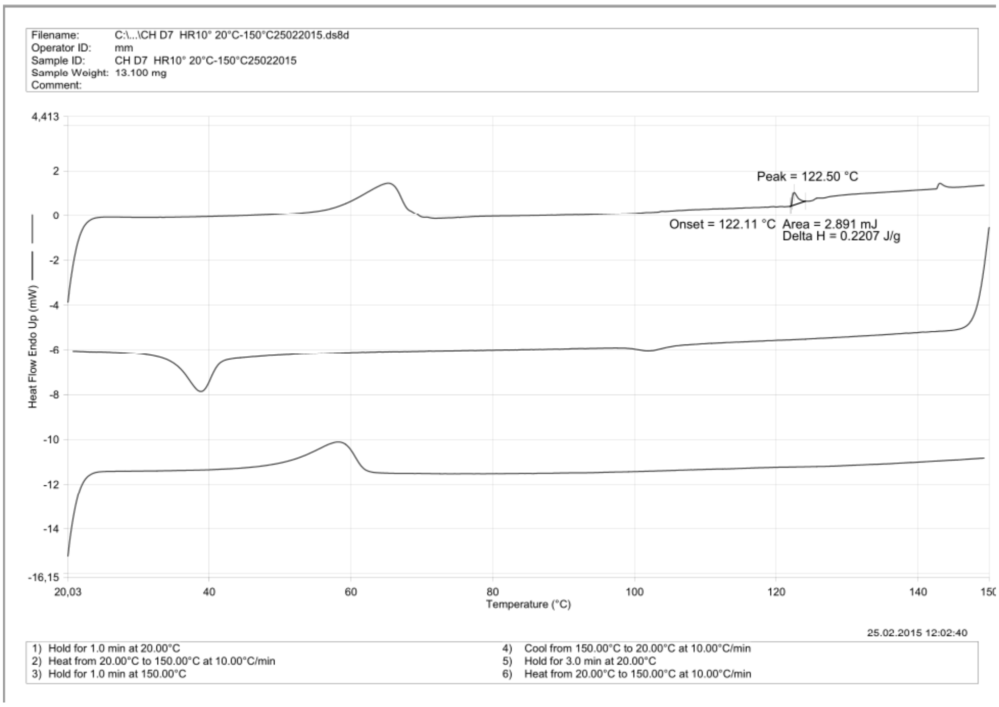


$^{1}$H- and $^{13}$C-NMR and DSC of 3,7,11-Tris(4-bromo-3,5-bis(dodecyloxy)phenyl)tris([1,2,4]triazolo)[4,3-a:4',3'-c:4'',3''-e][1,3,5]triazine ***t*-31**

**3,5-Di(hexadecyloxy)benzoic acid**

Methyl 3,5-di(hexadecyloxy)benzoate (16.3 g), prepared according to GP1, was refluxed in 2-propanol/water (150 mL, 2/1) and KOH (5.2 g, 92.2 mmol) for 14 h.. The cooled mixture was diluted with water, acidulated wit HCl (2n; pH = 2) and solid isolated by suction filtration. Yield: 15.6 g (26.0 mmol, 98%), white soliud with m.p. = 77 °C.

**IR:** (ATR) ṽ = 2916 s, 2848 s, 1692 m, 1612 m, 1468, 1445, 1522, 1396, 1317, 1271, 1168, 1065, 935, 856, 804, 794, 762, 735 $cm^{-1}$. **H-NMR:** (300 MHz, $CDCl_3$) $\delta$ = 7.22 (d, $^4J$ = 2.3 Hz, 2H, H-2, H-6), 6.68 (t, $^4J$ = 2.3 Hz, 1H, 4-H), 3.98 (t, $^3J$ = 6.5 Hz, 6H, OC$H_2$), 1.84 – 1.72 (m, 4H, OCH2C$H_2$), 1.52 – 1.16 (m, 57H, C$H_2$), 0.93 – 0.82 (m, 6H, C$H_3$) ppm.

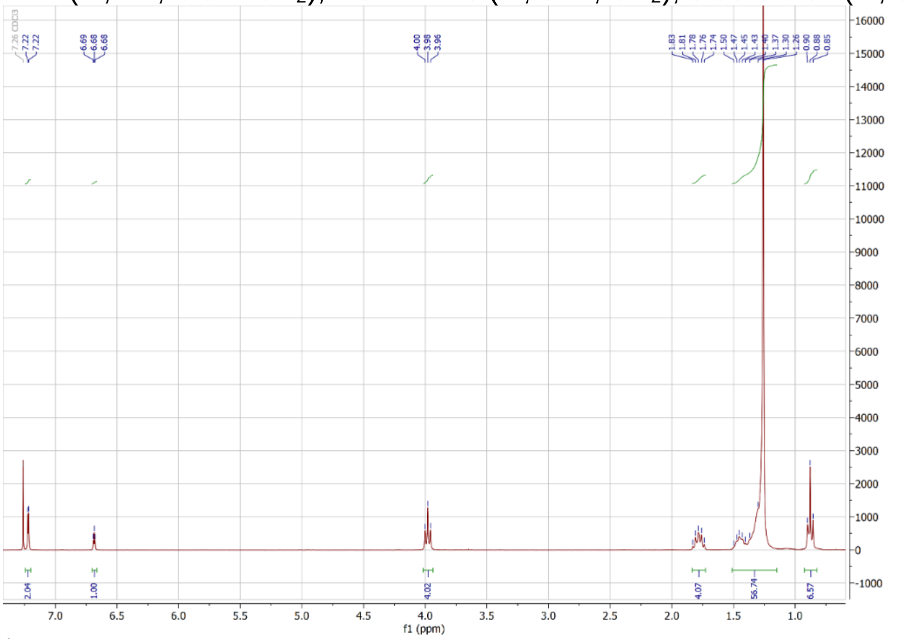


$^{1}$H-NMR (300 MHz, $CDCl_3$) von 3,5-Di(hexadecyloxy)benzoic acid

**3,5-Di(hexadecyloxy)benzoic acid amide**

The amide was prepared from the acid according to the general procedure, yield: 88%, m.p. 92-94 °C after recrystallization from petroleum ether. **IR:** (ATR) ṽ = 3411, 3190, 2914, 2848, 1654, 1600, 1468, 1440, 1406, 1381, 1322, 1299, 1255, 1167, 1103, 1069, 1045, 940, 865, 850, 775 $cm^{-1}$; **$^1$H-NMR:** (300 MHz, $CDCl_3$ + DMSO-*$d_6$*) δ = 6.86 (d, $^4J$ = 2.1 Hz, 2H, H-2, H-6), 6.46 (s, 1H, 4-H), 5.77 (d, br, 2H, N*$H_2$*), 3.86 (t, $^3J$ = 6.5 Hz, 4H), 1.74 – 1.59 (m, 4H, OC*$H_2$*), 1.42 – 1.05 (m, 57H, C*$H_2$*), 0.77 (t, $^3J$=6.5 Hz, 6H, C*$H_3$*) ppm.

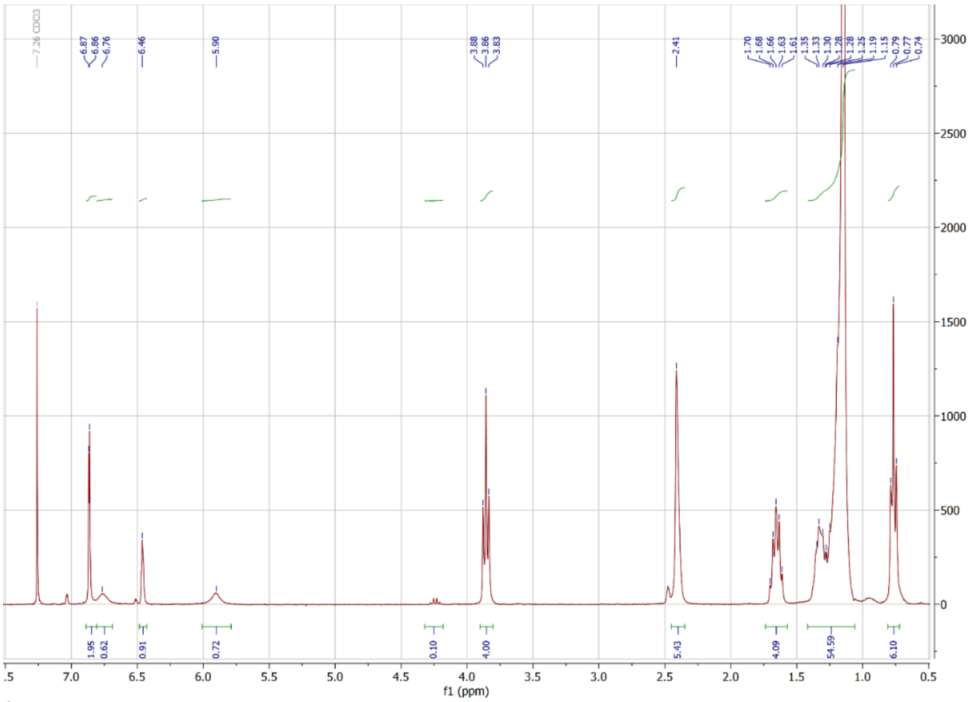

$^1$H-NMR (300 MHz, $CDCl_3$ + DMSO-$d_6$) 3,5-di(hexadecyloxy)benzoic acid amide

**5-(3,5-Di(hexadecyloxy)phenyl)-2*H*-tetrazole**

This compound was prepared according to GP6 with an additional portion of $NaN_3$ and $SiCl_4$. Purification via chromatography, $SiO_2$; toluene:ethyl acetate = 2:1 + 3% Triethylamin → ; toluene:ethyl acetate = 2:1 +1% acetic acid).Yield 30% of a colorless solid with m.p. 83 – 84 °C (Ethanol). IR: (ATR) ṽ = 2915, 1848, 1605, 1568, 1466, 1420, 1391, 1330, 1290, 1265, 1170, 1057, 992, 847, 823, 754, 720 cm-1; 1H-NMR: (300 MHz, $CDCl_3$) δ = 7.24 (d, J = 1.9 Hz, 2H, H-2, H-6), 6.55 (t, 4J = 2.2 Hz, 1H, H-4), 3.98 (t, 3J = 6.6 Hz, 4H, OC*$H_2$*), 1.83 – 1.69 (m, 4H, C*$H_2$*), 1.51 – 1.11 (m, 55H, C*$H_2$*), 0.91 – 0.82 (m, 6H, C*$H_3$*) ppm; **$^{13}$C-NMR:** (75 MHz, $CDCl_3$ + 2 Tropfen DMSO-*$d_6$*) *δ* = 160.74, 125.99, 105.46, 104.24, 68.30 ($OCH_2$), 31.87, 29.65, 29.61, 29.57, 29.55, 29.34, 29.31, 29.15, 25.98, 22.64 $CH_2$), 14.10 (CH3) ppm; FD-MS: m/z (%): 599.1 (10), 600.2 (1) [M-$N_2$]•+; 627.0 (4), 628.1 (11), 629.1 (4) $[M]^{•+}$; 1228.0 (1), 1229.4 (1), 1230.3 (1), 1231.1 (1), 1232.0 (1) $[2M-N_2]^{•+}$; 1253.2 (1), 1254.0 (4), 1255.2 (100), 1256.2 (75), 1257.3 (39), 1258.0 (5) $[M_2]^{•+}$.

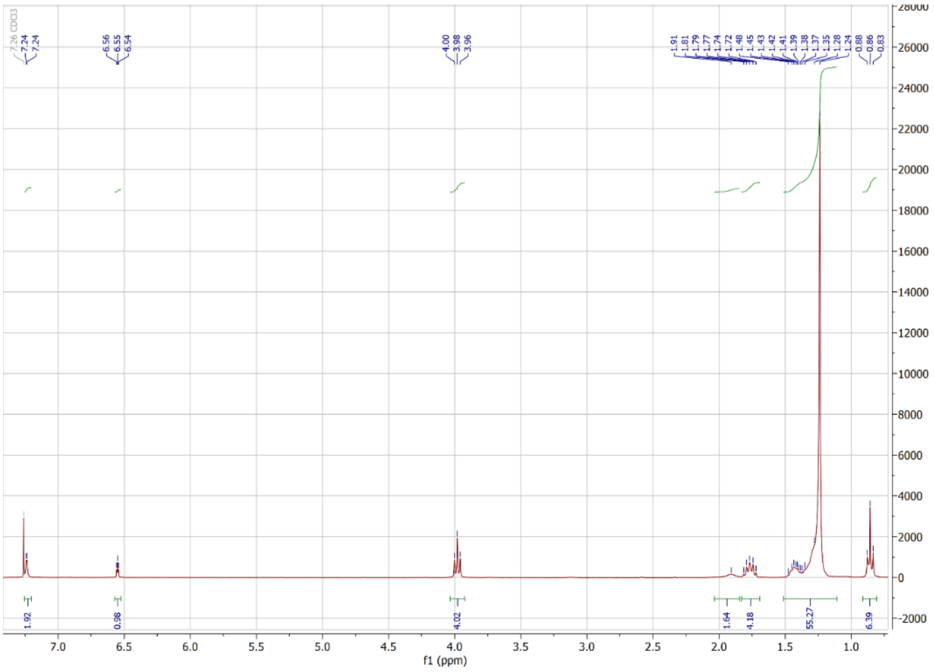


**$^1$H-NMR (300 MHz, $CDCl_3$) 5-(3,5-di(hexadecyloxy)phenyl)-2H-tetrazole**

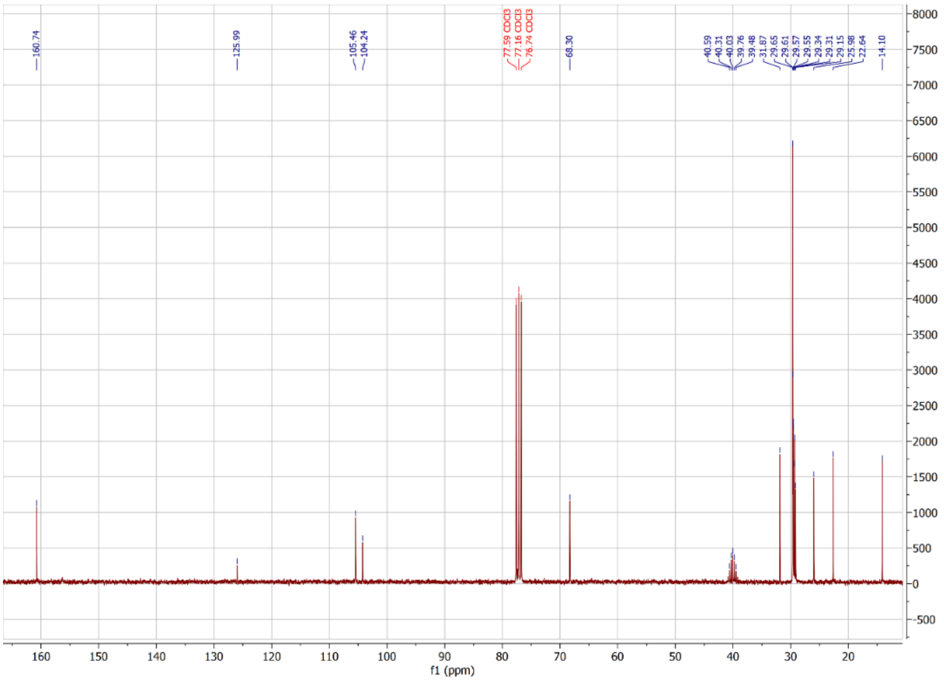


**$^{13}$C-NMR (75 MHz, $CDCl_3$ + 1 drop DMSO-$d_6$) 5-(3,5-di(hexadecyloxy)phenyl)-2H-tetrazole**

### 3,7,11-Tris(3,5-di(hexadecyloxy)phenyl)tris([1,2,4]triazolo)[4,3-a:4',3'-c:4'',3''-e][1,3,5] triazine *t*-32

This TTT was prepared according to the general procedure from the tetrazole and cyanuric chloride, purification via chromatography on $SiO_2$ +5 cm $Al_2O_3$; toluene:ethyl acetate = 40:1 to yield 66% of an off-white solid wit m.p. =70 °C (DSC). **IR:**(ATR) ṽ = 2916, 2849, 1594, 1523, 1465, 1436, 1268, 1164, 1055, 842, 772, 718 cm$^{-1}$; **$^1$H-NMR:**(300 MHz, $CDCl_3$) $\delta$ = 7.25 (d, $^4J$ = 2.2 Hz, 2H, H-2, H-6, ph), 6.70 (t, $^3J$ = 2.2 Hz, 1H, H-4, ph), 4.01 (t, $^3J$ = 6.5 Hz, 4H, OC*H₂*), 1.95 – 1.65 (m, 4H, C*H₂*), 1.52 – 1.16 (m, 62H, C*H₂*), 0.98 – 0.77 (m, 6H, C*H₃*) ppm; **$^{13}$C-NMR:** (75 MHz, $CDCl_3$) $\delta$ = 160.33 (C-3, C-5, ph), 151.07 (C-5, trl), 140.64 (C-3,

trl), 125.24 (C-1, ph), 108.62 (C-4, ph), 105.58 (C-2, C-6, ph), 68.59 ($OCH_2$), 32.08, 29.86, 29.84, 29.81, 29.78, 29.75, 29.58, 29.52, 29.34, 26.19, 22.84 ($CH_2$), 14.27 ($CH_3$) ppm; **FD-MS:** m/z (%): 1872.0 (51), 1873.2 (100), 1873.7 (67), 1874.3 (40), 1875.3 (18), 1875.9 (20) $[M]^{\bullet+}$.

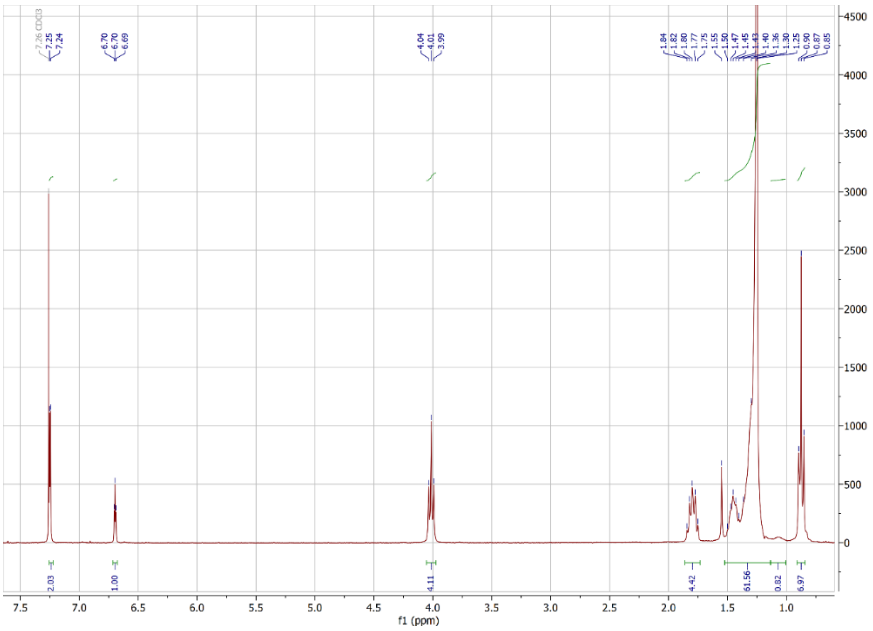

$^{1}$H-NMR (300 MHz, $CDCl_3$) 3,7,11-Tris(3,5-di(hexadecyloxy)phenyl)tris([1,2,4]tri-azolo)[4,3-a:4',3'-c:4'',3''-e][1,3,5]triazine ***t*-32**

.

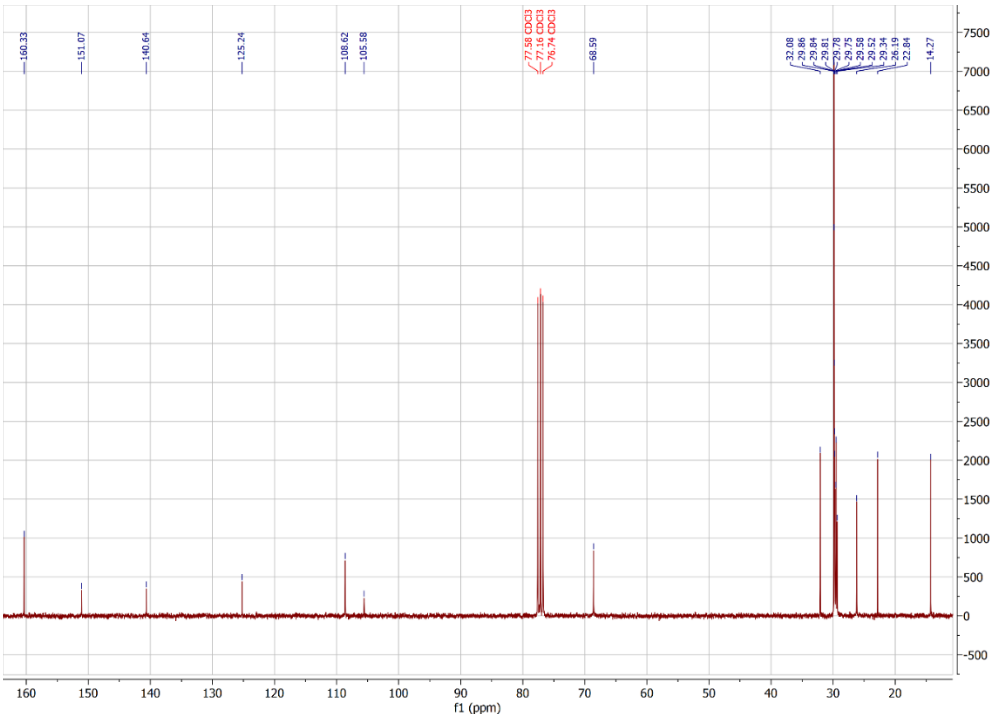

$^{13}$C-NMR (75 MHz, $CDCl_3$)

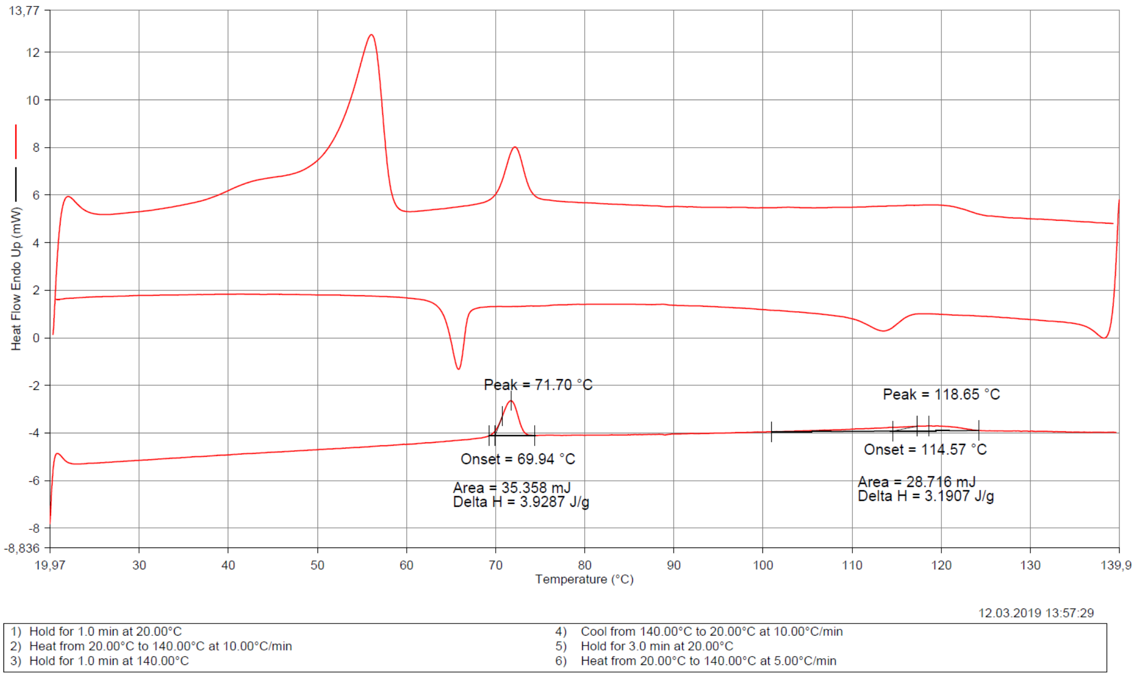


DSC of ***t*-32**

**3,4,5-Trihexyloxy-benzamide**[2] was prepared according to GP2-4 3.96 g (20 mmol) ethyl gallate and 10.73 g (65 mmol) 1-bromohexane in 250 mL dry acetonitrile were treated according to the general procedure. Hydrolysis of the ester with 3.37 g (60 mmol) KOH in 100mL dioxane/isopropanol 1:1 and chlorination with 5.82 mL (80 mmol) thionylchloride in 100 mL abs. toluene followed by ammonolysis yielded 6.57 g (78%) of a colorless solid. **$^1$H-NMR** (400 MHz, DMSO-*d*$_6$): $\delta$ [ppm] = 0.71-0.86 (m, 9H, CH3); 1.14-1.29 (m, 12H, CH2); 1.32-1.43 (m, 6 H, CH2); 1.58-1.77 (m, 6H, CH2); 3.85-3,96 (m, 6H, Ar-O-CH2); 7.02 (s, 2H, C(3)-H, C(3´)-H); 6.10 (s(br), 1H, N-H); 7.15 (s(br), 1H, N-H).

**5-(3,4,5-Trihexyloxyphenyl)-*2H*-tetrazole**[2] 1.11 g (2.75 mmol) 3,4,5-trihexyloxybenzonitrile, 652 mg $NaN_3$ (9.6 mmol) and 1.32 g (9.6 mmol) triethylamine hydrochloride gave according to the general procedure after 7 days 960 mg (78 %), of an off-white solid with m.p. 85 °C. **$^1$H-NMR** (400 MHz, DMSO-*d*$_6$): δ= 0.84-0.91 (m, 9H, $CH_3$); 1.23-1.36 (m, 12H, $CH_2$); 1.37-1.49 (m, 6H, $CH_2$); 1.58-1.68 (m, 2H, $CH_2$); 1.69-1.78 (m, 4H, $CH_2$); 3.90 (t, $^3J$= 6.4 Hz, 2H, Ar-O-CH2); 4.03 (t, $^3J$= 6.4 Hz, 4H, Ar-O-CH2); 7.31 (s, 2H, C(3)-H, C(3´)-H); **$^{13}$C-NMR** (75 MHz, DMSO-*d*$_6$): δ= 13.9 ($CH_3$); 22.2, 25.3, 28.9, 29.8, 31.1, 31.2 ($CH_2$); 68.6, 72,6 (Ar-O-$CH_2$); 105.2 (C(3), C(3´)); 118.9 (C(2)); 139.5 (C(5)); 153.2 (C(4), C(4´)); 155.2 (C(1)); **IR** (ATR): $\tilde{\nu}$ [cm$^{-1}$]= 2923; 2855; 2613; 1586; 1503; 1440; 1402; 1384; 1309; 1250; 1235; 1145; 1112; 1050; 989; 922; 881; 845; 822. **FD-MS:** m/z (%) = 446.2 (100), 447.3 (11) [M]$^{+\cdot}$; 893.4 (11) [2M]$^{+\cdot}$. **HR ESI-MS**: m/z calcd. for $C_{25}H_{42}O_3N_4$+Na: 469.3155, found 469.3162.

**3,7,11-Tris(3,4,5-trihexyloxyphenyl)tris([1,2,4]triazolo)[4,3-a:4',3'-c:4'',3''-e][1,3,5]triazine**[2] ***t*-33**
H-NMR ($CDCl_3$): δ= 0.81-0.91 (m, 27H), 1.23-1.36 (m, 36H), 1.37-1.51 (m, 18H), 1.73-1.83 (m, 18H), 4.04 (t, J=6.4 Hz 18H), 7.42 (s, 6H ). C-NMR (CDCl3): δ= 14.0, 22.6, 22.7, 25.7, 29.2, 30.3, 31.6, 31.7, 69.3, 73.6, 108.4 (CH), 118.2 (Cq), 140.5 (Cq), 141.6 (Cq), 151.1 (Cq), 153.0 (Cq).

**2,6,10-Tris-(3,4,5-trihexyloxyphenyl)tris([1,2,4]triazolo)[1,5-a:1',5'-c:1'',5''-e][1,3,5]triazine *r*-33**
This compound was prepared according to general procedure from 22.1 mg (0.0171 mmol) 3,7,11-tris-(3,4,5-trihexyloxyphenyl)([1,2,4]triazolo)[4,3-a:4',3'-c:4'',3''-e][1,3,5]triazine

31.3 mg (0.0.156 mmol) *p*-bromobenzoic acid and 1.208 g octadecane. The mixture was heated for 22 h at 235 °C and purified via column chromatography. The reaction afforded 7 mg (0.0054 mmol, 32 %) of the desired product. **$^1$H-NMR** (600 MHz, $CDCl_3$) δ = 7.57 (s, 6H), 4.10 (t, $^3J$ = 6.6 Hz, 12H, OC*H₂*), 3.98 (t, $^3J$ = 6.5 Hz, 6H, OC*H₂*), 1.93 – 1.76 (m, 12H, $OCH_2CH_2$), 1.77 – 1.66 (m, 6H, $OCH_2CH_2$), 1.56 – 1.43 (m, 18H, $CH_2$), 1.38 – 1.30 (m, 36H, $CH_2$), 0.94 – 0.84 (m, 18H, $CH_3$). **$^{13}$C-NMR** (151 MHz, $CDCl_3$) δ = 165.10, 153.68, 144.68, 140.99, 123.27, 106.20, 73.88, 69.62, 32.16, 31.95, 30.68, 29.69, 26.14, 23.07, 23.02, 14.49, 14.41. **IR** (neat): ṽ [1/cm] = 2955 s, 2925 s, 2855 s, 1738 w, 1629 s, 1588 m, 1466 ss, 1435 ss, 1381 s, 1332 s, 1230 s, 1113 ss, 986 m, 926 w, 877 w, 849 m, 795 w, 747 s, 690 w, 634 w, 613 w.

**3,7,11-Tris(3,4,5-tri(octyloxy)phenyl)tris([1,2,4]triazolo)[4,3-a:4',3'-c:4'',3''-e][1,3,5]triazine *t*-34**
According to the general procedure, 0.40 g (0.75 mmol) 5-(3,4,5-tri(octyloxyphenyl)-2*H*-tetrazole, 24 mg (0.13 mmol) cyanuric chloride, and 0.1 mL collidine in 25 mL xylenes were stirred for 15 min when a solution of cyanuric chloride (4 mL, 6mg in 6 mL xylenes) was added, stirred for 30 min and successively heated to 80 °C. After 6 h further 0.4 mL of the cyanuric chloride solution was added and heated for 12 h to 80 °C.and successively heated to 80 . When the reaction was finished (TLC), acidic work-up, extraction with ethyl acetate, evaporation of the solvents and chromatography of the residue through a silica column with toluene/ethyl acetate 40/1 yielded 129 mg (71 %) of a colorless solid with m.p. = 73 °C (DSC). Aqueous work-up, chromatography on a column of basic alumina with a head of silica with toluene/ethyl acetate 40/1 yielded 288 mg (0.18 mmol, 72 %) of a colorless solid with **$^1$H-NMR** (400 MHz, $CDCl_3$): *δ* [ppm] = 7.43 (s, 2H, 2-H, 6-H, ph), 4.09 – 4.05 (m, 6H, $OCH_2$), 1.87 – 1.75 (m, 6H, $CH_2$), 1.52 – 1.24 (m, 30H), 0.91 – 0.86 (m, 9H, $CH_3$).

**2,6,10-Tris(3,4,5-tri(octyloxy)phenyl)tris([1,2,4]triazolo)[1,5-a:1',5'-c:1'',5''-e][1,3,5]triazine *r*-34** 110 mg 3,7,11-tris(3,4,5-tri(octyloxy)phenyl)tris([1,2,4]triazolo)[4,3-a:4',3'-c:4'',3''-e][1,3,5]triazine in 2.5 mL octadecane war stirred for 65 h at 275 °C. The cooled mixture was diluted with light petroleum, filtered through silica and after octadecane had been eluted with petroleum ether, the product was isolated with toluene. Chromatography on silica with a gradient staritng with petroleum ether/toluene 10/1 up to 3/1 gave 29 mg (26 %) of a colorless solid with m.p. = 35 °C (DSC, 2$^{nd}$ heating). **$^1$H-NMR** (400 MHz, $CDCl_3$): *δ* [ppm] = 7.59 (s, 2H, 2-H, 6-H, ph), 4.11 (t, $^3J$ = 6.5 Hz, 4H, $OCH_2$), 4.01 (t, $^3J$ = 6.5 Hz, 2H, $OCH_2$), 1.89 – 1.70 (m, 6H, $CH_2$), 1.56 – 1.21 (m, 42H, $CH_2$), 0.89 (t, 9H, $CH_3$). **$^{13}$C-NMR** (100 MHz, $CDCl_3$): *δ* [ppm] = 165.17 (C-5, trl), 153.57 (C-3, C-5, ph), 144.54 (C-3, trl), 141.12 (C-4, ph), 123.01 (C-1, ph), 106.26 (C-2, ph), 73.72, 69.52 ($OCH_2$), 32.10, 32.03, 30.56, 29.74, 29.57, 29.49, 26.30, 22.85 ($CH_2$), 14.27 ($CH_3$). **IR** (ATR): $\tilde{\nu}$ [$cm^{-1}$] = 2922 s, 2853 m, 1627 s, 1587 w, 1466 s, 1435 s, 1381 m, 1332 s, 1227 m, 1113 s, 986 w, 848 w, 747 m, 722 w. **HR-ESI**: calcd. for $C_{96}H_{159}N_9O_9 + H^+$: 1583.2339; found: 1583.2380.

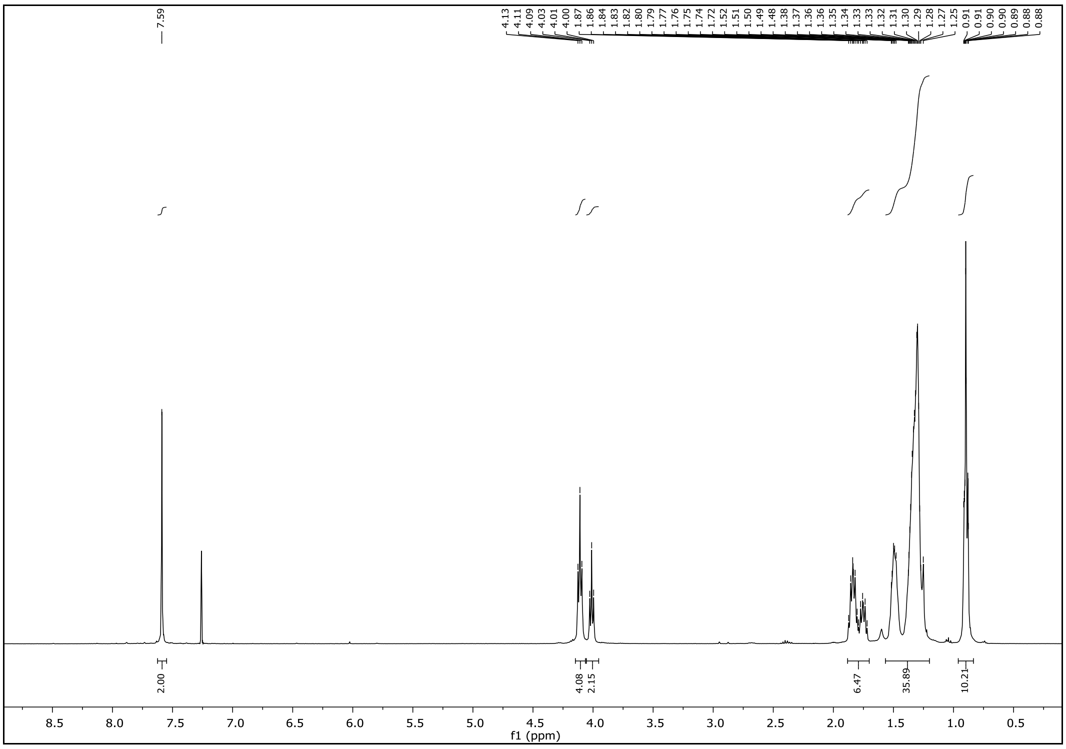

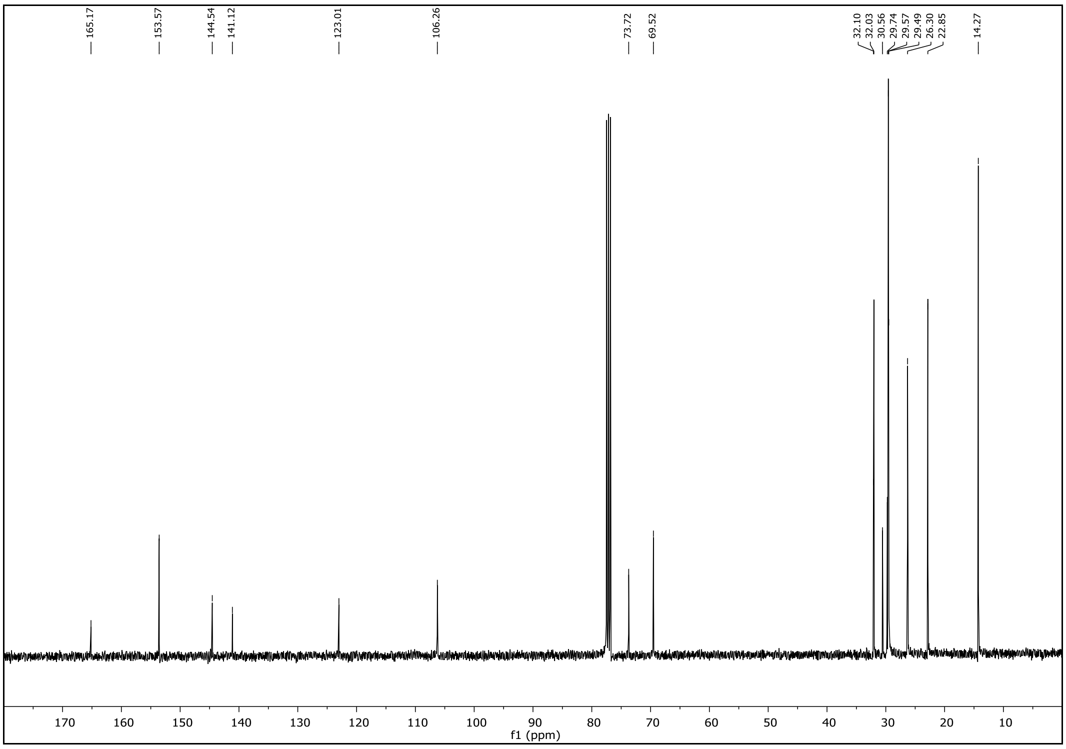

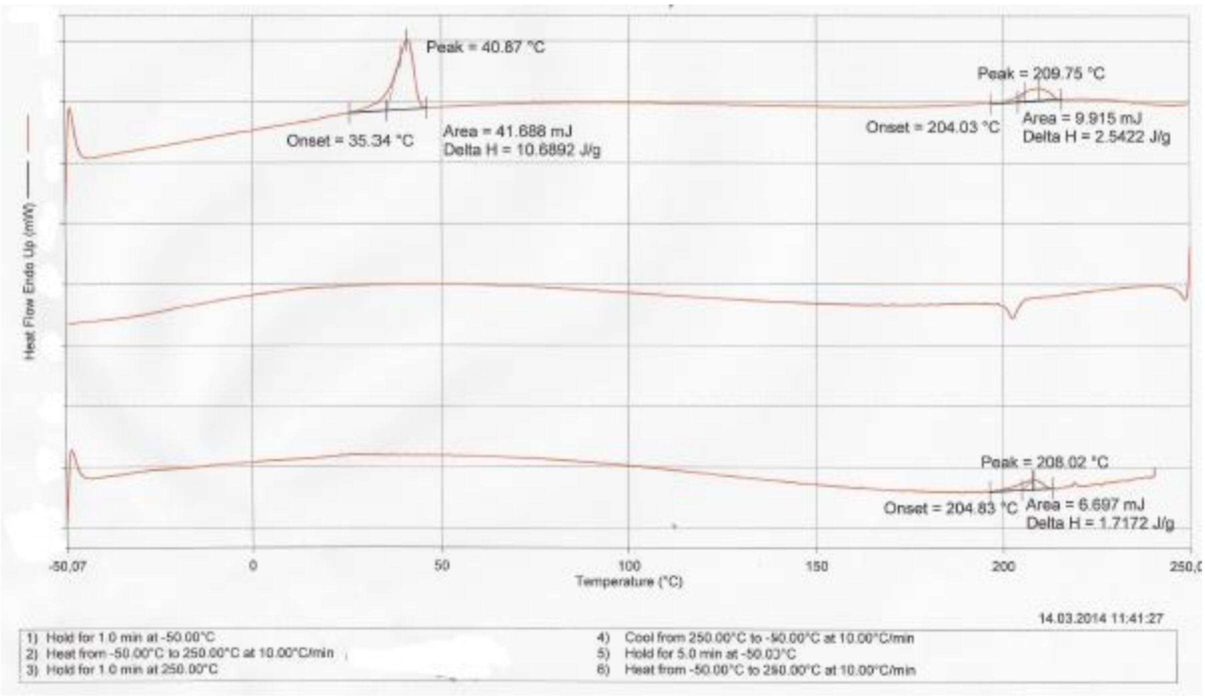


DSC of ***r*-34**

**3,4,5-Tridecyloxy-benzoic amide**[4] 1.98 g (10 mmol) ethyl gallate and 7.74 g (65 mmol) 1-bromodecane in 50 mL dry acetonitrile were treated according to the general procedures GP2-GP4. Hydrolysis of the ester with 3.37 g (60 mmol) KOH in 100mL dioxane/isopropanol 1:1 and chlorination with 3.6 mL (50 mmol) thionylchloride in 100 mL abs. toluene followed by ammonolysis yielded 4.3 g (73%) of a colorless solid. **$^1$H-NMR** (400 MHz, DMSO-$d_6$): $\delta$ [ppm] = 0.83-0.88 (m, 9H, $CH_3$); 1.16-1.32 (m, 12H, $CH_2$); 1.34-1.42 (m, 28 H, $CH_2$); 1.58-1.76 (m, 6H, $CH_2$); 3.93 (t, $^3$J=6.2 Hz, 6H, Ar-O-CH2); 7.04 (s, 2H, C(3)-H, C(3´)-H); 6.26 (s(br), 1H, N-H); 7.21 (s(br), 1H, N-H).

**5-(3,4,5-Tridecyloxy)phenyl-*2H*-tetrazole** 767 mg (1.3 mmol) 3,4,5-Tridecyloxy-benzoic amide, 507 mg (7.8 mmol) $NaN_3$ and 0.30 mL (2.6 mmol) $SiCl_4$, gave, according to GP6 530 mg (66 %) of a colorless solid with m.p. 78 °C. **$^1$H-NMR** (400 MHz, $CDCl_3$): $\delta$ = 0.86-0.98 (m, 9H, CH3); 1.20-1.41 (m, 36H, CH2); 1.38-1.62 (m, 6H, CH2); 1.64-1.95 (m, 6H, CH2); 3.86 (t, $^3$J=6.3 Hz, 4H, Ar-O-CH2); 4.24 (t, $^3$J=6.4 Hz, 4H, Ar-O-CH2); 7.56 (s, 2H, C(3)-H, C(3´)-H). **$^{13}$C-NMR** (75 MHz, $CDCl_3$): $\delta$ = 14.1 (CH3); 22.9, 26.3, 29.6, 29.9, 30.0, 30.8 32.1 (CH2); 69.3; 73.5 (Ar-OCH2); 106.0 (C(3), C(3´)); 119.1 (C(2)); 141.2 (C(5)); 154.2 (C(4), C(4´)); 157.9 ( C(1)). **IR** (KBr): $\tilde{\upsilon}$ [cm$^{-1}$]= 2922; 2807; 2711; 2611; 2488; 1587; 1505; 1467; 1445; 1385; 1308; 1251; 1236; 1148; 1117; 1050; 991; 928; 887; 861; 844; 825. **FD-MS**: m/z (%)= 613.9 (100); 614.9 (42); 615.9 (9) [M]+.; 1229.1 (4) $[2M]^{+\cdot}$. **EA:** $C_{37}H_{66}N_4O_3$ (614.96) calcd.: 72.27%C 10.82 %H 9.11%N, found: 72.36%C 10.58 %H 8.77%N.

**Tri(3,4,5-tridecyloxy)phenyl-tris[1,2,4]triazolo[4,3-a:4´,3´-c:4″,3″-e]-[1,3,5]triazine *t*-35** According to the general procedure GP7, 0.184 g (0.3 mmol) 5-(3,4,5-di(decyloxyphenyl)-2*H*-tetrazole, 17 mg (0.09 mmol) cyanuric chloride, and 0.5 mL pyridine in 30 mL xylenes were stirred at 80 °C. When the reaction was finished (TLC), acidic work-up, extraction with ethyl acetate, evaporation of the solvents and chromatography of the residue through a silica column with toluene/ethyl acetate 40/1 yielded 121 mg (73 %) of a colorless solid with m.p. = 124 °C (DSC). **$^1$H-NMR** (400 MHz, $C_6D_6$): $\delta$= 0.83-0.96 (m, 27H, CH3); 1.17-1.43 (m, 108H, CH2); 1.43-1.52 (m, 12H, CH2); 1.60-1.70 (m, 6H, CH2); 1.73-1.85 (m,12H, CH2); 1.91-2.01 (m, 6H, CH2); 4.18 (t, $^3$J=6.4 Hz, 12H, Ar-O-CH2); 4.31 (t, $^3$J=6.4 Hz, 6H, Ar-O-CH2); 8.15 (s, 6H, C(3)-H, C(3´)-H). **$^{13}$C-NMR** (75 MHz, $C_6D_6$): $\delta$= 14.1 ($CH_3$); 22.9, 26.4, 29.6, 29.8, 30.0,

30.8, 32.1 (CH2); 69.4, 73.5 (Ar-OCH2); 109.4 (C(3), C(3´)); 119.3 (C(2)); 140.5 (C(1a)); 142.1 (C(5)); 150.7 (C(1b)); 153.6 (C(4), C(4´)). **IR** (ATR): $\tilde{\upsilon}$ [cm$^{-1}$]= 2952; 2921; 2852; 1583; 1487; 1466; 1431; 1385; 1340; 1282; 1230; 1114; 978; 872; 835; 719; 702. FD-MS: m/z (%)= 917.6 (4) $[M]^{2+\cdot;}$ 1833.1 (83), 1834.1 (100), 1835.1 (74) $[M]^{+\cdot}$. **EA**: $C_{114}H_{195}N_9O_9$ (1835.88) calcd.: 61.36%C 6.09%H 19.51%N; found: 61.30%C 6.04%H 19.42%N.

**2,6,10-Tris(3,4,5-tri(decyloxy)phenyl)tris([1,2,4]triazolo)[1,5-a:1',5'-c:1'',5''-e][1,3,5]triazine *r*-35** 250 mg 3,7,11-tris(3,4,5-tri(decyloxy)phenyl)tris([1,2,4]triazolo)[4,3-a:4',3'-c:4'',3''-e][1,3,5]triazine in 2.5 mL octadecane was stirred for 60 h at 250 °C. The cooled mixture was diluted with light petroleum, filtered through silica and after octadecane had been eluted with petroleum ether, the product was isolated with toluene. Chromatography on silica with petroleum ether/ethyl acetate 30/1 gave 54 mg (22 %) of a colorless solid. **$^1$H-NMR** (400 MHz, $CDCl_3$): $\delta$ [ppm] = 7.59 (s, 2H, 2-H, 6-H, ph), 4.11 (t, $^3J$ = 6.5 Hz, 4H, $OCH_2$), 4.01 (t, $^3J$ = 6.5 Hz, 2H, $OCH_2$), 1.89 – 1.70 (m, 6H, $CH_2$), 1.56 – 1.21 (m, 42H, $CH_2$), 0.89 (t, 9H, $CH_3$). **$^{13}$C-NMR** (100 MHz, $CDCl_3$): $\delta$ [ppm] = 165.14 (C-5, trl), 153.56 (C-3, C-5, ph), 144.53 (C-3, trl), 141.10 (C-4, ph), 123.00 (C-1, ph), 106.24 (C-2, ph), 73.72, 69.51 ($OCH_2$), 32.12, 32.10, 30.57, 29.95, 29.89, 29.86, 29.81, 29.64, 29.59, 29.56, 26.31, 22.86 ($CH_2$), 14.27 ($CH_3$). **IR** (ATR): $\tilde{\upsilon}$ [cm$^{-1}$] = 2920 s, 2851 m, 1629 s, 1586 w, 1465 s, 1435 s, 1381 m, 1332 s, 1227 m, 1114 s, 982 w, 847 w, 746 m, 721 w. **HR-ESI:** calcd. for $C_{114}H_{195}N_9O_9$ + $H^+$: 1835.5156; found: 1835.5225.

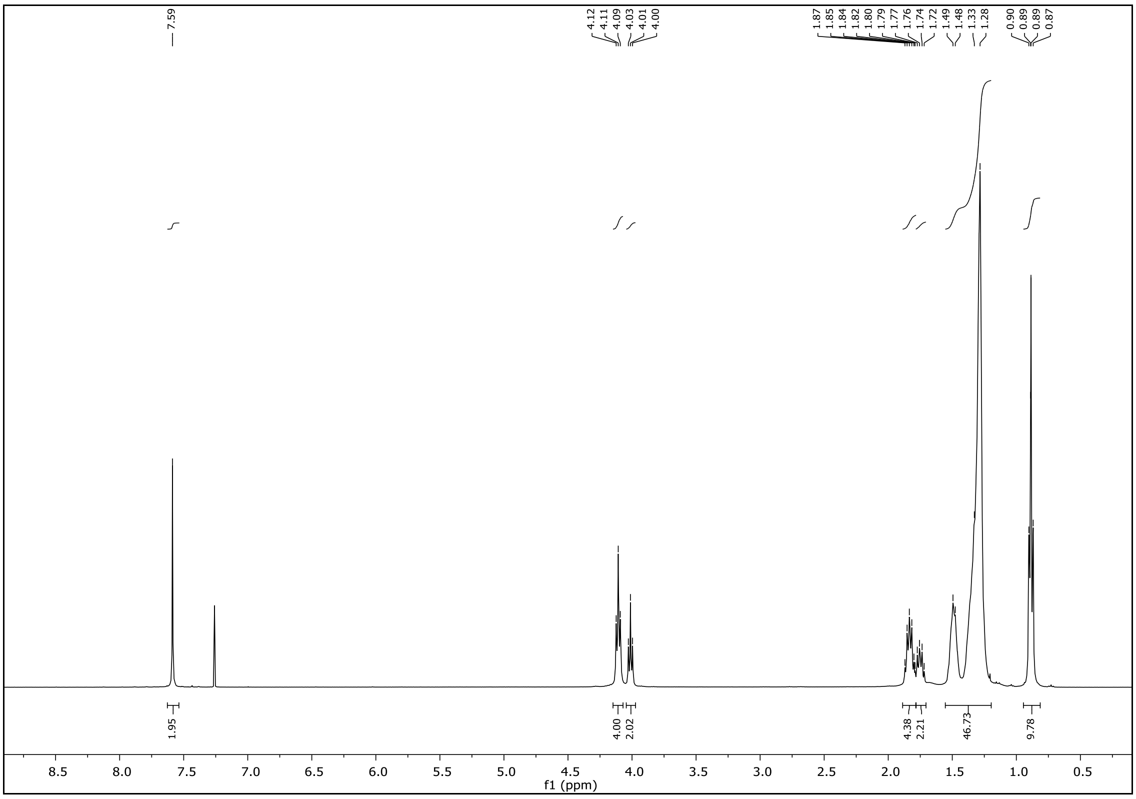

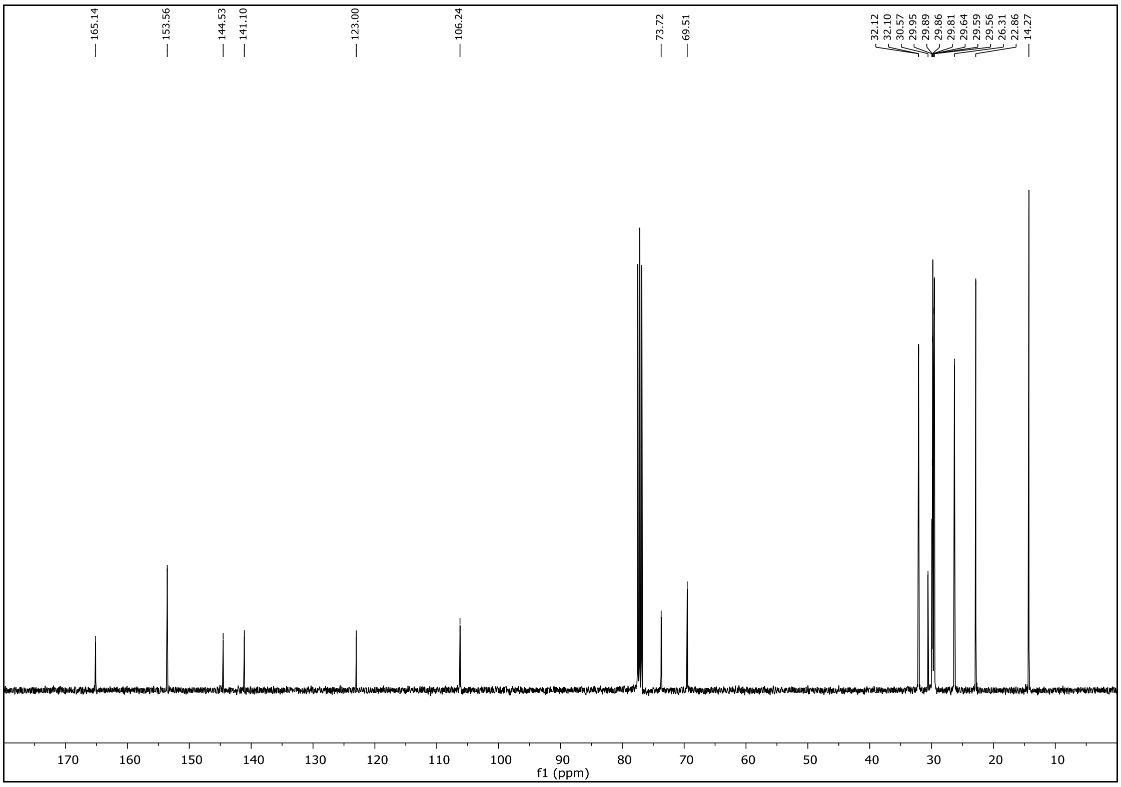


**3,4,5-Tridodecyloxy-benzoic amide[4]**
According to GP2-GP4, 2.18 g (11 mmol) ethyl gallate and 8.72 g (35 mmol) 1-bromododecane in 50 mL dry acetonitrile were treated according to the general procedure. Hydrolysis of the ester with 1.69 g (30 mmol) KOH in 100mL dioxane/isopropanol 1:1 and chlorination with 4.0 mL (55 mmol) thionylchloride in 100 mL abs. toluene followed by ammonolysis yielded 4.9 g (66%) of a colorless solid. **$^1$H-NMR** (400 MHz, $CDCl_3$): $\delta$ [ppm] = 0.83-0.87 (m, 9H, $CH_3$); 1.14-1.32 (m, 32H, $CH_2$); 1.32-1.47 (m, 6H, $CH_2$); 1.65-1.78 (m, 6H, CH2); 3.92 ( t, $^3J$=6.6 Hz, 4H, Ar-O-$CH_2$); 4.01 (t, $^3J$=6.6 Hz, 2H, Ar-O-$CH_2$); 5.89 (s,(br)), 2H, $ArCONH_2$); 7.24 (s(br), 2H, C(3)-H, C(3´)-H). **IR** (Film): $\tilde{\upsilon}$ [$cm^{-1}$]= 2921; 2851; 2712; 2617; 2484; 1589; 1503; 1468; 1444; 1385; 1306.

**5-(3,4,5-Tridodecyloxy)phenyl-*2H*-tetrazole**
According to the general procedure GP6, 742 mg (1.1 mmol) 3,4,5-tridodecyloxy-benzoic amide, 585 mg (9 mmol) $NaN_3$ and 0.34 mL (3 mmol) $SiCl_4$ gave 324 mg (36 %) of a colorless solid with m.p. = 94 °C. **$^1$H-NMR** (400 MHz, $CDCl_3$): $\delta$ [ppm] = 0.83-0.87 (m, 9H, $CH_3$); 1.14-1.32 (m, 54H, $CH_2$); 1.32-1.47 (m, 6H, $CH_2$); 1.65-1.78 (m, 6H, $CH_2$); 3.92 ( t, $^3J$=6.6 Hz, 4H, Ar-O-$CH_2$); 4.01 ( t, $^3J$=6.6 Hz, 2H, Ar-O-$CH_2$); 7.24 (s(br), 2H, C(3)-H, C(3´)-H). **$^{13}$C-NMR** (75 MHz, $CDCl_3$): $\delta$= 14.1 ($CH_3$); 22.7, 26.1, 29.2, 29.4, 30.2, 31.9 ($CH_2$); 69.3, 73.9 (Ar-O-$CH_2$); 105.5 (C(3), C(3´)); 118.6 (C(2)); 140.3 (C(5)); 153.7 (C(4), C(4´)); 156.9 (br, C(1)). **IR** (ATR): $\tilde{\upsilon}$ [$cm^{-1}$]= 2921; 2851; 2712; 2617; 2484; 1589; 1503; 1468; 1444; 1385; 1306; 1249; 1054; 991; 949; 847; 824; 758; 721. **FD-MS**: m/z (%)= 668.8 (18), 669.8 (9) $[M-N_2]^+$; 696.8 (100), 697.8 (65), 698.8 (16) $[M]^+$; 1395.0 (25),1396.0 (20) $[2M]^{+\cdot}$.

**Tri(3,4,5-tri(dodecyloxy)phenyl)-tris[1,2,4]triazolo[4,3-a:4´,3´-c:4″,3″-e]-[1,3,5]triazine *t*-36**
According to GP7, 189 mg (0.27 mmol) (3,4,5-tri(dodecyloxyphenyl)-*2H*-tetrazole and 15 mg (0.08 mmol) cyanuric chloride in 50 mL xylenes with 0.5 mL pyridine gave, after column chromatography ($SiO_2$; toluene/ethyl acetate 40/1, $R_f$=0.33) 142 mg (85 %) of a colorless solid with m.p. = 125 °C. **$^1$H-NMR** (400 MHz, $CDCl_3$): $\delta$= 0.83-0.87 (m, 27H, $CH_3$); 1.14-1.34 (m, 162 H, $CH_2$); 1.38-1.49 (m, 18H, $CH_2$); 1.72-1.85 (m, 18H, $CH_2$); 4.04 (t, $^3J$=6.4 Hz, 18H,

Ar-O-$CH_2$); 7.41 (s, 6H, C(3)-H, C(3´)-H). **$^{13}$C-NMR** (75 MHz, $CDCl_3$): δ= 14.1 ($CH_3$); 22.7, 26.1, 29.4, 29.5, 29.6, 29.8, 31.9 ($CH_2$); 69.3, 73.6 (Ar-$OCH_2$); 108.7 (C(3), C(3´)); 118.2 (C(2)); 140.6 (C(5)); 141.3 (C(1a)); 151.2 (C(1b)); 153.0 (C(4), C(4´)). **IR** ): $\tilde{\upsilon}$ [$cm^{-1}$]= 2924; 2853; 1588; 1489; 1468; 1387; 1341; 1285; 1248; 1232; 1118; 1005; 870; 799; 721. **FD-MS:** m/z (%)= 2085.7 (100), 2086.1 (63) $[M]^{+\cdot}$. **EA:** $C_{132}H_{231}N_9O_9$ (2088.37) calcd.: 75.92%C 11,15% H 6,04%N found: 75.61%C 11.13% H 5.87%N.

**5-(3,4,5-Tri(hexadecyloxy)phenyl)-2*H*-tetrazole**
According to the procedure GP6, 2.50 g (3mmol) 5-(3,4,5-tri(hexadecyloxyphenyl)-benzoic amide, 700 mg (11 mmol) $NaN_3$, and 1.46 g (11 mmol) $SiCl_4$ in 50 mL toluene were stirred for 12 h under reflux. Additional reagents (200 mg (3 mmol) $NaN_3$, 0.41 g (3 mmol) $Et_3NHCl$) were added, stirring under reflux for 10 h. Filtration, aqueous work-up and recrystallization from toluene yielded 159 mg (60 %) of a colorless solid with m.p. = 90 – 92 °C. **$^1$H-NMR** (400 MHz, $CDCl_3$): *δ* [ppm] = 7.27 (s ‚breit', 2H, 2-H, 6-H, ph), 4.03 (t, $^3J$ = 6.5 Hz, 2H, $OCH_2$), 3.95 (t, $^3J$ = 6.5 Hz, 4H, $OCH_2$), 1.80 – 1.71 (m, 6H, $CH_2$), 1.48 – 1.24 (m, 78H), 0.87 (t, 9H, $CH_3$). **$^{13}$C-NMR** (100 MHz, $CDCl_3$): *δ* [ppm] = 153,07 (C-3, C-5, ph), 139,76 (C-4, ph), 118.57 (C-1, ph), 105.09 (C-2, C-6, ph) 73,00, 68.68 ($OCH_2$), 31.39, 29.79, 29.22, 29.18, 29.13, 29.06, 28.89, 28.84, 28.80, 25.59, 25.56, 22.16 ($CH_2$), 13.61 ($CH_3$). **IR** (ATR): $\tilde{\upsilon}$ [$cm^{-1}$] = 2915 s, 2848 s, 1585 w, 1505 w, 1468 m, 1447 m, 1378 w, 1310 w, 1247 w, 1120 s, 1052 w, 983 w, 843 w, 719 w. **HR-ESI**: calcd. for $C_{55}H_{102}N_4O_3 + H^+$: 867.8030; found: 657.8038.

**3,7,11-Tris(3,4,5-tri(hexadecyloxy)phenyl)tris([1,2,4]triazolo)[4,3-a:4',3'-c:4",3"-e]-[1,3,5]triazine *t*-37**

According to the general procedure GP7, 500 mg (0.58 mmol) (3,4,5-tri(hexadecyloxyphenyl)-*2H*-tetrazole and collidine (0.1 mL) were stirred in xylenes (30 mL) for 15 min. A solution of 30 mg cyanuric chloride in 3 mL xylenes was added and every hour 1 mg cyanuric chloride was added until all tetrazole had been consumed (TLC). Aqueous work-up and chromatography on a column of silica with a head of basic alumina (gradient toluene to toluene / ethyl acetate 40/1) yielded 55 mg (12 %) of a colorless solid with m.p. = 124 °C (DSC, 2$^{nd}$ heating). $^{1}$H-NMR (400 MHz, $CDCl_3$): $\delta$ [ppm] = 7.43 (s, 2H, 2-H, 6-H, ph), 4.07 (t, 6H, $OCH_2$), 1.86 – 1.75 (m, 6H, $CH_2$), 1.52 – 1.25 (m, 78H), 0.89 – 0.86 (m, 9H, $CH_3$). $^{13}$C-NMR (100 MHz, $CDCl_3$): $\delta$ [ppm] = 153.16 (C-3, C-5, ph), 151.37 (C-5, trl), 141.45, 140.72 (C-3, trl; C-4, ph), 118.32 (C-1), 108.88 (C-2, C-6), 73.80, 69.47 ($OCH_2$), 32.09, 30.54, 29.93, 29.89, 29.83, 29.79, 29.63, 29.53, 29.50, 26.31, 26.27, 22.85 ($CH_2$), 14.28 ($CH_3$). IR (ATR): $\tilde{\nu}$ [cm$^{-1}$] = 2916 s, 2848 s, 1576 m, 1489 m, 1468 m, 1430 m, 1389 w, 1337 m, 1222 w, 1121 m, 968 w, 795 m, 720 m. FD-MS: m/z (%): 1296.6 (15) [$M^{2+}$]; 2592.3 (100) [$M^{+}$].

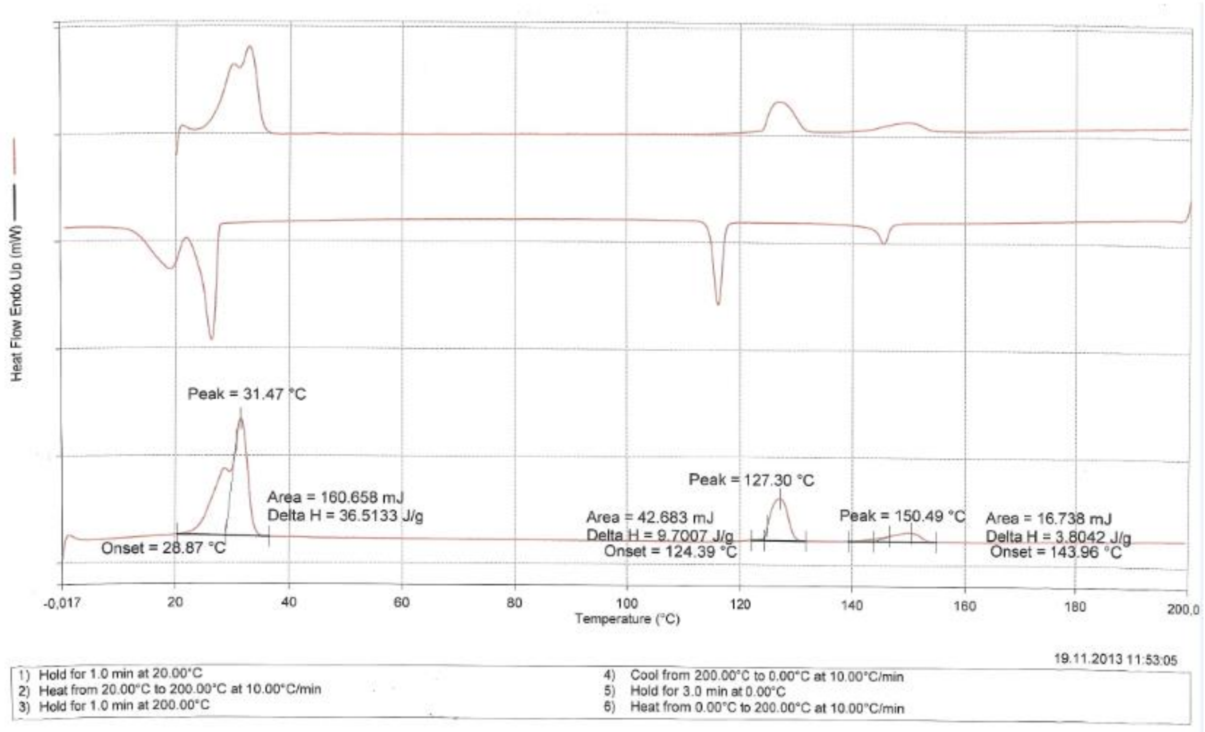


DSC of ***t*-37**

**6-Decyloxy-2-naphthonitrile**

3.18 g 2-Bromo-6-(decyloxy)naphthalene (8.8 mmol) and 1.60 g CuCN (18.0 mmol) in 50 mL NMP were heated to 200 °C for 6 h. Aqueous work-up, extraction with ether (Kutscher-Steudel) and recrystallization from light petroleum yielded 2.0 g (73 %) of a colorless solid with m.p. 78 - 80 °C. **$^{1}$H-NMR** (400 MHz, $CDCl_3$): $\delta$ [ppm] = 8.12 (s, 1H, 1-H, naph), 7.77 (m, 2H, 4-H, 8-H, naph), 7.55 (dd, $^{3}J$ = 8.5, $^{4}J$ = 1.3 Hz, 1H, 3-H, naph), 7.24 (dd, $^{3}J$ = 9.0, $^{3}J$ = 2.4 Hz, 1H, 7-H, naph), 7.13 (d, $^{3}J$ = 2.4 Hz, 1H, 5-H, naph), 4.09 (t, 2H, $OCH_2$), 1.92 – 1.74 (m, 2H, $CH_2$), 1.55 – 1.16 (m, 14H, $CH_2$), 0.88 (t, 3H, $CH_3$). **$^{13}$C-NMR** (100 MHz, $CDCl_3$): $\delta$ [ppm] = 159.71 (C-6), 136.61 (C-4a), 133.88, 130.03, 127.86 (CH, naph), 127.75 (C-8a), 127.12, 121.09 (CH, naph), 119.79 (CN), 106.67 (CH, naph, C-2), 68.44 ($OCH_2$), 32.03, 29.70, 29.51, 29.45, 29.22, 26.19, 22.81 ($CH_2$), 14.25 ($CH_3$). **IR** (ATR): $\tilde{\nu}$ [cm$^{-1}$] = 2922 m, 2850 w, 2220 w, 1623 m, 1469 m, 1391 m, 1265 m, 1225 m, 1175 s, 1120 w, 1015 m, 919 m, 844 s, 827 m, 805 m, 724 m. **HR-ESI**: calcd. for $C_{21}H_{27}NO + Na^{+}$: 332.1990; found: 332.1998.

**5-(6-(Decyloxy)naphth-2-yl)-2*H*-tetrazole**

According to the general procedure GP5, 1.90 g (6.1 mmol) 6-decyloxy-2-naphthonitrile, 1.20 g (18.00 mmol) $NaN_3$, 2.54 g (18 mmol) $NEt_3HCl$ in 50 mL toluene were refluxed for 12 h. Aqueous work-up and recrystallization from petroleum ether yielded 1.32 g (3.7 mmol, 62 %) of a colorless solid with m.p. = 155 - 157 °C. **$^1$H-NMR** (400 MHz, $CDCl_3$): $\delta$ [ppm] = 8.49 (s, 1H, 1-H, naph), 8.04 (d, $^3J$ = 8.6 Hz, 1H, 3-H, naph), 7.77 (d, 2H, 4-H, 8-H, naph), 7.15 (dd, $^3J$ = 8.9, $^4J$ = 2.2 Hz, 1H, 7-H, naph), 7.09 (d, $^4J$ = 2.2 Hz, 1H, 5-H, naph), 4.04 (t, 2H, $OCH_2$), 1.86 – 1.74 (m, 2H, $CH_2$), 1.50 – 1.13 (m, 14H, $CH_2$), 0.82 (t, 3H, $CH_3$). **$^{13}$C-NMR** (100 MHz, $CDCl_3$): $\delta$ [ppm] = 158.56 (C-6), 135.93 (C-4a), 130.17, 128.36 (C-8a), 127.71, 127.28, 124.38, 120.19, 106.51 (CH, naph), 68.17 ($OCH_2$), 31.87, 29.55, 29.38, 29.30, 29.16, 26.07, 22.66 ($CH_2$), 14.12 ($CH_3$). **IR** (ATR): $\tilde{\upsilon}$ [$cm^{-1}$] = 2920 m , 1629 m, 1564 m, 1507 w, 1469 m, 1392 m, 1264 m, 1216 s, 1173 m, 1052 w, 1021 m, 921 w, 847 m, 788 s. **HR-ESI**: calcd. for $C_{21}H_{28}N_4O + H^+$: 353.2341; found: 353.2346.

**3,7,11-Tris(6-(decyloxy)naphth-2-yl)tris([1,2,4]triazolo)[4,3-a:4',3'-c:4'',3''-e] [1,3,5]triazine *t*-38**

According to the general procedure GP7, 600 mg (1.70 mmol) 6-decyloxynaphth-2-yl-*2H*-tetrazole, 95 mg (0.5 mmol) cyanuric chloride and collidine (0.25 mL) were stirred in xylenes (30 mL) and the temperature was gradually raised to 60 °C. After 12 h, aqueous work-up and chromatography on a column of basic alumina with a gradient from toluene to toluene / ethyl acetate 30 / 1 yielded 245 mg (45 %) of a colorless solid with m.p. = 120 - 121 °C. **$^1$H-NMR** (400 MHz, $CDCl_3$): $\delta$ [ppm] = 8.71 (d, $^4J$ = 1 Hz, 1H, 1-H, Naph), 8.11 (dd, $^3J$ = 8.6, $^4J$ = 1 Hz, 1H, 3-H, Naph), 7.84 (m, 2H, 4-H, 8-H, naph), 7.18 (dd, $^3J$ = 9.0, $^4J$ = 2.1 Hz, H, 7-H, naph), 7.12 (d, $^3J$ = 2.1 Hz, 1H, 5-H, Naph), 4.05 (t, 2H, $OCH_2$), 1.90 – 1.75 (m, 2H, $CH_2$), 1.56 – 1.21 (m, 14H, $CH_2$), 0.90 (t, 3H, $CH_3$). **$^{13}$C-NMR** (100 MHz, $CDCl_3$): $\delta$ [ppm] = 159.11 (C-6, naph), 151.28 (C-5, trl), 140.71 (C-3, trl), 136.37 (C-4a, naph), 130.87, 130.79 (CH, naph), 127.92 (C-8a, naph), 127.03, 126.63, 120.20 (CH, naph), 118.78 (C-2, naph), 106.52 (CH, naph), 68.33 ($OCH_2$), 32.07, 29.75, 29.61, 29.50, 29.34, 26.24, 22.84 ($CH_2$), 14.28 ($CH_3$). **IR** (ATR): $\tilde{\upsilon}$ [$cm^{-1}$] = 2921 m, 2852 m, 1627 m, 1590 s, 1495 m, 1466 w, 1389 m, 1261 m, 1213 s, 1198 m, 1126 m, 916 w, 850 m, 754 s, 712 m. **HR-ESI**: calcd. for $C_{66}H_{81}N_9O_3 + H^+$: 1048.6541; found: 1048.6500.

**3,7,11-Tris(5,6-di(octyloxy)naphth-2-yl)tris([1,2,4]triazolo)[4,3-a:4',3'-c:4'',3''-e] [1,3,5]triazine *t*-39**

According to the general procedure GP7, 435 mg (0.96 mmol) (5,6-dioctyloxynaphth-2-yl)-*2H*-tetrazole and collidine (0.2 mL) were stirred in xylenes (30 mL) for 15 min. 54 mg (0.3 mmol) Cyanuric chloride 50 mg (0.29 mmol) was added and the stirred mixture gradually heated to 80 °C. After 12 h, aqueous work-up and chromatography on a column of basic alumina with toluene followed by toluene / ethyl acetate 30/1 yielded 218 mg (72 %) of a colorless solid with m.p. = 128 °C (DSC, 1. heating). **$^1$H-NMR** (400 MHz, $CDCl_3$): $\delta$ [ppm] = 8.77 (d, $^4J$ = 1.5 Hz, 1H, 1-H, naph), 8.31 (d, $^3J$ = 8.9 Hz, 1H, 4-H, naph), 8.16 (dd, $^3J$ = 8.9, $^4J$ = 1.5 Hz, 1H, 3-H, naph), 7.75 (d, $^3J$ = 9.0 Hz, 1H, 8-H, naph), 7.35 (d, $^3J$ = 9.0 Hz, 1H, 7-H, naph), 4.17 – 4.13 (m, 4H, $OCH_2$), 1.96 – 1.81 (m, 4H, $CH_2$), 1.57 – 1.26 (m, 20H, $CH_2$), 0.90 (t, 6H, $CH_3$). **$^{13}$C-NMR** (100 MHz, $CDCl_3$): $\delta$ [ppm] = 151.45 (C-5, trl), 149.95, 142.46 (C-5, C-6, naph), 140.87 (C-3, trl), 131.11, 130.99, 128.54, 126.11, 125.50, 122.25, 119.28, 117.14 (C, CH, naph), 74.05, 69.81 ($OCH_2$), 32.03, 31.99, 30.64, 29.78, 29.70, 29.55, 29.50, 29.45, 26.35, 26.28, 22.83 ($CH_2$), 14.09 ($CH_3$). **IR** (ATR): $\tilde{\upsilon}$ [$cm^{-1}$] = 3071 w, 2923 s, 2853 s, 1624 m, 1588 s, 1509 m, 1465 m, 1425 m 1351 s, 1258 s, 1186 m, 1102 s, 1047 s, 895 w, 827 m, 713 s. **FD-MS:** m/z (%): 674.0 (14) [$M^{2+}$]; 1348.2 (100) [$M^+$]. **HR-ESI:** calcd. for $C_{84}H_{117}N_9O_6 + H^+$: 1348.9205; found: 1348.9193.

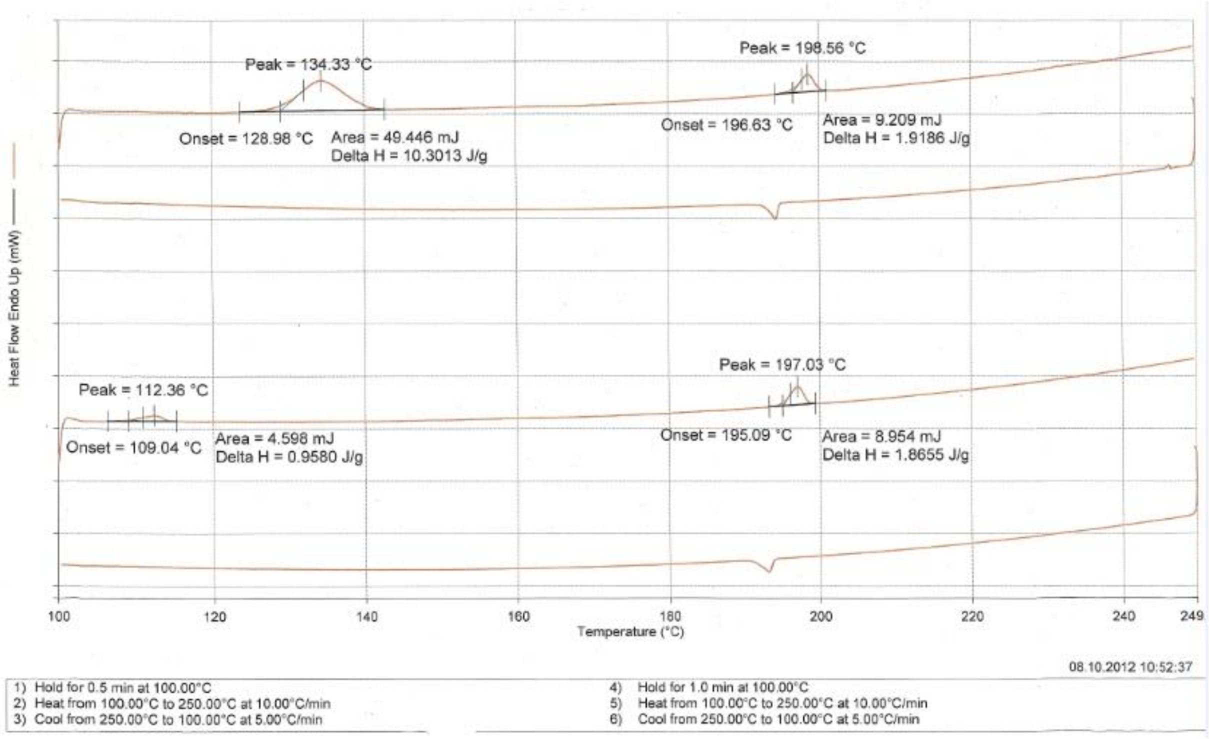


DSC of ***t*-39**

**6-Bromo-1,2-di(decyloxy)naphthalene**
6-Bromo-1,2-dihydroxynaphthalene (2.43 g, 10 mmol), potassium carbonate (4.21 g, 30 mmol), and 1-bromdecane (4.95 g, 22 mmol) in 50 mL DMF were stirred at ambient temperature for 60 h. Aqueous work-up, extraction with ethyl acetate and recrystallization from light petroleum yielded 4.08 g (7.8 mmol, 76 %) of a colorless solid with m.p. = 32 - 34 °C. **$^1$H-NMR** (400 MHz, $CDCl_3$): $\delta$ [ppm] = 8.00 (d, $^3J$ = 9.0 Hz, 1H, 8-H, naph), 7.90 (d, $^4J$ = 1.8 Hz, 1H, 5-H, naph), 7.49 (dd, $^3J$ = 9.0 Hz, $^4J$ = 1,8 Hz, 1H, 7-H, naph), 7.45 (d, $^3J$ = 9.0, 1H, 4-H, naph), 7.27 (d, $^3J$ = 9.0 Hz, 1H, 3-H, naph), 4.13 – 4.09 (m, 4H, $OCH_2$), 1.93 – 1.74 (m, 4H, $CH_2$), 1.62 – 1.16 (m, 28H, $CH_2$), 0.88 (t, 6H, $CH_3$). **$^{13}$C-NMR** (100 MHz, $CDCl_3$): $\delta$ [ppm] = 148.19, 142.84 (C-1, C-2), 130.81 (C-naph), 129.56, 129.25 (CH-naph), 128.22 (C-naph), 123.76, 122.92 (CH-naph), 117.98 (C-6), 117.88 (CH-naph), 73.98, 70.08 ($OCH_2$), 32.07, 30.59, 29.82, 29.78, 29.74, 29.70, 29.58, 29.50, 26.33, 26.28, 22.84 ($CH_2$), 14.28 ($CH_3$). **IR** (ATR): $\tilde{\nu}$ [cm$^{-1}$] = 2916 s, 2849 s, 1585 m, 1497 w, 1463 m, 1351 m, 1273 s, 1202 w, 1102 m, 1072 s, 953 w, 875 m, 825s, 796 m. **FD-MS:** m/z (%) = 518.6 $[M]^+$; 1038.7 $[M_2]^+$. **HR-ESI:** calcd. for $C_{30}H_{47}BrO_2 + Na^+$: 541.2657; found: 541.2647.

**5,6-Di(decyloxy)-2-naphthonitrile**
A mixture of 1.9 g (3.6 mmol) 6-bromo-1,2-di(decyloxy)naphthalene, 1.4 g (16.0 mmol) CuCN in 60 mL DMF was stirred at 150 °C until bromonaphthalene had been consumed (TLC). Aqueous work-up, extraction with light petroleum gave 1g (57%) of a colorless solid with m.p. = 48 - 49 °C. **$^1$H-NMR** (400 MHz, $CDCl_3$): $\delta$ [ppm] = 8.19 (d, $^3J$ = 8.8 Hz, 1H, 4-H, naph), 8.14 (d, $^4J$ = 1.6 Hz, 1H, 1-H, naph), 7.61 (d, $^3J$ = 9.0 Hz, 1H, 8-H, naph), 7.54 (dd, $^3J$ = 8.8, $^4J$ = 1.6 Hz, 1H, 3-H, naph), 7.37 (d, $^3J$ = 9.0 Hz, 1H, 7-H, naph), 4.15 (t, 2H, $OCH_2$), 4.12 (t, 2H, $OCH_2$), 1.90 – 1.83 (m, 4H, $CH_2$), 1.55 – 1.48 (m, 4H, $CH_2$), 1.40 – 1.25 (m, 24H, $CH_2$), 0.90 – 0.87 (m, 6H, $CH_3$). **$^{13}$C-NMR** (100 MHz, $CDCl_3$): $\delta$ [ppm] = 150.53, 142.29 (C-5, C-6), 134.14, (CH-naph), 130.98, 128.09 (C-4a, C-8a), 126.30, 124.64, 123.09 (CH-naph), 119.81 (C-2), 117.59 (CH-naph), 107.18 (CN), 74.03, 69.74 ($OCH_2$), 32.06, 30.56, 29.81, 29.77, 29.72, 29.68, 29.54, 29.50, 26.31, 26.25, 22.83 ($CH_2$), 14.26 ($CH_3$). **IR** (ATR): $\tilde{\nu}$ [cm$^{-1}$] = 2924 s, 2849 s, 2223 m, 1690 w, 1660 w, 1627 m, 1604 m, 1465 s, 1357 s, 1271 s, 1191 w, 1100

m, 1049 s, 895 w, 826 m, 798 w, 718 w. **HR-ESI**: calcd. for $C_{31}H_{47}NO_2$ + $Na^+$: 488.3504; found: 488.3521.

**5-(5,6-Di(decyloxy)naphth-2-yl)-2*H*-tetrazole**

According to the general procedure, 1.00 g (2.14 mmol) 5,6-didecyloxy-2-naphthonitrile, 0.78 g (12.00 mmol) $NaN_3$, 1.66 g (12 mmol) $NEt_3HCl$ in 30 mL toluene were refluxed until the nitrile had been consumed (TLC). Aqueous work-up and recrystallization from ethanol yielded 0.89 g (1.8 mmol, 82 %) of a colorless solid with m.p. = 133 - 134 °C (ethanol). **$^1$H-NMR** (400 MHz, $CDCl_3$/ DMSO-$d_6$): $\delta$ [ppm] = 8.48 (s, 1H, 1-H, naph), 8.15 (d, $^3J$ = 8.8 Hz, 1H, 4-H, naph), 8.00 (d, $^3J$ = 8.8 Hz, 1H, 3-H, naph), 7.58 (d, $^3J$ = 9.0 Hz, 1H, 8-H, naph), 7.23 (d, $^3J$ = 9.0 Hz, 1H, 7-H, naph), 4.05 (t, 4H, $OCH_2$), 1.83 – 1.87 (m, 4H, $CH_2$), 1.47 – 1.17 (m, 28H, $CH_2$), 0.80 – 0.77 (m, 6H, $CH_3$). **$^{13}$C-NMR** (75 MHz, $CDCl_3$/DMSO-$d_6$): $\delta$ [ppm] = 156.83 (C-5, tet), 149.00, 142.09 (C-5, C-6, naph), 130.20, 128.83 (C-4a, C-8a, naph), 127.08, 124.62, 123.68, 122.51 (CH-naph), 120.08 (C-2, naph), 116.85 (CH-naph), 73.65, 69.49 ($OCH_2$), 31.78, 30.32, 29.56, 29.51, 29.46, 29.33, 29.22, 26.05, 26.02, 22.55 ($CH_2$), 13.97 ($CH_3$). **IR** (ATR): $\tilde{\nu}$ [$cm^{-1}$] = 3390 s, br, 2924 m, 2849 m, 1664 m, 1563 w, 1512 w, 1467 w, 1336 w, 1271 w, 1026 s, 1003 s, 826 m, 769 m. **HR-ESI**: calcd. for $C_{31}H_{48}N_4O_2$ + $H^+$: 509.3856; found: 509.3848.

**3,7,11-Tris(5,6-di(decyloxy)naphth-2-yl)tris([1,2,4]triazolo)[4,3-a:4',3'-c:4'',3''-e][1,3,5]-triazine *t*-40**

According to GP7, 452 mg (0.89 mmol) (5,6-didecyloxynaphth-2-yl)-*2H*-tetrazole and collidine (0.2 mL) were stirred in xylenes (30 mL) for 15 min. 50 mg (0.3 mmol) cyanuric chloride (0.26 mmol) was added and the mixture was stirred for 24 h. Aqueous work-up and chromatography on a column of basic alumina with a gradient from light petroleum to toluene yielded 240 mg (53 %) of a colorless solid with m.p. = 83 °C (DSC, 1. heating). **$^1$H-NMR** (400 MHz, $CDCl_3$): $\delta$ [ppm] = 8.76 (d, $^4J$ = 1.7 Hz, 1H, 1-H, naph), 8.31 (d, $^3J$ = 8.9 Hz, 1H, 4-H, naph), 8.16 (dd, $^3J$ = 8.9, $^4J$ = 1.7 Hz, 1H, 3-H, naph), 7.75 (d, $^3J$ = 9.0 Hz, 1H, 8-H, naph), 7.36 (d, $^3J$ = 9.0 Hz, 1H, 7-H, naph), 4.16 (t, 4H, $OCH_2$), 1.94 – 1.84 (m, 4H, $CH_2$), 1.61 – 1.23 (m, 28H, $CH_2$), 0.91 – 0.87 (m, 6H, $CH_3$). **$^{13}$C-NMR** (100 MHz, $CDCl_3$): $\delta$ [ppm] = 151.46 (C-5, trl), 149.96, 142.49 (C-5, C-6, naph), 140.88 (C-3, trl), 131.11, 131.01, 128.56, 126.10, 125.49, 122.27, 119.28, 117.17 (C, CH, naph), 74.07, 69.83 ($OCH_2$), 32.08, 30.66, 29.87, 29.81, 29.77, 29.61, 29.53, 26.38, 26.30, 22.85 ($CH_2$), 14.28 ($CH_3$). **IR** (ATR): $\tilde{\nu}$ [$cm^{-1}$] = 2918 s, 2855 s, 1624 m, 1590 s, 1513 m, 1467 m, 1419 w, 1353 m, 1277 s, 1254 s, 1186 w, 1106 m, 1055 m, 894, 832 w, 793 w, 758 w, 707 w, 685 w. **FD-MS**: m/z (%): 758.8 (11) [$M^{2+}$]; 1517.1 (100) [$M^+$]. **HR-ESI**: calcd. for $C_{96}H_{141}N_9O_6$ + $H^+$: 1517.1083; found: 1517.1113.

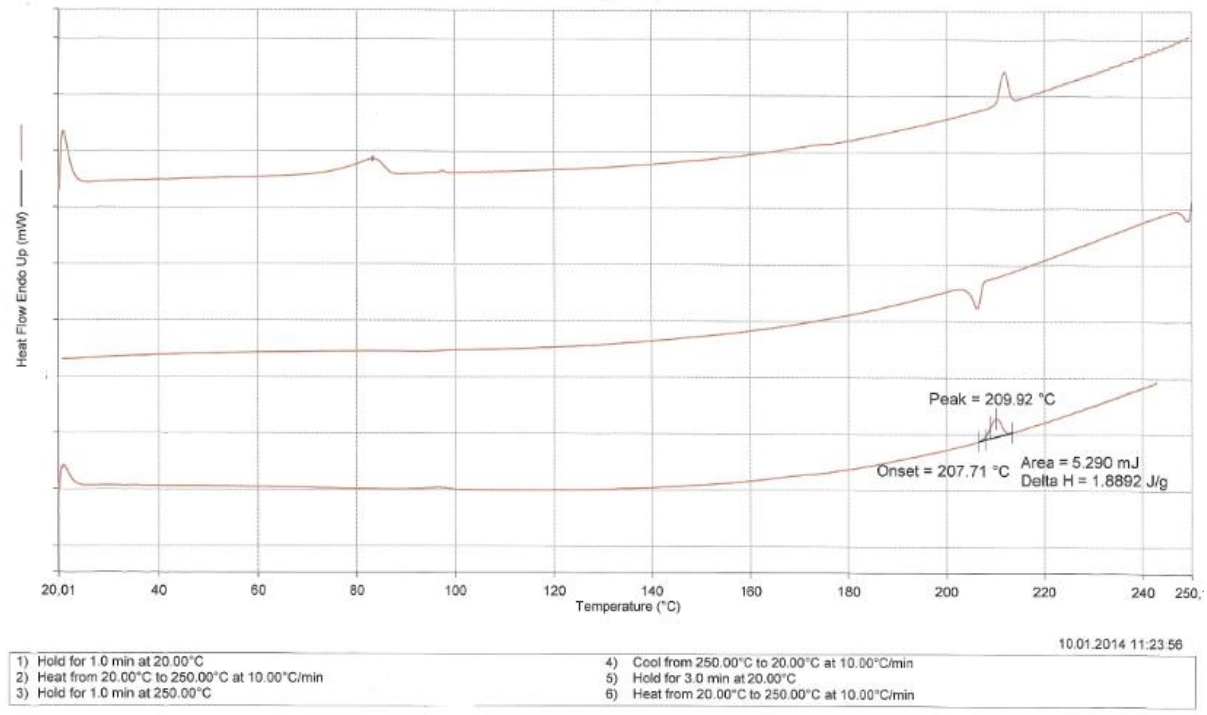


DSC of ***t*-40**

**6-Bromo-1,2-di(dodecyloxy)naphthalene**
6-Bromo-1,2-dihydroxynaphthalene (2.43 g, 10 mmol), potassium carbonate (4.21 g, 30 mmol), and 1-bromdodecane (5.57 g, 22 mmol) in 50 mL DMF were stirred at ambient temperature for 60 h. Aqueous work-up, extraction with light petroleum and recrystallization from light petroleum yielded 5.32 g (9.2 mmol, 92 %) of a colorless solid with m.p. = 44 - 46 °C. **$^{1}$H-NMR** (400 MHz, $CDCl_3$): $\delta$ [ppm] = 8.00 (d, $^{3}J$ = 9.0 Hz, 1H, 8-H, naph), 7.90 (d, $^{4}J$ = 1.9 Hz, 1H, 5-H, naph), 7.49 (dd, $^{3}J$ = 9.0 Hz, $^{4}J$ = 2.0 Hz 1H, 7-H, naph), 7.45 (d, $^{3}J$ = 9.0, 1H, 4-H, naph), 7.27 (d, $^{3}J$ = 9.0 Hz, 1H, 3-H, naph), 4.13 – 4.09 (m, 4H, $OCH_2$), 1.94 – 1.75 (m, 4H, $CH_2$), 1.56 – 1.47 (m, 4H, $CH_2$), 1.42 – 1.22 (m, 32H, $CH_2$), 0.88 (t, 6H, $CH_3$). **$^{13}$C-NMR** (100 MHz, $CDCl_3$): $\delta$ [ppm] = 148.18, 142.82 (C-1, C-2), 130.79 (C-naph), 129.56, 129.24 (CH-naph), 128.20 (C-naph), 123.75, 122.91 (CH-naph), 117.97 (C-6), 117.86 (CH-naph), 73.97, 70.06 ($OCH_2$), 32.09, 30.59, 29.83, 29.70, 29.59, 29.53, 26.34, 26.29, 22.86 ($CH_2$), 14.28 ($CH_3$). **IR** (ATR): $\tilde{\nu}$ [cm$^{-1}$] = 2915 s, 2849 s, 1585 m, 1463 m, 1351 m, 1271 s, 1197 w, 1106 m, 1074 s, 994 w, 875 m, 825s, 796 m. **HR-ESI**: calcd. for $C_{34}H_{55}BrO_2$ + $Na^+$: 597.3283; found: 597.3282.

**5,6-Di(dodecyloxy)-2-naphthonitrile**
A mixture of 2.5 g (4.0 mmol) 6-bromo-1,2-di(dodecyloxy)naphthalene, 0.89 g (10.0 mmol) CuCN in 50 mL DMF was stirred at 150 °C until bromonaphthalene had been consumed (48 h, TLC). Aqueous work-up, extraction with ether (Kutscher-Steudel) and recrystallization from ethanol gave 1.8 g (84%) of a colorless solid with m.p. = 55 - 56 °C. **$^{1}$H-NMR** (400 MHz, $CDCl_3$): $\delta$ [ppm] = 8.19 (d, $^{3}J$ = 8.8 Hz, 1H, 4-H, naph), 8.14 (d, $^{4}J$ = 1.6 Hz, 1H, 1-H, naph), 7.61 (d, $^{3}J$ = 9.0 Hz, 1H, 8-H, naph), 7.54 (dd, $^{3}J$ = 8.8, $^{4}J$ = 1.6 Hz, 1H, 3-H, naph), 7.37 (d, $^{3}J$ = 9.0 Hz, 1H, 7-H, naph), 4.15 (t, 2H, $OCH_2$), 4.13 (t, 2H, $OCH_2$), 1.92 – 1.80 (m, 4H, $CH_2$), 1.61 – 1.19 (m, 36H, $CH_2$), 0.90 – 0.86 (m, 6H, $CH_3$). **$^{13}$C-NMR** (75 MHz, $CDCl_3$): $\delta$ [ppm] = 150.55, 142.30 (C-5, C-6), 134.14, (CH-naph), 130.99, 128.09 (C-4a, C-8a), 126.32, 124.65, 123.10 (CH-naph), 119.82 (C-2), 117.59 (CH-naph), 107.19 (CN), 74.04, 69.74 ($OCH_2$), 32.07, 30.55, 29.81, 29.77, 29.69, 29.52, 26.31, 26.25, 22.84 ($CH_2$), 14.27 ($CH_3$). **IR** (ATR): $\tilde{\nu}$ [cm$^{-1}$] = 2916 s, 2848 s, 2359 w, 2219 m, 1791 m, 1731 w, 1622 w, 1596 m, 1484 m, 1463 m, 1354 s, 1280 s, 1195 s, 1161 m, 1103 w, 1072 w, 1022 w, 893 m, 832 m, 800 s, 772 w, 721 m. **HR-ESI**: calcd. for $C_{35}H_{55}NO_2$ + $Na^+$: 544.4130; found: 544.4131.

**5-(5,6-Di(dodecyloxy)naphth-2-yl)-2*H*-tetrazole**

According to the general procedure, 1.20 g (2.2 mmol) 5,6-didodecyloxy-2-naphthonitrile, 0.52 g (8.00 mmol) $NaN_3$, 1.10 g (8 mmol) $NEt_3HCl$ in 50 mL toluene were refluxed. After 24 h, additional reagents (0.52 g $NaN_3$, 1.10 g $NEt_3HCl$) were added and refluxed for further 12 h. Aqueous work-up and recrystallization from ethanol yielded 0.80 g (1.4 mmol, 70 %) of a colorless solid with m.p. = 120 - 122 °C (ethanol). **$^1$H-NMR** (400 MHz, $CDCl_3$/DMSO-$d_6$): $\delta$ [ppm] = 8.48 (s, 1H, 1-H, naph), 8.15 (d, $^3J$ = 8.8 Hz, 1H, 4-H, naph), 8.00 (d, $^3J$ = 8.8 Hz, 1H, 3-H, naph), 7.58 (d, $^3J$ = 9.0 Hz, 1H, 8-H, naph), 7.23 (d, $^3J$ = 9.0 Hz, 1H, 7-H, naph), 4.05 (t, 4H, $OCH_2$), 1.83 – 1.87 (m, 4H, $CH_2$), 1.47 – 1.17 (m, 28H, $CH_2$), 0.80 – 0.77 (m, 6H, $CH_3$). **$^{13}$C-NMR** (100 MHz, $CDCl_3$/DMSO-$d_6$): $\delta$ [ppm] = 156.43 (C-5, tet), 148.70, 141.67 (C-5, C-6, naph), 129.88, 128.34 (C-4a, C-8a, naph), 126.79, 124.19, 123.17, 122.17 (CH-naph), 119.15 (C-2, naph), 116.55 (CH-naph), 73.22, 69.05 ($OCH_2$), 31.31, 29.86, 29.08, 29.04, 28.97, 28.82, 28.75, 25.59, 25.54, 22.07 ($CH_2$), 13.53 ($CH_3$). **IR** (ATR): $\tilde{\nu}$ [cm$^{-1}$] = 3855 w, 3736 w, 2916 s, 2849 s, 2608 m, 2496 m, 1633 w, 1558 s, 1509 m, 1472 s, 1364 m, 1333 m, 1273 s, 1104 m, 1034 m, 901 m, 795 m, 668 m. **HR-ESI**: calcd. for $C_{35}H_{56}N_4O_2 + H^+$: 565.4482; found: 565.4479.

**3,7,11-Tris(5,6-di(dodecyloxy)naphth-2-yl)tris([1,2,4]triazolo)[4,3-a:4',3'-c:4",3"-e]-[1,3,5]triazine *t*-41**

According to the general procedure, 400 mg (0.70 mmol) (5,6-didodecyloxynaphth-2-yl)-*2H*-tetrazole and collidine (0.1 mL) were stirred in xylenes (40 mL) for 30 min. 31 mg (0.17 mmol) cyanuric chloride was added and the stirred for 4 h. After 4 h and 8 h, further 8 mg cyanuric chloride was added. Aqueous work-up and chromatography on a column of basic alumina with a gradient from light petroleum to toluene yielded 250 mg (67 %) of a colorless solid with m.p. = 59 °C (DSC, 2. heating). **$^1$H-NMR** (400 MHz, $CDCl_3$): $\delta$ [ppm] = 8.76 (d, $^4J$ = 1.8 Hz, 1H, 1-H, naph), 8.32 (d, $^3J$ = 8.9 Hz, 1H, 4-H, naph), 8.15 (dd, $^3J$ = 8.9, $^4J$ = 1.8 Hz, 1H, 3-H, naph), 7.75 (d, $^3J$ = 9.0 Hz, 1H, 8-H, naph), 7.36 (d, $^3J$ = 9.0 Hz, 1H, 7-H, naph), 4.17 (t, 4H, $OCH_2$), 1.94 – 1.85 (m, 4H, $CH_2$), 1.59 – 1.20 (m, 36H, $CH_2$), 0.88 (t, 6H, $CH_3$). **$^{13}$C-NMR** (100 MHz, $CDCl_3$): $\delta$ [ppm] = 151.47 (C-5, trl), 149.96, 142.47 (C-5, C-6, naph), 140.90 (C-3, trl), 131.11, 131.00, 128.55, 126.08, 125.49, 122.28, 119.26, 117.16 (C, CH, naph), 74.07, 69.83 ($OCH_2$), 32.09, 30.66, 29.88, 29.83, 29.79, 29.62, 29.54, 26.38, 26.30, 22.85 ($CH_2$), 14.29 ($CH_3$). **IR** (ATR): $\tilde{\nu}$ [cm$^{-1}$] = 2921 s, 2849 s, 1686 w, 1658 w, 1596 s, 1465 m, 1345 w, 1265 m, 1214 w, 1055 w, 695 s. **HR-ESI:** calcd. for $C_{108}H_{165}N_9O_6 + H^+$: 1685.2961; found: 1685.3022.

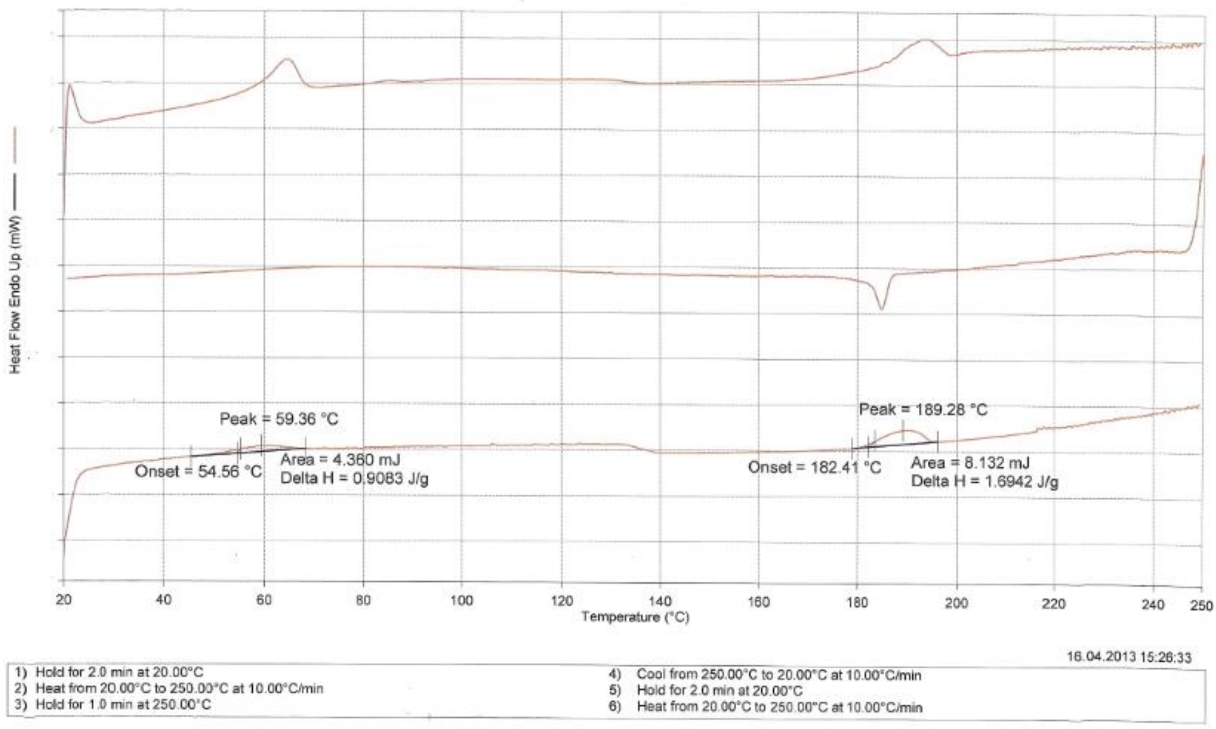


DSC of ***t*-41**

**3,7,11-Tris(3',4'-di(octyloxy)-[1,1'-biphenyl]-4-yl)tris([1,2,4]triazolo)[4,3-a:4',3'-c:4'',3''-e]-[1,3,5]triazine *t*-42**

According to the general procedure, 300 mg (0.63 mmol) 5-(3',4'-di(octyloxy)-[1,1'-biphenyl]-4-yl)-2*H*-tetrazole, 35 mg (0.19 mmol) cyanuric chloride and collidine (0.1 mL) were stirred in xylenes (20 mL). After 4 h , aqueous work-up and chromatography on a column of basic alumina with a gradient from toluene to toluene / ethyl acetate 20 / 1 yielded 118 mg (45 %) of a colorless solid with m.p. = 148 °C (DSC). **$^1$H-NMR** (400 MHz, $CDCl_3$): $\delta$ [ppm] = 8.24 (d, $^3J$ = 8.4 Hz, 2H, 3-H, 5-H, ph), 7.72 (d, $^3J$ = 8.4 Hz, 2H, 2-H, 6-H, ph), 7.15 (m, 2H, 2‘-H, 6‘-H, ph‘), 6.90 (d, $^3J$ = 8.9 Hz, 1H, 5‘-H, ph‘), 4.05 (t, 6H, $OCH_2$), 3.99 (t, 2H, $OCH_2$), 1.87 – 1.77 (m, 4H, $CH_2$), 1.54 – 1.24 (m, 20H, $CH_2$), 0.91 – 0.87 (m, 6H, $CH_3$). **$^{13}$C-NMR** (100 MHz, $CDCl_3$): $\delta$ [ppm] = 150.91 (C-5, trl), 149.48 (C-3‘, C-4‘, ph‘), 144.53 (C-1, ph), 140.69 (C-3, trl), 132.99 (C-1‘, ph‘), 130.73, 126.85 (CH, ph), 122.11 (C-4, ph), 119.96, 113.94, 113.09 (CH, ph‘), 69.57, 69.50 ($OCH_2$), 31.99, 29.56, 29.54, 29.46, 29.42, 26.19, 22.83 ($CH_2$), 14.26 ($CH_3$). **IR** (ATR): $\tilde{\upsilon}$ [cm$^{-1}$] = 2920 s, 2852 m, 1581 vs, 1509 m, 1467 s, 1428 w, 1312 w, 1249 s, 1204 m, 1144 m, 1015 w, 1011 m, 834 m, 800 m, 706 m. **FD-MS:** m/z (%): 713.3 [$M^{2+}$]; 1427.2 [$M^+$]; 2852.2 [$M_2^+$]. **HR-ESI**: calcd. for $C_{90}H_{123}N_9O_6 + H^+$: 1426.9675; found: 1426.9618.

**5-(3',4'-Di(decyloxy)-[1,1'-biphenyl]-4-yl)-2*H*-tetrazole**

In 50 mL of 1,2-dimethoxyethane and water (1:1), 0.69 g (2.5 mmol) 5-(4-iodophenyl)-2*H*-tetrazole, 1.00 g (2.3 mmol) 3,4-(di(decyloxy)phenyl)boronic acid, 0.95 g (7.2 mmol) $K_2CO_3$ and 0.13 g (0.1 mmol, 5 mol%) tetrakis(triphenylphosphin) palladium(0) as catalyst were refluxed until the startin material had been consumed (TLC). The hot mixture was filtered through celite, acidulated with 2 N HCl to pH 2 and the precipitated tetrazole was filtered and recrystallized from chloroform to yield 0.30 g (0.6 mmol, 27 %) of a colorless solid with m. p. = 132 - 133 . **$^1$H-NMR** (400 MHz, $CDCl_3$): $\delta$ [ppm] = 8.18 (d, $^3J$ = 8.1 Hz, 2H, 3-H, 5-H, ph), 7.67 (d, $^3J$ = 8.1 Hz, 2H, 2-H, 6-H, ph), 7.14 (m, 2H, 2‘-H, 6‘-H, ph‘), 6.93 (d, $^3J$ = 8.0 Hz, 1H,

5-H, ph‘), 4.03 (m, 4H, $OCH_2$), 1.85 – 1.75 (m, 4H, $CH_2$), 1.59 – 1.10 (m, 20H, $CH_2$), 0.89 – 0.84 (t, 6H, $CH_3$). **$^{13}$C-NMR** (100 MHz, $CDCl_3$): $\delta$ [ppm] = 156.36 (C-5, tet), 149.63, 149.41 (C-3‘, C-4‘, ph‘), 144.63 (C-1, ph), 132.54 (C-1‘, ph‘), 128.17, 127.67 (CH, ph), 121.21 (C-4, ph), 120.08, 114.05, 112.97 (CH, ph‘), 69.78, 69.56 ($OCH_2$), 32.06, 29.78, 29.73, 29.58, 29.50, 29.45, 29.38, 26.18, 22.83 ($CH_2$), 14.26 ($CH_3$). **IR (ATR):** $\tilde{\nu}$ [$cm^{-1}$] = 2919 s, 2849 s, 1599 m, 1574 w, 1498 m, 1467 m, 1416 m, 1330 m, 1254 m, 1198 m, 1144 m, 1067 m, 1025 w, 987 m, 835 m, 781 m, 754 m, 737 m, 671 m. **HR-ESI**: calcd. for $C_{33}H_{51}N_4O_2$ + $H^+$: 535.4012; found: 535.4004.

**3,7,11-Tris(3',4'-di(decyloxy)-[1,1'-biphenyl]-4-yl)tris([1,2,4]triazolo)[4,3-a:4',3'-c:4'',3''-e]-[1,3,5]triazine *t*-43**

According to the general procedure, 300 mg (0.56 mmol) 5-(3',4'-di(decyloxy)-[1,1'-biphenyl]-4-yl)-2*H*-tetrazole, 31 mg (0.17 mmol) cyanuric chloride and collidine (0.1 mL) were stirred in xylenes (20 mL) and successively heated to 60 °C. After the reaction was completed, aqueous work-up and chromatography on a column of basic alumina with toluene / ethyl acetate 20 / 1 yielded 142 mg (52 %) of a colorless solid with m.p. = 168 °C (DSC). **$^1$H-NMR** (400 MHz, $CDCl_3$): $\delta$ [ppm] = 8.24 (d, $^3J$ = 8.3 Hz, 2H, 3-H, 5-H, ph), 7.75 (d, $^3J$ = 8.3 Hz, 2H, 2-H, 6-H, ph), 7.19 (m, 2H, 2‘-H, 6‘-H, ph), 6.94 (d, $^3J$ = 8.9 Hz, 3H, 5‘-H, ph), 4.08 – 4.01 (m, 4H, $OCH_2$), 1.89 – 1.80 (m, 4H, $CH_2$), 1.49 – 1.28 (m, 28H, $CH_2$), 0.90 – 0.86 (m, 6H, $CH_3$). **$^{13}$C-NMR** (75 MHz, $CDCl_3$): $\delta$ [ppm] = 150.94 (C-5, trl), 149.52, 149.49 (C-3‘, C-4‘, ph‘), 144.57 (C-1, ph), 140.71 (C-3, trl), 132.97 (C-1‘, ph‘), 130.72, 126.87 (CH, ph), 122.10 (C-4, ph), 119.97, 113.96, 113.09 (CH, ph‘), 69.58, 69.49 ($OCH_2$), 32.07, 29.81, 29.75, 29.60, 29.52, 29.43, 26.20, 22.84 ($CH_2$), 14.27 ($CH_3$). **IR** (ATR): $\tilde{\nu}$ [$cm^{-1}$] = 2919 s, 2851 m, 1590 s, 1508 m, 1467 s, 1250 m, 1206 m, 1142 m, 1105 w, 948 w, 867 w, 836 s, 800 m, 708 m. **FD-MS**: m/z (%): 797,3 [$M^{2+}$]; 1594,9 [$M^+$]; 3186,7 [$M_2^+$]. **HR-ESI:** calcd. for $C_{102}H_{147}N_9O_6$ + $H^+$: 1595.1553; found: 1595.1577.

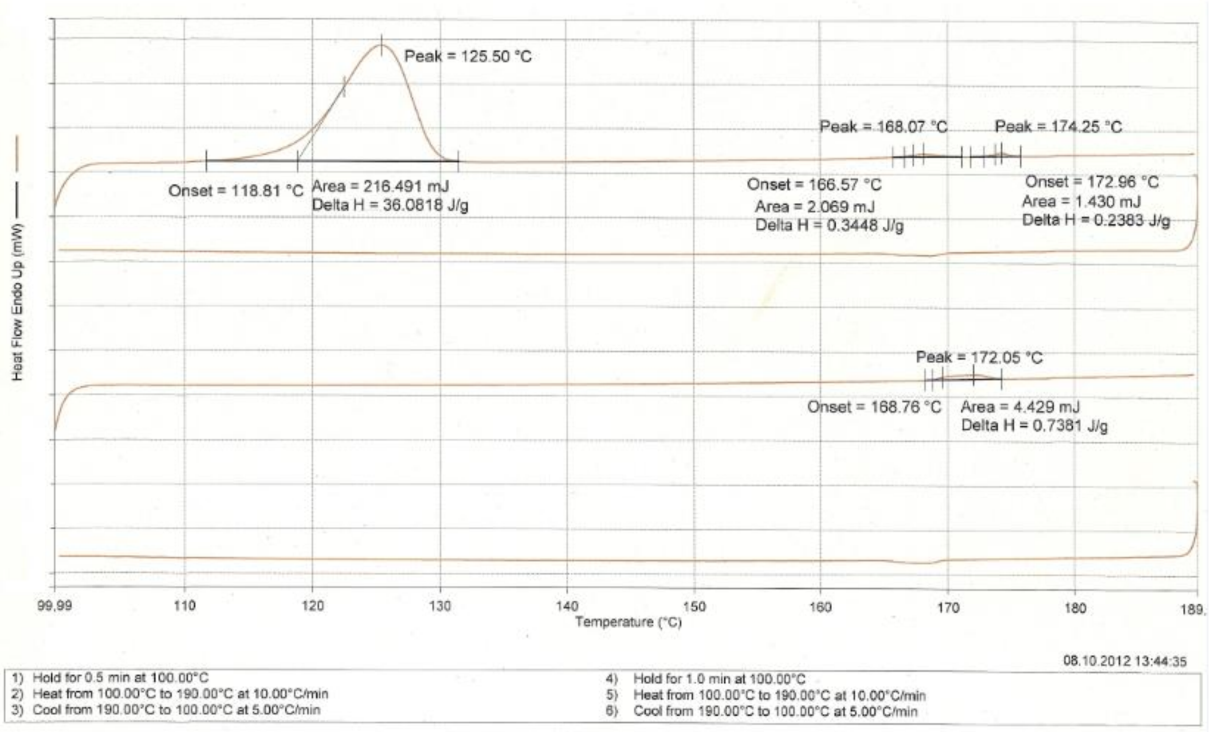


DSC of ***t*-43**

**5-(3',4'-Di(dodecyloxy)-[1,1'-biphenyl]-4-yl)-2*H*-tetrazole**

In 90 mL of toluene and water (2:1), 0.87 g (3.2 mmol) 5-(4-iodophenyl)-2*H*-tetrazole, 1.50 g (3.5 mmol) 3,4-(di(dodecyloxy)phenyl)boronic acid, 1.41 g (10 mmol) $K_2CO_3$ were heated

while stirring to 100 . 0.18 g (0.16 mmol, 5 mol%) tetrakis(triphenylphosphin) palladium(0) as catalyst was added in three portins of 60 mg over three days. The hot mixture was filtered through celite, acidulated with 2 N HCl to pH 2 and the precipitated tetrazole was filtered and recrystallized from chloroform to yield 1.42 g (2.4 mmol, 68 %) of a colorless solid with m. p. = 130 - 131 °C. **$^1$H-NMR** (400 MHz, $CDCl_3$/DMSO-$d_6$): *δ* [ppm] = 8.04 (d, $^3J$ = 8.4 Hz, 2H, 3-H, 5-H, ph), 7.58 (d, $^3J$ = 8.4 Hz, 2H, 2-H, 6-H, ph), 7.06 (m, 2H, 2‘-H, 6‘-H, ph), 6.85 (d, $^3J$ = 8.1 Hz, 1H, 5‘-H, ph), 3.97 – 3.91 (m, 4H, $OCH_2$), 1.77 – 1.69 (m, 4H, $CH_2$), 1.39 – 1.15 (m, 36H, $CH_2$), 0.78 – 0.74 (m, 6H, $CH_3$). **HR-ESI:** calcd. for $C_{37}H_{58}N_4O_2$ + $H^+$: 591.4638; found: 591.4656.

**3,7,11-Tris(3',4'-di(dodecyloxy)-[1,1'-biphenyl]-4-yl)tris([1,2,4]triazolo)[4,3-a:4',3'-c:4'',3''-e]-[1,3,5]triazine *t*-44**

According to the general procedure, 250 mg (0.42 mmol) 5-(3',4'-di(dodecyloxy)-[1,1'-biphenyl]-4-yl)-2*H*-tetrazole, 24 mg (0.17 mmol) cyanuric chloride and collidine (0.1 mL) were stirred for 4 h in xylenes (20 mL). After the reaction was completed, aqueous work-up and chromatography on a column of basic alumina with toluene / ethyl acetate 20 / 1 yielded 100 mg (44 %) of a colorless solid with m.p. = 90 °C (DSC). **$^1$H-NMR** (400 MHz, $CDCl_3$): *δ* [ppm] = 8.24 (d, $^3J$ = 8.4 Hz, 2H, 3-H, 5-H, ph), 7.73 (d, $^3J$ = 8.4 Hz, 2H, 2-H, 6-H, ph), 7.17 – 7.15 (m, 2H, 2‘-H, 6‘-H, ph), 6.92 (d, $^3J$ = 8.9 Hz, 3H, 5‘-H, ph), 4.05 (t, 2H, $OCH_2$), 4.00 (t, 2H, $OCH_2$), 1.86 – 1.80 (m, 4H, $CH_2$), 1.53 – 1.27 (m, 36H, $CH_2$), 0.90 – 0.86 (m, 6H, $CH_3$). **$^{13}$C-NMR (**100 MHz, $CDCl_3$): *δ* [ppm] = 150.93 (C-5, trl), 149.52, 149.50 (C-3‘, C-4‘, ph‘), 144.55 (C-1, ph), 140.70 (C-3, trl), 132.98 (C-1‘, ph‘), 130.72, 126.86 (CH, ph), 122.11 (C-4, ph), 119.96, 113.96, 113.10 (CH, ph‘), 69.59, 69.50 ($OCH_2$), 32.09, 29.88, 29.83, 29.64, 29.61, 29.54, 29.49, 29.44, 26.22, 22.85 ($CH_2$), 14.28 ($CH_3$). **IR (ATR):** $\tilde{\nu}$ [$cm^{-1}$] = 2918 s, 2850 s, 1582 s, 1510 m, 1467 s, 1389 w, 1311 w, 1275 w, 1248 s, 1205 m, 1132 m, 1006 w, 891 w, 836 m, 800 m, 720 m. **FD-MS:** m/z (%): 881.8 (28) [$M^{2+}$]; 1764.1 (78) [$M^+$]. **HR-ESI:** calcd. for $C_{114}H_{171}N_9O_6$ + $H^+$: 1763.3431; found: 1763.3445.

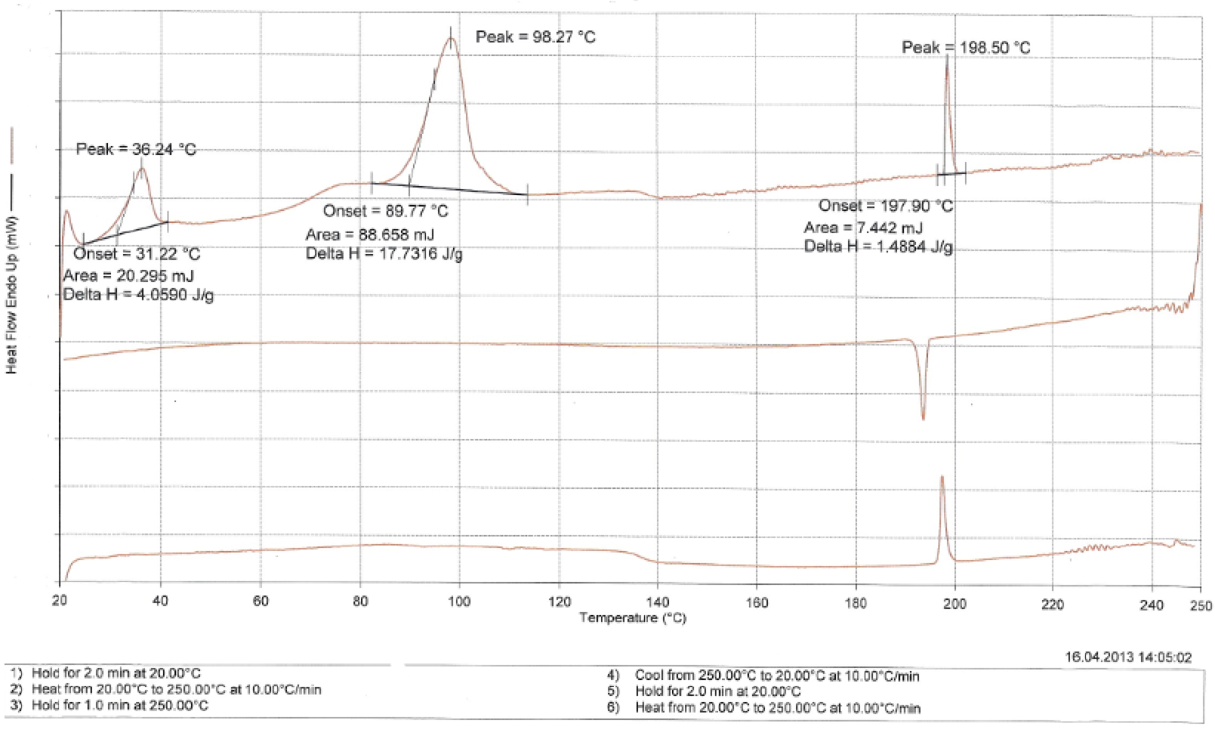


DSC of ***t*-44**

**4-(2,5-Di(decyloxy)phenyl)acetophenone**

8.0 g (35 mmol) 4-(2,5-Dihydroxyphenyl)-acetophenone 19.4 g (140 mmol) $K_2CO_3$, and 25.6 g (120 mmol) 1-bromodecane in 250 mL dimethylformamide were stirred 50 h at 80 °C.

Acidulated with 2N HCl, extraction with light petroleum and usual work-up yielded 16.3 g (32 mmol, 91 %) of a colorless solid. **$^1$H-NMR** (400 MHz, $CDCl_3$): $\delta$ [ppm] = 7.98 (d, $^3J$ = 8.4 Hz, 2H, 3‘-H, 5‘-H, ph‘), 7.65 (d, $^3J$ = 8.4 Hz, 2H, 2‘-H, 6‘-H), 6.96 – 6.80 (m, 3H, 3-H, 4-H, 6-H, ph), 3.94 (t, 2H, $OCH_2$), 3.87 (t, 2H, $OCH_2$), 2.64 (s, 3H), 1.81 – 1.74 (m, 2H, $CH_2$), 1.69 – 1.62 (m, 2H, $OCH_2$), 1.45 – 1.20 (m, 28H), 0.88 (t, 6H, $CH_3$).

**4-(2,5-Di(decyloxy)phenyl)benzoic acid**
Bromine (6.6 g, 2.1 mL) was added to 5.5 M KOH (8,8 g in 40.0 mL $H_2O$) below 10 °C). The mixture was cooled to 0 °C and added dropwise to a solution of 7.0 g (14 mmol) 4-(2,5-di(decyloxy)phenyl)-acetophenone in 20 mL dioxane and stirred for 2 h at ambient temperature and 2 h at 40 °C. 2 N HCl was added until pH 2, extraction with ether, washing, drying and recrystallization from ethanol yielded 6.1 g (12 mmol, 86 %) of a colorless solid with m.p. = 65 – 66 °C (ethanol). **$^1$H-NMR** (400 MHz, $CDCl_3$): $\delta$ [ppm] = 8.14 (d, $^3J$ = 8.4 Hz, 2H, 2-H, 6-H, ph), 7.67 (d, $^3J$ = 8.4 Hz, 2H, 3-H, 5-H, ph), 6.90 (m, 3H, 3‘-H, 4‘-H, 6‘-H, ph‘), 3.95 (t, 2H, $OCH_2$), 3.88 (t, 2H, $OCH_2$), 1.81 – 1.74 (m, 2H, $CH_2$), 1.70 – 1.63 (m, 2H, $CH_2$), 1.47 – 1.24 (m, 28H, $CH_2$), 0.89 – 0.86 (t, 6H, $CH_3$). **IR** (ATR): $\tilde{\nu}$ [cm$^{-1}$] = 2918 s, 2847 m, 1697 vs, 1608 w, 1496 m, 1463 m, 1428 m, 1396 w, 1314 m, 1292 m, 1235 m, 1210 s, 1071 m, 939 w, 848 m, 777 w.

**4-(2,5-Di(decyloxy)phenyl)benzamide**
3.00 g (5,9 mmol) 4-(2,5-di(decyloxy)phenyl)benzoic acid, dissolved in 50 mL toluene was added to 2.2 mL (30.0 mmol) thionyl chloride and refluxed for 12 h. After 12 h, the mixture was concentrated to 5 mL and added dropwise to a heavily stirred, ice-cold solution of ammonia (25 %). The amide was extracted with $CHCl_3$ (3 x 50 mL), the pooled solutions washed, concentrated and recrystallization from ethanol yielded 2.72 g (5.3 mmol, 91 %) of a colorless solid with m.p. = 109 – 111 °C.
**$^1$H-NMR** (400 MHz, $CDCl_3$): $\delta$ [ppm] = 7.84 (d, $^3J$ = 8.4 Hz, 2H, 2-H, 6-H, ph), 7.65 (d, $^3J$ = 8.4 Hz, 2H, 3-H, 5-H, ph), 6.90 (m, 3H, 3‘-H, 4‘-H, 6‘-H, ph‘), 6.20 (s, 1H, $NH_2$), 5.87 (s, 1H, $NH_2$), 3.94 (t, 2H, $OCH_2$), 3.87 (t, 2H, $OCH_2$), 1.81 – 1.73 (m, 2H, $CH_2$), 1.69 – 1.62 (m, 2H, $CH_2$), 1.47 – 1.24 (m, 28H, $CH_2$), 0.88 (t, 6H, $CH_3$). **$^{13}$C-NMR** (100 MHz, $CDCl_3$): $\delta$ [ppm] = 169.97 (C-7), 153.47, 150.29 (C-2‘, C-5‘, ph‘), 143.14 (C-4, ph), 130.74, 130.60 (C-1, ph, C-1‘, ph‘), 129.98, 127.22 (CH, ph), 117.28, 114.84, 114.44 (CH, ph‘), 69.58, 68.85 ($OCH_2$), 32.04, 29.71, 29.69, 29.56, 29.46, 29.40, 26.21, 26.19, 22.83 ($CH_2$), 14.27 ($CH_3$). **IR** (ATR): $\tilde{\nu}$ [cm$^{-1}$] = 3371 w, 3188 w, 2918 s, 2849 m, 1650 vs, 1620 m, 1497 m, 1463 m, 1390 m, 1233 m, 1210 s, 1125 w, 1070 w, 847 w, 719 w. **HR-ESI:** calcd. for $C_{33}H_{51}NO_3$ + $Na^+$: 532.3767; found: 532.3768.

**5-(2',5'-Di(decyloxy)-[1,1'-biphenyl]-4-yl)-*2H*-tetrazole**
Under exclusion of moisture, 1.68 g (26.0 mmol) $NaN_3$ was added to 1.47 g (8.6 mmol) $SiCl_4$ in 30 mL dry acetonitrile and the mixture was stirred for 1 h at ambient temperature. 2.00 g (3,9 mmol) 4-(2,5-di(decyloxy)phenyl)benzamid was added, after 12 h stirring at ambient temperature, further 0.85 g (13 mmol) $NaN_3$ and 0.74 g (4.3 mmol) $SiCl_4$ were added, the mixture was heated for 1 h to 50 °C. Addition of water, extraction with ether (3 x 50 mL), evaporation and recrystallization from ethanol yielded 0.89 g (1.6 mmol, 45 %) of a colorless solid with m.p. = 84 – 86 °C (ethanol). **$^1$H-NMR** (400 MHz, $CDCl_3$): $\delta$ [ppm] = 8.09 (d, $^3J$ = 8.3 Hz, 2H, 3-H, 5-H, ph), 7.74 (d, $^3J$ = 8.3 Hz, 2H, 2-H, 6-H, ph), 6.97 – 6.83 (m, 3H, 3‘-H, 4‘-H, 6‘-H, ph‘), 3.95 (t, 2H, $OCH_2$), 3.89 (t, 2H, $OCH_2$), 1.81 – 1.74 (m, 2H, $CH_2$), 1.71 – 1.63 (m, 2H, $CH_2$), 1.49 – 1.22 (m, 28H, $CH_2$), 0.89 – 0.84 (m, 6H, $CH_3$). **$^{13}$C-NMR** (100 MHz, $CDCl_3$): $\delta$ [ppm] = 153.87, 152.42, 150.35, 120.88, 113.76, 113.74, 111.82, 69.80, 69.05 ($OCH_2$), 32.04, 32.00, 29.72, 29.70, 29.63, 29.52, 29.46, 29.41, 29.38, 26.30, 26.13, 22.83, 22.81 ($CH_2$), 14.27, 14.25 ($CH_3$), signals missing due to poor solubility. **IR** (ATR): $\tilde{\nu}$ [cm$^{-1}$] = 2917 s, 2848 m, 1616 w, 1496 m, 1464 m, 1395 w, 1319 w, 1218 s, 1151 w, 1070 m, 986 w, 940 w, 843 m, 811 m, 759 w, 719 m. **HR-ESI:** calcd. for $C_{33}H_{50}N_4O_2$ + $H^+$: 535.4012; found: 535.4022.

**3,7,11-Tris(2',5'-di(decyloxy)-[1,1'-biphenyl]-4-yl)tris([1,2,4]triazolo)[4,3-a:4',3'-c:4'',3''-e]-[1,3,5]triazine *t*-45**
200 mg (2.0 mmol) 5-(2',5'-Di(decyloxy)-[1,1'-biphenyl]-4-yl)-*2H*-tetrazole and 0.1 mL sym-collidine were stirred in 25 mL dry xylenes. After 30 min, 0.8 mL of a solution of 40 mg cyanuric chloride in 2 mL xylenes were added, the mixture was stirred at ambient temperature and after 4 h, and after 8 h, additional 0.2 mL of the cyanuric chloride solution was added. 10 h stirring and chromatography ($SiO_2$ with $Al_2O_3$-head, toluene/ethyl acetate = 30/1) yielded 130 mg (0.08 mmol, 66 %) of a colorless solid with m.p. = 87 °C. **$^1$H-NMR** (400 MHz, $CDCl_3$): $\delta$ [ppm] = 8.24 (d, $^3J$ = 8.5 Hz, 2H, 3-H, 5-H, ph), 7.80 (d, $^3J$ = 8.5 Hz, 2H, 2-H, 6-H, ph), 7.00 (d, $^4J$ = 3.0 Hz, 1H, 6'-H, ph), 6.92 (d, $^3J$ = 9.0 Hz, 1H, 4'-H, ph), 6.86 (dd, $^3J$ = 9.0, $^4J$ = 3.0 Hz, 1H, 3'-H, ph), 3.96 (t, 2H, $OCH_2$), 3.91 (t, 2H, $OCH_2$), 1.82 – 1.68 (m, 4H, $CH_2$), 1.50 – 1.23 (m, 28H, $CH_2$), 0.90 – 0.83 (m, 6H, $CH_3$). **$^{13}$C-NMR** (100 MHz, $CDCl_3$): $\delta$ [ppm] = 153.47, 151.17, 150.38 (C-5, trl, C-2', C-5', ph'), 142.45 (C-1, ph), 140.77 (C-3, trl), 130.66 (C-1', ph'), 129.83, 129.80 (CH, ph), 122.33 (C-4, ph), 117.04, 115.14, 114.47 (CH, ph'), 69.63, 68.90 ($OCH_2$), 32.04, 29.73, 29.58, 29.55, 29.47, 26.21, 22.82 ($CH_2$), 14.25 ($CH_3$). **IR** (ATR): $\tilde{\nu}$ [cm$^{-1}$] = 2920 s, 2851 m, 1589 s, 149 m, 1464 s, 1391 w, 1293 w, 1205 m, 1046 m, 1012 m, 838 m, 795 m, 708 m, 685 w. **HR-ESI**: calcd. for $C_{102}H_{147}N_9O_6$ + $H^+$: 1595.1553; found: 1595.1490.

**3,7,11-Tris(4´-hexyloxystilben-4-yl)tris([1,2,4]triazolo)[4,3-a:4',3'-c:4'',3''-e][1,3,5]triazine[2] *t*-46**
H-NMR ($CDCl_3$): δ=8.06 (d, *J* = 9 Hz, 6 H), 6.72 (d, 3*J* = 9 Hz, 6 H), 3.17 („t", 12 H), 1.63 (m, 12 H), 1.33 (m, 36 H), 0.90 (t, 18 H). C-NMR: 151.6, 150.3, 140.3, 131.5, 110.6, 109.6, 51.0, 31.7, 27.2, 26.8, 22.7 14.0.

**3,7,11-Tris(3´,5´-dihexyloxystilben-4-yl)tris([1,2,4]triazolo)[4,3-a:4',3'-c:4'',3''-e][1,3,5]triazine *t*-47**
$^1$H-NMR ($CDCl_3$): δ= 0.87 (t, J=6.7 Hz, 18H), 1.18-1.44 (m, 36H), 1.57-1.77 (m, 12H), 3.83 (t, J=6.1 Hz, 12H), 6.23 (s, 3H), 6.46 (s, 6H), 6.89 - 7.06 (m, 6 H), 7.58 (d, 6H), 8.27 (d, J = 8.1 Hz, 6H). $^{13}$C-NMR ($CDCl_3$): δ = 14.4, 22.9, 26.0, 29.5, 32.0, 68.4, 101.0,105.1, 123.1, 126.7, 128.5, 130.9, 131.0, 138.8, 140.6, 140.7, 151.1, 160.8.

**3,7,11-Tris(3´,4´,5´-trihexyloxystilben-4-yl)tris([1,2,4]triazolo)[4,3-a:4',3'-c:4'',3''-e][1,3,5]triazine *t*-48**
$^1$H-NMR ($CDCl_3$): δ= 0.86 (m, 27), 1.15-1.51 (m, 54H), 1.64-1.84 (m, 18H), 3.93 (t, J=6.6 Hz, 6H), 3.99(t, J=6.5 Hz, 12H), 6.69 (s, 6H), 6.98 (d, J = 16.1 Hz, 3H), 7.10 (d, J = 16.1 Hz, 3H), 7.65 (d, J= 8.1 Hz, 6H), 8.22 (d, J= 8.3 Hz, 6H). $^{13}$C-NMR ($CDCl_3$): δ = 14.4, 14.5, 23.0. 23.1, 26.1, 29.7, 30.0, 30.6, 32.0, 32.1, 69.4, 73.9, 105.5, 123.0, 126.6, 126.9, 130.1, 131.5, 132.4, 138.6, 141.0, 151.0, 153.6.

**4-((3, 7-Dimethyloctyl)oxy)benzonitrile**
A solution of 4-(3,7-dimethyloctyloxy)benzaldehyde (2.00 g, 7.6 mmol, 1.0 eq.) and ammonium hydroxide (50 mL, 25%) in THF (70 mL) was cooled to 0 °C. Then, iodine (2.13 g, 8.4 mmol, 1.1 eq.) was added in small portions at 0 °C. After the reaction was finished, the phases of the red reaction mixture were separated and the organic layer was washed with sodium bisulfite solution (3 x 80 mL) and brine (2 x 30 mL) and was dried with magnesium sulfate. Removal of the organic solvent in vacuo gave the crude red product (2.10 g). The crude product was then subjected to column chromatography on silica gel (150 g, fill level: l = 20 cm, d = 10 cm, eluent: toluene) to yield 1.91 g (7.4 mmol, 97%) of an orange oil. ***$R_f$*** = 0.57 ($SiO_2$, toluene).
**$^1$HNMR** (300 MHz, $CDCl_3$, 298 K): δ (ppm) = 7.56 (d, $^3J$HH = 9.0 Hz, 2H, 2'-H, 6'-H), 6.93 (d, $^3J$HH = 9.0 Hz, 2H, 3'-H, 5'-H), 4.13 – 3.87 (m, 2H, 1''-H), 1.92 – 1.75 (m, 1H, 7''-H), 1.72 – 1.45 (m, 3H, 2''-H, 3''-H, sup.), 1.37 – 1.11 (m, 6H, 4''-H – 6''-H, sup.), 0.94 (d, $^3J$HH = 6.4 Hz, 3H, 10''-H), 0.86 (d, $^3J$HH = 6.6 Hz, 6H, 9''-H, 8''-H, sup.). **$^{13}$C-NMR** (75MHz, $CDCl_3$): δ(ppm) = 162.5 (C-4'), 134.0(C-2', C-6', sup.),119.4 (C-1),115.3 (C-3', C-5', sup.),103.7 (C-

1'), 66.9 (C-1''), 39.3 (C-6''), 37.3 (C-4''), 36.0 (C-3''), 29.9 (C-2''), 28.1 (C-7''), 24.7 (C-5''), 22.8 (C-9 *C*H3), 22.7 [C-9'', C-8''], 19.7 (C-10''). **FD-MS:** *m/z* (%) = 259.6 (100.0)[$M^+$], 260.6 (16.5), 261.6 (1.7). **IR:** v[$cm^{-1}$] = 2925 br s., 2224 s, 1706 w, 1605 ss, 1574 m, 1508 ss, 1470 s, 1385 m, 1302 s, 1256 ss, 1171 ss, 1113w, 1047 w, 1010 m, 833 ss, 707 m, 667 m. **EA:** $C_{17}H_{25}NO$ (259.39) calcd.(%) C 78.72, H 9.71, N 5.40, found (%) C 78.69, H 9.46, N 6.02.

**5-(4-((3, 7-Dimethyloctyl)oxy)phenyl)-2*H*-tetrazole**
A solution of 4-((3,7-dimethyloctyl)oxy)benzonitrile (1.00 g,3.9mmol, 1.0 eq.), sodium azide (0.88 g, 13.5 mmol, 3.5 eq.) and triethylammonium chloride (1.86 g, 13.5 mmol, 3.5 eq.) in THF (30 mL) was heated to reflux (bath temperature: 90 °C) over night. After reaction was finished (TLC control, eluent toluene/ ethyl acetate 3:1) 2N HCl (20 mL) was added and the reaction mixture was extracted with ethyl acetate (3 x 50 mL). The combined organic layers were washed with water (2 x 30 mL) and brine (2 x 30 mL) and were dried with magnesium sulfate. Removal of the organic solvent in vacuo gave the crude product which was then recrystallized from chloroform to yield 558 mg (48%) of a colorless solid with m.p.: 115 °C (chloroform).
**$^1$HNMR** (300 MHz, $CDCl_3$, 298 K): δ (ppm) = 8.17 – 7.93 (m, 2H, 2'-H, 6'-H), 7.11 – 6.80 (m, 2H, 3'-H, 5'-H), 4.19 – 3.85 (m, 2H, 1''-H), 1.93 – 1.75 (m, 1H, 7''-H), 1.75 – 1.45 (m, 3H, 2''-H, 3''-H, sup.), 1.40 – 1.07 (m, 6H, 4''-H – 6''-H, sup.), 0.94 (d, $^3J$HH = 6.4 Hz, 3H, 10''-H), 0.86 (d, $^3J$HH = 6.6 Hz, 6H, 9''-H, 8''-H, sup.). **$^{13}$C-NMR** (75MHz, $CDCl_3$): δ(ppm) = 162.2 (C-4'), 156.2 (C-5), 129.4 (C-2', C-6', sup.), 115.6 (C-3', C-5', sup.), 115.3 (C-1'), 66.8 (C-1''), 39.4 (C-6''), 37.4 (C-4''), 36.2 (C-3''), 30.0 (C-2''), 28.1 (C-7''), 24.8 (C-5''), 22.8, 22.7 [C-9'', C-8''], 19.8 (C-10''). **FD-MS:** *m/z* (%) = 274.6 (1.8), 302.6 (13.21) [$M^+$], 303.7 (6.0), 579.9 (3.1), 580.8 (1.1), 603.8 (1.9), 604.8 (2.1), 605.9 (100.0) [$M_2^+$], 606.9 (46.1), 607.8 (8.5), 608.9 (1.0), 907.7 (2.9) [$M_3^+$], 908.8 (1.7). **IR:** v[$cm^{-1}$] = 3903 w, 3853 w, 3838 w, 3820 w, 3800 w, 3726 w, 3709 w, 3676 w, 3649 w, 3628 w, 3596 w, 3567 w, 3066 w, 2925 br s, 2925 br s, 2868 br m, 2716 br m, 2618 br m, 2553 br m, 2470 br m, 1895 br w, 1772 w, 1716 w, 1698 w, 1684 w, 16.47 w, 1613 ss, 1583m, 1558 w, 1541 w, 1500 ss, 1468 m, 1444 w, 1406 m, 1384 w, 1365 w, 1309 w, 1294 s, 1260 ss, 1178 s, 1165 m, 1119 w, 1056 s, 1023 s, 990 s, 933 w, 879 w, 837 ss, 752 ss, 700 m, 668 m, 658 m. **EA:** $C_{17}H_{26}N_4O$ (302.41) calcd.(%) C 67.52, N 18.53, found (%) C 67.23, N 18.72.

**3,7,11-Tris(4-((3,7-dimethyloctyl)oxy)phenyl)tris([1,2,4]triazolo)[4,3-a:4',3'-c:4'',3''-e][1,3,5]triazine *t*-53**
A suspension of 5-(4-((3,7-dimethyloctyl)oxy)phenyl)-2*H*-tetrazole **(46)** (233 mg, 0.77 mmol, 3.3 eq.), cyanuric chloride **(35)** (43 mg, 0.23 mmol, 1eq.) and 2,4,6-collidine (1 mL) in dry xylene (10 mL) was stirred for one day at room temperature and under nitrogen as the inert gas. Afterwards, the reaction mixture was slowly heated to 70 °C (heating rate: ca. 10 °C/ h) and the resulting mixture was heated at 70 °C for 4 d and then for one day at 105 °C. Then 2N HCl (10 mL) was added and the mixture was extracted with ethyl acetate (4 x 30 mL). The combined organic layers were washed with water and with brine and dried with magnesium sulfate. Removal of the organic solvents in vacuo gave the crude product mixture (275 mg) which was adsorbed on a small amount of silica gel (ca. 5 g) and then subjected to column chromatography on basic alumina (15 g, eluent: toluene/ethyl acetate, 15:1) to yield 124 mg (0.39 mmol, 58%) of **54** as a viscous yellow product. Due to contamination with grease the produkt was subjected to column chromatography on silica gel (10 g, fill level: l = 5cm, d = 1 cm, eluent: cyclohexene, afterwards dichloromethane).
**$^1$HNMR** (300 MHz, $CDCl_3$, 298 K): δ (ppm) = 8.07 (d, $^3J$HH = 8.9 Hz, 6H, 2'-H, 6'-H), 7.05 (d, $^3J_{HH}$ = 8.9 Hz, 6H, 3'-H, 5'-H), 4.11 – 4.02 (m, 6H, 1''-H), 1.91-1.82 (m, 3H, 7''-H), 1.77 – 1.46 (m, 11H, 2''-H, 3''-H, sup.), 1.38 – 1.17 (m, 25H, 4''-H – 6''-H, sup.), 0.97 (d, $^3J_{HH}$ = 6.4 Hz, 9H, 10''-H), 0.88 (d, $^3J$HH = 6.6 Hz, 19H, 8''-H, 9''-H, sup.). ). **$^{13}$C-NMR** (75MHz, $CDCl_3$): δ(ppm) = 162.1 (C-4'), 151.0 (C-4), 140.6 (C-1), 131.9 (C-2', C-6', sup.), 115.9 (C-1'), 114.6 (C-3', C-5', sup.), 66.7 (C-1''), 39.4 (C-6''), 37.4 (C-4''), 36.2 (C-3''), 30.0 (C-2''), 28.1 (C-7''), 24.8 (C-5''), 22.9, 22.8 [C-9'', C-8''], 19.76 (C-10''). **FD-MS:** *m/z* (%) = 895.9 (8.0), 896.9 (10.7), 897.9 (100.0) [M+], 898.9 (55.9), 899.9 (19.6), 900.9 (7.2), 902.0 (2.4). **IR:** v[$cm^{-1}$] = 3076 w, 2952 br s, 2925 br s, 2869 br s, 1609 ss, 1593 ss, 1541 w, 1523w, 1485 ss, 1471 ss,

1428 m, 1383 w, 1365 w, 1308 m, 1294 m, 1252 ss, 1178 ss, 1119 w, 1096 w, 1048 w, 1007 m, 976 w, 952 w, 916 w, 881 w, 832 ss, 730 s, 697 m, 667 m.
**EA:** $C_{54}H_{75}N_9O_3$ (898.23) calcd.(%) C 72.21, N 14.03, found (%) C 72.39, N 13.66.

**3,7,11-Tris(4-(neomenthyloxy)phenyl)tris([1,2,4]triazolo)[4,3-a:4',3'c:4'',3''-e][1,3,5]triazine *t*-54** [5]
A suspension of 5-(4-((2-isopropyl-5-methylcyclohexyl)oxy) phenyl)-2*H*-tetrazole (500 mg, 1.66 mmol, 3.3 eq), cyanuric chloride **(35)** (93 mg, 0.50 mmol, 1.0 eq.) and 2,4,6-collidine (1 mL) in dried xylene (10 mL) was stirred over night at room temperature and under nitrogen as the inert gas. Afterwards, the reaction mixture was slowly heated to 70 °C (heating rate: ca. 10 °C/h) and the resulting mixture was then heated at 70 °C for 4 d. After this time, 2N HCl (10 mL) was added and the mixture was extracted with ethyl acetate (4 x 30 mL). The combined organic layers were washed with water (2 x 50 mL) and brine (80 mL) and dried with magnesium sulfate. Removal of the organic solvents in vacuo gave the crude product mixture (929 mg). The crude product was adsorbed on a small amount of silica gel (ca. 5 g) and then subjected to column chromatography on basic alumina (20 g, eluent: toluene/ethyl acetate, 19:1) to yield 316 mg (0.35 mmol, 70%) of **t-9b** as a rosy solid. Crystallization from dichloromethane/ methanol resulted in colorless needles with m.p.: 185 °C (dichloromethane/ methanol). **$^1$HNMR** (300 MHz, $CDCl_3$, 298 K): δ (ppm) = 8.41 – 7.74 (m, 6H, 2'-H, 6'-H), 7.13 – 6.91 (m, 6H, 3'-H, 5'-H), 4.76 (s, 3H, 1''-H), 2.17 (d, $^3J$HH = 15.3 Hz, 3H, 2''-H), 1.84 – 1.61 (m, 15H), 1.15 – 0.98 (m, 8H), 0.95 (d, $^3J$HH = 6.7 Hz, 10H, 10´´-H), 0.88 (d, $^3J$HH = 6.7 Hz, 9H), 0.85 (d, $^3J$HH = 6.5 Hz, 10H ). **$^{13}$C-NMR** (75MHz, $CDCl_3$): δ(ppm) = 161.4 (C-4'), 151.2 (C-4), 140.6 (C-1), 132.1 (C-2', C-6', overlapped.), 115.4 (C-3', C-4', C-5', overlapped), 73.7 (C-1''), 47.8 (C-2''), 37.7 (C-4''), 35.1 (C-6''), 29.4 (C-5''), 26.4 (C-3''), 25.0 (C-7''), 22.4 (C-10''), 21.2, 21.0 (C-9'', C-8'' ). **FD-MS:** *m/z* (%) = 611.8 (0.6), 636.8 (1.5) [$M^+$ – 2 neomethyl], 637.8 (0.6), 753.7 (0.6), 754.9 (1.1) [$M^+$ – neomenthyl], 889.9 (1.04), 891.0 (1.2), 891.8 (100.0) [$M^+$], 892.9 (61.0), 893.9 (26.5), 894.8 (3.0), 1782.5 (3.1), 1783.8 (5.6) [$M_2^+$], 1785.9 (0.7). **IR:** ν[$cm^{-1}$] = 2945 s, 2924 s, 2868 m, 2845 w, 1592 ss, 1530 w, 1480 ss, 1457 w, 1427 m, 1369 w, 1290 m, 1252 ss, 1223 w, 1200 m, 1176 s, 1150 m, 1121 w, 1025 m, 1012 m, 963 m, 940 m, 910 w, 892 w, 832 s, 730 s, 708 w. **EA:** $C_{54}H_{69}N_9O_3$ (892.18) calcd.(%) C 72.70, H 7.80, N 14.13, found (%) C 72.36, H 7.86, N 14.09.

**3,4-Bis(2-ethylhexyloxy)benzonitrile**

1.0 g (7.4 mmol) 4-Cyanocatechol, 4.5 g (33 mmol) $K_2CO_3$ in 60 mL *N,N*-dimethylformamid and 2.91 mL (3.14 g, 16.3 mmol) 3-(bromomethyl)heptane gave, according to the general procedure, gave 2.66 g (6.40 mmol, 86 %) of a colorless oil. **$^1$H-NMR** (300 MHz, $CDCl_3$): *δ* / ppm = 7.22 (dd, $^3J$ = 8.3, $^4J$ = 1.9 Hz, 1H, 6-H (Ph)), 7.06 (d, $^4J$ = 1.9 Hz, 1H, 2-H (Ph)), 6.86 (d, $^3J$ = 8.3 Hz, 1H, 5-H (Ph)), 3.87 (m, 4H, $OCH_2$), 1.76 (m, 2H, CH), 1.68 – 1.18 (m, 16H, Alkyl), 1.15 – 0.72 (m, 12H, $CH_3$). **$^{13}$C-NMR** (75 MHz, $CDCl_3$): *δ* / ppm = 153.50 (C-4(Ph)), 149.51(C-3 (Ph)), 126.30 (CN), 119.71 (C-6 (Ph)), 115.70 (C-1 (Ph)), 112.51 (C-5 (Ph)), 103.40 (C-2 (Ph)), 71.76, 71.46 ($OCH_2$), 39.54, 39.48, 30.66, 29.21, 24.01, 23.77, 23.16 (Alkyl), 14.20, 11.31 ($CH_3$). **IR** (ATR): $\tilde{\nu}$ / $cm^{-1}$ = 2958 m, 2926 m, 2870 m, 2224 w, 1597 w, 1513 s, 1463 m, 1266 ss, 1135 s, 1016 m, 850 w, 806 m. **HRMS-ES(+)**: $[M+H]^+$ calcd.: 382.2722; found: 382.2726.

**5-(3,4-Bis(2-ethylhexyloxyphenyl)tetrazole**
1.96 g (5.45 mmol) 3,4-Bis(2-ethylhexyloxy)benzonitrile, 1.24 g (19.1 mmol) $NaN_3$ 2.63 (19.1 mmol) $NEt_3HCl$ in 50 mL Toluol were stirred for 6 days at 110 °C erhitzt. The cooled mixture was diluted with water (100 mL) and acidulated with 2M HCl to pH 2. Toluene was evaporated, the residue was extracted with ethyl acetate and the organic layer, after drying with $MgSO_4$ , was concentrated and the residue purified by column chromatography (toluene/ethyl acetate = 10:1, $SiO_2$) to yield 1.52 g (3.78 mmol, 69 %) of a colorless oil.
**$^1$H-NMR** (300 MHz, $CDCl_3$): *δ* / ppm = 8.85 (s, br, 1H, Tetrazol-H), 7.76 – 7.68 (m, 2H, 6-H, 2-H (Ph)), 6.95 (d, $^3J$ = 8.2 Hz, 1H, 5-H (Ph)), 4.05 – 3.73 (m, 4H, $OCH_2$), 1.88 – 1.63 (m, 2H, CH), 1.61 – 1.11 (m, 16H, Alkyl), 1.02 – 0.77 (m, 12H, $CH_3$). **IR** (ATR): $\tilde{\nu}$ / $cm^{-1}$ = 2958 m,

2927 m, 2872 m, 1608 m, 1507 s, 1460 s, 1263 ss, 1232 s, 1136 m, 1027 m, 891 w, 811 w.

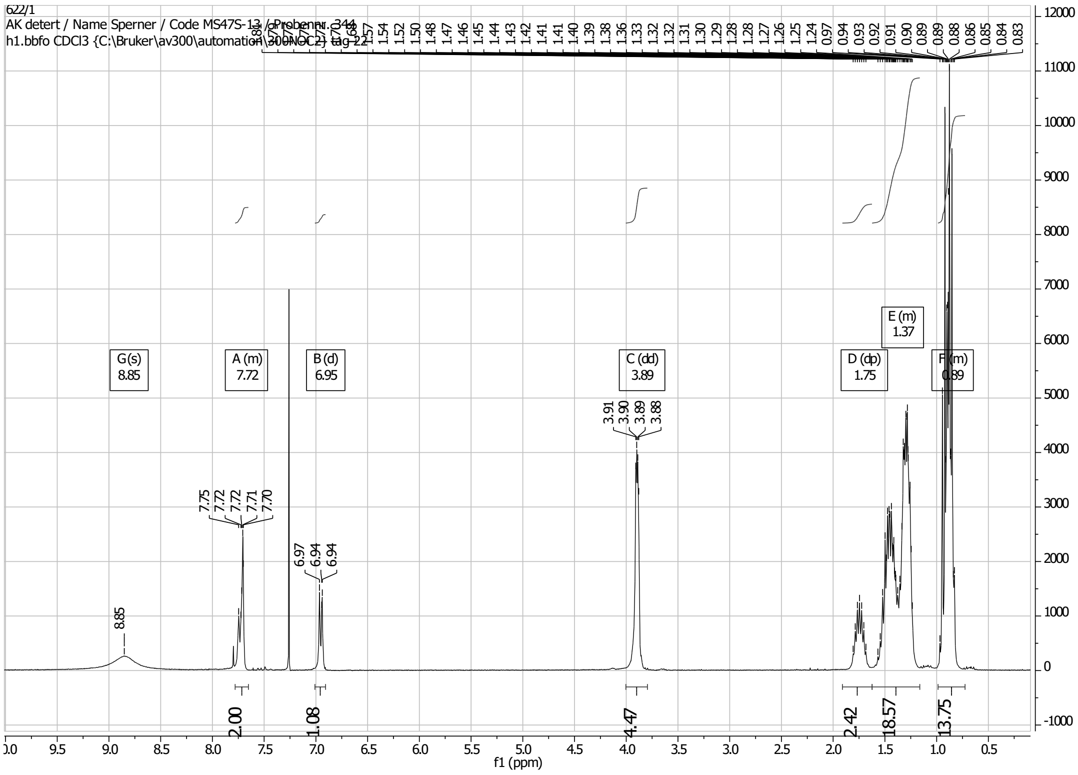


$^{1}$H-NMR of 5-(3,4-Bis(2-ethylhexyloxyphenyl)tetrazole

**3,7,11-Tris{3,4-bis(2-ethylhexoxy)phenyl}tris([1,2,4]triazolo)[4,3-a:4',3'-c:4",3"-e][1,3,5]triazine *t*-56**

5-(3,4-Bis(2-ethylhexyloxy)phenyl)tetrazole **(**745 µmol, 300 mg**)** and (146 mg, 1.20 mmol, 160 µL) 2,4,6-collidine in 20 mL dry toluene were stirred for 1 h and cyanuric chloride (90 mg, 745 µmol, 100 µL) in toluene (c = 10 mg mL$^{-1}$) added and stirring was continued for 3 days. After the addition of further $C_3N_3Cl_3$ (40 mg) in toluene and stirring for 3 days, aqueous work-up and chromatography on silica gel with a head of basic alumina and toluene ethyl acetate (75/1) as eluent yielded 246 mg (205 µmol, 83 %) of a colorless solid with clearing point 157 °C (DSC).

**$^{1}$H-NMR** (300 MHz, $CDCl_3$): $\delta$ / ppm = 7.84 (dd, $^{3}J$ = 8.4, $^{4}J$ = 2.0 Hz, 1H, 6-H (ph)), 7.66 (d, $^{4}J$ = 2.0 Hz, 1H, 2-H (ph)), 7.03 (d, $^{3}J$ = 8.4 Hz, 1H, 5-H (ph)), 4.12 – 3.81 (m, 4H, $OCH_2$), 1.80 (m, 2H, CH), 1.68 – 1.19 (m, 16H, $CH_2$), 1.14 – 0.73 (m, 12H, $CH_3$). **$^{13}$C-NMR** (75 MHz, $CDCl_3$): $\delta$ / ppm = 152.74 (C-5 (triaz), 151.26 (C-4 (ph)), 149.26 (C-3 (ph)), 140.64 (C-3 (triaz)), 123.83 (C-6 (ph)), 115.77 (C-1 (ph)), 114.80 (C-5 (ph)), 112.21 (C-2 (ph)), 71.84 ($OCH_2$), 71.41 ($OCH_2$), 39.62, 39.56, 30.72, 29.25, 24.06, 23.23 ($CH_2$), 14.25, 11.35 ($CH_3$). **IR** (ATR): $\tilde{\nu}$ / cm$^{-1}$ = 2925 s, 2865 s, 1583 s, 1464 ss, 1259 ss, 1146 m, 1023 m, 809 w. **FD-MS:** *m/z* (%) = 1197.8 (100), 1198.7 (70), 1199.8 (20) [M]$^{+}$. **EA:** calcd. for $C_{72}H_{111}N_9O_6$ (1198.71): 72.15% C, 9.33% H, 10.52% N, found: 71.75% C, 8.67% H, 10.33% N.

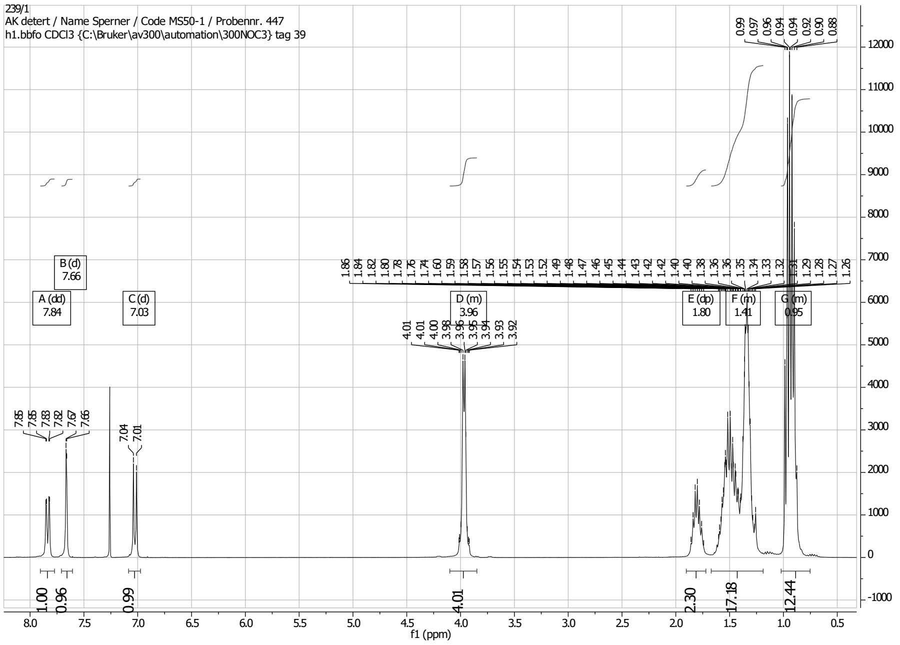
239/1
AK detert / Name Sperner / Code MS50-1 / Probennr. 447
h1.bbfo CDCl3 {C:\Bruker\av300\automation\300NOC3} tag 39
A (dd) 7.84
B (d) 7.66
C (d) 7.03
D (m) 3.96
E (dp) 1.80
F (m) 1.41
G (m) 0.95
f1 (ppm)

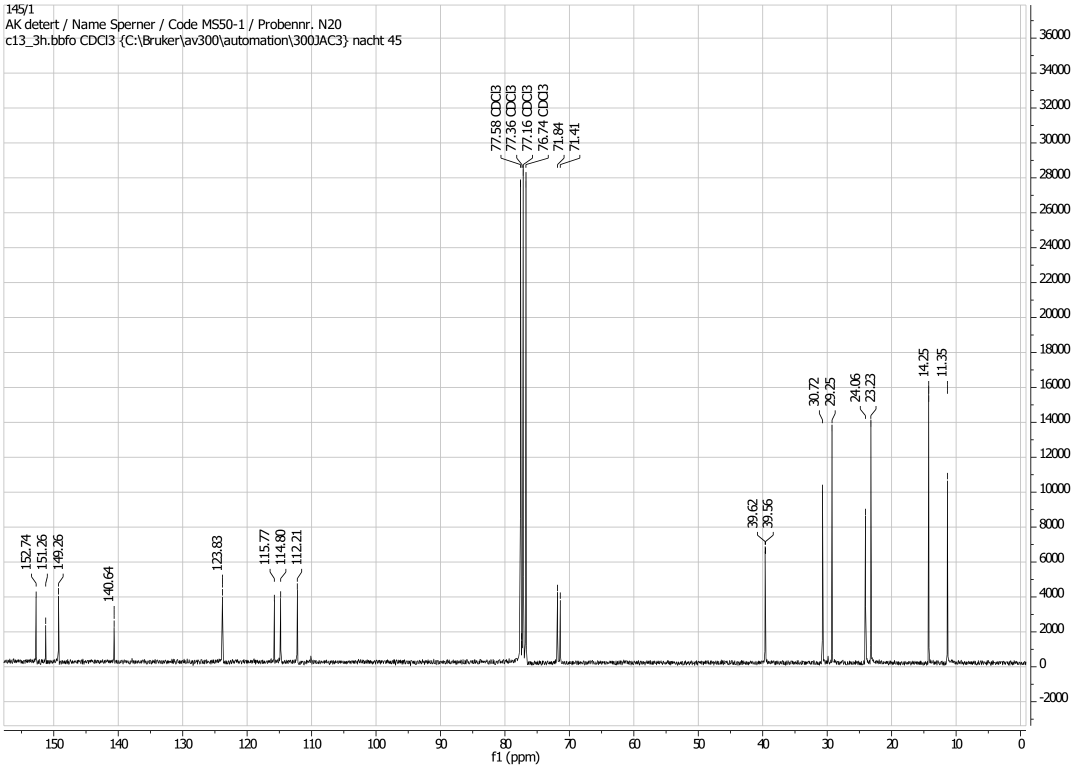
145/1
AK detert / Name Sperner / Code MS50-1 / Probennr. N20
c13_3h.bbfo CDCl3 {C:\Bruker\av300\automation\300JAC3} nacht 45
77.58 CDCl3
77.36 CDCl3
77.16 CDCl3
76.74 CDCl3
f1 (ppm)

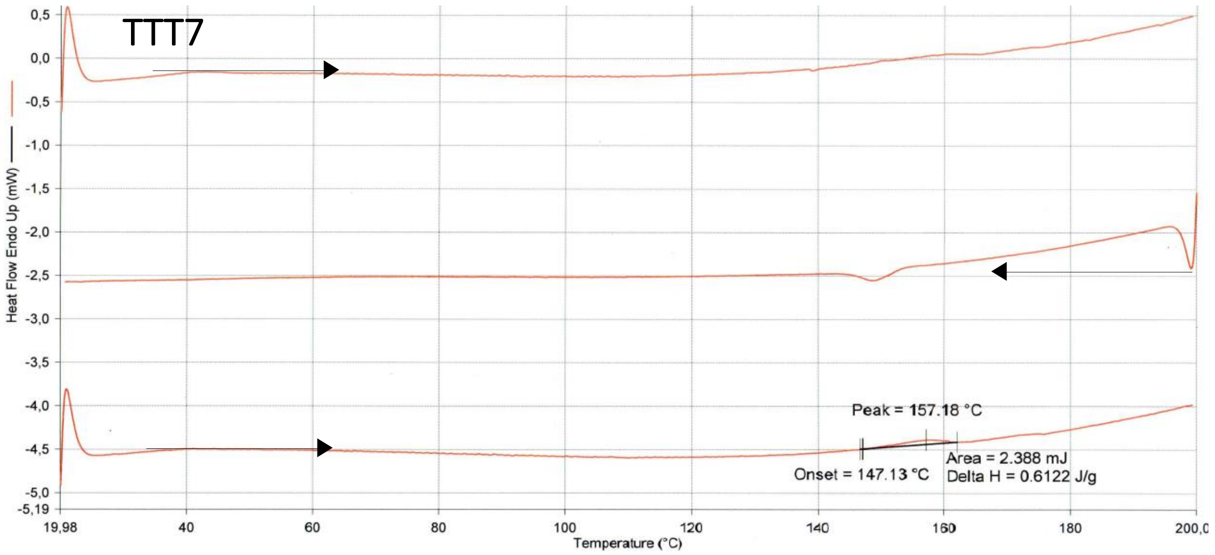


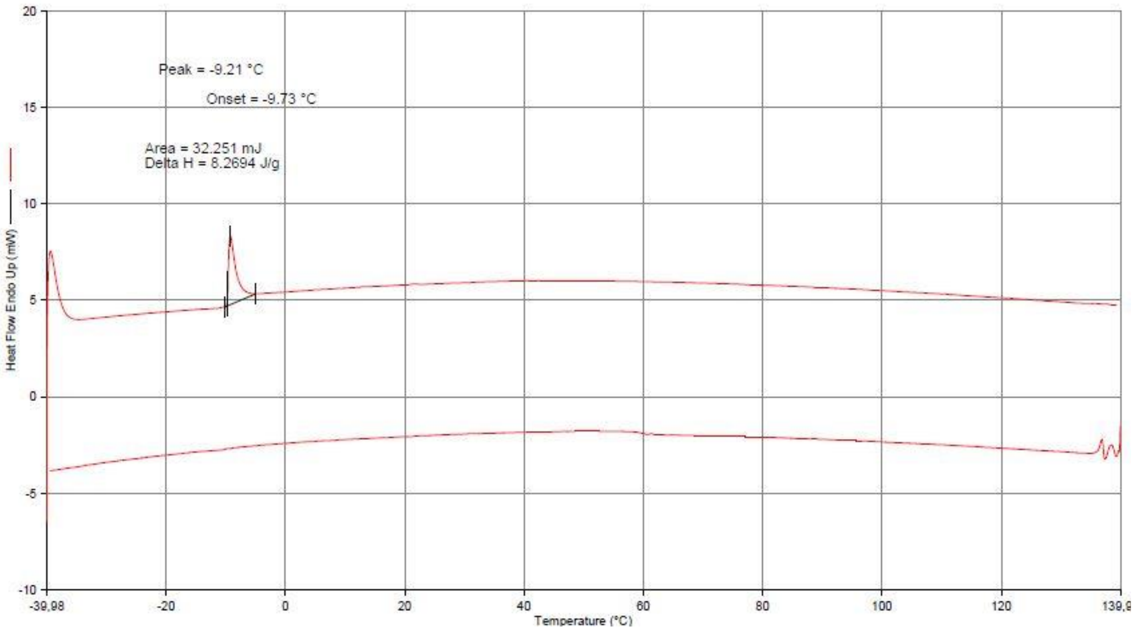


DSC, $^{1}$H- and $^{13}$C-NMR and spectra of ***t*-56**

**3,4-Di((4-ethyloctyl)oxy)benzonitrile**
According to the general procedure, 1.00 g (7.5 mmol) 3,4-dihydroxybenzonitrile, 3.07 g (22 mmol) potash, 3.60 g (16 mmol) 1-bromo-4-ethyloctane in 50 mL DMF under nitrogen stirred at 80 °C for 16 h Additional 1.00 g potash and 1.2 g 1-bromo-4-ethyloctane and stirring for 12 h until reaction was finished (TLC). Acidulation with 2 N HCl, extraction with light petroleum (3*30 ml), brine and $MgSO_4$ gave, 3.14 g (7.1 mmol, 94 %) of a colorless oil.
**$^{1}$H-NMR** (400 MHz, $CDCl_3$): *δ* [ppm] = 7.23 (dd, $^{3}J$ = 8.3, $^{4}J$ = 1.9 Hz, 1H, H-6, ph), 7.06 (d, $^{4}J$ = 1.9 Hz, 1H, H-2, ph), 6.86 (d, $^{3}J$ = 8.3 Hz, 1H, H-5, ph), 4.03 – 3.96 (m, 4H, $OCH_2$), 1.87 – 1.74 (m, 4H, $CH_2$), 1.46 – 1.19 (m, 28H, $CH_2$), 0.94 – 0.79 (m, 12H, $CH_3$). **$^{13}$C-NMR** (100 MHz, $CDCl_3$): *δ* [ppm] = 153.11, 149.11 (C-3, C-4), 126.39 (CH-ph), 119.57 (C-1), 115.95 (CH-ph), 112.70 (CH-ph), 103.59 (C-7, CN), 69.87, 69.57 ($OCH_2$), 38.66, 32.87, 29.25,

29.23, 29.03, 26.33, 26.25, 25.90, 23.24 ($CH_2$), 14.27, 10.93 ($CH_3$). **IR** (ATR): $\tilde{\upsilon}$ [$cm^{-1}$] = 2925 m, 2859 m, 2224 w, 1597 w, 1513 s, 1465 m, 1420 w, 1267 s, 1135 s, 1014 m, 851 w, 806 w. **HR-ESI:** calcd. for $C_{27}H_{45}NO_2 + Na^+$: 438.3348; found: 438.3345.

**5-(3,4-Di((4-ethyloctyl)oxy)phenyl)-2*H*-tetrazole**
According to the general procedure, 2.00 g (4.80 mmol) 3,4-di(4-ethyloctyloxy)benzonitrile, 1.11 g (17.00 mmol) $NaN_3$, 2.32 g (17 mmol) $NEt_3HCl$ in 50 mL toluene, 16 h reflux. Then additional 0.32 g (4.8 mmol) $NaN_3$ and 0.66 g (4.8 mmol) triethylammonium chloride, 12 h reflux. Yield: 1.80 g (3.90 mmol, 82 %) of a colorless solid with m.p. = 65 - 68 °C (toluene).
**$^1$H-NMR** (400 MHz, $CDCl_3$): $\delta$ [ppm] = 7.66 – 7.64 (m, 2H, H-2, H-6, ph), 6.96 (d, $^3J$ = 8.9 Hz, 1H, H-5, ph), 4.04 – 4.00 (m, 4H, $OCH_2$), 1.84 – 1.73 (m, 4H, $CH_2$), 1.41 – 1.21 (m, 22H, $CH_3$), 0.89 – 0.79 (m, 12H, $CH_3$). **$^{13}$C-NMR** (100 MHz, $CDCl_3$): $\delta$ [ppm] = 156.42 (C-5, tet), 152.20, 149.71 (C-3, C-4, ph), 120.74 (ph-CH), 115.56 (C-1, ph), 113.22 (ph-CH), 112.06 (ph-CH), 69.94, 69.71 ($OCH_2$), 38.69, 32.89, 29.29, 29.05, 26.44, 26.38, 25.91, 25.87, 23.26 ($CH_2$), 14.29 ($CH_3$), 10.94 ($CH_3$).
**IR** (ATR): $\tilde{\upsilon}$ [$cm^{-1}$] = 2927 m, 2861 m, 1609 w, 1514 s, 1440 m, 1392 m, 1268 s, 1229 s, 1135 s, 1040 m, 868 m, 808 m, 753 m, 716 m. **HR-ESI:** calcd. for $C_{27}H_{46}N_4O_2 + Na^+$: 481.3518; found: 481.3536.

**3,7,11-Tris(3,4-di((4-ethyloctyl)oxy)phenyl)tris([1,2,4]triazolo)[4,3-a:4',3'-c:4'',3''-e]-[1,3,5]triazine *t*-57**

According to the general procedure, 0.50 g (1 mmol) 5-(3,4-di(4-ethyloctyloxy)phenyl)-2*H*-tetrazole and 0.2 mL *sym*-collidine in 30 mL xylenes were stirred for 60 min. Addition of 0.5 mL of a 0.6 M solution of cyanuric chloride in toluene to the stirred mixture. After 4 h, 0.1 mL of this solution was added and repeated every 2 h until the reaction was complete (TLC). Dilution with water, 50 mL 2N HCl and extraction with ethyl acetate (3 x 20 mL) gave the crude product that was absorbed on 0.5 g silica gel. Chromatography on a silica columns with head of basic alumina with toluene, followed by toluene/ethyl acetate 30/1 gave 0.44 g (0.32 mmol, 88 %) of a colorless wax with m.p. = 72 °C (DSC, 2$^{nd}$ heating).
**$^1$H-NMR (400 MHz, $CDCl_3$):** $\delta$ [ppm] = 7.82 (dd, $^3J$ = 8.5, $^4J$ = 2.1 Hz, 3H, H-6, ph), 7.69 (d, $^4J$ = 2.1 Hz, 3H, H-2, ph), 7.02 (d, $^3J$ = 8.5 Hz, 3H, H-5, ph), 4.07 (t, 12H, $OCH_2$), 1.89 – 1.80 (m, 12H, $CH_2$), 1.47 – 1.20 (m, 66H, $CH_2$), 0.92 – 0.83 (m, 36H, $CH_3$). **$^{13}$C-NMR** (100 MHz, $CDCl_3$): $\delta$ [ppm] = 152.32 (C-4, ph), 151.19 (C-5, trl), 148.82 (C-3, ph), 140.64 (C-3, trl), 123.89 (C-6, ph), 115.95 (C-1, ph), 114.95 (C-5, ph), 112.47 (C-2, ph), 69.87, 69.58 ($OCH_2$), 38.73, 32.91, 32.89, 29.37, 29.35, 29.06, 29.05, 26.54, 26.44, 25.94, 25.91, 23.26 ($CH_2$), 14.28 ($CH_3$), 10.96 ($CH_3$). **IR** (ATR): $\tilde{\upsilon}$ [$cm^{-1}$] = 2925 s, 2858 m, 1596 m, 1527 w, 1491 s, 1466 s, 1379 w, 1327 w, 1259 vs, 1224 m, 1141 m, 1011 m, 878 w, 805 m, 714 w.
**HR-ESI:** calcd. for $C_{84}H_{135}N_9O_6 + H^+$: 1367.0614; found: 1367.0619.

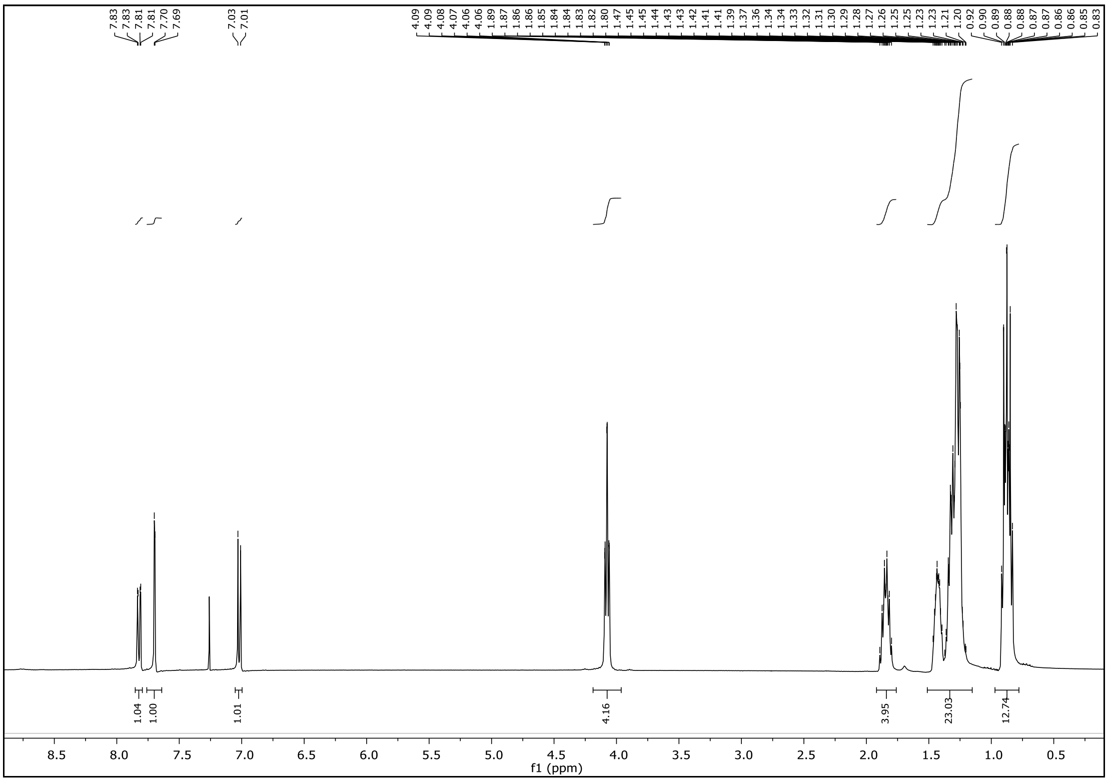


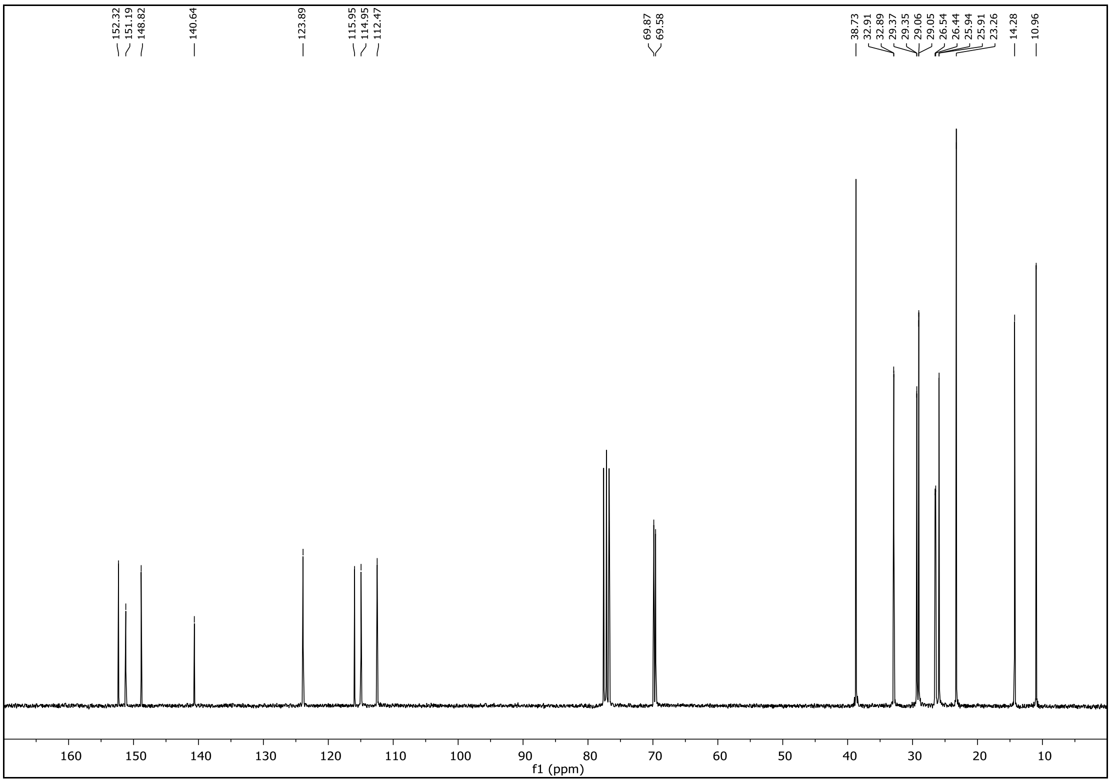


$^{1}$H- and $^{13}$C-NMR spectra of ***t*-57**

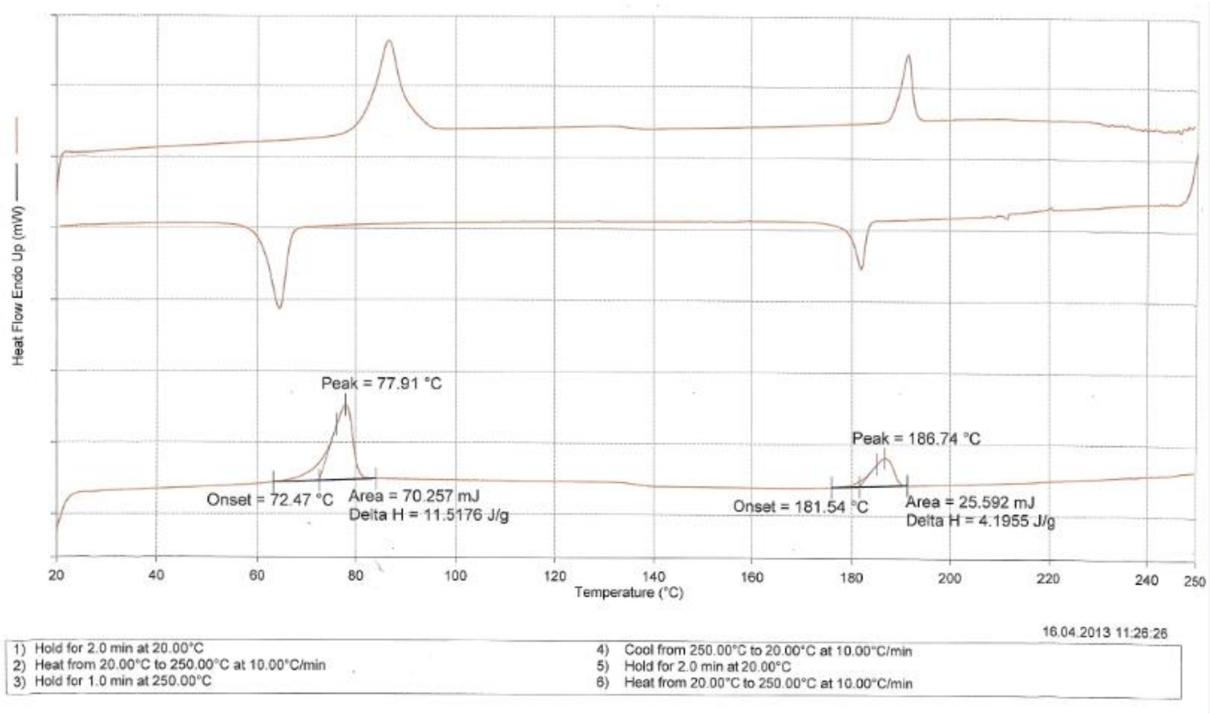


DSC of ***t*-57**

**3,4-Di((3,7-dimethyloctyl)oxy)benzonitrile**
According to the general procedure, 1.00 g (7.5 mmol) 3,4-dihydroxybenzonitrile, 3.07 g (22 mmol) potash, 3.60 g (16 mmol) 1-bromo-3,7-dimethyloctane in 50 mL DMF under nitrogen stirred at 80 °C for 16 h Additional 1.00 g potash and 1.2 g 1-bromo-4-ethyloctane and stirring for 12 h until reaction was finished (TLC). Acidulated with 2 N HCl, extraction with light petroleum (3*30 ml), brine and $MgSO_4$ gave, 3.28 g (7.4 mmol, 98 %) of a colorless oil.
**$^1$H-NMR** (400 MHz, $CDCl_3$): $\delta$ [ppm] = 7.23 (dd, $^3J$ = 8.3, $^4J$ = 1.9 Hz, 1H, 6-H, ph), 7.07 (d, $^4J$ = 1.9 Hz, 1H, 2-H, ph), 6.87 (d, $^3J$ = 8.3 Hz, 1H, 5-H, ph), 4.11 – 3.95 (m, 4H, $OCH_2$), 1.93 – 1.80 (m, 2H, $CH_2$), 1.72 – 1.46 (m, 2H, $CH_2$), 1.38 – 1.09 (m, 28H, $CH_2$), 0.98 – 0.96 (m, 6H, $CH_3$), 0.90 – 0.88 (m, 12H, $CH_3$). **$^{13}$C-NMR** (100 MHz, $CDCl_3$): $\delta$ [ppm] = 153.14, 149.16 (C-3, C-4), 126.39 (CH-ph), 119.57 (C-1), 115.95 (CH-ph), 112.70 (CH-ph), 103.60 (C-7, CN), 67.90, 67.60 ($OCH_2$), 39.34, 37.40, 36.06, 35.96, 30.03, 28.10, 24.82, 22.82, 22.73 ($CH_2$), 19.79 ($CH_3$). **IR** (ATR): $\tilde{\nu}$ [cm$^{-1}$] = 2925 m, 2867 m, 2224 m, 1598 m, 1513 s, 1469 m, 1421 w, 1383 w, 1334 w, 1266 s, 1135 s, 1004 m, 851 m, 807 m, 732 w. **HR-ESI:** calcd. for $C_{27}H_{45}NO_2 + Na^+$: 438.3348; found: 438.3334.

**5-(3,4-Di((3,7-dimethyloctyl)oxy)phenyl)-2*H*-tetrazole**
According to the general procedure, 2.00 g (4.80 mmol) 3,4-di(3,7-dimethyl octyloxy)benzonitrile, 1.11 g (17.00 mmol) $NaN_3$, 2.32 g (17 mmol) $NEt_3HCl$ in 50 mL toluene, 12 h reflux. Then additional 0.37 g (5.6 mmol) $NaN_3$ and 0.76 g (5.6 mmol) triethylammonium chloride, 10 h reflux. Chromatography on silica with toluene/ethyl acetate. Yield: 1.42 g (3.10 mmol, 65 %) of a colorless solid with m.p. = 94 - 95 °C (petroleum ether).
**$^1$H-NMR** (400 MHz, $CDCl_3$): $\delta$ [ppm] = 7.74 – 7.70 (m, 2H, H-2, H-6, ph ), 6.97 (d, $^3J$ = 8.4 Hz, 1H, H-5, ph ), 4.11 – 4.00 (m, 4H, $OCH_2$), 1.88 – 1.79 (m, 2H, $CH_2$), 1.65 – 1.42 (m, 6H, $CH_2$), 1.32 – 1.07 (m, 12H, $CH_2$), 0.93 – 0.81 (m, 18H, $CH_3$). **$^{13}$C-NMR** (100 MHz, $CDCl_3$): $\delta$ [ppm] = 156.33(C-5, tet), 152.22, 149.75 (C-3, C-4, ph), 120.94 (CH-ph), 115.45(C-1, ph), 113.26, 112.05 (CH-ph), 67.91, 67.73 ($OCH_2$), 39.36, 37.47, 36.19, 36.10, 30.08, 30.01, 28.10, 24.83 ($CH_2$), 22.82, 22.73, 19.78, 19.73 ($CH_3$). **IR** (ATR): $\tilde{\nu}$ [cm$^{-1}$] = 2925 m, 2871 m, 1609 m, 1513 s, 1468 m, 1384 m, 1267 s, 1226 s, 1134 s, 1042 m, 1005 m, 970 m, 868 m, 815 m, 752 m, 706 m. **HR-ESI:** calcd. for $C_{27}H_{46}N_4O_2 + Na^+$: 481.3518; found: 481.3531.

**3,7,11-Tris(3,4-di((3,7-dimethyloctyl)oxy)phenyl)tris([1,2,4]triazolo)[4,3-a:4',3'-c:4'',3''-e]-[1,3,5]triazine *t*-58**

According to the general procedure, 0.15 g (0.3 mmol) 5-(3,7-dimethyloctyl)phenyl)-2*H*-tetrazole and 0.1 mL *sym*-collidine in 30 mL xylenes were stirred for 30 min. Addition of 0.8 mL of a 0.11 M solution of cyanuric chloride in toluene to the stirred mixture. After 4 h, 0.1 mL of this solution was added and repeated every 2 h until the reaction was complete (TLC). Dilution with water, 50 mL 2N HCl and extraction with ethyl acetate (3 x 20 mL) gave the crude product that was absorbed on 0.5 g silica gel. Chromatography on a silica columns with head of basic alumina with toluene/ethyl acetate 30/1 gave 0.135 g (0.1 mmol, 90 %) of a colorless wax with m.p. = 51 °C (DSC, 2$^{nd}$ heating).
$^{1}$**H-NMR** (400 MHz, $CDCl_3$): $\delta$ [ppm] = 7.83 (dd, $^{3}J$ = 8.5, $^{4}J$ = 2.1 Hz, 3H, 6-H, ph), 7.68 (d, $^{4}J$ = 2.1 Hz, 3H, 2-H, ph), 7.04 (d, $^{3}J$ = 8.5 Hz, 3H, 5-H, ph), 4.18 – 4.09 (m, 12H, $OCH_2$), 1.93 – 1.90 (m, 6H, $CH_2$), 1.81 – 1.63 (m, 12H, $CH_2$), 1.57 – 1.48 (m, 6H, $CH_2$), 1.36 – 1.11 (m, 36H, $CH_2$), 0.97 – 0.93 (m, 18H, $CH_3$), 0.88 – 0.84 (m, 36H, $CH_3$). $^{13}$**C-NMR** (100 MHz, $CDCl_3$): 152.39 (C-4, ph), 151.23 (C-5, trl), 148.90 (C-3, ph), 140.65 (C-3, trl), 123.94 (C-6, ph), 115.93 (C-1, ph), 114.99 (C-5, ph), 112.45 (C-2, ph), 67.92, 67.60 ($OCH_2$), 39.40, 37.49, 36.27, 36.16, 30.13, 28.14, 24.87 ($CH_2$), 22.86, 22.76, 19.84 ($CH_3$). **IR** (ATR): $\tilde{\upsilon}$ [cm$^{-1}$] = 2925 s, 2871 m, 1596 m, 1523 w, 1491 m, 1466 s, 1383 w, 1329 w, 1258 vs, 1222 m, 1141 m, 1141 m, 1003 m, 866 w, 804 w, 714 w. **HR-ESI:** calcd. for $C_{84}H_{135}N_9O_6$ + $H^+$: 1367.0614; found: 1367.0588.

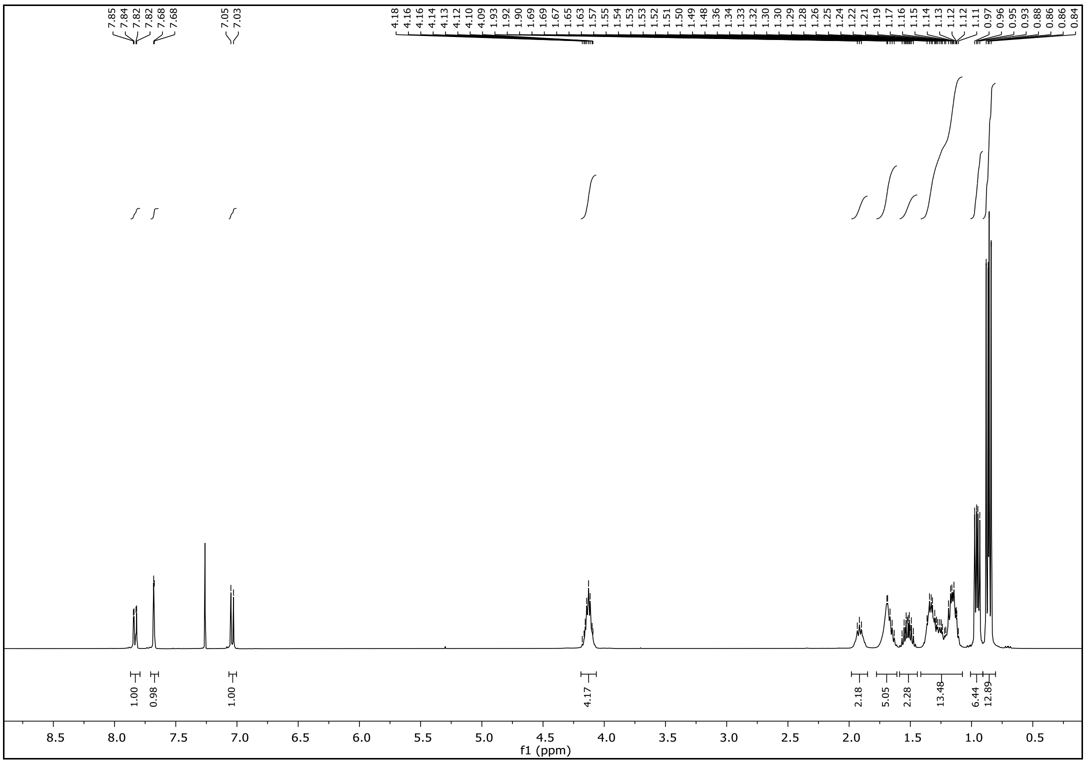

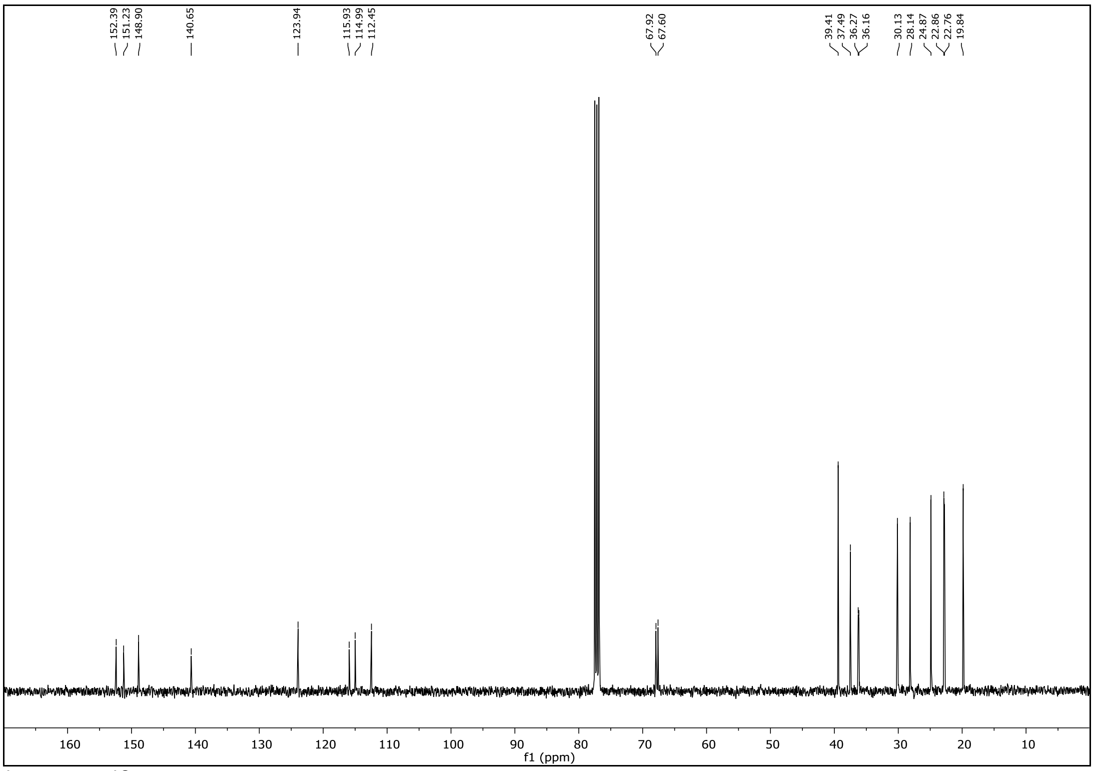


$^{1}$H- and $^{13}$C-NMR spectra of ***t*-58**

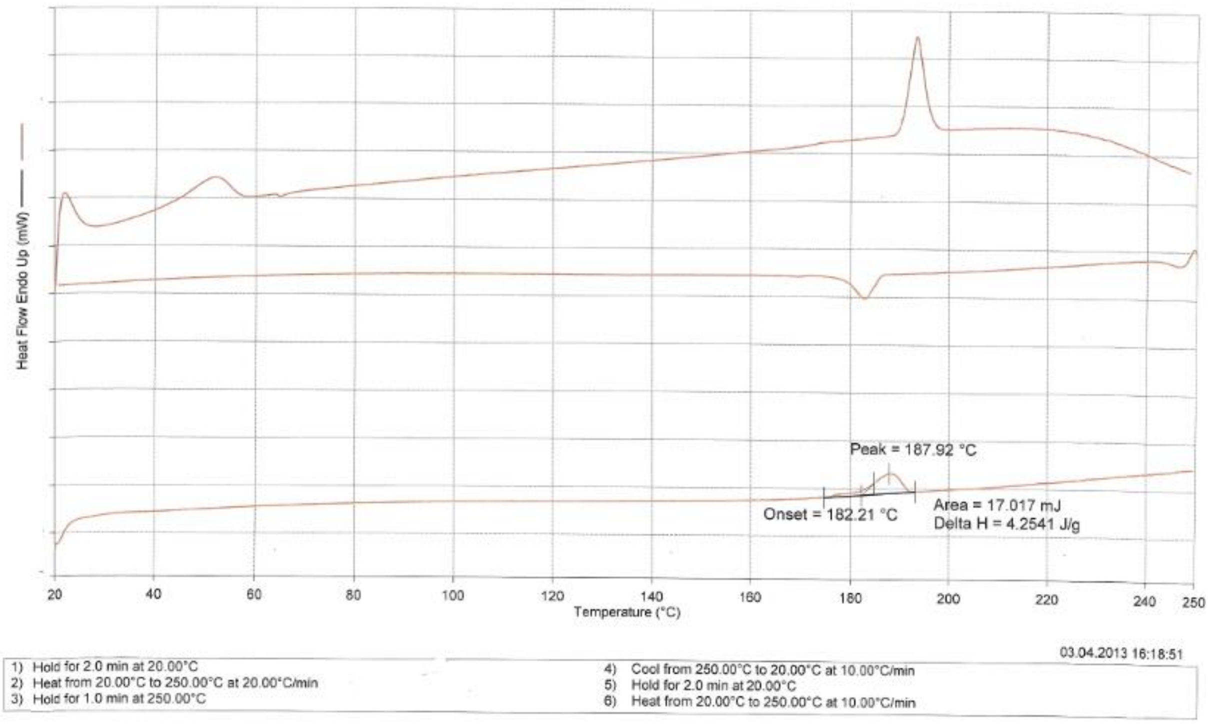


DSC of ***t*-58**

## 1-Bromo-3,3-dimethyloctane

7.12 g (45,64 mmol) 3,3-Dimethyloctan-1-ol, 10 mL hydrobromic acid (48 %) and 1,8 mL conc. sulfuric acid were mixed in an ice bath and heated to reflux für 2d. Dilution with water,

extraction with petroleum ether and chromatography ($SiO_2$, PE) gave 6.63 g (66 %) of a yellowish oil. $n_D^{26}$= 1,6783. **$^1$H-NMR** (300 MHz, $CDCl_3$)**:** δ (ppm): 3.44 – 3.35 (m, 2H, BrCH2), 1.91 – 1.78 (m, 2H, $BrCH_2CH_2$), 1.41 - 1.10 (m, 8H), 0.94 – 0.88 (m, 9H, $CH_3$). **IR:** 2965 (s), 2929 (s), 2860 (m), 1469 (m), 1366 (w), 1336 (w), 1236 (m), 911 (s), 735 (vs).

**3,4-Di(3,3-dimethyloctyloxy)benzonitrile**
0.51 g 3,4-Dihydroxybenzonitrile (3.78 mmol), 2.06 g 1-Bromo-3,3-dimethyloctane (9.4 mmol) and 1.4 g Kaliumcarbonat in 30 mL acetonitrile were stirred under reflux für 5d. Usual work-up and chromatography (SiO2, Tol:PE 1:1) yielded 770 mg (1.85 mmol, 50 %) of an off-white solid with m.p. = 57 °C.**$^1$H-NMR** (300 MHz, $CDCl_3$)**:** δ (ppm): 7.24 (dd, $^3J$ = 8.4 Hz,$^4J$= 1.9 Hz, 1H), 7.07 (d, $^4J$ = 1.9 Hz, 1H), 6.87 (d, $^3J$ = 8.4 Hz, 1H), 4.11 – 3.98 (m, 4H, $OCH_2$), 1.81 – 1.73 (m, 1H, $OCH_2CH_2$), 1.38 – 1.19 (m, 16H), 0.97 – 0.93 (m, 12H, $C(CH_3)_2$), 0.93 – 0.83 (m, 6H, $CH_3$). **$^{13}$C-NMR** (75 MHz, $CDCl_3$)**:** δ (ppm): 152.9, 149.0, 126.2, 119.5, 115.4, 112.3, 103.4, 66.5, 66.2, 42.5 (2C), 40.0, 39.9, 32.8 (2C), 32.2 (2C), 27.5, 27.4, 23.7 (2C), 22.7 (2C), 14.1 (2C). **IR:** 2955 (vs), 2929 (vs), 2870 (m), 2224 (m), 1597 (m), 1513 (vs), 1469 (m), 1267 (vs), 1135 (m), 1018 (m). **MS:** $C_{27}H_{45}NO_2 + H^+$: m/z calcd.: 416.3523 , found: 416.3523

**1*H*-5-(3,4-Di(3,3-dimethyloctyloxy)phenyl)tetrazole**
0.75 g 3,4-Di(3,3-dimethyloctyloxy)benzonitrile (1.80 mmo), 0.48 g $NaN_3$ (7.38 mmol) and 1.01 g Triethylammonium chloride (7.26 mmol) in 20 mL abs. toluene were refluxed for 5d. Work-up accordimg to the general procedure and chromatography ($SiO_2$, Tol →Tol:EE 2:1) yielded 685 mg (1.50 mmol, 83 %) of a colourless solid with m.p. = 56 °C. **$^1$H-NMR** (300 MHz, $CDCl_3$)**:** δ (ppm): 7.71 – 7.64 (m, 2H), 7.00 – 6.94 (m, 1H), 4.12 – 4.02 (m, 4H, $OCH_2$), 1.81 – 1.69 (m, 4H, $OCH_2CH_2$), 1.36 – 1.13 (m, 16H), 0.94 (s, 6H, $C(CH_3)_2$), 0.89 (s, 6H, $C(CH_3)_2$´), 0.85 (t, $_3J$ = 7.2 Hz, 6H. $CH_2CH_2$). **$^{13}$C-NMR** (75 MHz, $CDCl_3$)**:** δ (ppm): 156.3, 151.9, 149.6, 120.6, 115.5, 112.7, 111.5, 66.4, 66.2, 42.5 (2C), 40.2, 40.0, 32.8 (2C), 32.2, 32.1, 27.4 (2C), 27.3 (2C), 23.7 (2C), 22.7 (2C), 14.1 (2C). **IR:** 2954 (m), 2928 (s), 2870 (m), 1608 (m), 1506 (s), 1468 (m), 1387 (w), 1366 (w), 1322 (w), 1261 (s), 1230 (m), 1135 (m), 1062 (w), 1015 (m), 909 (s), 862 (w), 812 (w), 731 (vs). **MS:** $C_{27}H_{46}N_4O_2 + H^+$: m/z (calcd.: 459.3694, found: 459.3702.

**3,7,11-Tris(3,4-di(3,3-dimethyloctyloxy)phenyl)tris[1,2,4]-triazolo[4,3-a:4´,3´-c:4´´,3´´-e][1,3,5]triazine *t*-60**
According to the general procedure, 305 mg 1*H*-5-(3,4-Di(3,3-dimethyloctyloxy)phenyl)-tetrazole (0.67 mmol), 0.09 mL Collidin (0.67 mmol) in 20 mL abs. xylenes and cyanuric chloride (30 mg, 0.20 mmol) were stirred for 6 h at ambient temperatzure, for 15 h at 60 °C, 8 h at 90 °C and 15 h at 120 °C. Work-up and chromatography ($SiO_2$, 1 cm AlOx (basic), Tol:EE 40:1) gave 169 mg (0.12 mmol, 62 %) of a colourless solid. **$^1$H-NMR** (300 MHz, $CDCl_3$)**:** δ (ppm): 7.86 (dd, $^3J$ = 8.5 Hz, $^4J$ = 2.1 Hz, 3H, H6), 7.68 (d, $^4J$ = 2.1 Hz, 3H, H2), 7.04 (d, $^3J$ = 8.6 Hz, 3H, H5), 4.14 (m, 12H, $OCH_2$), 1.86 – 1.77 (m, 12H, $OCH_2CH_2$), 1.39 – 1.17 (m, 48H), 0.97 (s, 18H, $C(CH_3)_2$), 0.94 (s, 18H, $C(CH_3)_2$´), 0.93 – 0.83 (m, 18H, $CH_3$). **$^{13}$C-NMR** (75 MHz, $CDCl_3$)**:** δ (ppm): 152.1 (3C), 121.1 (3C), 148.7 (3C), 140.5 (3C), 123.7 (3C), 115.7 (3C), 114.4 (3C), 112.0 (3C), 66.3 (3C), 66.1 (3C), 42.5 (6C), 40.1 (3C), 40.0 (3c), 32.8 (6C), 32.3 (3C), 32.2 (3C), 27.5 (6C), 27.4 (6C), 23.7 (6C), 22.7 (6C), 14.1 (6C). **IR:** 3098 (vw), 2954 (s), 2928 (vs), 2864 (m), 1737 (w), 1578 (m), 1524 (w), 1468 (vs), 1437 (m), 1386 (m), 1366 (m), 1310 (m), 1256 (vs), 1224 (s), 1143 (m), 1102 (w), 1018 (m), 913 (vs), 812 (w), 745 (vs). **MS:** $C_{84}H_{135}N_9O6 + H^+$: m/z calcd.: 1367.0608, found: 1367.0634.

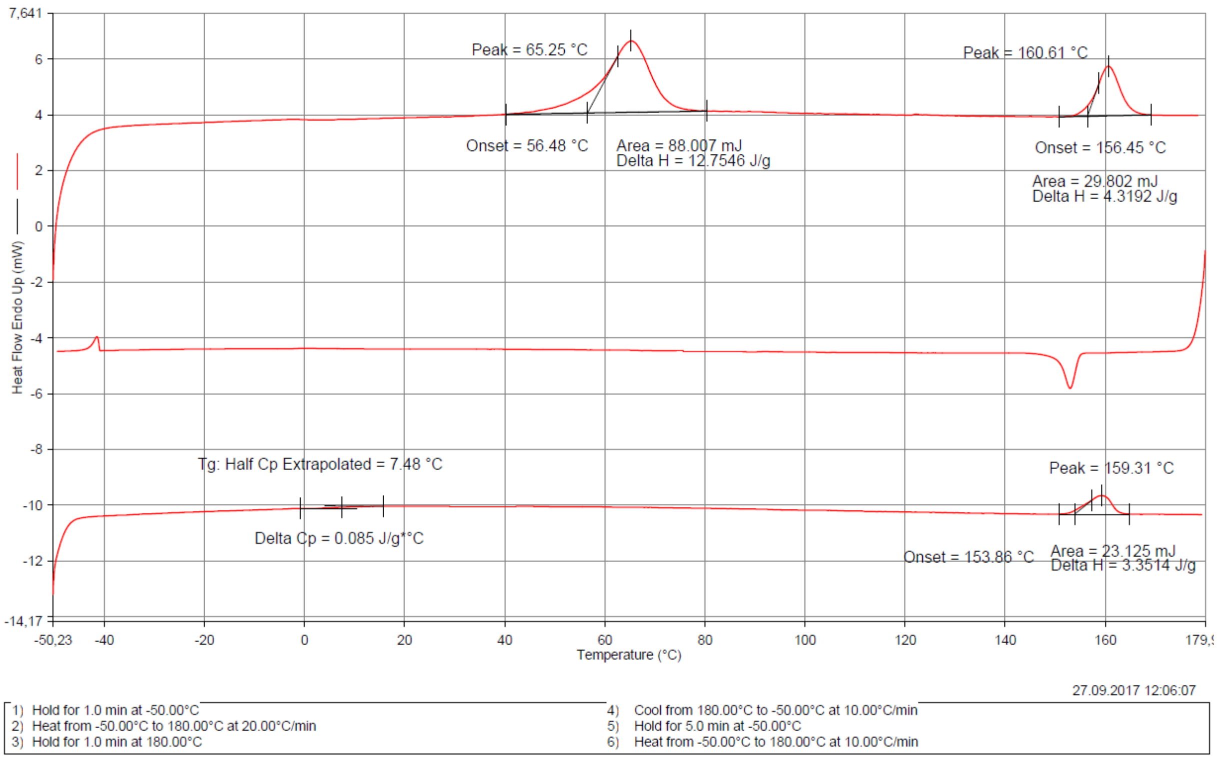


**DSC of *t*-60**

**Ethyl 3,4,5-tri((2-ethylhexyl)oxy)benzoate**
According to the general procedure, 2.40 g (12 mmol) ethyl 3,4,5-trihydroxybenzoate, 9.9 g (72 mmol) potassium carbonate and 8.8 g (80 mmol) 1-bromo-2-ethyloctane 100 mL dimethylformamide were stirred at 100 °C until the reaction was finished (TLC). Aqueous work-up yielded a yellow oil (6.0 g, 11 mmol, 92 %) that was used without further purification. **$^1$H-NMR** (400 MHz, $CDCl_3$): $\delta$ [ppm] = 7.25 (s, 2H, 2-H, 6-H, ph), 4.35 (q, $^3J$ = 7.1 Hz, 2H, 8-H, $OCH_2CH_3$), 3.91 – 3.88 (m, 6H, $OCH_2$), 1.78 – 1.66 (m, 3H, $CH_2$), 1.51 – 1.29 (m, 27H, $OCH_2CH_3$, $CH_2$), 0.95 – 0.87 (m, 18H, $CH_3$).

**3,4,5-Tri(2-ethylhexyloxy)benzoic acid**
60 mL Iso-propanol/water (2:1), 5.5 g (7.3 mmol) ethyl 3,4,5-tri(2-ethylhexyloxy)benzoate, 2.16 g (25 mmol) KOH 4 h reflux. Acidulation with 2 N HCl, extraction with ether yielded 4.9 g (9.6 mmol, 96 %) of a colorless solid that was used without further purification. **$^1$H-NMR** (400 MHz, $CDCl_3$): $\delta$ [ppm] = 7.32 (s, 2H, 2-H, 6-H, ph), 3.94 – 3.88 (m, 6H, $OCH_2$), 1.80 – 1.66 (m, 3H, $CH_2$), 1.50 – 1.28 (m, 24H, $CH_2$), 0.95 – 0.89 (m, 18H, $CH_3$).

**3,4,5-Tri(2-ethylhexyloxy)benzoic amide**
1.4 mL (19.7 mmol) Thionyl chloride was added to 2.0 g (3.9 mmol) 3,4,5-tri(2-ethylhexyl)oxy)benzoic acid in 50 mL toluene and the mixture was refluxed for 12 h. Concentration to ca. 7 mL and dropwise addition to heavily stirred cold ammonia (25%), extraction with chloroform and chromatography on silica gel with toluene/ethyl acetate/triethyl amine 40/10/1 yielded 1.8 g (3,6 mmol, 92 %) of a colorless solid with m.p. = 135 - 137 °C. **$^1$H-NMR** (400 MHz, $CDCl_3$): $\delta$ [ppm] = 7.01 (s, 2H, 2-H, 6-H, ph), 5.98 (s, breit, 2H, $NH_2$) 3.91 – 3.84 (m, 6H, $OCH_2$), 1.78 – 1.66 (m, 6H, $CH_2$), 1.56 – 1.27 (m, 33H, $CH_2$), 0.94 – 0.87 (m, 18H, $CH_3$). **$^{13}$C-NMR** (100 MHz, $CDCl_3$): $\delta$ [ppm] = 169.70 (C-7), 153.40 (C-3, C-5), 141.67 (C-4), 127.96 (C-1), 105.60 (C-2, C-6), 76.12, 71.56 ($OCH_2$), 40.74, 39.73, 30.62, 30.57, 29.23, 23.93, 23.79, 23.27, 23.22 ($CH_2$), 14.28, 14.24 ($CH_3$), 11.24 ($CH_3$). **IR** (ATR): $\tilde{\nu}$ [cm$^{-1}$] = 3387 w, 3198 w, 2922 m, 2861 m, 1643 m, 1577 m, 1425 m, 1377 m, 1315 w, 1237 m, 1040 w, 806 w, 695 w. **HR-ESI:** calcd. for $C_{31}H_{55}NO_4$ + $Na^+$: 528.4029; found: 528.4026.

**5-(3,4,5-Tri((2-ethylhexyl)oxy)phenyl)-2*H*-tetrazole**
According to the procedure, 1.70 g (3.4 mmol) 5-(3,4,5-tri(2-ethylhexyloxyphenyl)-benzamide, 1.44 g (22mmol) $NaN_3$, and 1.15 g (6.8 mmol) $SiCl_4$ in 30 mL acetonitrile were stirred for 11 h at ambiennt temperature. Additional reagents (0.66 mg (10 mmol) $NaN_3$, 0.74 g (3 mmol) $SiCl_4$) were added, stirring at 50 °C for 14 h. Aqueous work-up, extraction with chloroform and recrystallization from ethanol gave 1.46 mg (81 %) of a colorless solid with m.p. = 110 – 113 °C.
**$^1$H-NMR (400 MHz, $CDCl_3$):** $\delta$ [ppm] = 7.35 (s, 2H, 2-H, 6-H, ph), 3.93 – 3.83 (m, 6H, $OCH_2$) 1.73 – 1.67 (m, 3H, $CH_2$), 1.61 – 1.20 (m, 24H), 0.94 – 0.83 (m, 18H, $CH_3$). **$^{13}$C-NMR** (100 MHz, $CDCl_3$): $\delta$ [ppm] = 156.72 (C-7, tet) 154.24 (C-3, C-5, ph), 141.02 (C-4, ph), 117.71 (C-1, ph), 105.42 (C-2, C-6, ph), 76.30, 71.59 ($OCH_2$), 40.70, 39.67, 30.55, 29.41, 29.16, 23.84, 23.77, 23.26, 23.17 ($CH_2$), 14.26, 14.21 ($CH_3$), 11.30, 11.21 ($CH_3$). **IR** (ATR): $\tilde{\nu}$ [cm$^{-1}$] = 2956 m, 2926 m, 2872 m, 1587 m, 1495 m, 1452 s, 1379 m, 1306 m, 1243 m, 1120 s, 1055 w, 997 w, 837 w, 751 w, 712 w. **HR-ESI:** calcd. for $C_{31}H_{54}N_4O_3$ + $Na^+$: 553.4094; found: 553.4079.

**3,7,11-Tris(3,4,5-tri((2-ethylhexyl)oxy)phenyl)tris([1,2,4]triazolo)[4,3-a:4',3'-c:4'',3''-e]-[1,3,5]triazine *t*-61**
According to the general procedure, 514 mg (1 mmol) (3,4,5-tri(2-ethylhexyloxyphenyl)-*2H*-tetrazole and collidine (0.1 mL) were stirred in xylenes (30 mL) for 15 min. 54 mg (0.3 mmol) Cyanuric chloride was added and the stirred mixture gradually heated to 80 °C. After 16 h, aqueous work-up and chromatography on a silica column with head of basic alumina with toluene followed by toluene / ethyl acetate 30/1 yielded 378 mg (71 %) of a colorless solid with m.p. = 159 - 161 °C (POM). **$^1$H-NMR** (400 MHz, $CDCl_3$): $\delta$ [ppm] = 7.42 (s, 2H, 2-H, 6-H, ph), 3.99 – 3.92 (m, 6H, $OCH_2$), 1.82 – 1.70 (m, 3H, $CH_2$), 1.62 – 1.26 (m, 24H), 0.98 – 0.87 (m, 18H, $CH_3$). **$^{13}$C-NMR** (100 MHz, $CDCl_3$): $\delta$ [ppm] = 153.36 (C-3, C-5, ph), 151.41 (C-5, trl), 141.44, 140.72 (C-3, trl; C-4, ph), 118.25 (C-1), 108.25 (C-2, C-6), 76.23, 71.64 ($OCH_2$), 40.77, 39.72, 30.63, 30.61, 29.46, 29.25, 23.94, 23.83, 23.32, 23.26 ($CH_2$), 14.32, 14.27 ($CH_3$), 11.36, 11.29 ($CH_3$). **IR** (ATR): $\tilde{\nu}$ [cm$^{-1}$] = 2956 m, 2924 m, 2858 m, 1577 s, 1486 m, 1460 m, 1429 m, 1379 w, 1335 m, 1220 w, 1110 s, 999 w, 841 w, 715 m. **HR-ESI:** calcd. for $C_{96}H_{159}N_9O_9$ + $H^+$: 1583.2339; found: 1583.2318.

**2,6,10-Tris(3,4,5-tri((2-ethylhexyl)oxy)phenyl)tris([1,2,4]triazolo)[1,5-a:1',5'-c:1'',5''-e]-[1,3,5]triazine *r*-61**
215 mg 3,7,11-tris(3,4,5-tri(2-ethylhexyloxy)phenyl)tris([1,2,4]triazolo)[4,3-a:4',3'-c:4'',3''-e][1,3,5]triazine in 2.0 mL octadecane was stirred for 22 h at 275 °C. The cooled mixture was diluted with light petroleum, filtered through silica and after octadecane had been eluted with petroleum ether, the product was isolated with toluene. Chromatography on silica with petroleum ether/toluene 3/1 gave 60 mg (28 %) of a colorless solid with m.p. = 242 - 244 °C (POM). **$^1$H-NMR** (400 MHz, $CDCl_3$): $\delta$ [ppm] = 7.64 (s, 2H, 2-H, 6-H, ph), 4.06 – 3.92 (m, 6H, $OCH_2$), 1.84 – 1.72 (m, 3H, $CH_2$), 1.65 – 1.30 (m, 24H), 0.99 – 0.91 (m, 18H, $CH_3$). **$^{13}$C-NMR** (100 MHz, $CDCl_3$): $\delta$ [ppm] = 165.72 (C-5, trl), 153.87 (C-3, C-5, ph), 144.63 (C-3, trl), 141.23 (C-4, ph), 122.71 (C-1), 105.78 (C-2, C-6), 76.17, 71.64 ($OCH_2$), 40.80, 39.85, 30.67, 30.64, 29.48, 29.28, 24.02, 23.86, 23.29 ($CH_2$), 14.32, 14.29, ($CH_3$), 11.39, 11. 28 ($CH_3$). **IR** (ATR): $\tilde{\nu}$ [cm$^{-1}$] = 2924 m, 2861 m, 1625 s, 1584 w, 1457 s, 1433 s, 1382 m, 1331 s, 1227 m, 1117 s, 984 w, 851 m, 747 m. **HR-ESI:** calcd. for $C_{96}H_{159}N_9O_9$ + $H^+$: 1583.2339; found: 1583.2306.

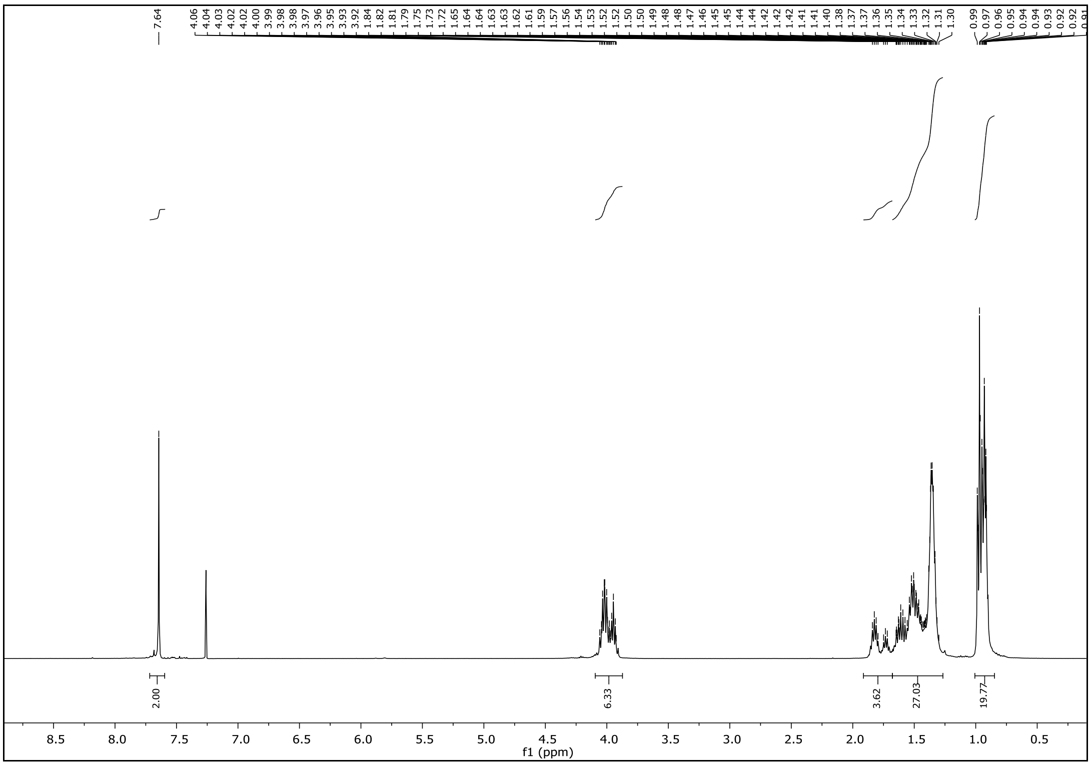


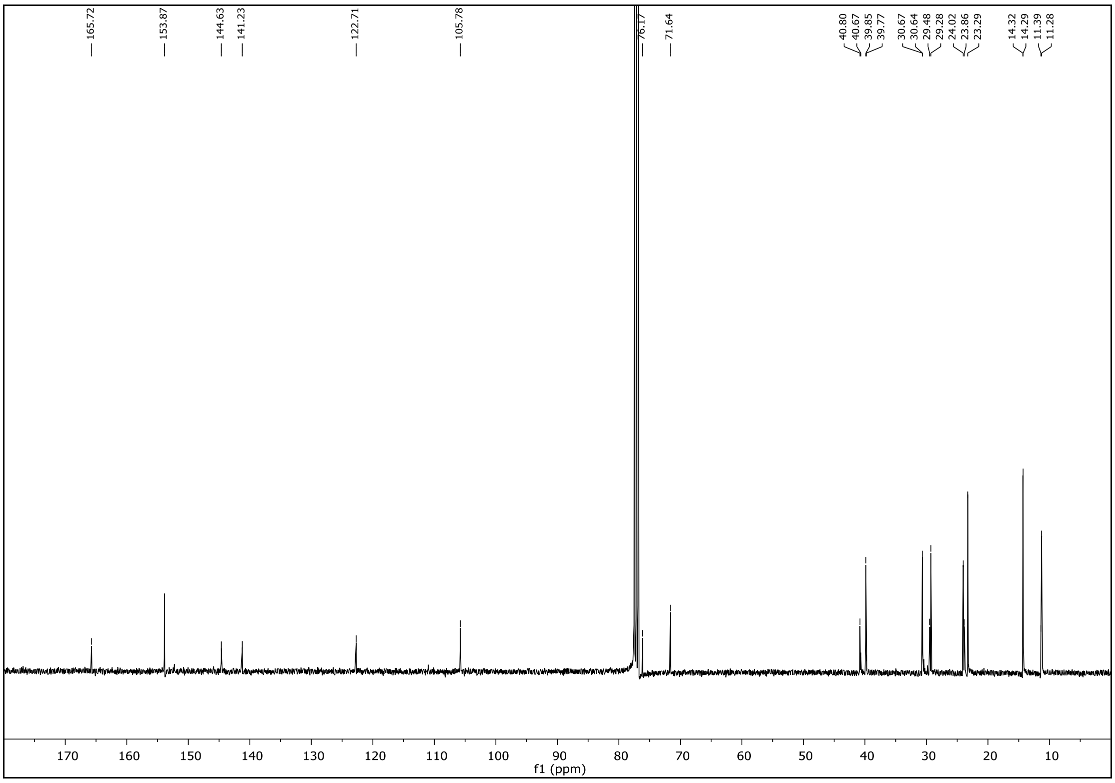


$^1$H- and $^{13}$C-NMR spectra of ***r*-61**

**Ethyl 3,4,5-tri((4-ethyloctyl)oxy)benzoate[1]**

According to the general procedure GP3, 2.00 g (10 mmol) ethyl 3,4,5-trihydroxybenzoate, 5.58 g (40 mmol) potassium carbonate and 7.48 g (33 mmol) 1-bromo-4-ethyloctane 80 mL dimethylformamide were stirred at 100 °C; after 16 h, additional 1.12 g (5.1 mmol) 1-bromo-

4-ethyloctane and 0.70 g (5.5 mmol) potassium carbonate were added, stirring was continued for 24 h. Aqueous work-up yielded a yellow oil (4.8 g, 7.8 mmol, 77 %) that was used without further purification. **$^1$H-NMR** (400 MHz, $CDCl_3$): $\delta$ [ppm] = 7.25 (s, 2H, 2-H, 6-H, ph), 4.35 (q, $^3J$ = 7.1 Hz, 2H, 8-H, $OCH_2CH_3$), 4.03 – 3.99 (m, 6H, $OCH_2$), 1.83 – 1.69 (m, 6H, $CH_2$), 1.48 – 1.14 (m, 36H, $OCH_2CH_3$, $CH_2$), 0.91 – 0.82 (m, 18H, $CH_3$). **IR** (ATR): $\tilde{\upsilon}$ [cm$^{-1}$] = 2957 m, 2924 m, 2855 m, 1715 m, 1590 w, 1499 w, 1459 w, 1425 m, 1379 w, 1370 w, 1334 m, 1216 s, 1112 s, 1032 m, 861 w, 764 m.

**3,4,5-Tri((4-ethyloctyl)oxy)benzoic acid[1]**

45 mL Iso-propanol/water (2:1), 4.5 g (7.3 mmol) ethyl 3,4,5-tri((4-ethyloctyl)oxy)benzoate, 1.43 g (25 mmol) KOH 1 h reflux. Evaporation of i-propanol, acidulation with 2 N HCl, extraction with ether yielded 4.3 g (7.27 mmol, 99 %) of a colorless solid that was used without further purification. **$^1$H-NMR** (400 MHz, $CDCl_3$): $\delta$ [ppm] = 7.32 (s, 2H, 2-H, 6-H, ph), 4.06 – 4.00 (m, 6H, $OCH_2$), 1.84 – 1.70 (m, 6H, $CH_2$), 1.46 – 1.25 (m, 33H, $CH_2$), 0.91 – 0.83 (m, 18H, $CH_3$). **IR** (ATR): $\tilde{\upsilon}$ [cm$^{-1}$] = 2924 m, 2858 m, 1685 m, 1585 m, 1502 w, 1430 m, 1379 w, 1327 m, 1227 m, 1113 s, 1006 w, 864 w, 768 w, 723 w.

**3,4,5-Tri((4-ethyloctyl)oxy)benzoic amide[1]**

2.6 mL (37,0 mmol) thionyl chloride was added to 4.3 g (7.3 mmol) 3,4,5-tri((4-ethyloctyl)oxy)benzoic acid in 50 mL toluene and the mixture was refluxed for 12 h. Concentration to ca. 7 mL and dropwise addition to heavily stirred cold ammonia (25%), extraction with chloroform and recrystallization from ethanol yielded 4.25 g of a colorless solid with m.p. = 78 - 80 °C. **$^1$H-NMR** (400 MHz, $CDCl_3$): $\delta$ [ppm] = 7.01 (s, 2H, 2-H, 6-H, ph), 5.85 (d, broad, 2H, $NH_2$) 4.01 – 3.98 (m, 6H, $OCH_2$), 1.82 – 1.69 (m, 6H, $CH_2$), 1.46 – 1.16 (m, 33H, $CH_2$), 0.91 – 0.83 (m, 18H, $CH_3$). **$^{13}$C-NMR** (100 MHz, $CDCl_3$): $\delta$ [ppm] = 169.39 (C-7), 153.18 (C-3, C-5), 141.76 (C-4), 128.23 (C-1), 106.28 (C-2, C-6), 74.18, 69.99 ($OCH_2$), 38.90, 38.73, 32.91, 29.58, 29.52, 29.07, 27.72, 26.72, 25.93, 23.28 ($CH_2$), 14.30 ($CH_3$), 10.97 ($CH_3$). **IR** (ATR): $\tilde{\upsilon}$ [cm$^{-1}$] = 3378 w, 3185 w, 2924 m, 2857 m, 1645 m, 1577 m, 1505 w, 1461 m, 1428 m, 1377 m, 1318 w, 1231 m, 1115 s, 1006 w, 785 w, 697 w. **HR-ESI:** calcd. for $C_{37}H_{67}NO_4$ + $Na^+$: 612.4968; found: 612.4979.

**5-(3,4,5-Tri((4-ethyloctyl)oxy)phenyl)-2*H*-tetrazole[1]**

According to procedure GP6, 2.00 g (3.4 mmol) 5-(3,4,5-tri(4-ethyloctyloxyphenyl)-benzamide, 1.32 g (20mmol) $NaN_3$, and 1.15 g (6.8 mmol) $SiCl_4$ in 30 mL acetonitrile were stirred for 16 h at ambient temperature. Additional reagents (0.66 mg (10 mmol) $NaN_3$, 0.74 g (3 mmol) $SiCl_4$) were added, stirring at 50 °C for 1 h. Aqueous work-up and chromatography on silica, first with toluene/ethyl acetate/triethyl amine (40/10/1) followed by toluene ethyl acetate (2/1) gave 1.3 mg (63 %) of a colorless solid with m.p. = 70 – 73 °C. **$^1$H-NMR** (400 MHz, $CDCl_3$): $\delta$ [ppm] = 7.25 (s, 2H, 2-H, 6-H, ph), 4.06 (t, 2H, $OCH_2$), 3.97 (t, 4H, $OCH_2$) 1.80 – 1.73 (m, 6H, $CH_2$), 1.44 – 1.22 (m, 33H), 0.91 – 0.83 (m, 18H, $CH_3$). **$^{13}$C-NMR** (100 MHz, $CDCl_3$): $\delta$ [ppm] = 153.86 (C-3, C-5, Ph), 140.55 (C-4), 118.80 (C-1), 105.66 (C-2, C-6), 74.49, 69.91 ($OCH_2$), 38.88, 38.71, 32.91, 32.87, 29.55, 29.50, 29.08, 29.05, 27.68, 26.64, 25.90, 25.87, 23.27 ($CH_2$), 14.30 ($CH_3$), 10.94 ($CH_3$). **IR** (ATR): $\tilde{\upsilon}$ [cm$^{-1}$] = 2958, 2859, 1589, 1507, 1496, 1457, 1378, 1240, 1116, 913, 775. **HR-ESI**: calcd. for $C_{37}H_{66}N_4O_3$ + $H^+$: 615.5213; found: 615.5207.

**3,7,11-Tris(3,4,5-tri((4-ethyloctyl)oxy)phenyl)tris([1,2,4]triazolo)[4,3-a:4',3'-c:4'',3''-e]-[1,3,5]triazine *t*-62**

According to the general procedure, 300 mg (0.5 mmol) (3,4,5-tri(4-ethyloctyloxyphenyl)-2*H*-tetrazole and collidine (0.1 mL) were stirred in xylenes (25 mL) for 15 min. Two ml of a solution of 40 mg cyanuric chloride in 4 mL xylenes was added and the stirred mixture gradually heated to 80 °C. After 6 h, 0.8 mL of the cyanuric chloride solution were added, after 12 h 0.2 mL of this solution. Aqueous work-up after 4 h, chromatography on a silica column with head of basic alumina with toluene / ethyl acetate 30/1 yielded 244 mg (79 %) of

a colorless solid with m.p. = 124 C (DSC, 2nd heating). **$^{1}$H-NMR** (400 MHz, $CDCl_3$): $\delta$ [ppm] = 7.44 (s, 2H, 2-H, 6-H, ph), 4.09 – 4.05 (m, 6H, $OCH_2$), 1.86 – 1.76 (m, 6H, $CH_2$), 1.46 – 1.25 (m, 33H), 0.91 – 0.84 (m, 18H, $CH_3$). **$^{13}$C-NMR** (100 MHz, $CDCl_3$): $\delta$ [ppm] = 153.15 (C-3, C-5, ph), 151.39 (C-5, trl), 141.46, 140.73 (C-3, trl; C-4, ph), 118.29 (C-1), 108.88 (C-2, C-6), 74.32, 69.92 ($OCH_2$), 38.92, 38.78, 32.96, 32.92, 29.59, 29.12, 29.07, 27.80, 26.75, 25.95, 23.32, 23.28 ($CH_2$), 14.30 ($CH_3$), 10.99 ($CH_3$). **IR** (ATR): $\tilde{\nu}$ [$cm^{-1}$] = 2924 m, 2858 m, 1577 m, 1486 m, 1463 m, 1430 m, 1378 w, 1337 m, 1282 w, 1227 w, 1112 s, 1004 w, 867 w, 802 w, 707 w, 675 w. **HR-ESI**: calcd. for $C_{114}H_{195}N_9O_9 + H^+$: 1835.5156; found: 1835.5103.

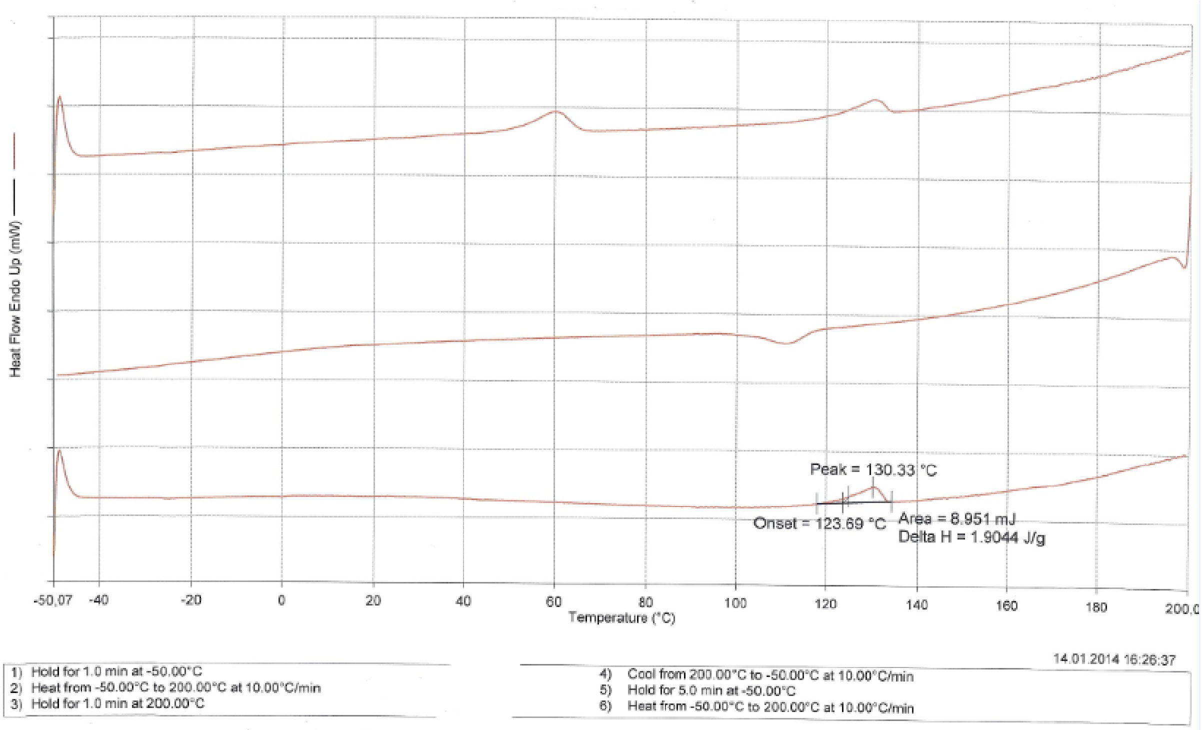


DSC of ***t*-62**

**2,6,10-Tris(3,4,5-tri((4-ethyloctyl)oxy)phenyl)tris([1,2,4]triazolo)[1,5-a:1',5'-c:1'',5''-e]-[1,3,5]triazine *r*-62**

120 mg 3,7,11-tris(3,4,5-tri(4-ethyloctyloxy)phenyl)tris([1,2,4]triazolo)[4,3-a:4',3'-c:4'',3''-e][1,3,5]triazine in 2.0 mL octadecane was stirred for 56 h at 275 °C. The cooled mixture was diluted with light petroleum, filtered through silica and after octadecane had been eluted with petroleum ether, the product was isolated with toluene. Chromatography on silica with petroleum ether/ethyl acetate 30/1 gave 39 mg (32 %) of a colorless solid with m.p. = 75 °C (DSC, 2. heating). **$^{1}$H-NMR** (400 MHz, $CDCl_3$): $\delta$ [ppm] = 7.64 (s, 2H, 2-H, 6-H, ph), 4.13 (t, $^{3}J$ = 6.6 Hz, 4H, $OCH_2$), 4.06 (t, $^{3}J$ = 6.6 Hz, 2H, $OCH_2$), 1.93 – 1.67 (m, 6H, $CH_2$), 1.53 – 1.17 (m, 33H, $CH_2$), 0.92 – 0.84 (m, 18H, $CH_3$). **$^{13}$C-NMR** (100 MHz, $CDCl_3$): $\delta$ [ppm] = 165.56 (C-5, trl), 153.64 (C-3, C-5, ph), 144.63 (C-3, trl), 141.40 (C-4, ph), 122.87 (C-1, ph), 106.44 (C-2, ph), 74.27, 70.02 ($OCH_2$), 38.94, 38.74, 32.97, 32.93, 29.63, 29.56, 29.10, 27.81, 26.78, 25.97, 23.32 ($CH_2$), 14.34, 11.00 ($CH_3$). **IR (ATR):** $\tilde{\nu}$ [$cm^{-1}$] = 2924 m, 2858 m, 1627 s, 1587 w, 1464 s, 1435 s, 1379 m, 1333 s, 1228 m, 1115 s, 989 w, 866 w, 848 w, 747 m. **HR-ESI:** calcd. for $C_{114}H_{195}N_9O_9 + H^+$: 1835.5156; found: 1835.5194.

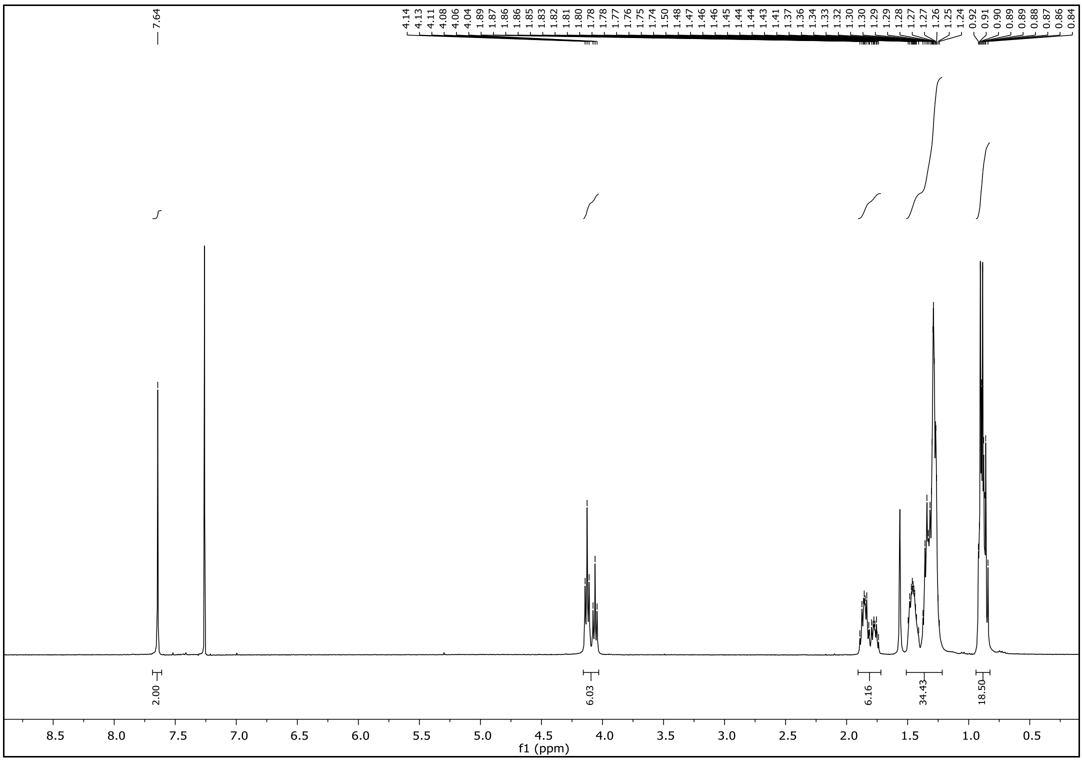


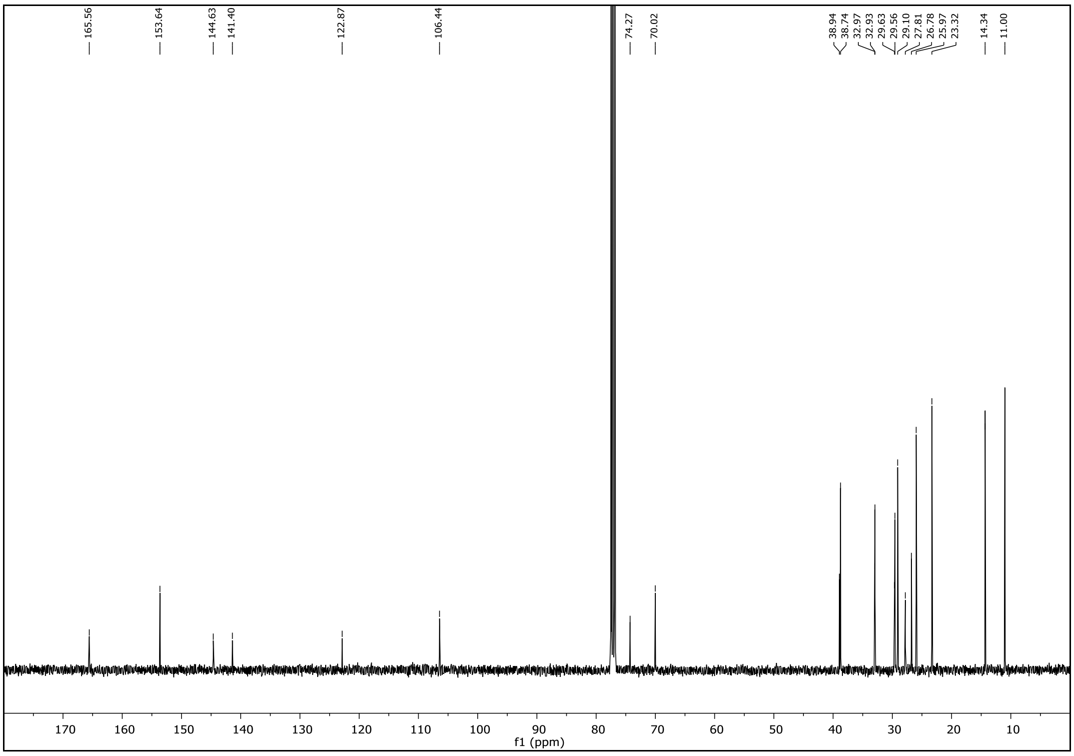


$^{1}$H- and $^{13}$C-NMR spectra of ***r*-62**

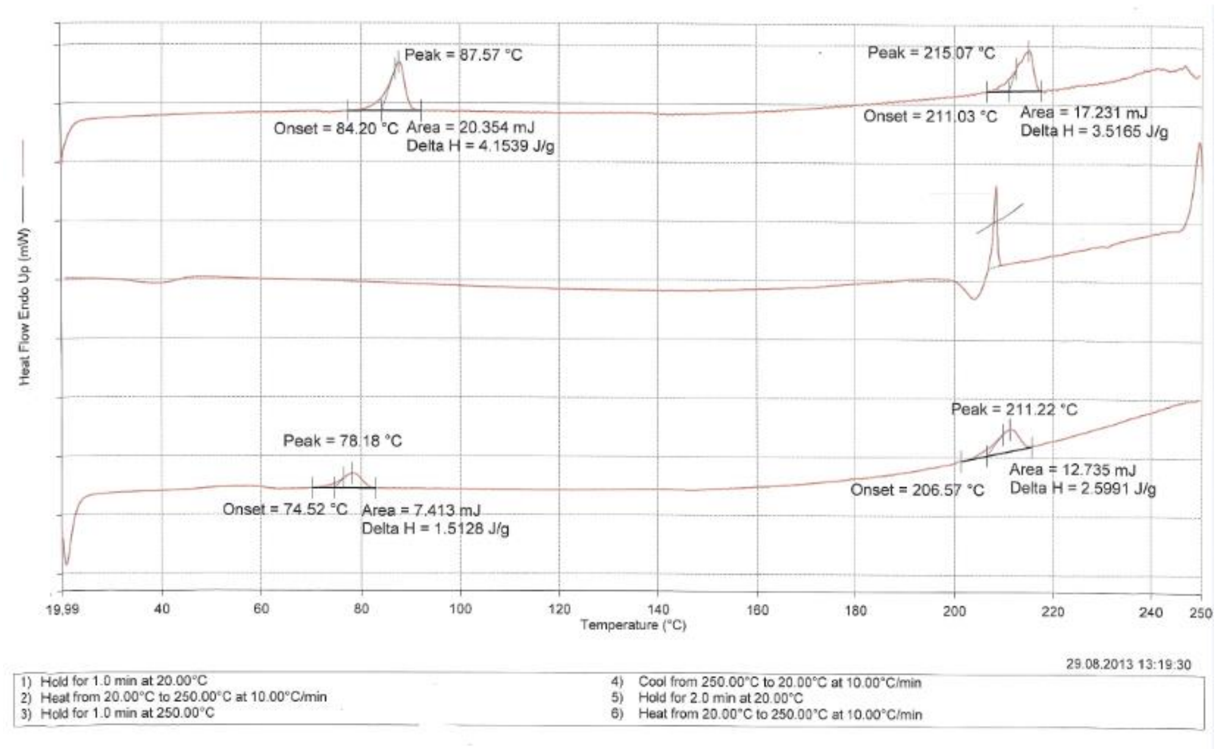


DSC of ***r*-62**

**Ethyl 3-(2-octyldecyloxy)benzoate**

According to the general procedure, 4.54 g (13.6 mmol) 2-octyldecylbromide, 1.88 g (13.6 mmol) $K_2CO_3$, 2.05 g (12.3 mmol) ethyl 3-hydroxybenzoate and 1 drop Aliquat 336 in 60 mL acetonitrile gave, after 9 h reflux, avaporation of the solvent, acidulation with HCl and extraction with chloroform, and chromatography ($SiO_2$; PE) 4.51 g (10.8 mmol, 88 %) of a brownisch oil with $n_D^{20}$ = 1.4855.

**IR**: 2955 m, 2923 ss, 2853 s, 1721 ss, 1466 m, 1442 m, 1274 ss, 1222 s, 1100 s, 1030 s, 754 ss. **$^1$H-NMR** (300 MHz, $CDCl_3$): δ/ppm = 7.62 (dt, $^3J$ = 7.7 Hz, $^4J$ = 1.3 Hz, 2H), 7.56 (dd, $^4J$ = 2.7 Hz; 1.5 Hz, 1H), 7.33 (t, $^3J$ = 7.9 Hz), 7.09 (ddd, $^3J$ = 8.2 Hz, $^4J$ = 2.7 Hz, $^5J$ = 1.0 Hz), 4.38 (q, $^3J$ = 7.1 Hz, 3H), 3.87(d, $^3J$ = 5.6 Hz, 2H), 1.82-1.75 (m, 1H), 1.49-1.26 (m, 28H), 1.40 (t, 3J = 7.1 Hz), 0.88 (t, 3J = 6.8 Hz). **$^{13}$C-NMR** (75.5 MHz, $CDCl_3$): δ/ppm = 166.7, 159.5, 131.8, 129.4, 121.8, 119.8, 114.9, 71.2, 61.1, 38.1, 32.1, 31.5, 30.2, 29.7, 29.5, 27.0, 22.8, 14.5, 14.3. **MS** (HR-ESI): m/z = 419.3530 ([M+H]$^+$, calcd.: 419.3520), 441.2996 ([M+Na]$^+$, found.: 441.3339).

**3-(2-Octyldecyloxy)benzoic acid**

Following the general procedure, 3.51 g (8.4 mmol) 3-(2-octyldecyloxy)benzoic acid in 20 mL ethanol with 2.18 g (54.5 mmol) NaOH in 28 mL water was refluxed for 15 min, concentrated and acidulated. After 3 d at pH = 2 and -18 °C, the precipitate was filtered, washed and dried in vacuo over $CaCl_2$ to yield 3.15 g (8.1 mmol, 96 %) of an off-white solid with m.p. = 46 °C.

**IR**: 2918 s, 2851 s, 1685 ss, 1605 w, 1588 m, 1451 ss, 1417 w, 1291 ss, 1240 ss, 1117 w, 1033 s, 930 m, 769 m, 757 ss, 721 w, 682 m, 666 w. **$^1$H-NMR** (300 MHz, $CDCl_3$): δ/ppm = 7.67 (dt, $^3J$ = 7.7 Hz, $^4J$ = 1.2 Hz, 1H, 6-H), 7.59-7.58 (m, 1H, 4-H), 7.35 (t, $^3J$ = 7.9 Hz, 5-H), 7.13 (ddd, $^3J$ = 8.2 Hz, $^4J$ = 2.6 Hz, $^5J$ = 1.0 Hz, 2-H), 3.87 (d, $^3J$ = 5.6 Hz, 2H, $OCH_2$), 1.79-

1.76 (m, 1H, CH), 1.44-1.25 (m, 28H, $CH_2$), 0.88 (t, $^3J$ = 6.7 Hz, 6H, $CH_3$). **$^{13}$C-NMR** (75.5 MHz, $CDCl_3$): δ/ppm = 172.1, 159.5, 131.2, 129.5, 122.4, 120.8, 115.2, 71.2, 38.1, 32.1, 31.5, 30.2, 29.7, 29.5, 27.0, 22.8, 14.3. **MS** (HR-ESI): m/z = 391.3206 ([M+H]+, found: 391.3207).

**3-(Octyldecyloxy)benzoic acid amide**

This compound wass prepared following the general procedure from 3.00 g (7.7 mmol) 3-(2-octyldecyloxy)benzoeic acid in 5 mL (8.20 g, 68.9 mmol) thionyl chloride. Refluxing for 16 h, removal of excess reagent, dilution with 50 mL toluene and dropwisde addition n ice-cold and heaviliy stirred concentrated ammonia. Filtration gave 0.83 g, 2.07 g were obtained after extraction with chloroform, the combined material was recrystalklized from ethanol to yield 2.33 g (6.0 mmol, 78 %) of an off-white powder with m.p. = 75 °C.

**IR**: 3367 m, 3168 m, 2920 ss, 2850 s, 1650 ss, 1622 m, 1603 m, 1583 s, 1446 ss, 1392 s, 1327 m, 1249 ss, 1139 m, 1032 m, 869 m, 795 m, 749 m, 721 m, 684 s. **$^1$H-NMR** (300 MHz, $CDCl_3$): δ/ppm = 7.39-7.28 (m, 3H) , 7.08-7.04 (m, 1H), 3.87 (d, $^3J$ = 5.7 Hz, 2H), 1.80-1.72 (m, 1H), 1.48-1.25 (m, 28H), 0.86 (t, $^3J$ = 6.7 Hz). **$^{13}$C-NMR** (75.5 MHz, $CDCl_3$): δ/ppm = 169.5.1, 159.8, 134.8, 129.7, 119.0, 118.8, 113.3, 71.2 , 38.1, 32.1, 31.5, 30.2, 29.7, 29.5, 27.0, 22.8, 14.3. **MS** (HR-ESI): m/z = 412.3210 ([M+Na]+, calcd.: 412:3186).

**5-(3-(2-Octyldecyloxy)phenyl)-1*H*-tetrazole**

A mixture of 3.17 g (48.8 mmol) $NaN_3$ in 60 mL dry acetonitrile, 2 mL (3.0 g, 17.7 mmol) tetrachlorsilane was stirred under nitrogen for 1 h, 2.13 g (5.5 mmol) 3-(2-octyldecyloxy)-benzoic acid amide Was added and the mixture stirred for 26 h at 20 °C. A yellow solid was formed. Addition of 150 mL water, filtration, dissolution of the filter cake in ethanol, filtration and evaporation followed by chromatography on silica gel with Tol:$Et_3N$ = 1:5 %v/v → Tol:AcOH = 1:5 %v/v gave, after evaporation of solvent, a solid that was dissolved in chloroform, extracted with hydrochloric acid (2N), wahed with brine, dired over magnesium sulfate and concentrated wto yield 1.14 g (2.7 mmol, 49 %) of a brownisch solid with m.p. = 72 °C. **IR**: 2952 m, 2922 ss, 2853 s, 2759 w, 2624 w, 1589 s, 1559 s, 1490 s, 1456 s, 1287 w, 1246 ss, 1038 m, 876 m, 793 m, 758 ss, 746 ss, 685 m. **$^1$H-NMR** (300 MHz, $CDCl_3$): δ/ppm = 7.69-7.67 m, 7.41 (t, $^3J$ = 8.2 Hz, 1H), 7.09-7.05 (m, 1H), 3.87 (d, $^3J$ = 5.6 Hz, 2H), 1.80-172 (m, 1H, CH), 1.46-1.24 (m, 28H, $CH_2$), 0.86 (t, $^3J$ = 6.7 Hz, 6H, $CH_3$). **$^{13}$C-NMR** (75.5 MHz, $CDCl_3$): δ/ppm = 160,3, 156.9, 130.6, 124.7, 119.5, 119.0, 112.8, 71.4, 38.1, 32.0, 31.4, 30.2, 29.7 , 29.7, 29.5, 27.0, 22.8, 14.3. **MS** (HR-ESI): m/z = 415.3434 ([M+H]+, ber.: 415.3431), 437.3191 ([M+Na]+, calcd: 437.3251).

**3,7,11-Tris(3-(2-octyldecyloxy)phenyl)tris[1,2,4]triazolo[4,3-a:4´,3´-c:4´´,3´´e] [1,3,5]-triazine**

0.5018 g (1.21 mmol) 5-(3-(2-Octyldecyloxy)phenyl)-1H-tetrazole, 0.16 mL (146 mg, 1.21 mmol) 2,4,6-collidine in 50 mL dry xylenes was stirred for 30 min at 20 °C. After addition of 68.2 mg (0.37 mmol) cyanuric chloride gegeben, the mixtiure was stirred for 3 h, heated to 90 °C for 17 h and 4 h at 120 °C. The mixture was acidulated with 50 mL hydrochloric acid and the aqueous layer was extracted with chloroform. The combined organic layers were dried, concentrated purified by column chromatography (2 cm basic $Al_2O_3$ + 20 cm $SiO_2$; toluene) to yield a yellowish, highly viscous oil (368 mg, 81%). IR: 2954 m, 2922 ss, 2853 s, 1578 ss,1464 s, 1322 m, 1239 s, 1030 m, 874 w, 788 m, 716 s, 686 s. 1H-NMR (300 MHz, CDCl3): δ/ppm = 7.74-7.71 (m, 3H, 4-H), 7.64-7.63 (m, 3H, 2-H), 7.51-7.46 (m, 1H, 5-H), 7.19-7.15 (m, 3H, 6-H), 3.92 (d, $^3J$ = 5.7 Hz, 6H, O-CH2), 1.84-1.78 (m, 3H, CH), 1.56-1.20 (m, 86H, $CH_2$), 0.87 (t, $^3J$ = 6.7 Hz, 18H, $CH_3$). $^{13}$C-NMR (75.5 MHz, $CDCl_3$): δ/ppm = 159.5 (3C, 4-C), 151.0 (3C, Triazol-3’-C), 140.7 (3C, Triazol-5’-C), 129.7 (3C, 5-C), 124.9 (3C, 1-C), 122.4 (3C, 4-C), 119.0 (3C, 6-C),
115.8 (3C, 2-C), 71.4 (3C, $OCH_2$), 38.1 (3C, CH), 32.1 (6C, $CH_2$), 31.5 (6C, $CH_2$), 30.2 (6C, $CH_2$), 29.7 (6C, $CH_2$), 29.5 (6C, $CH_2$), 27.0 (6C, $CH_2$), 22.8 (6C, $CH_2$), 14.3 (6C, $CH_3$).

**2,6,10-Tris(3-((2-octyldecyl)oxy)phenyl)tris([1,2,4]triazolo)[1,5-a:1',5'-c:1'',5''-e][1,3,5]triazine *r*-63** This compound was prepared according to general procedure DL-*r*-TTT from 85 mg (0.069 mmol) 3,7,11-tris(3-((2-octyldecyl)oxy)phenyl)tris([1,2,4]triazolo)[4,3-a:4',3'-c:4'',3''-e][1,3,5]triazine, 105 mg (0.52 mmol) *p*-bromobenzoic acid and 1.731 g octadecane. The mixture was heated for 26 h at 235 °C and purified via column chromatography. The reaction afforded 18 mg (0.015 mmol, 21 %) of the desired product. $^{1}$H-NMR (400 MHz, $CDCl_3$) δ 8.05 (dt, $^{3}J$ = 7.7 Hz, $^{4}J$ = 1.2 Hz, 3H), 7.98 (dd, $^{4}J$ = 2.6 Hz, $^{4}J$ = 1.5 Hz, 3H), 7.44 (pseudo-t, $^{3}J$ = 8.0 Hz, 3H), 7.12 (ddd, $^{3}J$ = 8.3 Hz, $^{4}J$ = 2.6 Hz, $^{4}J$ = 1.0 Hz, 3H), 3.98 (d, $^{3}J$ = 5.6 Hz, 6H), 1.92 – 1.69 (m, 3H), 1.53 – 1.14 (m, 84H), 0.99 – 0.73 (m, 18H). $^{13}$C-NMR (101 MHz, $CDCl_3$) δ 165.44, 160.01, 144.66, 129.99, 129.31, 120.27, 118.85, 113.09, 71.34, 38.23, 32.08, 31.53, 30.22, 29.85, 29.78, 29.52, 27.05, 22.85, 14.29. IR (neat): ṽ [1/cm] = 2954 m, 2923 s, 2853 s, 1627 s, 1512 m, 1457 s, 1412 m, 1327 s, 1236 m, 1114 m, 1030 m, 912 m, 746 ss, 684 m, 641 m, 619 ss.

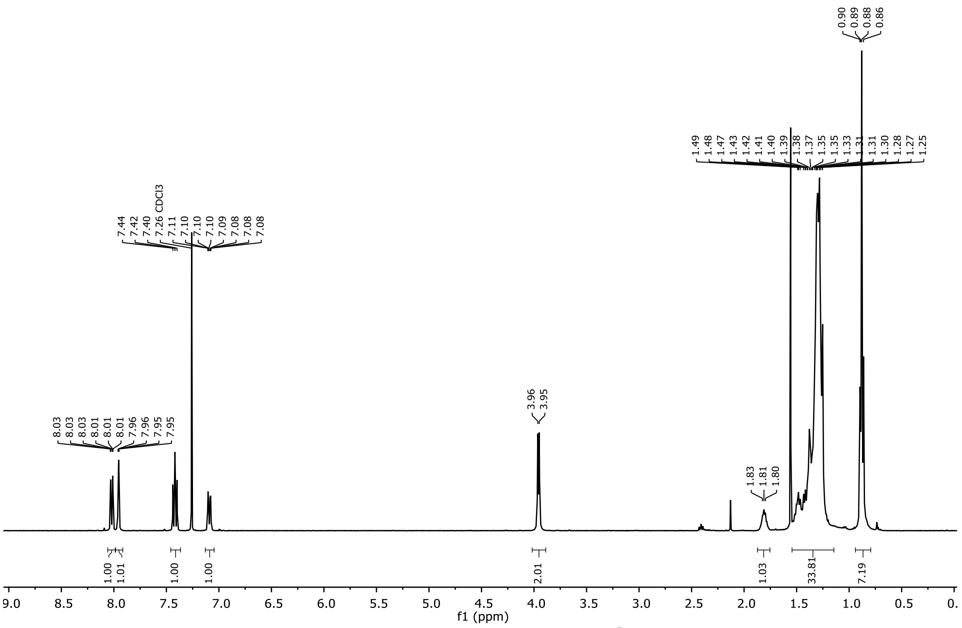


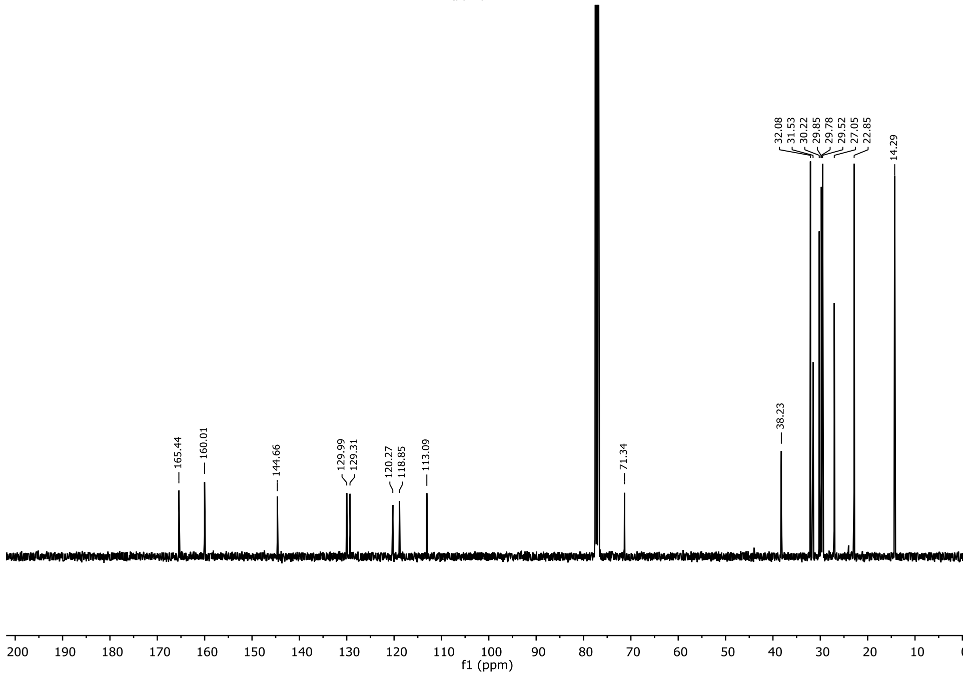

$^1$H- and $^{13}$C-NMR spectra of 2,6,10-Tris(3-((2-octyldecyl)oxy)phenyl)tris([1,2,4]triazolo)[1,5-a:1',5'-c:1'',5''-e][1,3,5]triazine ***r*-63**

**4-((2-Hexyloctyl)oxy)benzonitrile**

This compound was prepared according to the general procedure from 1.66 g (13.9 mmol, 1 eq) 4-hydroxybenzonitrile, 4.40 g (15.9 mmol, 1.1 eq) 1-bromo-2-hexyloctane and 2.16 g (15.6 mmol, 1.1 eq) $K_2CO_3$ in 50 mL acetonitrile. Unreacted starting material was removed by crystallization from ethanol. The crude product, containing excessive bromoalkene, was used without further purification.

**1*H*-5-(4-((2-Hexyloctyl)oxy)phenyl)tetrazole**

This compound was prepared according to the general procedure from 4.23 g (13.4 mmol, 1 eq) 4-((2-hexyloctyl)oxy)benzonitrile, 5.93 g (91.2 mmol, 6.8 eq) sodium azide and 12.65 g (91.9 mmol, 6.9 eq) triethylammonium chloride in 115 mL toluene. Triethylammonium salt was removed by crystallization from ethanol/petroleum ether (1/5). The crude product (1.79 g) was used without further purification. m.p.: 114-117 °C. $^1$H-NMR (300 MHz, $CDCl_3$) δ = 8.13 (d, $^3J$ = 8.8 Hz, 2H), 7.02 (d, $^3J$ = 8.9 Hz, 2H) 3.87 (d, $^3J$ = 5.7 Hz, 2H, O*CH₂*), 2.07 – 1.62 (m, 1H, $OCH_2CH$), 1.56 – 1.09 (m, 20H, $CH_2$), 1.00 – 0.80 (m, 6H, $CH_3$). $^{13}$C-NMR (75 MHz, $CDCl_3$) δ = 162.40, 156.09, 129.40 (2C), 115.58 (2C), 115.18, 71.34, 38.00, 31.97 (2C), 31.42 (2C), 29.80 (2C), 26.92 (2C), 22.80 (2C), 14.24 (2C). IR (neat): ṽ [1/cm] = 2955 m, 2926 s, 2856 m, 2734 w, 1729 w, 1614 m, 1499 m, 1466 m, 1378 w, 1295 m, 1263 ss, 1178 m, 1062 w, 1021 m, 839 m, 736 ss, 704 ss, 868 w, 748 m, 661 w, 638 w, 620 w, 605 w. **HRMS-ES(+):** [M+H]+: calc.: 359.2805 found: 359.2803.

**3,7,11-Tris(4-((2-hexyloctyl)oxy)phenyl)tris([1,2,4]triazolo)[4,3-a:4',3'-c:4'',3''-e][1,3,5]triazine *t*-64**

This compound was prepared according to general procedure from 333 mg (0.929 mmol, 3.2 eq) 1*H*-5-(4-(2-hexyloctyloxy)phenyl)tetrazole **180**, 0.5 mL (3.8 mmol, 13.1 eq) 2,4,6-collidine and 53 mg (0.29 mmol, 1.0 eq) cyanuric chloride in 20 mL xylene. Column chromatography ($SiO_2$ + 2 cm $Al_2O_3$; Tol:EA = 20:1) afforded 130 mg (0.12 mmol, 42 %) of the product as colorless solid. $^1$H-NMR (400 MHz, $CDCl_3$) δ = 8.13 – 8.02 (m, 6H, 2,6-H-Ph), 7.12 – 7.01 (m, 6H, 3,5-H-Ph), 3.93 (d, $^3J$ = 5.6 Hz, 6H, $OCH_2$), 1.87 – 1.77 (m, 3H, $OCH_2CH$), 1.52 – 1.20 (m, 60H, $CH_2$), 1.04 – 0.65 (m, 18H, $CH_3$). $^{13}$C-NMR (101 MHz, $CDCl_3$) δ = 162.42 (4-C-Ph), 151.07 (3,7,11-C), 140.61 (4a,8a,12a-C), 131.96 (2,6-C-Ph), 115.80 (1-C-Ph), 114.67 (3,5-C-Ph), 71.20 ($OCH_2$), 38.03 ($OCH_2CH$), 32.01 (2x$CH_2$), 31.50 (2x$CHCH_2$), 29.84 (2x$CH_2$), 26.97 (2x$CH_2$), 22.84 (2x$CH_2$), 14.28 (2x$CH_3$). IR (neat): ṽ [1/cm] = 2952 m, 2925 ss, 2856 m, 1608 s, 1596 s, 1486 s, 1468 s, 1429 m, 1294 m, 1256 ss, 1179 m, 1016 w, 833 m, 768 m, 750 m. **HRMS-ES(+):** [M+H]+: calc.: 1066.7944 found: 1066.7943.

**p-(2-Octyldecyloxy)benzonitrile**

A suspension of 1.33 g (9.6 mmol) K2CO3, 3.08 g (9.2 mmol) 2-cctyldecylbromide and 1.00 g (8.4 mmol) p-hydroxybenzonitrile in 40 mL toluene and 1 drop Aliquat 336 was refluxed for 7 d. Work-up according to the general procedure and chromatography (SiO2; LM: Tol:PE = 3:1) gave 0.90 g (2.4 mmol, 29 % of a yellowish oil, $n_D^{22}$ = 1.4950. **IR**: 2952 m, 2923 ss, 2853 s, 2224 m, 1605 ss, 1508 ss, 1466 m, 1301 m, 1256 ss, 1170 s , 1018 m, 832 ss, 723 m. **$^1$H-NMR** (300 MHz, $CDCl_3$): δ/ppm = 7.59-7.54 (m, 2H,2-H, 6-H), 6.96-6.91 (m, 2H, 3-H, 5-H), 3.87(d, 2H, $^3J$ = 5.6 Hz, $OCH_2$), 1.83-1.75 (m, 1H, CH), 1.45-1.25 (m, 28H, $CH_2$), 0.88 (t, 6H, $^3J$ = 6.7 Hz, $CH_3$). **$^{13}$C-NMR** (75.5 MHz, $CDCl_3$): δ/ppm = 162.8 (4-C), 134.1 (2-C, 6-C), 119.5 (CN), 115.3 (3-C, 5-C), 103.7 (1-C), 71.4 (O-CH2), 37.9 (CH), 32.0, 31.4, 30.1, 29.7, 29.5, 26.9, 22.8 ($CH_2$), 14.3 ($CH_3$). MS HR-ESI: m/z = 372.3160 [M+H]+, calcd.: 372.3161.

**5-(4-(2-Octyldecyloxy)phenyl)-1H-tetrazole**
Following the general procedure, 0.63 g (1.7 mmol) p-(2-octyldecyloxy)benzonitrile, 0.44 g (6,8 mmol) $NaN_3$ and 0.93 g (6.8 mmol) triethylammonium chloride in 15 mL toluene were refluxed for 7 d. Usual work-up gave 0.3919 g (0.95 mmol, 56 %) of a brownish solid with m.p. = 102 °C. IR: 2952 m, 2920 ss, 2852 s, 2709 w, 2613 w, 2547 w,2465 w, 2365 w, 2337 w, 1611 ss, 1498 ss, 1465 m, 1405 w, 1294 m, 1263 ss, 1250 ss, 1175 ss, 1055 m, 1021 m, 991 m, 836 ss, 751 s, 699 w, 659 w. **$^1$H-NMR** (300 MHz, $CDCl_3$): δ/ppm = 8.05-8.02 (m, 2H, 2-H, 6-H), 7.01-6.98 (m, 2H, 3-H, 5-H), 3.86j (d, $^3J$ = 5.7 Hz, 2H, O-$CH_2$), 1.80-1.74 (m, 1H, CH), 1.42-1.24 (m, 28H, $CH_2$), 0.87 (t, $^3J$ = 6.8 Hz, 6H, $CH_3$). **$^{13}$C-NMR** (75.5 MHz, $CDCl_3$): δ/ppm = 162,.4 (4-C), 156.3 (Tetrazol-C) 129.3 (2-C, 5-C), 115.6 (3-C, 5-C), 115.2 (1-C), 71.4 (O-$CH_2$), 38.0 (CH), 32.0 ($CH_2$), 31.4 ($CH_2$), 30.1 ($CH_2$), 29.7 ($CH_2$), 29.5 ($CH_2$), 29.5 ($CH_2$), 27.0 ($CH_2$), 22.8 ($CH_2$), 14.3 ($CH_3$). MS HR-ESI: m/z = 415.3432 (found, [M+H]+, calcd.: 415.3431.

**3,7,11-Tris(4-(2-octyldecyloxy)phenyl)tris[1,2,4]triazolo[4,3-a:4´,3´-c:4´´,3´´e][1,3,5]-triazine *t*-65**
Following the general procedure, 0.05 mL (46 mg, 0.38 mmol) 2,4,6-collidine, 151.0 mg (0.364 mmol) 5-(4-(2-octyldecyloxy)phenyl)-1H-tetrazole and 21.7 mg (0.118 mmol) cyanuric chloride in 10 mL xylenes were stirred at 20 °C for 135 min, 14 h at 90 °C and 3.5 h at120 °C. Usual work-up and chromatography ($SiO_2$, Tol) gave 89.4 mg (0.072 mmol, 61 %) of a colourless solid. **IR**: 2952 m, 2920 ss, 2851 ss, 1593 .ss, 1484 ss, 1466 ss, 1428 m, 1293 m, 1252 ss, 1177 ss , 1022 m, 831 ss, 729 m; **$^1$H-NMR** (300 MHz, $CDCl_3$): δ/ppm = 8.11-8.07 (m, 6H, 2-CH, 6-CH), 7.10-7.04 (m, 6H, 3-CH, 5-CH), 3.93 (d, $^3J$ = 5.6 Hz, 6H, O-$CH_2$), 1.85-1.79 (m, 3H, CH), 1.51-1.27 (m, 84H, $CH_2$), 0.89 (t, $^3J$ = 6.7 Hz, 18H, $CH_3$). **$^{13}$C-NMR** (75.5 MHz, $CDCl_3$): δ/ppm = 162.4 (3C, 4-C), 151.0 (3C, Triazol-3'-C), 140.6 (3C, Triazol-5'-C), 131.9 (6C, 2-C, 5-C), 115.8 (3C, 1-C), 114.6 (6C, 4-C), 71.2 (3C, OCH2), 38.0 (3C, CH), 32.1 (6C, $CH_2$), 31.5 (6C, $CH_2$), 30.2 (6C, $CH_2$), 29.7 (6C, $CH_2$), 29.5 (6C, $CH_2$), 27.0 (6C, $CH_2$), 22.8 (6C, $CH_2$), 14.3 (6C, $CH_3$).

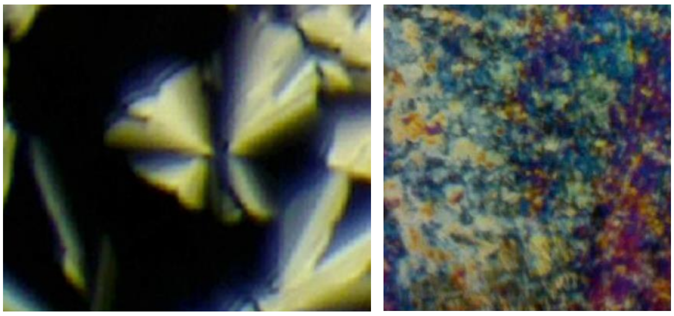

Polmic of ***t*-65** during transition from M to isotropic at 100 °C (left) and after shearing at 95 °C

**2-Decyldodecan-1-ol** 1.88 g (49.5 mmol) lithium aluminum hydride were suspended in 50 mL dry THF, cooled with ice and 5.85 g (crude product) methyl 2-decyldodecanoate were added through a dropping funnel. After completion of the addition, the mixture was refluxed for three hours. Water and aqueous sulfuric acid was added to the mixture and the phases were separated. The aqueous phase was extracted with diethyl ether and the combined organic phases dried over $MgSO_4$. The solvent was removed under reduced pressure and afforded 4.62 g (0.0141 mol, 85 % (2 steps)) of the desired product as brown oil which solidified eventually. **$^1$H-NMR** (400 MHz, $CDCl_3$) δ = 3.54 (d, $^3J$ = 5.5 Hz, 2H), 1.55 – 1.38 (m, 1H), 1.36 – 1.24 (m, 36H), 0.99 – 0.80 (m, 6H). **$^{13}$C-NMR** (101 MHz, $CDCl_3$) δ = 65.91, 40.68, 32.08 (2C), 31.08 (2C), 30.23 (2C), 29.84 (2C), 29.81 (4C), 29.51 (2C), 27.04 (2C), 22.85 (2C), 14.28 (2C). **IR** (neat): ṽ [1/cm] = 3335 br, m, 2956 s, 2921 ss, 2852 ss, 1465 s, 1377 m, 1040 s, 719 s.

**1-Bromo-2-decyldodecane** To a cold solution of 3.60 g (0.011 mol, 1 eq) 2-decyldodecan-1-ol in 0.3 mL (0.006 mol, 0.5 eq) sulfuric acid 1.87 mL (48%, 0.034 mol, 3 eq) hydrobromic acid was added dropwise. The mixture was stirred under reflux and successively sulfuric acid and hydrobromic acid added until the starting material vanished (TLC). Water was added and the mixture was extracted with petroleum ether. The combined organic phases dried over $MgSO_4$. The solvent was removed under reduced pressure and the crude product was purified via column chromatography ($SiO_2$; PE). The reaction afforded 2.755 g (7.07 mmol, 64 %) of the desired product as colorless oil. **$^1$H-NMR** (400 MHz, $CDCl_3$) δ = 3.45 (d, $^3J$ = 4.7 Hz, 2H), 1.63 – 1.54 (m, 1H), 1.42 – 1.16 (m, 36H), 0.97 – 0.83 (m, 6H). **$^{13}$C-NMR** (101 MHz, $CDCl_3$) δ = 39.92, 39.65, 32.71 (2C), 32.07 (2C), 29.94 (2C), 29.79 (4C), 29.75 (2C), 29.51 (2C), 26.72 (2C), 22.85 (2C), 14.29 (2C). **IR** (neat): ṽ [1/cm] = 2954 m, 2922 ss, 2852 s, 1465 m, 1377 w, 1258 w, 1233 w, 721 m.

**4-((2-Decyldodecyl)oxy)benzonitrile**
This compound was prepared according to the general procedure from 250 mg (2.10 mmol, 1 eq) 4-hydroxybenzonitrile, 926 mg (2.38 mmol, 1.1 eq) 1-bromo-2-decyldodecane and 351 mg (2.54 mmol, 1.2 eq) $K_2CO_3$ in 10 mL acetonitrile. Column chromatography ($SiO_2$; PE:Tol = 3:1) afforded 705 mg (1.65 mmol, 79 %) of the desired product as colorless oil.
**$^1$H-NMR** (400 MHz, $CDCl_3$) δ = 7.58 – 7.56 (m (AA'XX'), 2H), 7.01 – 6.89 (m (AA'XX'), 2H), 3.86 (d, $^3J$ = 5.6 Hz, 2H, OC*H₂*), 1.78 (hept, $^3J$ = 5.6 Hz, 1H, $OCH_2CH$), 1.48 – 1.17 (m, 36H, $CH_2$), 0.89 – 0.87(m, 6H, $CH_3$). **$^{13}$C-NMR** (101 MHz, $CDCl_3$) δ = 162.82, 134.07 (2C), 119.52, 115.34 (2C), 103.69, 71.42, 37.92, 32.07, 32.05, 31.37 (2C), 30.10 (2C), 29.80 (2C), 29.78 (2C), 29.75 (2C), 29.50 (2C), 26.94 (2C), 22.84 (2C), 14.28 (2C). **IR** (neat): ṽ [1/cm] = 2922 ss, 2852 s, 2225 m, 1605 s, 1574 m, 1508 ss, 1466 m, 1377 w, 1301 s, 1256 ss, 1170 s, 1111 m, 1013 m, 832 ss, 720 m. **HRMS-ES(+):** [M+H]+: calc.: 428.3887 found: 428.3878.

**1*H*-5-(4-((2-Decyldodecyl)oxy)phenyl)tetrazole**
This compound was prepared according to the general procedure from 560 mg (1.31 mmol, 1 eq) 4-((2-decyldodecyl)oxy)benzonitrile **160**, 0.39 g (5.54 mmol, 4.2 eq) sodium azide and 0.86 g (6.25 mmol, 4.8 eq) triethylammonium chloride in 12 mL toluene. The crude product was recrystallized from toluene and afforded 362 mg (0.769 mmol, 59 %) of the product as colorless solid. m. p.: 98°C (tol). **$^1$H-NMR** (300 MHz, $CDCl_3$) δ = 8.08 – 7.85 (m (AA'XX'), 2H), 7.13 – 6.94 (m (AA'XX'), 2H), 3.89 (d, $^3J$ = 5.7 Hz, 2H, $OCH_2$), 1.96 – 1.64 (m, 1H, $OCH_2CH_2$), 1.50 – 1.25 (m, 36H, $CH_2$), 0.93 – 0.77 (m, 6H, $CH_3$). **$^{13}$C-NMR** (75 MHz, $CDCl_3$) δ = 162.33, 129.10 (2C), 115.53 (2C), 71.37 (2C), 38.02 (2C), 32.07 (2C), 31.42 (2C), 30.15 (2C), 29.82 (2C), 29.79 (4C), 29.50 (2C), 26.98 (2C), 22.84 (2C), 14.27 (2C). **IR** (neat): ṽ [1/cm] = 2920 ss, 2850 s, 2723 w, 2610 w, 2547 w, 2465 w, 1612 s, 1583 w, 1498 s, 1465 m, 1405 m, 1295 m, 1263 ss, 1251 ss, 1175 s, 1055 m, 1021 m, 991 m, 837 s, 751 s, 680 w, 661 w. **HRMS-ES(+):** [M+H]+: calc.: 471.4057 found: 471.4055.

**3,7,11-Tris(4-((2-decyldodecyl)oxy)phenyl)tris([1,2,4]triazolo)[4,3-a:4',3'-c:4'',3''-e][1,3,5]triazine *t*-66**
This compound was prepared according to the general procedure from 357 mg (0.758 mmol, 3.3 eq) 1H-5-(4-(2-decyldodecyloxy)phenyl)tetrazole 184, 0.15 mL (1.1 mmol, 4.8 eq) 2,4,6-collidine and 43 mg (0.23 mmol, 1.0 eq) cyanuric chloride in 20 mL xylene. Column chromatography ($SiO_2$ + 2 cm $Al_2O_3$; Tol) afforded 205 mg (0.15 mmol, 64 %) of the product as colorless solid.

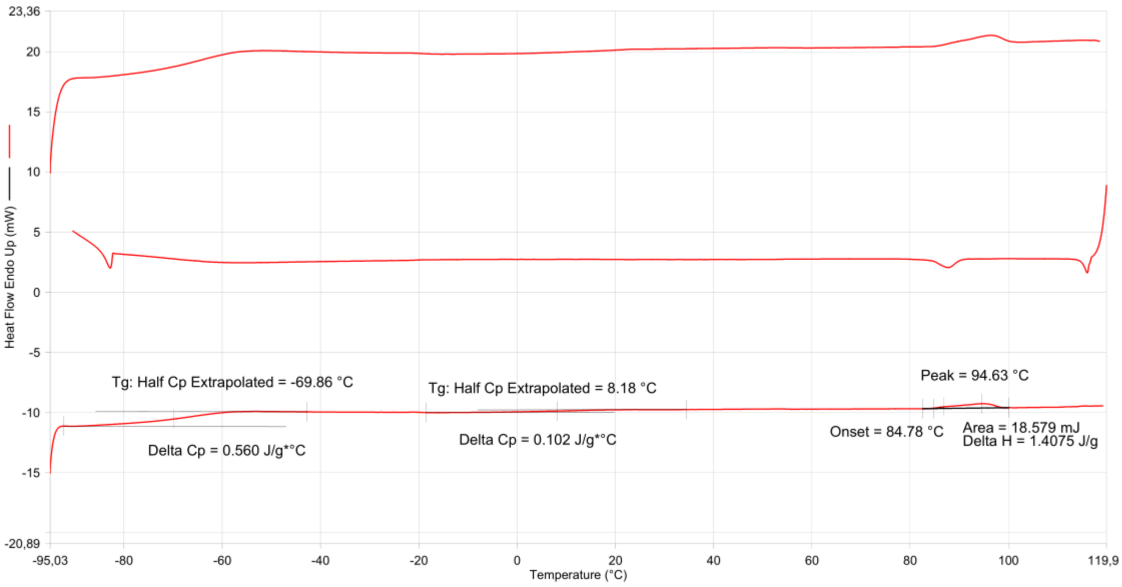


DSC of 3,7,11-Tris(4-((2-decyldodecyl)oxy)phenyl)tris([1,2,4]triazolo)[4,3-a:4',3'-c:4'',3''-e][1,3,5]triazine ***t*-66**

**2,6,10-Tris-(4-(2-decyldodecyloxy)phenyl)tris([1,2,4]triazolo)[1,5-a:1',5'-c:1'',5''-e][1,3,5]triazine *r*-66**

This compound was prepared according to general procedure from 99.7 mg (0.0711 mmol) 3,7,11-tris-(4-(2-decyldodecyloxy)phenyl)([1,2,4]triazolo)[4,3-a:4',3'-c:4'',3''-e][1,3,5]triazine **69**, 95.4 mg (0.474 mmol) *p*-bromobenzoic acid and 1.787 g octadecane. The mixture was heated for 31 h at 235 °C and purified via column chromatography. The reaction afforded 16 mg (0.011 mmol, 16 %) of the desired product. **$^1$H-NMR** (300 MHz, $CDCl_3$) δ = 8.43 – 7.85 (m, AA'XX', 6H), 7.09 – 6.76 (m, AA'XX', 6H), 3.91 (d, $^3J$ = 5.6 Hz, 6H), 2.05 – 1.67 (m, 3H), 1.55 – 1.17 (m, 108H), 0.97 – 0.62 (m, 18H). **$^{13}$C-NMR** (75 MHz, $CDCl_3$) δ = 165.22, 162.35, 144.58, 129.64, 120.46, 114.85, 71.23, 38.08, 32.09, 31.49, 30.19, 29.86, 29.81 (2C), 29.52, 27.02, 22.85, 14.29. **IR** (neat): ṽ [1/cm] = 2952 s, 2921 ss, 2852 ss, 1630 ss, 1608 ss, 1577 m, 1534 m, 1463 ss, 1427 ss, 1378 m, 1331 s, 1303 s, 1250 ss, 1170 ss, 1122 m, 1109 m, 1027 s, 954 m, 839 ss, 753 ss, 712 m, 671 m, 634 w, 614 m.

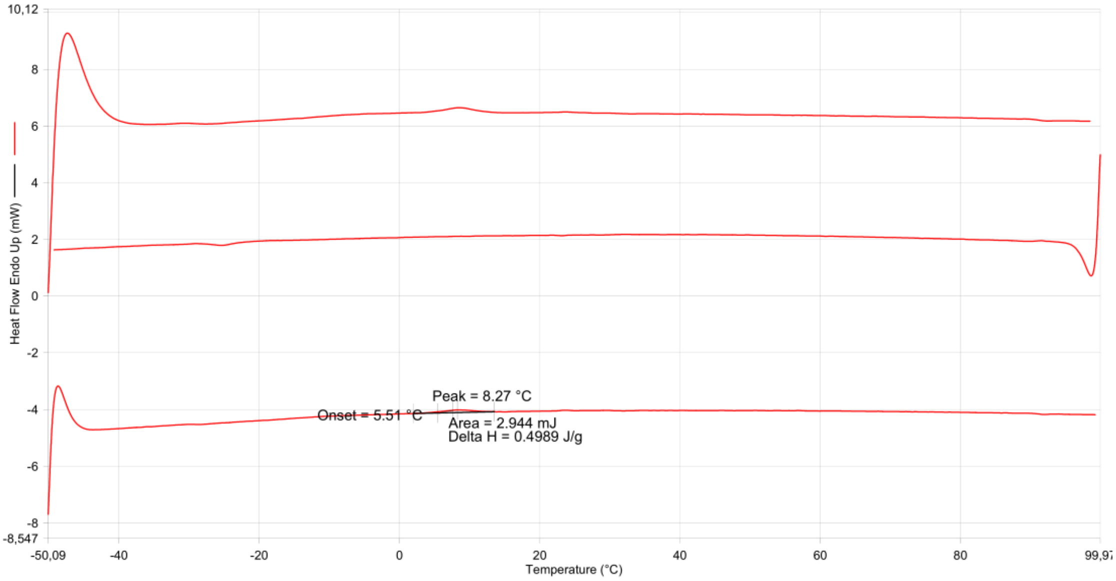


DSC of 2,6,10-Tris-(4-(2-decyldodecyloxy)phenyl)([1,2,4]triazolo)[1,5-a:1',5'-c:1'',5''-e][1,3,5]triazine ***r*-66**

**2-Dodecyltetradecan-1-ol**
1.09 g (28.7 mmol) lithium aluminum hydride were suspended in 50 mL dry THF, cooled with ice and 3.93 g (crude product) methyl 2-dodecyltetradecanoate **202** were added through a dropping funnel. After the completion of the addition the mixture was refluxed for three hours. Water and aqueous sulfuric acid was given to the mixture and the phases were separated. The aqueous phase was extracted with diethyl ether and the combined organic phases dried over $MgSO_4$. The solvent was removed under reduced pressure and the crude product was used without further purification.

**1-Bromo-2-dodecyltetradecane**
3.30 g (8.68 mmol, 1 eq) 2-dodecyltetradecan-1-ol and 0.4 mL (4 mmol, 0.5 eq) phosphorus tribromide were dissolved in 20 mL dry toluene and stirred at room temperature overnight. 1.7 mL hydrobromic acid were added to the mixture and stirred for two days. Water and petroleum ether were given to the mixture and the phases were separated. The aqueous phase was extracted with petroleum ether and the combined organic phases dried over $MgSO_4$. The solvent was removed under reduced pressure and the crude product was purified via column chromatography ($SiO_2$; PE). The reaction afforded 1.98 g (4.44 mmol, 30 % (3 steps)) of the desired product as colorless oil. **$^1$H-NMR** (300 MHz, $CDCl_3$) δ = 3.45 (d, $^3J$ = 4.7 Hz, 2H), 1.70 – 1.54 (m, 1H), 1.43 – 1.18 (m, 44H), 0.99 – 0.78 (m, 6H). **$^{13}$C-NMR** (75 MHz, $CDCl_3$) δ = 39.91, 39.65, 32.71 (2C), 32.09 (2C), 29.94 (2C), 29.85 (2C), 29.83 (2C), 29.81 (2C), 29.80 (2C), 29.75 (2C), 29.53 (2C), 26.71 (2C), 22.86 (2C), 14.29 (2C). **IR** (neat): ṽ [1/cm] = 3330 w, 2921 ss, 2851 s, 1465 m, 1377 w, 1044 m, 719 m.

**4-((2-Dodecyltetradecyl)oxy)benzonitrile**
This compound was prepared according to general procedure from 188 mg (1.58 mmol, 1 eq) 4-hydroxybenzonitrile, 769 mg (1.73 mmol, 1.1 eq) 1-bromo-2-decyldodecane, 32 mg (1.68 mmol, 1.1 eq) $K_2CO_3$ and one drop ofaliquat 336 in 15 mL toluene. Column chromatography ($SiO_2$; PE:Tol = 1:1) afforded 365 mg (0.754 mmol, 47 %) of the desired product as colorless oil. **$^1$H-NMR** (300 MHz, $CDCl_3$) δ = 7.59 – 7.54 (m (AA'XX'), 2H), 7.96 – 6.91 (m (AA'XX'), 2H), 3.86 (d, $^3J$ = 5.7 Hz, 2H, OC*H₂*), 1.82 1.73 (m, 1H, $OCH_2CH$), 1.48 – 1.19 (m, 36H, $CH_2$), 0.90 – 0.86 (m, 6H, $CH_3$). **$^{13}$C-NMR** (75 MHz, $CDCl_3$) δ = 162.82, 134.06 (2C), 119.52, 115.34 (2C), 103.68, 71.41, 37.92, 32.07 (2C), 31.37 (2C), 30.10 (2C), 29.83 – 29.80 (8C), 29.75 (2C), 29.51 (2C), 26.94 (2C), 22.85 (2C), 14.28 (2C). **IR** (neat): ṽ [1/cm] = 2922 ss, 2852 s, 2225 m, 1606 s, 1574 w, 1509 s, 1467 m, 1377 w, 1301 m, 1256 s, 1171 s, 1112 w, 1016 m, 913 w, 833 s, 809 w, 770 m, 721 m. **HRMS-ES(+):** [M·]+: calc.: 482.4356 found: 482.4348.

**1*H*-5-(4-(2-Dodecyltetradecyloxy)phenyl)tetrazole**
This compound was prepared according to general procedure from 301 mg (0.622 mmol, 1 eq) 4-(2-dodecyltetradecyloxy)benzonitrile, 173 mg (2.66 mmol, 4.3 eq) sodium azide and 358 mg (2.60 mmol, 4.2 eq) triethylammonium chloride in 20 mL toluene. The crude product was recrystallized first from petroleum ether and then from ethanol. The reaction afforded 221 mg (0.419 mmol, 67 %) of the desired product as colorless solid. m.p.: 99-102 °C. **$^1$H - NMR** (400 MHz, $CDCl_3$) δ = 8.09 (d, $^3J$ = 8.6 Hz, 2H), 7.02 (d, $^3J$ = 8.6 Hz, 2H), 3.87 (d, $^3J$ = 5.7 Hz, 2H), 1.89 – 1.64 (m, 1H), 1.54 – 1.15 (m, 44H), 0.94 – 0.74 (m, 6H). **$^{13}$C-NMR** (101 MHz, $CDCl_3$) δ = 162.44, 156.13, 129.31 (2C), 115.58 (2C), 115.16, 71.38, 38.02, 32.07, 31.41, 30.16, 29.84 (3C), 29.81, 29.79, 29.51, 26.98, 22.84, 14.27. **IR** (neat): ṽ [1/cm] = 2921 ss, 2852 s, 1736 w, 1615 s, 1503 m, 1466 m, 1451 m, 1294 w, 1254 s, 1178 m, 1017 w, 836 s, 752 m, 720 w, 662 w. **HRMS-ES(+):** [M+H]+: calc.: 527.4683 found: 527.4687.

**3,7,11-Tris(4-((2-dodecyltetradecyl)oxy)phenyl)tris([1,2,4]triazolo)[4,3-a:4',3'-c:4'',3''-e][1,3,5]triazine *t*-67**

Thiscompound was prepared according to general procedure from 186 mg (0.353 mmol, 3.3 eq) 1*H*-5-(4-((2-dodecyltetradecyl)oxy)phenyl)tetrazole, 0.05 mL (0.4 mmol, 5.7 eq) 2,4,6-collidine and 20 mg (0.108 mmol, 1.0 eq) cyanuric chloride in 15 mL xylene. Column chromatography ($SiO_2$ + 2 cm $Al_2O_3$; Tol) afforded 101 mg (0.064 mmol, 60 %) of the product as colorless solid. **$^1$H-NMR** (300 MHz, $CDCl_3$) δ = 8.37 – 8.05 (m, 6H, 2,6-H-Ph), 7.16 – 6.88 (m, 6H, 3,5-H-Ph), 3.93 (d, $^3J$ = 5.6 Hz, 6H, OC*H₂*), 1.94 – 1.71 (m, 3H, $OCH_2H$), 1.52 – 1.17 (m, 132H, $CH_2$), 0.98 – 0.76 (m, 18H, $CH_3$). **$^{13}$C-NMR** (75 MHz, $CDCl_3$) δ = 162.42, 151.06, 140.61, 131.96, 115.79, 114.66, 71.21, 38.03, 32.08, 31.48, 30.18, 29.85, 29.81, 29.52, 27.01, 22.85, 14.28. **IR** (neat): ṽ [1/cm] = 2921 ss, 2852 ss, 1738 w, 1594 s, 1485 s, 1467 s, 1428 m, 1375 w, 1295 m, 1254 ss, 1179 s, 1017 m, 832 m, 729 m. **HRMS-ES(+):** [M·]+: calc.: 1571.3578 found: 1571.3544.

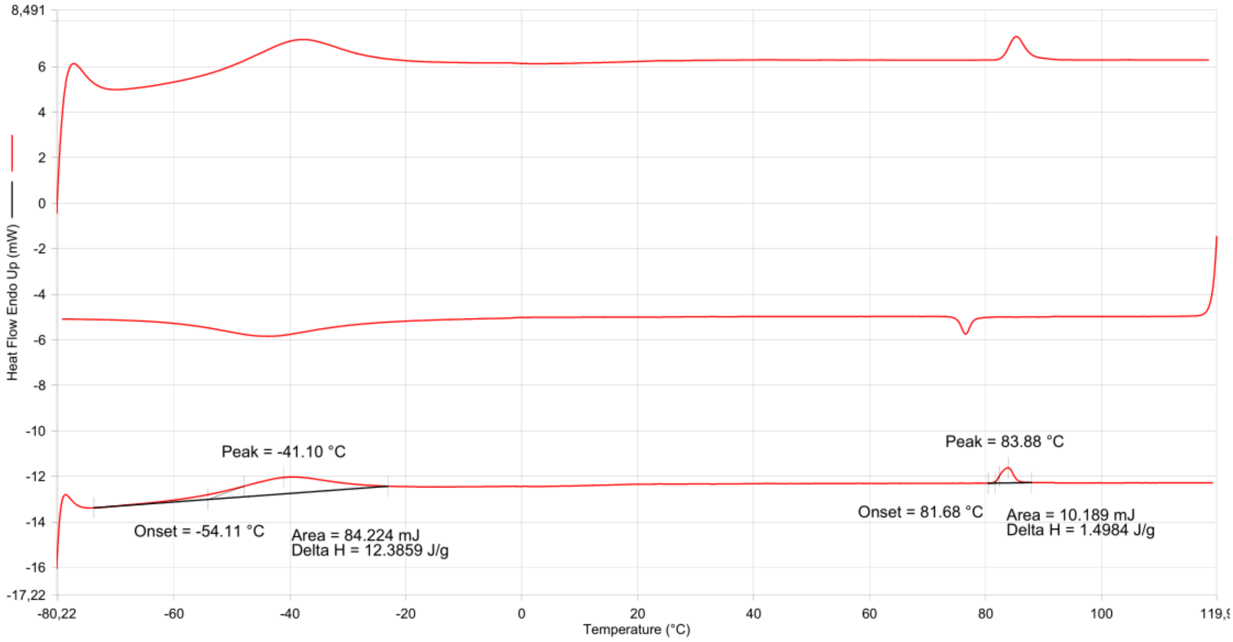


DSC of 3,7,11-Tris(4-((2-dodecyltetradecyl)oxy)phenyl)tris([1,2,4]triazolo)[4,3-a:4',3'-c:4'',3''-e][1,3,5]triazine

***p*-(1-Hexylheptyloxy)benzonitrile**

According to the general procedure, 3.50 g 7-Bromotridecane (13.3 mmol), 1,61 g *p*-hydroxybenzonitrile (13.3 mmol), 2.04 g $K_2CO_3$ (14.76 mmol) one drop Aliquat 336 in 40 mL toluene gave, after chromatography (SiO2, PE →Tol) 0.55 g (1.84 mmol, 14%) of a yellow-ish oil. $n_D^{22}$= 1.4760 ; **$^1$H-NMR** (300 MHz, $CDCl_3$)**:** δ (ppm): 7.58 – 7.52 (m, 2H), 6.93 – 6.87 (m, 2H), 4.28 (quint, $^3J$ = 5.9 Hz, 1H, OCH), 1.70 – 1.56 (m, 4H, $CHCH_2$), 1.35 – 1.19 (m, 20H), 0.92 - 0.78 (m, 6H, $CH_3$). **$^{13}$C-NMR** (75 MHz, $CDCl_3$)**:** 162.1, 134.0 (2C), 119.4, 116.0 (2C), 103.1, 78.5, 33.7 (2C), 31.7 (2C), 29.3 (2C), 25.2 (2C), 22.6 (2C), 14.1 (2C). **IR:** 2927 (s), 2857 (m), 2224 (s), 1895 (vw), 1718 (w), 1603 (vs), 1570 (w), 1505 (vs), 1464 (w), 1377 (w), 1297 (m), 1255 (vs), 1219 (w), 1169 (s), 1124 (w), 1011 (w), 961 (w), 913 (m), 833 (s), 772 (vs), 744 (m), 700 (vw). **MS:** $C_{20}H_{31}NO + H^+$: m/z calcd: 302.2478; found: 302.2475.

**1*H*-5-(4-(1-Hexylheptyloxy)phenyl)tetrazole**

According to the general procedure, 0.55 g *p*-(1-Hexylheptyloxy)benzonitrile (1.83 mmol) and 0.48 g $NaN_3$ (65,1 mmol, 4,0 Äq) and 1.02 g Triethylammonium chloride (7.41 mmol) in

20 mL abs. toluene gave, after 6 h relux and acidulation with 2M HCl, followed by chromatography (SiO2, Tol →EE) 479 mg (1.40 mmol, 77 %) of a brownish solid with m.p. 83 °C. **$^1$H-NMR (**300 MHz, $CDCl_3$)**:** δ (ppm): 8.08 – 8.01 (m, 2H), 7.04 – 6.97 (m, 2H), 4.31 (quint, $^3J$ = 5.8 Hz, 1H, OCH), 1.75 – 1.55 (m, 4H, CHC*H*2), 1.50 – 1.14 (m, 16H), 0.94 – 0.79 (m, 6H, CH3). **$^{13}$C-NMR** (75 MHz, $CDCl_3$)**:** δ (ppm): 161.7, 155.9, 129.3 (2C), 116.4 (2C), 114.8, 78.3, 33.8 (2C), 31.8 (2C), 29.4 (2C), 25.3 (2C), 22.6 (2C), 14.1 (2C). **IR:** 2929 (s), 2857 (m), 2737 (w), 2631 (w), 1613 (vs), 1496 (vs), 1455 (m), 1293 (m), 1257 (vs), 1178 (m), 1125 (w), 1059 (w), 1019 (w), 962 (w), 913 (vs), 837 (s), 748 (vs), 698 (w) **MS:** $C_{20}H_{31}N_4O + H^+$: m/z calcd.: 345.2649, found.: 345.2648

**3,7,11-Tris(4-(1-hexylheptyloxy)phenyl)tris[1,2,4]-triazolo[4,3-a:4´,3´-c:4´´,3´´-e][1,3,5]triazine *t*-68**

According to the general procedure 367 mg 1*H*-5-(4-(1-hexylheptyloxy)phenyl)tetrazole (1.07 mmol), 0.15 mL Collidin (1.13 mmol) in 20 mL abs. xylenes and 48 mg cyanuric chloride (0.32 mmol) were stirred for 6 h at ambient temperature, for 15 h at 60 °C and 8 h at 90°C, finally 15 h at 120 °C. Cheromatography (SiO2, 1 cm AlOx (basic), Tol:EE 60:1) gave 163 mg (0.16 mmol, 50 %) of a viscous oil with m.p. 9 °C (DSC, Chloroform). **$^1$H-NMR** (300 MHz, $CDCl_3$)**:** δ (ppm): 8.15 – 8.07 (m, 6H), 7.08 – 7.01 (m, 6H), 4.34 (quint, $^3J$ = 5.8 Hz, 3H, OCH), 1.79 – 1.59 (m, 12H, CHC*H*2), 1.51 – 1.19 (m, 48H), 0.98 -0.79 (m, 18H, CH3). **$^{13}$C-NMR** (75 MHz, $CDCl_3$)**:** δ (ppm): 161.7 (3C), 151.0 (3C), 140.5 (3C), 131.9 (6C), 115.4 (6C), 78.2, 33.9 (6C), 31.8 (6C), 29.4 (6C), 25.4 (6C), 22.6 (6C), 14.1 (6C) . **IR:** 2927 (s), 2856 (m), 1736 (w), 1591 (vs), 1480 (vs), 1427 (m), 1376 (w), 1292 (m), 1250 (vs), 1177 (s), 1122 (w), 1096 (w), 1005 (w), 964 (w), 913 (m), 833 (m), 742 (m), 662 (w) . **MS:** $C_{63}H_{93}N_9O_3$ + H+: m/z calcd: 1024.7474, found: 1024.7489.

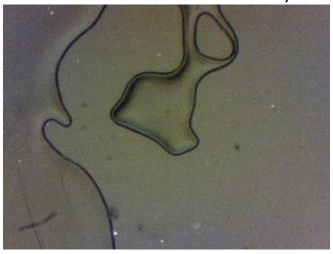

POM of ***t*-68** at ambient temperature

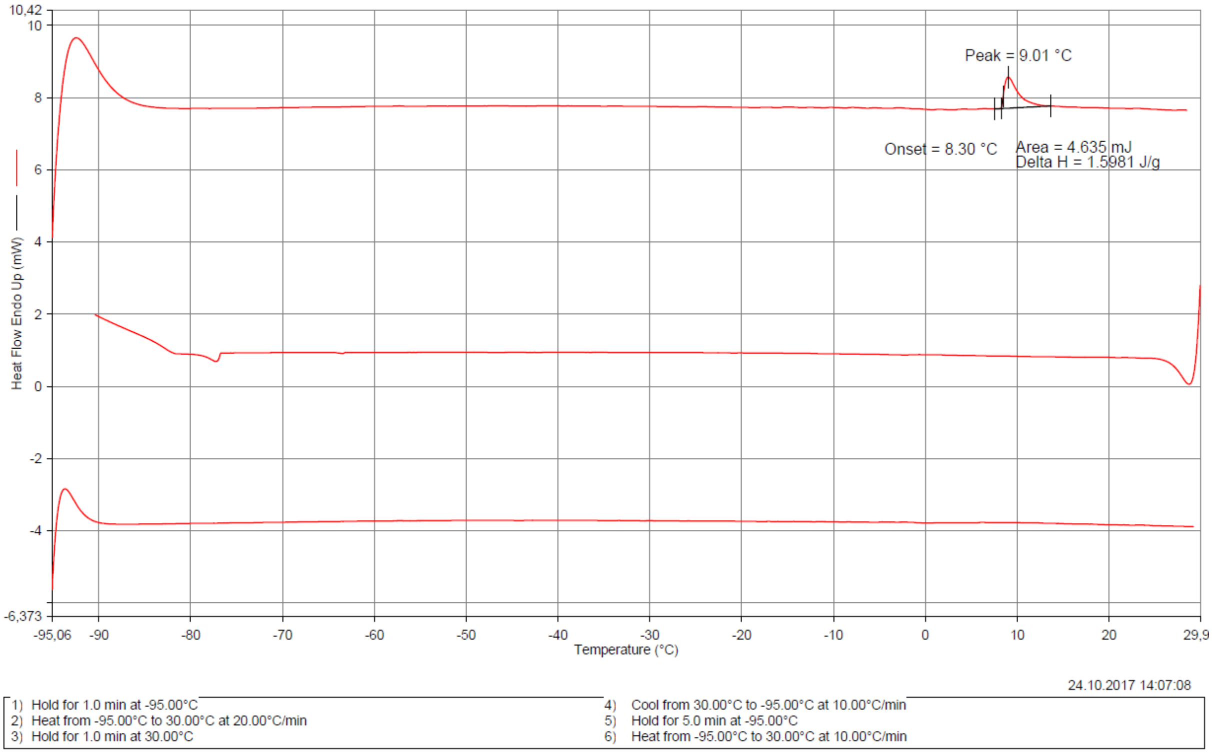


DSC of ***t*-68**

**2,6,10-Tris(4-(tridecan-7-yloxy)phenyl)tris([1,2,4]triazolo)[1,5-a:1',5'-c:1'',5''-e][1,3,5]triazine *r*-68**

This compound was prepared according to general procedure from 73 mg (0.071 mmol) 3,7,11-tris(4-(tridecan-7-yloxy)phenyl)tris([1,2,4]triazolo)[4,3-a:4',3'-c:4'',3''-e][1,3,5]triazine **73**, 95 mg (0.47 mmol) *p*-bromobenzoic acid and 1.697 g octadecane. The mixture was heated for 26 h at 235 °C and purified via column chromatography. The reaction afforded 20 mg (0.020 mmol, 27 %) of the desired product. **$^1$H-NMR** (400 MHz, $CDCl_3$) δ 8.35 (d, $^3J$ = 8.8 Hz, 6H), 7.00 (d, $^3J$ = 8.9 Hz, 6H), 4.35 (p, $^3J$ = 5.8 Hz, 3H, OC*H*), 1.87 – 1.55 (m, 12H, OCHC*H$_2$*), 1.58 – 1.11 (m, 48H, C*H$_2$*), 1.04 – 0.77 (m, 18H, C*H$_3$*). **$^{13}$C-NMR** (101 MHz, $CDCl_3$) δ 165.32, 161.81, 144.66, 129.78, 120.29, 115.89, 78.26, 34.01, 31.93, 29.53, 25.45, 22.76, 14.24. **IR** (neat): ṽ [1/cm] = 2923 s, 2854 m, 1630 s, 1606 ss, 1577 m, 1531 w, 1466 ss, 1426 s, 1377 m, 1330 m, 1302 m, 1247 ss, 1170 s, 1122 m, 1064 m, 1010 m, 964 m, 839 m, 754 s, 713 m, 665 w, 634 w. **HRMS-ES(+):** [M+H]+: calc.: 1024.7474 found: 1024.7459.

**3-Hexylnonylbromide**

1.9 g (8.47 mmol) 3-Hexylnonan-1-ol, 0.4 mL conc. $H_2SO_4$ and 2.05 mL (19 mmol) 48% hydrobromic acid were stirred for 29 h under reflux. Dilution with water, extraction with PE and chromatography yielded 0.815 g (2.80 mmol, 33 %) of a colourless oil, $n_D^{22}$= 1,462. **IR** ($cm^{-1}$) = 2957 (m), 2925 (vs), 2856 (m), 1595 (w), 1466 (m), 1379 (w), 1254 (w), 1088 (w), 721 (w), 647 (w), 607 (w). **$^1$H-NMR** (300 MHz, $CDCl_3$): 3.42 (t, 3J = 7.4 Hz, 2H, BrCH2), 1.81 (td, 3J = 7.5, 6.4 Hz, 2H, $BrCH_2CH_2$), 1.49 (m, 1H, CH), 1.42 _ 1.19 (m, 20H, CH2), 0.99 _ 0.84 (m, 6H, $CH_3$). **$^{13}$C-NMR** (75 MHz, $CDCl_3$): = 37.27 (1C), 36.48 (1C), 33.11 (2C), 32.34 (1C), 31.88 (2C), 29.68 (2C), 26.38 (2C), 22.69 (2C), 14.12 (2C).

**4-(3-Hexylnonyloxy)benzonitrile**

0.492 g (1.69 mmol) 3-Hexylnonylbromide, 0.168 g (1.41 mmol) 4-hydroxybenzonitrile, 0.237 g (1.71 mmol) $K_2CO_3$ andnd 15 mL toluene with 1 drop Aliquat 336 were stirred for 2d under reflux. Usual work-up and chromatography ($SiO_2$; PE to PE/Tol = 2/1) gave 0.29 g (0,89 mmol, 63 %) of a viscous oil, $n_D^{22}$ = 1,499. **IR** : 3839 (w), 3725 (w), 3709 (w), 3628 (w), 3601 (w), 2951 (m), 2925 (s), 2855 (m), 2360 (w), 2342 (w), 2225 (m), 1606 (vs),1574 (m), 1508 (vs), 1467 (m), 1393 (w), 1377 (w), 1301 (s), 125 (vs), 1171 (s), 1112 (w), 1012 (m), 833 (s), 72 (m), 707 (m),678 (m), 659 (w), 609 (w). **$^1$H-NMR** (300 MHz, $CDCl_3$): 7.60 _ 7.55 (m, 2H), 6.95 - 6.90 (m, 2H), 4.01 (t, $^3$J = 6.9 Hz, 2H, $OCH_2$), 1.75 (pseudoq, $^3$J = 6.7 Hz, 2H, $OCH_2CH_2$), 1.53 (m, 1H, CH), 1.38 - 1.25 (m, 20H, $CH_2$), 0.93 -0.83 (m, 6H, $CH_3$). **$^{13}$C-NMR** (75 MHz, CDCl3): _ (ppm) = 162.42 (1C), 133.95(2C), 119.35 (1C), 115.18 (2C), 103.61 (1C), 66.85 (1C), 34.51 (1C), 33.66 (2C), 32.80 (1C), 31.89 (2C), 29.69 (2C), 26.51 (2C), 22.69 (2C), 14.12 (2C). **HR-APCI-MS** m/z (Calc) = 330.2791, m/z (Found) = 330.2784.

**5-(4-(3-Hexylnonyloxy)phenyl)-1H-tetrazole**

Following the general procedure, 0.220 g (0.67 mmol) 4-(3-hexylnonyloxy)benzonitrile, 0.176 g (2.71 mmol) $NaN_3$ and 0.370 g (2.69 mmol) triethylammonium chloride in 10 mL toluene were stirred for 3 d under reflux. Addieitian reagents (0.088 g (1.35 mmol) $NaN_3$ , 0.187 g (1.36 mmol) $NEt_3HCl$) and 72 h reflux yielded, after work-up and chromatography ($SiO_2$; Tol to Tol/EE = 2/1) 0.157 g (0.42 mmol 63 %) of an off-white solid with m.p. = 111 °C. **IR** = 3734 (w), 3630 (w), 3598 (w), 2914 (vs), 2925 (s), 2849 (m), 1645 (m), 1602 (m), 1559 (s), 1470 (s), 1429 (m), 1401 (m), 1264 (s), 1193 (m), 1125 (m), 1075 (m), 1042 (s), 1006 (m), 984 (m), 952 (w), 876 (m), 862 (m), 808 (w), 766 (m), 747 (s), 718 (m), 693 (s). **$^1$H-NMR** (300 MHz, $CDCl_3$ + DMSO-$d_6$): 8.07 - 7.79 (m, 2H), 7.00 - 6.79 (m, 2H), 4.01 (t, $^3$J = 6.9 Hz, 2H, $OCH_2$), 1.75 (pseudo-q, $^3$J = 6.8 Hz, 2H, $OCH_2CH_2$), 1.53 (m, br, 1H, CH), 1.38 - 1.25 (m, 20H, $CH_2$), 0.87 - 0.63 (m, 6H, $CH_3$). **$^{13}$C-NMR** (75 MHz, $CDCl_3$, DMSO-$d_6$): 160.52, 128.06 (2C), 114.22(2C), 65.81, 33.75, 32.88 (2C), 32.14, 31.08 (2C), 28.88 (2C), 25.70 (2C), 21.87 (2C), 13.31 (2C). **HR-APCI-MS** m/z Calcd. = 373.2962, Found = 373.2964

**3,7,11-Tris(4-(3-hexylnonyloxy)phenyl)tris[1,2,4]-triazolo[4,3-a:4´,3´- c:4´´,3´´-e][1,3,5]triazine *t*-69**

According tot he general procedure, 0.122 g (0.33 mmol) 5-(4-(3-hexylnonyloxy)phenyl)-1*H*-tetrazole, 0.05 mL (0.38 mmo) collidine and 15 mL abs. xylenes were stirred for 60 min at ambient temperature and 0.11 mmol cyanuric chloride was added, Stirring für 3 h at ambient temperature, 18 h at 62 °C, 6 h at 92 °C and 18 h at 120 °C gave, after repeated chromatography ($SiO_2$; Tol) 0.065 g (0.05 mmol, 47 %) of an off-white wax. **IR** = 2954 (m), 2924 (s), 2854 (m), 1720 (w), 1610 (s), 1602 (m), 1594 (s), 1524 (w), 1485 (s), 1470 (s), 1428 (m), 1393 (w), 1378 (w), 1294 (m), 1252 (vs), 1179 (s), 1120 (w), 1097 (w), 1007 (m), 952 (w), 876 (m), 833 (m), 729 (m), 699 (w), 664 (w), 638 (w), 615 (w). **$^1$H-NMR** (300 MHz, $CDCl_3$): 8.15 - 8.06 (m, 6H, Ph-H), 7.11 - 7.01 (m, 6H, Ph-H), 4.08 (t, 3J = 6.9 Hz, 6H, OCH2), 1.79 (pseudo-q, $^3$J= 6.7 Hz, 6H, $OCH_2$CH2), 1.59 (m, 3H, CH), 1.29 (m, 60H, $CH_2$), 0.96 - 0.80 (m, 18H, $CH_3$). **$^{13}$C-NMR** (75 MHz, $CDCl_3$): 162.02 (3C), 150.89 (3C), 140.47 (3C), 131.84 (6C), 115.73 (3C), 114.50 (6C), 66.68 (3C), 34.61 (3C), 33.71 (6C), 32.99 (3C), 31.93 (6C), 29.74 (6C), 26.54 (6C), 22.71 (6C), 14.14 (6C). **HR-APCI-MS** m/z calcd: 1108.8413, found: 1108.839.

**1-Bromo-2-(3-methylbutyl)-5-methylhexane**

15.2 g 2-(3-Methylbutyl)-5-methylhexan-1-ole (81.7 mmol) and, while cooling with ice, addition of $H_2SO_4$ (2 mL) and 13.5 mL HBr (48%), the mixture was refluxed for 2 h, 2.5 mL HBr were added and afte 2h h diluted with water, extracted with petrouleum ether and chromatography ($SiO_2$, PE) gave 18.7 g (76.9 mmol, 94 %) of a yellowish oil. $n_D^{26}$= 1,4483; **$^1$H-NMR** (300 MHz, $CDCl_3$)**:** δ (ppm): 3.45 (d, 3*J* = 4.7 Hz, 2H, BrCH), 1.60 – 1.44 (m, 3H), 1.42 – 1.30 (m, 4H), 1.22 – 1.08 (m, 4H), 0.89 (d, 3*J* = 6.6 Hz, 12H, $CH_3$). **IR:** 2954 (m), 2931 (w), 2869 (w), 1467 (m), 1384 (w), 1367 (w), 1219 (m), 772 (vs).

***p*-(2-(3-Methylbutyl)-5-methylhexyloxy)benzonitrile**

Following the general procedure, 4.01 g 1-bromo-2-(3-methylbutyl)-5-methylhexane (16.1 mmol), 1.91 g *p*-hydroxybenzonitrile (16.1 mmol) and 2.45 g $K_2CO_3$ (17.7 mmol) in 50 mL acetonitrile were refluxed for 67 h. Chromatography ($SiO_2$, Tol:PE 1:2) yielded 2.71 g (9.43 mmol, 59 %) of a yellowish oil, $n_D^{26}$= 1,6783. **$^1$H-NMR** (300 MHz, $CDCl_3$)**:** δ (ppm): 7.65 – 7.56 (m, 2H), 7.00 – 6.93 (m, 2H), 3.89 (d, 3*J* = 5.7 Hz, 2H), 1.77 (pseudo-quint, $^3J$ = 5,7 Hz, 1H), 1.62 – 1.32 (m, 6H), 1.29 -1.16 (m, 4H), 0.91 (d, $^3J$ = 6.6 Hz, 12H, $CH_3$). **$^{13}$C-NMR** (75 MHz, $CDCl_3$)**:** δ (ppm): 126.7, 133.9 (2C), 119.4, 115.2 (2C), 103.6, 71.2, 38.2, 36.0 (2C), 28.9 (2C), 28.3 (2C), 22,7 (2C), 22.6 (2C). **IR:** 2953 (m), 2931 (m), 2865 (m), 2224 (m), 1605 (s), 1573 (w), 1508 (s), 1497 (m), 1467 (m), 1384 (w), 1366 (w), 1300 (m), 1256 (vs), 1219 (w), 1170 (s), 1110 (w), 1013 (w), 970 (w), 913 (s), 833 (s), 772 (s), 745 (s). **MS:** $C_{19}H_{29}NO$ + $H^+$: m/z calcd.: 288.2322, found: 288.2317.

**1*H*-5-((2-(3-Methylbutyl)-5-methylhexyloxy)phenyl)tetrazole**

1.49 g *p*-(2-(3-Methylbutyl)-5-methylhexyloxy)benzonitrile (5.19 mmol), 1.37 g $NaN_3$ (21.1 mmol) and 2.89 g Triethylammonium chloride (20.9 mmo) in 40 mL abs. toluene gave, following the general procedure, after 3 d reflux and chromatography (SiO2, Tol →EE)) 1.15 g (3.48 mmol, 67 %) of a off-white solidt with m.p. = 224 °C. **$^1$H-NMR** (300 MHz, $CDCl_3$, $d_6$-DMSO)**:** δ (ppm): 7.96 – 7.86 (m, 2H), 6.95 – 6.86 (m, 2H), 3.81 – 3.76 (m, 2H, OCH2), 1.74 – 1.57 (m, 1H, $OCH_2$C*H*), 1.51 – 1.21 (m, 6H), 1.19 -1.00 (m, 4H), 0.87 – 0.69 (m, 12H, $CH_3$). **$^{13}$C-NMR** (75 MHz, $CDCl_3$, $d_6$-DMSO)**:** δ (ppm): 161.1, 128.4 (2C), 114.6 (2C), 70.6, 37.8, 35.5 (2C), 28.4 (2C), 27.8 (2C), 22.2 (2C), 22.1 (2C). **IR:** 2954 (s), 2926 (s), 2869 (m), 1614 (vs), 1501 (vs), 1467 (m), 1254 (vs), 1186 (m), 1024 (vs), 837 (s), 753 (w). **MS:** $C_{19}H_{30}N_4O$ + $H^+$: m/z calcd: 331.2492, found: 331.2492.

**3,7,11-Tris((2-(3-methylbutyl)-5-methylhexyloxy)phenyl)tris[1,2,4]-triazolo[4,3-a:4´,3´-c:4´´,3´´-e][1,3,5]triazine *t*-70**

The reaction of 288 mg 1*H*-5-((2-(3-methylbutyl)-5-methylhexyloxy)phenyl)tetrazole (0.91 mmo) with 41 mg cyanuric chloride (0.27 mmol) in the presence of 0.12 mL collidine (0.91

mmol) in 20 mL abs. xylenes following bthe general procedure gave, after 3 h at room temperature and heating for 15 h to 60 °C, 8 h to 90 °C and 15 h at 120 °C, followed by chromatograhy ($SiO_2$, 1cm AlOx (basic), Tol:EE 40:1) 156 mg (0.16 mmol, 59 %) of a glassy solid. glasartigen Feststoffs als Produkt erhalten. **$^1$H-NMR(**300 MHz, $CDCl_3$)**:** δ (ppm): 8,10 (d, 3*J*=8,8 Hz, 6H, C14H), 7,08 (d, $^3J$=8,8 Hz, 6H, C15H), 3,94 (d, $^3J$=6,0 Hz, 6H), 1,79 (pseudo-quint, $^3J$= 6,0 Hz, 3H), 1,55 (m, 12H), 1,43 (m, 12H), 1,24 (td, $^3J$=7,8, $^3J$ = 6,5 Hz, 12H), 0,91(d, $^3J$=6,5 Hz, 36H). **$^{13}$C-NMR** (75 MHz, $CDCl_3$)**:** δ (ppm): 162,3 (C16), 150,9 (C3,7,11), 140,5 (C4,8,12), 131,8 (C14), 115,7 (C13), 114,5 (C15), 71,0 (C17), 38,3 (C18), 36,0 (C20), 29,0 (C19), 28,4 (C21), 22,7 (C22). **IR:** 2952 (s), 2926 (s), 2868 (m), 1737 (w), 1592 (vs), 1483 (s), 1467 (s), 1428 (m), 1383 (w), 1366 (w), 1294 (m), 1252 (vs), 1179 (s), 1096 (vw), 1015 (w), 913 (m), 832 (s), 743 (s), 703 (w), 666 (w). **MS:** $C_{60}H_{87}N_9O_3$ + $H^+$: calcd: 982.7005, found: 982.7048.

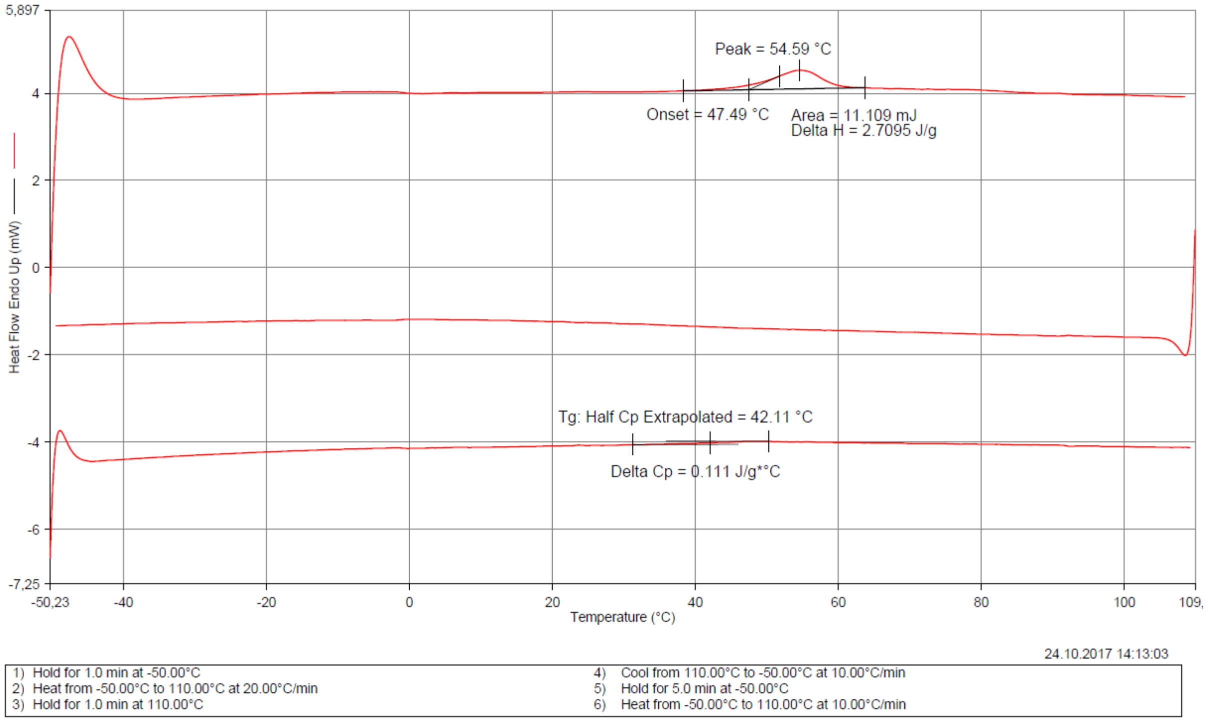


DSC of ***t*-70**

**Dimethyl 2,2-bis(2-ethylhexyl)malonate** Freshly ground potassium hydroxide (14.7 g, 0.262 mol, 3.0 eq) was stirred in 120 mL DMSO and 120 mL toluene. After 15 minutes 10 mL (0.087 mol, 1 eq) dimethyl malonate and 34 mL (0.19 mol, 2.2 eq) 1-bromo-2-ethylhexane were added to the mixture and stirred at room temperature. After 24 h additional 18 mL (0.10 mol, 1.2 eq) 1-bromo-2-ethylhexane were added to the mixture. The reaction was quenched after 72 h with water and the phases were separated. The aqueous phase was extracted with diethyl ether and the combined organic phases dried over $MgSO_4$. The solvent was removed under reduced pressure and the crude product was vacuum distilled and used without further purification.

**Methyl 4-ethyl-2-(2-ethylhexyl)octanoate**
14.18 g (crude product) dimethyl 2,2-didecylmalonate and 8.7 g (0.15 mol) sodium chloride were suspended in 50 mL DMSO and heated to reflux for 120 h. Water was given to the mixture and the phases were separated. The aqueous phase was extracted with diethyl ether and the combined organic phases dried over $MgSO_4$. The solvent was removed under reduced pressure and the crude product was used without further purification.

**4-Ethyl-2-(2-ethylhexyl)octan-1-ol**
2.54 g (66.9 mmol) lithium aluminum hydride were suspended in 40 mL dry THF, cooled with ice and 6.54 g (crude product) methyl 4-ethyl-2-(2-ethylhexyl)octanoate, dissolved in 20 mL THF, were added through a dropping funnel. After the completion of the addition the mixture was refluxed for three hours. Water and aqueous sulfuric acid was given to the mixture and the phases were separated. The aqueous phase was extracted with diethyl ether and the combined organic phases dried over $MgSO_4$. The solvent was removed under reduced pressure and afforded 3.25 g (0.0120 mol, 14 % (3 steps)) of the desired product as colorless oil. The obtained spectra are a superimposition of (2r,4S)-4-ethyl-2-((R)-2-ethylhexyl)octan-1-ol, (2s,4S)-4-ethyl-2-((R)-2-ethylhexyl)octan-1-ol and (S)-4-ethyl-2-((S)-2-ethylhexyl)octan-1-ol or (R)-4-ethyl-2-((R)-2-ethylhexyl)octan-1ol. **$^1$H NMR** (300 MHz, $CDCl_3$) δ = 3.52 (d, $^3J$ = 5.0 Hz, 2H), 1.64 – 1.52 (m, 1H), 1.37 – 1.18 (m, 21H), 1.14 – 1.02 (m, 2H), 0.96 – 0.77 (m, 12H). **$^{13}$C-NMR** (75 MHz, $CDCl_3$) δ = 66.56, 66.51, 36.43, 36.40, 36.32, 36.13, 36.09, 35.73, 35.67, 33.33, 33.16, 33.14, 29.07, 28.77, 28.76, 26.42, 26.38, 26.14, 23.34, 23.31, 14.32, 10.96, 10.92, 10.65. **IR** (neat): ṽ [1/cm] = 3334 m, 2957 ss, 2923 ss, 2858 s, 1460 s, 1379 m, 1051 m, 1029 m, 809 w, 765 w, 743 w, 729 w. **HRMS-ES(+):** [M+H]+: calc.: 267.2682 found: 267.268.

**7-(Bromomethyl)-5,9-diethyltridecane**
3.23 g (0.012 mol, 1 eq) 4-ethyl-2-(2-ethylhexyl)octan-1-ol was cooled to -10 °C and 0.45 mL (4.7 mmol, 0.4 eq) phosphorus tribromide were slowly added. The mixture was stirred at room temperature overnight. 2.0 mL hydrobromic acid (62 %) were added to the mixture and stirred for two days. Water and petroleum ether were given to the mixture and the phases were separated. The aqueous phase was extracted with petroleum ether and the combined organic phases dried over $MgSO_4$. The solvent was removed under reduced pressure and the crude product was purified via column chromatography ($SiO_2$; PE). The reaction afforded 1.71 g (5.13 mmol, 43 %) of the desired product as colorless oil. The obtained spectra are a superimposition of (5R,7s,9S)-7-(bromomethyl)-5,9-diethyltridecane, (5R,7r,9S)-7-(bromo-methyl)-5,9-diethyltridecane and (5S,9S)-7-(bromomethyl)-5,9-diethyltridecane or (5R,9R)-7-(bromomethyl)-5,9-diethyltridecane. **$^1$H-NMR** (300 MHz, $CDCl_3$) δ = 3.44 (d, $^3J$ = 4.3 Hz, 2H, Br-C*H₂*), 1.79 – 1.65 (m, 1H, Br-$CH_2$C*H*), 1.38 – 1.06 (m, 22H, C*H*, C*H₂*), 0.95 – 0.69 (m, 12H, C*H₃*). **$^{13}$C-NMR** (75 MHz, $CDCl_3$) δ = 41.02, 40.99, 40.93, 37.62, 37.57, 37.54, 36.12, 36.07, 35.88, 35.85, 34.49, 34.37, 34.25, 33.27, 33.24, 32.91, 32.89, 28.99, 28.95, 28.71, 28.68, 26.43, 26.39, 25.84, 23.34, 23.26, 14.31, 10.98, 10.93, 10.58. **IR** (neat): ṽ [1/cm] = 2958 s, 2925 s, 2858 m, 1736 w, 1458 m, 1379 m, 1233 w, 759 w.

**4-(4-Ethyl-2-(2-ethylhexyl)octyloxy)benzonitrile**
This compound was prepared according to the general procedure from 328 mg (2.75 mmol, 1 eq) 4-hydroxybenzonitrile, 1,01 g (3.03 mmol, 1.1 eq) 7-bromomethyl-5,9-diethyltridecane, 453 mg (3.28 mmol, 1.2 eq) $K_2CO_3$ and one drop ofaliquat 336 in 15 mL toluene. Column chromatography ($SiO_2$; PE→Tol) afforded 668 mg (1.80 mmol, 65 %) of the desired product as colorless oil. The obtained spectra are a superposition of 4-(((2*r*,4*S*)-4-ethyl-2-((*R*)-2-ethylhexyl)octyl)oxy)benzonitrile, 4-(((2*s*,4*S*)-4-ethyl-2-((*R*)-2-ethylhexyl)octyl)-oxy)benzo-nitrile and 4-(((*S*)-4-ethyl-2-((*S*)-2-ethylhexyl)octyl)oxy)benzonitrile or 4-(((*R*)-4-ethyl-2-((*R*)-2-ethylhexyl)octyl)oxy)benzonitrile. **$^1$H-NMR** (400 MHz, $CDCl_3$) δ = 7.59 – 7.55 (m (AA'XX'), 2H), 6.95 – 6.91 (m (AA'XX'), 2H), 3.85 – 3.83 (m, 2H, OC*H₂*), 1.94 – 1.86 (m, 1H, O$CH_2$C*H*), 1.40 – 1.15 (m, 22H, C*H₂*, C*H*), 0.91 – 0.82 (m, 12H, C*H₃*). **$^{13}$C-NMR** (101 MHz, $CDCl_3$) δ = 162.81, 134.06 (2C), 119.52, 115.31 (2C), 103.68, 72.10, 72.04, 71.94, 36.56, 36.50, 36.47, 36.42, 36.34, 36.32, 33.29, 33.24, 33.20, 33.16, 33.14, 28.94, 28.89, 28.80, 28.77, 26.35, 26.30, 26.16, 23.32, 23.25, 14.30, 10.86, 10.82, 10.70. **IR** (neat): ṽ [1/cm] = 2957 m, 2925 s, 2858 m, 2224 m, 1605 s, 1574 w, 1508 s, 1465 m, 1379 w, 1301 m, 1256 ss, 1171 s, 1111 w, 1014 m, 909 w, 832 s, 734 m. **HRMS-ES(+):** [M+H]+: calc.: 372.3261 found: 372.3257.

**1*H*-5-(4-(4-Ethyl-2-(2-ethylhexyl)octyloxy))tetrazole**
This compound was prepared according to the general procedure from 636 mg (1.71 mmol, 1 eq) 4-(4-Ethyl-2-(2-ethylhexyl)octyloxy)benzonitrile, 448 mg (6.89 mmol, 4.0 eq) sodium

azide and 992 mg (7.21 mmol, 4.2 eq) triethylammonium chloride in 15 mL toluene. Column chromatography ($SiO_2$; Tol:EA:$Et_3N$ = 8:2:1→Tol:EA:HOAc = 8:2:1) afforded 530 mg (1.28 mmol, 75 %) of the product as brown solid. The obtained spectra are a super-position of 4-(((2r,4S)-4-ethyl-2-((R)-2-ethylhexyl)octyl)oxy)benzonitrile, 4-(((2s,4S)-4-ethyl-2-((R)-2-ethylhexyl)octyl)oxy)benzonitrile and 4-(((S)-4-ethyl-2-((S)-2-ethylhexyl)octyl) oxy)benzonitrile or 4-(((R)-4-ethyl-2-((R)-2-ethylhexyl)octyl)oxy)benzonitrile. m.p.: 72-74 °C. **$^1$H-NMR** (300 MHz, $CDCl_3$) δ = 8.06 (d, $^3J$ = 8.8 Hz, 2H), 7.03 (d, $^3J$ = 8.9 Hz, 2H), 3.86 (d, $^3J$ = 5.2 Hz, 2H, O*CH$_2$*), 2.19 – 1.77 (m, 1H, OCH$_2$C*H*), 1.47 – 1.12 (m, 22H, C*H$_2$*, C*H*), 1.00 – 0.73 (m, 12H, C*H$_3$*). **$^{13}$C NMR** (75 MHz, $CDCl_3$) δ = 162.37, 156.26, 129.18 (2C), 115.53 (2C), 115.42, 72.04, 71.99, 71.91, 36.63, 36.57, 36.48, 36.43, 36.33, 33.39, 33.32, 33.26, 33.22, 33.19, 33.16, 28.95, 28.90, 28.81, 28.78, 26.37, 26.31, 26.15, 23.34, 23.27, 14.32, 10.88, 10.83, 10.71. **IR** (neat): ṽ [1/cm] = 2955 s, 2922 s, 2857 s, 2747 w, 2650 w, 1613 s, 1580 w, 1498 s, 1464 s, 1377 m, 1295 m, 1261 s, 1181 s, 1158 m, 1052 m, 1024 s, 838 ss, 750 s, 696 m, 661 s, 620 s, 608 m. **HRMS-ES(+):** [M+H]+: calc.: 415.3431 found: 415.3428.

**3,7,11-Tris(4-((4-ethyl-2-(2-ethylhexyl)octyl)oxy)phenyl)tris([1,2,4]triazolo)[4,3-a:4',3'-c:4'',3''-e][1,3,5]triazine *t*-71**

This compound was prepared according to the general procedure from 252 mg (0.608 mmol, 3.3 eq) 5-(4-((4-ethyl-2-(2-ethylhexyl)octyl)oxy)phenyl)-1*H*-tetrazole, 0.1 mL (0.8 mmol, 4.3 eq) 2,4,6-collidine and 1.90 mL (0.186 mmol, 1.0 eq, 0.0980 M in xylene) of a cyanuric chloride solution in 20 mL xylene. Column chromatography ($SiO_2$ + 2 cm $Al_2O_3$; Tol:EA = 40:1) afforded 196 mg (0.159 mmol, 85 %) of the product as colorless solid and a diasteriomeric mixture. **$^1$H-NMR** (300 MHz, $CDCl_3$) δ = 8.21 – 7.94 (m, 6H, 2/6-H-Ph), 7.17 – 6.90 (m, 6H, 3/5-H-Ph), 3.91 (d, $^3J$ = 5.1 Hz, 6H, OC*H$_2$*), 2.09 – 1.83 (m, 3H, OCH$_2$C*H*), 1.56 – 1.17 (m, 69H, C*H*$_2$, C*H*), 1.00 – 0.74 (m, 36H, $CH_3$). **$^{13}$C-NMR** (75 MHz, $CDCl_3$) δ = 162.45 (4-C-Ph), 151.08 (3,7,11-C), 140.61 (4a,8a,12a-C), 131.96 (2,6-C-Ph), 115.80(1-C-Ph), 114.64 (3,5-C-Ph), 71.86 (O*C*H$_2$), 36.69, 36.64, 36.49, 36.45, 36.35, 33.41, 33.34, 33.29, 33.26, 33.17, 28.97, 28.93, 28.82, 26.41, 26.36, 26.14, 23.37, 23.30, 14.34, 10.91, 10.86, 10.71. **IR** (neat): ṽ [1/cm] = 2959 m, 2923 s, 2857 m, 1736 w, 1593 s, 1484 s, 1464 s, 1378 m, 1294 m, 1251 ss, 1177 s, 1018 m, 829 m, 752 m, 728 m, 695 m, 620 m. **HRMS-ES(+):** [M·]+: calc.: 1234.9822 found: 1234.982.

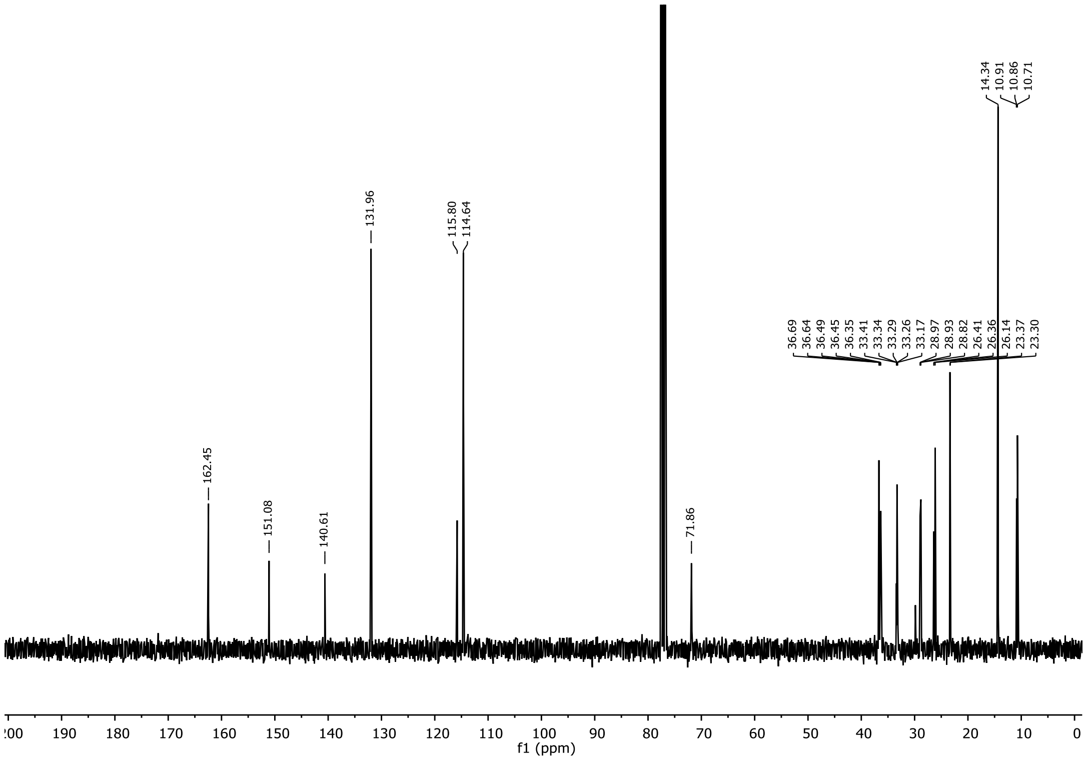

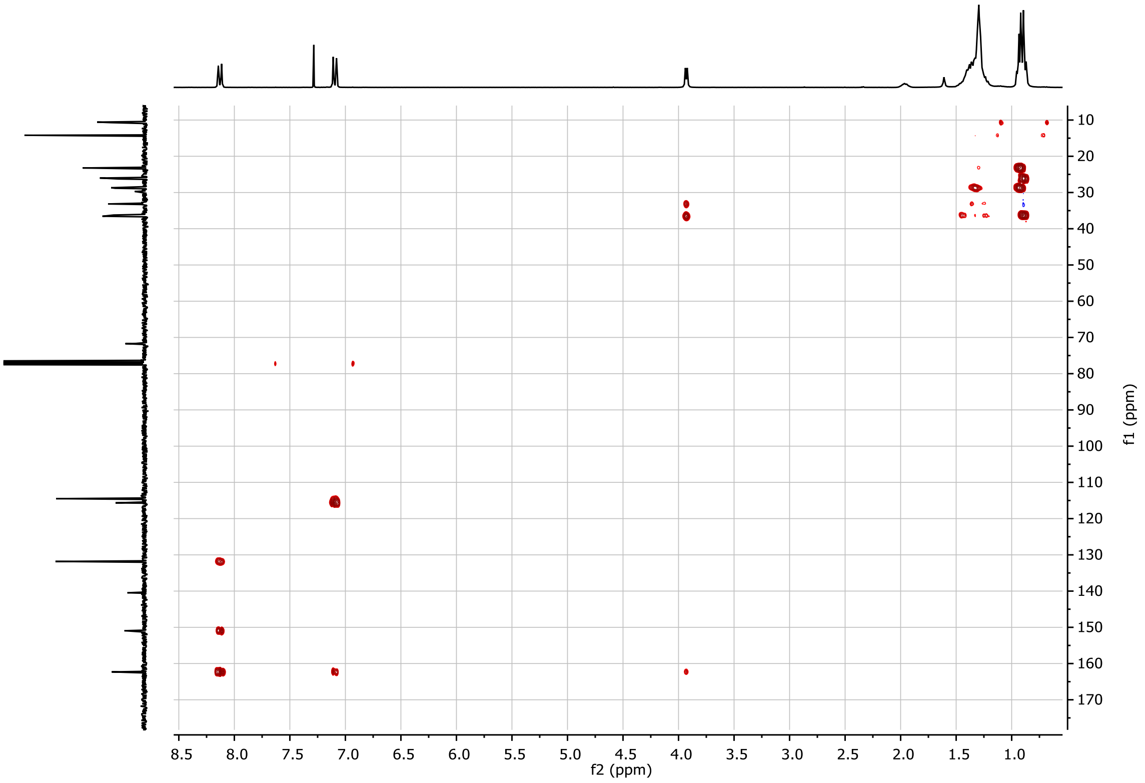


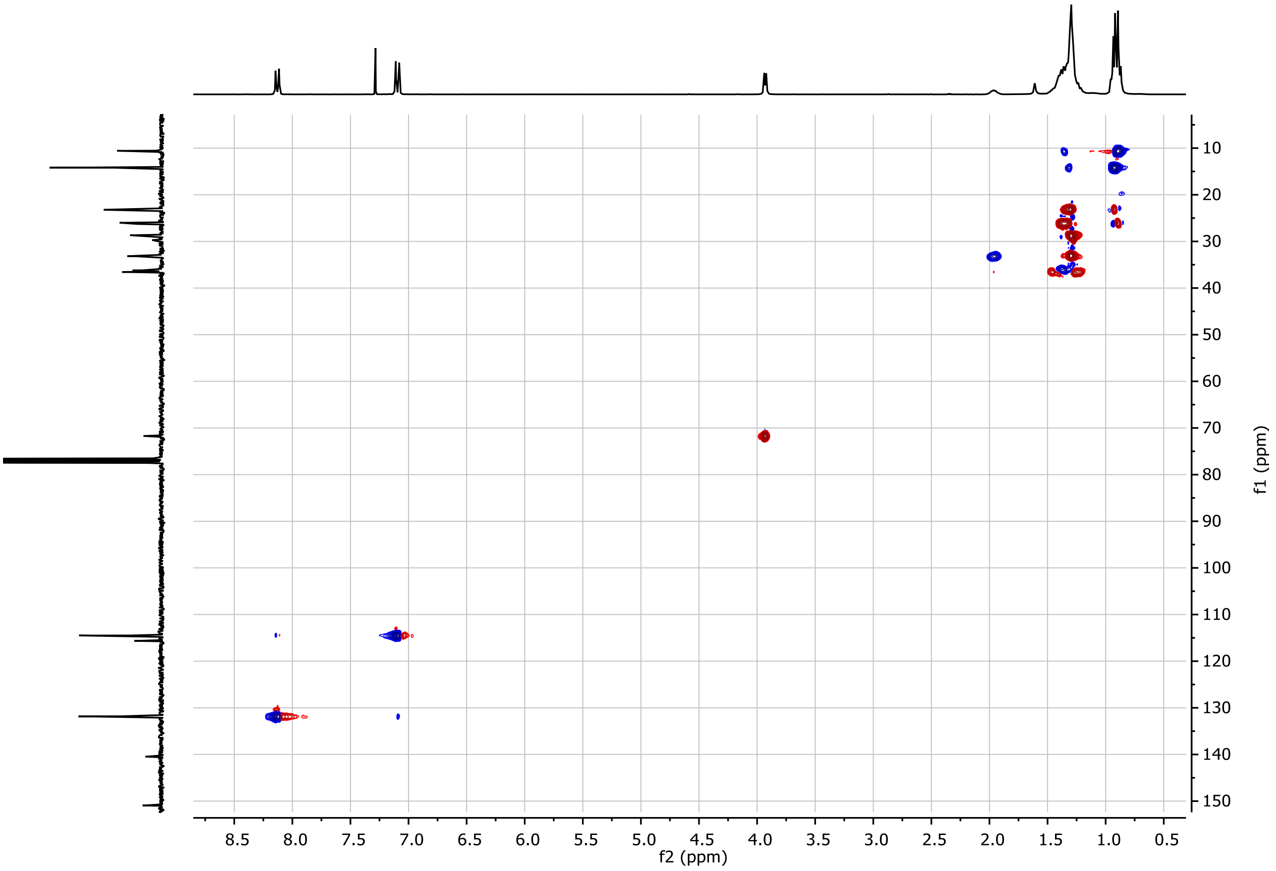


$^{1}$H- and $^{13}$C-NMR-spectra of 3,7,11-Tris(4-((4-ethyl-2-(2-ethylhexyl)octyl)oxy)phenyl)tris([1,2,4]triazolo)[4,3-a:4',3'-c:4'',3''-e][1,3,5]triazine ***t*-71**

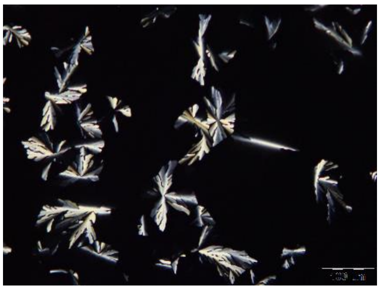 POM of ***t*-71** upon cooling from isotropic to 120 °C.

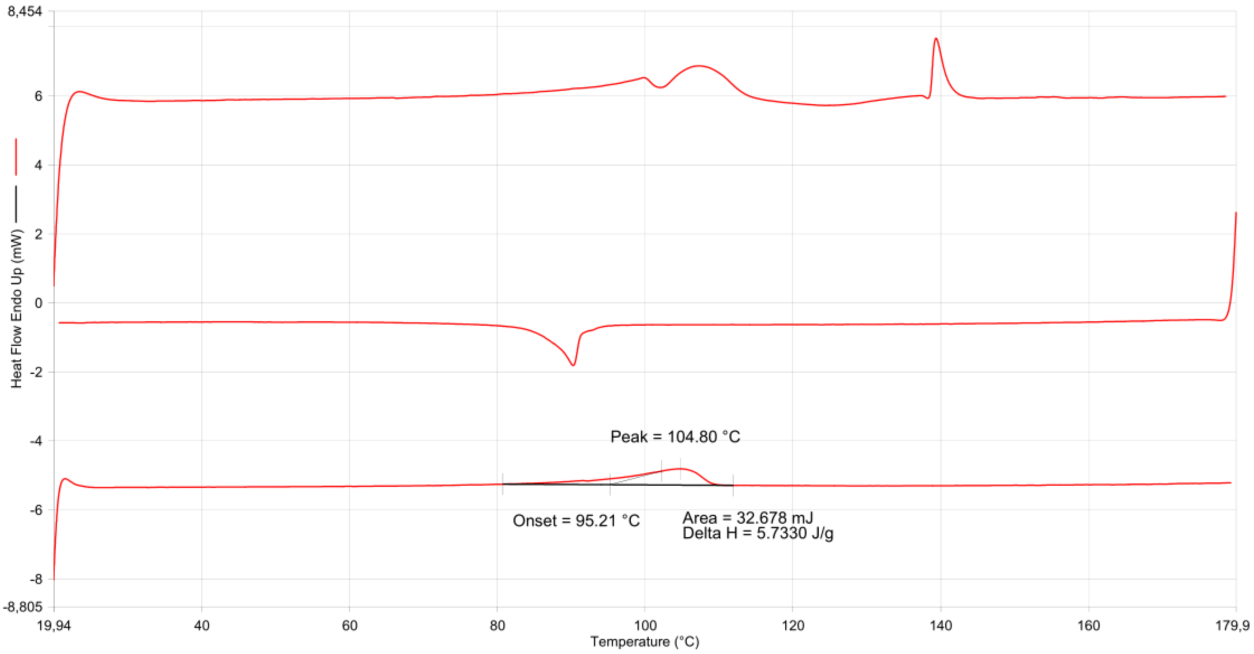


DSC of ***t*-71**

**2,6,10-Tris(4-((4-ethyl-2-(2-ethylhexyl)octyl)oxy)phenyl)tris([1,2,4]triazolo)[1,5-a:1',5'-c:1'',5''-e][1,3,5]triazine *r*-71**

This compound was prepared according to general procedure DL-*r*-TTT from 98 mg (0.079 mmol) 3,7,11-Tris(4-((4-ethyl-2-(2-ethylhexyl)octyl)oxy)phenyl)tris([1,2,4]triazolo)[4,3-a:4',3'-c:4'',3''-e][1,3,5]triazine **71**, 100 mg (0.50 mmol) *p*-bromobenzoic acid and 1.479 g octadecane. The mixture was heated for 39 h at 235 °C and purified via column chromatography. The reaction afforded 17 mg (0.014 mmol, 17 %) of the desired product. **$^1$H-NMR** (400 MHz, $CDCl_3$) δ 8.44 – 8.27 (m (AA'XX'), 6H), 7.09 – 6.94 (m (AA'XX'), 6H), 3.90 (d, $^3J$ = 5.4 Hz, 6H, OC$H_2$), 2.05 – 1.89 (m, 3H, $OCH_2CH$), 1.43 – 1.19 (m, 66H, C$H_2$), 0.97 – 0.74 (m, 36H, C$H_3$). **$^{13}$C-NMR** (101 MHz, $CDCl_3$) δ 165.32, 162.38, 144.64, 129.68, 120.49, 114.88, 71.96, 71.89, 71.80, 36.72, 36.69, 36.65, 36.49, 36.44, 36.37, 36.36, 33.41, 33.35, 33.32, 33.28, 33.19, 33.16, 28.98, 28.93, 28.83, 28.80, 26.41, 26.36, 26.18, 23.37, 23.31, 14.34, 10.90, 10.85, 10.74, 10.73. **IR** (neat): ṽ [1/cm] = 2955 m, 2925 m, 2857 w, 1630 m, 1609 m, 1464 m, 1428 m, 1331 w, 1276 m, 1257 m, 1171 m, 1120 w, 1020 w, 913 w, 841 w, 751 ss, 634 w. **HRMS-ES(+):** [M+H]+: calc.: 1234.9822 found: 1234.9809

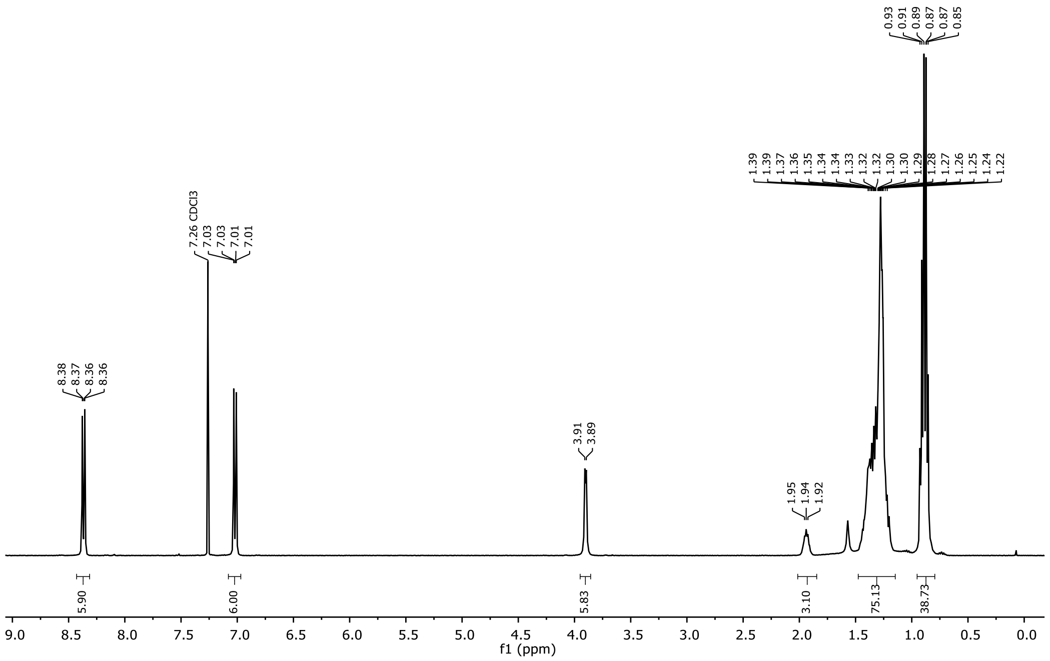


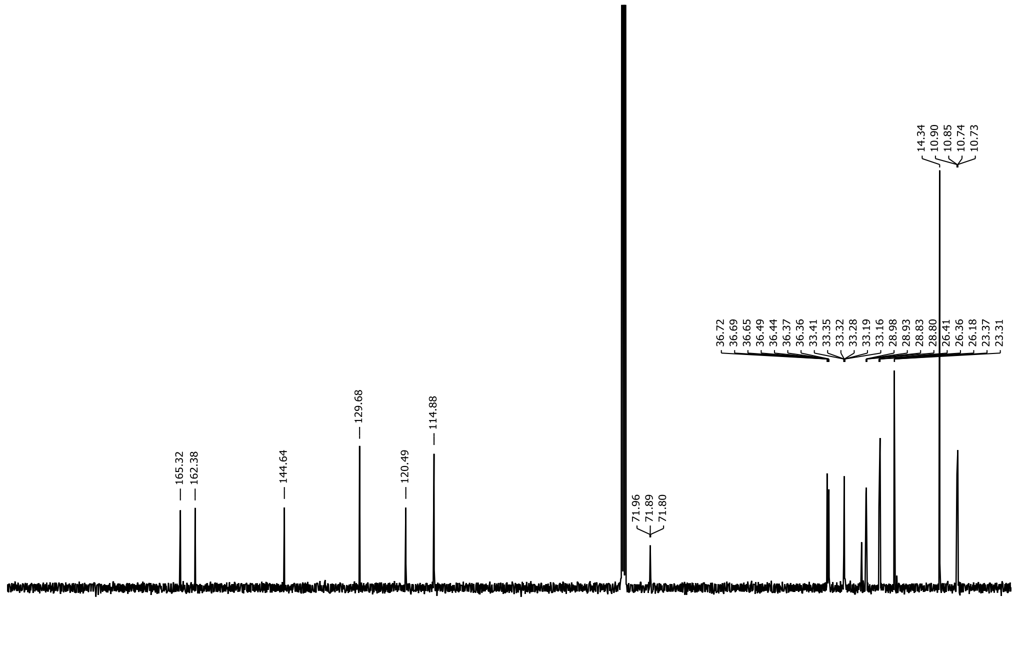


$^{1}$H- and $^{13}$C-NMR spectra of 2,6,10-Tris(4-((4-ethyl-2-(2-ethylhexyl)octyl)oxy)phenyl)tris([1,2,4]triazolo)[1,5-a:1',5'-c:1'',5''-e][1,3,5]triazine ***r*-71**

**3,4-Bis((2-hexyloctyl)oxy)benzonitrile** This compound was prepared according to general procedure DL-Alk from 0.50 g (3.70 mmol, 1 eq) 3,4-dihydroxybenzonitrile, 2.52 g (9.09 mmol, 2.5 eq) 1-bromo-2-hexyloctane and 1.28 g (9.26 mmol, 2.5 eq) $K_2CO_3$ in 40 mL acetonitrile. Column chromatography ($SiO_2$; PE→PE:Tol = 10:1) afforded 1.126 g (2.135 mmol, 58 %) of the desired product as colorless oil. **$^{1}$H-NMR** (400 MHz, $CDCl_3$) δ = 7.22 (dd,

$^3J$ = 8.3 Hz, $^4J$ = 1.9 Hz, 1H, 6-H), 7.05 (d, $^4J$ = 2.0 Hz, 1H, 2-H), 6.85 (d, $^3J$ = 8.3 Hz, 1H, 5-H), 3.88 (d, $^3J$ = 5.5 Hz, 2H, OC*H*$_2$), 3.84 (d, $^3J$ = 5.6 Hz, 2H, OC*H*$_2$), 1.85 – 1.78 (m, 2H, OCH$_2$C*H*), 1.51 – 1.25 (m, 47H, C*H*$_2$), 0.90 – 0.86 (m, 12H, C*H*$_3$). **$^{13}$C-NMR** (101 MHz, CDCl$_3$) δ = 153.54, 149.55, 126.29, 119.71, 115.73, 112.53, 103.38, 72.04, 71.73, 38.16, 38.09, 32.02 (2C), 31.45, 31.42, 29.87, 29.85, 26.99 (2C), 22.84 (2C), 14.26 (2C). **IR** (neat): ṽ [1/cm] = 2954 m, 2924 ss, 2855 s, 2224 w, 1597 m, 1513 s, 1466 s, 1420 m, 1378 w, 1334 m, 1267 ss, 1242 s, 1135 s, 1018 m, 850 m, 807 m, 723 m. **HRMS-ES(+):** [M·]+: calc.: 527.4697 found: 527.4686.

**1*H*-5-(3,4-Bis((2-hexyloctyl)oxy)phenyl)tetrazole**
This compound was prepared according to general procedure from 0.93 g (1.76 mmol, 1 eq) 3,4-bis((2-hexyloctyl)oxy)benzonitrile, 0.46 g (7.08 mmol, 4.0 eq) sodium azide and 1.05 g (7.63 mmol, 4.3 eq) triethylammonium chloride in 12 mL toluene. Additional sodium azide and triethylammonium chloride was added during the reaction. The crude product (940 mg) was used without further purification.

**3,7,11-Tris(3,4-bis((2-hexyloctyl)oxy)phenyl)tris([1,2,4]triazolo)[4,3-a:4',3'-c:4'',3''-e][1,3,5]triazine *t*-72**
This compound was prepared according to the general procedure from 940 mg (crude product) 1*H*-5-(3,4-bis(2-hexyloctyloxy)phenyl)tetrazole, 0.5 mL 2,4,6-collidine and 94 mg (0.51 mmol) cyanuric chloride in 25 mL xylene. Column chromatography (SiO$_2$ + 2 cm Al$_2$O$_3$; Tol:EA = 40:1) afforded 234 mg (0.137 mmol, 27 %) of the product as colorless solid.
$^1$H-NMR (300 MHz, CDCl$_3$) δ = 7.83 (dd, $^3J$ = 8.4 Hz, $^4J$ = 2.1 Hz, 3H, 6-H-Ph), 7.64 (d, $^4J$ = 2.1 Hz, 3H, 2-H-Ph), 7.01 (d, $^3J$ = 8.6 Hz, 3H), 5-H-Ph, 4.06 – 3.85 (m, 12H, OC*H*$_2$CH), 1.96 – 1.68 (m, 6H, OCH$_2$C*H*), 1.54 – 1.20 (m, 120H, C*H*$_2$), 0.95 – 0.82 (m, 36H, C*H*$_3$). $^{13}$C-NMR (75 MHz, CDCl$_3$) δ = 152.78, 151.28, 149.32, 140.64, 123.81, 115.76, 114.76, 112.22, 72.10, 71.67, 38.26, 38.19, 32.06, 31.51, 29.93, 27.03, 22.87, 14.27. IR (neat): ṽ [1/cm] = 2955 s, 2924 ss, 2855 ss, 1579 s, 1525 m, 1490 s, 1465 ss, 1439 m, 1378 w, 1337 m, 1308 m, 1259 ss, 1229 m, 1196 w, 1145 m, 1100 w, 1021 m, 885 w, 860 w, 809 m, 750 w, 720 m. **HRMS-ES(+):** [M+H]+: calc.: 1703.4364 found: 1793.4292.

**Methyl 3,5-di(hexadecyloxy)benzoate and methyl 3-(hexadecyloxy)-5-hydroxy benzoate**
Methy 3,5-dihydroxybenzoate (15.0 g, 89 mmol) , K$_2$CO$_3$ (19.7 g, 143 mmol) were stirred in DMF and after 30 min, bromohexadecane (40 mL, 131 mmol) was added, and the mixture stirred at 100 °C for 22 h, additional K$_2$CO$_3$ (5 g, 36 mmol) was added and heatig continued for 19 h. The mixture was acidulated with hydrochloric acid (2M) angesäuert, precipitated product was isolated and separated on a silica column with toluene:petroleum ether ether = 1:1 → toluene: ethyl acetate = 1:4). gereinigt. Yield: 17.4 g (28 mmol) methyl 3,5-dihexadecyloxybenzoate, m. p. 77 °C (petroleum ether) and 7.1 g (18 mmol) methyl 3-hexadecyloxy-5-hydroxybenzoate, m.p. 98 °C (chloroform).

**Methyl 3,5-di(hexadecyloxy)benzoate IR:** (ATR) ṽ = 2917, 2849, 1722, 1602, 1470, 1442, 1391, 1324, 1236, 1165, 1054, 996, 897, 856, 762, 741, 717 cm$^{-1}$; **$^1$H-NMR:** (300 MHz, CDCl$_3$) *δ* = 7.15 (d, $^4J$ =2.3 Hz, 2H, H-2, H-6), 6.63 (t, $^4J$ = 2.3 Hz, 1H, H-4), 3.96 (t, $^3J$ =6.5 Hz, 4H, OC*H*$_2$), 3.89 (s, 3H, OC*H*$_3$), 1.83 – 1.71 (m, 4H, C*H*$_2$), 1.51 – 1.17 (m, 56H, C*H*$_2$), 0.93 – 0.82 (m, 6H, C*H*$_3$) ppm.

**Methyl 3-(hexadecyloxy)-5-hydroxy benzoate IR:** (ATR) ṽ = 3429, 2918, 2848, 1697, 1612, 1502, 1469, 1440, 1393, 1356, 1316, 1258, 1152, 1047, 1001, 862, 764, 719 cm$^{-1}$; **$^1$H-NMR**: (300 MHz, CDCl$_3$) *δ* = 7.15 (dd, $^4J$ = 2.3 Hz, $^4J$ = 1.3 Hz, 1H, H-6), 7.12 (dd, $^4J$ = 2.3 Hz, $^4J$ = 1.3 Hz, 1H, H-2), 6.60 (t, $^4J$ = 2.3 Hz, 1H, H-4), 5.46 (s, 1H, O*H*), 3.95 (t, $^3J$ = 6.5 Hz, 2H, OC*H*$_2$), 3.90 (s, 3H, OC*H*$_3$), 1.77 (p, $^4J$ = 6.6 Hz, 2H, C*H*$_2$), 1.50 – 1.15 (m, 29H, C*H*$_2$), 0.93 – 0.84 (m, 3H, C*H*$_3$) ppm.

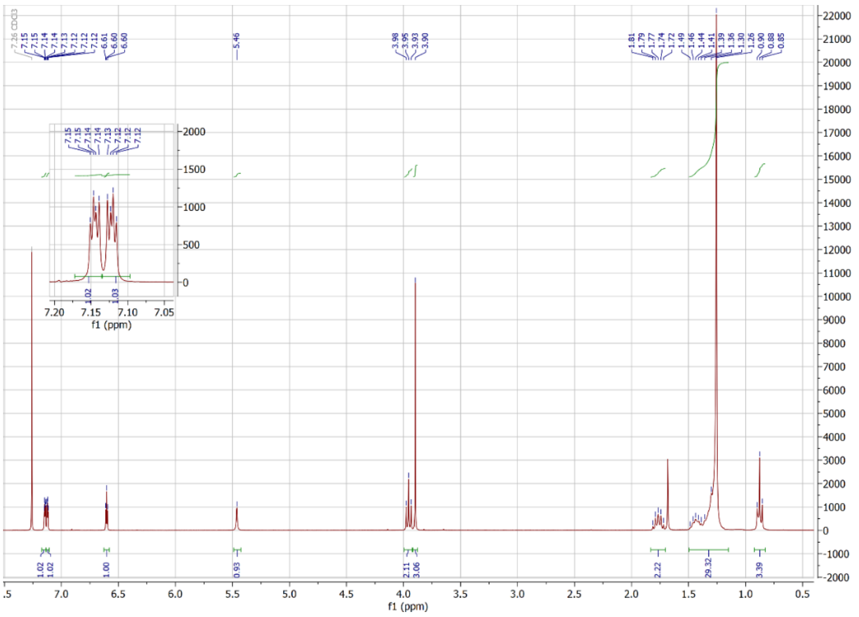


$^{1}$H-NMR (300 MHz, $CDCl_3$) methyl 3-(hexadecyloxy)-5-hydroxybenzoate

**Methyl 3-(butyloxy)-5-hexadecyloxy benzoate**: 3-(hexadecyloxy)-5-hydroxy benzoate (2.35 g, 6.0 mmol ) in dioxane (50 mL) , 1-bromobutane (0.9 mL, 8.4 mmol) and $K_2CO_3$ (1.29 g, 9.3 mmol) together with 18-crown-6 (93 mg, 0.4 mmol) were refluxed for 4 days. The mixture was concentrated, acidulated (HCl, 2n) and the precipitate filtered and recrystallized from ethanol to yield 2.41 g (5.4 mmol, 90%9 of a colorless solid with m.p. = 58 °C. **IR**: (ATR) ṽ = 2917, 2848, 1721, 1602, 1470, 1443, 1390, 1323, 1235, 1163, 1115, 1054, 999, 857, 762, 717 $cm^{-1}$; **$^{1}$H-NMR**: (300 MHz, $CDCl_3$) $\delta$ = 7.16 (d, $^{4}J$ = 2.3 Hz, 2H, H-2, H-6), 6.63 (t, $^{4}J$ = 2.3 Hz, 1H, H-4), 4.02 – 3.92 (m, 4H, OC*H*$_2$), 3.89 (s, 3H, OC*H*$_3$), 1.84 – 1.70 (m, 4H), 1.63 – 1.19 (m, 31H), 0.97 (t, $^{3}J$ = 7.4 Hz, 3H, $C_4$-C*H*$_3$), 0.92 – 0.84 (m, 3H, $C_{16}$– C*H*$_3$) ppm

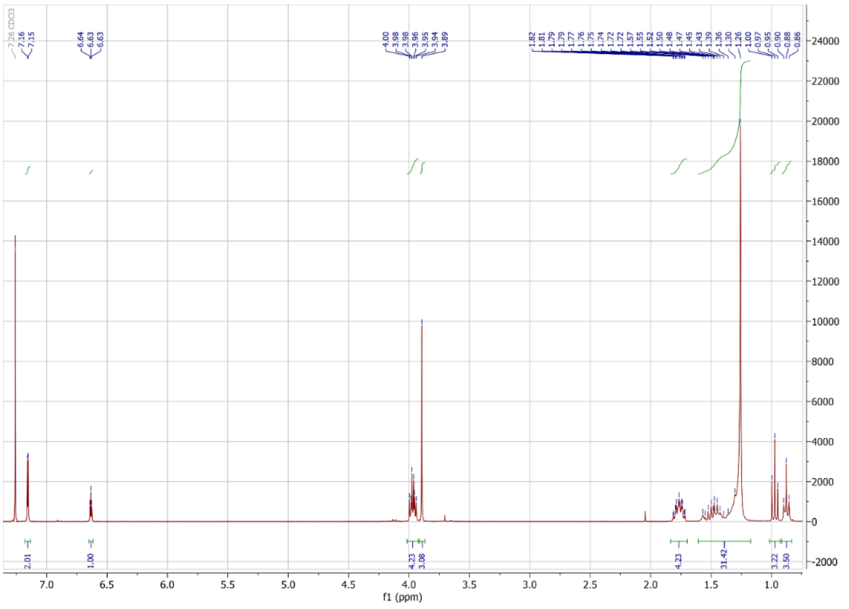


. $^{1}$H-NMR (300 MHz, $CDCl_3$) methyl 3-butoxy-5-(hexadecyloxy)benzoate

**3-Butoxy-5-(hexadecyloxy)benzoic acid** was prepared according to GP3 to yield 1.94 g (83%) of the crude amide with m.p. = 67 °C. **IR**: (ATR) ṽ = 2916, 2848, 1693, 1597, 1469, 1445, 1420, 1389, 1347, 1300, 1271, 1171, 1059, 933, 876, 845, 765, 732, 718 $cm^{-1}$; **$^1$H-NMR**: (300 MHz, $CDCl_3$) $\delta$ = 7.22 (d, $^4J$ = 2.3 Hz, 2H), 6.69 (t, $^4J$ = 2.3 Hz, 1H), 4.03 – 3.94 (m, 4H), 1.85 – 1.71 (m, 4H), 1.59 – 1.16 (m, 30H), 0.98 (t, $^3J$ = 7.4 Hz, 3H, $C_4$–$CH_3$), 0.92 – 0.84 (m, 3H, $C_{16}$–$CH_3$) ppm.

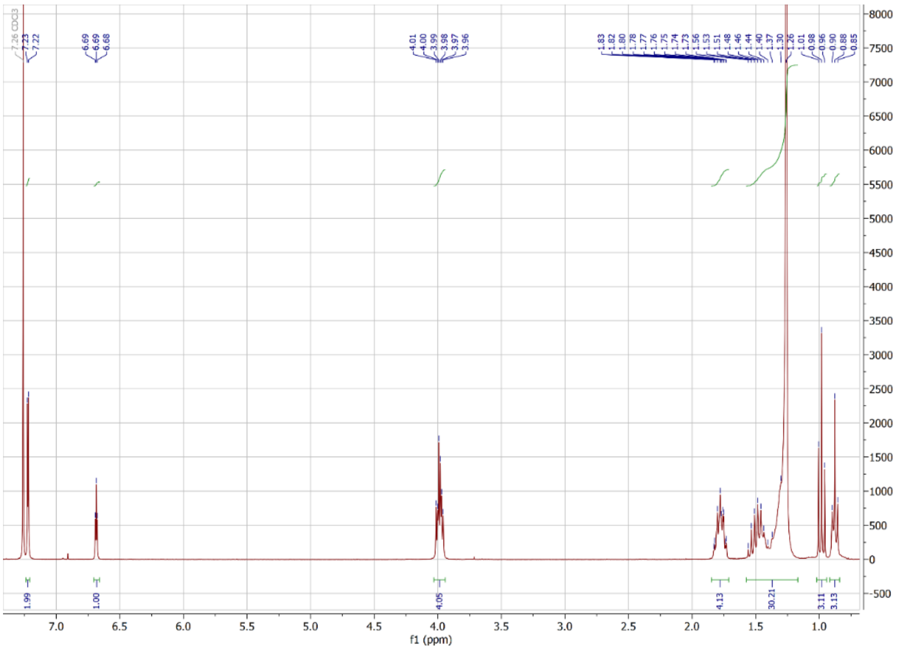


$^1$H-NMR (300 MHz, $CDCl_3$) 3-butoxy-5-(hexadecyloxy)benzoic acid.

**3-Butoxy-5-(hexadecyloxy)benzoic amide** was prepared according to GP4 to yield 1.61 g (83%) of an off-white solid with m.p. = 79 °C after recrystallization from ethanol. **IR**: (ATR) ṽ = 3360, 3179, 2952, 2916, 2849, 1649, 1597, 1467, 1439, 1414, 1327, 1301, 1170, 1120, 1065, 935, 851, 794, 720, 677 $cm^{-1}$; **$^1$H-NMR**: (300 MHz, $CDCl_3$) $\delta$ = 6.90 (d, $^4J$ = 2.2 Hz, 2H, H-2, H-6), 6.59 (t, $^4J$ = 2.2 Hz, 1H, H-4), 5.77 (d, br, 2H, $NH_2$), 4.02 – 3.92 (m, 4H, $OCH_2$), 1.82 – 1.70 (m, 4H, $CH_2$), 1.55 – 1.15 (m, 32H, $CH_2$), 0.97 (t, $^3J$ = 7.4 Hz, 3H, C4-$CH_3$), 0.91 – 0.83 (m, 3H, C16-$CH_3$) ppm; **$^{13}$C-NMR**: (75 MHz, $CDCl_3$ + 1 drop DMSO-$d_6$) $\delta$ = 169.36 (C-7), 160.32 (C-3, C-5), 135.53 (C-1), 105.81, 104.80, 68.32 ($OCH_2$), 68.00 ($OCH_2$), 31.93, 31.23, 29.70, 29.67, 29.61, 29.59, 29.37, 29.20, 26.02, 22.70, 19.22 ($CH_2$), 14.15 ($CH_2$), 13.85 ($CH_3$) ppm.

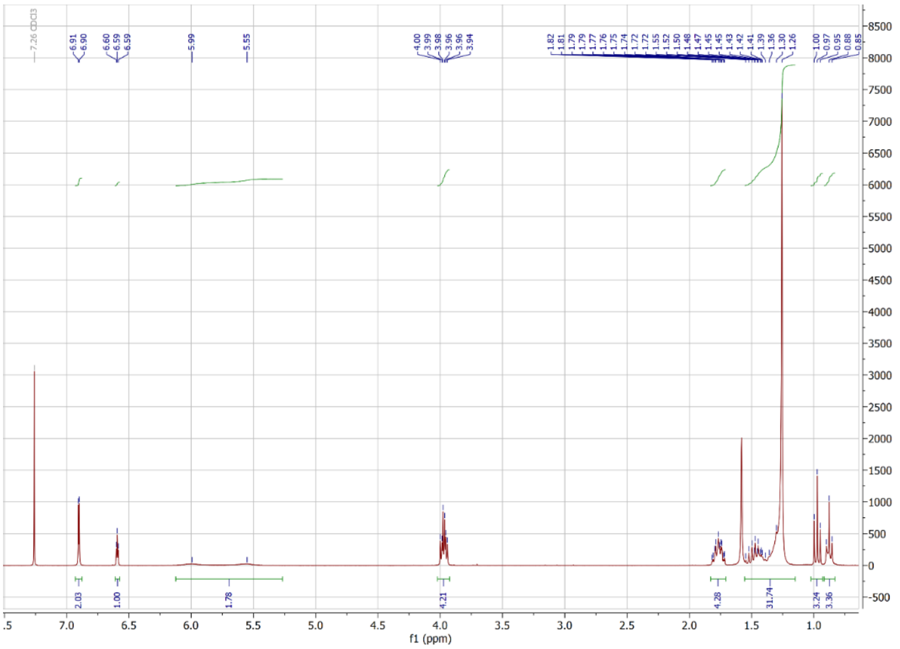

$^{1}H$-NMR (300 MHz, $CDCl_3$) 3-butoxy-5-(hexadecyloxy)benzoic amide

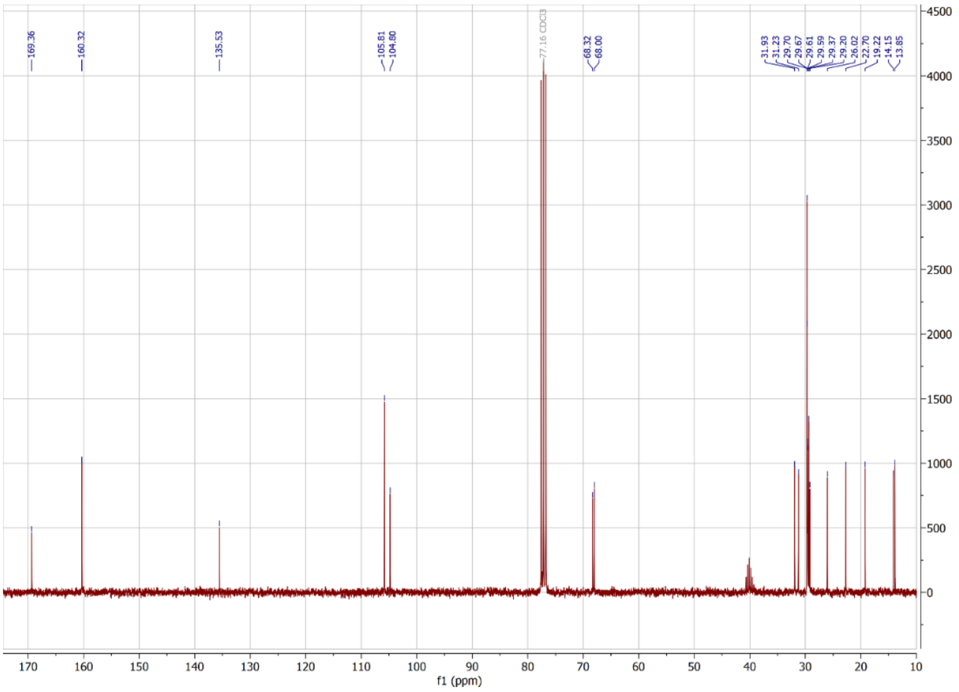

$^{13}C$-NMR (75 MHz, $CDCl_3$ +1 drop DMSO-$d_6$) 3-butoxy-5-(hexadecyloxy)benzoic amide

**5-(3-Butoxy-5-(hexadecyloxy)phenyl)-2*H*-tetrazole** was prepared according to GP6 to yield 287 mg (58%) of a colorless solid with mp. 48-49 °C after recrystallization from ethanol. Chromatography on silica with toluol:ethyl acetate = 2:1 + 3% triethyl amine → toluol:ethyl acetate = 2:1 + 1% acetic acid. **IR**: (ATR) ṽ = 2913, 2848, 1603, 1560, 1470, 1421, 1390, 1366, 1290, 1251, 1174, 1049, 851, 833, 747, 717 $cm^{-1}$; **$^1$H-NMR**:(300 MHz, $CDCl_3$) *δ* = 10.86 (s, 1H, N*H*), 7.22 (d, $^4J$ = 2.2 Hz, 2H, H-2, H-6), 6.57 (t, $^4J$ = 2.2 Hz, 1H, H-4), 3.98 – 3.86 (m, 4H, OC$H_2$), 1.79 – 1.65 (m, 4H, C$H_2$), 1.50 – 1.13 (m, 32H, C$H_2$), 0.96 – 0.83 (m, 6H, C$H_3$) ppm; **$^{13}$C-NMR**: (75 MHz, $CDCl_3$) *δ* = 161.12 (C-3, C-5, ph), 157.18 (C-5, tet), 125.14 (C-1), 105.62, 105.34, 68.60 ($OCH_2$), 68.27 ($OCH_2$), 32.07, 31.30, 29.86, 29.84, 29.82, 29.79, 29.74, 29.56, 29.52, 29.30, 26.13, 22.84, 19.31 ($CH_2$), 14.27 ($CH_3$), 13.94 ($CH_3$) ppm; **FD-MS**: m/z (%): 458.8 (6), 459.8 (21), 460.8 (2) $[M]^{•+}$; 918.3 (100), 919.3 (73), 920.2 (21) $[M_2]^{•+}$.

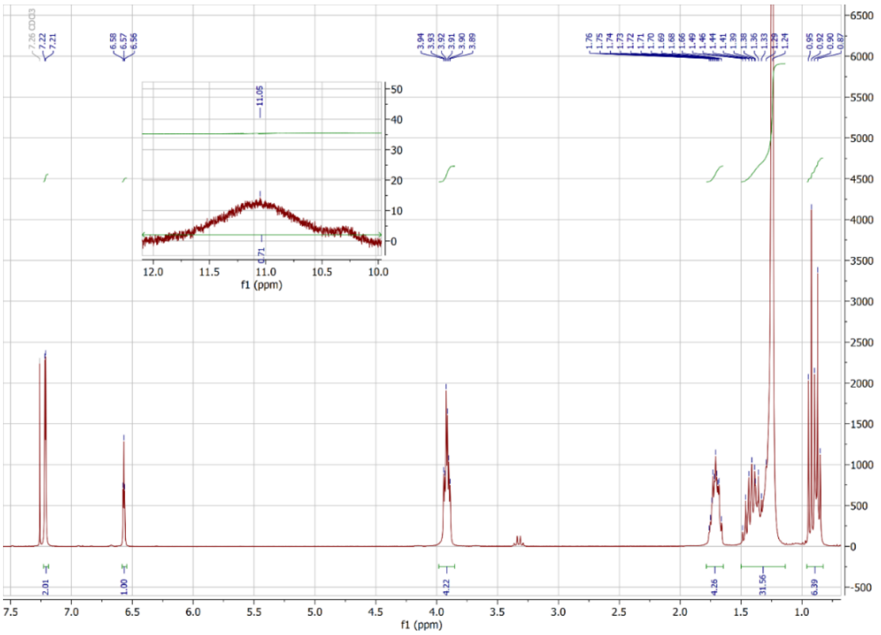

**$^1$H-NMR (300 MHz, $CDCl_3$) 5-(3-butoxy-5-(hexadecyloxy)phenyl)-2*H*-tetrazole**

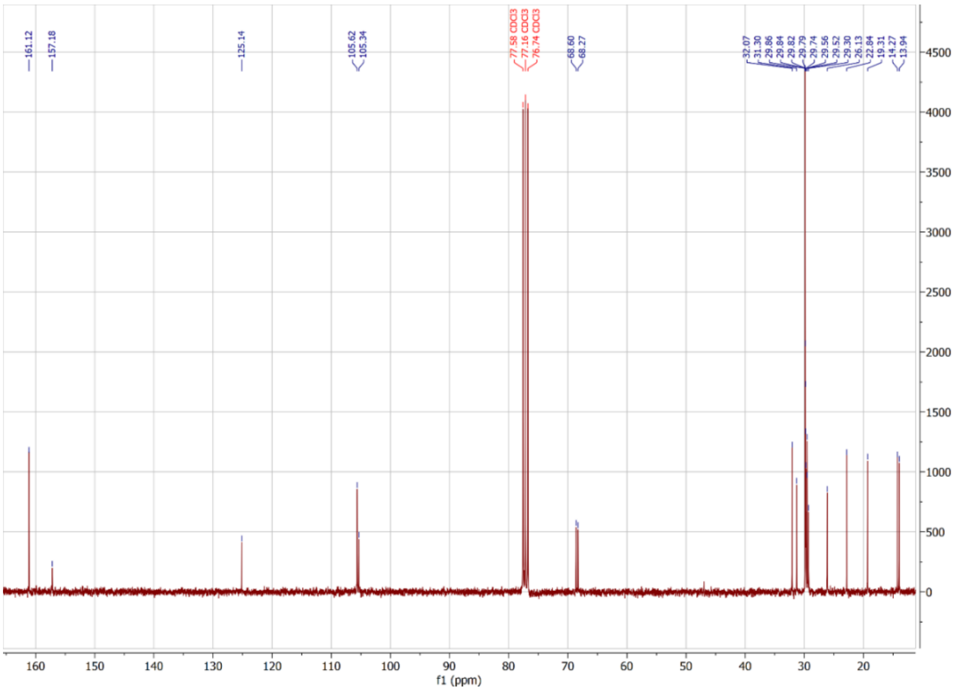

$^{13}$C-NMR (75 MHz, $CDCl_3$) 5-(3-butoxy-5-(hexadecyloxy)phenyl)-2H-tetrazole

**3,7,11-Tris(3-butoxy-5-(hexadecyloxy)phenyl)tris([1,2,4]triazolo)[4,3-a:4',3'-c:4'',3''-e][1,3,5]triazine *t*-73** was prepared according to GP7 to yield, after chromatography ($SiO_2$ + 5 cm $Al_2O_3$; toluene:petroleum ether = 40:1) 233 mg (0.17 mmol, 84%) of a off-white solid with m.p. = 47 °C (DSC). **IR**: (ATR) ṽ = 2921, 2851, 1595, 1573, 1523, 1463, 1436, 1389, 1337, 1270, 1165, 1056, 841, 772, 717 $cm^{-1}$; **$^{1}$H-NMR**: (300 MHz, $CDCl_3$) $\delta$ =7.25 (d, $^{4}J$ = 2.1 Hz, 2H, H-2, H-6, ph), 6.70 (t, $^{4}J$ = 2.2 Hz, 1H, H-4, ph), 4.08 – 3.97 (m, 4H, O$CH_2$), 1.87 – 1.71 (m, 4H, $CH_2$), 1.61 – 1.12 (m, 33H, $CH_2$), 0.98 (t, $^{4}J$ = 7.4 Hz, 3H, C4-$CH_3$), 0.92 – 0.84 (m, 3H, C16-$CH_3$) ppm; **$^{13}$C-NMR**: (75 MHz, $CDCl_3$) $\delta$ = 160.33 (C-3, C-5, ph), 151.07 (C-5, trl), 140.64(C-3, trl), 125.25 (C-1, ph), 108.61 (C-4, ph), 105.58 (C-2, C-6, ph), 68.59, 68.26 ($OCH_2$), 32.07, 31.36, 29.85, 29.83, 29.81, 29.78, 29.74, 29.57, 29.51, 29.34, 26.19, 22.84, 19.39 ($CH_2$), 14.27, 14.00 ($CH_3$) ppm; **FD-MS**: m/z (%): 1368.0 (9), 1368.7 (100) $[M]^{\bullet+}$.

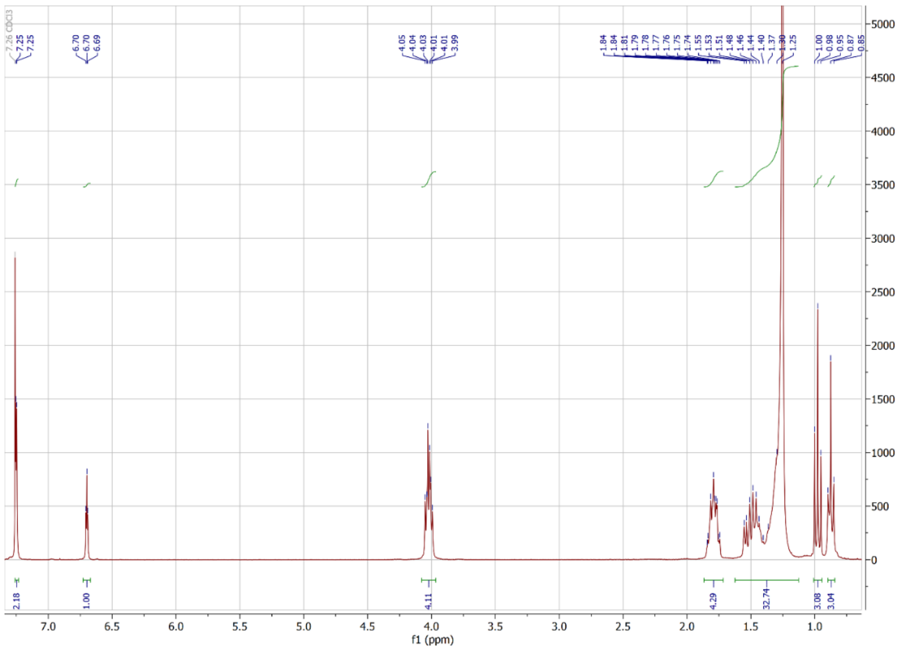


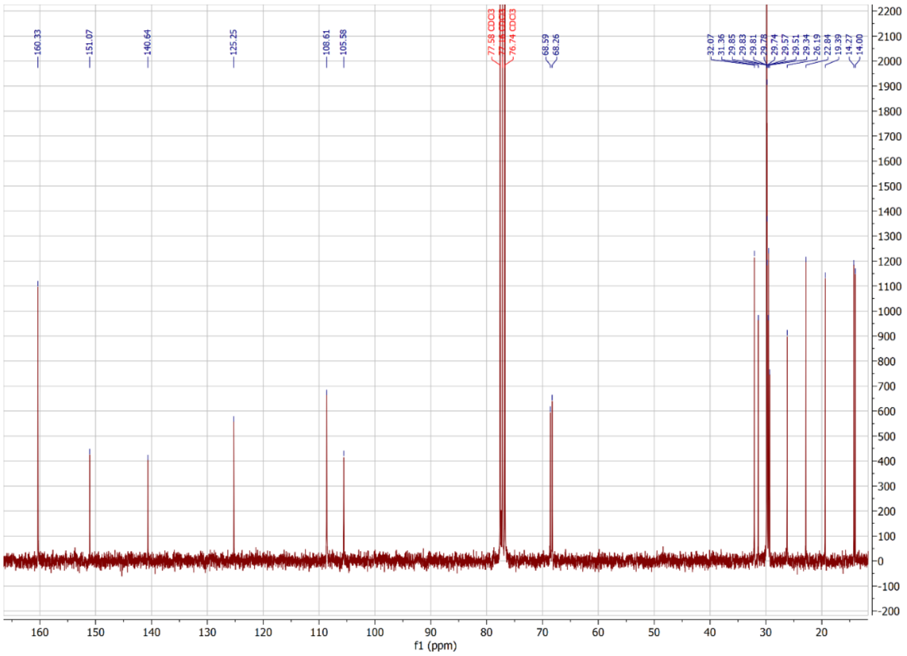


$^{1}$H-NMR (300 MHz, $CDCl_3$) and $^{13}$C-NMR (75 MHz, $CDCl_3$) 3,7,11-Tris(3-butoxy-5-(hexadecyloxy)phenyl)tris([1,2,4]triazolo)[4,3-a:4',3'-c:4'',3''-e][1,3,5]triazine

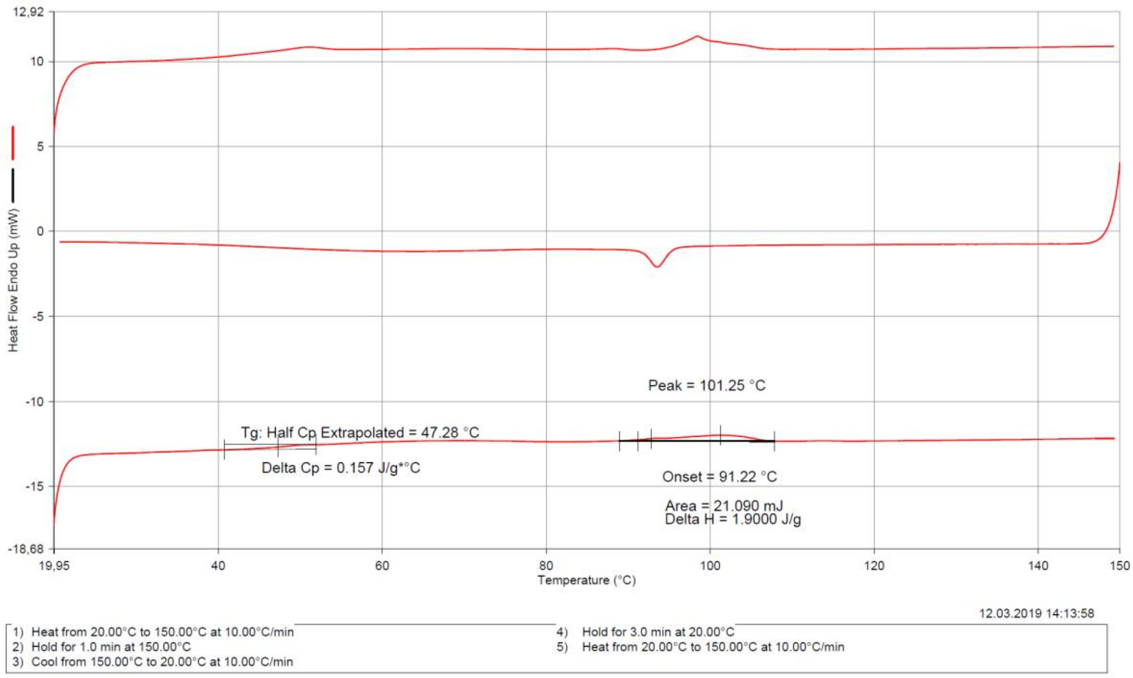


DSC of ***t*-73**

**2,6,10-Tris(3-butoxy-5-(hexadecyloxy)phenyl)tris([1,2,4]triazolo)[1,5-a:1',5'-c:1'',5''-e][1,3,5]triazine *r*-73** 3,7,11-Tris(3-butoxy-5-(hexadecyloxy)phenyl)tris([1,2,4]triazolo)-[4,3-a:4',3'-c:4'',3''-e][1,3,5]triazine, (98.6 mg, 0.07 mmol), 4-bromobenzoic acid (27.3 mg, 0.14 mmol) and octadecane (1.557 g) were heated under nitrogen for 24 h to 235 °C. The cooled mixture was dissolved in petroleum ether and purified via chromatography ($SiO_2$+ 1 cm $Al_2O_3$; petroleum ether $\rightarrow$ petroleum ether:toluene = 1:1 $\rightarrow$ toluene:ethyl acetate = 30:1) to yield 33.6 mg (0.02 mmol, 34%) of a colorless solid with 99 °C (DSC). **IR**:(ATR) ṽ = 2921, 2851, 1629, 1605, 1505, 1462, 1434, 1390, 1331, 1278, 1163, 1054, 750, 722 $cm^{-1}$; **$^1$H-NMR**: (300 MHz, $CDCl_3$) $\delta$ = 7.53 (d, $^4J$ = 2.0 Hz, 2H, H-2, H-6, ph), 6.57 (t, $^4J$ = 2.2 Hz, 1H, H-4, ph), 4.10 – 3.94 (m, 4H, O$CH_2$), 1.80 – 1.67 (m, 4H, $CH_2$), 1.58 – 1.17 (m, 33H, $CH_2$), 0.98 (t, $^3J$ = 7.3 Hz, 3H, C4-$CH_3$), 0.91 – 0.82 (m, 3H, C16-$CH_3$) ppm; **$^{13}$C-NMR**: (75 MHz, $CDCl_3$) $\delta$ = 164.76 (C-5, trl), 160.64 (C-3, C-5, ph), 144.15 (C-3, trl), 129.74(C-1, ph), 105.69 (C-2, C-6, ph), 104.97 (C-4, ph), 68.63 ($OCH_2$), 32.09, 31.39, 29.91, 29.84, 29.82, 29.66, 29.54, 29.37, 26.15, 22.85, 19.32, ($CH_2$), 14.27, 14.03 ($CH_3$) ppm; **APCI-MS**: m/z (%): 1367.06 (100), 1368.06 (93), 1369.06 (38).

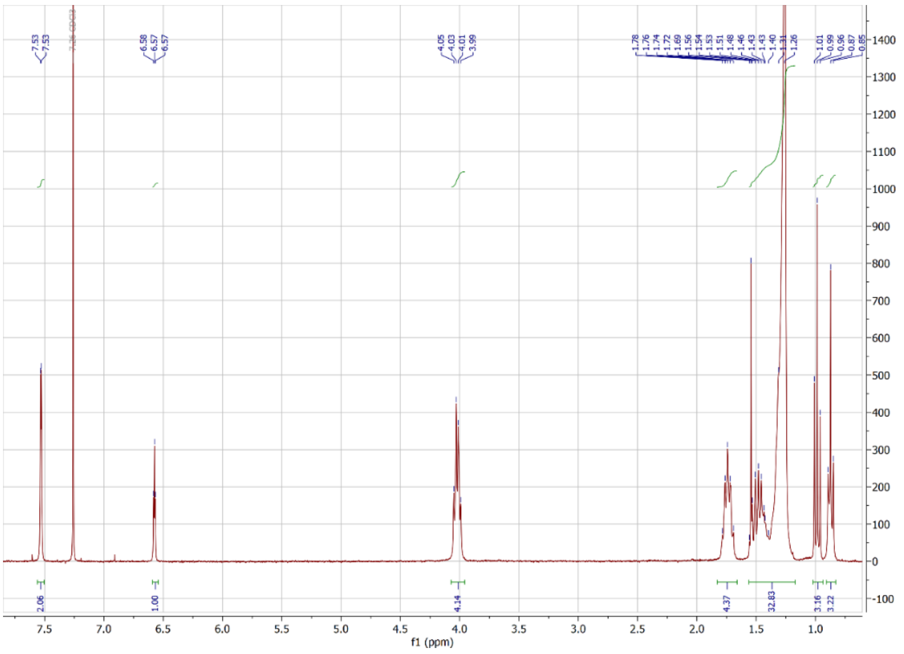


$^{1}$H-NMR (300 MHz, CDCl3) 2,6,10-Tris(3-butoxy-5-(hexadecyloxy)phenyl)tris([1,2,4]triazolo)[1,5-a:1',5'-c:1'',5''-e][1,3,5]triazine

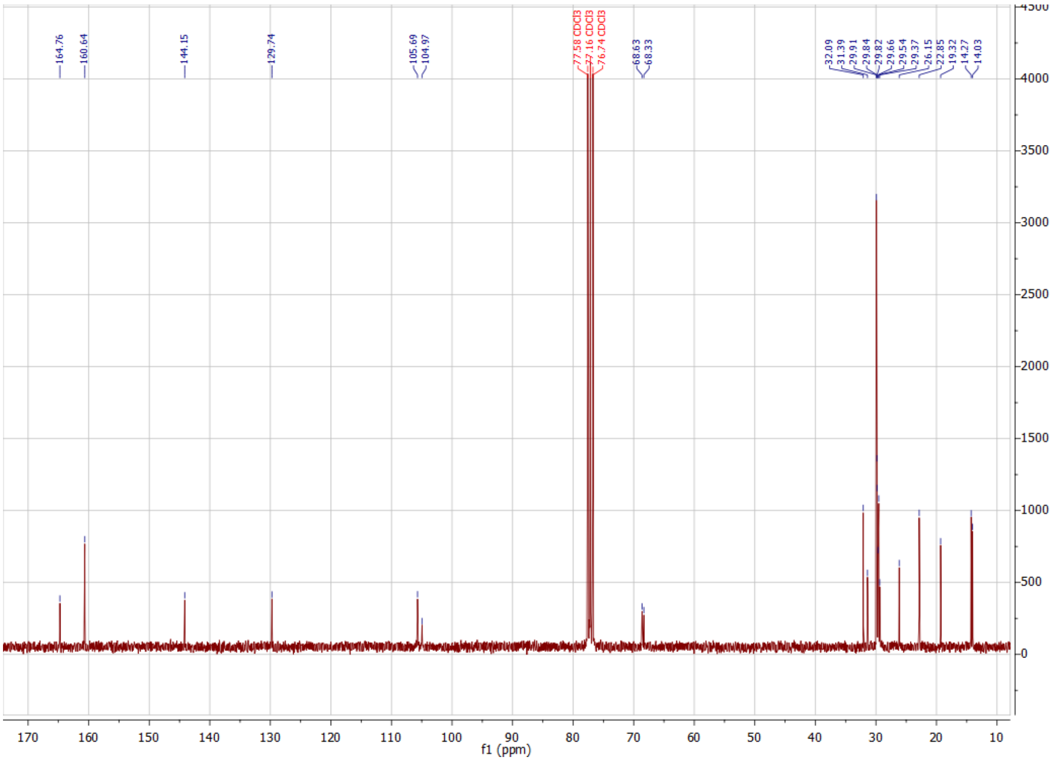


$^{13}$C-NMR (75 MHz, $CDCl_3$) 2,6,10-Tris(3-butoxy-5-(hexadecyloxy)phenyl)tris([1,2,4]triazolo)[1,5-a:1',5'-c:1'',5''-e][1,3,5]triazine

**Ethyl 3-hydroxy-5-hexyloxybenzoate**

Ethyl 3,5 dihydroxybenzoate (60 mmol, 10.93 g), potash (66 mmol, 8.29 g), 18-crown-6 (3 mmol, 0.79 g), and 1-bromohexane (30 mmol, 4.95 g) in 300 mL acetone were refluexed 1for 12 h. gelöst, mit Kaliumcarbonat sowie 18-Krone-6 versetzt und fünf Minuten bei Raumtemperatur gerührt. Additional 1-bromohexane (30 mmol, 4.95 g) was added, refluxing continued for 48 h, and the acetone removed in vacuo. The residue was dissolved in water,

extracted with ethyl acetate and the organic layers combined, washedm, dried and the solvent evaporated. Chromatography on a short solica column with light petroleum/toluene 1/1 yielded methyl dihexyloxybenzoate, elution with ethyl acetate/toluene (1/6) gave 5.82 g (36%) of the monoalkylated ester as yellowish solid
**$^1$H NMR** (300 MHz, $CDCl_3$): δ = 7.16 (dd, $^4J$ = 2.3, $^4J$ = 1.3 Hz, 1H, 6-H, Ar-H), 7.12 – 7.09 (m, 1H, 2-H, Ar-H), 6.59 (t, $J$ = 2.3 Hz, 1H, 4-H, Ar-H), 4.35 (q, $^3J$ = 7.1 Hz, 2H,-$COOCH_2$), 3.96 (t, $J$ = 6.5 Hz, 2H, $OCH_2$), 1.84 – 1.70 (m, 2H, $OCH_2$-$CH_2$), 1.43 – 1.28 (m, $CH_2$), 0.94 – 0.83 (m, $CH_3$).

**Ethyl 3-tetradecyloxy-5-hexyloxybenzoate**
Ethyl 3-hydroxy-5-hexyloxybenzoate (22 mmol, 5.82 g), (26 mmol, 4.33 g) 1-bromotetradecane, and potash (33 mmol, 4.53 g) in 15 mL DMF were stirred for 24 h at 80 °C. Additional (1.08 g) 1-bromotetradecane, and potash (1.5 g) were added, stirring at 80 °C continued for 24 h. The mixture was acidulated with 2 N HCL, extracted with light petroleum and the combined organic layers were washed with 2N NaOH, water, and dried to yield 8.36 g of a colorless oil. **$^1$H-NMR** (300 MHz, $CDCl_3$): δ = 7.16 (d, $^4J$ = 2.3 Hz, 2H,2-H, 6-H, Ar-H), 6.63 (t, $^4J$ = 2.4 Hz, 1H, 4-H, Ar-H), 4.35 (q, $^3J$ = 7.1 Hz, 2H, -$COOCH_2$), 3.97 (t, $^3J$ = 6.4 Hz, 4H, -$OCH_2$), 1.85 – 1.69 (m, 4H, -$OCH_2$-$CH_2$), 1.52 – 1.23 (m, 24H, $COOCH_2$-$CH_3$, -$CH_2$,), 0.98 – 0.80 (m, 6H, -$CH_2$-$CH_3$). **$^{13}$C NMR** (75 MHz, $CDCl_3$): δ = 166.7 (-COO-$CH_2$), 160.3 (3-C,5-C, Ar-C), 132.3 (1-C, Ar-C), 107.8 (2-C,6-C, Ar-CH), 106.4 (4-C, Ar-CH), 68.4 (-$OCH_2$), 61.2 (-$COOCH_2$-$CH_3$), 32.1, 31.7, 29.8, 29.8, 29.8, 29.7, 29.5, 29.3, 29.3, 26.2, 25.8, 22.8, 22.8 (-$CH_2$), 14.5 (-$COOCH_2$-$CH_3$), 14.3, 14.2(-$CH_2$-$CH_3$). **IR** (ATR): $\tilde{\nu}$ [$cm^{-1}$] = 2952 w, 2923 s, 2853 m, 1720 m, 1595 m, 1446 m, 1388 w, 1367 w, 1346 w, 1322 m, 1297 m, 1227 m, 1164 s, 1103 m, 1051 m, 860 w, 844 w, 767 m, 723 w, 677 w. **HR-ESI:** calcd. for $C_{29}H_{50}O_4$ + $Na^+$: 485.3607; found: 485.3595.

**3-Tetradecyloxy-5-hexyloxybenzoic acid**
Ethyl 3-tetradecyloxy-5-hexyloxybenzoate (17 mmol, 8.00 g) in 70 mL 2-propanol and 35 mL of 10 % KOH in water were refluxed for 2 h. Evaporation oft he alcohol, acidulation with 2 N HCL to pH = 2 and extraction with ether yielded 7.39 g (98 %) of a colorless soid with m.p. = 52 - 54 °C. **$^1$H-NMR** (300 MHz, $CDCl_3$): δ = 7.23 (d, $^4J$ = 2.4 Hz, 2H, 2-H, 6-H, Ar-H), 6.69 (t, $^4J$ = 2.3 Hz, 1H, 4-H, Ar-H), 3.98 (t, $^3J$ = 6.5 Hz, 4H, -$OCH_2$), 1.84 – 1.73 (m, 4H, 9-H, 15-H, -$OCH_2$-$CH_2$), 1.53 – 1.20 (m, 23H, -$CH_2$), 0.95 – 0.85 (m, 6H, -$CH_2$-$CH_3$). **$^{13}$C NMR** (75 MHz, $CDCl_3$): δ = 171.8 (-COOH), 160.3 (3-C,5-C, Ar-C), 131.0 (1-C, Ar-C), 108.3 (2-C,6-C, Ar-CH), 107.6 (4-C, Ar-CH), 68.5 (-$OCH_2$), 32.1, 31.7, 29.8, 29.8, 29.8, 29.7, 29.5, 29.3, 29.3, 26.2, 25.8, 25.5, 22.9, 22.8 (-$CH_2$), 14.3, 14.2 (-$CH_2$-$CH_3$). **IR** (ATR): $\tilde{\nu}$ [$cm^{-1}$] = 2916 s, 2849 m, 1692 s, 1609 m, 1445 m, 1421 m, 1394 m, 1317 m, 1269 m, 1167 s, 1060 m, 938 w, 855 m, 762 m, 736 m, 669 w. **HR-ESI**: calcd. for $C_{27}H_{46}O_4$ + $Na^+$: 457.3294; found: 457.3302.

**3-Tetradecyloxy-5-hexyloxybenzamide**
3-Tetradecyloxy-5-hexyloxybenzoic acid (17 mmol, 7.39 g) in 50 mL toluene and 10.1 g (85 mmol, 6.2 mL) thionyl chloride were refluxed for 12 h, concentrated to 7 mL and added dropwise to heavily stirred, ice-cold concentrated ammonia. The solid was isolated by suction filtration, recrystallized from ethanol to yield 6.99 g (95% d.Th.) colorless solid with m.p. = 74 - 76 °C.
**$^1$H-NMR** (300 MHz, $CDCl_3$): δ = 6.90 (d, $^4J$ = 2.2 Hz, 2H, 2-H, 6-H, Ar-H), 6.59 (t, $^4J$ = 2.3 Hz, 1H, 4-H, Ar-H), 6.05 (d, $^2J$ = 28.9 Hz, 2H, -$CONH_2$), 3.96 (t, $^3J$ = 6.5 Hz, 4H, -$OCH_2$), 1.84 – 1.68 (m , 4H, $OCH_2$-$CH_2$), 1.49 – 1.22 (m, 21H, 10-H – 12-H, 16-H – 26-H, -$CH_2$), 0.93 – 0.84 (m, 6H, 13-H, 27-H, -$CH_2$-$CH_3$). **$^{13}$C NMR** (75 MHz, $CDCl_3$): δ = 169.6 (-$CONH_2$), 160.5 (3-C, 5-C), 135.4 (1-C), 105.8 (2-C,6-C), 105.0 (4-C), 68.5 ( -$OCH_2$), 32.1, 31.7, 29.8, 29.8, 29.7, 29.7, 29.6, 29.5, 29.3, 29.3, 26.1, 25.8, 22.8, 22.7 ( -$CH_2$), 14.3, 14.2 (-$CH_2$-$CH_3$). **IR** (ATR):$\tilde{\nu}$ [$cm^{-1}$] = 3414 m, 3196 m, 2914 ss, 2849 s, 1653 s, 1599 s, 1471 m, 1439 m, 1405 m, 1378 m, 1167 ss, 1101 w, 1066 w, 866 w, 851 m, 774 m, 716 m, 676 m, 655 m. **HR-ESI**: calcd. for $C_{27}H_{47}N_1O_3$ + $Na^+$: 456.3454; found: 456.3440.

**5-(3-Tetradecyloxy-5-hexyloxyphenyl)-*2H*-tetrazole**

The tetrazole was prepared from (30.4 mmol, 1.98 g) $NaN_3$, 3-tetradecyloxy-5-hexyloxybenzamide (4.6 mmol, 2.00 g), and $SiCl_4$ (10.2 mmol, 1.72 g, 1.2 mL) in 25 mL acetonitrile according to the general procedure. After stirring for 44 h, aqueou work-up, extraction with dichloromethane and recrystallization from ethanol yielded a colorless solid (38 %, 810 mg) with m.p. = 57 - 61 °C. **$^1$H-NMR** (300 MHz, $CDCl_3$): δ = 7.23 (d, $^4J$ = 2.2 Hz, 2H, 2-H, 6-H, Ar-H), 6.58 (t, $J$ = 2.2 Hz, 1H, 4-H, Ar-H), 3.94 (t, $^3J$ = 6.6 Hz, 4H, -$OCH_2$), 1.73 (p, $^3J$ = 6.7 Hz, 4H, $OCH_2$-$CH_2$), 1.52 – 1.21 (m, 28H, $CH_2$), 0.93 – 0.81 (m, 6H, -$CH_3$). **$^{13}$C NMR** (75 MHz, $CDCl_3$): δ = 161.1 (3-C, 5-C, Ar-C), 156.8 (7-C, Tet.-C), 124.8 (1-C, Ar-C), 105.7 (2-C, 6-C, Ar-CH), 105.5 (4-C, Ar-CH), 68.6 (-$OCH_2$), 32.1, 31.7, 29.9, 29.8, 29.8, 29.7, 29.6, 29.5, 29.3, 29.2, 26.1, 25.8, 22.8, 22.7 (-$CH_2$), 14.3, 14.2 (-$CH_3$). **IR** (ATR): $\tilde{\nu}$ [$cm^{-1}$] = 2918 s, 2851 m, 1605 m, 1566 w, 1459 m, 1390 w, 1168 ss, 1057 m, 852 w, 683 w. **HR-ESI**: calcd. for $C_{27}H_{47}N_4O_2$ + $Na^+$: 459.3699; found: 459.3683.

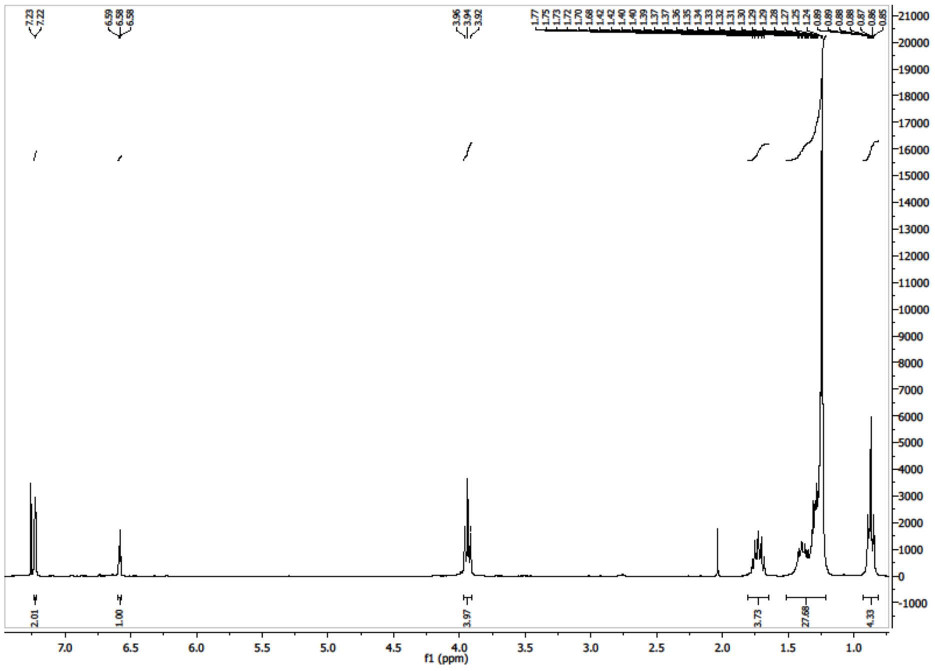


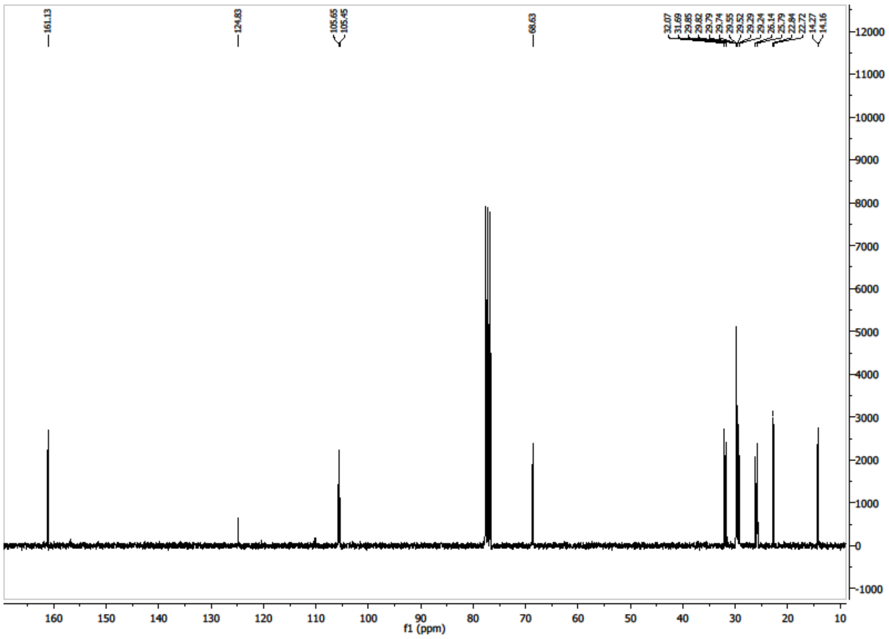


$^1$H- and $^{13}$C-NMR of 5-(3-Tetradecyloxy-5-hexyloxyphenyl)-*2H*-tetrazole

**3,7,11-Tris(3-(hexyloxy)-5-(tetradecyloxy)phenyl)tris([1,2,4]triazolo)[4,3-a:4',3'-c:4'',3''-e][1,3,5]triazine *t*-66**

5-(3-Tetradecyloxy-5-hexyloxyphenyl)-*2H*-tetrazole (1.30 mmol, 600 m), cyanuric chloride (0.38 mmol, 71 mg) in 50 mL xylenes were mixed with 0.5 mL pyridine. Within 2 h, the temperature of the stirred mixture was raised to 110 °C. After 24 h, aqueous work-up yielded 900 mg of a brown solid. Chromatography on silica gel with toluene/ethyl acetate (20/1) yielded a brownish, waxy solid (240 mg, 36%) with m.p. = 120 °C (DSC). Chromatography on a silica column with head of basic alumina using toluenen as eluent yielded 182 mg (35 %) of a coloeless wax with m.p. = 132 (DSC, 2$^{nd}$ heating). **$^{1}$H-NMR**(300 MHz, $CDCl_3$): δ = 7.25 (d, $^{4}J$ = 2.3 Hz, 6H, 2-H, 6-H, Ar-H), 6.70 (t, $^{4}J$ = 2.2 Hz, 3H, 4-H, Ar-H), 4.10 – 3.96 (m, 12H, -$OCH_2$), 1.86 – 1.74 (m, 12H, -$OCH_2$-C$H_2$), 1.52 – 1.21 (m, 61H, -$CH_2$), 0.97 – 0.82 (m, 18H, -$CH_3$). **$^{13}$C NMR** (75 MHz, $CDCl_3$): δ = 160.3 (5-C, 3-C, Ar-C), 151.1 (5-C, TTT-C), 140.6 (3-C, TTT-C), 125.2 (1-C, Ar-C), 108.6 (6-C, 2-C, Ar-CH), 105.6 (3-C, Ar-CH), 68.6 (-$OCH_2$), 32.1, 31.7, 29.8, 29.8, 29.7, 29.6, 29.5, 29.3, 29.3, 26.2, 25.9, 22.8, 22.8 (-$CH_2$), 14.3, 14.2 (-$CH_3$). **IR** (ATR): $\tilde{\nu}$ [$cm^{-1}$] = 2923 ss, 2853 s, 1595 m, 1522 w, 1463 m, 1436 m, 1391 m, 1338 m, 1261 s, 1167 s, 1097 s, 1056 s, 858 m, 803 s, 716 m. **HR-ESI**: calcd. for $C_{84}H_{136}N_9O_6$ + $H^+$: 1367.0614; found: 1367.0579.

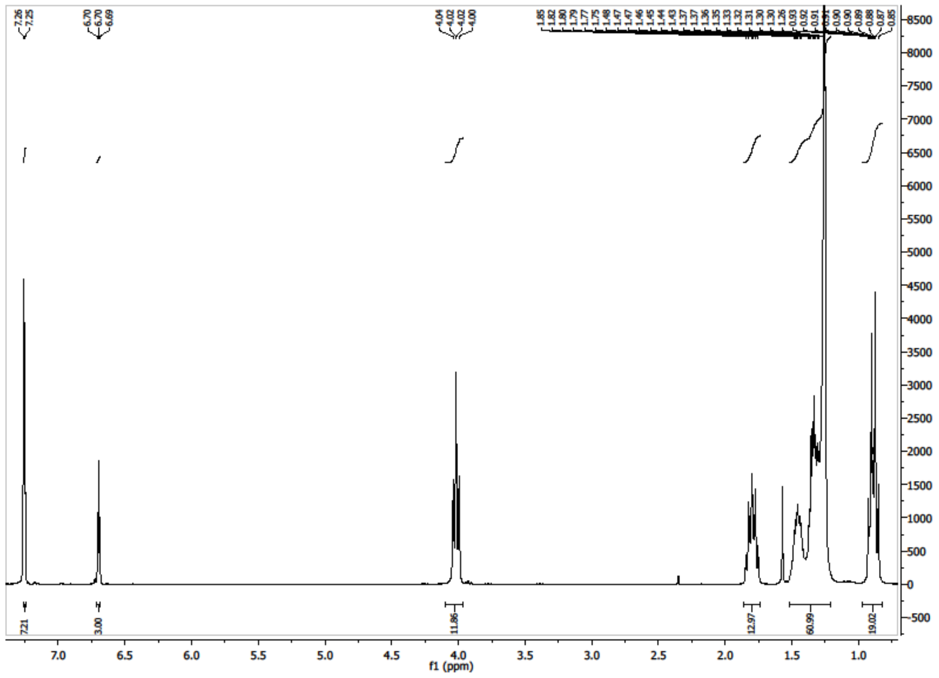

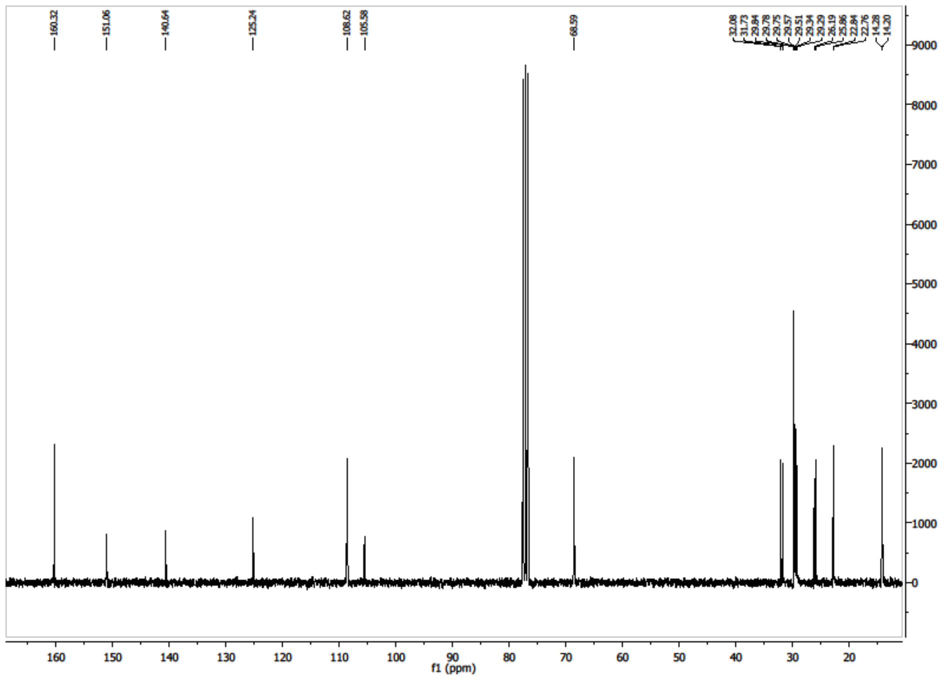


$^{1}$H- and $^{13}$C-NMR of ***t*-66**

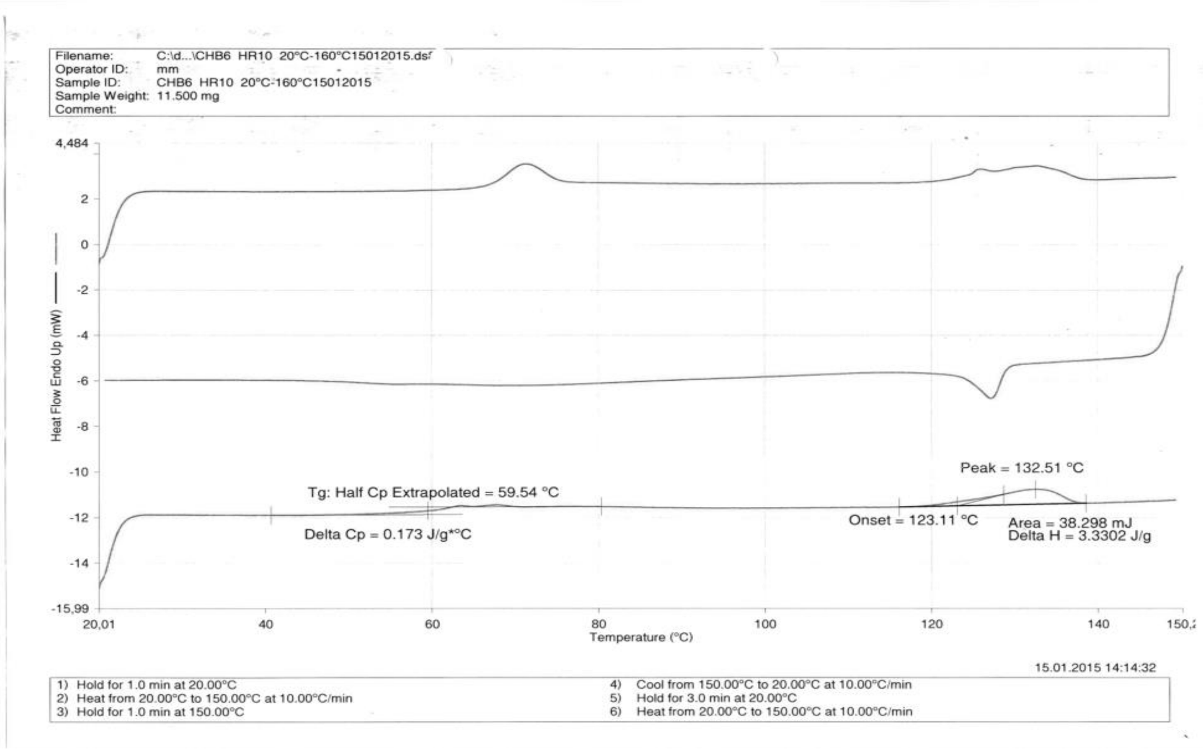


$^{1}$H- and $^{13}$C-NMR and DSC of 3,7,11-Tris(3-(hexyloxy)-5-(tetradecyloxy)phenyl)tris([1,2,4] triazolo)[4,3-a:4',3'-c:4'',3''-e][1,3,5]triazine ***t*-66**

**2,6,10-Tris-(3-hexyloxy-5-tetradecyloxyphenyl)tris([1,2,4]triazolo)[1,5-a:1',5'-c:1'',5''-e][1,3,5]triazine *r*-66**

This compound was prepared according to general procedure DL-*r*-TTT from 56.8 mg (0.0415 mmol) 3,7,11-tris-(3-hexyloxy-5-tetradecyloxyphenyl)([1,2,4]triazolo)[4,3-a:4',3'-c:4'',3''-e][1,3,5]triazine **130**, 58.2 mg (0.289 mmol) *p*-bromobenzoic acid and 2.025 g octadecane. The mixture was heated for 24.5 h at 235 °C and purified via column chromatography. $^{1}$H-NMR (300 MHz, $CDCl_3$) δ = 7.45 (d, $^{4}J$ = 2.2 Hz, 6H), 6.46 (t, $^{4}J$ = 2.3 Hz, 3H), 3.94 (t, $^{3}J$ = 6.7 Hz, 12H), 1.80 – 1.53 (m, 12H), 1.49 – 1.17 (m, 84H), 1.00 – 0.76 (m, 18H). $^{13}$C-NMR (75 MHz, $CDCl_3$) δ = 164.86, 160.66 (3,5-C-Ph), 144.21, 129.74, 105.74 (2,6-C-Ph), 105.07 (4-C-Ph), 68.62, 32.10, 31.78, 29.90 – 29.81 (6C), 29.66, 29.55, 29.39, 29.32, 26.17, 25.82, 22.86, 22.80, 14.29, 14.24. IR (neat): ṽ [1/cm] = 2923 ss, 2853 s, 1738 m, 1631 s, 1608 s, 1505 m, 1465 s, 1438 m, 1378 m, 1334 m, 1254 m, 1169 s, 1057 m, 852 w, 750 m, 680 m, 630 w, 614 w. **HRMS-ES(+):** [M+H]+: calc.: 1367.0608 found: 1367.0591.

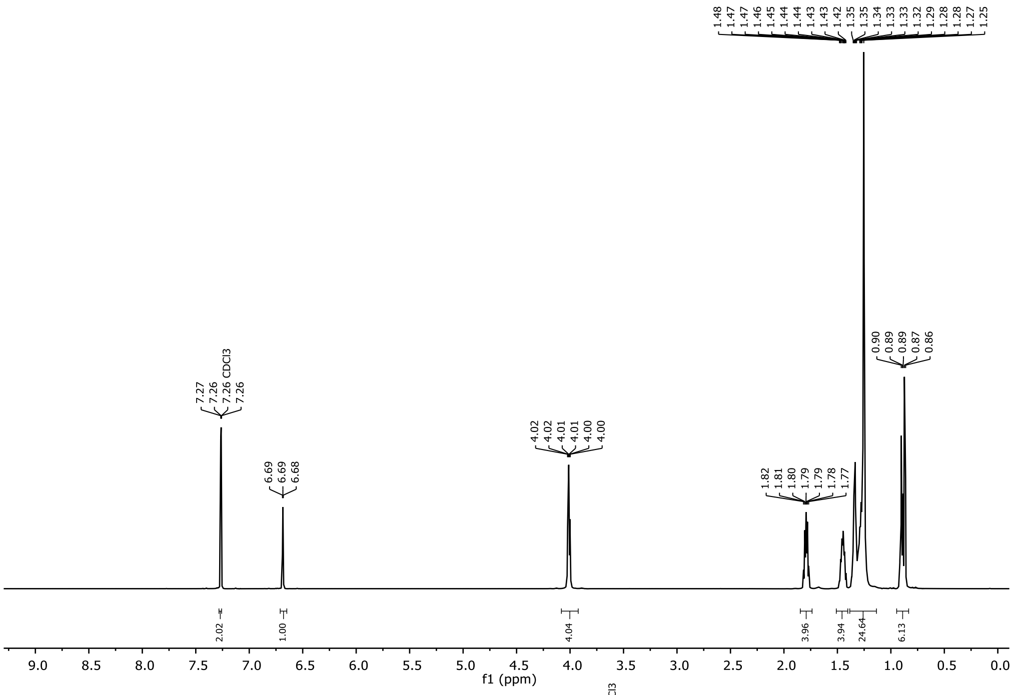


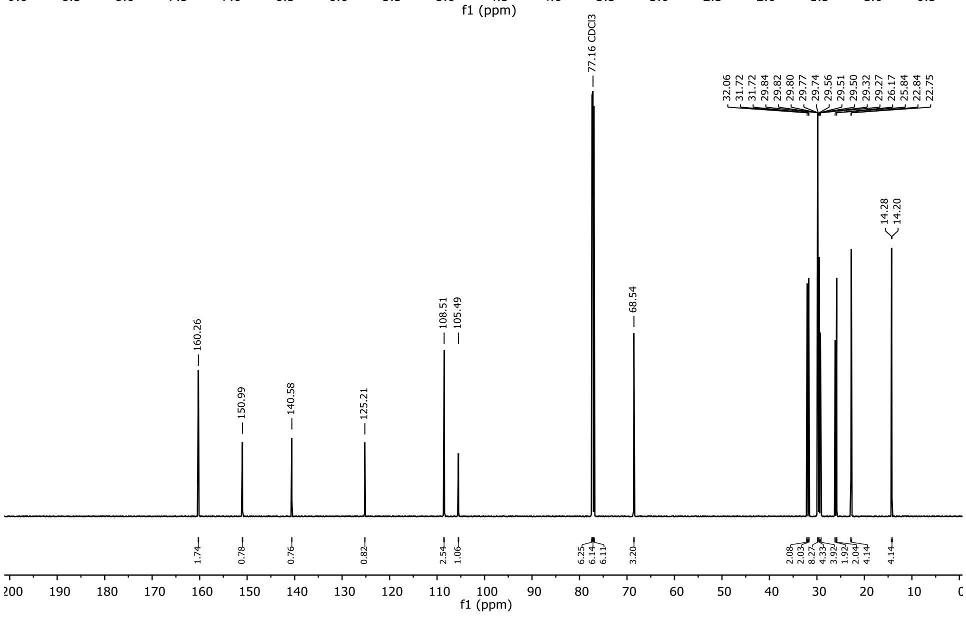


$^{1}$H- and $^{13}$C-NMR spectra of 3,7,11-Tris(3-hexyloxy-5-tetradecyloxy)phenyl)tris([1,2,4]triazolo)[4,3-a:4',3'-c:4'',3''-e][1,3,5]triazine ***r*-66**

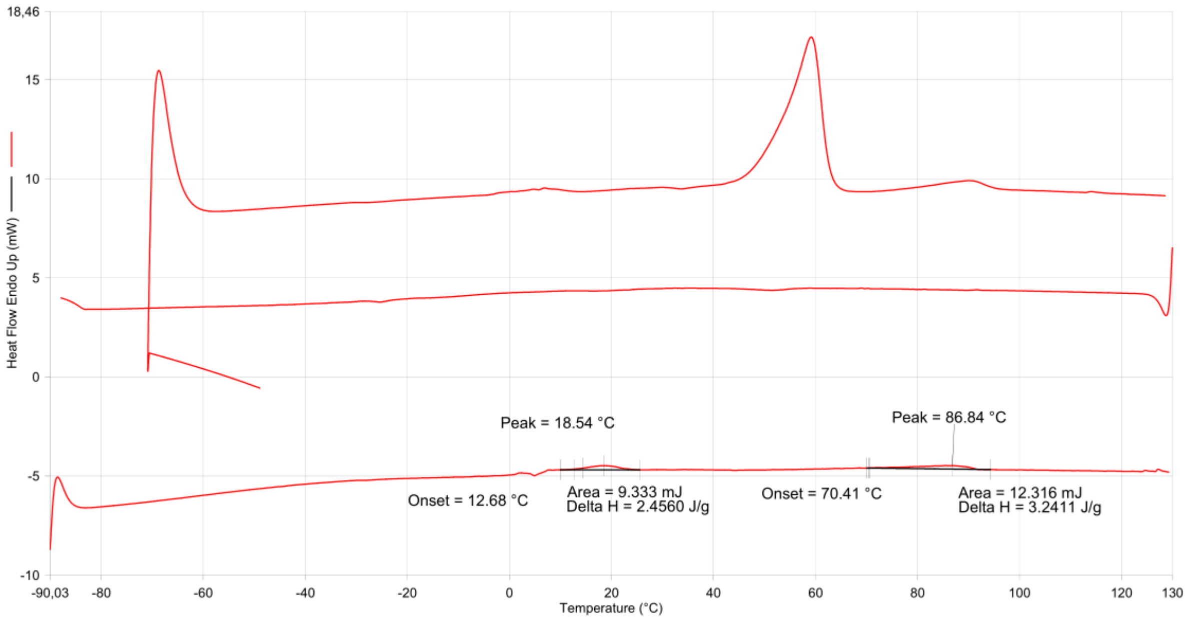


**DSC of 3,7,11-Tris(4-((4-ethyl-2-(2-ethylhexyl)octyl)oxy)phenyl)tris([1,2,4]triazolo)[4,3-a:4',3'-c:4'',3''-e][1,3,5]triazine r-66**

**5-Bromo-2-hexyloxy-benzaldehyde**
5-Bromosalicylic aldehyde(24.9 mmol, 5.00 g), 1-bromohexane (27.4 mmol, 4.52 g), potash (49.8 mmol, 6.87 g) in 22 mL DMF were stirred for 16 h. Aqueous work-up and crystallization from petroleum ether at -18 °C yielded yellowish crystals (4.86 g, 69%) with m.p. = 31 - 32 °C. **$^1$H-NMR** (300 MHz, CDCl$_3$): δ = 10.42(s, 1H, -CHO), 7.92 (d, $^4J$=2.6 Hz, 1H, 6-H, Ar-H), 7.60 (dd, $^3J$ = 8.9 Hz, $^4$J = 2.6 Hz, 1H, 4-H, Ar-H), 6.88 (d, $^3J$ = 8.9 Hz, 1H, 3-H, Ar-H), 4.06 (t, $^3J$ = 6.5 Hz, 2H, -OC*H*$_2$), 1.90 – 1.78 (m, 2H, -CH$_2$), 1.52 – 1.30 (m, 6H, -CH$_2$), 0.95 – 0.86 (m, 3H, -CH$_3$). **IR** (ATR): $\tilde{\nu}$ [cm$^{-1}$] = 2929 s, 2857 s,1681 s, 1590 m, 1482 m, 1464 s, 1383 m, 1268 ss, 1240 s, 1176 m, 1121 m, 1010 w, 937 w, 880 m, 808 m, 726 m, 656 m.

**5-Bromo-2-hexyloxyphenol**
See ref 2. 5-Bromo-2-hexyloxy-benzaldehyde(17.04 mmol4.86 g) and *meta*-chloro perbenzoic acid (34.08 mmol, 5.88 g, 70% - 75%) were stirred in 75 mL dichloromethane for 5 h at ambient temperature. Cooling to 0 °C, filtration. evaporation of the solvent and dissolution of the residue in 50 mL methanol was followed by addition of 1,25 g KOH in 10 mL water. The mixture was neutralized with 2 N HCl, extracted with dichloromethane and after recrystallization from petroleum ether, colorless crystals (3.73 g, 13.65 mmol, 80%) with m.p. 44-45 °C were obtained.
**$^1$H-NMR** (300 MHz, CDCl$_3$): δ = 7.06 (d, $^3J$ = 2.4 Hz, 1H, 6-H, Ar-H), 6.94 (dd, $^4J$ = 8.6 Hz, $^3$J = 2.4 Hz, 1H, 4-H, Ar-H), 6.70 (d, $^4J$ = 8.6 Hz, 1H, 3-H, Ar-H), 5.66 (s, 1H, Ar-OH), 4.01 (t, $^3J$ = 6.6 Hz, 2H, -OCH$_2$), 1.80 (m, 2H, -OCH$_2$-C*H*$_2$), 1.51 – 1.25 (m, 6H, -CH$_2$), 0.95 – 0.87 (m, 3H, -CH$_3$).

**5-Bromo-2-hexyloxy-1-tetradecyloxybenzene**
5-Bromo-2-hexyloxyphenol (13.7 mmol, 3.73 g), potash (30.0 mmol, 4.15 g) and 1-bromotetradecane (15.0 mmol, 4.16 g) in 23 mL DMF were stirred under nitrogen at 100 for 24 h; acidulation with 2 N HCl, extraction with petroleum ether, wahsing oft he organic layer with brine, drying with MgSO$_4$ concentration and cooling to -18 °C gave gave 2.96 g (46%) white crystals with m.ü. = 43 °C. **$^1$H-NMR** (300 MHz, CDCl$_3$): δ = 6.98 (dd, $^4J$ = 6.5 Hz, $^5$J= 2.3 Hz, 2H,4-H, 6-H, Ar-H), 6.73 (dd, $^3J$ = 5.6 Hz, $^5$J= 2.8 Hz, 1H, 3-H, Ar-H), 4.00 – 3.91 (m, 4H, -OCH$_2$), 1.87 – 1.72 (m, 4H, OCH$_2$-C*H*$_2$), 1.54 – 1.21 (m, 34H, -CH$_2$), 0.89 (q, $^3J$ = 6.9 Hz, 6H, CH$_2$-C*H*$_3$).

**4-Hexyloxy-3-tetradecyloxybenzonitrile**
4-Bromo-1-hexyloxy-2-tetradecyloxybenzene (5.30 mmol, 2.5 g), (10.60 mmol, 0.95 g) CuCN, CuJ (0.57 mmol, 0.11 g) and *N,N*'-dimethylethylendiamine (0.60 mmol, 0.53 g, 0.64 mL) in 10 mL *N*-methyl-2-pyrrolidone were stirred for 1.5 h at 200 °C. the cooled solution was diluted with water (60 mL), hydrochlorid acid (36 %, 1.2 mL) and $FeCl_3$ (3.1 g) were added and the mixture stirred for 30 min at 60 °C. Extraction with methylene chloride, washing of the organic layer brine, drying with sodium sulfate and evaporation of the solvent was followed by recrystallization from ethaol/petroleum ether (10/1) to yield 1.6 g (72%) of a colorless solid with m.p. = 58 -60 °C. **$^1$H-NMR** (300 MHz, $CDCl_3$): δ = 7.23 (dd, $^3J$ = 8.3 Hz, $^4$J = 1.9 Hz, 1H, 6-H), 7.07 (d, $^3J$ = 1.9 Hz, 1H, 2-H), 6.86 (d, $^4J$ = 8.4 Hz, 1H, 5-H), 4.06 – 3.95 (m, 4H, -$OCH_2$), 1.90 – 1.76 (m, 4H, -$OCH_2$-C*H₂*), 1.51 – 1.23 (m, 18H,-$CH_2$), 0.96 – 0.83 (m, 6H, -$CH_2$-$CH_3$). **$^{13}$C NMR** (75 MHz, $CDCl_3$): δ = 153.2 (4-C, Ar-C), 149.1 (3-C, Ar-C), 126.4 (6-C, Ar-CH), 119.6 (-CN), 116.1 (5-C, Ar-CH), 112.8 (2-C, Ar-CH), 103.6 (1-C, Ar-C), 69.5, 69.2 (-$OCH_2$), 32.0, 31.6, 29.8, 29.8, 29.7, 29.5, 29.1, 29.0, 26.1, 25.7, 22.8, 22.7 (-$CH_2$), 14.2, 14.1 (-$CH_3$). **IR** (ATR): $\tilde{\nu}$ [cm$^{-1}$] = 2915 s, 2850 s, 2220 m, 1595 m, 1517 s, 1466 m, 1276 s, 1243 s, 1136 s, 1046 w, 869 w, 809 s. **HR-ESI:** calcd. for $C_{27}H_{45}O_2N_1$ + $H^+$: 416.3529; found: 416.3521.

**5-(3-Tetradecyloxy-4-hexyloxyphenyl)-*2H*-tetrazole**
4-Hexyloxy-3-tetradecyloxybenzonitrile (3.61 mmol, 1.5 g), (12.63 mmol, 0.82 g) $NaN_3$, $NEt_3$ HCl (12.63 mmol, 1.74 g) in 50 mL toluene were refluxed for 24 h. Additional reagents ( 0.23 g $NaN_3$, 0.49 g $NEt_3$ HCl) were added and after further 48 h refluxing, aqueous work-up with 2 N HCL and Extraction with ethyl acetate followed by chromatography on silica gel using toluene/ethyl acetate/ acetic acid 5/571 yielded, after recrystallization from toluene, 593 mg (1.29 mmol) of a colorless solid with m.p. = 153-155 °C.
**$^1$H-NMR** (300 MHz, $CDCl_3$): δ = 7.63 (d, $^4J$ = 2.0 Hz, 1H, 2-H, Ar-H), 7.58 (dd, $^3J$ = 8.3 Hz, $^4$J = 2.0 Hz, 1H, 6-H, Ar-H), 6.92 (d, $^3J$ = 8.4 Hz, 1H, 5-H, Ar-H), 4.07 – 3.97 (m, 4H, -$OCH_2$), 1.80 (p, $^3J$ = 6.8 Hz, 4H, -$OCH_2$-$CH_2$), 1.52 – 1.39 (m, 4H, -$CH_2$), 1.37 – 1.17 (m, 21H,-$CH_2$), 0.91 – 0.80 (m, 6H, $CH_2$). **$^{13}$C NMR** (75 MHz, $CDCl_3$): δ = 151.5 (4-C, Ar-C), 149.4 (3-C, Ar-C), 120.3 (6-C, Ar-CH), 113.2 (5-C, Ar-C), 112.3 (2-C, Ar-C), 69.3, 69.2 (-$OCH_2$), 32.0, 31.6, 29.7, 29.7, 29.5, 29.4, 29.2, 29.1, 26.1, 25.7, 22.7, 22.6 (-$CH_2$), 14.2, 14.1 (-$CH_3$). **IR** (ATR): $\tilde{\nu}$ [cm$^{-1}$] = 2916 s, 2850 m, 1607 w, 1509 s, 1464 m, 1397 w, 1269 s, 1235 m, 1132 m, 1039 m, 997 m, 874 m, 811 m, 745 m. **HR-ESI:** calcd. for $C_{27}H_{46}O_2N_4$ + $H^+$: 459.3699; found: 459.3711.

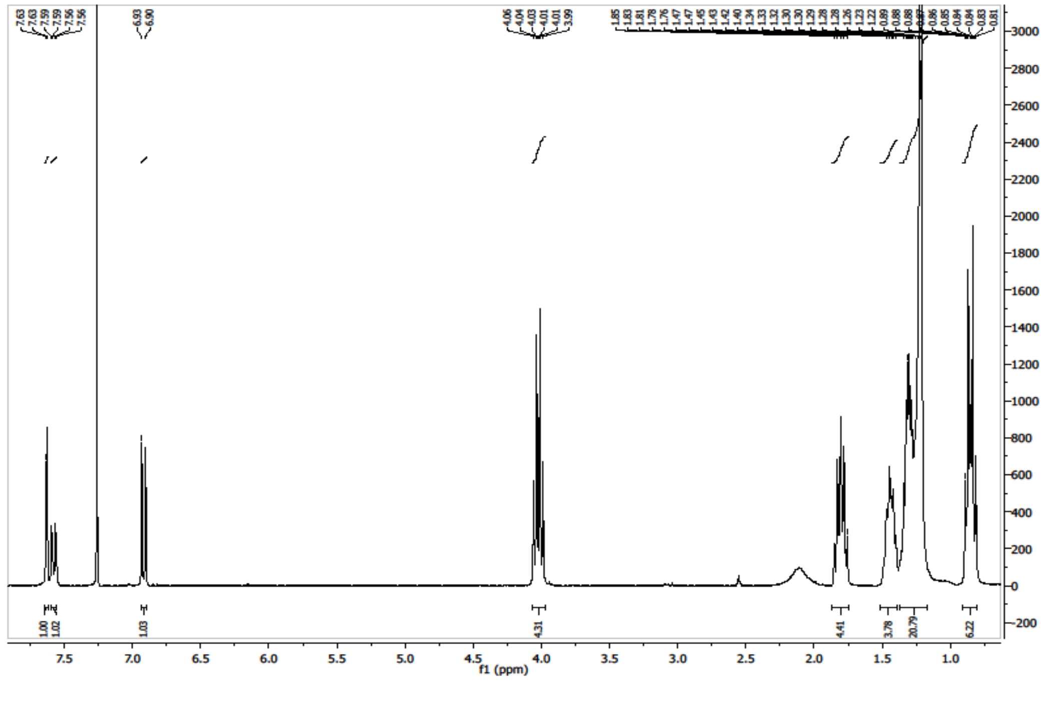

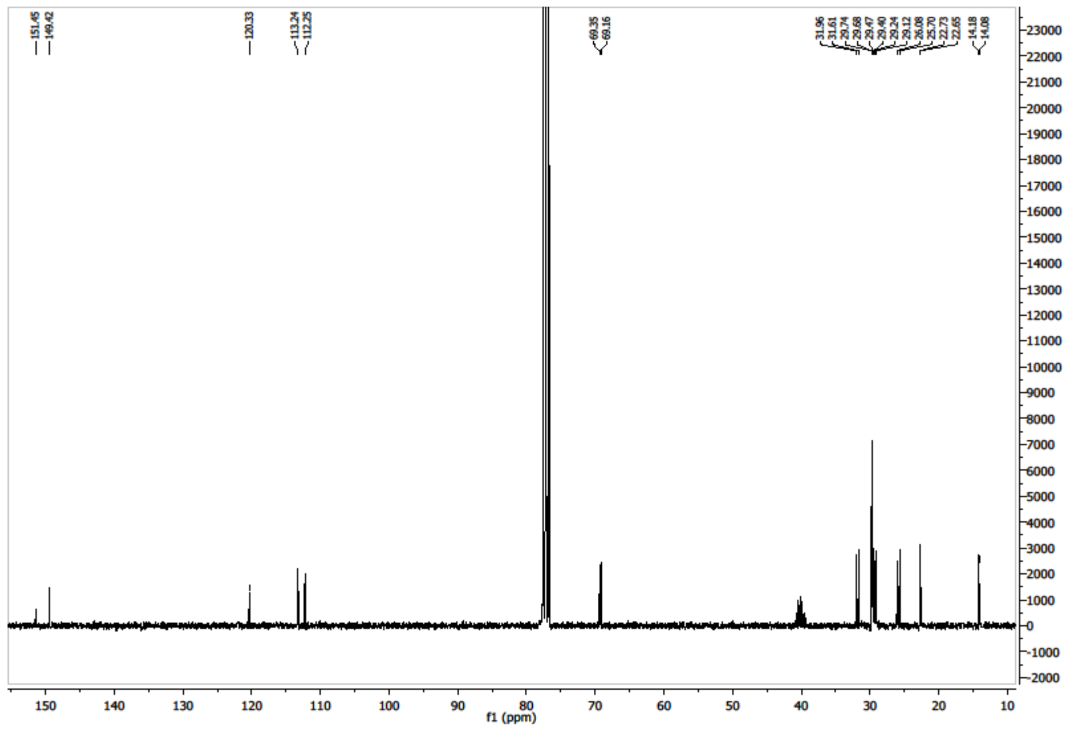


$^{1}$H- and $^{13}$C-NMR spectra of 4-(hexyloxy)-3-(tetradecyloxy)phenyl)tetrazole

**3,7,11-Tris(3-(hexadecyloxy)-4-methoxyphenyl)tris([1,2,4]triazolo)[4,3-a:4',3'-c:4'',3''-e][1,3,5]triazine *t*-75**

According to the general procedure, hexadecyloxy-methoxyphenyltetrazole (559 mg), collidine (162 mg) and cyanuric chloride (745 mg) in 20 mL dry toluene gave 328 mg (65%) of a waxy solid with m.p. = 98 after chromatography on silica with toluene/ethyl acetate 20/1. **$^1$H-NMR** (300 MHz, $CDCl_3$): δ = 7.85 (dd, $^3J$ = 8.5 Hz, $^4J$ = 2.0 Hz, 1H, Ar-H), 7.70 (d, $^4J$ = 2.0 Hz, 1H, Ar-H), 7.04 (d, $^3J$ = 8.6 Hz, 1H, Ar-H), 4.11 (m, 2H, $-OCH_2$), 3.96 (s, 3 H), 1.93 – 1.83 (m, 2H, $-OCH_2$-C*$H_2$*), 1.39 – 1.18 (m, 26H,$-CH_2$), 0.87 (t, 3H, $-CH_3$). **$^{13}$C NMR** (75 MHz, $CDCl_3$): δ = 152.5, 151.1 (5-C, TTT-C, 4-C, Ar-C), 148.5 (3-C, Ar-C), 140.6 (3-C, TTT-C), 123.8 (6-C,Ar-C), 116.1 (1-C, Ar-C), 114.3 (5-C, Ar-C), 111.1 (2-C, Ar-C), 69.4, ($-OCH_2$), 56.2 ($OCH_3$), 32.1 - 22.8 ($-CH_2$), 14.3, 14.2 ($-CH_3$). **IR** (ATR): $\tilde{\nu}$ [$cm^{-1}$] = 2921 ss, 2852 s, 1598 s, 1492 s, 1467 s, 1263 s, 1231 m. FD-MS: 1239 ($M^+$, 100%).Analysis: **HR-ESI:** calcd. for $C_{75}H_{117}O_6N_9$ : C 72.60, H 9.50, N 10.16; found: C 72.62, H 9,52; N: 9.73.

**3,7,11-Tris(4-(hexyloxy)-3-(tetradecyloxy)phenyl)tris([1,2,4]triazolo)[4,3-a:4',3'-c:4'',3''-e][1,3,5]triazine *t*-76**

According to the general procedure, 5-(4-hexyloxy-3-tetradecyloxyphenyl)-2*H*-tetrazole (1.02 mmol, 470 mg) , cyanuric chloride( 57 mg) were stirred and successively heated to 110 °C. After 14 h, the mixture wascooled, acidulated and the organic layer washed with brine. The combined aqueous layers were extracted with ethyl acetate. The combined organic layers were dried, concentrated and evaporated to yield 700 mg of a brown solid. This was purified by chromatography on a silica gel column with head of basic alumina and toluene/ethyl acetate 20/1 as an eluent zto yield 238 mg (56 %) of a colorless solid with m.p. = 182 °C (DSC, 2nd heating). **$^1$H-NMR** (300 MHz, $CDCl_3$): δ = 7.82 (dd, $^3J$ = 8.4 Hz, $^4J$ = 2.1 Hz, 1H, 6-H, Ar-H), 7.70 (d, $^4J$ = 2.1 Hz, 1H, 2-H, Ar-H), 7.02 (d, $^3J$ = 8.6 Hz, 1H, 5-H, Ar-H), 4.14 – 4.06 (m, 4H, $-OCH_2$), 1.94 – 1.79 (m, 4H, $-OCH_2$-C*$H_2$*), 1.56 – 1.21 (m, 28H,$-CH_2$), 0.95 –

0.83 (m, 6H, $-CH_3$). **$^{13}$C NMR** (75 MHz, $CDCl_3$): δ = 152.4, 151.2 (5-C, TTT-C, 4-C, Ar-C), 148.9 (3-C, Ar-C), 140.6 (3-C, TTT-C), 123.9 (6-C,Ar-C), 116.0 (1-C, Ar-C), 115.1 (5-C, Ar-C), 112.6 (2-C, Ar-C), 69.5, 69.2 ($-OCH_2$), 32.1, 31.7, 29.9, 29.8, 29.6, 29.5, 29.3, 29.2, 26.2, 25.8, 22.8, 22.8 ($-CH_2$), 14.3, 14.2 ($-CH_3$). **IR** (ATR): $\tilde{\nu}$ [$cm^{-1}$] = 2920 ss, 2851 s, 1596 s, 1574 s, 1524 w, 1490 s, 1466 s, 1397 w, 1328 w, 1259 s, 1230 m, 1139 m, 1010 m, 884 m, 804 m, 713 m, 698 m, 657 w. **HR-ESI:** calcd. for $C_{84}H_{135}O_6N_9 + H^+$: 1367.0614, found: 1367.0659.

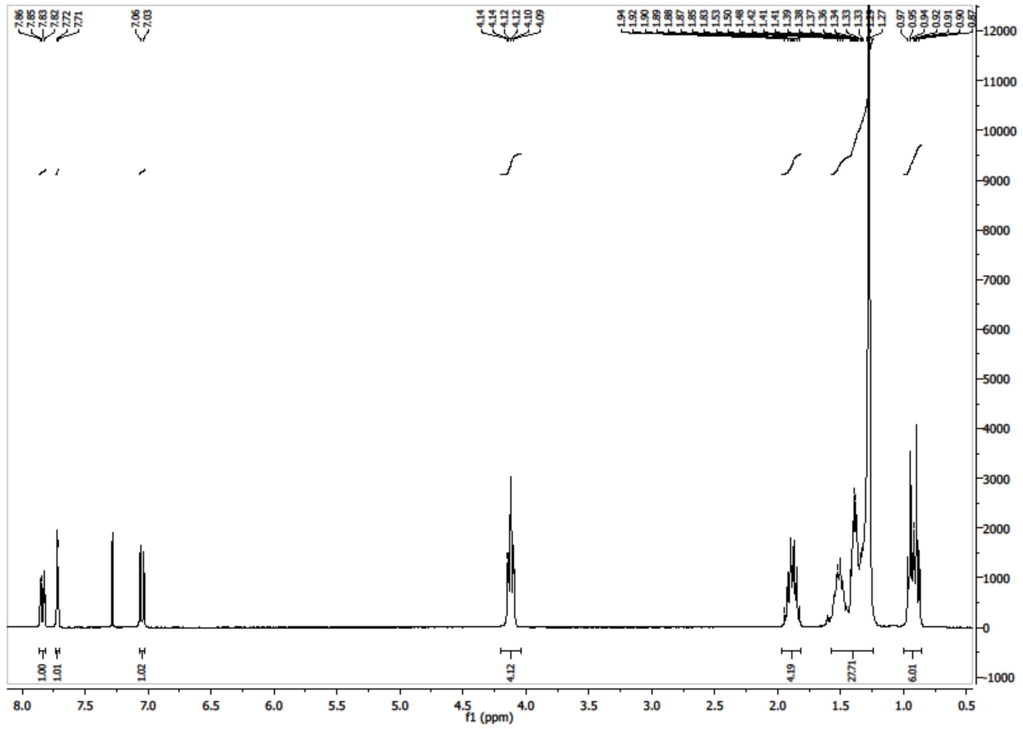


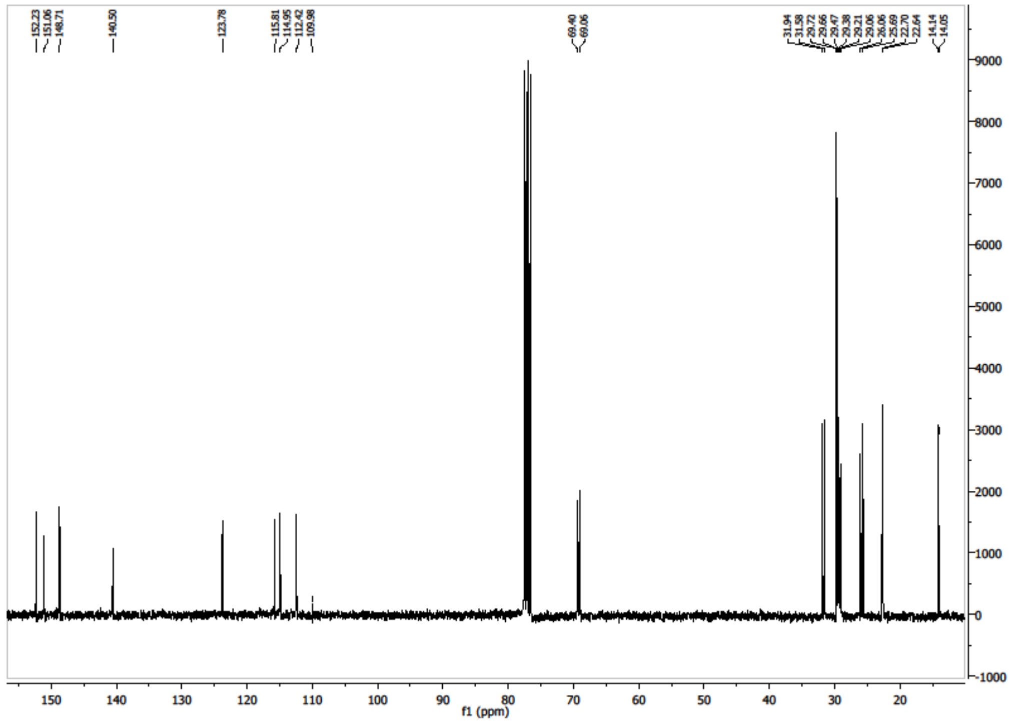


$^1$H- and $^{13}$C-NMR spectra of ***t*-76**

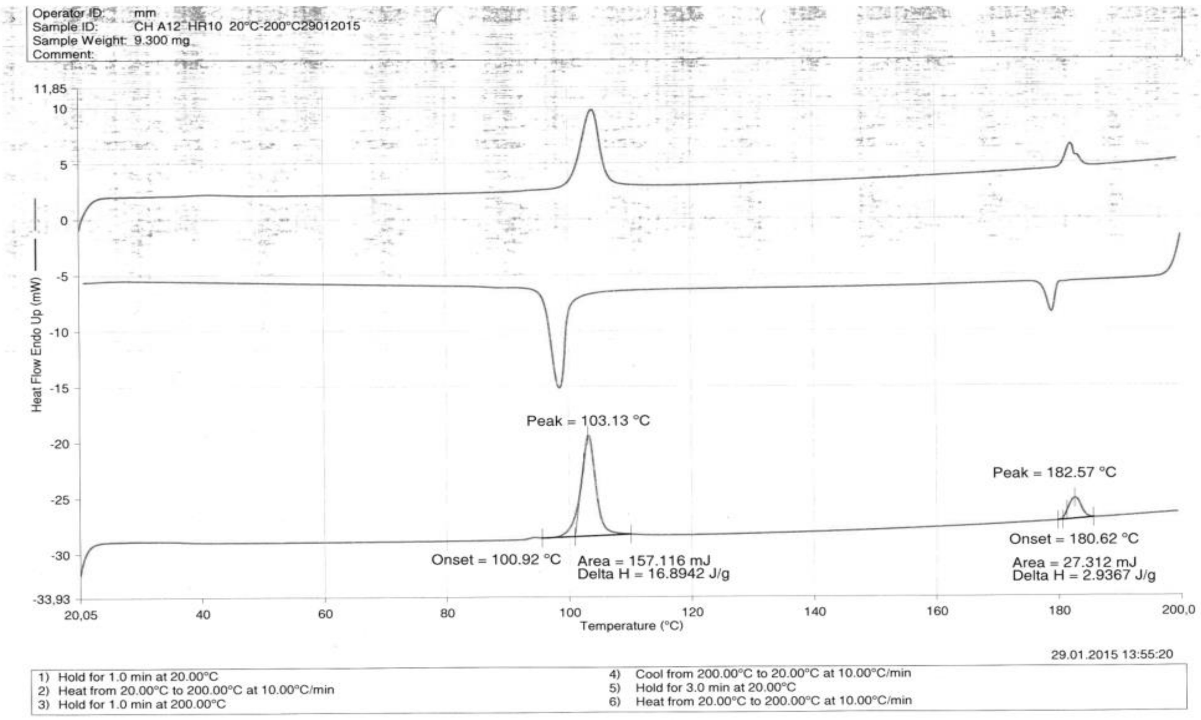


DSC trace of ***t*-76**

**4-Cyanosalicylic acid**

0.98 g (3.7 mmol, 1.0 eq) 4-iodosalicylic acid and 498 mg (5.57 mmol, 1.5 eq) copper(I) cyanide were susepended in dry DMF and stirred at 140 °C for 22 h and at 90 °C for 66 h. The mixture was quenched with 20 mL water and filtered. The filter cake was washed with ethyl acetate, the filtrate acidified with hydrochloric acid (2N) and extracted with ethyl acetate. The combined extracts were washed with a saturated solution of ammonium chloride and brine and dried over $MgSO_4$. The solvent was removed under reduced pressure and the crude product purified via column chromatography ($SiO_2$; EA→EA:HOAc = 100:2). 140 mg (0.86 mmol, 23 %) of the desired product was obtained as a brown solid.
**$^1$H-NMR** (300 MHz, acetone-$_{d6}$) δ = 8.05 (d, $^3J$ = 8.1 Hz, 1H), 7.34 (d, $^4J$ = 1.6 Hz, 1H), 7.29 (dd, $^3J$ = 8.1 Hz, $^4J$ = 1.6 Hz, 1H).

**Decyl 4-cyano-2-(decyloxy)benzoate** This compound was prepared according to general procedure from 305 mg (1.87 mmol, 1 eq) 4-cyano-2-hydroxybenzoic acid, 0.82 mL (4.0 mmol, 2.1 eq) 1-bromodecane and 505 mg (4.3 mmol, 2.3 eq) $K_2CO_3$ in 20 mL DMF. Recrystallization from ethanol afforded 536 mg (1.21 mmol, 65 %) of the product as colorless solid. m.p.: 54-55 °C. **$^1$H NMR** (300 MHz, $CDCl_3$) δ = 7.79 (d, $^3J$ = 7.9 Hz, 1H, 6-H), 7.30 – 7.20 (m, 1H, 5-H), 7.18 (d, $^4J$ = 1.3 Hz, 1H, 2-H), 4.30 (t, $^3J$ = 6.7 Hz, 2H, OOC*H₂*), 4.03 (t, $^3J$ = 6.5 Hz, 2H, OC$H_2$), 1.89 – 1.68 (m, 4H, (O)OC$H_2$C$H_2$), 1.54 – 1.13 (m, 28H, C$H_2$), 0.96 – 0.79 (m, 6H, C$H_3$). **$^{13}$C-NMR** (75 MHz, $CDCl_3$) δ = 165.67, 158.22, 132.08, 125.61, 123.71, 118.24, 116.18 (2C), 69.52, 65.91, 32.05 (2C), 29.72 (4C), 29.47 (4C), 29.10, 28.81, 26.16 (2C), 26.04 (2C), 22.83 (2C), 14.26 (2C). **IR** (neat): ṽ [1/cm] = 3734 w, 2920 s, 2851 s, 2227 w, 1702 ss, 1607 m, 1560 m, 1466 m, 1420 s, 1397 m, 1296 ss, 1255 s, 1171 m, 1137 s, 1097 m, 1021 m, 994 m, 957 m, 863 m, 835 m, 780 s, 720 m, 696 m, 670 w. **HRMS-ES(+):** [M+H]+: calc.: 444.3472 found: 444.3464.

**Decyl 2-(decyloxy)-4-(1*H*-tetrazol-5-yl)benzoate**

This compound was prepared from 431 mg (0.97 mmol, 1 eq) decyl 4-cyano-2-(decyloxy)benzoate 189 mg (2.92 mmol, 3 eq) sodium azide and 156 mg (2.92 mmol, 3 eq) ammonium chloride in 15 mL dry DMF. The mixture was stirred for 21 h at 130 °C before the reaction was quenched with hydrochloric acid (2N). The solution was cooled and a white solid precipitated. The mixture was filtered and the filter cake washed with water and dried on air. The reaction afforded 418 mg (0.859 mmol, 89 %) of the desired product as a colorless solid. m.p.: 118-120 °C. **$^1$H-NMR** (300 MHz, $CDCl_3$) δ = 7.87 (d, $^3J$ = 8.0 Hz, 1H, 6-H), 7.72 (d, $^4J$ = 1.4 Hz, 1H, 3-H), 7.67 (dd, $^3J$ = 8.0, $^4J$ = 1.4 Hz, 1H, 5-H), 4.36 (t, $^3J$ = 6.7 Hz, 2H, OOC*H₂*), 4.03 (t, $^3J$ = 6.6 Hz, 2H, OC*H₂*), 1.86 – 1.65 (m, 4H, (O)OCH₂C*H₂*), 1.49 – 1.17 (m, 28H, C*H₂*), 0.94 – 0.81 (m, 6H, C*H₃*). **IR** (neat): ṽ [1/cm] = 3734 w, 2956 m, 2919 s, 2850 s, 2675 w, 2528 w, 2460 w, 1900 w, 1704 ss, 1568 m, 1498 m, 1448 s, 1368 m, 1292 ss, 1248 s, 1162 s, 1136 m, 1067 m, 1048 s, 1000 m, 849 m, 778 m, 747 s, 720 m, 690 m, 637 w, 617 m. **HRMS-ES(+):** [M+H]+: calc.: 487.3643 found: 487.3643.

**Tris(decyl) 4,4',4''-(tris([1,2,4]triazolo)[4,3-a:4',3'-c:4'',3''-e][1,3,5]triazine-3,7,11-triyl)tris(2-(decyloxy)benzoate) *t*-77**

This compound was prepared according to general procedure from 350 mg (0.73 mmol, 3.3 eq) decyl 2-(decyloxy)-4-(1*H*-tetrazol-5-yl)benzoate **215**, 0.2 mL (1.5 mmol, 6.6 eq) 2,4,6-collidine and 41 mg (0.22 mmol, 1.0 eq) cyanuric chloride in 15 mL xylene. Column chromatography ($SiO_2$ + 2 cm $Al_2O_3$; Tol:EA = 50:1) afforded 18 mg (0.012 mmol, 6 %) of the product as brown solid.

**$^1$H-NMR** (300 MHz, $CDCl_3$) δ = 7.95 (d, $^3J$ = 7.9 Hz, 1H,), 7.84 – 7.77 (m, 2H), 4.34 (t, $^3J$ = 6.7 Hz, 2H, OOC*H₂*), 4.15 (t, $^3J$ = 6.5 Hz, 2H, OC*H₂*), 1.95 – 1.66 (m, 4H, (O)OCH₂C*H₂*), 1.52 – 1.20 (m, 28H, C*H₂*), 0.94 – 0.78 (m, 6H, C*H₃*). **$^{13}$C-NMR** (75 MHz, $CDCl_3$) δ = 166.18 (3C), 158.27 (3C), 150.49 (3C), 140.84 (3C), 131.77 (3C), 127.80 (3C), 124.28 (3C), 121.74 (3C), 114.84 (3C), 69.48 (3C), 65.64 (3C), 32.06 (6C), 29.76 (12C), 29.58 (3C), 29.53 (3C), 29.50 (6C), 29.30 (3C), 28.92 (3C), 26.24 (3C), 26.16 (3C), 22.84 (6C), 14.27 (6C). **IR** (neat): ṽ [1/cm] = 3734 w, 2952 m, 2921 ss, 2852 s, 1716 s, 1593 s, 1572 s, 1523 w, 1467 m, 1434 s, 1394 m, 1291 s, 1251 ss, 1146 m, 1113 m, 1081 s, 869 m, 840 m, 781 m, 719 s, 638 w, 617 w, 608 w. **HRMS-ES(+):** [M+H]+: calc.: 1451.0456 found: 1451.0438.

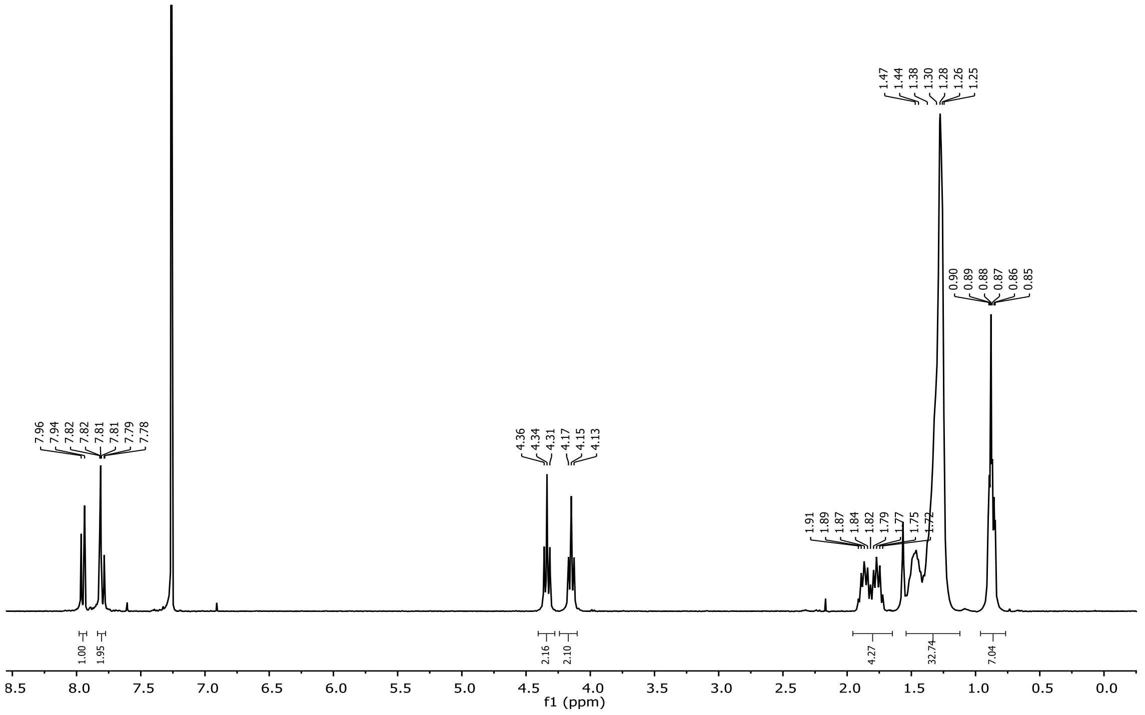

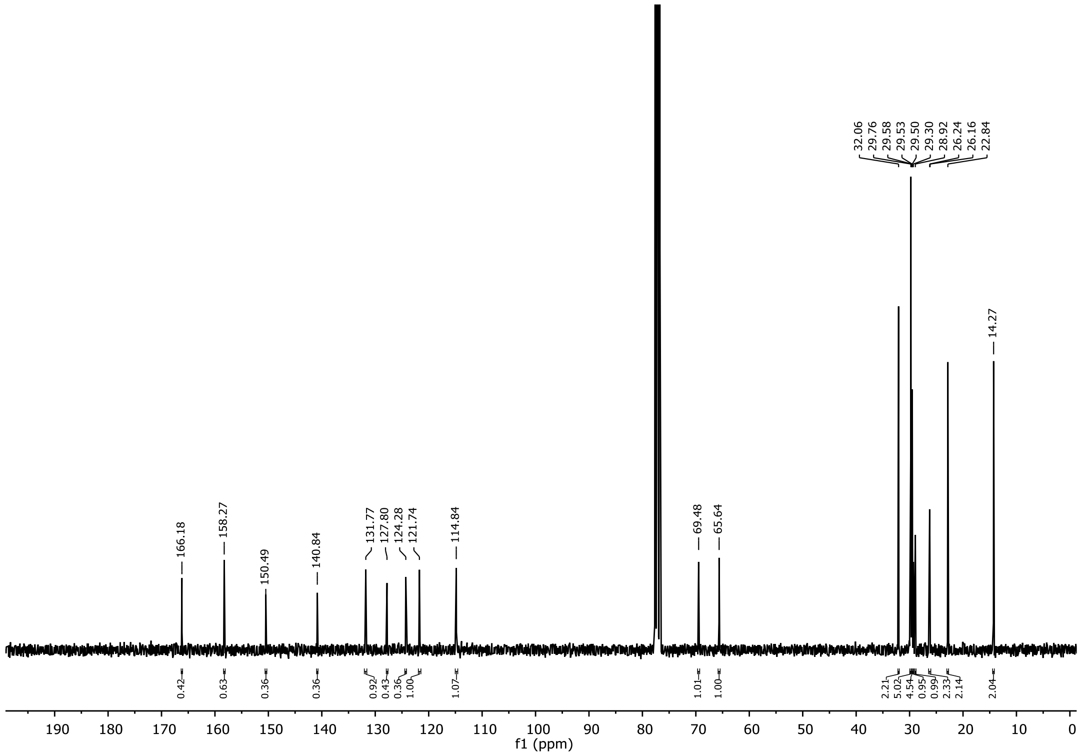


$^{1}$H- and $^{13}$C-NMR spectra of tris(decyl) 4,4',4''-(tris([1,2,4]triazolo)[4,3-a:4',3'-c:4'',3''-e][1,3,5]triazine-3,7,11-triyl)tris(2-(decyloxy)benzoate) ***t*-77**

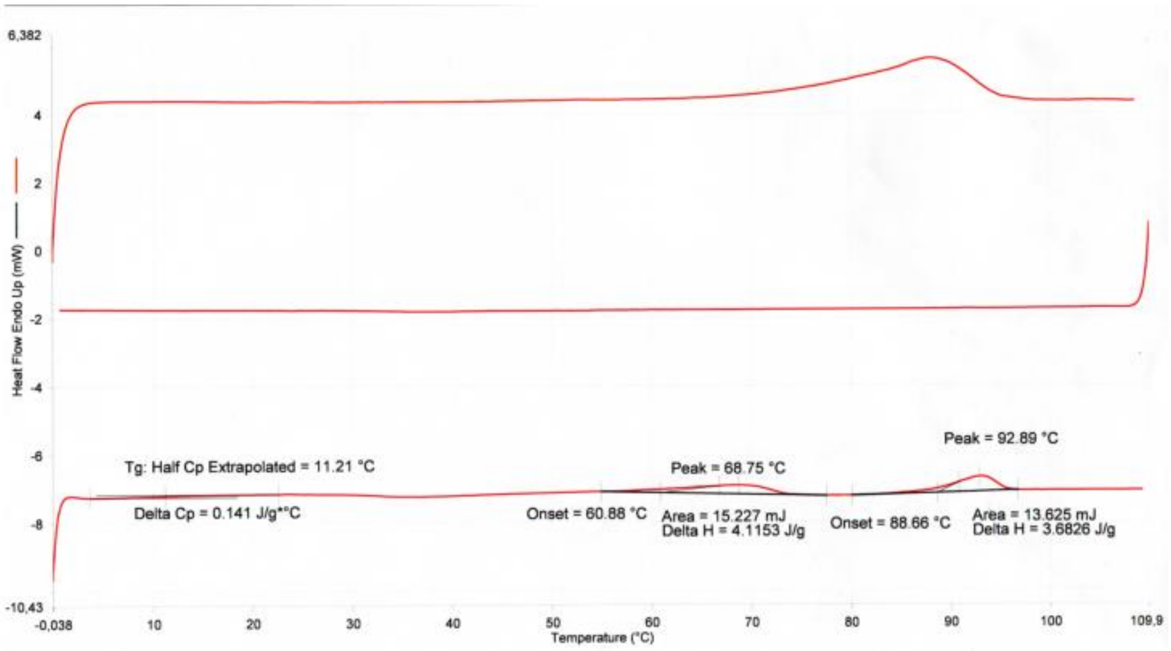


DSC of Tris(decyl) 4,4',4''-(tris([1,2,4]triazolo)[4,3-a:4',3'-c:4'',3''-e][1,3,5]triazine-3,7,11-triyl)tris(2-(decyloxy)benzoate) ***t*-77**

**3-(Dodecyloxy)-4,5-bis(hexyloxy)benzoic acid**

This compound was prepared according to general procedure from 643 mg (1.20 mmol, 1 eq) ethyl 3-dodecyloxy-4,5-di(hexyloxy)benzoate, 4 mL sodium hydroxide solution (2N, 8 mmol, 6.7 eq) and 16 mL ethanol. The crude product was used without further purification.

**3-(Dodecyloxy)-4,5-bis(hexyloxy)benzamide**
This compound was prepared according to general procedure from crude 3-(dodecyloxy)-4,5-bis(hexyloxy)benzoic acid and 5 mL (69 mmol) thionyl chloride. 25 mL toluene was used to dissolve the benzoyl chloride. The crude product was recrystallized from ethanol and afforded 517 mg (1.02 mmol, 85 % (3 steps)) of the desired product as a colorless solid. m.p.: 73-75 °C. **$^{1}$H-NMR** (400 MHz, $CDCl_3$) δ = 7.02 (s, 2H, 2/6-H), 5.93 (s, br, 1H, N*H*), 5.57 (s, br, 1H, N*H*), 4.05 – 4.01 (m, 6H, $OC\mathit{H_2}CH_2$), 1.87 – 1.73 (m, 6H, $OCH_2C\mathit{H_2}$), 1.54 – 1.45 (m, 6H), 1.41 – 1.28 (m, 24H, $C\mathit{H_2}$), 0.99 – 0.88 (m, 9H, $C\mathit{H_3}$). **$^{13}$C-NMR** (101 MHz, $CDCl_3$) δ = 169.08, 153.07 (2C), 141.66, 128.10, 106.16 (2C), 73.52, 69.40 (2C), 31.93, 31.74, 31.55, 30.27, 29.69, 29.65, 29.63 (2C), 29.39, 29.36 (2C), 29.30, 26.07, 25.73 (2C), 22.69 (2C), 22.62, 14.13, 14.10, 14.03. **IR** (neat): ṽ [1/cm] = 3351 m, 3181 w, 2920 m, 2851 m, 1648 m, 1620 m, 1577 m, 1505 w, 1467 m, 1428 m, 1378 m, 1316 m, 1235 m, 1115 ss, 921 w, 850 m, 806 m, 722 m, 688 m, 662 m. **HRMS-ES(+):** [M+Na]+: calc.: 528.4023 found: 528.4012.

**1*H*-5-(3-(Dodecyloxy)-4,5-bis(hexyloxy)phenyl)tetrazole** was prepared according to the general procedure from 480 mg (0.949 mmol, 1 eq) 3-(dodecyloxy)-4,5-bis(hexyloxy)benz-amide, 407 mg (6.25 mmol, 6.6 eq) sodium azide and 0.24 mL (2.09 mmol, 2.2 eq) silicon tetrachloride in 23 mL acetonitrile. Column chromatography ($SiO_2$; Tol→Tol: EA=4:1) afforded 128 mg (0.241 mmol, 25 %) of the product as colorless solid. m. p.: 79-80 °C. **$^{1}$H-NMR** (300 MHz, $CDCl_3$) δ = 7.22 (s, 2H, 2/6-H), 4.04 (t, $^{3}J$ = 6.7 Hz, 2H, $OC\mathit{H_2}$), 3.97 (t, $^{3}J$ = 6.5 Hz, 4H, $OC\mathit{H_2}$), 1.82 – 1.72 (m, 6H, $OCH_2C\mathit{H_2}$), 1.54 – 1.14 (m, 30H, $C\mathit{H_2}$), 0.95 – 0.81 (m, 9H, $C\mathit{H_3}$). **$^{13}$C-NMR** (75 MHz, $CDCl_3$) δ = 153.78 (2C), 140.32, 119.41, 105.52 (2C), 73.86, 69.30 (2C), 32.08, 31.89, 31.72, 30.41, 29.87, 29.83 (3C), 29.63, 29.53, 29.48, 29.40, 26.25, 25.86 (2C), 22.84 (2C), 22.74, 14.27, 14.23, 14.16. **IR** (neat): ṽ [1/cm] = 2921 s, 2851 m, 2617 w, 1583 m, 1503 m, 1444 s, 1379 m, 1308 m, 1249 m, 1116 ss, 1050 m, 990 w, 881 w, 846 m, 820 w, 722 w. **HRMS-ES(+):** [M·]+: calc.: 531.4269 found: 531.4269.

**3,7,11-Tris(3-(dodecyloxy)-4,5-bis(hexyloxy)phenyl)tris([1,2,4]triazolo)[4,3-a:4',3'-c:4'',3''-e][1,3,5]triazine *t*-78** was prepared according to general procedure from 126 mg (0.237 mmol, 3.4 eq) 1*H*-5-(3-(dodecyloxy)-4,5-bis(hexyloxy)phenyl)tetrazole, 0.05 mL (0.4 mmol, 5.7 eq) 2,4,6-collidine and 13 mg (0.070 mmol, 1.0 eq) cyanuric chloride in 10 mL xylene. Column chromatography ($SiO_2$ + 2 cm $Al_2O_3$; Tol) afforded 58 mg (0.037 mmol, 15 %) of the product as colorless solid. **$^{1}$H NMR** (300 MHz, $CDCl_3$) δ = 7.43 (s, 6H), 4.07 (t, $^{3}J$ = 6.6 Hz, 18H), 1.98 – 1.67 (m, 18H), 1.55 – 1.42 (m, 18H), 1.40 – 1.20 (m, 72H), 1.02 – 0.77 (m, 27H). **$^{13}$C-NMR** (75 MHz, $CDCl_3$) δ = 153.17, 151.38, 141.47, 140.72, 118.31, 108.90, 73.79, 69.47, 32.07, 31.92, 31.74, 30.47, 29.85, 29.79, 29.79, 29.60, 29.51, 29.50, 29.43, 26.28, 25.93, 25.89, 22.86, 22.85, 22.78, 14.27, 14.20. **IR** (neat): ṽ [1/cm] = 3119 w, 2955 m, 2933 m, 2870 w, 1738 w, 1586 m, 1488 m, 1458 m, 1433 m, 1377 w, 1340 m, 1230 m, 1117 m, 1004 w, 993 w, 840 w, 760 w, 721 w. **HRMS-ES(+):** [M+H]+: calc.: 1583.2334 found: 1583.2284.

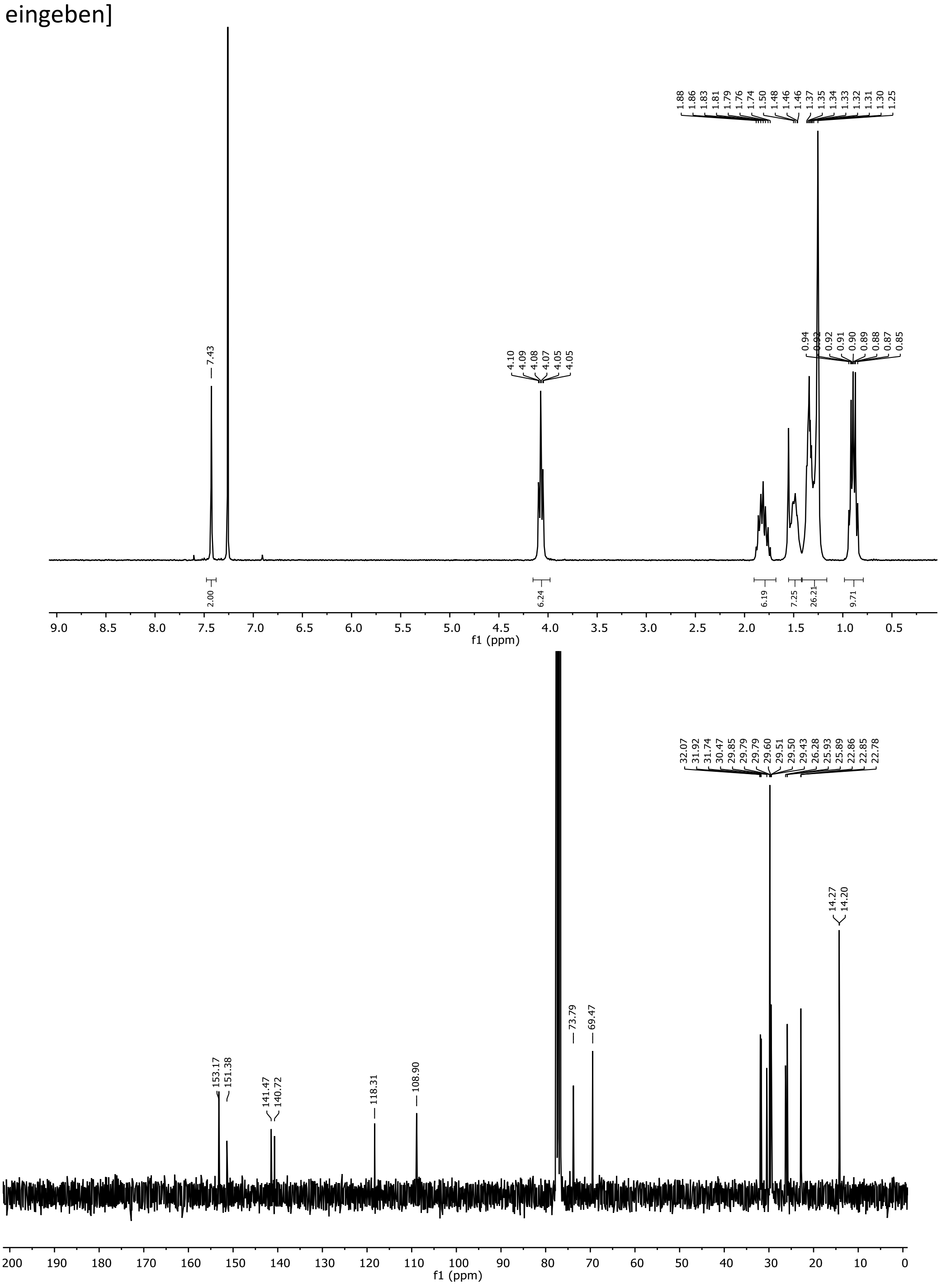


$^{1}H$- and $^{13}C$-NMR spectra of 3,7,11-Tris(3-(dodecyloxy)-4,5-bis(hexyloxy)phenyl)tris([1,2,4]triazolo)[4,3-a:4',3'-c:4'',3''-e][1,3,5]triazine ***t*-78**

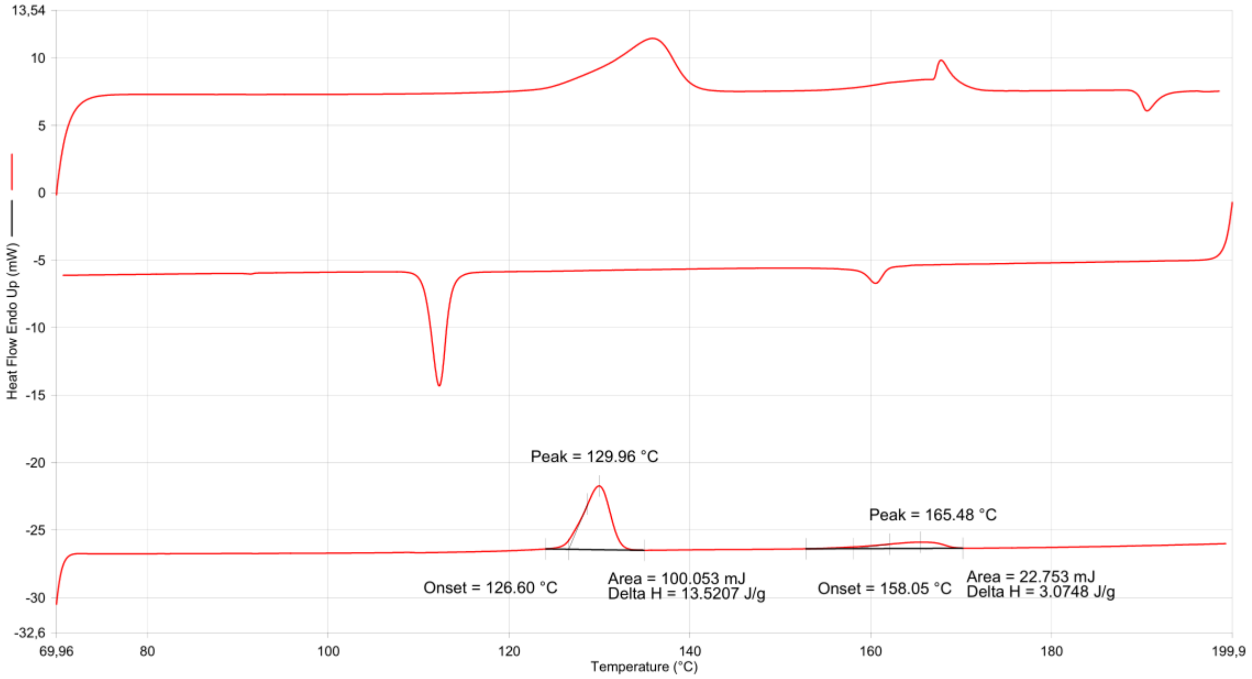


DSC of ***t*-78**

**Ethyl 3-(4-ethyloctyloxy)-4,5-di(hexyloxy)benzoate** This compound was prepared according to general procedure from 573 mg (1.56 mmol, 1 eq) ethyl 3-hydroxy-4,5-di(hexyloxy)benzoate, 0.41 g (1.85 mmol, 1.2 eq) 1-bromo-4-ethyloctane and 0.33 g (2.39 mmol, 1.5 eq) $K_2CO_3$ in 30 mL toluene. Additional 0.33 g (2.75 mmol, 1.8 eq) $K_2CO_3$ and 0.55 g (2.49 mmol, 1.6 eq) 1-bromo-4-ethyloctane were added to the reaction mixture. The ester was used without further purification.

**3-(4-Ethyloctyloxy)-4,5-bis(hexyloxy)benzoic acid**
This compound was prepared according to general procedure from ethyl 3-(4-ethyloctyloxy)-4,5-di(hexyloxy)benzoate (crude product), 10 mL sodium hydroxide solution (2N, 20 mmol) and 10 mL ethanol. The crude product was used without further purification in the next step. $^{1}$H NMR (300 MHz, $CDCl_3$) δ = 7.32 (s, 1H, 2/6-H), 4.07 – 3.99 (m, 6H, OC*H*$_2$), 1.87 – 1.71 (m, 6H), 1.53 – 1.26 (m, 23H), 0.94 – 0.84 (m, 12H).

**3-(4-Ethyloctyl)-4,5-bis(hexyloxy)benzamide** This compound was prepared according to general procedure DL-Amide from crude 3-(4-ethyloctyl)-4,5-bis(hexyloxy)benzoic acidand 5 mL (49 mmol) thionyl chloride. 15 mL toluene was used to dissolve the benzoyl chloride. The reaction afforded 356 mg (0.764 mmol, 49 % (4 steps)) of the desired product as a colorless solid without further purification.
m.p.: 70-80 °C. $^{1}$H-NMR (300 MHz, $CDCl_3$) δ = 7.00 (s, 2H, 2/6-H), 6.01 – 5.47 (m, 2H, N*H*$_2$), 4.11 – 3.84 (m, 6H, OC*H*$_2$), 1.90 – 1.64 (m, 6H, OCH$_2$C*H*$_2$), 1.54 – 1.19 (m, 23H, C*H*, C*H*$_2$), 0.93 – 0.83 (m, 12H, C*H*$_3$). $^{13}$C-NMR (75 MHz, $CDCl_3$) δ = 169.27, 153.21 (2C), 141.77, 128.23, 106.29, 106.24, 73.68, 69.98, 69.54, 38.74, 32.92, 31.89, 31.70, 30.42, 29.53, 29.45, 29.07, 26.72, 25.94, 25.87 (2C), 23.29, 22.83, 22.77, 14.31, 14.24, 14.19, 10.97. IR (neat): $\tilde{\nu}$ [1/cm] = 3369 w, 3185 w, 2956 s, 2933 s, 2871 m, 1738 m, 1647 s, 1578 s, 1429 s, 1380 s, 1230 m, 1119 ss, 748 w. **HRMS-ES(+):** [M+H]+: calc.: 478.3891 found: 478.3888.

**1*H*-5-(3-(4-Ethyloctyl)-4,5-bis(hexyloxy)phenyl)tetrazole** This compound was prepared according to general procedure DL-Tet2 from 349 mg (0.731 mmol, 1 eq) 3-(3-ethyloctyl)-4,5-bis(hexyloxy)benzamide, 291 mg (4.47 mmol, 6.1 eq) sodium azide and 0.17 mL (1.48

mmol, 2.0 eq) silicon tetrachloride in 20 mL acetonitrile. After 1 h 10 mL toluene was added to increase the solubility of the amide in the reaction medium. Column chromatography ($SiO_2$; Tol:EA:$Et_3N$ = 8:2:1→Tol:EA:HOAc = 8:2:1) afforded 77 mg (0.153 mmol, 21 %) of the product as beige solid. m.p.: 77-79 °C. **$^1$H-NMR** (400 MHz, $CDCl_3$) δ = 7.25 (s, 2H, 2/6-H), 5.62 (s, br, 1H, N*H*), 4.03 (t, $^3J$ = 6.7 Hz, 2H, OC*H₂*), 3.91 (t, $^3J$ = 6.5 Hz, 4H, OC$H_2$), 1.91 – 1.59 (m, 6H, $OCH_2CH_2$), 1.58 – 1.10 (m, 23H, C$H_2$), 0.93 – 0.82 (m, 9H, C$H_3$), 0.81 (t, $^3J$ = 7.0 Hz, 3H, C$H_3$). **$^{13}$C-NMR** (101 MHz, $CDCl_3$) δ = 153.84, 153.81, 140.33, 119.02, 105.60, 105.57, 73.98, 69.80, 69.38, 38.73, 32.85, 31.86, 31.68, 30.36, 29.85, 29.53, 29.36, 29.03, 26.65, 25.84 (2C), 25.82, 23.26, 22.81, 22.73, 14.28, 14.21, 14.15, 10.91. **IR** (neat): ṽ [1/cm] = 2952 s, 2928 s, 2859 s, 2621 m, 1588 m, 1498 s, 1450 s, 1381 m, 1308 m, 1242 s, 1116 ss, 995 m, 836 m, 748 m. **HRMS-ES(+):** [M+H]+: calc.: 503.3956 found: 503.3943.

**3,7,11-Tris(3-((4-ethyloctyl)oxy)-4,5-bis(hexyloxy)phenyl)tris([1,2,4]triazolo)[4,3-a:4',3'-c:4'',3''-e][1,3,5]triazine *t*-79**

This compound was prepared according to general procedure DL-TTT from 65 mg (0.129 mmol, 3.1 eq) 1*H*-5-(3-((4-ethyloctyl)oxy)-4,5-bis(hexyloxy)phenyl)tetrazole **189**, 0.05 mL (0.4 mmol, 5.0 eq) 2,4,6-collidine and 7.7 mg (0.042 mmol, 1.0 eq) cyanuric chloride in 10 mL xylene. Column chromatography ($SiO_2$ + 2 cm $Al_2O_3$; Tol:EA = 40:1) afforded 33 mg (0.022 mmol, 52 %) of the product as colorless solid.

$^1$H-NMR (400 MHz, $CDCl_3$) δ = 7.43 (s, 6H, 2/6-H-Ph), 4.21 – 3.87 (m, 18H, OC$H_2$), 1.92 – 1.66 (m, 18H, $OCH_2CH_2$), 1.54 – 1.11 (m, H, C$H_2$, C*H*), 0.99 – 0.71 (m, 36H, C$H_3$). $^{13}$C-NMR (101 MHz, $CDCl_3$) δ = 153.20, 153.16, 151.38, 141.44, 140.72, 118.31, 108.90, 108.82, 73.80, 69.90, 69.48, 38.80, 32.92, 31.92, 31.74, 30.47, 29.60, 29.44, 29.08, 26.75, 25.95, 25.93, 25.89, 23.29, 22.86, 22.78, 14.31, 14.26, 14.20, 10.99. IR (neat): ṽ [1/cm] = 3115 w, 2955 s, 2925 s, 2858 s, 1738 w, 1581 ss, 1487 s, 1466 s, 1432 s, 1380 s, 1338 s, 1281 m, 1229 s, 1112 ss, 1005 m, 926 m, 872 m, 837 m, 795 m, 719 m, 704 m, 687 w, 673 w. **HRMS-ES(+):** [M+H]+: calc.: 1499.1395 found: 1499.1398.

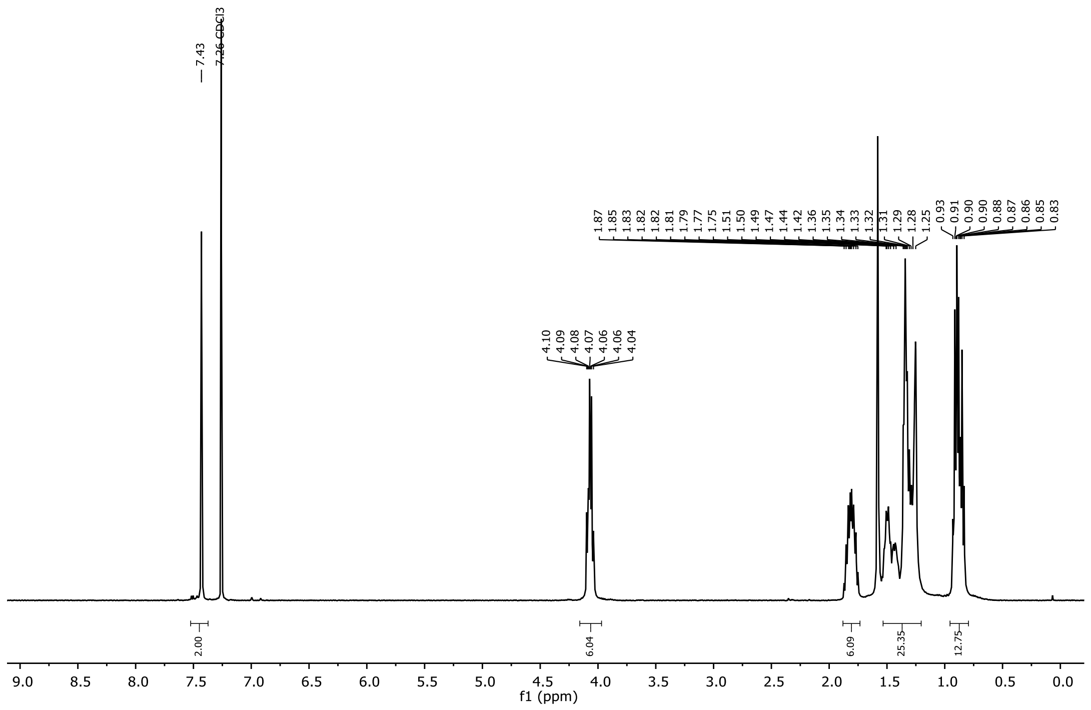

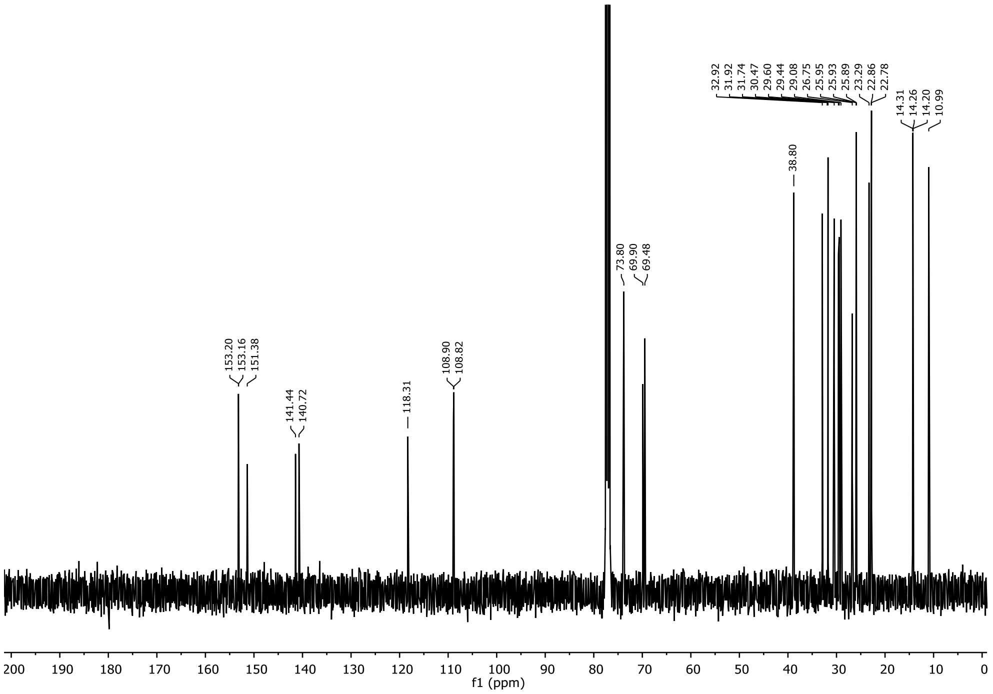


$^{1}$H- and [13] C-NMR spectra of 3,7,11-Tris(3-((4-ethyloctyl)oxy)-4,5-bis(hexyloxy)phenyl)tris([1,2,4]triazolo)[4,3-a:4',3'-c:4'',3''-e][1,3,5]triazine ***t*-79**

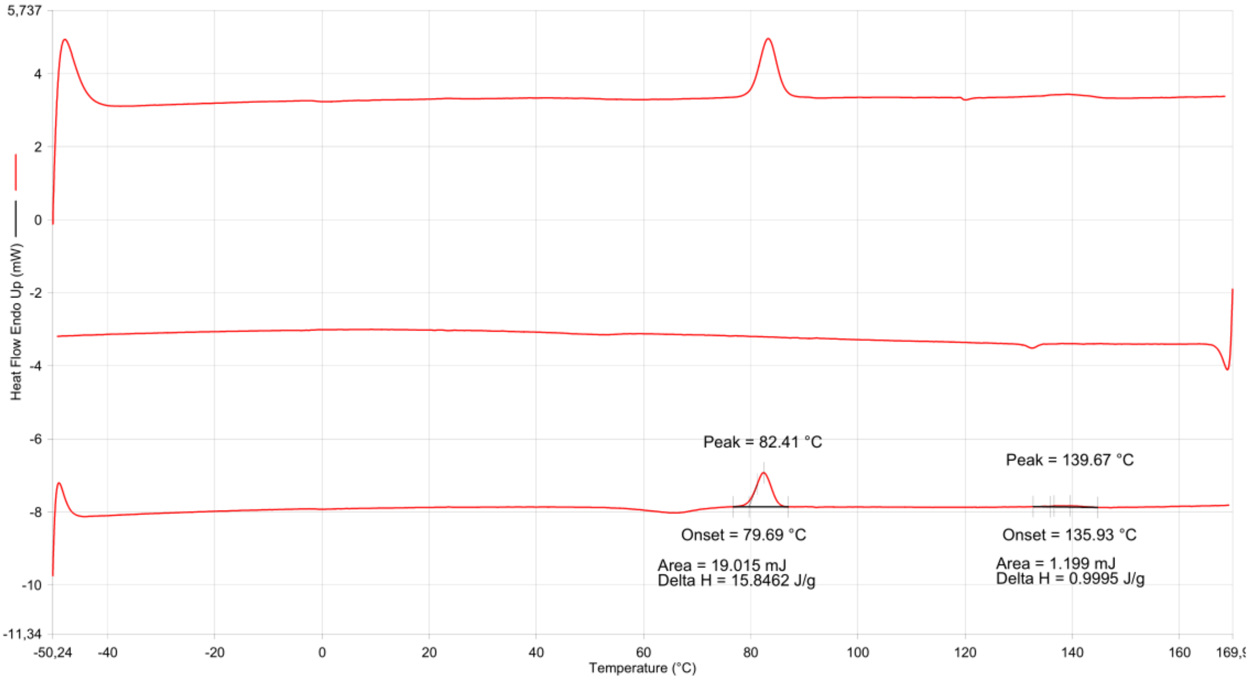


DSC of 3,7,11-Tris(3-((4-ethyloctyl)oxy)-4,5-bis(hexyloxy)phenyl)tris([1,2,4]triazolo)[4,3-a:4',3'-c:4'',3''-e][1,3,5]triazine ***t*-79**

**5-Chloro-3-(3-chloro-4-(hexyloxy)phenyl)-*1H*-1,2,4-triazole *r*-80** 780 mg (3mmol) 5-(4-Hexyloxyphenyl)-3-amino-*2H*-1,2,4-triazole was dissolved in glacial acetic acid (30 mL) and hydrochloric acid (10 mL) and cooled to 0°C. A solution of 550 mg (8 mmol) $NaNO_2$ in 4 mL water was added dropwise while stirring. After 1.5 h at -5°C, the suspension became clear. After 16 h at ambient temperature, $Na_2CO_3$ was added until pH = 6 and the mixture was extracted with ethyl acetate (2* 50 mL). The organic layers were washed with brine, dried ($MgSO_4$), concentrated and recrystallized from ethyl acetate to yield 441 mg (41 %) of a colorless solid with m.p. = 145 °C.

**$^1$H-NMR** (400 MHz, $CDCl_3$): $\delta$ = 0.84-0.87 (m, 3H, Alkoxy-$CH_3$); 1.17-1.30 (m, 4H, Alkoxy-$CH_2$); 1.31-1.43 (m, 2H, Alkoxy-$CH_2$); 1.64-1.80 (m, 2H, Alkoxy-$CH_2$); 3.97-4.02 (m, 2H, Ar-O-$CH_2$); 6.91 (d, $^3J$=8.7 Hz, C(4)-H); 7.78 (dd, $^3J$=8.7 Hz, $^4J$=2.1 Hz, C(3)-H); 7.97 (d, $^4J$=2.1 Hz, C(7)-H). **$^{13}$C-NMR** (75 MHz, $CDCl_3$): $\delta$ = 14.1 (Alkoxy-$CH_3$); 22.6, 25.6, 28.9, 31.5 (Alkoxy-$CH_2$); 69.4 (Ar-O-$CH_2$); 113.1 (C(3)); 118.9 (C(2)); 123.8 (C(6)); 126.2 (C(4)); 128.4 (C(7)); 151.9 (C(1a)); 156.4 (C(5)); 156.9 (C(1b)). **IR** (KBr): $\tilde{\nu}$ [$cm^{-1}$]= 3145; 3082;2930; 2856; 1608; 1493; 1459; 1297; 1141; 1062; 994; 883; 826; 739. **FD-MS:** m/z (%)= 313.1 (100), 314.1 (15), 315.1 (62), 316.1 (9) $[M]^{+\cdot}$. **EA:** $C_{14}H_{17}Cl_2N_3O$ (314.22) calcd.: 53.52%C 5.45 %H 13.37%N found: 53.37%C 5.37 %H 13.29%N.

## XRD pattern of TTTs

100 °C

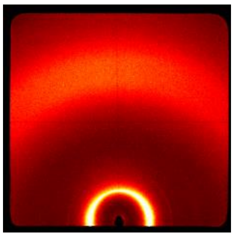

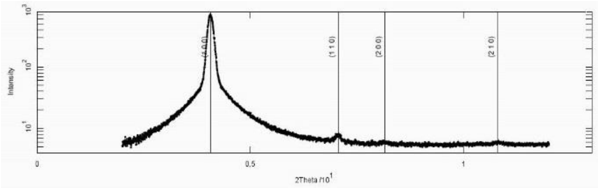

$a_{hex}$= 25,0 Å

125 °C

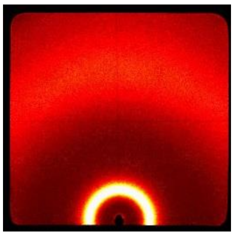

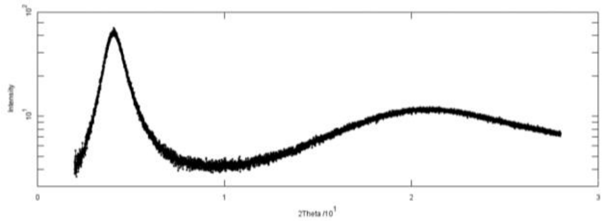

25 °C

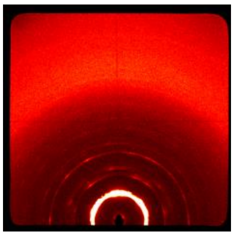

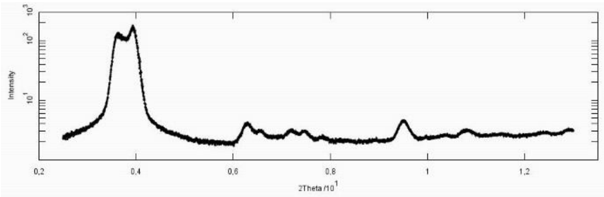

70 °C

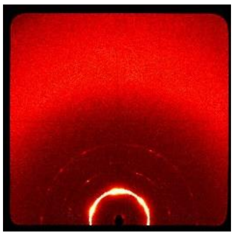

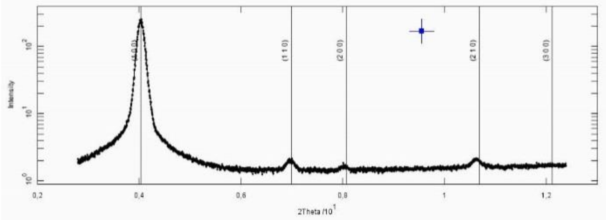

$a_{hex}$= 25,3 Å

Diffractograms of ***t*-79** in oriented filament at different temperatures

25 °C

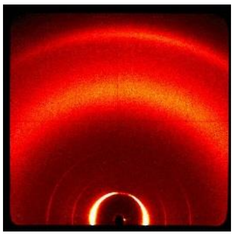

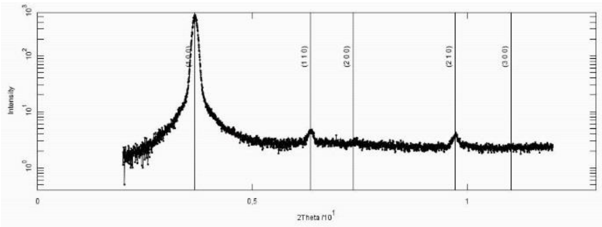

$a_{hex}$= 27,8 Å

*d*= 3,4 Å

130 °C

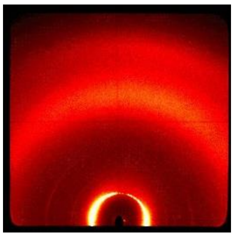

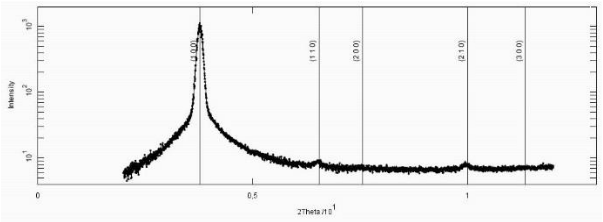

$a_{hex}$= 27,0 Å

*d*= 3,7 Å

100 °C

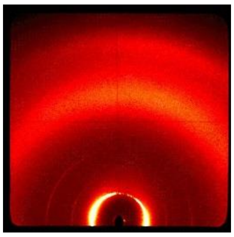

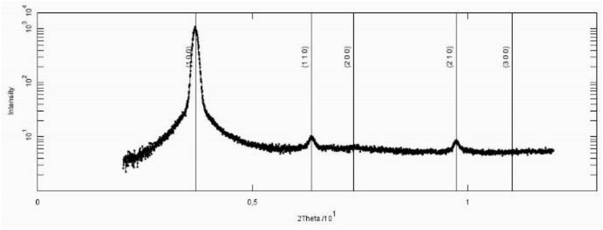

$a_{hex}$= 27,3 Å

*d*= 3,6 Å

25 °C

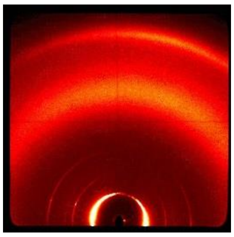

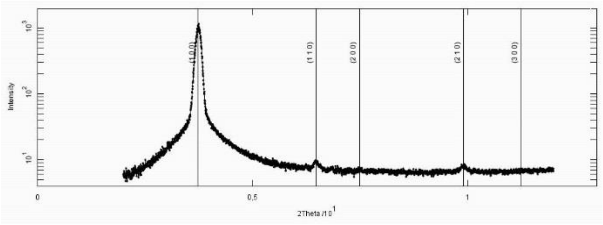

$a_{hex}$= 27,8 Å

*d*= 3,4 Å

Diffractograms of ***t*-60** in oriented filament at different temperatures

25 °C

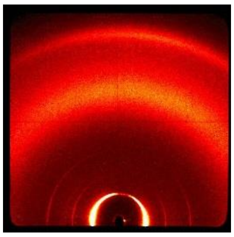

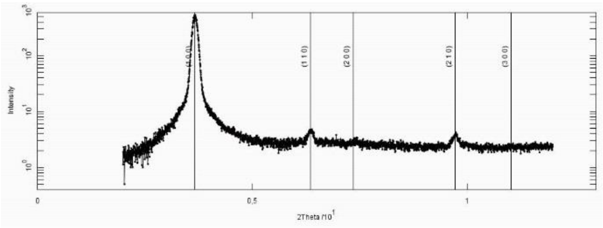

$a_{hex}$= 27,8 Å

*d*= 3,4 Å

130 °C

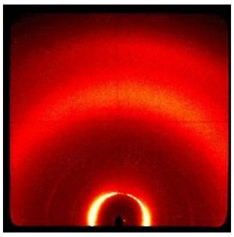

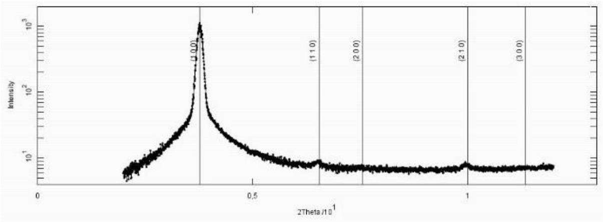

$a_{hex}$= 27,0 Å

*d*= 3,7 Å

X-ray diffractograms of ***t*-10** at 25 °C and 130 °C.

25 °C

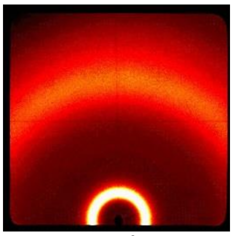

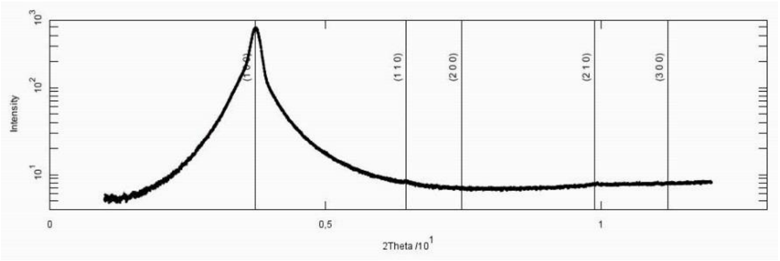


$a_{hex}$= 27.3 Å

Diffractograms of ***t*-64** in oriented filament at 25 °C

25 °C

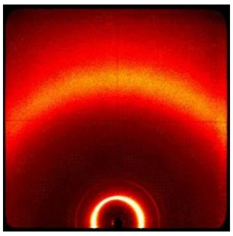

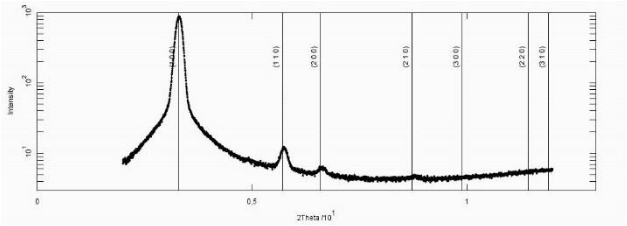

$a_{hex}$= 31,0 Å

49 °C

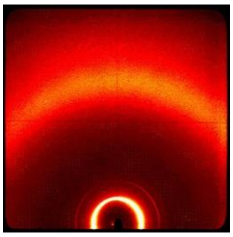

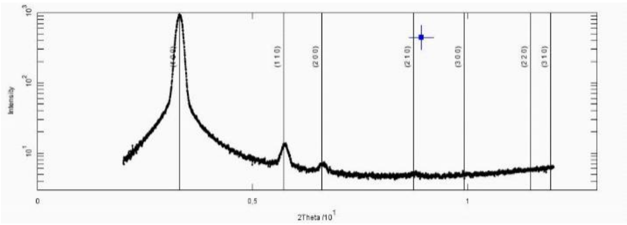

$a_{hex}$= 30,9 Å

71 °C

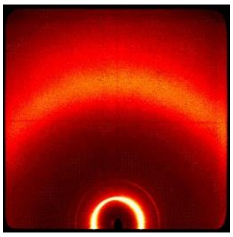

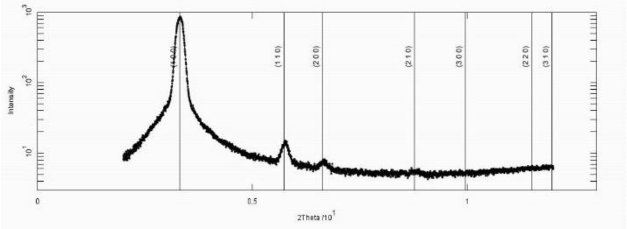

$a_{hex}$= 30,8 Å

25 °C

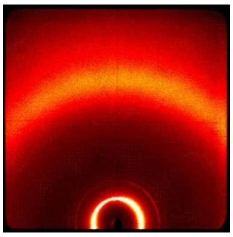

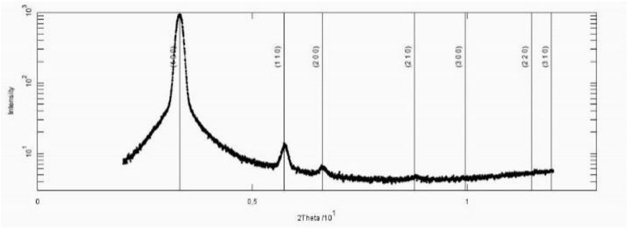

$a_{hex}$= 30,8 Å

Diffractograms of ***t*-66** in oriented filament at different temperatures

25 °C 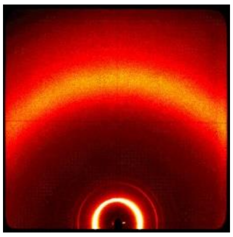 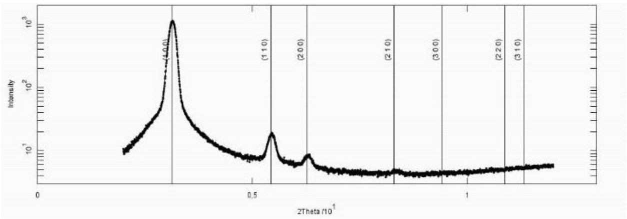

$a_{hex}$= 32,5 Å

49 °C 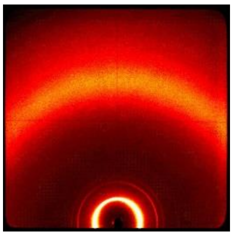 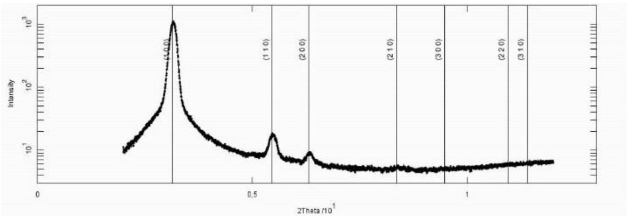

$a_{hex}$= 32,3 Å

71 °C 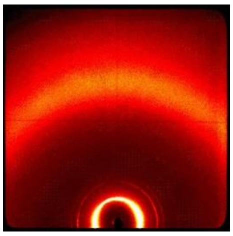 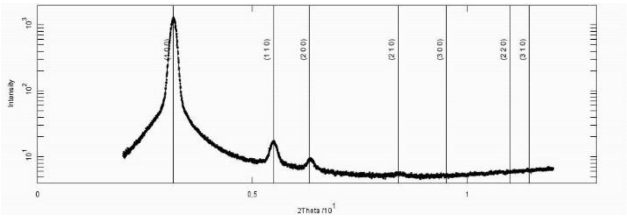

$a_{hex}$= 32,2 Å

25 °C 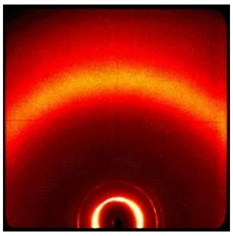 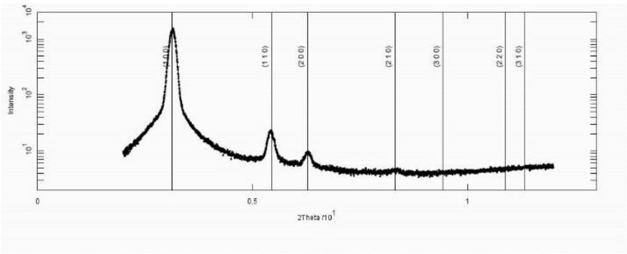

$a_{hex}$= 32,5 Å

Diffractograms of ***t*-67** in oriented filament at different temperatures

25 °C 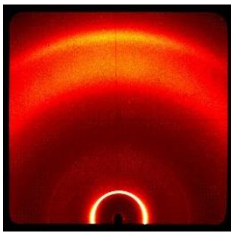 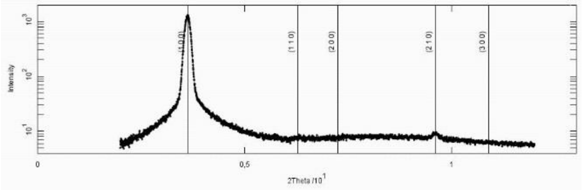

$a_{hex}$= 28,1 Å

$d$= 3,49 Å

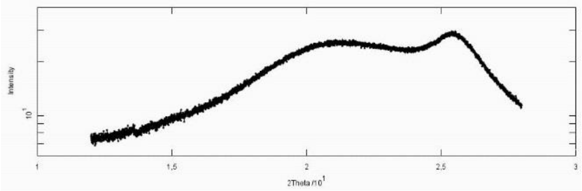

80 °C

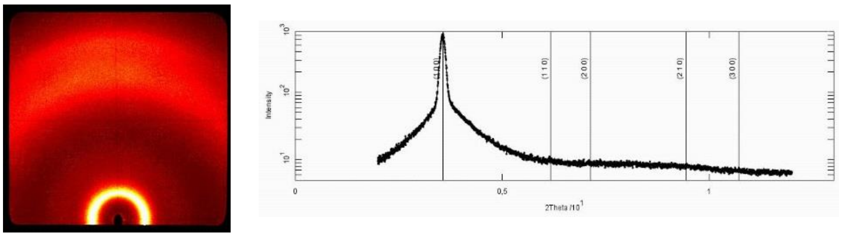

$a_{hex}$= 28,6Å

Diffractograms of ***r*-33** in oriented filament at different temperatures


References

[1] T. Rieth, N. Röder, M. Lehmann, H. Detert, *Chemistry-A European Journal*, **2018**, *24*, 93-96

[2] S. Glang, D. Borchmann, T. Rieth, H. Detert, Adv. Sci. Technol. **2013**, 77, 118-123.

[3] R. Cristiano, H. Gallardo, A. J. Bortoluzzi, I. H. Bechtold, C. E. M. Campos, R. L. Longo, Chem. Commun. 2008, 41, 5134-5136

[4] T. Rieth, N. Röder, M. Lehmann, H. Detert, Chemistry **2018**, *24*, 93-96.

[5] K. Herget, D. Schollmeyer, H. Detert, Acta Cryst. **2013**, E69 o365-o366.
